# Once-Weekly Semaglutide in Adults With Alcohol Use Disorder: A Randomized Clinical Trial

**DOI:** 10.1001/jamapsychiatry.2024.4789

**Published:** 2025-02-12

**Authors:** Christian S. Hendershot, Michael P. Bremmer, Michael B. Paladino, Georgios Kostantinis, Thomas A. Gilmore, Neil R. Sullivan, Amanda C. Tow, Sarah S. Dermody, Mark A. Prince, Robyn Jordan, Sherry A. McKee, Paul J. Fletcher, Eric D. Claus, Klara R. Klein

**Affiliations:** 1 Department of Population and Public Health Sciences and Institute for Addiction Science, Keck School of Medicine, University of Southern California, Los Angeles; 2 Department of Psychiatry, University of North Carolina at Chapel Hill; 3 Bowles Center for Alcohol Studies, University of North Carolina at Chapel Hill; 4 Department of Psychiatry and Neuroscience, University of North Carolina at Chapel Hill; 5 Department of Psychology, Toronto Metropolitan University, Toronto, Ontario, Canada; 6 Department of Psychiatry and the Behavioral Sciences, Keck School of Medicine, University of Southern California, Los Angeles; 7 Department of Psychiatry, Yale School of Medicine, New Haven, Connecticut; 8 Campbell Family Mental Health Research Institute, Centre for Addiction and Mental Health, Toronto, Ontario, Canada; 9 Department of Psychology, University of Toronto, Toronto, Ontario, Canada; 10 Department of Psychiatry, University of Toronto, Toronto, Ontario, Canada; 11 Department of Biobehavioral Health, The Pennsylvania State University, University Park; 12 Department of Medicine, University of North Carolina School of Medicine, Chapel Hill

## Abstract

**Importance:**

Preclinical, observational, and pharmacoepidemiology evidence indicates that glucagon-like peptide 1 receptor agonists (GLP-1RAs) may reduce alcohol intake. Randomized trials are needed to determine the clinical significance of these findings.

**Objective:**

To evaluate the effects of once-weekly subcutaneous semaglutide on alcohol consumption and craving in adults with alcohol use disorder (AUD).

**Design, Setting, and Participants:**

This was a phase 2, double-blind, randomized, parallel-arm trial involving 9 weeks of outpatient treatment. Enrollment occurred at an academic medical center in the US from September 2022 to February 2024. Of 504 potential participants assessed, 48 non–treatment-seeking participants with AUD were randomized.

**Intervention:**

Participants received semaglutide (0.25 mg/week for 4 weeks, 0.5 mg/week for 4 weeks, and 1.0 mg for 1 week) or placebo at weekly clinic visits.

**Main Outcomes and Measures:**

The primary outcome was laboratory alcohol self-administration, measured at pretreatment and posttreatment (0.5 mg/week). Secondary and exploratory outcomes, including prospective changes in alcohol consumption and craving, were assessed at outpatient visits.

**Results:**

Forty-eight participants (34 [71%] female; mean [SD] age, 39.9 [10.6] years) were randomized. Low-dose semaglutide reduced the amount of alcohol consumed during a posttreatment laboratory self-administration task, with evidence of medium to large effect sizes for grams of alcohol consumed (β, −0.48; 95% CI, −0.85 to −0.11; *P* = .01) and peak breath alcohol concentration (β, −0.46; 95% CI, −0.87 to −0.06; *P* = .03). Semaglutide treatment did not affect average drinks per calendar day or number of drinking days, but significantly reduced drinks per drinking day (β, −0.41; 95% CI, −0.73 to −0.09; *P* = .04) and weekly alcohol craving (β, −0.39; 95% CI, −0.73 to −0.06; *P* = .01), also predicting greater reductions in heavy drinking over time relative to placebo (β, 0.84; 95% CI, 0.71 to 0.99; *P* = .04). A significant treatment-by-time interaction indicated that semaglutide treatment predicted greater relative reductions in cigarettes per day in a subsample of individuals with current cigarette use (β, −0.10; 95% CI, −0.16 to −0.03; *P* = .005).

**Conclusions and Relevance:**

These findings provide initial prospective evidence that low-dose semaglutide can reduce craving and some drinking outcomes, justifying larger clinical trials to evaluate GLP-1RAs for alcohol use disorder.

**Trial Registration:**

ClinicalTrials.gov Identifier: NCT05520775

## Key Points

**Question:**

Does the glucagon-like peptide 1 (GLP-1) receptor agonist semaglutide reduce alcohol consumption and craving in adults with alcohol use disorder (AUD)?

**Findings:**

In this randomized clinical trial, relative to placebo, low-dose semaglutide reduced the amount of alcohol consumed during a posttreatment laboratory self-administration procedure. Over 9 weeks of treatment, semaglutide led to reductions in some but not all measures of weekly consumption, significantly reduced weekly alcohol craving relative to placebo, and led to greater relative reductions in cigarettes per day in a subgroup of participants with current cigarette use.

**Meaning:**

These results justify larger clinical trials of incretin therapies for AUD.

## Teaser

This randomized clinical trial assesses the effects of subcutaneous semaglutide on alcohol consumption in adults with alcohol use disorder.

## Introduction

Alcohol use is a leading modifiable cause of morbidity and mortality, accounting for an estimated 4% to 5% of disease burden and 2.6 million deaths per year globally.^1,2^ Alcohol is causally linked to more than 200 medical and disability conditions,^2^ with increased risks of common diseases (including cardiovascular disease, liver disease, and cancers) accounting for a large proportion of alcohol-related morbidity.^1,3,4^ Increased incidence of alcohol-related liver disease since 2020 has contributed to a 29% increase in alcohol-related mortality in the US since 2016-2017.^3,5,6^ An estimated 178 000 US deaths per year are alcohol attributable,^3^ with further increases in rates of alcohol-related disease projected.^4,7,8^

While roughly 29% and 11% of US adults meet lifetime and past-year criteria for alcohol use disorder (AUD), respectively,^9,10^ less than 10% of those with AUD report past-year treatment^10^ and less than 2% receive pharmacotherapy,^11^ defining one of the largest known health care treatment gaps.^12^ Underutilization of AUD medications is attributed to multiple factors, including few Food and Drug Administration (FDA)–approved therapies, limited awareness of these medications, and barriers related to stigma.^12,13^ Reduced alcohol intake, irrespective of abstinence, is associated with improved health outcomes.^14,15,16^ Medications that facilitate reductions in alcohol use while achieving broad clinical uptake would fill a critical unmet need.

Glucagon-like peptide 1 (GLP-1) receptor agonists (GLP-1RAs) are incretin mimetic therapies with exceptional efficacy for the treatment of diabetes and obesity.^17,18,19^ Semaglutide, a long-acting GLP-1RA with superior efficacy to older GLP-1RA medications, received FDA approval for diabetes in 2017 and obesity in 2021.^20^ Rapidly increasing prescription rates have been accompanied by reports of reductions in alcohol use and craving during treatment.^21,22^ These observations were predated by substantial preclinical evidence that GLP-1RAs reduce voluntary alcohol consumption and attenuate alcohol reinforcement,^23,24,25,26^ suggesting potential clinical applications of GLP-1RAs for AUD.^26,27,28,29^ Although experimental evidence for semaglutide-related reductions in alcohol intake remains specific to nonhuman studies,^30,31,32,33^ off-label prescribing for AUD is already reported, necessitating clinical trials.^21,34^ This prospective phase 2 randomized clinical trial evaluated the effects of once-weekly subcutaneous semaglutide in non–treatment-seeking adults with AUD.

## Methods

### Trial Design

This phase 2 clinical trial used a hybrid design, combining clinical outpatient and human laboratory components, to evaluate the effects of semaglutide in non–treatment-seeking adults with AUD. The outpatient sequence involved 9 weeks of medication or placebo (weeks 1-9) and a final assessment visit (week 10). Objective laboratory alcohol self-administration was assessed at pretreatment (prior to week 1) and between weeks 8 and 9 (at 0.5 mg/week). Other outcomes were assessed prospectively at weekly clinic visits. This investigator-initiated trial was conducted at the University of North Carolina (UNC)–Chapel Hill School of Medicine with oversight from the UNC institutional review board and under an investigational new drug exemption granted by the FDA. The trial protocol is in Supplement 1. All participants provided written informed consent.

### Participant Sample

Non–treatment-seeking adults with AUD were recruited via online and public advertisements. Primary inclusion criteria included age 21 to 65 years, reporting past-year *DSM-5* criteria for AUD, past-month endorsement of more than 7 (women) or more than 14 (men) standard drinks in a week with 2 or more heavy drinking episodes (4 or more drinks for women and 5 or more for men), and ability to attend weekly clinic visits and pretreatment and posttreatment laboratory sessions. Key exclusion criteria included currently seeking treatment for alcohol problems or actively attempting to reduce consumption; past use of GLP-1 receptor agonists; weight loss medications; body mass index (BMI, calculated as weight in kilograms divided by height in meters squared) less than 23; past-year substance use disorder other than AUD, tobacco use disorder, or mild cannabis use disorder; recent (30-day) use of illicit drugs except cannabis; history of diabetes, and current medical or neurological illness precluding participation based on physician judgement. See the eMethods in Supplement 2 for full eligibility criteria. Enrollment lasted from September 2022 to February 2024.

### Study Procedures

Following written informed consent and eligibility confirmation, participants completed a pretreatment alcohol self-administration session before the week 1 medication visit. Participants then completed 8 additional medication or placebo visits (weeks 2-9), a posttreatment alcohol self-administration session (scheduled between weeks 8 and 9), and a discharge visit (week 10, no medication). See the eMethods in Supplement 2 for additional procedural details.

### Intervention

Subcutaneous semaglutide was administered according to standard practice, with dose increases every 4 weeks. To maximize safety and feasibility, only the 2 lowest dose sequences (0.25 mg/week for weeks 1-4 and 0.5 mg/week for weeks 5-8) were used prior to primary outcome collection (between weeks 8 and 9). To obtain additional safety and prospective data, participants received a final dose increase (1.0 mg) at week 9, contingent on tolerability. The week 9 dose was treated as flexible and could be held at 0.5 mg or deferred for safety or practical reasons, based on physician judgement. Participants, investigators, and outcome assessors were blind to condition (see the eMethods in Supplement 2 for additional placebo and blinding information).

### Outcomes Assessment

#### Laboratory Alcohol Self-Administration

Objective self-administration (the primary outcome) was assessed by embedding human laboratory procedures in the trial design. Prior work supports the sensitivity of laboratory self-administration to detect pharmacotherapy effects.^35,36^ A standardized procedure^37^ was used to estimate voluntary alcohol consumption and ability to delay drinking. Participants were presented with their preferred beverage and brand and could elect to delay drinking for up to 50 minutes for monetary reinforcement. Thereafter, participants were instructed to consume at their preferred pace to achieve preferred effects over 120 minutes.^37^ The available alcohol amount (in grams) was determined with anthropometric formulas based on a prespecified maximum breath alcohol concentration (BrAC) limit. BrAC was measured every 30 minutes after drinking onset. Estimated volume consumed (g-ETOH) and peak BrAC served as co–primary outcome measures. See the eMethods in Supplement 2 for additional details.

#### Weekly Consumption and Craving

Weekly consumption was assessed using calendar-based methods^38^ supplemented with daily logs to facilitate recall. Outcomes included average drinks per calendar day (registered secondary outcome), with additional quantity and frequency outcomes common to AUD trials (drinks per drinking day, number of heavy drinking days, number of drinking vs abstinent days), and weekly craving^39^ examined as exploratory and hypothesis-generating outcomes. The proportion of participants with zero heavy drinking days served as an exploratory outcome relevant to FDA end points.^40^ Cigarettes per day (registered secondary outcome) was assessed during calendar-based assessments.^38^ See the eMethods in Supplement 2 for additional information.

#### Additional Clinical and Safety Outcomes

Body weight and systolic and diastolic blood pressure were assessed weekly. Side effects, adverse events, and depression symptoms were queried weekly using standardized instruments.^41,42^

### Statistical Analyses

Power analyses informed a target sample of 48 randomized participants (see the eMethods in Supplement 2 for rationale and full analysis plan). Primary, secondary, and exploratory outcomes were evaluated with linear mixed models and full information maximum likelihood estimation to accommodate missing data (lme4 package version 35.5;^43^ RStudio version 2024.04.2 + 764 [R Foundation]).

Models of weekly outcomes used intention-to-treat principles (all 48 randomized participants included). Primary intention-to-treat analyses of laboratory outcomes consisted of residualized change models testing medication effects on posttreatment self-administration (g-ETOH and peak BrAC; session 2) controlling for pretreatment levels (session 1). These models estimated medication effects on quantitative consumption during self-administration. Some participants elected not to engage in alcohol consumption, resulting in the presence of missing data for g-ETOH and peak BrAC. Thus, those who opted not to engage in the task contributed data to the intention-to-treat analyses for weekly drinking outcomes and non–self-administration laboratory outcomes (eg, delay time), but were modeled as having missing data for analyses of g-ETOH and peak BrAC, resulting in a sample of 25 with complete data for residualized change analyses. An exploratory model also tested mean BrAC across the session (see the eMethods in Supplement 2 for full details and rationale for statistical models).

Intention-to-treat linear mixed models of weekly outcomes included a random effect for participant, a fixed effect of time (study week, within-subject factor), medication group (semaglutide vs placebo, the primary effect of interest), treatment-by-time interaction, and covariates (see the eMethods in Supplement 2 for full model details). A preplanned linear mixed model examining cigarettes per day (registered secondary outcome) was limited to those participants reporting cigarette smoking at baseline (n = 13; 7 in the placebo group and 6 in the semaglutide group), with restricted maximum likelihood estimation given the small sample size.

Because preliminary estimates of effect size are needed to inform future trials of GLP-1RAs, medication effect size estimates are reported in all models (β for linear mixed models and residualized change models; small effect: β = 0.10, medium: β = 0.30, large: β = 0.50). To compare effect sizes over the 2 dosage periods, Cohen *d* values computed for the monthly intervals corresponding with dosage (0.25 mg/week in weeks 1-4 and 0.5 mg/week in weeks 5-8) are presented for descriptive purposes, using percentage change from baseline to facilitate interpretation across outcomes. Cohen *d* (small effect: *d* = 0.20, medium: *d* = 0.50; large: *d* = 0.80) was computed in the R *lsr* package version 0.5.2.

## Results

### Sample Description and Retention

**Figure 1.**
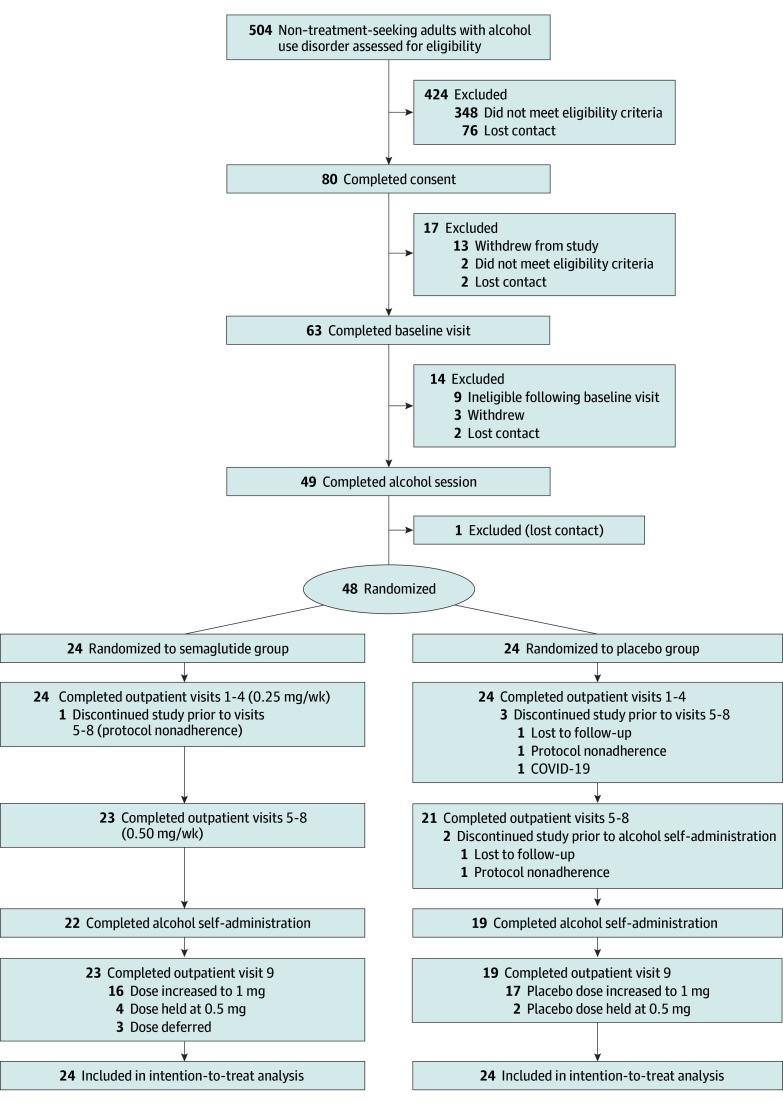
CONSORT Flow Diagram

Of the 48 participants randomized, 34 (71%) were female and 14 (29%) were male. The mean (SD) age was 39.9 (10.6) years. Most had BMI greater than 30 (n = 27) or 25.0-29.9 (n = 20); 1 participant had BMI less than 24.9. On average, participants endorsed moderate AUD severity (Table). Figure 1 presents the CONSORT diagram. Overall, 42 of 48 participants completed outpatient visits through week 9. All participants who completed posttreatment alcohol sessions received the required 4 doses at 0.5 mg/week. See the eResults in Supplement 2 for full retention details.

**Table.** Pretreatment Characteristics by Treatment Group for All Randomized Participants

Characteristic	Mean (SD)		
	Placebo	Semaglutide	Total
Randomized, No.	24	24	48
Sex, No.(%)			
Female	17 (71)	17 (71)	34 (71)
Male	7 (29)	7 (29)	14 (29)
Age, y	39.0 (10.9)	40.6 (10.5)	39.9 (10.6)
Race, No.(%)^a^			
Asian	2 (8)	0	2 (4)
Black/African American	4 (17)	3 (13)	7 (15)
Hawaiian/Pacific Islander	0	0	0
White	18 (75)	21 (88)	39 (81)
Other (unspecified) or multiple	0	0	0
Hispanic ethnicity, No.(%)^a^	2 (8)	2 (8)	4 (8)
AUD symptoms, *DSM-5*	4.3 (2.0)	4.1 (1.5)	4.2 (1.7)
AUDIT	14.2 (6.5)	12.7 (5.6)	13.4 (6.0)
Alcohol consumption^b^			
Drinks/calendar day	3.0 (1.7)	2.7 (1.7)	2.9 (1.7)
Drinks/drinking day	4.5 (2.5)	3.8 (1.8)	4.2 (2.2)
Drinking days	19.6 (5.5)	20.8 (6.8)	20.2 (6.1)
Heavy drinking days	9.8 (5.5)	8.4 (7.9)	9.1 (6.8)
Alcohol craving, PACS score	12.2 (6.5)	11.9 (4.7)	12.0 (5.6)
Current smoking, No.(%)^c^	7 (29)	6 (25)	13 (27)
Cigarettes per day	14.0 (13.5)	8.0 (7.7)	11.2 (11.2)
WHO risk level, No.(%)^d^			
1	5 (21)	8 (33)	13 (27)
2	10 (42)	10 (42)	20 (42)
3	7 (29)	4 (17)	11 (23)
4	2 (8)	2 (8)	4 (8)
Weight, kg	93.1 (15.1)	95.4 (20.9)	94.2 (18.1)
BMI	31.7 (4.5)	32.4 (6.7)	32.1 (5.6)
BMI category, No.(%)			
BMI <30 male	5 (21)	4 (17)	9 (19)
BMI <30 female	4 (17)	8 (33)	12 (25)
BMI ≥30 male	2 (8)	3 (13)	5 (10)
BMI ≥30 female	13 (54)	9 (38)	22 (46)
Blood pressure			
Systolic	127.9 (18.7)	125.1 (16.2)	126.5 (17.3)
Diastolic	85.7 (14.7)	83.7 (10.6)	84.7 (12.7)
Heart rate	78.8 (14.1)	75.0 (10.7)	76.9 (12.6)
HbA_1C_	5.16 (0.35)	5.09 (0.38)	5.13 (0.36)
Depression (CES-D score)	11.7 (10.0)	12.7 (7.4)	12.2 (8.7)

Abbreviations: AUD, alcohol use disorder; AUDIT, Alcohol Use Disorders Identification Test; BMI, body mass index (calculated as weight in kilograms divided by height in meters squared); CES-D, Center for Epidemiologic Studies Depression Scale; HbA_1c_, hemoglobin A1C; PACS, Penn Alcohol Craving Scale; WHO, World Health Organization.

^a^ Race and ethnicity were collected via self-report for purposes of characterizing the sample.

^b^ Alcohol consumption during the 28-day period before baseline visit.

^c^ Defined as participants who smoked at least 1 cigarette per week during the 28-day baseline period.

^d^ WHO risk-level criteria are determined by sex-specific criteria for baseline reported alcohol consumption per week: level 1 = <40 g for male individuals and <20 g for female individuals; level 2 = 40-60 g for male individuals and 20-40 g for female individuals; level 3 = 60-100 g for male individuals and 40-60 g for female individuals; and level 4 = >100 g for male individuals and >60 g for female individuals.

### Laboratory Alcohol Self-Administration

**Figure 2.**
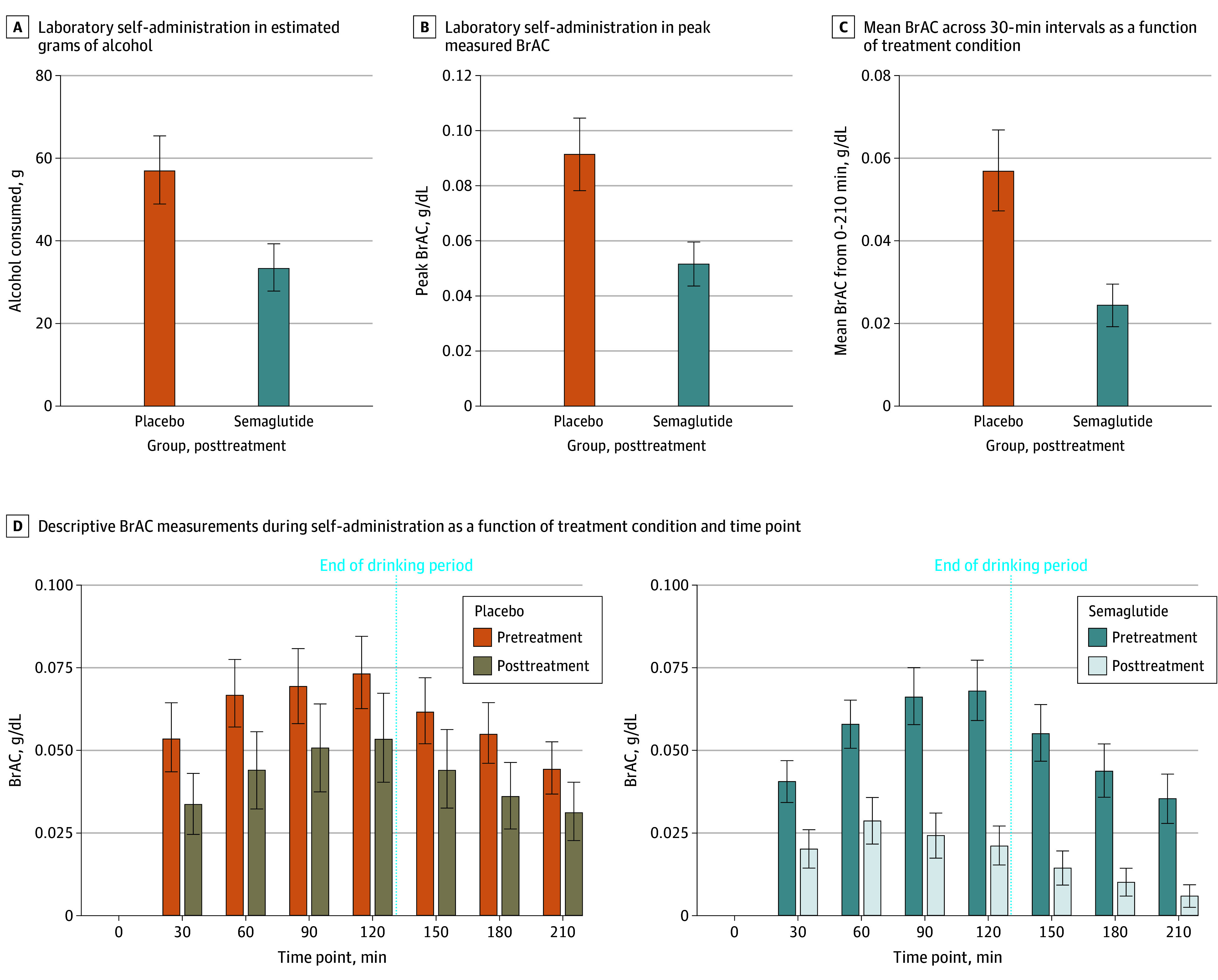
Laboratory Self-Administration Laboratory self-administration, measured in estimated grams of alcohol (A) and peak measured breath alcohol concentration (BrAC; B). Bars depict posttreatment alcohol self-administration following treatment week 8 (0.5 mg/wk), controlling for pretreatment self-administration among those without missing data on the self-administration procedure (n = 25; 12 in the placebo group and 13 in the semaglutide group). C, Mean BrAC measured across 30-minute intervals as a function of treatment condition. D, Descriptive BrAC measurements during self-administration as a function of treatment condition and time point. Bars depict group means and whiskers standard errors.

Results of residualized change models indicated that semaglutide reduced posttreatment laboratory consumption with a medium to large effect size for grams of alcohol consumed (β, −0.48; 95% CI, −0.85 to −0.11; *P* = .01) and peak breath alcohol concentration (β, −0.46; 95% CI, −0.87 to −0.06; *P* = .03) (Figure 2). See the eMethods in Supplement 2 for additional model information and eTable 2 in Supplement 2 for full model results. Descriptive information on BrAC levels across time is depicted in Figure 2; the exploratory analysis of mean BrAC yielded a significant medication effect (β = −0.48, 95% CI, −0.87 to −0.09; *P* = .02). Medication condition did not predict engagement (vs abstinence) in the self-administration task or duration of delay time (eResults in Supplement 2).

### Weekly Outcomes

**Figure 3.**
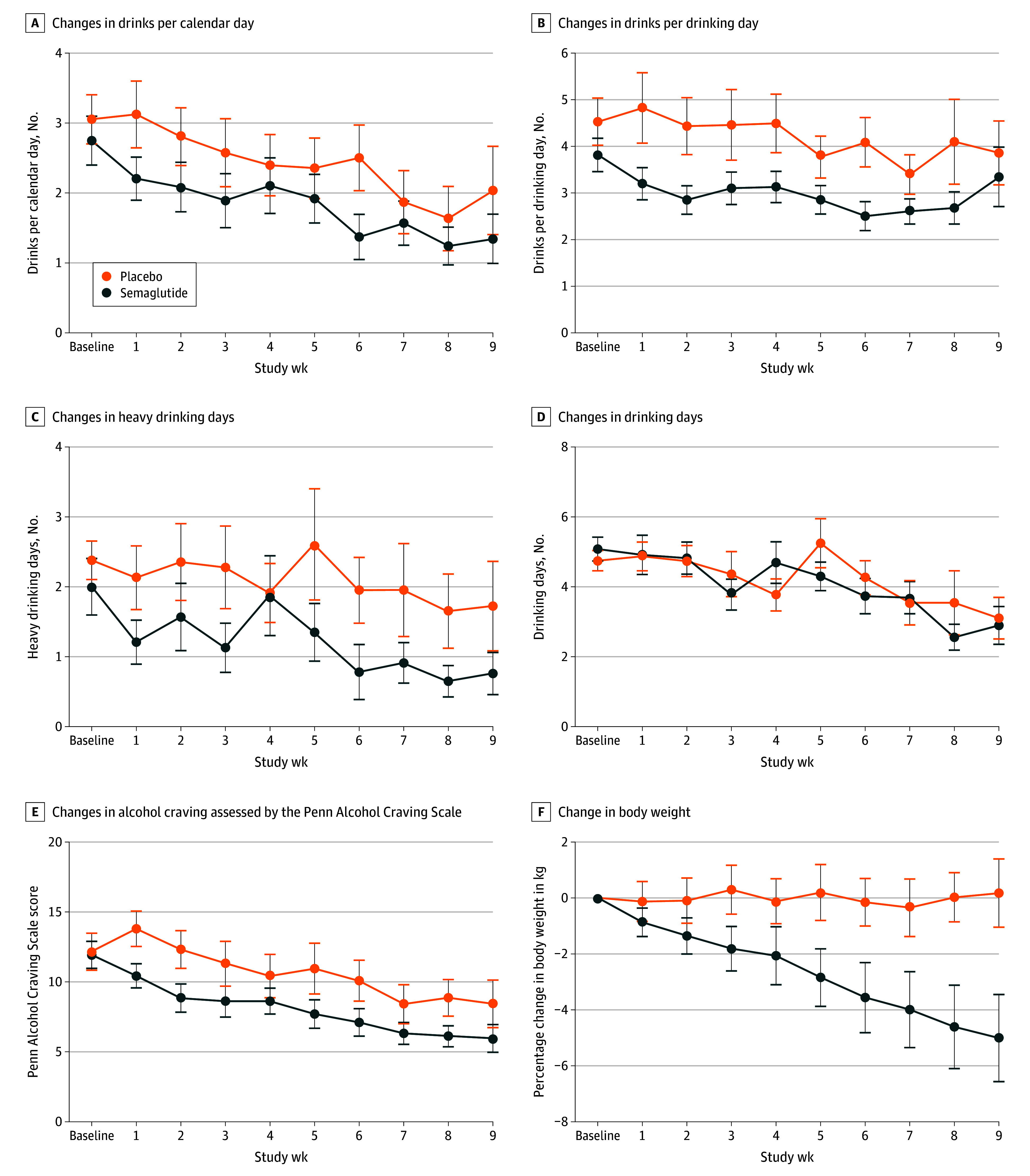
Prospective Changes in Weekly Alcohol Outcomes A, n = 39-48 across weeks (18-24 in the placebo group and 21-24 in the semaglutide group). B, n = 31-48 (15-24 in the placebo group and 16-24 in the semaglutide group). C, n = 39-48 (18-24 in the placebo group and 21-24 in the semaglutide group). D, n = 39-48 (18-24 in the placebo group and 21-24 in the semaglutide group). E, n = 41-48 (19-24 in the placebo group and 22-24 in the semaglutide group). F, n = 41-48 (19-24 in the placebo group and 22-24 in the semaglutide group). Alcohol craving was measured via the Penn Alcohol Craving Scale (0-30). Data points depict group means and standard errors for weekly measurements of the outcome. Medication commenced at week 1. Weekly time points indicate data collected at the end of the week following that week’s injection (eg, week 1 drinking data were measured at week 2 and reflect the week following the week 1 injection).

Figure 3 shows prospective changes in weekly outcomes. Medication effects were nonsignificant for drinks per calendar day (β, −0.27; 95% CI, −0.63 to 0.09; *P* = .17) and significant for drinks per drinking day (β, −0.41; 95% CI, −0.73 to −0.09; *P* = .04). A significant treatment-by-time interaction indicated greater reductions in heavy drinking days over time in the semaglutide group relative to the placebo group (β, 0.84; 95% CI, 0.71 to 0.99; *P* = .04). There was no evidence that semaglutide altered number of drinking vs abstinent days (β, 0.90; 95% CI, 0.73 to 1.12; *P* = .89). Semaglutide significantly reduced weekly craving (β, −0.39; 95% CI, −0.73 to −0.06; *P* = .01). The analysis among individuals reporting current cigarette smoking yielded a significant time-by-treatment interaction (β, −0.10; 95% CI, −0.16 to −0.03; *P* = .005), indicating relatively greater declines in cigarettes per day in the semaglutide vs placebo group over time (eFigure 2 in Supplement 2; eTable 3 in Supplement 2 presents all model results for weekly outcomes).

**Figure 4.**
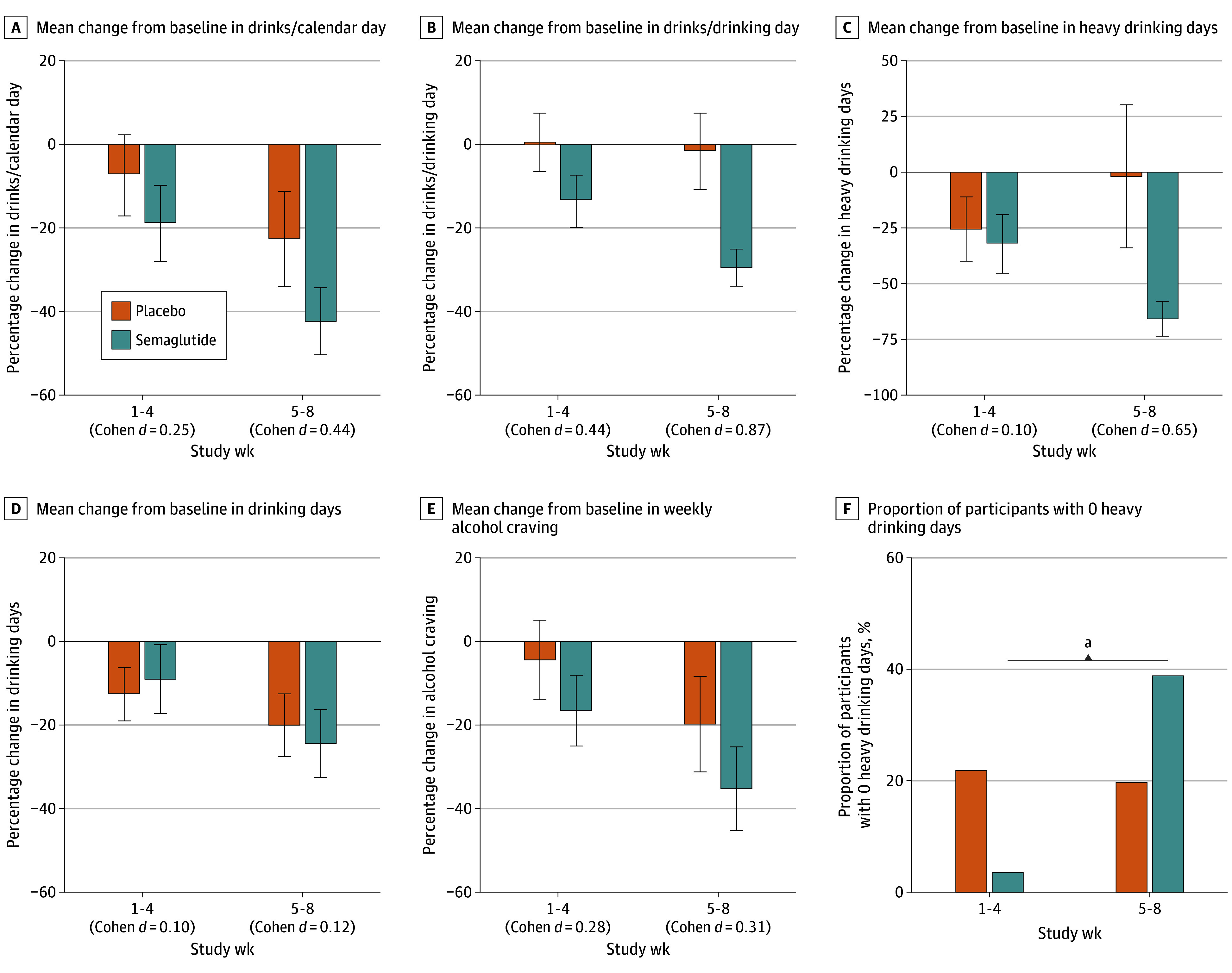
Medication Group Differences in Weekly Drinking and Craving as a Function of Treatment Period A-E, Bars depict group means for changes from baseline on alcohol consumption and craving outcomes during treatment weeks 1-4 (0.25 mg/wk) and 5-8 (0.5 mg/wk) and whiskers indicate standard errors. Effect size estimates (Cohen *d*) reflect medication vs placebo group differences on the outcomes presented. Conventional benchmarks are *d* = 0.20 for a small effect, *d* = 0.5 for a medium effect, and *d* = 0.8 for a large effect, Numbers of participants across weeks were 43-47 for all models (placebo: 23 in weeks 1-4 and 20 in weeks 5-8; semaglutide: 24 in weeks 1-4 and 23 in weeks 5-8). For visual presentation, panel C excludes the data from 1 participant with an extreme outlier >4 SD above the mean. F, Proportion of participants with zero heavy drinking days by treatment condition and dose (weeks 1-4, 0.25 mg/wk; weeks 5-8, 0.5 mg/wk). ^a^*P* < .005.

Figure 4A-E depicts monthly changes from baseline with estimated effect sizes. Medication effect sizes were mostly small (by convention, Cohen *d* = 0.20) through week 4 and increased during weeks 5 to 8, with large effect sizes (defined as *d* = 0.80) observed for drinks per drinking day and heavy drinking days. Comparing the proportion of participants with zero heavy drinking days by group and treatment period (Figure 4F) showed that the likelihood of zero heavy drinking days increased significantly from weeks 1 through 4 to 5 through 8 in the semaglutide group (*z* score, −2.93; *P* = .003), with no other significant comparisons.

### Weight and Safety and Adverse Effect Outcomes

Body weight (kg) change at discharge averaged −5% in the semaglutide group (mean [SD], −5.05% [3.56] vs 0.18% [2.5] in the placebo group; time-by-treatment interaction: β, −0.07; 95% CI, −0.08 to −0.05; *P* < .001) (Figure 3). No significant medication effects for hemoglobin A_1c_, blood pressure, or depression scores were observed. Participants treated with semaglutide reported expected adverse effects, which were largely mild in severity (eTable 1 in Supplement 2). No serious adverse events, adverse interactions with alcohol, or treatment-related discontinuations were recorded.

## Discussion

Preclinical studies have demonstrated GLP-1RA–induced suppression of voluntary alcohol intake and attenuation of behavioral and neurochemical measures of alcohol reward.^23,24,25,26^ Although most of these studies tested older GLP-1RAs (including exenatide and liraglutide), recent studies show that semaglutide reduces self-administration in mice, rats, and nonhuman primates.^30,31,32,33^ Separately, anecdotal and media reports of incidental reductions in alcohol use, first noted with older GLP-1RAs,^44^ have increased markedly alongside escalations in semaglutide prescriptions.^21,22^ Off-label prescribing of GLP-1RAs for AUD is already reported, necessitating data from controlled trials.^21,34^

This prospective clinical trial examined changes in laboratory and naturalistic alcohol consumption following weekly semaglutide treatment. Residualized change models of laboratory self-administration indicated that semaglutide reduced g-ETOH and peak BrAC among participants who engaged in a self-administration task at posttreatment, with evidence of medium to large effect sizes. Additionally, an exploratory analysis found that semaglutide reduced mean BrAC across the posttreatment self-administration session, with descriptive data suggesting the possibility of earlier de-escalation in consumption among participants treated with semaglutide, perhaps consistent with a satiety effect (Figure 2). For weekly drinking outcomes, medication effects on number of drinks per calendar day were nonsignificant; however, semaglutide significantly reduced alcohol craving and drinks per drinking day, also interacting with treatment week to predict reductions in heavy drinking days. Consequently, the proportion of participants with zero heavy drinking days increased significantly in the semaglutide group across the 2 dose phases. Semaglutide did not alter the proportion of abstinent vs drinking days. Though limited by a small subsample, an analysis of participants reporting current cigarette use revealed a significant medication-by-time interaction on changes in cigarettes per day.

Recent reports linking semaglutide or related drugs (including tirzepatide, a GLP-1/GIP dual receptor agonist) to alcohol-related outcomes have relied on observational or anecdotal data.^22,45,46^ The only prior randomized trial in an AUD sample^47^ found that exenatide—the first approved GLP-1RA—did not reduce overall consumption, but exploratory analyses indicated potential efficacy within a high-BMI (≥30) subgroup.^47^ Exploratory subgroup comparisons here (eFigure 3 in Supplement 2) did not indicate a similar pattern, suggesting the need to study efficacy across BMI levels. Although differences in sample characteristics or study design could contribute to differential evidence for efficacy across these 2 trials, the superior efficacy of semaglutide vs exenatide is likely the simplest interpretation.

Preliminary estimates of medication effect sizes yielded notable findings. Effect sizes approximated the small range (Cohen *d* = 0.20) during weeks 1 to 4 (0.25 mg/week), increasing during weeks 4 to 8 (0.5 mg/week). Effect sizes for heavy drinking days and drinks per drinking day reached the large range (*d* > 0.80) at the 0.5 mg/week dose, with objective laboratory data also suggesting large effect sizes on self-administration. By comparison, approved (and effective) AUD medications, including naltrexone, generally show small effect sizes in both clinical trials^15,48,49^ and laboratory studies of self-administration and other outcomes.^35,50^ While preliminary, the current effect size estimates are promising, especially considering those observed for FDA-approved AUD treatments.^51,52^ These findings are especially notable in that this study used the 2 lowest clinical doses of semaglutide, whereas doses for weight reduction reach 2.4 mg/week. Considering greater effects of semaglutide on other medical outcomes (eg, weight loss) with increasing dose and treatment duration, higher doses would presumably yield greater effects on alcohol reduction. However, safety profiles at higher doses in this population require careful evaluation. The extent of weight loss in the semaglutide group (−5% on average), though similar to that observed at these doses in populations with overweight or obesity, has additional safety risks for people with normal or low weight, necessitating evaluation of which doses (and which GLP-1RAs) optimally balance safety and efficacy in substance use disorder samples.

The focus on non–treatment-seeking participants has important considerations, one being that semaglutide-related reductions in drinking quantity occurred absent volitional attempts to reduce drinking. In contrast to treatment-seeking participants, this sample is arguably representative of the majority of those with AUD exposed to GLP-1RAs in general medical settings. Considering both the rapid adoption of GLP-1RAs and the demonstrated health benefits of net reductions in alcohol use,^53^ GLP-1RA–related reductions in consumption—when considered at large scale—could lead to improved health outcomes that are not currently appreciated. Recent pharmacoepidemiology results^54^ appear consistent with this possibility. Moreover, given low utilization of FDA-approved AUD therapies,^11^ the number of those with AUD receiving GLP-1RA medications already greatly exceeds the number who receive FDA-approved AUD medications. In this context, the broad uptake of GLP-1RAs presents an ideal scenario for medication repurposing, with potential to help reduce the wide treatment gap associated with AUD.

Evidence that semaglutide showed minimal effects on proportion of drinking vs abstinence days—with the largest effect sizes observed for drinking quantity and heavy drinking—also has possible clinical implications. The potential for selective effects on quantitative reductions in drinking could render GLP-1RAs well suited to nonabstinence goals,^14,15,16,53^ which are a preferred treatment goal for many with AUD. Notably, laboratory self-administration results also suggested no medication effects on delay time or abstinence (eResults in Supplement 2). Studies with treatment-seeking samples are needed to determine whether GLP-1RAs can facilitate abstinence or prevent relapse.

Though limited by a small subsample, a notable secondary finding was evidence for medication-related reductions in cigarettes per day among those reporting cigarette use. Based on preclinical evidence that GLP-1RAs reduce voluntary nicotine self-administration,^55^ recent studies have evaluated GLP-1RAs for smoking cessation and prevention of postcessation weight gain.^56,57^ Should GLP-1RAs prove efficacious for both alcohol reduction and smoking cessation, potential health implications could be substantial. Alcohol and cigarette use (along with obesity) are leading preventable causes of mortality and preventable cancer deaths,^58,59^ making individuals who smoke and drink heavily—including those with overweight or obesity—a priority population.^60,61,62^ Despite efforts to advance pharmacotherapies for concurrent AUD and tobacco use disorder,^63,64,65,66^ no medication is approved for both conditions.^62^

### Limitations

Among several study limitations are the modest sample size and short-term treatment duration, both reflecting the phase 2 stage of this trial. The use of low-dose semaglutide to maximize safety and feasibility likely limited detection of significant effects. However, the effect sizes observed at low doses are encouraging and provide information for estimating sample sizes for future trials. Another limitation is the moderate level of AUD severity of this sample, with consumption levels below those of most treatment-seeking samples. While this sample might reasonably approximate populations encountered in general medical settings, studies with heavier drinkers are necessary. Larger trials that address these questions while prioritizing FDA-accepted efficacy end points^40^ will ultimately inform the potential of GLP-1RAs as an emergent class of AUD therapies.^21,34^

## Conclusions

Since the FDA approval of the first AUD medication (disulfiram) in 1951, only 2 medications (naltrexone and acamprosate) have received subsequent FDA approval for AUD.^12,52^ The rate of 1 new approval every 20 to 25 years is inadequate and is in stark contrast with the pace of FDA approvals for diabetes medications, which now outnumber AUD medication approvals 20-fold. Should additional phase 2 and phase 3 clinical trials support repurposing 1 or more GLP-1RAs for AUD, these treatments could have broad clinical infiltration, with potential to bypass many traditional impediments to the uptake of AUD medications, including low public and provider awareness and stigma toward AUD treatments. Importantly, the increasing clinical uptake of GLP-1RAs would presumably reduce prescribing barriers, including in primary care, where AUD treatments have proven difficult to bring to scale. These possibilities call for larger clinical trials to evaluate efficacy of GLP-1RAs and other incretin therapies for AUD. Given numerous GLP-1RAs at various stages of development, future studies need not be limited to a single medication.

## Notes

Supplement 1. Trial protocol

Supplement 2. eMethods. Supplementary protocol and analysis plan information eResults. Supplementary results information eTable 1. Adverse events by medication group and treatment dose/month eTable 2. Full results for all models examining laboratory self-administration eTable 3. Full results for linear mixed models examining changes in weekly outcomes eFigure 1. Duration of delay in laboratory consumption by medication group eFigure 2. Mean cigarettes per day by week in semaglutide and placebo groups (n = 13) eFigure 3. Descriptive comparison of drinking outcomes by baseline body mass index (BMI)

Supplement 3. Data sharing statement

## References

[1] Shield K, Manthey J, Rylett M, . National, regional, and global burdens of disease from 2000 to 2016 attributable to alcohol use: a comparative risk assessment study. Lancet Public Health. 2020;5(1):e51-e61. doi:10.1016/S2468-2667(19)30231-2 31910980

[2] Global Status Report on Alcohol and Health and Treatment of Substance Use Disorders. World Health Organization; 2024.

[3] Esser MB, Sherk A, Liu Y, Naimi TS. Deaths from excessive alcohol use—United States, 2016-2021. MMWR Morb Mortal Wkly Rep. 2024;73(8):154-161. doi:10.15585/mmwr.mm7308a1 38421934 PMC10907037

[4] Manthey J, Shield KD, Rylett M, Hasan OSM, Probst C, Rehm J. Global alcohol exposure between 1990 and 2017 and forecasts until 2030: a modelling study. Lancet. 2019;393(10190):2493-2502. doi:10.1016/S0140-6736(18)32744-2 31076174

[5] Deutsch-Link S, Jiang Y, Peery AF, Barritt AS, Bataller R, Moon AM. Alcohol-associated liver disease mortality increased from 2017 to 2020 and accelerated during the COVID-19 pandemic. Clin Gastroenterol Hepatol. 2022;20(9):2142-2144.e2. doi:10.1016/j.cgh.2022.03.017 35314353 PMC8933289

[6] White AM, Castle IP, Powell PA, Hingson RW, Koob GF. Alcohol-related deaths during the COVID-19 pandemic. JAMA. 2022;327(17):1704-1706. doi:10.1001/jama.2022.4308 35302593 PMC8933830

[7] Julien J, Ayer T, Bethea ED, Tapper EB, Chhatwal J. Projected prevalence and mortality associated with alcohol-related liver disease in the USA, 2019-40: a modelling study. Lancet Public Health. 2020;5(6):e316-e323. doi:10.1016/S2468-2667(20)30062-1 32504584

[8] Julien J, Ayer T, Tapper EB, Barbosa C, Dowd WN, Chhatwal J. Effect of increased alcohol consumption during COVID-19 pandemic on alcohol-associated liver disease: a modeling study. Hepatology. 2022;75(6):1480-1490. doi:10.1002/hep.32272 34878683 PMC9015640

[9] Grant BF, Goldstein RB, Saha TD, . Epidemiology of *DSM-5* alcohol use disorder: results from the National Epidemiologic Survey on Alcohol and Related Conditions III. JAMA Psychiatry. 2015;72(8):757-766. doi:10.1001/jamapsychiatry.2015.0584 26039070 PMC5240584

[10] Substance Abuse and Mental Health Services Administration. National survey on drug use and health. Table 5.9A—alcohol use disorder in past year: among people aged 12 or older; by age group and demographic characteristics, numbers in thousands, 2021 and 2022. https://www.samhsa.gov/data/sites/default/files/reports/rpt42728/NSDUHDetailedTabs2022/NSDUHDetailedTabs2022/NSDUHDetTabsSect5pe2022.htm#tab5.9a

[11] Han B, Jones CM, Einstein EB, Powell PA, Compton WM. Use of medications for alcohol use disorder in the US: results from the 2019 National Survey on Drug Use and Health. JAMA Psychiatry. 2021;78(8):922-924. doi:10.1001/jamapsychiatry.2021.1271 34132744 PMC8209593

[12] Koob GF. Alcohol use disorder treatment: problems and solutions. Annu Rev Pharmacol Toxicol. 2024;64:255-275. doi:10.1146/annurev-pharmtox-031323-115847 38261428

[13] Witkiewitz K, Litten RZ, Leggio L. Advances in the science and treatment of alcohol use disorder. Sci Adv. 2019;5(9):eaax4043. doi:10.1126/sciadv.aax4043 31579824 PMC6760932

[14] Hasin DS, Wall M, Witkiewitz K, ; Alcohol Clinical Trials Initiative (ACTIVE) Workgroup. Change in non-abstinent WHO drinking risk levels and alcohol dependence: a 3 year follow-up study in the US general population. Lancet Psychiatry. 2017;4(6):469-476. doi:10.1016/S2215-0366(17)30130-X 28456501 PMC5536861

[15] Witkiewitz K, Hallgren KA, Kranzler HR, . Clinical validation of reduced alcohol consumption after treatment for alcohol dependence using the World Health Organization risk drinking levels. Alcohol Clin Exp Res. 2017;41(1):179-186. doi:10.1111/acer.13272 28019652 PMC5205540

[16] Aldridge AP, Zarkin GA, Dowd WN, . The relationship between reductions in WHO risk drinking levels during treatment and subsequent healthcare costs for the ACTIVE Workgroup. J Addict Med. 2022;16(4):425-432. doi:10.1097/ADM.0000000000000925 34864785 PMC9163210

[17] Drucker DJ. Efficacy and safety of GLP-1 medicines for type 2 diabetes and obesity. Diabetes Care. 2024;47(11):1873-1888. doi:10.2337/dci24-0003 38843460

[18] Drucker DJ. The GLP-1 journey: from discovery science to therapeutic impact. J Clin Invest. 2024;134(2):e175634. doi:10.1172/JCI175634 38226625 PMC10786682

[19] Drucker DJ. The benefits of GLP-1 drugs beyond obesity. Science. 2024;385(6706):258-260. doi:10.1126/science.adn4128 39024455

[20] Chao AM, Tronieri JS, Amaro A, Wadden TA. Semaglutide for the treatment of obesity. Trends Cardiovasc Med. 2023;33(3):159-166. doi:10.1016/j.tcm.2021.12.008 34942372 PMC9209591

[21] Leggio L, Hendershot CS, Farokhnia M, . GLP-1 receptor agonists are promising but unproven treatments for alcohol and substance use disorders. Nat Med. 2023;29(12):2993-2995. doi:10.1038/s41591-023-02634-8 38001271 PMC12320759

[22] Bremmer MP, Hendershot CS. Social media as pharmacovigilance: the potential for patient reports to inform clinical research on glucagon-like peptide 1 (glp-1) receptor agonists for substance use disorders. J Stud Alcohol Drugs. 2024;85(1):5-11. doi:10.15288/jsad.23-00318 37917019 PMC10846600

[23] Brunchmann A, Thomsen M, Fink-Jensen A. The effect of glucagon-like peptide-1 (GLP-1) receptor agonists on substance use disorder (SUD)-related behavioural effects of drugs and alcohol: a systematic review. Physiol Behav. 2019;206:232-242. doi:10.1016/j.physbeh.2019.03.029 30946836 PMC6520118

[24] Jerlhag E. Alcohol-mediated behaviours and the gut-brain axis; with focus on glucagon-like peptide-1. Brain Res. 2020;1727:146562. doi:10.1016/j.brainres.2019.146562 31759971

[25] Jerlhag E. The therapeutic potential of glucagon-like peptide-1 for persons with addictions based on findings from preclinical and clinical studies. Front Pharmacol. 2023;14:1063033. doi:10.3389/fphar.2023.1063033 37063267 PMC10097922

[26] Klausen MK, Thomsen M, Wortwein G, Fink-Jensen A. The role of glucagon-like peptide 1 (GLP-1) in addictive disorders. Br J Pharmacol. 2022;179(4):625-641. doi:10.1111/bph.15677 34532853 PMC8820218

[27] Fink-Jensen A, Vilsbøll T. Glucagon-like peptide-1 (GLP-1) analogues: a potential new treatment for alcohol use disorder? Nord J Psychiatry. 2016;70(8):561-562. doi:10.1080/08039488.2016.1176252 27151395

[28] Egecioglu E, Steensland P, Fredriksson I, Feltmann K, Engel JA, Jerlhag E. The glucagon-like peptide 1 analogue exendin-4 attenuates alcohol mediated behaviors in rodents. Psychoneuroendocrinology. 2013;38(8):1259-1270. doi:10.1016/j.psyneuen.2012.11.009 23219472

[29] Vallöf D, Kalafateli AL, Jerlhag E. Brain region specific glucagon-like peptide-1 receptors regulate alcohol-induced behaviors in rodents. Psychoneuroendocrinology. 2019;103:284-295. doi:10.1016/j.psyneuen.2019.02.006 30771711

[30] Aranäs C, Edvardsson CE, Shevchouk OT, . Semaglutide reduces alcohol intake and relapse-like drinking in male and female rats. EBioMedicine. 2023;93:104642. doi:10.1016/j.ebiom.2023.104642 37295046 PMC10363436

[31] Chuong V, Farokhnia M, Khom S, . The glucagon-like peptide-1 (GLP-1) analogue semaglutide reduces alcohol drinking and modulates central GABA neurotransmission. JCI Insight. 2023;8(12):e170671. doi:10.1172/jci.insight.170671 37192005 PMC10371247

[32] Fink-Jensen A, Wörtwein G, Klausen MK, . Effect of the glucagon-like peptide-1 (GLP-1) receptor agonist semaglutide on alcohol consumption in alcohol-preferring male vervet monkeys. Psychopharmacology (Berl). Published online June 17, 2024. doi:10.1007/s00213-024-06637-2 38884652 PMC11742737

[33] Marty VN, Farokhnia M, Munier JJ, Mulpuri Y, Leggio L, Spigelman I. Long-acting glucagon-like peptide-1 receptor agonists suppress voluntary alcohol intake in male Wistar rats. Front Neurosci. 2020;14:599646. doi:10.3389/fnins.2020.599646 33424537 PMC7785877

[34] Volkow ND, Xu R. GLP-1R agonist medications for addiction treatment. Addiction. July 24, 2024. doi:10.1111/add.16626 39049203 PMC12771407

[35] Hendershot CS, Wardell JD, Samokhvalov AV, Rehm J. Effects of naltrexone on alcohol self-administration and craving: meta-analysis of human laboratory studies. Addict Biol. 2017;22(6):1515-1527. doi:10.1111/adb.12425 27411969 PMC6139429

[36] McKee SA, Harrison EL, O’Malley SS, . Varenicline reduces alcohol self-administration in heavy-drinking smokers. Biol Psychiatry. 2009;66(2):185-190. doi:10.1016/j.biopsych.2009.01.029 19249750 PMC2863311

[37] McKee SA, Verplaetse TL. A novel human laboratory alcohol self-administration paradigm for medication screening: modeling the ability to resist drinking and heavy drinking. Drug Alcohol Depend Rep. Published online September 4, 2022. doi:10.1016/j.dadr.2022.100085 36120181 PMC9481061

[38] Sobell LC, Sobell MB. Timeline Follow-Back: A Technique for Assessing Self-Reported Alcohol Consumption. Measuring Alcohol Consumption: Psychosocial and Biochemical Methods. Springer; 1992:41-72.

[39] Flannery BA, Volpicelli JR, Pettinati HM. Psychometric properties of the Penn Alcohol Craving Scale. Alcohol Clin Exp Res. 1999;23(8):1289-1295. doi:10.1111/j.1530-0277.1999.tb04349.x 10470970

[40] Belnap MA, McManus KR, Grodin EN, Ray LA. Endpoints for pharmacotherapy trials for alcohol use disorder. Pharmaceut Med. 2024;38(4):291-302. doi:10.1007/s40290-024-00526-x 38967906 PMC11272707

[41] Johnson BA, Ait-Daoud N, Roache JD. The COMBINE SAFTEE: a structured instrument for collecting adverse events adapted for clinical studies in the alcoholism field. J Stud Alcohol Suppl. 2005;(15):157-167. doi:10.15288/jsas.2005.s15.157 16223067

[42] Lewinsohn PM, Seeley JR, Roberts RE, Allen NB. Center for Epidemiologic Studies Depression Scale (CES-D) as a screening instrument for depression among community-residing older adults. Psychol Aging. 1997;12(2):277-287. doi:10.1037/0882-7974.12.2.277 9189988

[43] Kuznetsova A, Brockhoff PB, Christensen RHB. lmerTest package: tests in linear mixed effects models. J Stat Softw. 2017;82(13). doi:10.18637/jss.v082.i13

[44] Kalra S, Kalra B, Sharma A. Change in alcohol consumption following liraglutide initiation: a real life experience. American Diabetes Association Annual Meeting 2011 Poster 1029. Alexandria, Virginia; 2011.

[45] Quddos F, Hubshman Z, Tegge A, . Semaglutide and tirzepatide reduce alcohol consumption in individuals with obesity. Sci Rep. 2023;13(1):20998. doi:10.1038/s41598-023-48267-2 38017205 PMC10684505

[46] Richards JR, Dorand MF, Royal K, Mnajjed L, Paszkowiak M, Simmons WK. Significant decrease in alcohol use disorder symptoms secondary to semaglutide therapy for weight loss: a case series. J Clin Psychiatry. 2023;85(1):23m15068. doi:10.4088/JCP.23m15068 38019594

[47] Klausen MK, Jensen ME, Møller M, . Exenatide once weekly for alcohol use disorder investigated in a randomized, placebo-controlled clinical trial. JCI Insight. 2022;7(19):e159863. doi:10.1172/jci.insight.159863 36066977 PMC9675448

[48] Maisel NC, Blodgett JC, Wilbourne PL, Humphreys K, Finney JW. Meta-analysis of naltrexone and acamprosate for treating alcohol use disorders: when are these medications most helpful? Addiction. 2013;108(2):275-293. doi:10.1111/j.1360-0443.2012.04054.x 23075288 PMC3970823

[49] Rösner S, Hackl-Herrwerth A, Leucht S, Vecchi S, Srisurapanont M, Soyka M. Opioid antagonists for alcohol dependence. Cochrane Database Syst Rev. 2010;(12):CD001867. doi:10.1002/14651858.CD001867.pub3 21154349 PMC13590218

[50] Ray LA, Green R, Roche DJO, Magill M, Bujarski S. Naltrexone effects on subjective responses to alcohol in the human laboratory: a systematic review and meta-analysis. Addict Biol. 2019;24(6):1138-1152. doi:10.1111/adb.12747 31148304 PMC6819195

[51] Kranzler HR, Hartwell EE. Medications for treating alcohol use disorder: a narrative review. Alcohol Clin Exp Res (Hoboken). 2023;47(7):1224-1237. doi:10.1111/acer.15118 37526592

[52] Leggio L, Falk DE, Ryan ML, Fertig J, Litten RZ. Medication development for alcohol use disorder: a focus on clinical studies. Handb Exp Pharmacol. 2020;258:443-462. doi:10.1007/164_2019_295 31628604

[53] Gapstur SM, Bouvard V, Nethan ST, . The IARC perspective on alcohol reduction or cessation and cancer risk. N Engl J Med. 2023;389(26):2486-2494. doi:10.1056/NEJMsr2306723 38157507

[54] Wang L, Volkow ND, Berger NA, Davis PB, Kaelber DC, Xu R. Associations of semaglutide with incidence and recurrence of alcohol use disorder in real-world population. Nat Commun. 2024;15(1):4548. doi:10.1038/s41467-024-48780-6 38806481 PMC11133479

[55] Herman RJ, Schmidt HD. Targeting GLP-1 receptors to reduce nicotine use disorder: preclinical and clinical evidence. Physiol Behav. 2024;281:114565. doi:10.1016/j.physbeh.2024.114565 38663460 PMC11128349

[56] Herman RJ, Hayes MR, Audrain-McGovern J, Ashare RL, Schmidt HD. Liraglutide attenuates nicotine self-administration as well as nicotine seeking and hyperphagia during withdrawal in male and female rats. Psychopharmacology (Berl). 2023;240(6):1373-1386. doi:10.1007/s00213-023-06376-w 37129617 PMC11088902

[57] Yammine L, Green CE, Kosten TR, . Exenatide adjunct to nicotine patch facilitates smoking cessation and may reduce post-cessation weight gain: a pilot randomized controlled trial. Nicotine Tob Res. 2021;23(10):1682-1690. doi:10.1093/ntr/ntab066 33831213 PMC8517504

[58] Islami F, Marlow EC, Thomson B, . Proportion and number of cancer cases and deaths attributable to potentially modifiable risk factors in the United States, 2019. CA Cancer J Clin. 2024;74(5):405-432. doi:10.3322/caac.21858 38990124

[59] Viner B, Barberio AM, Haig TR, Friedenreich CM, Brenner DR. The individual and combined effects of alcohol consumption and cigarette smoking on site-specific cancer risk in a prospective cohort of 26,607 adults: results from Alberta’s Tomorrow Project. Cancer Causes Control. 2019;30(12):1313-1326. doi:10.1007/s10552-019-01226-7 31535325

[60] Falk DE, Yi HY, Hiller-Sturmhöfel S. An epidemiologic analysis of co-occurring alcohol and tobacco use and disorders: findings from the National Epidemiologic Survey on Alcohol and Related Conditions. Alcohol Res Health. 2006;29(3):162-171. 17373404 PMC6527037

[61] Dawson DA. Drinking as a risk factor for sustained smoking. Drug Alcohol Depend. 2000;59(3):235-249. doi:10.1016/S0376-8716(99)00130-1 10812284

[62] McKee SA, Weinberger AH. How can we use our knowledge of alcohol-tobacco interactions to reduce alcohol use? Annu Rev Clin Psychol. 2013;9(1):649-674. doi:10.1146/annurev-clinpsy-050212-185549 23157448 PMC3651830

[63] Roche DJ, Ray LA, Yardley MM, King AC. Current insights into the mechanisms and development of treatments for heavy drinking cigarette smokers. Curr Addict Rep. 2016;3(1):125-137. doi:10.1007/s40429-016-0081-3 27162709 PMC4859339

[64] King A, Cao D, Vanier C, Wilcox T. Naltrexone decreases heavy drinking rates in smoking cessation treatment: an exploratory study. Alcohol Clin Exp Res. 2009;33(6):1044-1050. doi:10.1111/j.1530-0277.2009.00925.x 19302083 PMC4625790

[65] Kahler CW, Spillane NS, Metrik J. Alcohol use and initial smoking lapses among heavy drinkers in smoking cessation treatment. Nicotine Tob Res. 2010;12(7):781-785. doi:10.1093/ntr/ntq083 20507898 PMC2893295

[66] Ray LA, Courtney KE, Ghahremani DG, Miotto K, Brody A, London ED. Varenicline, low dose naltrexone, and their combination for heavy-drinking smokers: human laboratory findings. Psychopharmacology (Berl). 2014;231(19):3843-3853. doi:10.1007/s00213-014-3519-0 24733235 PMC4161630

