# Supplementary material for: Once-Weekly Semaglutide in Adults With Alcohol Use Disorder: A Randomized Clinical Trial
Source: JAMA Psychiatry. 2025 Feb 12;82(4):395–405. doi: 10.1001/jamapsychiatry.2024.4789 (PMC11822619; doi:10.1001/jamapsychiatry.2024.4789)
Supplement: Supplement 1. — Trial protocol [file jamapsychiatry-e244789-s001.pdf]

NIH-FDA Phase 2 and 3 IND/IDE Clinical Trial Protocol Template

Human Laboratory Screening of Semaglutide for Alcohol Use Disorder

Protocol Number: 21-1689

National Clinical Trial (NCT) Identified Number: Pending

Investigators:

Dr. Christian Hendershot (Principal Investigator)
Dr. Robyn Jordan (Co-Investigator)
Dr. Amanda Tow (Co-Investigator)
Dr. Sarah Dermody (Co-Investigator, Biostatistician)
Dr. Eric Claus (Co-Investigator)
Dr. Klara Klein (Co-Investigator)
Dr. Paul Fletcher (Other Significant Contributor)

Funded by: NIAAA

Version Number: v.1.7

9 Feb 2023

Summary of Changes from Previous Version:

Affected Section(s)	Summary of Revisions Made	Rationale
1.1 (population), 5.1, 5.2	Revised inclusion/exclusion criteria to remove cigarette smoking and use of nicotine products as eligibility factors; expanded BMI range for eligible participants (from 25-24 to 23+), and removed eligibility criterion related to TSH values (thyroid disease will still be exclusionary).	Adjustments are intended to make eligibility less restrictive, and to remove criteria that are not central to the primary objectives.

1 TABLE OF CONTENTS

STATEMENT OF COMPLIANCE ... 1
1 PROTOCOL SUMMARY ... 1
1.1 Synopsis ... 1
1.2 Schema ... 3
1.3 Schedule of Activities (SoA) ... 4
2 INTRODUCTION ... 5
2.1 Study Rationale ... 5
2.2 Background ... 6
2.3 Risk/Benefit Assessment ... 8
2.3.1 Known Potential Risks ... 8
2.3.2 Known Potential Benefits ... 10
2.3.3 Assessment of Potential Risks and Benefits ... 10
3 OBJECTIVES AND ENDPOINTS ... 11
4 STUDY DESIGN ... 13
4.1 Overall Design ... 13
4.2 Scientific Rationale for Study Design ... 22
4.3 Justification for Dose ... 22
4.4 End of Study Definition ... 23
5 STUDY POPULATION ... 23
5.1 Inclusion Criteria ... 23
5.2 Exclusion Criteria ... 23
5.3 Lifestyle Considerations ... 24
5.4 Screen Failures ... 25
5.5 Strategies for Recruitment and Retention ... 25
6 STUDY INTERVENTION ... 26
6.1 Study Intervention(s) Administration ... 26
6.1.1 Study Intervention Description ... 26
6.1.2 Dosing and Administration ... 27
6.2 Preparation/Handling/Storage/Accountability ... 28
6.2.1 Acquisition and accountability ... 28
6.2.2 Formulation, Appearance, Packaging, and Labeling ... 28

6.2.3 Product Storage and Stability ... 28
6.2.4 Preparation ... 29

6.3 Measures to Minimize Bias: Randomization and Blinding ... 29

6.4 Study Intervention Compliance ... 29

6.5 Concomitant Therapy ... 29
6.5.1 Rescue Medicine ... 30

7 STUDY INTERVENTION DISCONTINUATION AND PARTICIPANT DISCONTINUATION/WITHDRAWAL ... 30

7.1 Discontinuation of Study Intervention ... 30

7.2 Participant Discontinuation/Withdrawal from the Study ... 30

7.3 Lost to Follow-Up ... 31

8 STUDY ASSESSMENTS AND PROCEDURES ... 32

8.1 Efficacy Assessments ... 32

8.2 Safety and Other Assessments ... 32

8.3 Adverse Events and Serious Adverse Events ... 33
8.3.1 Definition of Adverse Events (AE) ... 33
8.3.2 Definition of Serious Adverse Events (SAE) ... 33
8.3.3 Classification of an Adverse Event ... 33
8.3.4 Time Period and Frequency for Event Assessment and Follow-Up ... 34
8.3.5 Adverse Event Reporting ... 35
8.3.6 Serious Adverse Event Reporting ... 35
8.3.7 Reporting Events to Participants ... 36
8.3.8 Events of Special Interest ... 36
8.3.9 Reporting of Pregnancy ... 36

8.4 Unanticipated Problems ... 36
8.4.1 Definition of Unanticipated Problems (UP) ... 36
8.4.2 Unanticipated Problem Reporting ... 37
8.4.3 Reporting Unanticipated Problems to Participants ... 37

9 STATISTICAL CONSIDERATIONS ... 37

9.1 Statistical Hypotheses ... 37

9.2 Sample Size Determination ... 38

9.3 Populations for Analyses ... 38

9.4 Statistical Analyses ... 38
9.4.1 General Approach ... 38
9.4.2 Analysis of the Primary Efficacy Endpoint(s) ... 39
9.4.3 Analysis of the Secondary Endpoint(s) ... 39
9.4.4 Safety Analyses ... 40

9.4.5 Baseline Descriptive Statistics ... 40
9.4.6 Planned Interim Analyses ... 40
9.4.7 Sub-Group Analyses ... 40
9.4.8 Tabulation of Individual participant Data ... 40
9.4.9 Exploratory Analyses ... 40

10 SUPPORTING DOCUMENTATION AND OPERATIONAL CONSIDERATIONS. 40

10.1 Regulatory, Ethical, and Study Oversight Considerations ... 40

10.1.1 Informed Consent Process ... 40
10.1.2 Study Discontinuation and Closure ... 41
10.1.3 Confidentiality and Privacy ... 42
10.1.4 Future Use of Stored Specimens and Data ... 42
10.1.5 Key Roles and Study Governance ... 43
10.1.6 Clinical Monitoring ... 43
10.1.7 Quality Assurance and Quality Control ... 44
10.1.8 Data Handling and Record Keeping ... 44
10.1.9 Protocol Deviations ... 44
10.1.10 Publication and Data Sharing Policy ... 44
10.1.11 Conflict of Interest Policy ... 45

10.2 Additional Considerations ... 45

10.3 Abbreviations ... 45

10.4 Protocol Amendment History ... 48

11 REFERENCES ... 50

STATEMENT OF COMPLIANCE

(1) The trial will be carried out in accordance with International Conference on Harmonization Good Clinical Practice (ICH GCP) and the following:

- United States (US) Code of Federal Regulations (CFR) applicable to clinical studies (45 CFR Part 46, 21 CFR Part 50, 21 CFR Part 56, 21 CFR Part 312, and/or 21 CFR Part 812)

National Institutes of Health (NIH)-funded investigators and clinical trial site staff who are responsible for the conduct, management, or oversight of NIH-funded clinical trials have completed Human Subjects Protection and ICH GCP Training.

The protocol, informed consent form(s), recruitment materials, and all participant materials will be submitted to the Institutional Review Board (IRB) for review and approval. Approval of both the protocol and the consent form must be obtained before any participant is enrolled. Any amendment to the protocol will require review and approval by the IRB before the changes are implemented to the study. In addition, all changes to the consent form will be IRB-approved; a determination will be made regarding whether a new consent needs to be obtained from participants who provided consent, using a previously approved consent form.

1 PROTOCOL SUMMARY

1.1 SYNOPSIS

Title: Human Laboratory Screening of Semaglutide for Alcohol Use Disorder

Study Description: This is an early-Phase II human laboratory trial using a randomized, placebo-controlled, dose-ranging design to investigate the effects of semaglutide (Ozempic®), a GLP-1 receptor agonist, on laboratory alcohol responses and consumption, naturalistic alcohol and cigarette consumption, and weight loss in smokers with alcohol use disorder (AUD). Participants will attend weekly visits while semaglutide dosage is increased to 1.0mg over a period of approximately 10 weeks. Participants will attend weekly visits for medication or placebo administration. At scheduled intervals, participants will complete 4 laboratory sessions involving alcohol self-administration and alcohol challenge to characterize medication effects on alcohol-related outcomes.

Objectives: Primary Objective: To examine effects of semaglutide vs. placebo on laboratory alcohol intake during a validated alcohol self-administration medication screening procedure.

Secondary Objectives: To examine medication vs. placebo group differences in 1) laboratory responses to alcohol, and 2) changes in naturalistic alcohol use and cigarette consumption over the course of the treatment period.

	Tertiary Objectives: To generate preliminary data on weight loss, HbA1c reductions and alcohol elimination among participants with AUD during treatment with a GLP-1 receptor agonist.
**Endpoints:**	Primary Endpoint: Change in amount of alcohol consumed during a validated laboratory alcohol self-administration protocol (primary endpoint). The specific effect of interest is the change in laboratory consumption from baseline to the maintenance dose (.5mg) as a function of medication condition (semaglutide vs. placebo) (i.e., treatment x time interaction).<br><br>Secondary Endpoints: Secondary endpoints will include laboratory measures of alcohol and cigarette demand (behavioral economic indices of drug motivation) and subjective responses to alcohol during an alcohol challenge procedure, and changes in naturalistic alcohol use and cigarette smoking over the treatment period. Within-person changes in these outcomes before vs. after treatment (baseline vs. 0.5mg) will be compared as a function of medication condition.
**Study Population:**	Participants who meet criteria for alcohol use disorder/AUD (N=48) will be enrolled. Participants will be recruited from the Triangle area (community and hospital settings). Participants must not be seeking current treatment for alcohol.
**Phase:**	Phase II
**Description of Sites/Facilities Enrolling Participants:**	This is a single-site study (UNC-Chapel Hill). The project will be based at the Bowles Center for Alcohol studies and the Department of Psychiatry, with clinical operations taking place at Eastowne Clinical Research Unit and CTRC.
**Description of Study Intervention:**	Semaglutide (Ozempic®) will be administered according to the FDA-approved schedule, which begins with a starting dose of 0.25mg once per week for four weeks. At week five, dosage is increased to 0.5mg/week for a subsequent four weeks (maintenance dose). Following four weeks at the maintenance dose, dosage will be increased to 1.0mg provided the patient has tolerated the medication. Semaglutide is administered subcutaneously via the Ozempic® pen, a cartridge-based device that calibrates medication delivery. The placebo condition will consist of weekly sham doses, which will involve a brief needle insertion (without an injection).
**Study Duration:**	Up to 13 months.
**Participant Duration:**	Up to 40-50 hours total (including in-person visits and remote questionnaires) over a period of approximately 12 weeks.

1.2 SCHEMA

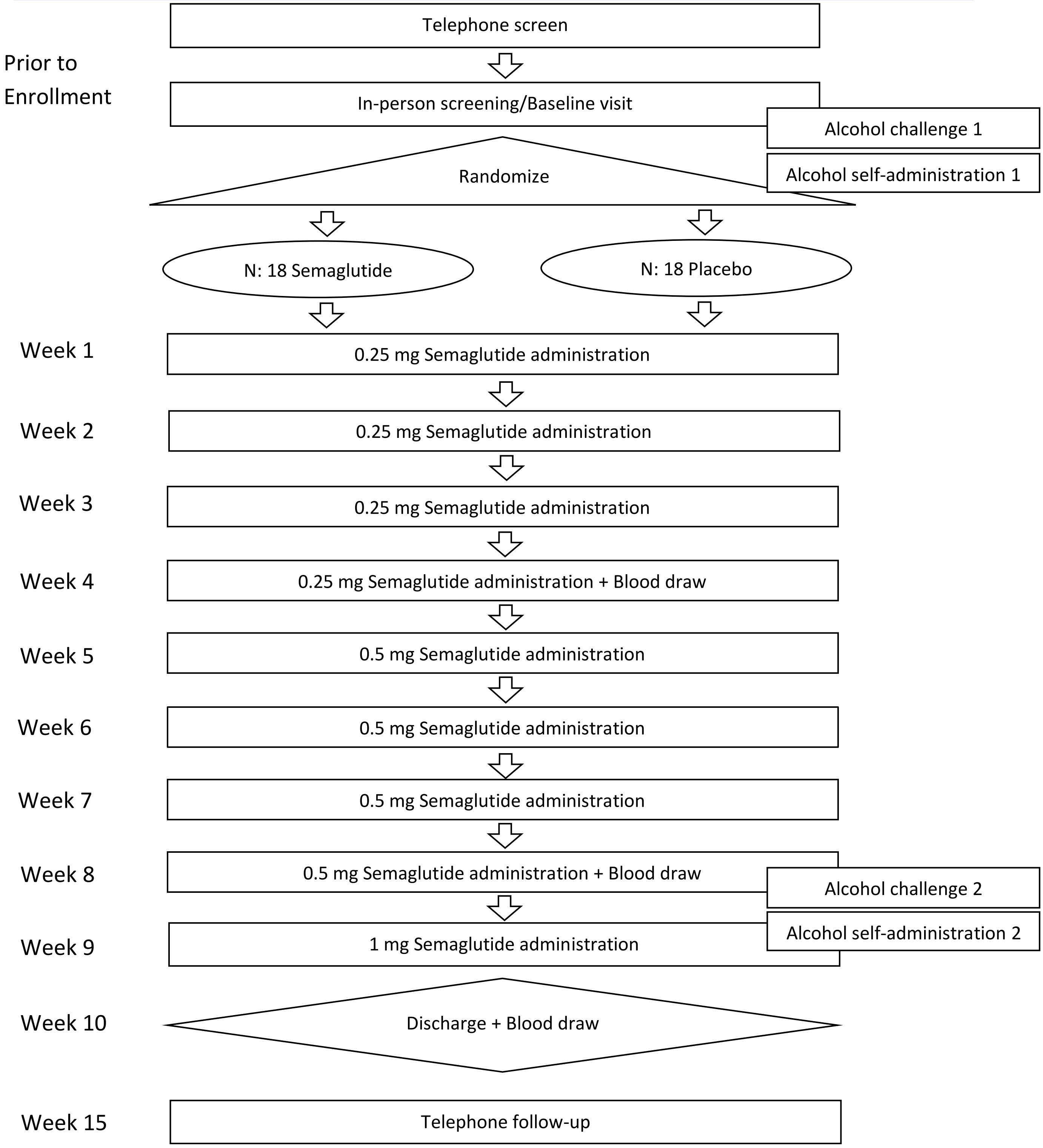
Telephone screen
Prior to Enrollment
In-person screening/Baseline visit
Alcohol challenge 1
Alcohol self-administration 1
Randomize
N: 18 Semaglutide
N: 18 Placebo
Week 1
0.25 mg Semaglutide administration
Week 2
0.25 mg Semaglutide administration
Week 3
0.25 mg Semaglutide administration
Week 4
0.25 mg Semaglutide administration + Blood draw
Week 5
0.5 mg Semaglutide administration
Week 6
0.5 mg Semaglutide administration
Week 7
0.5 mg Semaglutide administration
Week 8
0.5 mg Semaglutide administration + Blood draw
Alcohol challenge 2
Alcohol self-administration 2
Week 9
1 mg Semaglutide administration
Week 10
Discharge + Blood draw
Week 15
Telephone follow-up

1.3 SCHEDULE OF ACTIVITIES (SOA)

Procedures and Questionnaires	Telephone Screening	Screening/Baseline	Study Visit 1 – 3 Target Day 7, 14, 21	Study Visit 4 Target Day 28	Study Visit 5 – 7 Target Day 35, 42, 49	Study Visit 8 Target Day 56	Study Visit 9 Target Day 63	Discharge Target Day 70	Telephone follow-up Target Day 77, 105	Alcohol Challenge Target Day -10, 58	Alcohol Self-Admin. Target Day -12, 60
Telephone screening	X										
Administer 0.25 mg Semaglutide or placebo			X	X							
Administer 0.5 mg Semaglutide or placebo					X	X					
Administer 1.0 mg Semaglutide or placebo							X				
Breath Alcohol Concentration (BrAC) Test		X	X	X	X	X	X	X		X	X
Urine Drug Screen		X								X	X
Expired carbon monoxide (CO)		X	X	X	X	X	X	X		X	X
Locator		X									
Timeline Follow-Back (TLFB)		X	X	X	X	X	X	X			
BAC		X	X	X	X	X	X	X		X	X
Blood Draw		X		X		X		X			
Blood glucose – finger prick		X	X		X		X			X	X
Pregnancy (females only)		X	X	X	X	X	X	X		X	X
BMI		X	X	X	X	X	X	X		X	X
Height		X									
Vitals (HR, BP)		X	X	X	X	X	X	X		X	X
Weight		X	X	X	X	X	X	X		X	X
Temperature		X									
Demographics Form		X									
Structured Clinical Interview for DSM-5		X									
MINI Neuropsychiatric Interview		X									
Medical and Substance Use History Interviews		X									
Prediction of Alcohol Withdrawal Severity Scale (PAWS)		X									
Short Alcohol Withdrawal Scale (SAWS)		X								X	X
Saliva Collection for DNA and nicotine metabolite		X									
Case Report Forms (CRFs)	X	X	X	X	X	X	X	X	X	X	X
Alcohol Use Disorder Identification Test (AUDIT)		X									
Alcohol, Cigarette, and Food Purchase Test		X								X	X
Self-Report of the Effects of Alcohol Questionnaire (SRE)		X									
Fagerstrom Test for Nicotine Dependence (FTND)		X									
Nicotine Dependence Syndrome Scale (NDSS)		X									
Penn Alcohol Craving Scale (PACS)		X	X	X	X	X	X	X		X	X
Tiffany Questionnaire of Smoking Urges (TQSU)		X	X	X	X	X	X	X		X	X
Wisconsin Smoking Withdrawal Scale (WSWS)		X	X	X	X	X	X	X		X	X
Alcohol and Smoking Contemplation		X	X	X	X	X	X	X			
Three Factor Eating Questionnaire (TFEQ)		X	X	X	X	X	X	X		X	X
Monetary Choice Questionnaire (MCQ)		X	X	X	X	X	X	X		X	X
Cigarette Abstinence Self-Efficacy (CASE)		X	X	X	X	X	X	X		X	X
Alcohol Abstinence Self-Efficacy (AASE)		X	X	X	X	X	X	X		X	X
Inventory of Drinking Situations (IDS)		X									
Impaired Control Scale (ICS)		X									
Barratt Impulsivity Scale (BIS-11)		X									
UPPS Impulsive Behavior Scale (UPPS)		X									
Systematic Assessment for Treatment Emergent Effects (SAFTEE)		X	X	X	X	X	X	X	X	X	X

Reward Based Eating Drive Scale (R-BEDS)		X	X	X	X	X	X	X		X	X
Simplified Nutritional Appetite Questionnaire (SNAQ)		X	X	X	X	X	X	X		X	X
Control of Eating Questionnaire (CEQ)		X	X	X	X	X	X	X		X	X
Wisconsin Inventory of Smoking Dependence Motives (WISDM)		X									
Sweet Taste Preference Test		X						X			
Center for Epidemiological Studies Depression Scale (CES-D)		X									
Reward Probability Index – Environmental Reward Observation Scale (RPI-EROS)		X									
Toronto Alexithymia Scale (TAS-20)		X									
Profile of Mood States – Short Form (POMS-SF)										X	X
Biphasic Alcohol Effects Scale (BAES)										X	X
Alcohol Urge Questionnaire (AUQ)										X	X
Drug Effects Questionnaire (DEQ)										X	X
Obsessive-Compulsive Drinking Scale		X									
Graded Pain Scale		X									

2 INTRODUCTION

2.1 STUDY RATIONALE

Pharmacotherapy development is a critical objective for reducing health and societal burdens associated with alcohol use disorder (AUD) [1-3]. Despite advances in this area, medication development remains a slow- moving endeavor with a protracted path from drug discovery to market [2-4]. Given the high degree of heterogeneity in AUD clinical presentation and treatment response, it is recognized that no single medication will be effective for all patients [5]. Therefore, the National Institute on Alcohol Abuse and Alcoholism (NIAAA) medication development strategy specifies the long-term objective of establishing a repertoire of therapies to facilitate targeted interventions for specific AUD subgroups [3, 5, 6]. Strategies for expediting the study of novel treatments are central to these aims [3, 5, 6].

Epidemiological and clinical evidence shows that cigarette smokers comprise a sizable AUD subgroup with disproportionately high health burdens [7, 8] Alcohol and cigarette use are two of the three leading causes of preventable mortality worldwide, jointly accounting for an estimated seven million deaths per year [9]. Population-based research shows that cigarette consumption increases as a function of alcohol use severity [7]. In one large epidemiological study, nearly half (44.6% of men, 47.3% of women) of those with alcohol dependence also met criteria for nicotine dependence [7]. Co-use of alcohol and cigarettes predicts synergistic increases in risk for numerous disease conditions [8, 10, 11], including esophageal and laryngeal cancers [12, 13]. Given these health burdens, increasing effort has been allocated to developing targeted interventions for smokers with AUD [8, 11, 14, 15].

In the absence of FDA-approved medications for concurrent alcohol and nicotine addiction, identifying candidate drugs for this indication is a priority [11, 15, 16]. Several recent studies have examined whether FDA- approved AUD and smoking cessation drugs, either alone or in combination, can facilitate joint reductions in alcohol and cigarette use [11]. For instance, naltrexone reduces alcohol consumption in heavy-drinking smokers [17, 18], and in some cases reduces cigarette consumption [17, 19]. Recent findings further suggest that naltrexone's efficacy for reducing drinking is stronger in AUD participants

who smoke relative to non-smokers [20, 21]. However, a Cochrane review [22] and a large, prospective AUD trial [19, 23] concluded that naltrexone does not appear to reduce cigarette smoking [11, 23].

Varenicline has also been tested as a candidate therapy for smokers with heavy drinking or AUD. Informed by early findings that varenicline reduced alcohol self-administration in a human laboratory trial [16], large-scale clinical trials supported efficacy of varenicline for reducing alcohol consumption in heavy-drinking smokers [24, 25] and those with AUD [26]. Recent laboratory-based studies have also examined the combination of varenicline and naltrexone as a novel treatment [27, 28]. Because those with concurrent alcohol and nicotine addiction reflect a high-priority subgroup, further efforts are needed to screen candidate treatments for this population [8, 11, 28] . As reflected in these recent trials, and as noted explicitly under the NIAAA medication development framework [1-3], drug repurposing strategies can significantly expedite new treatment approaches by capitalizing on therapies that have already passed the extensive regulatory steps required for FDA approval.

Compounds with preclinical evidence for reducing both alcohol and nicotine intake are particularly good candidates for investigating new treatments for heavy-drinking smokers. Accumulating evidence suggests that *Glucagon-like peptide 1* (GLP-1) receptor agonists may have promise in this respect. GLP-1 is an incretin hormone produced and secreted both peripherally (in the intestines) and centrally (in the hindbrain) [29]. GLP-1 is stimulated following food consumption and stimulates satiety and decreases in blood glucose. As a peptide with both hormonal and neurotransmitter functions, GLP-1 plays a role in the "gut-brain axis," which has recently been invoked as a target for addiction therapies [30]. GLP-1 agonist medications were designed to mimic the function of the endogenous GLP-1 peptide, with the benefit of a much longer half-life than endogenous GLP-1. Based on their glucose-lowering and appetite-reducing properties, GLP-1 agonists were developed as anti-diabetic therapies.

Several GLP-1 receptor agonists are now in widespread use as treatments for type II diabetes (T2D), including exenatide, liraglutide, lixisenatide, albiglutide, dulaglutide and semaglutide. These medications have also proven efficacious for promoting weight loss, reducing glycated hemoglobin (HbA1c), and reducing risk for some cardiovascular events [29]. Although the primary indication of GLP-1 agonists is T2D, these therapies are increasingly being studied for other indications in non-diabetic patients, including obesity/weight management.

Of note, GLP-1 agonists require subcutaneous administration, typically by way of a pre-filled medication cartridge with a micro-needle attachment, which allows for easy self-administration in the home without assistance from medical staff. Semaglutide is a recently approved (2017) FDA-approved GLP-1 agonist. A new oral formulation of semaglutide was approved by the FDA in 2019, making semaglutide the first drug in its class to be available in both subcutaneous and oral formulations.

2.2 BACKGROUND

Accumulating evidence from preclinical studies suggests that GLP-1 agonists reduce alcohol and other drug intake and/or reinstatement in animal models. In a recent review of 17 preclinical studies [31], all studies reported significant effects of GLP-1 agonists on indices of drug motivation and intake, with the evidence being most robust for alcohol [30, 31]. Notably, the effects of GLP-1 agonists on drug intake appear to extend to several addictive drugs, including nicotine [32]. These findings have led to recent studies aiming to investigate the efficacy of GLP-1 agonists as candidate therapies for reducing intake or reinstatement of alcohol, nicotine, and other drugs (e.g., cocaine). Although several human trials are

underway in various regions, no RCTs have been published to date, and human data on the efficacy of GLP-1 agonists for reducing drug intake in substance-using populations is needed.

Although the precise mechanisms mediating the effects of GLP-1 agonists on alcohol-related behaviors remain to be clarified, it has been suggested that cholinergic-dopaminergic pathways are involved in altering the reward value of alcohol following exposure to GLP-1 agonists [30]. Additionally, GLP-1 agonists appear to exert their effects in part by way of interactions with the serotonin (5-HT) system, including interactions with 5-HT2C receptors. Specifically, GLP-1 agonists appear to induce serotonin release, and serotonin signaling appears critical to the efficacy of the medications [33, 34]. Some evidence also suggests that 5-HT2C receptors appear necessary for the efficacy and satiety-inducing effects of GLP-1 agonists [35, 36].

Of the approved FDA-approved GLP-1 agonists, most studies have focused on exenatide, an older GLP-1 agonist first approved in 2005. As noted, semaglutide is the newest GLP-1 agonist and the first available in both subcutaneous and oral formulations. Some evidence suggests superiority of semaglutide relative to other GLP-1 agonists with respect to efficacy for glycemic control [37]. Although clinical trials of GLP-1 receptor agonists in participants with substance use disorder are now underway, no studies (to our knowledge) are currently underway to examine semaglutide as a treatment for alcohol use disorder, or for the joint indication of alcohol use disorder and tobacco use disorder. Demonstrating tolerability and initial efficacy of semaglutide for these populations could ultimately allow for greater clinical flexibility if GLP-1 agonists prove efficacious for these populations, given that semaglutide is available in both subcutaneous and oral formulations.

Human laboratory methods for Phase II medication screening

As emphasized in the NIAAA medication development strategy [1, 4, 38], human laboratory methods play a pivotal role in drug development by providing a bridge from preclinical studies and full-scale Phase II/III trials [39-43]. Importantly, laboratory studies can expedite drug development by providing an efficient, cost-effective, and rigorous option for “early-Phase II” medication screening, thereby informing priorities for larger and more costly randomized trials. For example, we recently published meta-analytic evidence that human laboratory studies of naltrexone, when examined in aggregate, yield conclusions highly comparable to the aggregate data from large-scale randomized trials, reinforcing the notion that laboratory trials offer a valid and efficient approach for estimating the potential efficacy of candidate therapeutics [40]. Other advantages of laboratory trials include the ability to study putative treatment mechanisms under controlled conditions, and the ability to quickly generate initial data on drug tolerability in the clinical population of interest [1, 44].

Phase II pharmacotherapy trials for alcohol use disorder often rely on alcohol administration procedures to study medication effects on acute responses to alcohol in human laboratory settings [40]. Controlled alcohol exposure offers a means of testing medication effects on subjective response to alcohol (e.g., stimulation, sedation) and alcohol-induced craving. These subjective responses to alcohol are targets of existing pharmacotherapies, such as naltrexone [40]. Alcohol self-administration has also been used as a laboratory model of alcohol motivation, and has successfully been used to screen candidate AUD therapies [40]. Some have argued that alcohol self-administration should serve as the central endpoint in human laboratory studies, and validated procedures exist for modeling alcohol self-administration safely in the laboratory [16] [40]. More recently, behavioral economic measures have also been utilized as a means of studying drug motivation in laboratory contexts, and appear sensitive for detecting experimental manipulations on alcohol-related motivation. For example, *alcohol demand* indexes the

relative reinforcing value of alcohol at a particular point in time [45]. Measures of drug demand, such as the alcohol purchase task (APT) and cigarette purchase task (CPT), are considered objective and sensitive experimental assays of drug motivation [45]. The APT predicts frequency of heavy drinking days after treatment [45], and is sensitive to temporal changes in alcohol's relative reinforcing efficacy during drug administration [46]. Notably, initial evidence suggests that these measures are sensitive to the effects of pharmacological treatments, including naltrexone and varenicline [47, 48]. Measures of drug demand offer an alternative to studying self-administration in the laboratory, complementing measures of subjective drug responses.

One limitation of many laboratory-based pharmacotherapy trials is the tendency to focus on heavy drinkers without AUD, rather than participants who report symptoms of the disorder [49, 50]. A recent systematic review found evidence that recruiting non-clinical samples for laboratory trials can potentially limit the ability of laboratory findings to generalize to larger clinical trials, perhaps undermining translational research efforts [51]. While ethical considerations normally preclude laboratory alcohol administration methods with treatment-seeking AUD participants [50], studies of *non-treatment-seeking* AUD participants are ethically justified, and can provide important data on medication effects and safety in the target clinical population [52]. Additionally, focusing on participants with <8 of out 11 AUD symptoms endorsed) can ensure maximal safety when studying this population by eliminating the likelihood of clinically significant withdrawal or alcohol-related medical complications. Our team has ample prior experience with alcohol administration procedures in both clinical and non-clinical populations; we will utilize these methods to study the potential efficacy of semaglutide in participants with AUD.

2.3 RISK/BENEFIT ASSESSMENT

2.3.1 KNOWN POTENTIAL RISKS

Semaglutide (Ozempic®) received FDA approval in 2017 for use as an adjunct to diet and exercise to achieve glycemic control in adults with type 2 diabetes mellitus (T2D). Semaglutide received FDA approval based on the results of 25 completed trials with the goal of providing results that would be applicable to a broad type II diabetes (T2D) population, including the elderly, patients with renal impairment, and patients at high cardiovascular risk. These trials involved > 9,300 participants, of whom >5,700 were treated with semaglutide. In these trials, treatment with semaglutide alone was efficacious for reduction in hemoglobin A1C (HbA1c) below 7% (e.g., 73% of participants had HbA1c <7% at week 30, compared to 28% of placebo-treated patients). Significant body weight reductions were also reported, with the weight loss achieved at end-of-treatment ranging from -1.43 to -4.28 kg (corresponding to 2.3–4.9%). Weight reductions were evident after 4 weeks of treatment, and persisted up to 104 weeks [75]. The peak plasma concentration of semaglutide is reached 1-3 days post-dose. Steady-state exposure is achieved following 4-5 weeks of once-weekly administration, and elimination half-life is approximately 1 week.

<u>Risks related to medication.</u> The most commonly reported side effects/adverse events (AEs) are gastrointestinal, with nausea being the most frequently reported symptom. The majority of reports of nausea (0.5 mg dose vs placebo; 15.3 vs. 6.1%), vomiting (5.0 vs 2.3%), and/or diarrhea (8.5 vs 1.9%) occur shortly following a dose escalation. Abdominal pain (7.3 vs 4.6%) and constipation (5.0 vs 1.5%) were also reported [76]. In studies ranging from 30-56 weeks in duration, the discontinuation rate due

to these gastrointestinal AEs was ranged from 5-13%, with this number increasing during longer treatment durations [77].

Rarer AEs (occurring at a frequency of <5%) included symptomatic hypoglycemia (≤70 mg/dL glucose threshold), which occurred in 1.6% of semaglutide-treated participants versus 0% placebo participants; dyspepsia (3.5 vs 1.9%); eructation (2.7 vs 0%); and gastroesophageal reflux disease (1.9 vs 0%). In placebo-controlled trials, injection site reactions (e.g., discomfort, erythema) were reported in only 0.2% of semaglutide-treated patients, suggesting good tolerability of subcutaneous administration. Patients exposed to semaglutide had a mean increase from baseline in amylase of 13% and lipase of 22%. Cholelithiasis was reported in 1.5% of treated patients. The 0.5 mg dose resulted in a mean increase in heart rate of 2 to 3 beats per minute, while there was a mean decrease in heart rate of 0.3 beats per minute in placebo-treated patients. Hypersensitivity reactions (e.g., anaphylaxis, angioedema) have been reported with GLP-1 receptor agonists. [76]. Other adverse reactions with a frequency of >0.4% associated with semaglutide include fatigue, dysgeusia and dizziness [76].

Rodent studies have shown an increase in the incidence of thyroid C-cell tumors after lifetime exposure of semaglutide at clinically relevant plasma levels. However, several studies in T2D patients taking GLP-1R agonists have shown no increase in neoplasm risk, even after several years of use [78]. Nevertheless, a personal or family history of medullary thyroid cancer or multiple endocrine neoplasia 2A or 2B is considered a potential risk factor for taking the medication, and will be an exclusionary criterion.

In a 2-year trial, acute pancreatitis was observed in 8 semaglutide-treated patients (0.27 cases per 100 patient years) and 10 placebo-treated patients (0.33 cases per 100 patient years). One case of chronic pancreatitis was confirmed in a semaglutide-treated patient. History of pancreatitis will be an exclusion factor, and study participants receiving semaglutide will be observed for signs and symptoms of pancreatitis (via blood monitoring of serum amylase and lipase). If pancreatitis is suspected, semaglutide will be discontinued, and appropriate management recommended.

Other rare events include postmarketing reports of acute kidney injury and worsening of chronic renal failure, which may sometimes require hemodialysis, in patients receiving long-term treatment with GLP-1 receptor agonists. Some of these events have been reported in patients without known underlying renal disease. Most of these events occurred in patients who had experienced nausea, vomiting, diarrhea, or dehydration. Therefore, we will monitor renal function by including a measure of serum creatinine at baseline and at subsequent visits involving blood samples.

Although most safety data on semaglutide comes from trials with T2D participants, there are now a number of randomized trials underway examining GLP-1 receptor agonists in participants with substance use disorder, including trials on tobacco, cocaine, alcohol, cannabis, and opioids. A full list of registered randomized trials can be found by searching for “GLP-1” at ClinicalTrials.gov. This study will be the first to our knowledge to focus specifically on heavy-drinking smokers. The use of a short-term medication screening protocol (lasting roughly 10 weeks) will provide an opportunity to demonstrate tolerability and generate data on side effect profiles in this population while minimizing any risks associated with longer-term medication exposure.

Risks Related to Questionnaire and Interview Assessments. There may be potential risks associated with assessment procedures. Participants may find the battery of psychiatric and psychological assessments tedious or intrusive. Participants may experience discomfort resulting from questions about personal histories or substance use patterns.

Participation in Alcohol Administration Sessions. Other potential risks include typical risks related to participation in alcohol administration studies. For example, it is possible that participants may experience dizziness/nausea from drinking during these visits, or symptoms the following day (e.g., headache). Our group has considerable experience with alcohol administration and self- administration procedures [80-84], and the safety precautions required in these studies. Across these studies, the incidence of adverse events such as vomiting has been exceptionally low. Our procedures will follow the NIAAA recommended guidelines for administering alcohol to human subjects (https://www.niaaa.nih.gov/Resources/ResearchResources/job22.htm). Briefly, the NIAAA guidelines include recommendations for administering alcohol to participants who meet criteria for AUD. In particular, we have taken measures to protect against ethical concerns by *excluding treatment-seekers* (or those engaged in an active quit attempt), as per the NIAAA recommended guidelines. Additionally, we will screen out participants with severe AUD (defined as 6 or more of 11 symptoms). This approach allows us to generate data relevant to a clinical population while minimizing risks.

Risks Related to DNA Collection. The purpose of collecting saliva for DNA in this study is to allow secondary analyses to examine whether medication effects might be different according to certain genetic factors. These analyses may also focus on genetic factors potentially associated with drug use or related behaviors, such as nicotine and alcohol metabolism, dopaminergic function, or impulsivity. Potential risks associated with DNA collection involve potential identification, and there may be risks with genetic (DNA) tests that are as yet unknown. Genes may be shown at some point in the future to be related to mental illnesses or tendency to addiction. Samples will be labeled with participant ID only, and analyzed at an outside laboratory. Samples will be de-identified and links with participant identifiers will be broken upon completion of the analyses. We will not conduct any whole genome sequencing of the DNA samples. Additionally, the consent form will state that participants have the option to opt out of DNA collection, and to request that their samples are destroyed at any time during the study. Participants will not receive any feedback from any of the genetic tests from study staff.

2.3.2 KNOWN POTENTIAL BENEFITS

The participants in this study are not expected to benefit directly from their participation. However, the knowledge gained from this study could lead to a better understanding of a medication with potential to treat alcohol and nicotine addiction, and potentially other substance use disorders.

2.3.3 ASSESSMENT OF POTENTIAL RISKS AND BENEFITS

Given the vast health implications of substance use disorder and the currently limited array of effective medications, it is imperative to pursue novel pharmacotherapeutic options. A key strategy in this regard is to “repurpose” medications that are already approved (and have proven safe) in the context of other health conditions. Given the sizable amount of research on GLP-1 receptor agonists in other clinical populations, coupled with preclinical evidence that these medications reduce motivation for alcohol, nicotine, and other drugs, evaluating this medication class in the proposed population is warranted. Given no available pharmacotherapies for the indication of concurrent alcohol and nicotine addiction, any evidence for potential efficacy in heavy-drinking smokers could have substantial implications. From this standpoint, the risk/benefit profile for testing semaglutide in a population of non-diabetic participants is favorable.

Although we anticipate the risk of adverse events to be low based on the safety profile of the medication, gradual dose escalation, and relatively short duration of treatment, several steps will be taken to track and report medication-related side effects. As described further in later sections of this application, participants will be monitored regularly by virtue of attending weekly lab visits for medication delivery, at which side effects will be assessed and clinical lab results monitored. Participants will be given a contact number for study staff in case of emergent side effects (and instructed to seek medical attention in the event of any serious side effects), and participants will be contacted by phone shortly after dose escalations to monitor symptoms. We expect some participants to experience nausea shortly following dose escalations, but that these symptoms will usually be transient.

Given the focus on a non-obese population, we will be particularly vigilant in monitoring changes in body weight and blood glucose, and will exclude participants at the outset with body weight or blood glucose levels below normal range, based on physician judgement. We will carefully monitor participants with glucose tests and weight measurements at regular study visits (see schedule of activities table), and will instruct participants to be vigilant for potential symptoms of hypoglycemia. Periodic blood tests to measure fasting glucose, fasting insulin, serum lipase and amylase, serum creatinine, and serum calcitonin will occur at baseline and regularly thereafter (i.e., at visits prior to dose escalations). All adverse events will be documented and reported in accordance with relevant policies, and the regulations of NIH. We have enlisted the TraCS DSMB to assist with safety monitoring of the study. The board will convene at the beginning of the study and at scheduled points to review study progress and safety data, and to review any SAEs and considerations related to stopping rules (described further below).

Considering the use of an FDA approved medication that has been deemed safe in the context of chronic administration for glycemic control, the risks related to medication use in this short-term screening trial are acceptable in comparison to the potential benefits of the proposed research (i.e., demonstrating potential efficacy of a new medication for substance use disorder).

Risks Related to Alcohol Administration Sessions. Alcohol administration procedures are commonly used to study risk factors for alcohol use disorder (AUD), and have proven capable of identifying successful pharmacotherapies [40]. As per the NIAAA guidelines for administration of alcohol in human studies, such studies are typically restricted to non-treatment-seeking participants in order to minimize ethical concerns, as will be the approach in this study. Further details on safety procedures are provided later in this document.

3 OBJECTIVES AND ENDPOINTS

OBJECTIVES	ENDPOINTS	JUSTIFICATION FOR ENDPOINTS
Primary		
To examine effects of semaglutide vs. placebo on alcohol self-administration under laboratory conditions.	Amount of alcohol consumed and breath alcohol concentration (co-primary endpoints) during a validated laboratory alcohol self-administration protocol. In this procedure, participants are provided the option of choosing between drinks and	Primary: This outcome is a laboratory assay of alcohol intake, and has been used in prior human laboratory studies to screen candidate medications, allowing comparisons with effect sizes for other medications. This session will be conducted during the standard

OBJECTIVES	ENDPOINTS	JUSTIFICATION FOR ENDPOINTS
	monetary reward. The comparison of interest is the difference between medication and placebo groups in the change in laboratory consumption between baseline and the maintenance dose of 0.5mg.	maintenance dose (0.5mg) to mimic the common clinical scenario.<br><br>Secondary: Subjective alcohol responses and alcohol/cigarette demand are validated measures of drug reward/motivation, and are known to be sensitive to medication effects. Given substantial individual differences in alcohol pharmacokinetics, a within-subjects analysis is preferable for these endpoints (such that each participant will serve as their own control). Laboratory sessions will take place at two time points: baseline and 0.5mg maintenance dose.
Secondary		
To examine group differences in 1) alcohol/cigarette demand and subjective responses to alcohol during alcohol administration, and 2) naturalistic drinking and smoking behaviors over the course of the 10-week treatment period.	Laboratory measures of subjective responses to alcohol, alcohol demand, and cigarette demand during controlled laboratory alcohol administration (lab sessions will take place at baseline and again after achieving the maintenance dose).<br><br>Number of standard drinks consumed and number of cigarettes consumed per day during the treatment phase (Weeks 0-10), as measured by a daily diary report.<br><br>Similar to the primary outcome, secondary outcome analyses will examine medication vs. placebo group differences in within-subjects changes in these outcomes over time (baseline to 0.5mg).	Laboratory measures of alcohol response and alcohol/cigarette demand are clinically valid indicators of drug motivation, and are demonstrated to be sensitive to medication effects. Investigating these outcomes under controlled conditions will allow us to test for a medication “signal” on measures of alcohol and nicotine motivation.<br><br>Daily measures of alcohol consumption (standard drinks per day) and cigarettes per day are selected to afford greater power for modeling change in consumption over the treatment period. Note that these outcomes are considered secondary because this Phase II trial involves non-treatment-seeking participants who are not attempting to reduce alcohol or use or cigarette smoking.
Tertiary/Exploratory		
Body weight, HbA1c, alcohol elimination.	Changes in body weight, HbA1c, and alcohol elimination will be examined as exploratory endpoints based on potential clinical relevance.	GLP-1 receptor agonists lower HbA1c and body weight in indicated populations (e.g., Type II diabetes, obesity). Given high co-occurrence of obesity and alcohol use disorder, reporting whether medication is

OBJECTIVES	ENDPOINTS	JUSTIFICATION FOR ENDPOINTS
		associated with these reductions in the present sample is clinically relevant. Data from the alcohol administration sessions will be used to estimate whether medication predicts changes in alcohol elimination during treatment, and whether changes in body weight account for any such differences. These outcomes are exploratory.

4 STUDY DESIGN

4.1 OVERALL DESIGN

Overview of Study Design.

The aim of this protocol is to translate preclinical findings on GLP-1 agonists by conducting an early-Phase II investigation of semaglutide in non-treatment-seeking participants with AUD, with a focus on human laboratory outcomes. Given preclinical evidence for the potential efficacy of GLP-1 agonists for reducing both alcohol and nicotine intake, and the need for treatments targeting both alcohol and nicotine addiction, this proposal focuses on cigarette smokers with AUD.

This is a single-site, Phase II, two-arm, double-blind (participants, outcome assessors), randomized, placebo-controlled, dose-ranging study. Following a baseline assessment eligible participants will be randomized to medication or placebo conditions by the IDS staff at a 1:1 ratio. The treatment period will be approximately 10 weeks, taking into account the dose escalation necessary for this medication. Those randomized to semaglutide will receive 0.25mg/week for 4 weeks (Weeks 1-4), and 0.5mg/week for 4 weeks (Weeks 5-8). Provided that participants have tolerated the medication , participants will receive a final dose of 1.0mg (Week 9), and will return for a discharge lab visit with repeat lab tests at Week 10, followed by a final monitoring assessment by phone scheduled approximately 6 weeks after the last medication dose (Week 15). Those in the placebo condition will receive sham placebo administration at each visit (described in further detail in later sections). Participants will attend weekly visits at Eastowne CRU or CTRC to receive medication or placebo doses.

In addition to the medication visits, participants will be asked to attend four laboratory visits: two involving alcohol self-administration (one prior to the first medication visit and one during the 0.5mg treatment phase) and two involving alcohol challenge (one prior to the first medication visit and one during the 0.5mg treatment phase). These sessions are designed to assess changes in alcohol responses over the course of the treatment period. Laboratory sessions are described in further detail below. Measurements of naturalistic alcohol and cigarette use will also be recorded throughout the treatment period. These assessments will be captured using a daily diary that will be provided to participants. Participants will receive an automated text message daily via Phone.com as a reminder to complete the diary. Additional self-report measures of substance use will be collected at study visits.

As summarized above, the primary objectives are to examine whether treatment with semaglutide alters alcohol self-administration (primary outcome) and laboratory measures of alcohol response and motivation (i.e., demand and responses to alcohol, secondary outcomes) during alcohol administration. We hypothesize that the semaglutide treatment (versus placebo) will be associated with reduced alcohol self-administration during a laboratory session conducted at the maintenance dose of 0.5mg. We further hypothesize that semaglutide will lead to reductions in alcohol demand, cigarette demand, and subjective responses to alcohol (e.g., reduced stimulation) during alcohol administration sessions.

The primary and secondary outcomes for this trial will be assessed during exposure to the standard maintenance dose (.5mg). While an extended treatment phase at the 1.0mg dose is beyond the scope of this small-scale trial, the inclusion of a brief (1-week) phase at 1.0mg is designed to provide initial data on tolerability and any changes on selected secondary outcomes (i.e., drinks per day and cigarettes per day) in this population. Capturing these data will help to support feasibility and pilot data for subsequent funding proposals with longer treatment intervals.

Secondary objectives include examining changes in naturalistic alcohol consumption and cigarette smoking during the treatment period. Although participants will not be attempting to reduce drinking or smoking in this trial, it is hypothesized that treatment with semaglutide will result in reductions in alcohol consumption (drinks per day) and cigarettes per day over the course of the treatment period. Tertiary/exploratory outcomes include examining changes in body weight, HbA1c, and alcohol elimination during the trial.

In total, participation in this study is estimated to take a maximum of up to 50 hours of combined time over a period of up to approximately 16 weeks beginning with initial screening. This includes a screening and baseline visit (up to 4 hours), up to 9 subsequent laboratory visits for medication administration and side effects monitoring (up to 1 hour each), a final discharge visit (Week 10), and final telephone check-in (Week 15). If necessary for safety reasons, (e.g., based on lab results), participants may be asked to attend (unplanned) visit(s) for follow-up labs at the discretion of the study physician. Participants will also complete four visits involving alcohol administration over the course of Weeks 0-10; these sessions may last up to 7-8 hours each. Participants also will receive calls from staff 2-3 days after each medication phase begins (placebo, 0.25mg, 0.5mg, 1.0mg) to check in on side effects (10 minutes per call), and will be asked to complete brief daily questionnaires via a daily diary with reminders sent via daily text message prompt (1-2 minutes per day).

A detailed summary of study procedures is provided below.

Detailed Study Procedures.

Following initial recruitment (described in section 5.5), participants will be asked to complete the following procedures:

a) <u>Telephone screen</u>

Initial screening will be conducted by telephone by trained research staff and will assess participant alcohol, cigarette, and drug use, medications, and basic medical inclusion/exclusion factors. Potential participants will be given a brief description of the study. The initial telephone screen is estimated to take roughly 15-30 minutes. Contact information will be collected from interested callers who pass the initial screen. Following the initial screen, participants will proceed to be booked for an informed consent and baseline interview session. The informed consent procedures

will be conducted remotely using a HIPAA-compliant virtual meeting platform (e.g., Webex, Zoom) and Docusign. Depending on participant and staff availability, the phone screening and informed consent/baseline interview can happen on the same day (after the screening call) or at a future date.

b) Screening and Baseline Data Collection Visits

At the informed consent session, a member of the research staff will review the study procedures and carry out informed consent procedures (including electronic signature via Docusign) prior to proceeding to the baseline interview. Following informed consent, the staff member will conduct a semi-structured interview to assess inclusion/exclusion criteria. A research assistant will use a structured interview to measure diagnostic and substance use screening criteria, including symptoms of alcohol use disorder; inclusion/exclusion factors related to medical history will also be assessed (see criteria listed above). The Structured Clinical Interview for DSM-V [53] will be used to assess AUD and substance use disorder symptoms. History of other psychiatric diagnoses and medication use will be assessed via self-report. The research assistant will also collect information on demographic factors, criteria related to current medical status and medications, current alcohol, tobacco and other substance use (see "Phone Screen" document), as well as substance use disorder symptoms (via select modules from the SCID interview) and symptoms other psychiatric symptoms (using the MINI screening module). Participants who appear eligible will be booked for an in-person visit, which will take place at the laboratory site (Medical Wings C), with some procedures (e.g., blood draw) booked at CTRC as necessary.

The in-person screening/baseline visit will include a brief review of informed consent to answer any additional questions, and a brief review of self-report and interview questions from the remote screening session. The Timeline Follow-Back (TLFB) [54] will be used to assess alcohol, cigarette and other drug use in the 90 days prior. The participant will also provide a breathalyzer screen. Participants who have a positive BAC (greater than 0.00) will be monitored until their BAC is reduced to a safe level (BAC<.03g%), in accordance with NIAAA guidelines (or to 0.00g% in the event that the participant drove to the session).

Participants who remain eligible after the interview will complete vitals and measurements (HR, BP, height, weight, temp), and a measure of expired alveolar carbon monoxide (CO). A baseline saliva sample for DNA analysis will also be collected. Additionally, participants will provide a urine sample and complete a blood draw (up to 70-80 ml) for a comprehensive metabolic panel (e.g., Na, K, Cl, CO2, BUN, Creatinine, Glucose, Calcium, Albumin, Total protein, Total bilirubin, AST, ALT, Alk Phos), baseline measure of HbA1c, baseline measure of nicotine metabolites (e.g., 3HC, COT), serum amylase and lipase, and complete blood count (e.g., Auto WBC, Corrected WBC, RBC, HGB, HCT, MCV, MCH, MCHC, RDW, MPV, Platelet). These procedures will be conducted by the research assistant, and the baseline blood draw will be taken by a team member with phlebotomy training. If there is no team member with phlebotomy training available that day, the blood draw may also be booked at CTRC. If needed, the blood draw and other baseline procedures may be booked as separate visits. On the day of the visit that includes the blood draw, participants will be instructed to arrive fasting for 12 hours (to allow for assessment of baseline HbA1c and lipids). If participants are not able to arrive fasting for the baseline session, the fasting lipids sample may be deferred or completed at the next possible visit. Blood samples will be sent to McClendon laboratory for testing. A portion of the blood sample (de-identified) will also be stored (Thurston Bowles building and Neurosciences Research

Building) for subsequent analysis (e.g., nicotine metabolites and blood neutrophils, which are affected by cigarette smoking). Neutrophil analyses will be conducted by Dr. Robert Tarran's lab. The saliva sample will also be stored at Thurston Bowles building. The urine sample will be used to test for the presence of several drugs (for example, heroin, cocaine, methamphetamines, opiates). (For females, the test will include a pregnancy screen.) If a participant tests positive for drugs other than cannabis (or if females screen positive for pregnancy), the participant will be excluded from the study.

Participants will complete baseline questionnaires, which will be collected on paper. A Demographic Screening Questionnaire will assess basic demographic factors (e.g., age, education, income). A Locator form will collect contact information for three individuals who can be contacted if a participant is unable to be reached by study staff for follow-up visits. Other baseline measures will include the Alcohol Use Disorders Identification Test (AUDIT) [55], Self-Report of the Effects of Alcohol Questionnaire (SRE) [56, 57], Fagerström Test of Nicotine Dependence (FTND) [58], and the Nicotine Dependence Syndrome Scale (NDSS) [59], Barratt Impulsivity Scale (BIS-11) [60], UPPS Impulsive Behavior Scale (UPPS) [61], Impaired Control Scale (ICS) [62], Center for Epidemiological Studies Depression Scale (CES-D) [63], Toronto Alexithymia Scale (TAS-20) [64], the Reward Probability Index (RPI) and the Environmental Reward Observation Scale (EROS) [65]. Baseline measures that will be repeated at subsequent visits will include the Timeline Followback (TLFB), the Penn Alcohol Craving Scale (PACS) and Tiffany Questionnaire of Smoking Urges (TQSU) [66], and the Wisconsin Smoking Withdrawal Scale (WSWS) [67]. To assess potential medication effects on changes in motivation and self-efficacy for resisting alcohol/cigarettes, we will use alcohol and smoking Contemplation Ladder items, Alcohol and Cigarette Abstinence Self-Efficacy (AASE/CASE) (these scales will be repeated at each visit) and the Inventory of Drinking Situations (IDS) scales [68-70]. Appetite and eating behavior will be assessed with the Simplified Nutritional Appetite Questionnaire (SNAQ) [71], Control of Eating Questionnaire (CEQ) [72], the Three Factor Eating Questionnaire (TFEQ) [73] and the Reward-Based Eating Drive Scale (R-BEDS) [74]. During the medication phases, side effects will be assessed with the SAFTEE questionnaire [75], a standard tool for alcohol clinical trials. Participants will also complete previously validated Purchase Tasks for alcohol, cigarettes, and food, and delay discounting will be assessed with the Monetary Choice Questionnaire (MCQ) [76-79]. These brief behavioral economic tasks assess perceived value of reinforcers by presenting hypothetical scenarios involving choices between the reinforcer (alcohol, cigarette, food) and monetary rewards; these measures will be completed at each visit. Finally a previously validated sweet taste preference test will be administered at baseline and again on the final visit, since this phenotype has been shown to be implicated in both food and alcohol reward, and may be modulated by a GLP-1 agonist. This test involves participants tasting solutions of sugar water prepared to reach .05, .1, .2, .42, and .83 molar sucrose, and then rating preference and intensity of the taste. The sweet taste test will be overseen by a research assistant using previously published protocols from prior projects at UNC [80, **Error! Hyperlink reference not valid.**].

The baseline questionnaires and interview assessments are projected to take approximately 3-4 hours combined. To accommodate potential scheduling/time constraints or delays at the baseline visit, some questionnaires that are not time-sensitive (for instance, personality questionnaires) may be deferred until a later lab visit as needed, at the discretion of the study team. All interview records and paper questionnaires will be stored in study binders in a secure location for subsequent entry into an electronic database for analysis. Patients will be given

breaks as needed. Following the blood draw, participants will be offered a snack and bottled water (given the instructions to arrive fasting). If results of the baseline screening/medical assessment indicate that the participant is tentatively eligible, they will be given a comprehensive orientation to the next phases of study, including the follow-up medication visits, the laboratory alcohol administration visits, and the daily surveys. Locator/collateral contact information will also be obtained. A Locator form will collect contact information for three individuals who can be contacted if a participant is unable to be reached by study staff for follow-up visits. Following the completed baseline assessments, the participant's screening summary will be reviewed for eligibility, including review of medical criteria and any medications that the participant is taking by the study physician. Lab results will be uploaded to the EMR and reviewed by the study physician prior to prescribing the medication. Potential interactions with semaglutide will be verified using an established online database such as Epocrates or Lexicomp. In the event of any uncertainties about medication interactions, participants will be asked to contact their primary care physician to confirm no concerns with taking semaglutide.

For eligible participants, the study physician will provide sign-off on all medical criteria (e.g., medical history, medications, lab results), and the PI will provide sign-off on other baseline criteria. Once participants have been confirmed as eligible, the physician will submit an order to start medication or placebo. Medication and placebo will be dispensed at CTRC (Week 1 visit) at a date coordinated with the participant. Participants will be advised to be vigilant for symptoms of hypoglycemia at the medical visit, and instructed to contact the study team to review any symptoms that emerge (regular glucose tests will also be obtained at follow-up visits, as noted in the schedule of assessments). If necessary, the medication review will take place via telephone or telemedicine, depending on medical team availability.

Participants' Week 1 visit will be scheduled with enough lead time to review lab results and exclude participants (if needed) prior to administering the study medication. Additionally, if clarification of medical criteria is needed at any point during or following the phone or in-person screening process, or at any subsequent stage of the study, the study physician may meet with the participant via a remote contact method (e.g., phone, Webex/Zoom, or telehealth) to clarify medical questions or discuss test results.

c) Daily Assessments.

Throughout the study, participants will be asked to complete a brief self-report survey each day that includes measures of 1) prior-day number drinks consumed; 2) number of cigarettes smoked; and 3) prior day peak craving/demand for alcohol and cigarettes. Participants will be provided with a paper booklet in which to enter responses and will be asked to bring the booklet to each visit. At each visit the research staff will collect the interim daily surveys for retention in the participant's study binder. Participants will be texted an automated text message each day during their participation in the study as a reminder to complete the daily survey. If preferred, participants can opt to receive an email link. (No daily survey data will be collected from participants via text or email.) At each study visit, a staff member will provide compensation for the prior week's surveys, and will review survey adherence and progress towards compensation for good adherence (described later), as a means of motivating adherence to self-report assessments. In the event a participant completes the daily assessments but forgets to bring the packet to a given visit, they will be asked to bring their packet to the following session. Participants will be asked to either a) complete the measures in person at the current lab visit, or b) to schedule a brief meeting with a research team member via Zoom or

Webex in order to complete the questions (depending on scheduling and availability). These approaches will be used in specific circumstances if needed (e.g., if the participants forgets to complete the daily assessments altogether, or will be unable to bring it to the next session).

d) Weekly Laboratory Visits.

Medication administration visits will occur at either the Eastowne Clinical Research Unit (CRU) or the CTRC. Following the baseline (Week 0) visit, participants will visit CRU or UNC Hospital/CTRC once weekly (Weeks 1-9), for subsequent medication or placebo doses, and for a final follow-up/discharge visit at Week 10. (In addition, the study physician may request an additional follow-up lab visit(s) at any point in the study to monitor side effects.) In addition, to accommodate events such as missed lab visits or scheduling difficulties, assessment procedures at a given weekly visit (e.g., questionnaires, lab tests) may be conducted within a 7-day window of the original target date, as necessary, to avoid unnecessary missing data. In addition to medication/placebo administration, follow-up visits will include 1) vitals, weight measurements, and a brief side-effects assessment, 2) a finger-stick glucose test (or fasting blood draw depending on the week, see study flow chart), 3) a brief calendar-based self-report assessment of interim alcohol and cigarette use (TLFB), and 4) a repeat CO measurement. These procedures are expected to take roughly 30-60 minutes in total, and will be conducted by a trained study team member and/or a member of the CRU/CTRC team (e.g., RN). All side effects reports, labs, and other medical test results (e.g., glucose tests) will be uploaded in Epic (e.g., lab tests) or sent electronically (e.g., self-reported medication changes) for review by the study physician. If side effects or lab results suggest that further assessment is indicated, a member of the medical team will advise the study team (and if necessary, contact the participant) to provide direction. At the Week 4, Week 8, and final discharge visits, participants will be asked to arrive fasting to allow for a blood draw for follow-up lab assays (identical to those described in the baseline procedures above). As with the baseline sample, a portion of each sample will be stored for follow-up analyses (e.g., nicotine metabolites).

e) Alcohol Sessions.

To evaluate effects of medication phase on laboratory measures of alcohol response and self-administration, participants will be asked to complete four laboratory visits over the course of the study. Two visits will occur between the Week 0 and 1 visits (baseline phase), and two will occur between the Week 8 and 9 visits (0.5 mg phase). Minor scheduling deviations will be accommodated as needed, provided that the participant has completed at least three weeks of the .5mg phase at the time of the second set of laboratory sessions.

During each of these sessions participants will be asked to report to the lab having received instructions to abstain from alcohol and recreational drugs for 24h, and not to consume food for 4 hours prior to arrival. Participants will also be advised not to drive to these study appointments. Participants will also be asked to schedule a smoking break just prior to arrival (to ensure that participants are not nicotine-deprived during testing) [16]. Upon arrival participants will provide breathalyzer and expired CO readings, as well as measures of body weight and vitals (HR, BP) and a finger-stick blood glucose test. Participants will also receive the Short Alcohol Withdrawal Scale (SAWS) to ensure that no significant withdrawal symptoms are present. Evidence of withdrawal symptoms will require a consultation with an addiction medicine physician (Dr. Tow or Dr. Jordan) prior to proceeding. (We except a low likelihood that this will happen because participants with

severe AUD or history of withdrawal symptoms will be excluded). Female participants will also be asked to repeat the urine pregnancy screen at each alcohol session.

In the interim, participants will complete self-report questionnaires (e.g., Timeline Follow-Back to assess recent substance use). Participants will be seated in a comfortable recliner chair for the alcohol administration procedures. Participants will first complete baseline physiological readings (systolic and diastolic blood pressure, heart rate), and baseline measurements of self-report questionnaires (see below). Next, participants will complete one of two procedures (alcohol challenge, alcohol self-administration), dependent on the visit week. Specifically, the first alcohol challenge session (AC1) and the first self-administration session (SA1) will be scheduled to take place between Week 0 and 1. The second alcohol challenge session (AC2) and the first self-administration session (SA2) will be scheduled to take place between Week 8 and 9 (0.5mg treatment phase), allowing for minor scheduling adjustments as noted above. Prior to the self-administration session only (which is longer in duration), participants will consume a standardized, isocaloric meal in advance of the alcohol self-administration procedures, with a standardized wait time between completion of the meal and onset of beverage consumption. The two session types will be scheduled with at least one interim day scheduled between sessions when possible. For both sessions, participants will be instructed to expect to remain in the lab for a full day (e.g., 8 hours).

Alcohol Challenge Sessions (AC1, AC2)

The AC sessions are designed to generate data to estimate within-person changes in acute responses to alcohol as a function of medication phase (baseline vs. 0.5mg). Following baseline assessments (described above), participants will be asked to consume a pre-determined dose of alcohol calculated to achieve a target peak breath alcohol concentration (BrAC) of .06g%. This BrAC level reflects a moderate alcohol dose. Individualized doses will be calculated using established formulas [83] that account for determinants of total body water (e.g., height, weight, sex, age). Beverages will consist of 80-proof vodka combined with a non-alcoholic, sugar-free mixer at a 1:5 ratio. Beverages will be allocated to three equal portions in separate cups. Participants will be asked to consume the beverages at an even pace over 10 minutes. A separate placebo session is not incorporated for the AC sessions, because our primary interest is in examining *within-person* changes in laboratory alcohol responses as a function of medication dose (baseline vs. 0.5mg), and because our primary outcome is self-administration (described below). Although a limitation of this approach is the inability to dissociate expectancy from pharmacological effects in measuring subjective responses to alcohol, increasing the number of sessions to dissociate expectancy effects is not a major aim for this preliminary project.

After completion of the beverages, participants will wait 10 minutes (to allow an absorption period), then will complete an initial breath alcohol concentration (BrAC) reading. BrAC readings will be repeated at 10-minute intervals during the first hour of the session (peak BrAC is anticipated to occur around 30-40 minutes, on average, with variability across subjects). Participants will repeat the battery of self-report questionnaires (outlined below) and physiological assessments at three points on the ascending limb of the blood alcohol curve: upon the first BrAC reading that meets or surpasses .02g%, .04g%, .06g%. To account for the fact that some participants may not reach the .06g% target, the latter assessment will be conducted if/when it appears the participant will not reach the .06g% target (as evidenced by two consecutive descending BrAC readings after surpassing the .05g% mark).

Following completion of the last set of measures (.06g%), participants will then be escorted to a private room to begin the recovery phase, during which they will receive water and will be monitored, and take bathroom breaks as needed. During this time, BrAC measurements will continue every 15 minutes. On the descending limb of the blood alcohol concentration curve, participants will complete follow-up measures and physiological readings at the first BrAC readings that meet or fall below .04g% and .02g%. Prior to discharge, participants will be asked to provide an additional blood glucose sample (via fingerpick) to assess any differential changes in glucose levels following alcohol administration as a function of medication phase. Participants will also complete the Food Purchase Task to assess motivation for food following alcohol administration. After this point, participants will be provided with a lunch and snacks to consume during the recovery period. Participants will be compensated and discharged once BrAC reading reaches .00g% (to allow for estimating alcohol elimination rate across sessions AC1 and AC2 as a function of medication group).

Subjective Measures. At baseline (prior to alcohol ingestion) and each subsequent assessment point noted above, participants will complete several measures. Measures of subjective alcohol effects will include the Biphasic Alcohol Effects Scale (BAES) [84], the Alcohol Urge Questionnaire (AUQ) [85] and Drug Effects Questionnaire (DEQ) [86]; the latter will be supplemented with additional items to assess potential effects related to medication (e.g., “tired”, “confused”, “nauseous”, “dizzy”, “sick”, “drunk/intoxicated”) [16]. The Alcohol Purchase Task [45] Cigarette Purchase Task [87] Food Purchase Task [78] and Monetary Choice Questionnaire [76]will be also administered at the corresponding intervals noted above. Changes in mood will be assessed using the Profile of Mood States – Short Form (POMS-SF) [88]. During the recovery period, participants will complete another battery (all measures noted above) once reaching .04g% and .02g% on the descending slope of the blood alcohol curve.

Alcohol Self-Administration Sessions (SA1, SA2).

Medication effects on laboratory alcohol consumption will be examined using a validated oral self-administration procedure. Pre-session instructions and arrival procedures/tests will be identical to those noted above. After completing baseline physiological and baseline questionnaires, participants will be presented with their preferred beverage (beer, wine, or mixed drink). Participants will elect when to begin drinking and will then complete a 2-hour *ad libitum* self-administration period. Participants will have access to their beverage at a volume calculated (based on the person’s sex, weight, height, and age using established formulas) intended not to exceed a maximum predicted peak blood alcohol concentration .12g% over the consumption period of two hours (should the participant consume all of the beverages). Participants will be instructed to consume the amount of alcohol they wish (i.e., they are not required to drink all of the beverages). Participants will be seated in a comfortable chair/sofa and will view a neutral video (e.g., nature documentary) during the session. Beverages will be presented to the participant at the onset of the two-hour period, along with ice if requested. Participants will repeat rounds of self-report assessments (described above) at 30-minute intervals during the *ad libitum* phase (i.e., at 60, 90, and 120 min. after the end of the priming phase), with physiological assessments and BrAC readings taken at these intervals (or more frequently if indicated). The primary outcomes for this session are peak BrAC and volume of alcohol consumed.

Participants will then be escorted to a private room for the recovery period. Assessments during the recovery period will be identical to those described above. Participants will be monitored until being discharged when BrAC declines below .03% for two consecutive readings, consistent with NIAAA recommended guidelines. [52] As noted, participants will be instructed not to drive, and will be asked to arrange for a ride home from the study site. If a participant cannot arrange a ride home, they will be provided with reimbursement for a ride home via a rideshare service (e.g., Uber or Lyft) up to $25. If a participant reports having driven to a session, they will be required to remain at the site until BrAC is

measured at 0mg% for two consecutive readings. At alcohol sessions, participants may also be provided with a wristband transdermal alcohol monitor (BACTrack Skyn) to wear during the session, which will provide a continuous readout of estimated alcohol concentration (measured as total alcohol concentration in sweat). Estimated transdermal alcohol concentration will not be a primary outcome - as it is not an accurate measure of blood alcohol concentration - but will be collected for purposes of secondary data analyses. Specifically, we will record readouts from the transdermal device at regular intervals to compare to BrAC readings, which will inform our use of this device in future studies. Because we will only have one transdermal device available, it may not be used in all sessions (depending on availability).

<u>List of Study Assessment Measures (See Section 1.3 for assessment time points)</u>

1. Demographics form
2. Timeline Follow-Back (TLFB)
3. Structured Clinical Interview for DSM-5 Mental Disorders (select modules)
4. MINI Neuropsychiatric Interview (select modules)
5. Sweet Taste Preference Test
6. Alcohol Use Disorder Identification Test (AUDIT)
7. Self-Report of the Effects of Alcohol Questionnaire (SRE)
8. Center for Epidemiological Studies Depression Scale (CES-D)
9. Toronto Alexithymia Scale (TAS-20)
10. Barratt Impulsivity Scale (BIS-11)
11. UPPS Impulsive Behavior Scale (UPPS)
12. Reward Probability Index – Environmental Reward Observation Scale (RPI-EROS)
13. Impaired Control Scale (ICS)
14. Fagerstrom Test for Nicotine Dependence (FTND)
15. Nicotine Dependence Syndrome Scale (NDSS)
16. Penn Alcohol Craving Scale (PACS)
17. Tiffany Questionnaire of Smoking Urges (TQSU)
18. Wisconsin Smoking Withdrawal Scale (WSWS)
19. Wisconsin Inventory of Smoking Dependence Motives (WISDM)
20. Alcohol and Smoking Contemplation Ladder
21. Three Factor Eating Questionnaire (TFEQ)
22. Reward-Based Eating Drive Scale (R-BEDS)
23. Control of Eating Questionnaire (CEQ)
24. Simplified Nutritional Appetite Questionnaire (SNAQ)
25. Monetary Choice Questionnaire (MCQ)
26. Cigarette Abstinence Self-Efficacy (CASE)
27. Alcohol Abstinence Self-Efficacy (AASE)
28. Inventory of Drinking Situations (IDS)
29. Biphasic Alcohol Effects Scale (BAES)
30. Alcohol Urge Questionnaire (AUQ)
31. Drug Effects Questionnaire (DEQ)
32. Alcohol Purchase Task (APT)
33. Cigarette Purchase Task (CPT)
34. Food Purchase Task (FPT)
35. Profile of Mood States – Short Form (POMS-SF)
36. Systematic Assessment for Treatment Emergent Effects (SAFTEE)
37. Prediction of Alcohol Withdrawal Severity Scale (PAWS)

38. Short Alcohol Withdrawal Scale (SAWS)
39. Obsessive Compulsive Drinking Scale
40. Graded Pain Scale

Analysis schedule. Analyses of side effect frequency and AE/SAE frequency will be conducted prior to scheduled DSMB meetings. Other analyses will be conducted following the completion of the trial, or as otherwise required to fulfill DSMB requests or other reporting requirements.

4.2 SCIENTIFIC RATIONALE FOR STUDY DESIGN

This short-term trial is intended to produce initial data on the effects of semaglutide in non-treatment-seeking participants with AUD, with an emphasis on human laboratory outcomes. The dose escalation schedule is based on the standard clinical regimen, which is intended to minimize side effects common to GLP-1 agonists (i.e., nausea). Participants in the placebo group will complete an identical sequence while receiving sham subcutaneous injections (a small needle stick using a micro-needle of the same size, without an injection). This placebo condition is selected due to the absence of an available matching placebo cartridge from the manufacturer (see section 4.3). All participants will complete laboratory alcohol administration sessions at two time points, corresponding with the baseline and 0.5mg phases. This design allow for within-subjects analyses for laboratory outcomes that are best examined at the within-subject level (owing to high inter-individual variability in alcohol pharmacokinetics). Overall, this design is intended to maximize feasibility and sample size while also accommodating the extended dosage titration period required of GLP-1 agonists. Findings from this trial will be used to justify a larger, placebo-controlled Phase II clinical trial application via the R01 mechanism.

4.3 JUSTIFICATION FOR DOSE

Semaglutide (Ozempic®) will be administered according to the FDA-approved schedule, which begins with a starting dose of 0.25mg (for titration purposes) once per week for four weeks. At week five, dosage is increased to 0.5mg/week (the initial first maintenance dose) for a subsequent four weeks, at which point dosage can be increased further (1.0mg) if indicated (and presuming that the 0.5mg dose has been tolerated). The intent for this initial trial is to mimic the typical clinical scenario over the first several weeks of medication titration. Although primary analyses will focus on the 0.5mg dosage, participants will receive one week of 1.0mg treatment at the conclusion of the 0.5mg phase, in order to collect initial data on side effects and naturalistic drinking/cigarette use at this dosage in the current population. Participants will be monitored carefully and will not be escalated to the next dose if experiencing persistent side effects indicative of poor tolerability.

Given the lack of a true placebo (i.e., no manufacturer-developed placebo Ozempic® pen/cartridge), this study will use a placebo condition that consists of weekly sham doses. Sham doses will involve a brief needle prick using a very small (e.g., 32 gauge) needle. These procedures are described in more detail later in this protocol. By necessity, medical staff administering the medication cannot be blind to condition. However, other experimenters and outcomes assessors will remain blind to condition. Participants will be instructed during the informed consent and study orientation that they have a 50/50 chance of being randomized to medication and placebo groups.

4.4 END OF STUDY DEFINITION

A participant is considered to have completed the study if he or she has completed all phases of the study including the last visit shown in the Schedule of Activities (SoA), Section 1.3. The end of the study is defined as completion of the last visit shown in the SoA in the trial overall.

5 STUDY POPULATION

5.1 INCLUSION CRITERIA

In order to be eligible to participate in this study, an individual must meet the following criteria:

- Age 21-65
- Meeting DSM-5 criteria for current (past year) AUD, with between 2-7 symptoms endorsed, and NIAAA criteria for current at-risk drinking (i.e., >7/14 drinks in one week for women/men, with at least two episodes of 4+/5+ drinks in the past 30 days)
- Willingness/availability to take study medication and complete study procedures, including attending weekly visits for medication administration, side effect assessments, and glucose monitoring
- Willingness to complete laboratory sessions involving alcohol administration
- Ability to communicate and read in English

5.2 EXCLUSION CRITERIA

- Reporting past 30-day use of illicit drugs other than cannabis at baseline, or having a positive toxicology screen for illicit drugs other than cannabis at baseline
- Meeting past-year criteria for a substance use disorder (with the exception of alcohol, tobacco or mild cannabis use disorder)
- Current engagement in alcohol treatments, or currently engaged in intentional efforts to quit alcohol use
- Past 30-day use of: Sincalide, Sulfonylureas, insulin and insulin products or other medications that may interact with semaglutide;, or weight control medications
- Prior use of semaglutide or other GLP-1 agonists
- Known or suspected hypersensitivity to study medication or related products
- Lifetime diagnosis of severe mental illness (including schizophrenia and bipolar disorder)
- History of suicide attempt, or recent (past 30 day) suicidal ideation, or psychiatric hospitalization in the last 6 months
- Current significant medical or neurological illness (based on self-report or medical record) including severe hepatic impairment or cirrhosis, impaired renal function (eGFR <50ml/min), acute or chronic pancreatitis, gastroparesis, gallbladder disease or cholelithiasis, other severe gastrointestinal disease, heart failure, coronary artery disease, stroke, seizure disorder, or other

medical condition that poses a risk for the medication or alcohol administration components of the study (as determined by the MD)

- A personal or family history of medullary thyroid cancer or multiple endocrine neoplasia 2A or 2B
- Calcitonin greater than or equal to 50 ng/L
- Uncontrolled thyroid disease at screening
- History of major surgical procedures involving the stomach potentially affecting absorption of trial product (e.g., subtotal and total gastrectomy, sleeve gastrectomy, gastric bypass surgery)
- History of Type 1 or Type 2 diabetes, or HbA1c >6.5% measured at screening
- History of diabetic retinopathy, proliferative retinopathy, or maculopathy
- History of diabetic ketoacidosis
- History or presence of malignant neoplasms within the last 5 years (except basal and squamous cell skin cancer and carcinoma in situ)
- Currently nursing, pregnant, anticipating pregnancy in the next 6 months, or not using a highly effective contraceptive method as judged by the MD, and defined as:
    a. combined (estrogen and progestogen containing) hormonal contraception associated with inhibition of ovulation (oral, intravaginal, transdermal)
    b. progestogen-only hormonal contraception associated with inhibition of ovulation (oral, injectable, implantable)
    c. intrauterine device
    d. intrauterine hormone-releasing system
    e. bilateral tubal occlusion
    f. vasectomized partner
    g. sexual abstinence
- Elevation of serum lipase, amylase, direct (conjugated) bilirubin, or alkaline phosphatase (ALP), ALT, or AST) more than 3X the upper limit of normal on baseline bloodwork
- Baseline body mass index (BMI) <23kg/m2
- Uncontrolled hypertension or systolic BP >180 mmHg and/or diastolic BP >105 mmHg, averaged from three measurements
- Plans for travel outside of the local area in the upcoming 12 weeks that would interfere with lab visits during the study period (or other logistic factors that would make it difficult to commit to entire duration of study)

5.3 LIFESTYLE CONSIDERATIONS

Specific lifestyle instructions will include:

- Refrain from alcohol consumption or other drug use for 24 hours prior to alcohol administration sessions
- Arrive fasting at sessions scheduled for blood draws as needed

- Refrain from consuming food for 4 hours prior to alcohol sessions. Participants will receive a standardized snack on arrival for alcohol sessions.)
- Refrain from driving to the lab on the day of alcohol sessions. (Participants will be asked to arrange for transportation, or will be provided with transportation home via taxi or Uber)
- Refrain from operating other vehicles or machinery for the duration of day upon discharge from alcohol sessions
- Women participating in the study must report being willing to use a reliable birth control method during the trial (if sexually active), and must report no plans for pregnancy in the next 6 months.
- Participants will also understand that they may be excluded if they test positive for drugs other than cannabis during the time of scheduled drug screens (repeat drug tests will take place during weeks 4 and 8).

5.4 SCREEN FAILURES

Screen failures are defined as participants who consent to participate in the clinical trial but are not subsequently randomly assigned to the study intervention or entered in the study. A minimal set of screen failure information is required to ensure transparent reporting of screen failure participants, to meet the Consolidated Standards of Reporting Trials (CONSORT) publishing requirements and to respond to queries from regulatory authorities. Minimal information includes demography, screen failure details, eligibility criteria, and any serious adverse event (SAE).

Examples of screen failures would include participants excluded following baseline laboratory work, or those excluded based on a change in treatment-seeking status at the time of the baseline assessment. Individuals who do not meet the criteria for participation in this trial (screen failure) because of a specific modifiable factor (e.g., upcoming travel) may be rescreened at a later date. Rescreened participants will be assigned the same participant number as for the initial screening. Data pertaining to screening failures will be tabulated in an anonymized fashion.

5.5 STRATEGIES FOR RECRUITMENT AND RETENTION

Participants will be recruited from the community through advertisements, which may include ads in local online or print newspapers, advertisements in buses, print flyers placed in community or hospital settings, business cards, campus listservs, and online postings (e.g. Craigslist (ads will not be placed in employment section)), and social media ads (Facebook, Twitter, Instagram) that direct potential participants toward a study website/landing page (hosted on Research for Me). Participants may also be recruited from local medical clinics (e.g., primary care clinics) that grant approval for assisting with recruitment (e.g., by placing study advertisements in clinics). Flyers and online advertisements will include study contact information and a web link and/or QR code leading to the landing page.

In addition to these “passive” methods of recruitment, we will also include “active” recruitment methods. These methods will include members of the study team setting up recruitment booths and passing out business cards at public events (e.g., festivals) or public venues. Once initial contact with participants is made (e.g., via email or in person), research staff will schedule the participant for a phone call to explain the study and conduct a screening interview.

Participants will receive compensation. The payments will be allocated as follows (please see 1.2 for a summary of all scheduled sessions):

- $50 for completing the consent/screening/baseline sequence (Week 0)
- $75 for alcohol challenge session 1 (To be scheduled between Weeks 0-1)
- $75 for alcohol self-administration session 1 (To be scheduled between Weeks 0-1)
- $100 for alcohol challenge session 2 (To be scheduled between Weeks 9-10)
- $100 for alcohol self-administration session 2 (To be scheduled between Weeks 9-10)
- $30 per visit for each follow-up/medication administration visit during Weeks 1-10 (10 visits x$30 per visit = $300)
- Up to $150 for completing and returning daily questionnaires over the study period ($15 per weekly set of surveys x 10 weekly visits), which includes the week following the 1.0mg dose, to be paid at weekly medication visits)
- A $50 bonus for completing at least 9 of 10 weekly assessments
- Participants will receive a final bonus of $100 for completing the study through Week 10 (to be paid at the final discharge visit).

Therefore, the total maximum compensation over 10 weeks for study visits is $1000. Compensation for each pair of the alcohol sessions will occur at the end of the second of the session pair. The final payment (including bonuses) will be paid at the Week 10 discharge visit. Overall, these payments are structured to maximize retention in the study (given the long dose escalation period and time-intensive laboratory sessions). Additionally, an incentive-based structure that includes weekly incentives is important to reinforce engagement with daily surveys, and to minimize missing data. Participants will be updated at each visit with their progress toward the bonus incentives for adherence (e.g., weekly bonus of $10 and progress toward final adherence bonuses). In the event participants are not able to pick their final payments in person, we will offer the option of issuing the final payment via electronic gift card to the participant’s email address, with their permission.

We anticipate phone screening up to 400 individuals and randomizing up to 48 participants to medication or placebo conditions, in order to obtain a final sample of at least 36 eligible participants who complete the full course of the 0.5mg phase (allowing for 25% attrition). If attrition is higher than anticipated, we will submit an amendment to enroll additional participants to arrive at a final sample of at least 36 participants who complete study procedures through the end of the 0.5mg dosage phase (i.e., through the end of Week 9).

6 STUDY INTERVENTION

6.1 STUDY INTERVENTION(S) ADMINISTRATION

6.1.1 STUDY INTERVENTION DESCRIPTION

Semaglutide is a relatively new (approved in 2017) GLP-1 receptor agonist. A new oral formulation of semaglutide was approved by the FDA in 2019, making semaglutide the first drug in its class to be available in both subcutaneous and oral formulations. GLP-1 agonist medications were designed to mimic the function of the endogenous GLP-1 peptide, with the benefit of a much longer half-life than endogenous GLP-1. Based on their glucose-lowering and appetite-reducing properties, GLP-1 agonists

were developed as anti-diabetic therapies. Several GLP-1 agonists (e.g., exenatide, liraglutide, lixisenatide, albiglutide, dulaglutide and semaglutide,) are now in widespread use as treatments for type II diabetes (T2D); these medications have also proven efficacious for weight loss, reducing glycated hemoglobin (HbA1c), and reducing risk for some cardiovascular events [29]. Although the primary indication of GLP-1 agonists is T2D, these therapies are increasingly being studied for other indications in non-diabetic patients, including obesity/weight management. In June 2021, the FDA approved semaglutide at a higher dosage (2.4mg/week) for the indication of weight management, under the brand name Wegovy.

6.1.2 DOSING AND ADMINISTRATION

Semaglutide will be administered according to the FDA-approved schedule, which begins with a starting dose of 0.25mg once per week for four weeks. At week five, dosage is increased to 0.5mg for a subsequent four weeks (initial maintenance dose), after which point dosage can be increased further (to 1.0mg), presuming the medication is well tolerated.

Although semaglutide can be self-administered in the home setting, medication and placebo administration will be delivered by a research nurse or other medical team member at Eastowne CRU or CTRC in order to execute the placebo manipulation and ensure adherence to medication. This approach also allows us to collect safety outcomes on a regular basis, further ensuring that participants will be monitored carefully. Semaglutide is administered subcutaneously (in the abdomen, thigh, or upper arm). Doses are delivered via the Ozempic® pen, a cartridge-based device that calibrates medication delivery based on the desired dose. An extremely small needle (4mm, 32-gauge needle – the size of 2 human hairs) is used to deposit the medication subcutaneously. Because each pen contains 2mg of medication, each participant in the medication group will be allocated one pen to cover the first six weeks (i.e., 0.25mg/week for 4 weeks, followed by 0.5mg/week for 2 weeks), and a second pen for that will supply medication for the remaining three weeks (2 weeks at 0.5mg, and 1 week at 1.0mg). This schedule results in 9 medication administration sessions. At the week 9 visit, the 1.0mg dose will be delivered as 2 x 0.5 mg doses given in succession. This is because the medication pens being used for this study deliver dosages of 0.25 and 0.5mg, and participants are not being maintained on the 1.0mg dose after week 9. This timeframe also allows sufficient time to reach steady-state plasma concentration levels (achieved within 4-5 weeks of initiating the starting dose).

At the present time, we do not have access to a manufacturer-developed placebo (the medication relies on the proprietary Ozempic® pen for delivery). Therefore, this study will use weekly sham injections for the placebo group. Sham doses will involve a brief needle prick using a very small (e.g., 32 gauge) needle, identical to the one used in the Ozempic® pen (NovoFine), or a needle of similar gauge (e.g., an insulin needle). Given the extremely small volume of medication delivered in the treatment condition, we will not utilize saline or another inert substance for the placebo administration. During medication and placebo administration, participants' view will be shielded from the injection site to prevent unblinding. Medication will be delivered in the upper arm, abdomen, or thigh. Given the extremely small volume of the injections, we will not utilize saline or another inert substance for the placebo administration; rather, the sham doses will consist of briefly inserting the micro-needle.

Due to the nature of the placebo condition, it will not be possible to blind the staff RNs administering the medication. However, we will provide training to study staff to minimize the likelihood of contaminating the blinding procedures. We will also develop procedures to minimize contact between

the RNs and outcome assessors. All other staff members (including research assistants and outcomes assessors) will remain blind to condition, unless unblinding is necessary (e.g., due to an adverse event).

6.2 PREPARATION/HANDLING/STORAGE/ACCOUNTABILITY

6.2.1 ACQUISITION AND ACCOUNTABILITY

The team will collaborate with IDS to source the medication (and NovoFine or insulin needles for the placebo condition), which will be sourced and stored at the IDS pharmacy prior to dispensing. Participants assigned to medication will be prescribed one Ozempic® pen following randomization. As per IDS requirements, in between study visits participants will be given their assigned medication pen and asked to bring their pen back to each follow-up visit. In between visits, pens will be placed in a small plastic container and then encased opaque, tamper-proof bag in order to prevent unblinding. (For participants in the placebo condition, a plastic object of roughly equal size and weight will be placed in the plastic container and medication bag.) Participants will be instructed not to open the bag in between visits. A refill pen will be prescribed prior to the Week 7 visit, once the doses from the first pen have been allocated. Any unused doses from individual pens (e.g., in the event of participant drop-out) will be discarded/destroyed by IDS, as will any pens that expire during the course of the trial. In the event a pen expires before a participant completes the study, a replacement pen will be prescribed at the discretion of the study physicians.

6.2.2 FORMULATION, APPEARANCE, PACKAGING, AND LABELING

Ozempic® is manufactured by Novo Nordisk. As described above, the medication is dispensed from a proprietary device, the Ozempic® pen. Each pen contains 2mg of medication, requiring that each participant is prescribed two pens over the course of the study. A full description and visuals can be found at the following site:
https://www.Ozempic®pro.com/how-to-prescribe/Ozempic®-pen.html

The *placebo group* will receive sham injections that involve a brief needle-prick without dispensing a substance. The needles used for the sham injections will be the NovoFine 32G needle, the same needle in the Ozempic® pen (https://www.novoneedles.com/novofine.html) or a comparable needle of a similar gauge (e.g., an insulin needle). In no case will any fluid be injected during placebo sessions. To mimic the active medication administration, the medical staff member will insert the microneedle briefly and then remove it while the participant's view is shielded.

6.2.3 PRODUCT STORAGE AND STABILITY

Pens will be dispensed from IDS. For drug dispensing visits at CRU (Eastowne), scheduled for Weeks 1 and 7, pens will be delivered the day of the visit by courier. Ozempic pens will be kept in the participant's possession (sealed in a tamper-proof bag) between visits. According to the manufacturer, following the first use, the Ozempic® pen can be stored for up to 56 days at room temperature (59 to 86 F) or refrigerated at 36 to 46 F. As per our plan developed in collaboration with IDS, participants will be instructed to keep the medication bag at room temperature for simplicity. Each participant's pen will be labeled with the study ID number and pens will not be shared across participants. Placebo needles will remain packaged until use, and will be stored at room temperature in a secure location.

6.2.4 PREPARATION

Semaglutide does not require advance preparation; cartridges are pre-filled and doses are calibrated using the pen device. The RN or medical staff member will select the correct dosage and administer the medication as per manufacturer guidelines.

6.3 MEASURES TO MINIMIZE BIAS: RANDOMIZATION AND BLINDING

Following the baseline assessment, participants will be randomized to medication or placebo conditions at a 1:1 ratio by a member of the IDS team using a pre-determined randomization scheme. Medication and placebo administration will be carried out by a staff RN at CRU or CTRC. Due to the nature of the placebo condition, it will not be possible to blind the RNs to condition. However, all other staff members will remain blind to condition, and the staff members administering medication will be trained in protecting the blind (e.g., redirecting the conversation if the participant comments about medication condition; minimizing contact with experimenters.) To maintain blinding of participants, participants' view will be shielded and they will be asked to face away from the injection site during all placebo and medication administrations. Aside from the staff members administering medication, no study team members interacting with participants will be aware of participants' assignments. At the stage of interim analyses (e.g., in preparing safety reports for DSMB meetings) and final analyses, the study statistician will be provided with the assignment log. None of the study personnel involved in recruitment or enrollment will have knowledge of the details of the randomization table that will be used by the IDS pharmacy.

At the completion of the trial, participants will be provided with a questionnaire asking about perceived medication assignment (to assess the success of maintaining the blind). Due to common side effects of semaglutide (in particular nausea), we do not expect blinding to be successful in all cases.

6.4 STUDY INTERVENTION COMPLIANCE

Medication will be administered by CRU or CTRC staff to assure compliance and reduce potential sources of non-adherence. A missed session will constitute a missed medication dose. As per manufacturer directions, any missed doses/appointments will be corrected by asking the participant to come in for the dose as soon as possible within 5 days of the missed dose. After the 5-day mark has passed, the dose will be skipped, and the participant will resume with the next scheduled dose. Study medication logs will serve as the primary source document for tracking medication and placebo administration. Participants will be coded as non-adherent if they miss more than 2 scheduled doses between Weeks 1-9. In the event of missed medication doses or missed appointments, the physician will have the discretion to adjust the appointment date (within the manufacturer or standard practice guidelines), or to prescribe a replacement pen if needed to accommodate the target medication schedule.

6.5 CONCOMITANT THERAPY

Participants may be taking medications for other conditions (provided those treatments are not included in the list of exclusion criteria). For this protocol, a prescription medication is defined as a medication that can be prescribed only by a properly authorized/licensed clinician. Medications to be

reported in the Case Report Form (CRF) are concomitant prescription medications, over-the-counter medications and supplements. Changes to medication status or other treatments will be assessed at each in-person visit, with any changes reviewed by a study physician. Should a concomitant therapy preclude further participation for safety or scientific reasons (for instance, if a participant starts a therapy that constitutes an exclusion factor), this issue will be discussed by the study leads and the participant will be excused from further participation, if required to ensure safety. By definition, participants will not be seeking treatment for alcohol use or smoking cessation. If a participant makes the decision to seek treatment for alcohol or smoking during the study, s/he will be referred to appropriate treatment resources and excused from further participation.

6.5.1 RESCUE MEDICINE

Not applicable

7 STUDY INTERVENTION DISCONTINUATION AND PARTICIPANT DISCONTINUATION/WITHDRAWAL

7.1 DISCONTINUATION OF STUDY INTERVENTION

Study discontinuation: The study may be halted if deemed necessary by the lead investigators, DSMB, or IRB. Examples of reasons for study discontinuation include unexpectedly high frequency of serious adverse events, or significant increases in alcohol use during treatment, leading to the conclusion that the medication is not safe in this population. In addition to the participant discontinuation rules noted below, the following study stopping rules will trigger halting the study pending a DSMB evaluation: 1) Occurrence of any hospitalization or any SAE rated at Grade 4-5 (CTCAE) in a medication-treated participant (and deemed likely to be medication-attributable); 2) Documentation of SAEs in >3 medication-treated participants that are deemed likely attributable to medication; 3) Removal of >5 subjects due to adverse events deemed attributable to study-related procedures, inclusive of SAE and/or medical stopping rules (e.g., weight, alcohol consumption, disease progression), as defined in 7.2 below. In order to classify events as medication-attributable, the PI will request that IDS breaks the blind and provides a file containing condition assignment to the study physician(s). If any of the aforementioned events trigger a DSMB meeting, the full randomization table will be provided to a statistician, so that group differences can be fully evaluated at the meeting. Given the safety profile of semaglutide for other clinical conditions, as well as preclinical evidence that GLP-1 receptor agonists reduce drug intake, these scenarios are expected to be unlikely.

7.2 PARTICIPANT DISCONTINUATION/WITHDRAWAL FROM THE STUDY

Participants are free to withdraw from participation in the study at any time upon request. The investigators may discontinue or withdraw a participant from the study if clinically or otherwise indicated. Examples of reasons removing a participant include:

- Significant study intervention non-compliance
- Any clinical adverse event (AE), laboratory abnormality, or other medical condition or situation that indicates that continued participation in the study would be unsafe

- Pregnancy
- Diagnosis of significant disease or disease progression which requires discontinuation of the study intervention
- The participant meets an exclusion criterion (either newly developed or not previously recognized) that precludes further study participation
- Participant relocates from the local area, or is otherwise unable to attend visits
- Significant or persistent side effects that pose significant discomfort or preclude escalation to the 0.5mg dose (e.g., persistent significant nausea, or hypoglycemia, as indicated by repeated fasting glucose values below normal 54 mg/dL
- Changes in lab tests or clinical status from baseline that render the participant ineligible to continue in the judgement of the physician(s) (e.g., elevated liver enzymes, elevated serum amylase or lipase levels, weight loss as defined below)
- Excessive weight loss (defined as loss exceeding 5% of baseline weight or BMI < 20)
- A change in treatment-seeking status (i.e., the participant expresses a desire to enter treatment for alcohol or smoking cessation).
- A significant increase in alcohol consumption, as evidenced by a >50% increase in drinks per week (relative to baseline) lasting more than two weeks. Drinks per week in the 30 days preceding the Week 0 visit (measured by the TLFB) will serve as the baseline measure.
- Evidence of significant alcohol withdrawal symptoms
- Any other change in medical status or side effects that is significant enough to warrant removal in the judgement of the study physicians

If a participant exhibits changes in symptoms or clinical status that warrant potential removal, the study physicians will confer and render a decision as to whether to withdraw the participant. With respect to dosage increase, if a participant's side effects profile or laboratory tests during the initial 4-week phase (.25mg) indicate that s/he is unable to safely proceed to the 0.5mg dose, the participant may be removed from the study at the discretion of the physicians. If lab tests or side effects during the standard maintenance dose of 0.5mg suggest that a participant is unlikely to tolerate a higher dose (1mg), they may be excluded from the 1.0mg dose escalation at the discretion of the physicians. In this case, participants will be given the option of one additional week at the standard therapeutic dose of 0.5mg (i.e., highest tolerated dose). Reasons for participant discontinuation or withdrawal from the study will be recorded on the Case Report Form (CRF). Participants who sign the informed consent form but do not receive the study intervention (e.g., fail to attend the Week 1 visit for randomization) may be replaced. Participants who sign the informed consent form, and are randomized and receive the study intervention, and subsequently withdraw, or are withdrawn or discontinued from the study, may be replaced.

7.3 LOST TO FOLLOW-UP

A participant will be considered lost to follow-up if he or she fails to return for 2 consecutive scheduled medication visits and is unable to confirm attendance at the following visit (or is unable to be contacted by the study staff

The following actions will be taken if a participant fails to return to a required study visit:

- The team will attempt to contact the participant and reschedule the missed visit within one week, and counsel the participant on the importance of maintaining the assigned visit schedule and ascertain if the participant wishes to and/or should continue in the study.
- The team will attempt to contact the individuals that the participant had listed on the Locator Form at baseline (maximum of three attempted contacts per individual).
- Before a participant is deemed lost to follow-up, the investigator or designee will make every effort to regain contact with the participant (where possible, up to 3 telephone messages, up to 3 email messages, and if necessary, a letter to the participant's last known mailing address or local equivalent methods). These contact attempts will be documented in the participant's medical record or study file.
- Should the participant continue to be unreachable, he or she will be considered to have withdrawn from the study with a primary reason of lost to follow-up.

8 STUDY ASSESSMENTS AND PROCEDURES

8.1 EFFICACY ASSESSMENTS

The efficacy assessments/outcomes are noted in summary form in section 3. The ascertainment of these outcomes is described in further detail in section 4 (study design). Briefly, the endpoints are human laboratory measures of alcohol consumption and alcohol response; self-reported alcohol consumption and cigarette use over the course of the trial; and changes in weight, HbA1c, and alcohol elimination.

8.2 SAFETY AND OTHER ASSESSMENTS

Safety and assessments are summarized in detail in the study design section (Section 4.1). Briefly, safety assessments include a baseline assessment, body weight, bloodwork, vitals signs, and clinical interview to assess substance use and psychiatric symptoms. Follow-up medical visits will include repeat assessments of bloodwork and scheduled time points, as well as follow-up measurements of body weight and vitals signs. Medication side effects will be assessed at each visit, and a study team member will contact participants within 2-3 days of each dosage increase to evaluate potential side effects. Other assessments (questionnaires, interviews) are noted in Section 4.1.

Safety precautions during alcohol administration sessions. Our team has considerable experience with alcohol administration procedures. Our procedures follow the NIAAA guidelines for administering alcohol in human studies (https://www.niaaa.nih.gov/research/guidelines-and-resources/administering-alcohol-human-studies). Based on these guidelines, alcohol administration studies with participants who meet criteria for AUD are typically restricted to non-treatment-seeking participants in order to avoid ethical concerns, as will be the approach in this study. Additionally, our approach in this study is to only recruit people who meet criteria for AUD with <8 of out of 11 symptoms endorsed. Regarding the alcohol dose for alcohol challenge sessions, we will use sex- and body weight adjusted formulas [70] to ensure that dosage is calibrated across participants to achieve the target BrAC (.06g%). Participants will have undergone a medical screen prior to participation, and will be excluded if the study medical staff has reason to believe that alcohol is contraindicated. Participants will receive a BrAC reading on arrival to the alcohol sessions; the session will be rescheduled if the participant presents with a positive BrAC.

Following the completion of alcohol sessions, participants will be escorted to a private room designated for recovery purposes. During alcohol self-administration sessions, participants will only be discharged after their breath alcohol concentration descends below .03g%, which is consistent with NIAAA guidelines. During alcohol challenge sessions, participants will be asked to remain in the lab until BrAC reaches 0mg% (in order to collect BrAC data until alcohol is fully eliminated, for purposes of pharmacokinetic analyses). Participants will also be provided with reimbursement for transportation if they do not drive and are not able to locate assistance with transportation (participants will be advised not to drive to the alcohol sessions). If a participant presents with a positive BrAC, the session will be rescheduled, and the participant will be asked to remain in the lab until it is safe to leave.

To ensure participant safety, at least two members of the study staff will be present for the duration of all alcohol administration sessions. If an urgent medical situation arises at either medication or alcohol visits, a team member will contact the physicians on the team to consult, based on a predetermined sequence (e.g., contact physician A, followed by physician B, then physician C). Additionally, any participants who express a desire to seek treatment during the study will be excused from further participation and provided with alcohol or smoking cessation resources. Co-I Dr. Jordan directs outpatient addiction treatment at UNC-CH.

8.3 ADVERSE EVENTS AND SERIOUS ADVERSE EVENTS

8.3.1 DEFINITION OF ADVERSE EVENTS (AE)

Adverse event means any untoward medical occurrence associated with the use of an intervention in humans, whether or not considered intervention-related (21 CFR 312.32 (a)).

8.3.2 DEFINITION OF SERIOUS ADVERSE EVENTS (SAE)

An adverse event (AE) or suspected adverse reaction is considered “serious” if, in the view of either the investigator or sponsor, it results in any of the following outcomes: death, a life-threatening adverse event, inpatient hospitalization or prolongation of existing hospitalization, a persistent or significant incapacity or substantial disruption of the ability to conduct normal life functions, or a congenital anomaly/birth defect. Important medical events that may not result in death, be life-threatening, or require hospitalization may be considered serious when, based upon appropriate medical judgment, they may jeopardize the participant and may require medical or surgical intervention to prevent one of the outcomes listed in this definition. Examples of such medical events include an adverse reaction requiring intensive treatment in an emergency room or at home.

8.3.3 CLASSIFICATION OF AN ADVERSE EVENT

8.3.3.1 SEVERITY OF EVENT

For adverse events (Aes) not included in the protocol defined grading system, the following guidelines will be used to describe severity.

- **Mild** – Events require minimal or no treatment and do not interfere with the participant’s daily activities.

- **Moderate** – Events result in a low level of inconvenience or concern with the therapeutic measures. Moderate events may cause some interference with functioning.
- **Severe** – Events interrupt a participant's usual daily activity and may require systemic drug therapy or other treatment. Severe events are usually potentially life-threatening or incapacitating. Of note, the term "severe" does not necessarily equate to "serious".

8.3.3.2 RELATIONSHIP TO STUDY INTERVENTION

All adverse events (Aes) must have their relationship to study intervention assessed by the clinician who examines and evaluates the participant based on temporal relationship and his/her clinical judgment. The degree of certainty about causality will be graded using the categories below. In a clinical trial, the study product must always be suspect.

- **Related** – The AE is known to occur with the study intervention, there is a reasonable possibility that the study intervention caused the AE, or there is a temporal relationship between the study intervention and event. Reasonable possibility means that there is evidence to suggest a causal relationship between the study intervention and the AE.
- **Not Related** – There is not a reasonable possibility that the administration of the study intervention caused the event, there is no temporal relationship between the study intervention and event onset, or an alternate etiology has been established.

8.3.3.3 EXPECTEDNESS

The study physicians will be responsible for determining whether an adverse event (AE) is expected or unexpected. An AE will be considered unexpected if the nature, severity, or frequency of the event is not consistent with the risk information previously described for the study intervention.

8.3.4 TIME PERIOD AND FREQUENCY FOR EVENT ASSESSMENT AND FOLLOW-UP

The occurrence of an adverse event (AE) or serious adverse event (SAE) may come to the attention of study personnel during study visits and interviews of a study participant presenting for medical care, or upon review by a study monitor. At weekly medication visits, all participants will review a side effects checklist (SAFTEE) with a research assistant, which will then be provided for review by a medical staff member at CTRC. The staff member will begin with an open-ended question about side effects or any other adverse events, followed by a review of specific side effects using the SAFTEE. Any previously reported side effects will be queried to determine any change in status (presence or severity). These weekly visits will be a primary method for identifying side effects. Additionally, a research staff member will contact participants by phone in the week following the initial dose, as well as the week following each dose escalation. Phone calls will be scheduled 2-3 days following these events.

All Aes including local and systemic reactions not meeting the criteria for SAEs will be captured on the appropriate case report form (CRF). Information to be collected includes event description, time of onset, clinician's assessment of severity, relationship to study product (assessed only by those with the training and authority to make a diagnosis), and time of resolution/stabilization of the event. All Aes occurring while on study will be documented appropriately regardless of relationship. All Aes will be followed to adequate resolution.

Any medical condition that is present at the time that the participant is screened will be considered as baseline and not reported as an AE. However, if the study participant's condition deteriorates at any time during the study, it will be recorded as an AE.

Changes in the severity of an AE will be documented to allow an assessment of the duration of the event at each level of severity to be performed. Aes characterized as intermittent require documentation of onset and duration of each episode.

Under the direction of the PI, members of the research team will record all reportable events with start dates occurring any time after informed consent is obtained until 7 (for non-serious Aes) or 30 days (for SAEs) after the last day of study participation. At each study visit, the team member will inquire about the occurrence of AE/SAEs since the last visit. Any such events will be followed for outcome information until resolution or stabilization.

8.3.5 ADVERSE EVENT REPORTING

All project staff will be trained to identify adverse events (Aes), which must be reported to the PI and study physicians within 24 hours. In addition, any unanticipated participant issues will be discussed in weekly staff meetings, which will provide another opportunity to identify Aes.

8.3.6 SERIOUS ADVERSE EVENT REPORTING

Research staff will be trained to report potential Aes or SAEs to the PI immediately. For any potential Aes/SAEs, the study physician will make a determination to distinguish between Aes, SAEs and unanticipated problems and provide judgment about attributions. Management and reporting of Aes, SAEs, and unanticipated problems will be carried out in accordance with Good Clinical Practice (GCP) standards, as outlined by the Food and Drug Administration. Aes will be defined as any negative medical event during the trial (whether or not the event is deemed related to the study medication). SAEs are defied as events that are potentially life threatening or result in heath; cause congenital malformation; result in persistent/significant disability or incapacity; require inpatient hospitalization or prolongation of hospitalization; or reflect an otherwise notable medical event in the judgment of the study physician. Aes/SAEs will be monitored regularly by the study research team and medical staff, in particular by inquiring about adverse events at each weekly visit. Should Aes or SAEs occur, they will be recorded, rated as mild/moderate/severe by the study physician, and reported in accordance with UNC IRB requirements and GCP standards. Any Aes will be reported to the IRB in progress reports and at the conclusion of the study. SAEs and unanticipated events will be reported to the IRB and DSMB within 48 hours, and to NIAAA if judged as treatment-related. All staff working on this study will be made aware of the monitoring and reporting requirements for Aes/SAEs, and will have completed GCP training and other trainings required to conduct human subjects research at UNC.

Any Aes, SAEs or unanticipated problems will be followed until resolution by the Pis and/or research staff. Participants will be contacted regularly during follow-up and any observations of Aes/SAEs will be discussed among the Pis and the study physician at regular meetings (or immediately, in the event of SAEs or unanticipated problems). In the event of SAEs or unanticipated problems, the physician and

study Pis will be notified immediately, and the participant will be referred to or provided with appropriate medical support.

Participants will be advised to seek prompt medical attention in the event of an unanticipated medical event. In the event of an SAE, a member of the study medical staff will maintain regular contact with the participant until the event is resolved and fully documented. A determination of relatedness to medication will be made based on symptoms, timing, and other factors.

Monitoring will be the primary responsibility of the PI. In addition, a Data and Safety Monitoring Board will be established to maximize safety (the TraCS DSMB will be involved for this project, and will review progress at scheduled semiannual meetings during the trial).

8.3.7 REPORTING EVENTS TO PARTICIPANTS

If an event is deemed an AE or SAE, participants will receive a phone call from study personnel to inform them that the event has been recorded and will be reported as such. Any incidental findings uncovered during the baseline medical exam or weekly visits (e.g., via bloodwork or other measurements) will be reported to the participant by the study physician, and an appropriate follow-up plan will be recommended to the participant.

8.3.8 EVENTS OF SPECIAL INTEREST

Not applicable

8.3.9 REPORTING OF PREGNANCY

At the baseline visit and each study visit that involves an alcohol session, female subjects will undergo a urine pregnancy test upon arrival to the clinical site. In the case of a positive pregnancy test, the participant will be notified immediately, and study intervention will be discontinued while continuing safety follow-up. If any follow-up plan is recommended by the study physician, one of the staff members will communicate this recommendation to the participant.

8.4 UNANTICIPATED PROBLEMS

8.4.1 DEFINITION OF UNANTICIPATED PROBLEMS (UP)

The Office for Human Research Protections (OHRP) considers unanticipated problems involving risks to participants or others to include, in general, any incident, experience, or outcome that meets **all** of the following criteria:

- Unexpected in terms of nature, severity, or frequency given (a) the research procedures that are described in the protocol-related documents, such as the Institutional Review Board (IRB)-approved research protocol and informed consent document; and (b) the characteristics of the participant population being studied;

- Related or possibly related to participation in the research ("possibly related" means there is a reasonable possibility that the incident, experience, or outcome may have been caused by the procedures involved in the research); and
- Suggests that the research places participants or others at a greater risk of harm (including physical, psychological, economic, or social harm) than was previously known or recognized.

Any Ups identified by the study team will be discussed amongst the study leadership team, and corrective actions will be identified. If applicable, a protocol amendment will be developed and submitted to the IRB.

8.4.2 UNANTICIPATED PROBLEM REPORTING

The investigator will report unanticipated problems (Ups) to the reviewing Institutional Review Board (IRB), and if applicable, the DSMB. The UP report will include the following information:

- Protocol identifying information: protocol title and number, PI's name, and the IRB project number;
- A detailed description of the event, incident, experience, or outcome;
- An explanation of the basis for determining that the event, incident, experience, or outcome represents an UP;
- A description of any changes to the protocol or other corrective actions that have been taken or are proposed in response to the UP.

8.4.3 REPORTING UNANTICIPATED PROBLEMS TO PARTICIPANTS

In the case of a UP that may impact participants' safety or alter their experience during the trial (e.g., by necessitating a change in protocol), participants will be notified by a study team member at the subsequent visit. In the event of any UP that may have an imminent impact on participant safety, a team member will contact the participant by phone to inform them of the problem.

9 STATISTICAL CONSIDERATIONS

9.1 STATISTICAL HYPOTHESES

- Primary Efficacy Endpoint(s):
    - Breath alcohol concentration (BrAC) and volume of alcohol consumed during laboratory alcohol self-administration procedures. The outcomes are continuous variables. The effect of interest is the interaction between medication group (semaglutide vs. placebo) and time/dosage (baseline vs. 0.5mg). The baseline session will take place between Weeks 0 and 1, and the 0.5mg session will take place between weeks 8 and 9.
- Secondary Efficacy Endpoint(s):
    - Changes in subjective responses to alcohol (stimulation, sedation, craving) and alcohol and cigarette demand during laboratory alcohol challenge procedures. The outcomes will be

analyzed as continuous variables. The effect of interest is the interaction between medication group (semaglutide vs. placebo) and time/dosage (baseline vs. 0.5mg). The baseline session will take place between Weeks 0 and 1, and the 0.5mg session will take place between weeks 8 and 9.

- Changes in drinks per day and cigarette consumption over the course of the trial, as assessed via daily surveys. The outcome variables will be treated as continuous, however, this assumption will be tested and the modeling approach will revised as needed to properly account for the nature of the outcome variable (e.g., analyze as count variable with negative binomial distribution). The effect of interest is the interaction between medication group (semaglutide vs. placebo) and time (Week 1 to Week 10).

9.2 SAMPLE SIZE DETERMINATION

Because no human data on semaglutide's effects on alcohol consumption outcomes are available, there is no a priori effect size estimate to facilitate power calculations. We will seek a final sample size of 36 participants. Assuming at least 36 enrollees have complete data, our analyses suggest that this allows sufficient power (.80) to detect a medium effect size (f = .25) for an interaction between group (medication vs. placebo) and dosage (baseline, 0.25mg, 0.5mg). This estimate assumes an alpha level of .05 and a within-person correlation ® of 0.5 across time points. Further, because our multilevel model (MLM) analyses will involve more time points for certain analyses (e.g., daily assessments), this approach will increase power further, which is advantageous in the event that medication effects are in the small range. Assuming a 25% attrition rate, we anticipate needing to randomize 48 enrollees to arrive at a final sample of 36.

9.3 POPULATIONS FOR ANALYSES

Primary analyses will include all participants who were randomized and received a medication or placebo dose following the Week 0 visit (randomization will occur after the Week 0 visit, just prior to the Week 1 visit). MLM analyses can accommodate missing data, allowing inclusion of participants without complete data (e.g., missed visits or missed daily survey responses). Reasons for missing data and drop-out will be documented in the study database and reported in any publications.

A supplementary per-protocol analyses will be conducted using all participants who received 1) at least 7 of the 9 scheduled doses, and 2) those participants who receive the final (1.0mg) dose. This planned analysis will be conducted irrespective of the number of participants in these groups. If the sample permits, an exploratory subgroup analysis will examine participants as a function of weight (overweight/obese vs. not overweight or obese).

9.4 STATISTICAL ANALYSES

9.4.1 GENERAL APPROACH

Primary analyses will rely on multilevel models (MLM), which are well suited to examining within-person changes over time, while also accommodating missing data and unequal group sizes, and allowing the evaluation of between-subjects moderators (e.g., overweight/obesity status) if necessary. MLM with

maximum likelihood estimation can accommodate missing data under the assumption that the data are missing at random. Secondary, sensitivity analyses will be conducted to test the robustness of study findings for violations of the assumption that data is missing at random. Specifically, we will examine if baseline variables are associated with data missingness (for instance, age, sex, level of substance use) using t-tests and chi-square analyses (for continuous and dichotomous variables, respectively). Baseline variables that are associated with data missingness will be included as covariates in additional adjusted models (that adjust for the baseline variables related to missingness). Assumptions for the statistical methods being used will be tested. Should there be evidence of violations of these assumptions (e.g., presence of outlying and influential cases, distributional concerns for residuals, and so on), appropriate corrective actions will be taken. Sensitivity analyses will be used to determine the extent to which the main study results are influenced by these violations. We will focus our interpretations on small p-values ($p < .05$) that indicate evidence against the null hypothesis. We will report the number of decimal places of the p-value in line with the journal's guidelines and APA recommendations (usually 2 or 3 decimal places). For specific outcomes, the units of measurement and expected ranges of values (or example ranges, if the full range is unknown) are noted in the subsequent analysis sections 9.4.2 and 9.4.3. Any extreme outliers (defined as >3.0 standard deviations from the mean) will be recoded to one unit greater than the next most extreme value, in order to reduce undue influence of extreme outliers. For all statistical tests, confidence intervals will be evaluated in combination with tests of statistical significance. Assumptions for the statistical methods being used will be tested. Should there be evidence of violations of these assumptions (e.g., presence of outlying and influential cases, distributional concerns for residuals, and so on), appropriate corrective actions will be taken. Sensitivity analyses will be used to determine the extent to which the main study results are influenced by these violations.

9.4.2 ANALYSIS OF THE PRIMARY EFFICACY ENDPOINT(S)

Alcohol self-administration sessions: MLM will be used to examine changes in laboratory self-administration (defined as peak BAC and quantity of alcohol consumed, co-primary outcomes) as a function of medication phase (baseline; 0.5mg). These analyses will use medication phase as the within-subjects factor and condition (semaglutide vs. placebo) as the between-person factor. Self-administration will be measured based on recorded breath alcohol concentration (BrAC), measured in g% (e.g., .00g% - .08g%).

9.4.3 ANALYSIS OF THE SECONDARY ENDPOINT(S)

Alcohol challenge sessions: MLM will also be used to examine changes in 1) cigarette and alcohol demand during alcohol challenge (based on corresponding purchase tasks) (e.g., based on number of hypothetical drinks/cigarettes consumed at a given price point), and 2) self-reported stimulation (0-10 range), sedation (0-10 range), and craving (1-7 range), from BAC = .00 to BAC = .06g%, as a function of medication phase (within-person) and drug condition (between person). These analyses will examine changes in outcomes from BAC = .00 to .06 using medication phase as the within-subjects factor and condition as the between-person factor. The analyses will be complemented by analyses of peak subjective effects (e.g., stimulation, sedation, craving, on the same scales as noted above), across the two alcohol administration sessions (AC1 and AC2).

Daily self-report data: We will use MLM to estimate within-person changes in primary outcomes (e.g., daily alcohol use [number of drinks consumed per day] and cigarette use [number of cigarettes smoked per day] over the trial period), using study week as the within-subjects factor and condition (semaglutide, placebo) as the between-person factor.

9.4.4 SAFETY ANALYSES

Total side effects reported (e.g., 0-15), and number of severe side effects reported during each medication phase will be assessed using MLM with medication phase (baseline, 0.25mg, 0.5mg, 1.0mg) as the within-subject factor and condition (semaglutide, placebo) as the between-person factor. . These count variables will be analyzed using a negative binomial MLM, however, this assumption will be tested and the modeling approach will revised as needed to properly account for the nature of the outcome variable (e.g., zero-inflated negative binomial regression).

9.4.5 BASELINE DESCRIPTIVE STATISTICS

Descriptive statistics will be used to report relevant demographics such as age, sex, and education.

9.4.6 PLANNED INTERIM ANALYSES

No interim analyses of efficacy will be planned.

9.4.7 SUB-GROUP ANALYSES

Not applicable

9.4.8 TABULATION OF INDIVIDUAL PARTICIPANT DATA

Not applicable

9.4.9 EXPLORATORY ANALYSES

Sex will be examined as an exploratory moderator in all analyses.

10 SUPPORTING DOCUMENTATION AND OPERATIONAL CONSIDERATIONS

10.1 REGULATORY, ETHICAL, AND STUDY OVERSIGHT CONSIDERATIONS

10.1.1 INFORMED CONSENT PROCESS

In obtaining and documenting informed consent, the team will comply with applicable regulatory requirements (e.g., 45 CFR Part 46, 21 CFR Part 50, 21 CFR Part 56) and adhere to ICH GCP. Consent will be obtained remotely (via web teleconference and Docusign, as described previously) by a qualified study staff member in person at the outset of the baseline session. Additional procedures are noted below.

10.1.1.1 CONSENT/ASSENT AND OTHER INFORMATIONAL DOCUMENTS PROVIDED TO PARTICIPANTS

Consent forms will describe the study intervention, study procedures, and potential risks. The participant's electronic signature indicating consent is required prior to starting intervention/administering study intervention. Participants will also be given an informational sheet about the study medication. Additional procedures are described below.

10.1.1.2 CONSENT PROCEDURES AND DOCUMENTATION

Informed consent is a process that is initiated prior to the individual's agreeing to participate in the study and continues throughout the individual's study participation. Consent forms will be Institutional Review Board (IRB)-approved and the participant will be asked to read and review the document. The initial consent process will be conducted remotely using a HIPAA-compliant web platform (e.g., WebEx, Zoom, Teams) and Part 11-compliant Docusign to maintain optimal safety. A copy of the consent form will be provided to the participant in advance of the scheduled videoconference. A qualified team member will explain the research study to the participant and answer any questions that may arise. A verbal explanation will be provided in terms suited to the participant's comprehension of the purposes, procedures, and potential risks of the study and of their rights as research participants. Participants will have the opportunity to carefully review the consent form and ask questions prior to signing. The participants should have the opportunity to discuss the study with their family or surrogates or think about it prior to agreeing to participate. The participant will electronically sign the informed consent document prior to any procedures being done specifically for the study. Participants must be informed that participation is voluntary and that they may withdraw from the study at any time, without prejudice. A copy of the signed informed consent document will be given to the participants for their records. The informed consent process will be conducted and documented in the source document (including the date), and the form signed (via Docusign), before the participant undergoes any study-specific procedures. The rights and welfare of the participants will be protected by emphasizing to them that the study is voluntary and the quality of their medical care will not be adversely affected if they decline to participate. While the initial consent form will be conducted electronically, supplementary consent forms (e.g., consent to unencrypted communication, HIPAA authorization, and biospecimen consent) may be conducted either remotely (via Docusign) in advance of the baseline visit or in person (paper form) at the baseline visit, depending on timing.

10.1.2 STUDY DISCONTINUATION AND CLOSURE

This study may be temporarily suspended or prematurely terminated if there is sufficient reasonable cause. Written notification, documenting the reason for study suspension or termination, will be provided by the suspending or terminating party to study participants, investigator, funding agency, and regulatory authorities. If the study is prematurely terminated or suspended, the Principal Investigator (PI) will promptly inform study participants, the Institutional Review Board (IRB), and sponsor and will provide the reason(s) for the termination or suspension. Study participants will be contacted, as applicable, and be informed of changes to study visit schedule. Circumstances that may warrant termination or suspension include, but are not limited to determination of unexpected, significant, or unacceptable risk to participants. Study may resume once concerns about safety, protocol compliance, and data quality are addressed, and satisfy the sponsor, IRB and/or Food and Drug Administration (FDA).

10.1.3 CONFIDENTIALITY AND PRIVACY

Participant confidentiality and privacy is strictly held in trust by the participating investigators, their staff, and the sponsor(s) and their interventions. This confidentiality is extended to cover testing of biological samples and genetic tests in addition to the clinical information relating to participants. Therefore, the study protocol, documentation, data, and all other information generated will be held in strict confidence. No information concerning the study or the data will be released to any unauthorized third party. All research activities will be conducted in as private a setting as possible.

Any computerized data will be password protected and contained on a secure, password-protected computer or a secure server. Recruitment data (de-identified) will be stored in a secure, password-protected file accessible only to the study investigators and personnel. At the end of recruitment, eligible subject files will be separated from those failing to meet eligibility criteria, and records of ineligible recruitment subjects will be anonymized. Data pertaining to screening failures will be tabulated in an anonymized fashion. Data collected in other formats (e.g., medical screening notes) will be labeled only by study identifier and stored in binders in secure, locked cabinets while the study is taking place. Files will be stored and archived in accordance with Good Clinical Practice (GCP) standards. For all questionnaire and interview assessments, participants will be informed that they may decline to answer any question if they so choose.

The study monitor, other authorized representatives of the sponsor, representatives of the Institutional Review Board (IRB), regulatory agencies or pharmaceutical company supplying study product may inspect all documents and records required to be maintained by the investigator, including but not limited to, medical records (office, clinic, or hospital) and pharmacy records for the participants in this study. The clinical study site will permit access to such records.

The study participant’s contact information will be securely stored during the study for purposes of maintain contact with participants, and for communicating any safety information or unanticipated problems. At the end of the study, all records will continue to be kept in a secure location for as long a period as dictated by the reviewing IRB, Institutional policies, or sponsor requirements.

Study participant research data, which is for purposes of statistical analysis and scientific reporting, will be stored at UNC in the PI’s laboratory and on secure backup servers with password-protected access. This will not include the participant’s contact or identifying information. Rather, individual participants and their research data will be identified by a unique study identification number. Electronic data are stored on a password protected drive only accessible by the research team. Data obtained on paper will be transferred to an electronic data file (Excel, SPSS, REDCap), stored on a secure server, and identified by participants ID. For non-computer based forms, the data collection sheets are stored in a locked cabinet in a separate locked data storage space at the PI’s laboratory.

At the end of the study, all study databases will be de-identified and archived in the PI’s laboratory and backed up on a secure server hosted at UNC. De-identified data resulting from this study may also be presented at meetings, published in journals or presentations. In addition, this study will be registered at ClinicalTrials.gov, and de-identified information from this study will be submitted to ClinicalTrials.gov.

10.1.4 FUTURE USE OF STORED SPECIMENS AND DATA

Data collected for this study will be analyzed and stored at the PI's laboratory. The study biostatistician will be given access to the data necessary for conducting specified analyses, and data will be de-identified prior to giving access.

De-identified biological samples will be stored at UNC for the duration of the study, until all biological tests are completed and the data transferred to electronic format. De-identified DNA samples will be stored indefinitely, provided the participant has given consent for DNA storage, in order to examine future questions related to phenotypes of interest for this line of research.

During the conduct of the study, an individual participant can choose to withdraw consent to have biological specimens stored for future research. However, withdrawal of consent with regard to biosample storage may not be possible after the study is completed (after specimens are de-identified).

10.1.5 KEY ROLES AND STUDY GOVERNANCE

Provide the name and contact information of the Principal Investigator and the Medical Monitor.

**Principal Investigator**	**Medical Monitor**
Christian Hendershot, Ph.D., Associate Professor	*TBD (The medical monitor and DSMB members will be identified and added prior to data collection)*
UNC-Chapel Hill	
104 Manning Dr.	
Christian_Hendershot@med.unc."	
Christian_Hendershot@med.unc.edu	

The investigative team is summarized on the face page of this protocol. As noted, a DSMB will be formed prior to carrying out the study.

Safety oversight will be under the direction of a Data and Safety Monitoring Board (DSMB). Members of the DSMB will be independent from the study conduct and free of conflict of interest. The DSMB will dictate the intervals at which they meet to assess safety and efficacy data. At this time, each data element that the DSMB needs to assess will be clearly defined. The DSMB will provide its input to the IRB and National Institutes of Health as necessary.

10.1.6 CLINICAL MONITORING

Clinical site monitoring is conducted to ensure that the rights and well-being of trial participants are protected, that the reported trial data are accurate, complete, and verifiable, and that the conduct of the trial is in compliance with the currently approved protocol/amendment(s), with International Conference on Harmonisation Good Clinical Practice (ICH GCP), and with applicable regulatory requirement(s). A local study monitor will be appointed to provide on-site monitoring during the course of the research study.

10.1.7 QUALITY ASSURANCE AND QUALITY CONTROL

The PI will be responsible for addressing quality control issues and correcting errors or protocol deviations. The PI will have oversight over training study staff and ensuring that protocol elements are carried out with fidelity. Quality control will be ensured by developing SOPs for protocol elements to standardize data collection procedures. The investigational site will provide direct access to all trial related sites, source data/documents, and reports for the purpose of monitoring and auditing by the sponsor, and inspection by local and regulatory authorities.

10.1.8 DATA HANDLING AND RECORD KEEPING

10.1.8.1 DATA COLLECTION AND MANAGEMENT RESPONSIBILITIES

As a single-site, small-scale trial, the project will not involve coordinated data collection and management responsibilities across sites/teams. Data safety and storage precautions are outlined elsewhere in the study protocol.

10.1.8.2 STUDY RECORDS RETENTION

Study records will be retained for a minimum of 7 years following study completion. Paper source documents will be archived in accordance with UNC policies. Electronic materials and de-identified datasets will be stored securely as outlined above.

10.1.9 PROTOCOL DEVIATIONS

A protocol deviation is any noncompliance with the clinical trial protocol, International Conference on Harmonisation Good Clinical Practice (ICH GCP), or Manual of Procedures (MOP) requirements. The PI will report deviations within 7 working days of identification of the protocol deviation, or within 7 working days of the scheduled protocol-required activity. All deviations must be addressed in study source documents, reported to NIAAA Program Official and the IRB. Protocol deviations must be sent to the reviewing Institutional Review Board (IRB) per their policies. The site investigator is responsible for knowing and adhering to the reviewing IRB requirements. Further details about the handling of protocol deviations will be included in the MOP.

10.1.10 PUBLICATION AND DATA SHARING POLICY

This study will be conducted in accordance with the following publication and data sharing policies and regulations:

National Institutes of Health (NIH) Public Access Policy, which ensures that the public has access to the published results of NIH funded research. It requires scientists to submit final peer-reviewed journal manuscripts that arise from NIH funds to the digital archive PubMed Central upon acceptance for publication.

This study will comply with the NIH Data Sharing Policy and Policy on the Dissemination of NIH-Funded Clinical Trial Information and the Clinical Trials Registration and Results Information Submission rule. As such, this trial will be registered at ClinicalTrials.gov, and results information from this trial will be submitted to ClinicalTrials.gov. In addition, every attempt will be made to publish results in peer-reviewed journals. Data from this study may be requested from other researchers after the completion of the primary endpoint by contacting the PI.

10.1.11 CONFLICT OF INTEREST POLICY

Any actual conflict of interest of persons who have a role in the design, conduct, analysis, publication, or any aspect of this trial will be disclosed and managed. Furthermore, persons who have a perceived conflict of interest will be required to have such conflicts managed in a way that is appropriate to their participation in the design and conduct of this trial. All investigators will make a conflict of interest declaration at the outset of the study and at regular intervals thereafter in accordance with UNC policies.

10.2 ADDITIONAL CONSIDERATIONS

Not applicable

10.3 ABBREVIATIONS

AASE	Alcohol Abstinence Self-Efficacy
AE	Adverse Event
APT	Alcohol Purchase Task
AUD	Alcohol Use Disorder
AUDIT	Alcohol Use Disorder Identification Test
AUQ	Alcohol Urge Questionnaire
BAES	Biphasic Alcohol Effects Scale
BIS-11	Barratt Impulsivity Scale
BMI	Body Mass Index
BrAC	Breath Alcohol Concentration
CASE	Cigarette Abstinence Self-Efficacy
CES-D	Center for Epidemiological Studies Depression Scale
CEQ	Control of Eating Questionnaire
CFR	Code of Federal Regulations
CMP	Clinical Monitoring Plan
CO	Carbon Monoxide
CPT	Cigarette Purchase Task
CRF	Case Report Form
CTRC	Clinical & Translational Research Center
DEQ	Drug Effects Questionnaire
DNA	Deoxyribonucleic acid
DSM-5	Diagnostic and Statistical Manual for Mental Disorders v.5

DSMB	Data Safety Monitoring Board
FDA	Food and Drug Administration
FPT	Food Purchase Task
FTND	Fagerstrom test for Nicotine Dependence
GCP	Good Clinical Practice
GLP-1	Glucagon-Like Peptide 1
GLP-1R	Glucagon-Like Peptide 1 Receptor
HbA1c	Glycated hemoglobin
ICH	International Conference on Harmonisation
ICS	Impaired Control Scale
IDS	Inventory of Drinking Situations
IRB	Institutional Review Board
MCQ	Monetary Choice Questionnaire
MNWS	Minnesota Nicotine Withdrawal Scale
MOP	Manual of Procedures
NC Tracs	North Carolina Translational & Clinical Sciences Institute
NDSS	Nicotine Dependence Syndrome Scale
NIAAA	National Institute on Alcohol Abuse and Alcoholism
NIH	National Institutes of Health
NRT	Nicotine Replacement Therapy
PACS	Penn Alcohol Craving Scale
PI	Principal Investigator
POMS-SF	Profile Of Mood States Short Form
PAWS	Prediction of Alcohol Withdrawal Severity Scale
QA	Quality Assurance
QC	Quality Control
RBEDS	Reward Based Eating Drive Scale
RPI-EROS	Reward Probability Index – Environmental Reward Observation Scale
SAE	Serious Adverse Event
SAFTEE	Systematic Assessment for Treatment Emergent Effects
SAWS	Short Alcohol Withdrawal Scale
SNAQ	Simplified Nutritional Appetite Questionnaire
SOA	Schedule of Activities
SOP	Standard Operating Procedure
SRE	Self-Report of the Effects of Alcohol Questionnaire
SUD	Substance Use Disorder
T2D	Type II Diabetes
TAS-20	Toronto Alexithymia Scale
TFEQ	Three Factor Eating Questionnaire
TLFB	Timeline Follow-Back Interview
TQSU	Tiffany Questionnaire of Smoking Urges
UP	Unanticipated Problem
UPPS-P	UPPS-P Impulsive Behavior Scale
WISDM	Wisconsin Inventory of Smoking Dependence Motives
WSWS	Wisconsin Smoking Withdrawal Scale
5-HT2C	Serotonin 2C Receptor

10.4 PROTOCOL AMENDMENT HISTORY

The table below is intended to capture changes of IRB-approved versions of the protocol, including a description of the change and rationale. A Summary of Changes table for the current amendment is located in the Protocol Title Page.

Version	Date	Description of Change	Brief Rationale
1.2	July 26 2021	Elaboration on statistical analyses and clarification on other procedural issues.	Minor changes requested during initial review of the protocol.
1.3	Feb 28 2021	Clarification of medication dispensing procedures, addition of personnel, addition of Docusign procedures, other protocol modifications.	Changes addressed issues that emerged while preparing for recruitment and meeting with IDS and study investigators.
1.4	June 23 2022	Minor revisions to select assessment instruments, exclusion criteria, and clarification of medication dosing and DSMB meeting plan	Refinement of study procedures and clarification of monitoring schedule as requested by DSMB and IDS
1.5	August 22 2022	Updates to medication storage, use of Phone.com for text messaging, select questionnaire modifications, modifications to alcohol self-administration session	Medication storage changes requested by IDS; Phone.com added as per UNC requirements for text messaging; other changes intended to streamline and improve assessment procedures
1.6	October 31 2022	Updated inclusion criteria to allow for light smokers and increased range of AUD symptoms; added analysis for blood neutrophils; added additional recruitment flyers.	Changes made to facilitate recruitment and address an exploratory research question.

11 REFERENCES

1. Koob, G.F., G. Kenneth Lloyd, and B.J. Mason, *Development of pharmacotherapies for drug addiction: a Rosetta stone approach.* Nat Rev Drug Discov, 2009. **8**(6): p. 500-15.
2. Litten, R.Z., et al., *Medications development to treat alcohol dependence: a vision for the next decade.* Addiction biology, 2012. **17**(3): p. 513-27.
3. Litten, R.Z., et al., *Discovery, Development, and Adoption of Medications to Treat Alcohol Use Disorder: Goals for the Phases of Medications Development.* Alcohol Clin Exp Res, 2016. **40**(7): p. 1368-79.
4. Litten, R.Z., et al., *Potential medications for the treatment of alcohol use disorder: An evaluation of clinical efficacy and safety.* Subst Abus, 2016. **37**(2): p. 286-98.
5. Litten, R.Z., et al., *Heterogeneity of alcohol use disorder: understanding mechanisms to advance personalized treatment.* Alcohol Clin Exp Res, 2015. **39**(4): p. 579-84.
6. Heilig, M., et al., *Pharmacogenetic approaches to the treatment of alcohol addiction.* Nature reviews. Neuroscience, 2011. **12**(11): p. 670-84.
7. Falk, D.E., H.Y. Yi, and S. Hiller-Sturmhofel, *An epidemiologic analysis of co-occurring alcohol and tobacco use and disorders: findings from the National Epidemiologic Survey on Alcohol and Related Conditions.* Alcohol Res Health, 2006. **29**(3): p. 162-71.
8. McKee, S.A. and A.H. Weinberger, *How can we use our knowlegde of alcohol-tobacco interactions to reduce alcohol use?* Annu Rev Clin Psychol, 2013. **9**: p. 649–674.
9. Organization, W.H., *Global Health Risks. Mortality and Burden of Disease Attributable to Selected Major Risks*. 2009, World Health Organization: Geneva, Switzerland.
10. Dawson, D.A., *Drinking as a risk factor for sustained smoking.* Drug Alcohol Depend, 2000. **59**(3): p. 235-49.
11. Yardley, M.M., M.M. Mirbaba, and L.A. Ray, *Pharmacological Options for Smoking Cessation in Heavy-Drinking Smokers.* CNS Drugs, 2015. **29**(10): p. 833-45.
12. Prabhu, A., K.O. Obi, and J.H. Rubenstein, *The synergistic effects of alcohol and tobacco consumption on the risk of esophageal squamous cell carcinoma: a meta-analysis.* Am J Gastroenterol, 2014. **109**(6): p. 822-7.
13. Pelucchi, C., et al., *Cancer risk associated with alcohol and tobacco use: focus on upper aero-digestive tract and liver.* Alcohol Res Health, 2006. **29**(3): p. 193-8.
14. Kahler, C.W., N.S. Spillane, and J. Metrik, *Alcohol use and initial smoking lapses among heavy drinkers in smoking cessation treatment.* Nicotine Tob Res, 2010. **12**(7): p. 781-5.
15. Roche, D.J., et al., *Current insights into the mechanisms and development of treatments for heavy drinking cigarette smokers.* Curr Addict Rep, 2016. **3**(1): p. 125-137.
16. McKee, S.A., et al., *Varenicline reduces alcohol self-administration in heavy-drinking smokers.* Biol Psychiatry, 2009. **66**(2): p. 185-90.
17. King, A., et al., *Naltrexone decreases heavy drinking rates in smoking cessation treatment: an exploratory study.* Alcohol Clin Exp Res, 2009. **33**(6): p. 1044-50.
18. O'Malley, S.S., et al., *Dose-dependent reduction of hazardous alcohol use in a placebo-controlled trial of naltrexone for smoking cessation.* Int J Neuropsychopharmacol, 2009. **12**(5): p. 589-97.
19. King, A., et al., *Effects of the opioid receptor antagonist naltrexone on smoking and related behaviors in smokers preparing to quit: a randomized controlled trial.* Addiction, 2013. **108**(10): p. 1836-44.
20. Anton, R.F., et al., *Nicotine-Use/Smoking Is Associated with the Efficacy of Naltrexone in the Treatment of Alcohol Dependence.* Alcohol Clin Exp Res, 2018. **42**(4): p. 751-760.
21. Fucito, L.M., et al., *Cigarette smoking predicts differential benefit from naltrexone for alcohol dependence.* Biol Psychiatry, 2012. **72**(10): p. 832-8.
22. Hartmann-Boyce, J., et al., *Efficacy of interventions to combat tobacco addiction: Cochrane update of 2013 reviews.* Addiction, 2014. **109**(9): p. 1414-25.
23. Kahler, C.W., et al., *A Double-Blind Randomized Placebo-Controlled Trial of Oral Naltrexone for Heavy-Drinking Smokers Seeking Smoking Cessation Treatment.* Alcohol Clin Exp Res, 2017. **41**(6): p. 1201-1211.

24. Mitchell, J.M., et al., *Varenicline decreases alcohol consumption in heavy-drinking smokers.* Psychopharmacology (Berl), 2012. **223**(3): p. 299-306.
25. Fucito, L.M., et al., *A preliminary investigation of varenicline for heavy drinking smokers.* Psychopharmacology (Berl), 2011. **215**(4): p. 655-63.
26. Litten, R.Z., et al., *A double-blind, placebo-controlled trial assessing the efficacy of varenicline tartrate for alcohol dependence.* J Addict Med, 2013. **7**(4): p. 277-86.
27. Roche, D.J., et al., *Combined varenicline and naltrexone treatment reduces smoking topography intensity in heavy-drinking smokers.* Pharmacol Biochem Behav, 2015. **134**: p. 92-8.
28. Ray, L.A., et al., *Varenicline, low dose naltrexone, and their combination for heavy-drinking smokers: human laboratory findings.* Psychopharmacology (Berl), 2014. **231**(19): p. 3843-53.
29. Andersen, A., et al., *Glucagon-like peptide 1 in health and disease.* Nature Reviews Endocrinology, 2018. **14**(7): p. 390-403.
30. Jerlhag, E., *Alcohol-mediated behaviours and the gut-brain axis; with focus on glucagon-like peptide-1.* Brain Res, 2020. **1727**: p. 146562.
31. Brunchmann, A., M. Thomsen, and A. Fink-Jensen, *The effect of glucagon-like peptide-1 (GLP-1) receptor agonists on substance use disorder (SUD)-related behavioural effects of drugs and alcohol: A systematic review.* Physiol Behav, 2019. **206**: p. 232-242.
32. Egecioglu, E., J.A. Engel, and E. Jerlhag, *The glucagon-like peptide 1 analogue Exendin-4 attenuates the nicotine-induced locomotor stimulation, accumbal dopamine release, conditioned place preference as well as the expression of locomotor sensitization in mice.* PLoS One, 2013. **8**(10): p. e77284.
33. Anderberg, R.H., et al., *Glucagon-Like Peptide 1 and Its Analogs Act in the Dorsal Raphe and Modulate Central Serotonin to Reduce Appetite and Body Weight.* Diabetes, 2017. **66**(4): p. 1062-1073.
34. Howell, E., et al., *Glucagon-Like Peptide-1 (GLP-1) and 5-Hydroxytryptamine 2c (5-HT(2c)) Receptor Agonists in the Ventral Tegmental Area (VTA) Inhibit Ghrelin-Stimulated Appetitive Reward.* Int J Mol Sci, 2019. **20**(4).
35. Asarian, L., *Loss of cholecystokinin and glucagon-like peptide-1-induced satiation in mice lacking serotonin 2C receptors.* American Journal of Physiology-Regulatory, Integrative and Comparitive Physiology, 2009. **296**(1): p. R51-R56.
36. Nonogaki, K., et al., *The contribution of serotonin 5-HT2C and melanocortin-4 receptors to the satiety signaling of glucagon-like peptide 1 and liraglutide, a glucagon-like peptide 1 receptor agonist, in mice.* Biochem Biophys Res Commun, 2011. **411**(2): p. 445-8.
37. Doggrell, S.A., *Sgemaglutide in type 2 diabetes - is it the best glucagon-like peptide 1 receptor agonist (GLP-1R agonist)?* Expert Opin Drug Metab Toxicol, 2018. **14**(3): p. 371-377.
38. Litten, R.Z., et al., *Development of medications for alcohol use disorders: recent advances and ongoing challenges.* Expert Opin Emerg Drugs, 2005. **10**(2): p. 323-43.
39. Bujarski, S. and L.A. Ray, *Experimental psychopathology paradigms for alcohol use disorders: Applications for translational research.* Behav Res Ther, 2016.
40. Hendershot, C.S., et al., *Effects of naltrexone on alcohol self-administration and craving: meta-analysis of human laboratory studies.* Addict Biol, 2016.
41. McKee, S.A., *Developing human laboratory models of smoking lapse behavior for medication screening.* Addict Biol, 2009. **14**(1): p. 99-107.
42. Plebani, J.G., et al., *Human laboratory paradigms in alcohol research.* Alcohol Clin Exp Res, 2012. **36**(6): p. 972-83.
43. Ray, L.A., K.E. Hutchison, and M. Tartter, *Application of human laboratory models to pharmacotherapy development for alcohol dependence.* Curr Pharm Des, 2010. **16**(19): p. 2149-58.
44. Lerman, C., et al., *Translational research in medication development for nicotine dependence.* Nat Rev Drug Discov, 2007. **6**(9): p. 746-62.
45. MacKillop, J. and J.G. Murphy, *A behavioral economic measure of demand for alcohol predicts brief intervention outcomes.* Drug Alcohol Depend, 2007. **89**(2-3): p. 227-33.
46. Amlung, M., et al., *Increased behavioral economic demand and craving for alcohol following a laboratory alcohol challenge.* Addiction, 2015. **110**(9): p. 1421-8.
47. Green, R. and L.A. Ray, *Effects of varenicline on subjective craving and relative reinforcing value of cigarettes.* Drug Alcohol Depend, 2018. **188**: p. 53-59.

48. Murphy, C.M., et al., *Effects of varenicline versus transdermal nicotine replacement therapy on cigarette demand on quit day in individuals with substance use disorders.* Psychopharmacology (Berl), 2017. **234**(16): p. 2443-2452.
49. Roache, J.D., *Role of the Human Laboratory in the Development of Medications for Alcohol and Drug Dependence*, in *Addiction Medicine*, B.A. Johnson, Editor. 2010, Springer: New York. p. 129-157.
50. Enoch, M.A., et al., *Ethical considerations for administering alcohol or alcohol cues to treatment-seeking alcoholics in a research setting: can the benefits to society outweigh the risks to the individual? A commentary in the context of the National Advisory Council on Alcohol Abuse and Alcoholism -- Recommended Council Guidelines on Ethyl Alcohol Administration in Human Experimentation (2005).* Alcohol Clin Exp Res, 2009. **33**(9): p. 1508-12.
51. Motschman, C.A., et al., *Selection criteria limit generalizability of smoking pharmacotherapy studies differentially across clinical trials and laboratory studies: A systematic review on varenicline.* Drug Alcohol Depend, 2016. **169**: p. 180-189.
52. Alcoholism, N.I.o.A.A.a., *Recommended council guidelines on ethyl alcohol administration in human experimentation*. 2005, Department of Health and Human Services: Rockville, MD.
53. First MB, W.J., Karg RS, Spitzer RL, *Structured Clinical Interview for DSM-5—Research Version (SCID-5 for DSM-5, Research Version; SCID-5-RV)*. 2015, Arlington, VA: American Psychiatric Association.
54. Sobell, L.C., et al., *Reliability of a timeline method: assessing normal drinkers' reports of recent drinking and a comparative evaluation across several populations.* Br J Addict, 1988. **83**(4): p. 393-402.
55. Saunders, J.B., et al., *Development of the Alcohol Use Disorders Identification Test (AUDIT): WHO Collaborative Project on Early Detection of Persons with Harmful Alcohol Consumption--II.* Addiction, 1993. **88**(6): p. 791-804.
56. Schuckit, M.A., et al., *The Self-Rating of the Effects of Alcohol Questionnaire as a Predictor of Alcohol-Related Outcomes in 12-Year-Old Subjects.* Alcohol and Alcoholism, 2008. **43**(6): p. 641-646.
57. Schuckit, M.A. and T.L. Smith, *The relationships of a family history of alcohol dependence, a low level of response to alcohol and six domains of life functioning to the development of alcohol use disorders.* Journal of Studies on Alcohol, 2000. **61**(6): p. 827-835.
58. Heatherton, T.F., et al., *The Fagerstrom Test for Nicotine Dependence: a revision of the Fagerstrom Tolerance Questionnaire.* Br J Addict, 1991. **86**(9): p. 1119-27.
59. Shiffman, S., A. Waters, and M. Hickcox, *The nicotine dependence syndrome scale: a multidimensional measure of nicotine dependence.* Nicotine Tob Res, 2004. **6**(2): p. 327-48.
60. Patton, J.H., M.S. Stanford, and E.S. Barratt, *Factor structure of the Barratt impulsiveness scale.* Journal of clinical psychology, 1995. **51**(6): p. 768-774.
61. Whiteside, S.P., et al., *Validation of the UPPS impulsive behaviour scale: a four-factor model of impulsivity.* European Journal of personality, 2005. **19**(7): p. 559-574.
62. Heather, N., et al., *Development of a scale for measuring impaired control over alcohol consumption: a preliminary report.* Journal of studies on alcohol, 1993. **54**(6): p. 700-709.
63. Radloff, L.S., *The CES-D scale: A self-report depression scale for research in the general population.* Applied psychological measurement, 1977. **1**(3): p. 385-401.
64. Leising, D., T. Grande, and R. Faber, *The Toronto Alexithymia Scale (TAS-20): A measure of general psychological distress.* Journal of research in personality, 2009. **43**(4): p. 707-710.
65. Carvalho, J.P., et al., *The Reward Probability Index: Design and validation of a scale measuring access to environmental reward.* Behavior therapy, 2011. **42**(2): p. 249-262.
66. Tiffany, S.T. and D.J. Drobes, *The development and initial validation of a questionnaire on smoking urges.* Br J Addict, 1991. **86**(11): p. 1467-76.
67. Welsch, S.K., et al., *Development and validation of the Wisconsin Smoking Withdrawal Scale.* Exp Clin Psychopharmacol, 1999. **7**(4): p. 354-61.
68. DiClemente, C.C., et al., *The Alcohol Abstinence Self-Efficacy scale.* J Stud Alcohol, 1994. **55**(2): p. 141-8.
69. Gwaltney, C.J., et al., *Does smoking abstinence self-efficacy vary across situations? Identifying context-specificity within the Relapse Situation Efficacy Questionnaire.* J Consult Clin Psychol, 2001. **69**(3): p. 516-27.

70. Mann, K., et al., *Precision Medicine in Alcohol Dependence: A Controlled Trial Testing Pharmacotherapy Response Among Reward and Relief Drinking Phenotypes.* Neuropsychopharmacology, 2018. **43**(4): p. 891-899.
71. Sties, S.W., et al., *Simplified Nutritional Appetite Questionnaire (SNAQ) for cardiopulmonary and metabolic rehabilitation program.* Revista Brasileira de Medicina do Esporte, 2012. **18**: p. 313-317.
72. Dalton, M., et al., *Preliminary validation and principal components analysis of the Control of Eating Questionnaire (CoEQ) for the experience of food craving.* European journal of clinical nutrition, 2015. **69**(12): p. 1313-1317.
73. Lesdema, A., et al., *Characterization of the Three-Factor Eating Questionnaire scores of a young French cohort.* Appetite, 2012. **59**(2): p. 385-90.
74. Epel, E.S., et al., *The reward-based eating drive scale: a self-report index of reward-based eating.* PLoS One, 2014. **9**(6): p. e101350.
75. Johnson, B.A., N. Ait-Daoud, and J.D. Roache, *The COMBINE SAFTEE: a structured instrument for collecting adverse events adapted for clinical studies in the alcoholism field.* J Stud Alcohol Suppl, 2005(15): p. 157-67; discussion 140.
76. Kaplan, B.A., et al., *Automating scoring of delay discounting for the 21-and 27-item monetary choice questionnaires.* The Behavior Analyst, 2016. **39**(2): p. 293-304.
77. MacKillop, J., et al., *Further validation of a cigarette purchase task for assessing the relative reinforcing efficacy of nicotine in college smokers.* Experimental and clinical psychopharmacology, 2008. **16**(1): p. 57.
78. Epstein, L.H., et al., *Reinforcing value and hypothetical behavioral economic demand for food and their relation to BMI.* Eating behaviors, 2018. **29**: p. 120-127.
79. MacKillop, J. and J.G. Murphy, *A behavioral economic measure of demand for alcohol predicts brief intervention outcomes.* Drug and alcohol dependence, 2007. **89**(2-3): p. 227-233.
80. Jensterle, M., et al., *Does intervention with GLP-1 receptor agonist semaglutide modulate perception of sweet taste in women with obesity: study protocol of a randomized, single-blinded, placebo-controlled clinical trial.* Trials, 2021. **22**(1): p. 1-12.
81. Kampov-Polevoy, A.B., et al., *Association between sweet preference and paternal history of alcoholism in psychiatric and substance abuse patients.* Alcoholism: Clinical and Experimental Research, 2003. **27**(12): p. 1929-1936.
82. Weafer, J., S.H. Mitchell, and H. de Wit, *Recent translational findings on impulsivity in relation to drug abuse.* Current addiction reports, 2014. **1**(4): p. 289-300.
83. Brick, J., *Standardization of alcohol calculations in research.* Alcohol Clin Exp Res, 2006. **30**(8): p. 1276-87.
84. Martin, C.S., et al., *DEVELOPMENT AND VALIDATION OF THE BIPHASIC ALCOHOL EFFECTS SCALE.* Alcoholism-Clinical and Experimental Research, 1993. **17**(1): p. 140-146.
85. Bohn, M.J., D.D. Krahn, and B.A. Staehler, *DEVELOPMENT AND INITIAL VALIDATION OF A MEASURE OF DRINKING URGES IN ABSTINENT ALCOHOLICS.* Alcoholism-Clinical and Experimental Research, 1995. **19**(3): p. 600-606.
86. Johanson, C.E. and E.H. Uhlenhuth, *Drug preference and mood in humans: diazepam.* Psychopharmacology, 1980. **71**(3): p. 269-73.
87. MacKillop, J., et al., *Further validation of a cigarette purchase task for assessing the relative reinforcing efficacy of nicotine in college smokers.* Exp Clin Psychopharmacol, 2008. **16**(1): p. 57-65.
88. Curran, S.L., M.A. Andrykowski, and J.L. Studts, *Short form of the profile of mood states (POMS-SF): psychometric information.* Psychological assessment, 1995. **7**(1): p. 80.

Human Laboratory Screening of Semaglutide for Alcohol Use Disorder

Protocol Number: 21-1689

National Clinical Trial (NCT) Identified Number: Pending

Investigators:

Dr. Christian Hendershot (Principal Investigator)
Dr. Robyn Jordan (Co-Investigator)
Dr. Amanda Tow (Co-Investigator)
Dr. Sarah Dermody (Co-Investigator, Biostatistician)
Dr. Eric Claus (Co-Investigator)
Dr. Klara Klein (Co-Investigator)
Dr. Paul Fletcher (Other Significant Contributor)

Funded by: NIAA

Version Number: v.1.6

31 Oct 2022

Summary of Changes from Previous Version:

Affected Section(s)	Summary of Revisions Made	Rationale
2.2, 5.1, 8.2, phone screening form	Updated inclusion criteria to include less-than daily regular smokers (i.e., at least once per week instead of daily), and to change the range of eligible alcohol use disorder symptoms from 2-5 to 2-8 (out of 11 possible symptoms).	We will continue recruiting regular smokers with alcohol use disorder, but have broadened our criteria. Recruitment numbers indicated that our criteria were overly narrow. Changes have been made in consultation with study physicians.
4.1, Stored Specimens consent	Added analysis for blood neutrophils with a corresponding increase of the estimated blood sample to 70-80ml (formerly 50-60ml).	Blood neutrophils are impacted by cigarette smoking; we will now investigate this as an ancillary aim for purposes of pilot data collection.

5.5	Added additional recruitment flyers/text and bus recruitment (Flyer 3, attached).	Expansion of recruitment efforts.

1 TABLE OF CONTENTS

STATEMENT OF COMPLIANCE......1
1 PROTOCOL SUMMARY......1
1.1 Synopsis......1
1.2 Schema......3
1.3 Schedule of Activities (SoA)......4
2 INTRODUCTION......5
2.1 Study Rationale......5
2.2 Background......6
2.3 Risk/Benefit Assessment......8
2.3.1 Known Potential Risks......8
2.3.2 Known Potential Benefits......10
2.3.3 Assessment of Potential Risks and Benefits......10
3 OBJECTIVES AND ENDPOINTS......11
4 STUDY DESIGN......13
4.1 Overall Design......13
4.2 Scientific Rationale for Study Design......22
4.3 Justification for Dose......22
4.4 End of Study Definition......23
5 STUDY POPULATION......23
5.1 Inclusion Criteria......23
5.2 Exclusion Criteria......23
5.3 Lifestyle Considerations......24
5.4 Screen Failures......25
5.5 Strategies for Recruitment and Retention......25
6 STUDY INTERVENTION......26
6.1 Study Intervention(s) Administration......26
6.1.1 Study Intervention Description......26
6.1.2 Dosing and Administration......27
6.2 Preparation/Handling/Storage/Accountability......28
6.2.1 Acquisition and accountability......28
6.2.2 Formulation, Appearance, Packaging, and Labeling......28

6.2.3 Product Storage and Stability 28
6.2.4 Preparation 29

6.3 Measures to Minimize Bias: Randomization and Blinding 29

6.4 Study Intervention Compliance 29

6.5 Concomitant Therapy 29

6.5.1 Rescue Medicine 30

7 STUDY INTERVENTION DISCONTINUATION AND PARTICIPANT DISCONTINUATION/WITHDRAWAL 30

7.1 Discontinuation of Study Intervention 30

7.2 Participant Discontinuation/Withdrawal from the Study 30

7.3 Lost to Follow-Up 31

8 STUDY ASSESSMENTS AND PROCEDURES 32

8.1 Efficacy Assessments 32

8.2 Safety and Other Assessments 32

8.3 Adverse Events and Serious Adverse Events 33

8.3.1 Definition of Adverse Events (AE) 33
8.3.2 Definition of Serious Adverse Events (SAE) 33
8.3.3 Classification of an Adverse Event 33
8.3.4 Time Period and Frequency for Event Assessment and Follow-Up 34
8.3.5 Adverse Event Reporting 35
8.3.6 Serious Adverse Event Reporting 35
8.3.7 Reporting Events to Participants 36
8.3.8 Events of Special Interest 36
8.3.9 Reporting of Pregnancy 36

8.4 Unanticipated Problems 36

8.4.1 Definition of Unanticipated Problems (UP) 36
8.4.2 Unanticipated Problem Reporting 37
8.4.3 Reporting Unanticipated Problems to Participants 37

9 STATISTICAL CONSIDERATIONS 37

9.1 Statistical Hypotheses 37

9.2 Sample Size Determination 38

9.3 Populations for Analyses 38

9.4 Statistical Analyses 38

9.4.1 General Approach 38
9.4.2 Analysis of the Primary Efficacy Endpoint(s) 39
9.4.3 Analysis of the Secondary Endpoint(s) 39
9.4.4 Safety Analyses 40

9.4.5 Baseline Descriptive Statistics 40
9.4.6 Planned Interim Analyses 40
9.4.7 Sub-Group Analyses 40
9.4.8 Tabulation of Individual participant Data 40
9.4.9 Exploratory Analyses 40
10 SUPPORTING DOCUMENTATION AND OPERATIONAL CONSIDERATIONS. 40
10.1 Regulatory, Ethical, and Study Oversight Considerations 40
10.1.1 Informed Consent Process 40
10.1.2 Study Discontinuation and Closure 41
10.1.3 Confidentiality and Privacy 42
10.1.4 Future Use of Stored Specimens and Data 42
10.1.5 Key Roles and Study Governance 43
10.1.6 Clinical Monitoring 43
10.1.7 Quality Assurance and Quality Control 44
10.1.8 Data Handling and Record Keeping 44
10.1.9 Protocol Deviations 44
10.1.10 Publication and Data Sharing Policy 44
10.1.11 Conflict of Interest Policy 45
10.2 Additional Considerations 45
10.3 Abbreviations 45
10.4 Protocol Amendment History 48
11 REFERENCES 50

STATEMENT OF COMPLIANCE

(1) The trial will be carried out in accordance with International Conference on Harmonization Good Clinical Practice (ICH GCP) and the following:

- United States (US) Code of Federal Regulations (CFR) applicable to clinical studies (45 CFR Part 46, 21 CFR Part 50, 21 CFR Part 56, 21 CFR Part 312, and/or 21 CFR Part 812)

National Institutes of Health (NIH)-funded investigators and clinical trial site staff who are responsible for the conduct, management, or oversight of NIH-funded clinical trials have completed Human Subjects Protection and ICH GCP Training.

The protocol, informed consent form(s), recruitment materials, and all participant materials will be submitted to the Institutional Review Board (IRB) for review and approval. Approval of both the protocol and the consent form must be obtained before any participant is enrolled. Any amendment to the protocol will require review and approval by the IRB before the changes are implemented to the study. In addition, all changes to the consent form will be IRB-approved; a determination will be made regarding whether a new consent needs to be obtained from participants who provided consent, using a previously approved consent form.

1 PROTOCOL SUMMARY

1.1 SYNOPSIS

Title: Human Laboratory Screening of Semaglutide for Alcohol Use Disorder

Study Description: This is an early-Phase II human laboratory trial using a randomized, placebo-controlled, dose-ranging design to investigate the effects of semaglutide (Ozempic®), a GLP-1 receptor agonist, on laboratory alcohol responses and consumption, naturalistic alcohol and cigarette consumption, and weight loss in smokers with alcohol use disorder (AUD). Participants will attend weekly visits while semaglutide dosage is increased to 1.0mg over a period of approximately 10 weeks. Participants will attend weekly visits for medication or placebo administration. At scheduled intervals, participants will complete 4 laboratory sessions involving alcohol self-administration and alcohol challenge to characterize medication effects on alcohol-related outcomes.

Objectives: Primary Objective: To examine effects of semaglutide vs. placebo on laboratory alcohol intake during a validated alcohol self-administration medication screening procedure.

Secondary Objectives: To examine medication vs. placebo group differences in 1) laboratory responses to alcohol, and 2) changes in naturalistic alcohol use and cigarette consumption over the course of the treatment period.

	Tertiary Objectives: To generate preliminary data on weight loss, HbA1c reductions and alcohol elimination among participants with AUD during treatment with a GLP-1 receptor agonist.
**Endpoints:**	Primary Endpoint: Change in amount of alcohol consumed during a validated laboratory alcohol self-administration protocol (primary endpoint). The specific effect of interest is the change in laboratory consumption from baseline to the maintenance dose (.5mg) as a function of medication condition (semaglutide vs. placebo) (i.e., treatment x time interaction).<br><br>Secondary Endpoints: Secondary endpoints will include laboratory measures of alcohol and cigarette demand (behavioral economic indices of drug motivation) and subjective responses to alcohol during an alcohol challenge procedure, and changes in naturalistic alcohol use and cigarette smoking over the treatment period. Within-person changes in these outcomes before vs. after treatment (baseline vs. 0.5mg) will be compared as a function of medication condition.
**Study Population:**	Participants who meet criteria for alcohol use disorder/AUD (N=48) and smoke cigarettes weekly will be enrolled. Participants will be recruited from the Triangle area (community and hospital settings). Participants must not be seeking current treatment for alcohol or smoking cessation.
**Phase:**	Phase II
**Description of Sites/Facilities Enrolling Participants:**	This is a single-site study (UNC-Chapel Hill). The project will be based at the Bowles Center for Alcohol studies and the Department of Psychiatry, with clinical operations taking place at Eastowne Clinical Research Unit and CTRC.
**Description of Study Intervention:**	Semaglutide (Ozempic®) will be administered according to the FDA-approved schedule, which begins with a starting dose of 0.25mg once per week for four weeks. At week five, dosage is increased to 0.5mg/week for a subsequent four weeks (maintenance dose). Following four weeks at the maintenance dose, dosage will be increased to 1.0mg provided the patient has tolerated the medication. Semaglutide is administered subcutaneously via the Ozempic® pen, a cartridge-based device that calibrates medication delivery. The placebo condition will consist of weekly sham doses, which will involve a brief needle insertion (without an injection).
**Study Duration:**	Up to 13 months.
**Participant Duration:**	Up to 40-50 hours total (including in-person visits and remote questionnaires) over a period of approximately 12 weeks.

1.2 SCHEMA

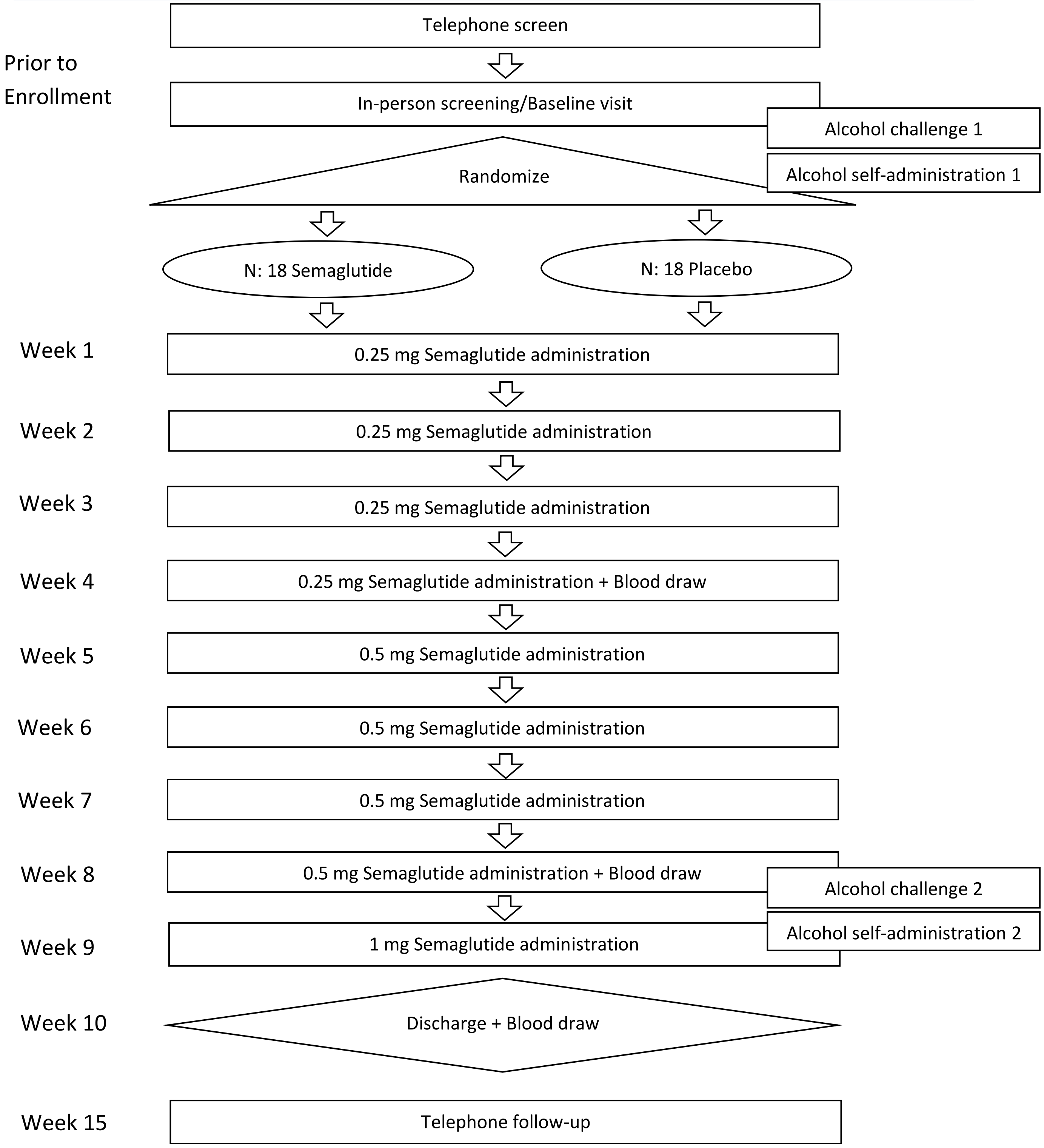
Prior to Enrollment
Telephone screen
In-person screening/Baseline visit
Alcohol challenge 1
Alcohol self-administration 1
Randomize
N: 18 Semaglutide
N: 18 Placebo
Week 1
0.25 mg Semaglutide administration
Week 2
0.25 mg Semaglutide administration
Week 3
0.25 mg Semaglutide administration
Week 4
0.25 mg Semaglutide administration + Blood draw
Week 5
0.5 mg Semaglutide administration
Week 6
0.5 mg Semaglutide administration
Week 7
0.5 mg Semaglutide administration
Week 8
0.5 mg Semaglutide administration + Blood draw
Alcohol challenge 2
Alcohol self-administration 2
Week 9
1 mg Semaglutide administration
Week 10
Discharge + Blood draw
Week 15
Telephone follow-up

1.3 SCHEDULE OF ACTIVITIES (SOA)

**Procedures and Questionnaires**	Telephone Screening	Screening/Baseline	Study Visit 1 – 3 Target Day 7, 14, 21	Study Visit 4 Target Day 28	Study Visit 5 – 7 Target Day 35, 42, 49	Study Visit 8 Target Day 56	Study Visit 9 Target Day 63	Discharge Target Day 70	Telephone follow-up Target Day 77, 105	Alcohol Challenge Target Day -10, 58	Alcohol Self-Admin. Target Day -12, 60
Telephone screening	X										
Administer 0.25 mg Semaglutide or placebo			X	X							
Administer 0.5 mg Semaglutide or placebo					X	X					
Administer 1.0 mg Semaglutide or placebo							X				
Breath Alcohol Concentration (BrAC) Test		X	X	X	X	X	X	X		X	X
Urine Drug Screen		X								X	X
Expired carbon monoxide (CO)		X	X	X	X	X	X	X		X	X
Locator		X									
Timeline Follow-Back (TLFB)		X	X	X	X	X	X	X			
BAC		X	X	X	X	X	X	X		X	X
Blood Draw		X		X		X		X			
Blood glucose – finger prick		X	X		X		X			X	X
Pregnancy (females only)		X	X	X	X	X	X	X		X	X
BMI		X	X	X	X	X	X	X		X	X
Height		X									
Vitals (HR, BP)		X	X	X	X	X	X	X		X	X
Weight		X	X	X	X	X	X	X		X	X
Temperature		X									
Demographics Form		X									
Structured Clinical Interview for DSM-5		X									
MINI Neuropsychiatric Interview		X									
Medical and Substance Use History Interviews		X									
Prediction of Alcohol Withdrawal Severity Scale (PAWS)		X									
Short Alcohol Withdrawal Scale (SAWS)		X								X	X
Saliva Collection for DNA and nicotine metabolite		X									
Case Report Forms (CRFs)	X	X	X	X	X	X	X	X	X	X	X
Alcohol Use Disorder Identification Test (AUDIT)		X									
Alcohol, Cigarette, and Food Purchase Test		X								X	X
Self-Report of the Effects of Alcohol Questionnaire (SRE)		X									
Fagerstrom Test for Nicotine Dependence (FTND)		X									
Nicotine Dependence Syndrome Scale (NDSS)		X									
Penn Alcohol Craving Scale (PACS)		X	X	X	X	X	X	X		X	X
Tiffany Questionnaire of Smoking Urges (TQSU)		X	X	X	X	X	X	X		X	X
Wisconsin Smoking Withdrawal Scale (WSWS)		X	X	X	X	X	X	X		X	X
Alcohol and Smoking Contemplation		X	X	X	X	X	X	X			
Three Factor Eating Questionnaire (TFEQ)		X	X	X	X	X	X	X		X	X
Monetary Choice Questionnaire (MCQ)		X	X	X	X	X	X	X		X	X
Cigarette Abstinence Self-Efficacy (CASE)		X	X	X	X	X	X	X		X	X
Alcohol Abstinence Self-Efficacy (AASE)		X	X	X	X	X	X	X		X	X
Inventory of Drinking Situations (IDS)		X									
Impaired Control Scale (ICS)		X									
Barratt Impulsivity Scale (BIS-11)		X									
UPPS Impulsive Behavior Scale (UPPS)		X									
Systematic Assessment for Treatment Emergent Effects (SAFTEE)		X	X	X	X	X	X	X	X	X	X

Reward Based Eating Drive Scale (R-BEDS)		X	X	X	X	X	X	X		X	X
Simplified Nutritional Appetite Questionnaire (SNAQ)		X	X	X	X	X	X	X		X	X
Control of Eating Questionnaire (CEQ)		X	X	X	X	X	X	X		X	X
Wisconsin Inventory of Smoking Dependence Motives (WISDM)		X									
Sweet Taste Preference Test		X						X			
Center for Epidemiological Studies Depression Scale (CES-D)		X									
Reward Probability Index – Environmental Reward Observation Scale (RPI-EROS)		X									
Toronto Alexithymia Scale (TAS-20)		X									
Profile of Mood States – Short Form (POMS-SF)										X	X
Biphasic Alcohol Effects Scale (BAES)										X	X
Alcohol Urge Questionnaire (AUQ)										X	X
Drug Effects Questionnaire (DEQ)										X	X
Obsessive-Compulsive Drinking Scale		X									
Graded Pain Scale		X									

2 INTRODUCTION

2.1 STUDY RATIONALE

Pharmacotherapy development is a critical objective for reducing health and societal burdens associated with alcohol use disorder (AUD) [1-3]. Despite advances in this area, medication development remains a slow- moving endeavor with a protracted path from drug discovery to market [2-4]. Given the high degree of heterogeneity in AUD clinical presentation and treatment response, it is recognized that no single medication will be effective for all patients [5]. Therefore, the National Institute on Alcohol Abuse and Alcoholism (NIAAA) medication development strategy specifies the long-term objective of establishing a repertoire of therapies to facilitate targeted interventions for specific AUD subgroups [3, 5, 6]. Strategies for expediting the study of novel treatments are central to these aims [3, 5, 6].

Epidemiological and clinical evidence shows that cigarette smokers comprise a sizable AUD subgroup with disproportionately high health burdens [7, 8] Alcohol and cigarette use are two of the three leading causes of preventable mortality worldwide, jointly accounting for an estimated seven million deaths per year [9]. Population-based research shows that cigarette consumption increases as a function of alcohol use severity [7]. In one large epidemiological study, nearly half (44.6% of men, 47.3% of women) of those with alcohol dependence also met criteria for nicotine dependence [7]. Co-use of alcohol and cigarettes predicts synergistic increases in risk for numerous disease conditions [8, 10, 11], including esophageal and laryngeal cancers [12, 13]. Given these health burdens, increasing effort has been allocated to developing targeted interventions for smokers with AUD [8, 11, 14, 15].

In the absence of FDA-approved medications for concurrent alcohol and nicotine addiction, identifying candidate drugs for this indication is a priority [11, 15, 16]. Several recent studies have examined whether FDA- approved AUD and smoking cessation drugs, either alone or in combination, can facilitate joint reductions in alcohol and cigarette use [11]. For instance, naltrexone reduces alcohol consumption in heavy-drinking smokers [17, 18], and in some cases reduces cigarette consumption [17, 19]. Recent findings further suggest that naltrexone's efficacy for reducing drinking is stronger in AUD participants

who smoke relative to non-smokers [20, 21]. However, a Cochrane review [22] and a large, prospective AUD trial [19, 23] concluded that naltrexone does not appear to reduce cigarette smoking [11, 23].

Varenicline has also been tested as a candidate therapy for smokers with heavy drinking or AUD. Informed by early findings that varenicline reduced alcohol self-administration in a human laboratory trial [16], large-scale clinical trials supported efficacy of varenicline for reducing alcohol consumption in heavy-drinking smokers [24, 25] and those with AUD [26]. Recent laboratory-based studies have also examined the combination of varenicline and naltrexone as a novel treatment [27, 28]. Because those with concurrent alcohol and nicotine addiction reflect a high-priority subgroup, further efforts are needed to screen candidate treatments for this population [8, 11, 28] . As reflected in these recent trials, and as noted explicitly under the NIAAA medication development framework [1-3], drug repurposing strategies can significantly expedite new treatment approaches by capitalizing on therapies that have already passed the extensive regulatory steps required for FDA approval.

Compounds with preclinical evidence for reducing both alcohol and nicotine intake are particularly good candidates for investigating new treatments for heavy-drinking smokers. Accumulating evidence suggests that *Glucagon-like peptide 1* (GLP-1) receptor agonists may have promise in this respect. GLP-1 is an incretin hormone produced and secreted both peripherally (in the intestines) and centrally (in the hindbrain) [29]. GLP-1 is stimulated following food consumption and stimulates satiety and decreases in blood glucose. As a peptide with both hormonal and neurotransmitter functions, GLP-1 plays a role in the "gut-brain axis," which has recently been invoked as a target for addiction therapies [30]. GLP-1 agonist medications were designed to mimic the function of the endogenous GLP-1 peptide, with the benefit of a much longer half-life than endogenous GLP-1. Based on their glucose-lowering and appetite-reducing properties, GLP-1 agonists were developed as anti-diabetic therapies.

Several GLP-1 receptor agonists are now in widespread use as treatments for type II diabetes (T2D), including exenatide, liraglutide, lixisenatide, albiglutide, dulaglutide and semaglutide. These medications have also proven efficacious for promoting weight loss, reducing glycated hemoglobin (HbA1c), and reducing risk for some cardiovascular events [29]. Although the primary indication of GLP-1 agonists is T2D, these therapies are increasingly being studied for other indications in non-diabetic patients, including obesity/weight management.

Of note, GLP-1 agonists require subcutaneous administration, typically by way of a pre-filled medication cartridge with a micro-needle attachment, which allows for easy self-administration in the home without assistance from medical staff. Semaglutide is a recently approved (2017) FDA-approved GLP-1 agonist. A new oral formulation of semaglutide was approved by the FDA in 2019, making semaglutide the first drug in its class to be available in both subcutaneous and oral formulations.

2.2 BACKGROUND

Accumulating evidence from preclinical studies suggests that GLP-1 agonists reduce alcohol and other drug intake and/or reinstatement in animal models. In a recent review of 17 preclinical studies [31], all studies reported significant effects of GLP-1 agonists on indices of drug motivation and intake, with the evidence being most robust for alcohol [30, 31]. Notably, the effects of GLP-1 agonists on drug intake appear to extend to several addictive drugs, including nicotine [32]. These findings have led to recent studies aiming to investigate the efficacy of GLP-1 agonists as candidate therapies for reducing intake or reinstatement of alcohol, nicotine, and other drugs (e.g., cocaine). Although several human trials are

underway in various regions, no RCTs have been published to date, and human data on the efficacy of GLP-1 agonists for reducing drug intake in substance-using populations is needed.

Although the precise mechanisms mediating the effects of GLP-1 agonists on alcohol-related behaviors remain to be clarified, it has been suggested that cholinergic-dopaminergic pathways are involved in altering the reward value of alcohol following exposure to GLP-1 agonists [30]. Additionally, GLP-1 agonists appear to exert their effects in part by way of interactions with the serotonin (5-HT) system, including interactions with 5-HT2C receptors. Specifically, GLP-1 agonists appear to induce serotonin release, and serotonin signaling appears critical to the efficacy of the medications [33, 34]. Some evidence also suggests that 5-HT2C receptors appear necessary for the efficacy and satiety-inducing effects of GLP-1 agonists [35, 36].

Of the approved FDA-approved GLP-1 agonists, most studies have focused on exenatide, an older GLP-1 agonist first approved in 2005. As noted, semaglutide is the newest GLP-1 agonist and the first available in both subcutaneous and oral formulations. Some evidence suggests superiority of semaglutide relative to other GLP-1 agonists with respect to efficacy for glycemic control [37]. Although clinical trials of GLP-1 receptor agonists in participants with substance use disorder are now underway, no studies (to our knowledge) are currently underway to examine semaglutide as a treatment for alcohol use disorder, or for the joint indication of alcohol use disorder and tobacco use disorder. Demonstrating tolerability and initial efficacy of semaglutide for these populations could ultimately allow for greater clinical flexibility if GLP-1 agonists prove efficacious for these populations, given that semaglutide is available in both subcutaneous and oral formulations.

Human laboratory methods for Phase II medication screening

As emphasized in the NIAAA medication development strategy [1, 4, 38], human laboratory methods play a pivotal role in drug development by providing a bridge from preclinical studies and full-scale Phase II/III trials [39-43]. Importantly, laboratory studies can expedite drug development by providing an efficient, cost-effective, and rigorous option for "early-Phase II" medication screening, thereby informing priorities for larger and more costly randomized trials. For example, we recently published meta-analytic evidence that human laboratory studies of naltrexone, when examined in aggregate, yield conclusions highly comparable to the aggregate data from large-scale randomized trials, reinforcing the notion that laboratory trials offer a valid and efficient approach for estimating the potential efficacy of candidate therapeutics [40]. Other advantages of laboratory trials include the ability to study putative treatment mechanisms under controlled conditions, and the ability to quickly generate initial data on drug tolerability in the clinical population of interest [1, 44].

Phase II pharmacotherapy trials for alcohol use disorder often rely on alcohol administration procedures to study medication effects on acute responses to alcohol in human laboratory settings [40]. Controlled alcohol exposure offers a means of testing medication effects on subjective response to alcohol (e.g., stimulation, sedation) and alcohol-induced craving. These subjective responses to alcohol are targets of existing pharmacotherapies, such as naltrexone [40]. Alcohol self-administration has also been used as a laboratory model of alcohol motivation, and has successfully been used to screen candidate AUD therapies [40]. Some have argued that alcohol self-administration should serve as the central endpoint in human laboratory studies, and validated procedures exist for modeling alcohol self-administration safely in the laboratory [16] [40]. More recently, behavioral economic measures have also been utilized as a means of studying drug motivation in laboratory contexts, and appear sensitive for detecting experimental manipulations on alcohol-related motivation. For example, *alcohol demand* indexes the

relative reinforcing value of alcohol at a particular point in time [45]. Measures of drug demand, such as the alcohol purchase task (APT) and cigarette purchase task (CPT), are considered objective and sensitive experimental assays of drug motivation [45]. The APT predicts frequency of heavy drinking days after treatment [45], and is sensitive to temporal changes in alcohol's relative reinforcing efficacy during drug administration [46]. Notably, initial evidence suggests that these measures are sensitive to the effects of pharmacological treatments, including naltrexone and varenicline [47, 48]. Measures of drug demand offer an alternative to studying self-administration in the laboratory, complementing measures of subjective drug responses.

One limitation of many laboratory-based pharmacotherapy trials is the tendency to focus on heavy drinkers without AUD, rather than participants who report symptoms of the disorder [49, 50]. A recent systematic review found evidence that recruiting non-clinical samples for laboratory trials can potentially limit the ability of laboratory findings to generalize to larger clinical trials, perhaps undermining translational research efforts [51]. While ethical considerations normally preclude laboratory alcohol administration methods with treatment-seeking AUD participants [50], studies of *non-treatment-seeking* AUD participants are ethically justified, and can provide important data on medication effects and safety in the target clinical population [52]. Additionally, focusing on participants with <8 of out 11 AUD symptoms endorsed) can ensure maximal safety when studying this population by eliminating the likelihood of clinically significant withdrawal or alcohol-related medical complications. Our team has ample prior experience with alcohol administration procedures in both clinical and non-clinical populations; we will utilize these methods to study the potential efficacy of semaglutide in participants with AUD.

2.3 RISK/BENEFIT ASSESSMENT

2.3.1 KNOWN POTENTIAL RISKS

Semaglutide (Ozempic®) received FDA approval in 2017 for use as an adjunct to diet and exercise to achieve glycemic control in adults with type 2 diabetes mellitus (T2D). Semaglutide received FDA approval based on the results of 25 completed trials with the goal of providing results that would be applicable to a broad type II diabetes (T2D) population, including the elderly, patients with renal impairment, and patients at high cardiovascular risk. These trials involved > 9,300 participants, of whom >5,700 were treated with semaglutide. In these trials, treatment with semaglutide alone was efficacious for reduction in hemoglobin A1C (HbA1c) below 7% (e.g., 73% of participants had HbA1c <7% at week 30, compared to 28% of placebo-treated patients). Significant body weight reductions were also reported, with the weight loss achieved at end-of-treatment ranging from -1.43 to -4.28 kg (corresponding to 2.3–4.9%). Weight reductions were evident after 4 weeks of treatment, and persisted up to 104 weeks [75]. The peak plasma concentration of semaglutide is reached 1-3 days post-dose. Steady-state exposure is achieved following 4-5 weeks of once-weekly administration, and elimination half-life is approximately 1 week.

<u>Risks related to medication.</u> The most commonly reported side effects/adverse events (AEs) are gastrointestinal, with nausea being the most frequently reported symptom. The majority of reports of nausea (0.5 mg dose vs placebo; 15.3 vs. 6.1%), vomiting (5.0 vs 2.3%), and/or diarrhea (8.5 vs 1.9%) occur shortly following a dose escalation. Abdominal pain (7.3 vs 4.6%) and constipation (5.0 vs 1.5%) were also reported [76]. In studies ranging from 30-56 weeks in duration, the discontinuation rate due

to these gastrointestinal AEs was ranged from 5-13%, with this number increasing during longer treatment durations [77].

Rarer AEs (occurring at a frequency of <5%) included symptomatic hypoglycemia (≤70 mg/dL glucose threshold), which occurred in 1.6% of semaglutide-treated participants versus 0% placebo participants; dyspepsia (3.5 vs 1.9%); eructation (2.7 vs 0%); and gastroesophageal reflux disease (1.9 vs 0%). In placebo-controlled trials, injection site reactions (e.g., discomfort, erythema) were reported in only 0.2% of semaglutide-treated patients, suggesting good tolerability of subcutaneous administration. Patients exposed to semaglutide had a mean increase from baseline in amylase of 13% and lipase of 22%. Cholelithiasis was reported in 1.5% of treated patients. The 0.5 mg dose resulted in a mean increase in heart rate of 2 to 3 beats per minute, while there was a mean decrease in heart rate of 0.3 beats per minute in placebo-treated patients. Hypersensitivity reactions (e.g., anaphylaxis, angioedema) have been reported with GLP-1 receptor agonists. [76]. Other adverse reactions with a frequency of >0.4% associated with semaglutide include fatigue, dysgeusia and dizziness [76].

Rodent studies have shown an increase in the incidence of thyroid C-cell tumors after lifetime exposure of semaglutide at clinically relevant plasma levels. However, several studies in T2D patients taking GLP-1R agonists have shown no increase in neoplasm risk, even after several years of use [78]. Nevertheless, a personal or family history of medullary thyroid cancer or multiple endocrine neoplasia 2A or 2B is considered a potential risk factor for taking the medication, and will be an exclusionary criterion.

In a 2-year trial, acute pancreatitis was observed in 8 semaglutide-treated patients (0.27 cases per 100 patient years) and 10 placebo-treated patients (0.33 cases per 100 patient years). One case of chronic pancreatitis was confirmed in a semaglutide-treated patient. History of pancreatitis will be an exclusion factor, and study participants receiving semaglutide will be observed for signs and symptoms of pancreatitis (via blood monitoring of serum amylase and lipase). If pancreatitis is suspected, semaglutide will be discontinued, and appropriate management recommended.

Other rare events include postmarketing reports of acute kidney injury and worsening of chronic renal failure, which may sometimes require hemodialysis, in patients receiving long-term treatment with GLP-1 receptor agonists. Some of these events have been reported in patients without known underlying renal disease. Most of these events occurred in patients who had experienced nausea, vomiting, diarrhea, or dehydration. Therefore, we will monitor renal function by including a measure of serum creatinine at baseline and at subsequent visits involving blood samples.

Although most safety data on semaglutide comes from trials with T2D participants, there are now a number of randomized trials underway examining GLP-1 receptor agonists in participants with substance use disorder, including trials on tobacco, cocaine, alcohol, cannabis, and opioids. A full list of registered randomized trials can be found by searching for “GLP-1” at ClinicalTrials.gov. This study will be the first to our knowledge to focus specifically on heavy-drinking smokers. The use of a short-term medication screening protocol (lasting roughly 10 weeks) will provide an opportunity to demonstrate tolerability and generate data on side effect profiles in this population while minimizing any risks associated with longer-term medication exposure.

<u>Risks Related to Questionnaire and Interview Assessments.</u> There may be potential risks associated with assessment procedures. Participants may find the battery of psychiatric and psychological assessments tedious or intrusive. Participants may experience discomfort resulting from questions about personal histories or substance use patterns.

Participation in Alcohol Administration Sessions. Other potential risks include typical risks related to participation in alcohol administration studies. For example, it is possible that participants may experience dizziness/nausea from drinking during these visits, or symptoms the following day (e.g., headache). Our group has considerable experience with alcohol administration and self- administration procedures [80-84], and the safety precautions required in these studies. Across these studies, the incidence of adverse events such as vomiting has been exceptionally low. Our procedures will follow the NIAAA recommended guidelines for administering alcohol to human subjects (https://www.niaaa.nih.gov/Resources/ResearchResources/job22.htm). Briefly, the NIAAA guidelines include recommendations for administering alcohol to participants who meet criteria for AUD. In particular, we have taken measures to protect against ethical concerns by *excluding treatment-seekers* (or those engaged in an active quit attempt), as per the NIAAA recommended guidelines. Additionally, we will screen out participants with severe AUD (defined as 6 or more of 11 symptoms). This approach allows us to generate data relevant to a clinical population while minimizing risks.

Risks Related to DNA Collection. The purpose of collecting saliva for DNA in this study is to allow secondary analyses to examine whether medication effects might be different according to certain genetic factors. These analyses may also focus on genetic factors potentially associated with drug use or related behaviors, such as nicotine and alcohol metabolism, dopaminergic function, or impulsivity. Potential risks associated with DNA collection involve potential identification, and there may be risks with genetic (DNA) tests that are as yet unknown. Genes may be shown at some point in the future to be related to mental illnesses or tendency to addiction. Samples will be labeled with participant ID only, and analyzed at an outside laboratory. Samples will be de-identified and links with participant identifiers will be broken upon completion of the analyses. We will not conduct any whole genome sequencing of the DNA samples. Additionally, the consent form will state that participants have the option to opt out of DNA collection, and to request that their samples are destroyed at any time during the study. Participants will not receive any feedback from any of the genetic tests from study staff.

2.3.2 KNOWN POTENTIAL BENEFITS

The participants in this study are not expected to benefit directly from their participation. However, the knowledge gained from this study could lead to a better understanding of a medication with potential to treat alcohol and nicotine addiction, and potentially other substance use disorders.

2.3.3 ASSESSMENT OF POTENTIAL RISKS AND BENEFITS

Given the vast health implications of substance use disorder and the currently limited array of effective medications, it is imperative to pursue novel pharmacotherapeutic options. A key strategy in this regard is to “repurpose” medications that are already approved (and have proven safe) in the context of other health conditions. Given the sizable amount of research on GLP-1 receptor agonists in other clinical populations, coupled with preclinical evidence that these medications reduce motivation for alcohol, nicotine, and other drugs, evaluating this medication class in the proposed population is warranted. Given no available pharmacotherapies for the indication of concurrent alcohol and nicotine addiction, any evidence for potential efficacy in heavy-drinking smokers could have substantial implications. From this standpoint, the risk/benefit profile for testing semaglutide in a population of non-diabetic participants is favorable.

Although we anticipate the risk of adverse events to be low based on the safety profile of the medication, gradual dose escalation, and relatively short duration of treatment, several steps will be taken to track and report medication-related side effects. As described further in later sections of this application, participants will be monitored regularly by virtue of attending weekly lab visits for medication delivery, at which side effects will be assessed and clinical lab results monitored. Participants will be given a contact number for study staff in case of emergent side effects (and instructed to seek medical attention in the event of any serious side effects), and participants will be contacted by phone shortly after dose escalations to monitor symptoms. We expect some participants to experience nausea shortly following dose escalations, but that these symptoms will usually be transient.

Given the focus on a non-obese population, we will be particularly vigilant in monitoring changes in body weight and blood glucose, and will exclude participants at the outset with body weight or blood glucose levels below normal range, based on physician judgement. We will carefully monitor participants with glucose tests and weight measurements at regular study visits (see schedule of activities table), and will instruct participants to be vigilant for potential symptoms of hypoglycemia. Periodic blood tests to measure fasting glucose, fasting insulin, serum lipase and amylase, serum creatinine, and serum calcitonin will occur at baseline and regularly thereafter (i.e., at visits prior to dose escalations). All adverse events will be documented and reported in accordance with relevant policies, and the regulations of NIH. We have enlisted the TraCS DSMB to assist with safety monitoring of the study. The board will convene at the beginning of the study and at scheduled points to review study progress and safety data, and to review any SAEs and considerations related to stopping rules (described further below).

Considering the use of an FDA approved medication that has been deemed safe in the context of chronic administration for glycemic control, the risks related to medication use in this short-term screening trial are acceptable in comparison to the potential benefits of the proposed research (i.e., demonstrating potential efficacy of a new medication for substance use disorder).

Risks Related to Alcohol Administration Sessions. Alcohol administration procedures are commonly used to study risk factors for alcohol use disorder (AUD), and have proven capable of identifying successful pharmacotherapies [40]. As per the NIAAA guidelines for administration of alcohol in human studies, such studies are typically restricted to non-treatment-seeking participants in order to minimize ethical concerns, as will be the approach in this study. Further details on safety procedures are provided later in this document.

3 OBJECTIVES AND ENDPOINTS

OBJECTIVES	ENDPOINTS	JUSTIFICATION FOR ENDPOINTS
Primary		
To examine effects of semaglutide vs. placebo on alcohol self-administration under laboratory conditions.	Amount of alcohol consumed and breath alcohol concentration (co-primary endpoints) during a validated laboratory alcohol self-administration protocol. In this procedure, participants are provided the option of choosing between drinks and	Primary: This outcome is a laboratory assay of alcohol intake, and has been used in prior human laboratory studies to screen candidate medications, allowing comparisons with effect sizes for other medications. This session will be conducted during the standard

OBJECTIVES	ENDPOINTS	JUSTIFICATION FOR ENDPOINTS
	monetary reward. The comparison of interest is the difference between medication and placebo groups in the change in laboratory consumption between baseline and the maintenance dose of 0.5mg.	maintenance dose (0.5mg) to mimic the common clinical scenario.<br><br>Secondary: Subjective alcohol responses and alcohol/cigarette demand are validated measures of drug reward/motivation, and are known to be sensitive to medication effects. Given substantial individual differences in alcohol pharmacokinetics, a within-subjects analysis is preferable for these endpoints (such that each participant will serve as their own control). Laboratory sessions will take place at two time points: baseline and 0.5mg maintenance dose.
Secondary		
To examine group differences in 1) alcohol/cigarette demand and subjective responses to alcohol during alcohol administration, and 2) naturalistic drinking and smoking behaviors over the course of the 10-week treatment period.	Laboratory measures of subjective responses to alcohol, alcohol demand, and cigarette demand during controlled laboratory alcohol administration (lab sessions will take place at baseline and again after achieving the maintenance dose).<br><br>Number of standard drinks consumed and number of cigarettes consumed per day during the treatment phase (Weeks 0-10), as measured by a daily diary report.<br><br>Similar to the primary outcome, secondary outcome analyses will examine medication vs. placebo group differences in within-subjects changes in these outcomes over time (baseline to 0.5mg).	Laboratory measures of alcohol response and alcohol/cigarette demand are clinically valid indicators of drug motivation, and are demonstrated to be sensitive to medication effects. Investigating these outcomes under controlled conditions will allow us to test for a medication “signal” on measures of alcohol and nicotine motivation.<br><br>Daily measures of alcohol consumption (standard drinks per day) and cigarettes per day are selected to afford greater power for modeling change in consumption over the treatment period. Note that these outcomes are considered secondary because this Phase II trial involves non-treatment-seeking participants who are not attempting to reduce alcohol or use or cigarette smoking.
Tertiary/Exploratory		
Body weight, HbA1c, alcohol elimination.	Changes in body weight, HbA1c, and alcohol elimination will be examined as exploratory endpoints based on potential clinical relevance.	GLP-1 receptor agonists lower HbA1c and body weight in indicated populations (e.g., Type II diabetes, obesity). Given high co-occurrence of obesity and alcohol use disorder, reporting whether medication is

OBJECTIVES	ENDPOINTS	JUSTIFICATION FOR ENDPOINTS
		associated with these reductions in the present sample is clinically relevant. Data from the alcohol administration sessions will be used to estimate whether medication predicts changes in alcohol elimination during treatment, and whether changes in body weight account for any such differences. These outcomes are exploratory.

4 STUDY DESIGN

4.1 OVERALL DESIGN

Overview of Study Design.

The aim of this protocol is to translate preclinical findings on GLP-1 agonists by conducting an early-Phase II investigation of semaglutide in non-treatment-seeking participants with AUD, with a focus on human laboratory outcomes. Given preclinical evidence for the potential efficacy of GLP-1 agonists for reducing both alcohol and nicotine intake, and the need for treatments targeting both alcohol and nicotine addiction, this proposal focuses on cigarette smokers with AUD.

This is a single-site, Phase II, two-arm, double-blind (participants, outcome assessors), randomized, placebo-controlled, dose-ranging study. Following a baseline assessment eligible participants will be randomized to medication or placebo conditions by the IDS staff at a 1:1 ratio. The treatment period will be approximately 10 weeks, taking into account the dose escalation necessary for this medication. Those randomized to semaglutide will receive 0.25mg/week for 4 weeks (Weeks 1-4), and 0.5mg/week for 4 weeks (Weeks 5-8). Provided that participants have tolerated the medication , participants will receive a final dose of 1.0mg (Week 9), and will return for a discharge lab visit with repeat lab tests at Week 10, followed by a final monitoring assessment by phone scheduled approximately 6 weeks after the last medication dose (Week 15). Those in the placebo condition will receive sham placebo administration at each visit (described in further detail in later sections). Participants will attend weekly visits at Eastowne CRU or CTRC to receive medication or placebo doses.

In addition to the medication visits, participants will be asked to attend four laboratory visits: two involving alcohol self-administration (one prior to the first medication visit and one during the 0.5mg treatment phase) and two involving alcohol challenge (one prior to the first medication visit and one during the 0.5mg treatment phase). These sessions are designed to assess changes in alcohol responses over the course of the treatment period. Laboratory sessions are described in further detail below. Measurements of naturalistic alcohol and cigarette use will also be recorded throughout the treatment period. These assessments will be captured using a daily diary that will be provided to participants. Participants will receive an automated text message daily via Phone.com as a reminder to complete the diary. Additional self-report measures of substance use will be collected at study visits.

As summarized above, the primary objectives are to examine whether treatment with semaglutide alters alcohol self-administration (primary outcome) and laboratory measures of alcohol response and motivation (i.e., demand and responses to alcohol, secondary outcomes) during alcohol administration. We hypothesize that the semaglutide treatment (versus placebo) will be associated with reduced alcohol self-administration during a laboratory session conducted at the maintenance dose of 0.5mg. We further hypothesize that semaglutide will lead to reductions in alcohol demand, cigarette demand, and subjective responses to alcohol (e.g., reduced stimulation) during alcohol administration sessions.

The primary and secondary outcomes for this trial will be assessed during exposure to the standard maintenance dose (.5mg). While an extended treatment phase at the 1.0mg dose is beyond the scope of this small-scale trial, the inclusion of a brief (1-week) phase at 1.0mg is designed to provide initial data on tolerability and any changes on selected secondary outcomes (i.e., drinks per day and cigarettes per day) in this population. Capturing these data will help to support feasibility and pilot data for subsequent funding proposals with longer treatment intervals.

Secondary objectives include examining changes in naturalistic alcohol consumption and cigarette smoking during the treatment period. Although participants will not be attempting to reduce drinking or smoking in this trial, it is hypothesized that treatment with semaglutide will result in reductions in alcohol consumption (drinks per day) and cigarettes per day over the course of the treatment period. Tertiary/exploratory outcomes include examining changes in body weight, HbA1c, and alcohol elimination during the trial.

In total, participation in this study is estimated to take a maximum of up to 50 hours of combined time over a period of up to approximately 16 weeks beginning with initial screening. This includes a screening and baseline visit (up to 4 hours), up to 9 subsequent laboratory visits for medication administration and side effects monitoring (up to 1 hour each), a final discharge visit (Week 10), and final telephone check-in (Week 15). If necessary for safety reasons, (e.g., based on lab results), participants may be asked to attend (unplanned) visit(s) for follow-up labs at the discretion of the study physician. Participants will also complete four visits involving alcohol administration over the course of Weeks 0-10; these sessions may last up to 7-8 hours each. Participants also will receive calls from staff 2-3 days after each medication phase begins (placebo, 0.25mg, 0.5mg, 1.0mg) to check in on side effects (10 minutes per call), and will be asked to complete brief daily questionnaires via a daily diary with reminders sent via daily text message prompt (1-2 minutes per day).

A detailed summary of study procedures is provided below.

Detailed Study Procedures.

Following initial recruitment (described in section 5.5), participants will be asked to complete the following procedures:

a) <u>Telephone screen</u>

Initial screening will be conducted by telephone by trained research staff and will assess participant alcohol, cigarette, and drug use, medications, and basic medical inclusion/exclusion factors. Potential participants will be given a brief description of the study. The initial telephone screen is estimated to take roughly 15-30 minutes. Contact information will be collected from interested callers who pass the initial screen. Following the initial screen, participants will proceed to be booked for an informed consent and baseline interview session. The informed consent procedures

will be conducted remotely using a HIPAA-compliant virtual meeting platform (e.g., Webex, Zoom) and Docusign. Depending on participant and staff availability, the phone screening and informed consent/baseline interview can happen on the same day (after the screening call) or at a future date.

b) Screening and Baseline Data Collection Visits

At the informed consent session, a member of the research staff will review the study procedures and carry out informed consent procedures (including electronic signature via Docusign) prior to proceeding to the baseline interview. Following informed consent, the staff member will conduct a semi-structured interview to assess inclusion/exclusion criteria. A research assistant will use a structured interview to measure diagnostic and substance use screening criteria, including symptoms of alcohol use disorder; inclusion/exclusion factors related to medical history will also be assessed (see criteria listed above). The Structured Clinical Interview for DSM-V [53] will be used to assess AUD and substance use disorder symptoms. History of other psychiatric diagnoses and medication use will be assessed via self-report. The research assistant will also collect information on demographic factors, criteria related to current medical status and medications, current alcohol, tobacco and other substance use (see "Phone Screen" document), as well as substance use disorder symptoms (via select modules from the SCID interview) and symptoms other psychiatric symptoms (using the MINI screening module). Participants who appear eligible will be booked for an in-person visit, which will take place at the laboratory site (Medical Wings C), with some procedures (e.g., blood draw) booked at CTRC as necessary.

The in-person screening/baseline visit will include a brief review of informed consent to answer any additional questions, and a brief review of self-report and interview questions from the remote screening session. The Timeline Follow-Back (TLFB) [54] will be used to assess alcohol, cigarette and other drug use in the 90 days prior. The participant will also provide a breathalyzer screen. Participants who have a positive BAC (greater than 0.00) will be monitored until their BAC is reduced to a safe level (BAC<.03g%), in accordance with NIAAA guidelines (or to 0.00g% in the event that the participant drove to the session).

Participants who remain eligible after the interview will complete vitals and measurements (HR, BP, height, weight, temp), and a measure of expired alveolar carbon monoxide (CO). A baseline saliva sample for DNA analysis will also be collected. Additionally, participants will provide a urine sample and complete a blood draw (up to 70-80 ml) for a comprehensive metabolic panel (e.g., Na, K, Cl, CO2, BUN, Creatinine, Glucose, Calcium, Albumin, Total protein, Total bilirubin, AST, ALT, Alk Phos), baseline measure of HbA1c, baseline measure of nicotine metabolites (e.g., 3HC, COT), serum amylase and lipase, and complete blood count (e.g., Auto WBC, Corrected WBC, RBC, HGB, HCT, MCV, MCH, MCHC, RDW, MPV, Platelet). These procedures will be conducted by the research assistant, and the baseline blood draw will be taken by a team member with phlebotomy training. If there is no team member with phlebotomy training available that day, the blood draw may also be booked at CTRC. If needed, the blood draw and other baseline procedures may be booked as separate visits. On the day of the visit that includes the blood draw, participants will be instructed to arrive fasting for 12 hours (to allow for assessment of baseline HbA1c and lipids). If participants are not able to arrive fasting for the baseline session, the fasting lipids sample may be deferred or completed at the next possible visit. Blood samples will be sent to McClendon laboratory for testing. A portion of the blood sample (de-identified) will also be stored (Thurston Bowles building and Neurosciences Research

Building) for subsequent analysis (e.g., nicotine metabolites and blood neutrophils, which are affected by cigarette smoking). Neutrophil analyses will be conducted by Dr. Robert Tarran's lab. The saliva sample will also be stored at Thurston Bowles building. The urine sample will be used to test for the presence of several drugs (for example, heroin, cocaine, methamphetamines, opiates). (For females, the test will include a pregnancy screen.) If a participant tests positive for drugs other than cannabis (or if females screen positive for pregnancy), the participant will be excluded from the study.

Participants will complete baseline questionnaires, which will be collected on paper. A Demographic Screening Questionnaire will assess basic demographic factors (e.g., age, education, income). A Locator form will collect contact information for three individuals who can be contacted if a participant is unable to be reached by study staff for follow-up visits. Other baseline measures will include the Alcohol Use Disorders Identification Test (AUDIT) [55], Self-Report of the Effects of Alcohol Questionnaire (SRE) [56, 57], Fagerström Test of Nicotine Dependence (FTND) [58], and the Nicotine Dependence Syndrome Scale (NDSS) [59], Barratt Impulsivity Scale (BIS-11) [60], UPPS Impulsive Behavior Scale (UPPS) [61], Impaired Control Scale (ICS) [62], Center for Epidemiological Studies Depression Scale (CES-D) [63], Toronto Alexithymia Scale (TAS-20) [64], the Reward Probability Index (RPI) and the Environmental Reward Observation Scale (EROS) [65]. Baseline measures that will be repeated at subsequent visits will include the Timeline Followback (TLFB), the Penn Alcohol Craving Scale (PACS) and Tiffany Questionnaire of Smoking Urges (TQSU) [66], and the Wisconsin Smoking Withdrawal Scale (WSWS) [67]. To assess potential medication effects on changes in motivation and self-efficacy for resisting alcohol/cigarettes, we will use alcohol and smoking Contemplation Ladder items, Alcohol and Cigarette Abstinence Self-Efficacy (AASE/CASE) (these scales will be repeated at each visit) and the Inventory of Drinking Situations (IDS) scales [68-70]. Appetite and eating behavior will be assessed with the Simplified Nutritional Appetite Questionnaire (SNAQ) [71], Control of Eating Questionnaire (CEQ) [72], the Three Factor Eating Questionnaire (TFEQ) [73] and the Reward-Based Eating Drive Scale (R-BEDS) [74]. During the medication phases, side effects will be assessed with the SAFTEE questionnaire [75], a standard tool for alcohol clinical trials. Participants will also complete previously validated Purchase Tasks for alcohol, cigarettes, and food, and delay discounting will be assessed with the Monetary Choice Questionnaire (MCQ) [76-79]. These brief behavioral economic tasks assess perceived value of reinforcers by presenting hypothetical scenarios involving choices between the reinforcer (alcohol, cigarette, food) and monetary rewards; these measures will be completed at each visit. Finally a previously validated sweet taste preference test will be administered at baseline and again on the final visit, since this phenotype has been shown to be implicated in both food and alcohol reward, and may be modulated by a GLP-1 agonist. This test involves participants tasting solutions of sugar water prepared to reach .05, .1, .2, .42, and .83 molar sucrose, and then rating preference and intensity of the taste. The sweet taste test will be overseen by a research assistant using previously published protocols from prior projects at UNC [80, **Error! Hyperlink reference not valid.**].

The baseline questionnaires and interview assessments are projected to take approximately 3-4 hours combined. To accommodate potential scheduling/time constraints or delays at the baseline visit, some questionnaires that are not time-sensitive (for instance, personality questionnaires) may be deferred until a later lab visit as needed, at the discretion of the study team. All interview records and paper questionnaires will be stored in study binders in a secure location for subsequent entry into an electronic database for analysis. Patients will be given

breaks as needed. Following the blood draw, participants will be offered a snack and bottled water (given the instructions to arrive fasting). If results of the baseline screening/medical assessment indicate that the participant is tentatively eligible, they will be given a comprehensive orientation to the next phases of study, including the follow-up medication visits, the laboratory alcohol administration visits, and the daily surveys. Locator/collateral contact information will also be obtained. A Locator form will collect contact information for three individuals who can be contacted if a participant is unable to be reached by study staff for follow-up visits. Following the completed baseline assessments, the participant’s screening summary will be reviewed for eligibility, including review of medical criteria and any medications that the participant is taking by the study physician. Lab results will be uploaded to the EMR and reviewed by the study physician prior to prescribing the medication. Potential interactions with semaglutide will be verified using an established online database such as Epocrates or Lexicomp. In the event of any uncertainties about medication interactions, participants will be asked to contact their primary care physician to confirm no concerns with taking semaglutide.

For eligible participants, the study physician will provide sign-off on all medical criteria (e.g., medical history, medications, lab results), and the PI will provide sign-off on other baseline criteria. Once participants have been confirmed as eligible, the physician will submit an order to start medication or placebo. Medication and placebo will be dispensed at CTRC (Week 1 visit) at a date coordinated with the participant. Participants will be advised to be vigilant for symptoms of hypoglycemia at the medical visit, and instructed to contact the study team to review any symptoms that emerge (regular glucose tests will also be obtained at follow-up visits, as noted in the schedule of assessments). If necessary, the medication review will take place via telephone or telemedicine, depending on medical team availability.

Participants’ Week 1 visit will be scheduled with enough lead time to review lab results and exclude participants (if needed) prior to administering the study medication. Additionally, if clarification of medical criteria is needed at any point during or following the phone or in-person screening process, or at any subsequent stage of the study, the study physician may meet with the participant via a remote contact method (e.g., phone, Webex/Zoom, or telehealth) to clarify medical questions or discuss test results.

c) <u>Daily Assessments.</u>

Throughout the study, participants will be asked to complete a brief self-report survey each day that includes measures of 1) prior-day number drinks consumed; 2) number of cigarettes smoked; and 3) prior day peak craving/demand for alcohol and cigarettes. Participants will be provided with a paper booklet in which to enter responses and will be asked to bring the booklet to each visit. At each visit the research staff will collect the interim daily surveys for retention in the participant’s study binder. Participants will be texted an automated text message each day during their participation in the study as a reminder to complete the daily survey. If preferred, participants can opt to receive an email link. (No daily survey data will be collected from participants via text or email.) At each study visit, a staff member will provide compensation for the prior week’s surveys, and will review survey adherence and progress towards compensation for good adherence (described later), as a means of motivating adherence to self-report assessments. In the event a participant completes the daily assessments but forgets to bring the packet to a given visit, they will be asked to bring their packet to the following session. Participants will be asked to either a) complete the measures in person at the current lab visit, or b) to schedule a brief meeting with a research team member via Zoom or

Webex in order to complete the questions (depending on scheduling and availability). These approaches will be used in specific circumstances if needed (e.g., if the participants forgets to complete the daily assessments altogether, or will be unable to bring it to the next session).

d) Weekly Laboratory Visits.

Medication administration visits will occur at either the Eastowne Clinical Research Unit (CRU) or the CTRC. Following the baseline (Week 0) visit, participants will visit CRU or UNC Hospital/CTRC once weekly (Weeks 1-9), for subsequent medication or placebo doses, and for a final follow-up/discharge visit at Week 10. (In addition, the study physician may request an additional follow-up lab visit(s) at any point in the study to monitor side effects.) In addition, to accommodate events such as missed lab visits or scheduling difficulties, assessment procedures at a given weekly visit (e.g., questionnaires, lab tests) may be conducted within a 7-day window of the original target date, as necessary, to avoid unnecessary missing data. In addition to medication/placebo administration, follow-up visits will include 1) vitals, weight measurements, and a brief side-effects assessment, 2) a finger-stick glucose test (or fasting blood draw depending on the week, see study flow chart), 3) a brief calendar-based self-report assessment of interim alcohol and cigarette use (TLFB), and 4) a repeat CO measurement. These procedures are expected to take roughly 30-60 minutes in total, and will be conducted by a trained study team member and/or a member of the CRU/CTRC team (e.g., RN). All side effects reports, labs, and other medical test results (e.g., glucose tests) will be uploaded in Epic (e.g., lab tests) or sent electronically (e.g., self-reported medication changes) for review by the study physician. If side effects or lab results suggest that further assessment is indicated, a member of the medical team will advise the study team (and if necessary, contact the participant) to provide direction. At the Week 4, Week 8, and final discharge visits, participants will be asked to arrive fasting to allow for a blood draw for follow-up lab assays (identical to those described in the baseline procedures above). As with the baseline sample, a portion of each sample will be stored for follow-up analyses (e.g., nicotine metabolites).

e) Alcohol Sessions.

To evaluate effects of medication phase on laboratory measures of alcohol response and self-administration, participants will be asked to complete four laboratory visits over the course of the study. Two visits will occur between the Week 0 and 1 visits (baseline phase), and two will occur between the Week 8 and 9 visits (0.5 mg phase). Minor scheduling deviations will be accommodated as needed, provided that the participant has completed at least three weeks of the .5mg phase at the time of the second set of laboratory sessions.

During each of these sessions participants will be asked to report to the lab having received instructions to abstain from alcohol and recreational drugs for 24h, and not to consume food for 4 hours prior to arrival. Participants will also be advised not to drive to these study appointments. Participants will also be asked to schedule a smoking break just prior to arrival (to ensure that participants are not nicotine-deprived during testing) [16]. Upon arrival participants will provide breathalyzer and expired CO readings, as well as measures of body weight and vitals (HR, BP) and a finger-stick blood glucose test. Participants will also receive the Short Alcohol Withdrawal Scale (SAWS) to ensure that no significant withdrawal symptoms are present. Evidence of withdrawal symptoms will require a consultation with an addiction medicine physician (Dr. Tow or Dr. Jordan) prior to proceeding. (We except a low likelihood that this will happen because participants with

severe AUD or history of withdrawal symptoms will be excluded). Female participants will also be asked to repeat the urine pregnancy screen at each alcohol session.

In the interim, participants will complete self-report questionnaires (e.g., Timeline Follow-Back to assess recent substance use). Participants will be seated in a comfortable recliner chair for the alcohol administration procedures. Participants will first complete baseline physiological readings (systolic and diastolic blood pressure, heart rate), and baseline measurements of self-report questionnaires (see below). Next, participants will complete one of two procedures (alcohol challenge, alcohol self-administration), dependent on the visit week. Specifically, the first alcohol challenge session (AC1) and the first self-administration session (SA1) will be scheduled to take place between Week 0 and 1. The second alcohol challenge session (AC2) and the first self-administration session (SA2) will be scheduled to take place between Week 8 and 9 (0.5mg treatment phase), allowing for minor scheduling adjustments as noted above. Prior to the self-administration session only (which is longer in duration), participants will consume a standardized, isocaloric meal in advance of the alcohol self-administration procedures, with a standardized wait time between completion of the meal and onset of beverage consumption. The two session types will be scheduled with at least one interim day scheduled between sessions when possible. For both sessions, participants will be instructed to expect to remain in the lab for a full day (e.g., 8 hours).

Alcohol Challenge Sessions (AC1, AC2)
The AC sessions are designed to generate data to estimate within-person changes in acute responses to alcohol as a function of medication phase (baseline vs. 0.5mg). Following baseline assessments (described above), participants will be asked to consume a pre-determined dose of alcohol calculated to achieve a target peak breath alcohol concentration (BrAC) of .06g%. This BrAC level reflects a moderate alcohol dose. Individualized doses will be calculated using established formulas [83] that account for determinants of total body water (e.g., height, weight, sex, age). Beverages will consist of 80-proof vodka combined with a non-alcoholic, sugar-free mixer at a 1:5 ratio. Beverages will be allocated to three equal portions in separate cups. Participants will be asked to consume the beverages at an even pace over 10 minutes. A separate placebo session is not incorporated for the AC sessions, because our primary interest is in examining *within-person* changes in laboratory alcohol responses as a function of medication dose (baseline vs. 0.5mg), and because our primary outcome is self-administration (described below). Although a limitation of this approach is the inability to dissociate expectancy from pharmacological effects in measuring subjective responses to alcohol, increasing the number of sessions to dissociate expectancy effects is not a major aim for this preliminary project.

After completion of the beverages, participants will wait 10 minutes (to allow an absorption period), then will complete an initial breath alcohol concentration (BrAC) reading. BrAC readings will be repeated at 10-minute intervals during the first hour of the session (peak BrAC is anticipated to occur around 30-40 minutes, on average, with variability across subjects). Participants will repeat the battery of self-report questionnaires (outlined below) and physiological assessments at three points on the ascending limb of the blood alcohol curve: upon the first BrAC reading that meets or surpasses .02g%, .04g%, .06g%. To account for the fact that some participants may not reach the .06g% target, the latter assessment will be conducted if/when it appears the participant will not reach the .06g% target (as evidenced by two consecutive descending BrAC readings after surpassing the .05g% mark).

Following completion of the last set of measures (.06g%), participants will then be escorted to a private room to begin the recovery phase, during which they will receive water and will be monitored, and take bathroom breaks as needed. During this time, BrAC measurements will continue every 15 minutes. On the descending limb of the blood alcohol concentration curve, participants will complete follow-up measures and physiological readings at the first BrAC readings that meet or fall below .04g% and .02g%. Prior to discharge, participants will be asked to provide an additional blood glucose sample (via fingerpick) to assess any differential changes in glucose levels following alcohol administration as a function of medication phase. Participants will also complete the Food Purchase Task to assess motivation for food following alcohol administration. After this point, participants will be provided with a lunch and snacks to consume during the recovery period. Participants will be compensated and discharged once BrAC reading reaches .00g% (to allow for estimating alcohol elimination rate across sessions AC1 and AC2 as a function of medication group).

Subjective Measures. At baseline (prior to alcohol ingestion) and each subsequent assessment point noted above, participants will complete several measures. Measures of subjective alcohol effects will include the Biphasic Alcohol Effects Scale (BAES) [84], the Alcohol Urge Questionnaire (AUQ) [85] and Drug Effects Questionnaire (DEQ) [86]; the latter will be supplemented with additional items to assess potential effects related to medication (e.g., "tired", "confused", "nauseous", "dizzy", "sick", "drunk/intoxicated") [16]. The Alcohol Purchase Task [45] Cigarette Purchase Task [87] Food Purchase Task [78] and Monetary Choice Questionnaire [76]will be also administered at the corresponding intervals noted above. Changes in mood will be assessed using the Profile of Mood States – Short Form (POMS-SF) [88]. During the recovery period, participants will complete another battery (all measures noted above) once reaching .04g% and .02g% on the descending slope of the blood alcohol curve.

Alcohol Self-Administration Sessions (SA1, SA2).

Medication effects on laboratory alcohol consumption will be examined using a validated oral self-administration procedure. Pre-session instructions and arrival procedures/tests will be identical to those noted above. After completing baseline physiological and baseline questionnaires, participants will be presented with their preferred beverage (beer, wine, or mixed drink). Participants will elect when to begin drinking and will then complete a 2-hour *ad libitum* self-administration period. Participants will have access to their beverage at a volume calculated (based on the person's sex, weight, height, and age using established formulas) intended not to exceed a maximum predicted peak blood alcohol concentration .12g% over the consumption period of two hours (should the participant consume all of the beverages). Participants will be instructed to consume the amount of alcohol they wish (i.e., they are not required to drink all of the beverages). Participants will be seated in a comfortable chair/sofa and will view a neutral video (e.g., nature documentary) during the session. Beverages will be presented to the participant at the onset of the two-hour period, along with ice if requested. Participants will repeat rounds of self-report assessments (described above) at 30-minute intervals during the *ad libitum* phase (i.e., at 60, 90, and 120 min. after the end of the priming phase), with physiological assessments and BrAC readings taken at these intervals (or more frequently if indicated). The primary outcomes for this session are peak BrAC and volume of alcohol consumed.

Participants will then be escorted to a private room for the recovery period. Assessments during the recovery period will be identical to those described above. Participants will be monitored until being discharged when BrAC declines below .03% for two consecutive readings, consistent with NIAAA recommended guidelines. [52] As noted, participants will be instructed not to drive, and will be asked to arrange for a ride home from the study site. If a participant cannot arrange a ride home, they will be provided with reimbursement for a ride home via a rideshare service (e.g., Uber or Lyft) up to $25. If a participant reports having driven to a session, they will be required to remain at the site until BrAC is

measured at 0mg% for two consecutive readings. At alcohol sessions, participants may also be provided with a wristband transdermal alcohol monitor (BACTrack Skyn) to wear during the session, which will provide a continuous readout of estimated alcohol concentration (measured as total alcohol concentration in sweat). Estimated transdermal alcohol concentration will not be a primary outcome - as it is not an accurate measure of blood alcohol concentration - but will be collected for purposes of secondary data analyses. Specifically, we will record readouts from the transdermal device at regular intervals to compare to BrAC readings, which will inform our use of this device in future studies. Because we will only have one transdermal device available, it may not be used in all sessions (depending on availability).

List of Study Assessment Measures (See Section 1.3 for assessment time points)

1. Demographics form
2. Timeline Follow-Back (TLFB)
3. Structured Clinical Interview for DSM-5 Mental Disorders (select modules)
4. MINI Neuropsychiatric Interview (select modules)
5. Sweet Taste Preference Test
6. Alcohol Use Disorder Identification Test (AUDIT)
7. Self-Report of the Effects of Alcohol Questionnaire (SRE)
8. Center for Epidemiological Studies Depression Scale (CES-D)
9. Toronto Alexithymia Scale (TAS-20)
10. Barratt Impulsivity Scale (BIS-11)
11. UPPS Impulsive Behavior Scale (UPPS)
12. Reward Probability Index – Environmental Reward Observation Scale (RPI-EROS)
13. Impaired Control Scale (ICS)
14. Fagerstrom Test for Nicotine Dependence (FTND)
15. Nicotine Dependence Syndrome Scale (NDSS)
16. Penn Alcohol Craving Scale (PACS)
17. Tiffany Questionnaire of Smoking Urges (TQSU)
18. Wisconsin Smoking Withdrawal Scale (WSWS)
19. Wisconsin Inventory of Smoking Dependence Motives (WISDM)
20. Alcohol and Smoking Contemplation Ladder
21. Three Factor Eating Questionnaire (TFEQ)
22. Reward-Based Eating Drive Scale (R-BEDS)
23. Control of Eating Questionnaire (CEQ)
24. Simplified Nutritional Appetite Questionnaire (SNAQ)
25. Monetary Choice Questionnaire (MCQ)
26. Cigarette Abstinence Self-Efficacy (CASE)
27. Alcohol Abstinence Self-Efficacy (AASE)
28. Inventory of Drinking Situations (IDS)
29. Biphasic Alcohol Effects Scale (BAES)
30. Alcohol Urge Questionnaire (AUQ)
31. Drug Effects Questionnaire (DEQ)
32. Alcohol Purchase Task (APT)
33. Cigarette Purchase Task (CPT)
34. Food Purchase Task (FPT)
35. Profile of Mood States – Short Form (POMS-SF)
36. Systematic Assessment for Treatment Emergent Effects (SAFTEE)
37. Prediction of Alcohol Withdrawal Severity Scale (PAWS)

38. Short Alcohol Withdrawal Scale (SAWS)
39. Obsessive Compulsive Drinking Scale
40. Graded Pain Scale

Analysis schedule. Analyses of side effect frequency and AE/SAE frequency will be conducted prior to scheduled DSMB meetings. Other analyses will be conducted following the completion of the trial, or as otherwise required to fulfill DSMB requests or other reporting requirements.

4.2 SCIENTIFIC RATIONALE FOR STUDY DESIGN

This short-term trial is intended to produce initial data on the effects of semaglutide in non-treatment-seeking participants with AUD, with an emphasis on human laboratory outcomes. The dose escalation schedule is based on the standard clinical regimen, which is intended to minimize side effects common to GLP-1 agonists (i.e., nausea). Participants in the placebo group will complete an identical sequence while receiving sham subcutaneous injections (a small needle stick using a micro-needle of the same size, without an injection). This placebo condition is selected due to the absence of an available matching placebo cartridge from the manufacturer (see section 4.3). All participants will complete laboratory alcohol administration sessions at two time points, corresponding with the baseline and 0.5mg phases. This design allow for within-subjects analyses for laboratory outcomes that are best examined at the within-subject level (owing to high inter-individual variability in alcohol pharmacokinetics). Overall, this design is intended to maximize feasibility and sample size while also accommodating the extended dosage titration period required of GLP-1 agonists. Findings from this trial will be used to justify a larger, placebo-controlled Phase II clinical trial application via the R01 mechanism.

4.3 JUSTIFICATION FOR DOSE

Semaglutide (Ozempic®) will be administered according to the FDA-approved schedule, which begins with a starting dose of 0.25mg (for titration purposes) once per week for four weeks. At week five, dosage is increased to 0.5mg/week (the initial first maintenance dose) for a subsequent four weeks, at which point dosage can be increased further (1.0mg) if indicated (and presuming that the 0.5mg dose has been tolerated). The intent for this initial trial is to mimic the typical clinical scenario over the first several weeks of medication titration. Although primary analyses will focus on the 0.5mg dosage, participants will receive one week of 1.0mg treatment at the conclusion of the 0.5mg phase, in order to collect initial data on side effects and naturalistic drinking/cigarette use at this dosage in the current population. Participants will be monitored carefully and will not be escalated to the next dose if experiencing persistent side effects indicative of poor tolerability.

Given the lack of a true placebo (i.e., no manufacturer-developed placebo Ozempic® pen/cartridge), this study will use a placebo condition that consists of weekly sham doses. Sham doses will involve a brief needle prick using a very small (e.g., 32 gauge) needle. These procedures are described in more detail later in this protocol. By necessity, medical staff administering the medication cannot be blind to condition. However, other experimenters and outcomes assessors will remain blind to condition. Participants will be instructed during the informed consent and study orientation that they have a 50/50 chance of being randomized to medication and placebo groups.

4.4 END OF STUDY DEFINITION

A participant is considered to have completed the study if he or she has completed all phases of the study including the last visit shown in the Schedule of Activities (SoA), Section 1.3. The end of the study is defined as completion of the last visit shown in the SoA in the trial overall.

5 STUDY POPULATION

5.1 INCLUSION CRITERIA

In order to be eligible to participate in this study, an individual must meet the following criteria:

- Age 21-65
- Meeting DSM-5 criteria for current (past year) AUD, with between 2-7 symptoms endorsed, and NIAAA criteria for current at-risk drinking (i.e., >7/14 drinks in one week for women/men, with at least two episodes of 4+/5+ drinks in the past 30 days)
- Regular smoker, defined as reporting smoking 1+ time smoking cigarettes per week, on average, over the past month (avg cigarettes per week ≥ 1)
- Willingness/availability to take study medication and complete study procedures, including attending weekly visits for medication administration, side effect assessments, and glucose monitoring
- Willingness to complete laboratory sessions involving alcohol administration
- Ability to communicate and read in English

5.2 EXCLUSION CRITERIA

- Regular use of electronic nicotine delivery systems (ENDs; vaping), cigars, chewing tobacco or snuff, based on at least weekly use in the past 30 days
- Past 30-day use of nicotine replacement therapies/products
- Reporting past 30-day use of illicit drugs other than cannabis at baseline, or having a positive toxicology screen for illicit drugs other than cannabis at baseline
- Meeting past-year criteria for a substance use disorder (with the exception of alcohol, tobacco or mild cannabis use disorder)
- Current engagement in alcohol or smoking cessation treatments, or currently engaged in intentional efforts to quit alcohol use
- Past 30-day use of: Sincalide, Sulfonylureas, insulin and insulin products or other medications that may interact with semaglutide;, or weight control medications
- Prior use of semaglutide or other GLP-1 agonists
- Known or suspected hypersensitivity to study medication or related products
- Lifetime diagnosis of severe mental illness (including schizophrenia and bipolar disorder)
- History of suicide attempt, or recent (past 30 day) suicidal ideation, or psychiatric hospitalization in the last 6 months

- Current significant medical or neurological illness (based on self-report or medical record) including severe hepatic impairment or cirrhosis, impaired renal function (eGFR <50ml/min), acute or chronic pancreatitis, gastroparesis, gallbladder disease or cholelithiasis, other severe gastrointestinal disease, heart failure, coronary artery disease, stroke, seizure disorder, or other medical condition that poses a risk for the medication or alcohol administration components of the study (as determined by the MD)
- A personal or family history of medullary thyroid cancer or multiple endocrine neoplasia 2A or 2B
- Calcitonin greater than or equal to 50 ng/L
- Uncontrolled thyroid disease TSH >6.0 mIU/L or <0.4 mIU/L at screening
- History of major surgical procedures involving the stomach potentially affecting absorption of trial product (e.g., subtotal and total gastrectomy, sleeve gastrectomy, gastric bypass surgery)
- History of Type 1 or Type 2 diabetes, or HbA1c >6.5% measured at screening
- History of diabetic retinopathy, proliferative retinopathy, or maculopathy
- History of diabetic ketoacidosis
- History or presence of malignant neoplasms within the last 5 years (except basal and squamous cell skin cancer and carcinoma in situ)
- Currently nursing, pregnant, anticipating pregnancy in the next 6 months, or not using a highly effective contraceptive method as judged by the MD, and defined as:
    a. combined (estrogen and progestogen containing) hormonal contraception associated with inhibition of ovulation (oral, intravaginal, transdermal)
    b. progestogen-only hormonal contraception associated with inhibition of ovulation (oral, injectable, implantable)
    c. intrauterine device
    d. intrauterine hormone-releasing system
    e. bilateral tubal occlusion
    f. vasectomized partner
    g. sexual abstinence
- Elevation of serum lipase, amylase, direct (conjugated) bilirubin, or alkaline phosphatase (ALP), ALT, or AST) more than 3X the upper limit of normal on baseline bloodwork
- Baseline body mass index (BMI) <25kg/m2 or >35kg/m2
- Uncontrolled hypertension or systolic BP >180 mmHg and/or diastolic BP >105 mmHg, averaged from three measurements
- Plans for travel outside of the local area in the upcoming 12 weeks that would interfere with lab visits during the study period (or other logistic factors that would make it difficult to commit to entire duration of study)

5.3 LIFESTYLE CONSIDERATIONS

Specific lifestyle instructions will include:

- Refrain from alcohol consumption or other drug use for 24 hours prior to alcohol administration sessions
- Arrive fasting at sessions scheduled for blood draws as needed
- Refrain from consuming food for 4 hours prior to alcohol sessions. Participants will receive a standardized snack on arrival for alcohol sessions.)
- Refrain from driving to the lab on the day of alcohol sessions. (Participants will be asked to arrange for transportation, or will be provided with transportation home via taxi or Uber)
- Refrain from operating other vehicles or machinery for the duration of day upon discharge from alcohol sessions
- Women participating in the study must report being willing to use a reliable birth control method during the trial (if sexually active), and must report no plans for pregnancy in the next 6 months.
- Participants will also understand that they may be excluded if they test positive for drugs other than cannabis during the time of scheduled drug screens (repeat drug tests will take place during weeks 4 and 8).

5.4 SCREEN FAILURES

Screen failures are defined as participants who consent to participate in the clinical trial but are not subsequently randomly assigned to the study intervention or entered in the study. A minimal set of screen failure information is required to ensure transparent reporting of screen failure participants, to meet the Consolidated Standards of Reporting Trials (CONSORT) publishing requirements and to respond to queries from regulatory authorities. Minimal information includes demography, screen failure details, eligibility criteria, and any serious adverse event (SAE).

Examples of screen failures would include participants excluded following baseline laboratory work, or those excluded based on a change in treatment-seeking status at the time of the baseline assessment. Individuals who do not meet the criteria for participation in this trial (screen failure) because of a specific modifiable factor (e.g., upcoming travel) may be rescreened at a later date. Rescreened participants will be assigned the same participant number as for the initial screening. Data pertaining to screening failures will be tabulated in an anonymized fashion.

5.5 STRATEGIES FOR RECRUITMENT AND RETENTION

Participants will be recruited from the community through advertisements, which may include ads in local online or print newspapers, advertisements in buses, print flyers placed in community or hospital settings, business cards, campus listservs, and online postings (e.g. Craigslist (ads will not be placed in employment section)), and social media ads (Facebook, Twitter, Instagram) that direct potential participants toward a study website/landing page (hosted on Research for Me). Participants may also be recruited from local medical clinics (e.g., primary care clinics) that grant approval for assisting with recruitment (e.g., by placing study advertisements in clinics). Flyers and online advertisements will include study contact information and a web link and/or QR code leading to the landing page.

In addition to these “passive” methods of recruitment, we will also include “active” recruitment methods. These methods will include members of the study team setting up recruitment booths and

passing out business cards at public events (e.g., festivals) or public venues. Once initial contact with participants is made (e.g., via email or in person), research staff will schedule the participant for a phone call to explain the study and conduct a screening interview.

Participants will receive compensation. The payments will be allocated as follows (please see 1.2 for a summary of all scheduled sessions):

- $50 for completing the consent/screening/baseline sequence (Week 0)
- $75 for alcohol challenge session 1 (To be scheduled between Weeks 0-1)
- $75 for alcohol self-administration session 1 (To be scheduled between Weeks 0-1)
- $100 for alcohol challenge session 2 (To be scheduled between Weeks 9-10)
- $100 for alcohol self-administration session 2 (To be scheduled between Weeks 9-10)
- $30 per visit for each follow-up/medication administration visit during Weeks 1-10 (10 visits x$30 per visit = $300)
- Up to $150 for completing and returning daily questionnaires over the study period ($15 per weekly set of surveys x 10 weekly visits), which includes the week following the 1.0mg dose, to be paid at weekly medication visits)
- A $50 bonus for completing at least 9 of 10 weekly assessments
- Participants will receive a final bonus of $100 for completing the study through Week 10 (to be paid at the final discharge visit).

Therefore, the total maximum compensation over 10 weeks for study visits is $1000. Compensation for each pair of the alcohol sessions will occur at the end of the second of the session pair. The final payment (including bonuses) will be paid at the Week 10 discharge visit. Overall, these payments are structured to maximize retention in the study (given the long dose escalation period and time-intensive laboratory sessions). Additionally, an incentive-based structure that includes weekly incentives is important to reinforce engagement with daily surveys, and to minimize missing data. Participants will be updated at each visit with their progress toward the bonus incentives for adherence (e.g., weekly bonus of $10 and progress toward final adherence bonuses). In the event participants are not able to pick their final payments in person, we will offer the option of issuing the final payment via electronic gift card to the participant’s email address, with their permission.

We anticipate phone screening up to 400 individuals and randomizing up to 48 participants to medication or placebo conditions, in order to obtain a final sample of at least 36 eligible participants who complete the full course of the 0.5mg phase (allowing for 25% attrition). If attrition is higher than anticipated, we will submit an amendment to enroll additional participants to arrive at a final sample of at least 36 participants who complete study procedures through the end of the 0.5mg dosage phase (i.e., through the end of Week 9).

6 STUDY INTERVENTION

6.1 STUDY INTERVENTION(S) ADMINISTRATION

6.1.1 STUDY INTERVENTION DESCRIPTION

Semaglutide is a relatively new (approved in 2017) GLP-1 receptor agonist. A new oral formulation of

semaglutide was approved by the FDA in 2019, making semaglutide the first drug in its class to be available in both subcutaneous and oral formulations. GLP-1 agonist medications were designed to mimic the function of the endogenous GLP-1 peptide, with the benefit of a much longer half-life than endogenous GLP-1. Based on their glucose-lowering and appetite-reducing properties, GLP-1 agonists were developed as anti-diabetic therapies. Several GLP-1 agonists (e.g., exenatide, liraglutide, lixisenatide, albiglutide, dulaglutide and semaglutide,) are now in widespread use as treatments for type II diabetes (T2D); these medications have also proven efficacious for weight loss, reducing glycated hemoglobin (HbA1c), and reducing risk for some cardiovascular events [29]. Although the primary indication of GLP-1 agonists is T2D, these therapies are increasingly being studied for other indications in non-diabetic patients, including obesity/weight management. In June 2021, the FDA approved semaglutide at a higher dosage (2.4mg/week) for the indication of weight management, under the brand name Wegovy.

6.1.2 DOSING AND ADMINISTRATION

Semaglutide will be administered according to the FDA-approved schedule, which begins with a starting dose of 0.25mg once per week for four weeks. At week five, dosage is increased to 0.5mg for a subsequent four weeks (initial maintenance dose), after which point dosage can be increased further (to 1.0mg), presuming the medication is well tolerated.

Although semaglutide can be self-administered in the home setting, medication and placebo administration will be delivered by a research nurse or other medical team member at Eastowne CRU or CTRC in order to execute the placebo manipulation and ensure adherence to medication. This approach also allows us to collect safety outcomes on a regular basis, further ensuring that participants will be monitored carefully. Semaglutide is administered subcutaneously (in the abdomen, thigh, or upper arm). Doses are delivered via the Ozempic® pen, a cartridge-based device that calibrates medication delivery based on the desired dose. An extremely small needle (4mm, 32-gauge needle – the size of 2 human hairs) is used to deposit the medication subcutaneously. Because each pen contains 2mg of medication, each participant in the medication group will be allocated one pen to cover the first six weeks (i.e., 0.25mg/week for 4 weeks, followed by 0.5mg/week for 2 weeks), and a second pen for that will supply medication for the remaining three weeks (2 weeks at 0.5mg, and 1 week at 1.0mg). This schedule results in 9 medication administration sessions. At the week 9 visit, the 1.0mg dose will be delivered as 2 x 0.5 mg doses given in succession. This is because the medication pens being used for this study deliver dosages of 0.25 and 0.5mg, and participants are not being maintained on the 1.0mg dose after week 9. This timeframe also allows sufficient time to reach steady-state plasma concentration levels (achieved within 4-5 weeks of initiating the starting dose).

At the present time, we do not have access to a manufacturer-developed placebo (the medication relies on the proprietary Ozempic® pen for delivery). Therefore, this study will use weekly sham injections for the placebo group. Sham doses will involve a brief needle prick using a very small (e.g., 32 gauge) needle, identical to the one used in the Ozempic® pen (NovoFine), or a needle of similar gauge (e.g., an insulin needle). Given the extremely small volume of medication delivered in the treatment condition, we will not utilize saline or another inert substance for the placebo administration. During medication and placebo administration, participants' view will be shielded from the injection site to prevent unblinding. Medication will be delivered in the upper arm, abdomen, or thigh. Given the extremely small volume of the injections, we will not utilize saline or another inert substance for the placebo administration; rather, the sham doses will consist of briefly inserting the micro-needle.

Due to the nature of the placebo condition, it will not be possible to blind the staff RNs administering the medication. However, we will provide training to study staff to minimize the likelihood of contaminating the blinding procedures. We will also develop procedures to minimize contact between the RNs and outcome assessors. All other staff members (including research assistants and outcomes assessors) will remain blind to condition, unless unblinding is necessary (e.g., due to an adverse event).

6.2 PREPARATION/HANDLING/STORAGE/ACCOUNTABILITY

6.2.1 ACQUISITION AND ACCOUNTABILITY

The team will collaborate with IDS to source the medication (and NovoFine or insulin needles for the placebo condition), which will be sourced and stored at the IDS pharmacy prior to dispensing. Participants assigned to medication will be prescribed one Ozempic® pen following randomization. As per IDS requirements, in between study visits participants will be given their assigned medication pen and asked to bring their pen back to each follow-up visit. In between visits, pens will be placed in a small plastic container and then encased opaque, tamper-proof bag in order to prevent unblinding. (For participants in the placebo condition, a plastic object of roughly equal size and weight will be placed in the plastic container and medication bag.) Participants will be instructed not to open the bag in between visits. A refill pen will be prescribed prior to the Week 7 visit, once the doses from the first pen have been allocated. Any unused doses from individual pens (e.g., in the event of participant drop-out) will be discarded/destroyed by IDS, as will any pens that expire during the course of the trial. In the event a pen expires before a participant completes the study, a replacement pen will be prescribed at the discretion of the study physicians.

6.2.2 FORMULATION, APPEARANCE, PACKAGING, AND LABELING

Ozempic® is manufactured by Novo Nordisk. As described above, the medication is dispensed from a proprietary device, the Ozempic® pen. Each pen contains 2mg of medication, requiring that each participant is prescribed two pens over the course of the study. A full description and visuals can be found at the following site:
https://www.Ozempic®pro.com/how-to-prescribe/Ozempic®-pen.html

The *placebo group* will receive sham injections that involve a brief needle-prick without dispensing a substance. The needles used for the sham injections will be the NovoFine 32G needle, the same needle in the Ozempic® pen (https://www.novoneedles.com/novofine.html) or a comparable needle of a similar gauge (e.g., an insulin needle). In no case will any fluid be injected during placebo sessions.
To mimic the active medication administration, the medical staff member will insert the microneedle briefly and then remove it while the participant's view is shielded.

6.2.3 PRODUCT STORAGE AND STABILITY

Pens will be dispensed from IDS. For drug dispensing visits at CRU (Eastowne), scheduled for Weeks 1 and 7, pens will be delivered the day of the visit by courier. Ozempic pens will be kept in the participant's possession (sealed in a tamper-proof bag) between visits. According to the manufacturer, following the first use, the Ozempic® pen can be stored for up to 56 days at room temperature (59 to 86 F) or refrigerated at 36 to 46 F. As per our plan developed in collaboration with IDS, participants will be instructed to keep the medication bag at room temperature for simplicity. Each participant's pen will be

labeled with the study ID number and pens will not be shared across participants. Placebo needles will remain packaged until use, and will be stored at room temperature in a secure location.

6.2.4 PREPARATION

Semaglutide does not require advance preparation; cartridges are pre-filled and doses are calibrated using the pen device. The RN or medical staff member will select the correct dosage and administer the medication as per manufacturer guidelines.

6.3 MEASURES TO MINIMIZE BIAS: RANDOMIZATION AND BLINDING

Following the baseline assessment, participants will be randomized to medication or placebo conditions at a 1:1 ratio by a member of the IDS team using a pre-determined randomization scheme. Medication and placebo administration will be carried out by a staff RN at CRU or CTRC. Due to the nature of the placebo condition, it will not be possible to blind the RNs to condition. However, all other staff members will remain blind to condition, and the staff members administering medication will be trained in protecting the blind (e.g., redirecting the conversation if the participant comments about medication condition; minimizing contact with experimenters.) To maintain blinding of participants, participants' view will be shielded and they will be asked to face away from the injection site during all placebo and medication administrations. Aside from the staff members administering medication, no study team members interacting with participants will be aware of participants' assignments. At the stage of interim analyses (e.g., in preparing safety reports for DSMB meetings) and final analyses, the study statistician will be provided with the assignment log. None of the study personnel involved in recruitment or enrollment will have knowledge of the details of the randomization table that will be used by the IDS pharmacy.

At the completion of the trial, participants will be provided with a questionnaire asking about perceived medication assignment (to assess the success of maintaining the blind). Due to common side effects of semaglutide (in particular nausea), we do not expect blinding to be successful in all cases.

6.4 STUDY INTERVENTION COMPLIANCE

Medication will be administered by CRU or CTRC staff to assure compliance and reduce potential sources of non-adherence. A missed session will constitute a missed medication dose. As per manufacturer directions, any missed doses/appointments will be corrected by asking the participant to come in for the dose as soon as possible within 5 days of the missed dose. After the 5-day mark has passed, the dose will be skipped, and the participant will resume with the next scheduled dose. Study medication logs will serve as the primary source document for tracking medication and placebo administration. Participants will be coded as non-adherent if they miss more than 2 scheduled doses between Weeks 1-9. In the event of missed medication doses or missed appointments, the physician will have the discretion to adjust the appointment date (within the manufacturer or standard practice guidelines), or to prescribe a replacement pen if needed to accommodate the target medication schedule.

6.5 CONCOMITANT THERAPY

Participants may be taking medications for other conditions (provided those treatments are not included in the list of exclusion criteria). For this protocol, a prescription medication is defined as a medication that can be prescribed only by a properly authorized/licensed clinician. Medications to be reported in the Case Report Form (CRF) are concomitant prescription medications, over-the-counter medications and supplements. Changes to medication status or other treatments will be assessed at each in-person visit, with any changes reviewed by a study physician. Should a concomitant therapy preclude further participation for safety or scientific reasons (for instance, if a participant starts a therapy that constitutes an exclusion factor), this issue will be discussed by the study leads and the participant will be excused from further participation, if required to ensure safety. By definition, participants will not be seeking treatment for alcohol use or smoking cessation. If a participant makes the decision to seek treatment for alcohol or smoking during the study, s/he will be referred to appropriate treatment resources and excused from further participation.

6.5.1 RESCUE MEDICINE

Not applicable

7 STUDY INTERVENTION DISCONTINUATION AND PARTICIPANT DISCONTINUATION/WITHDRAWAL

7.1 DISCONTINUATION OF STUDY INTERVENTION

Study discontinuation: The study may be halted if deemed necessary by the lead investigators, DSMB, or IRB. Examples of reasons for study discontinuation include unexpectedly high frequency of serious adverse events, or significant increases in alcohol use during treatment, leading to the conclusion that the medication is not safe in this population. In addition to the participant discontinuation rules noted below, the following study stopping rules will trigger halting the study pending a DSMB evaluation: 1) Occurrence of any hospitalization or any SAE rated at Grade 4-5 (CTCAE) in a medication-treated participant (and deemed likely to be medication-attributable); 2) Documentation of SAEs in >3 medication-treated participants that are deemed likely attributable to medication; 3) Removal of >5 subjects due to adverse events deemed attributable to study-related procedures, inclusive of SAE and/or medical stopping rules (e.g., weight, alcohol consumption, disease progression), as defined in 7.2 below. In order to classify events as medication-attributable, the PI will request that IDS breaks the blind and provides a file containing condition assignment to the study physician(s). If any of the aforementioned events trigger a DSMB meeting, the full randomization table will be provided to a statistician, so that group differences can be fully evaluated at the meeting. Given the safety profile of semaglutide for other clinical conditions, as well as preclinical evidence that GLP-1 receptor agonists reduce drug intake, these scenarios are expected to be unlikely.

7.2 PARTICIPANT DISCONTINUATION/WITHDRAWAL FROM THE STUDY

Participants are free to withdraw from participation in the study at any time upon request. The investigators may discontinue or withdraw a participant from the study if clinically or otherwise indicated. Examples of reasons removing a participant include:

- Significant study intervention non-compliance
- Any clinical adverse event (AE), laboratory abnormality, or other medical condition or situation that indicates that continued participation in the study would be unsafe
- Pregnancy
- Diagnosis of significant disease or disease progression which requires discontinuation of the study intervention
- The participant meets an exclusion criterion (either newly developed or not previously recognized) that precludes further study participation
- Participant relocates from the local area, or is otherwise unable to attend visits
- Significant or persistent side effects that pose significant discomfort or preclude escalation to the 0.5mg dose (e.g., persistent significant nausea, or hypoglycemia, as indicated by repeated fasting glucose values below normal 54 mg/dL
- Changes in lab tests or clinical status from baseline that render the participant ineligible to continue in the judgement of the physician(s) (e.g., elevated liver enzymes, elevated serum amylase or lipase levels, weight loss as defined below)
- Excessive weight loss (defined as loss exceeding 5% of baseline weight or BMI < 20)
- A change in treatment-seeking status (i.e., the participant expresses a desire to enter treatment for alcohol or smoking cessation).
- A significant increase in alcohol consumption, as evidenced by a >50% increase in drinks per week (relative to baseline) lasting more than two weeks. Drinks per week in the 30 days preceding the Week 0 visit (measured by the TLFB) will serve as the baseline measure.
- Evidence of significant alcohol withdrawal symptoms
- Any other change in medical status or side effects that is significant enough to warrant removal in the judgement of the study physicians

If a participant exhibits changes in symptoms or clinical status that warrant potential removal, the study physicians will confer and render a decision as to whether to withdraw the participant. With respect to dosage increase, if a participant's side effects profile or laboratory tests during the initial 4-week phase (.25mg) indicate that s/he is unable to safely proceed to the 0.5mg dose, the participant may be removed from the study at the discretion of the physicians. If lab tests or side effects during the standard maintenance dose of 0.5mg suggest that a participant is unlikely to tolerate a higher dose (1mg), they may be excluded from the 1.0mg dose escalation at the discretion of the physicians. In this case, participants will be given the option of one additional week at the standard therapeutic dose of 0.5mg (i.e., highest tolerated dose). Reasons for participant discontinuation or withdrawal from the study will be recorded on the Case Report Form (CRF). Participants who sign the informed consent form but do not receive the study intervention (e.g., fail to attend the Week 1 visit for randomization) may be replaced. Participants who sign the informed consent form, and are randomized and receive the study intervention, and subsequently withdraw, or are withdrawn or discontinued from the study, may be replaced.

7.3 LOST TO FOLLOW-UP

A participant will be considered lost to follow-up if he or she fails to return for 2 consecutive scheduled medication visits and is unable to confirm attendance at the following visit (or is unable to be contacted by the study staff

The following actions will be taken if a participant fails to return to a required study visit:

- The team will attempt to contact the participant and reschedule the missed visit within one week, and counsel the participant on the importance of maintaining the assigned visit schedule and ascertain if the participant wishes to and/or should continue in the study.
- The team will attempt to contact the individuals that the participant had listed on the Locator Form at baseline (maximum of three attempted contacts per individual).
- Before a participant is deemed lost to follow-up, the investigator or designee will make every effort to regain contact with the participant (where possible, up to 3 telephone messages, up to 3 email messages, and if necessary, a letter to the participant's last known mailing address or local equivalent methods). These contact attempts will be documented in the participant's medical record or study file.
- Should the participant continue to be unreachable, he or she will be considered to have withdrawn from the study with a primary reason of lost to follow-up.

8 STUDY ASSESSMENTS AND PROCEDURES

8.1 EFFICACY ASSESSMENTS

The efficacy assessments/outcomes are noted in summary form in section 3. The ascertainment of these outcomes is described in further detail in section 4 (study design). Briefly, the endpoints are human laboratory measures of alcohol consumption and alcohol response; self-reported alcohol consumption and cigarette use over the course of the trial; and changes in weight, HbA1c, and alcohol elimination.

8.2 SAFETY AND OTHER ASSESSMENTS

Safety and assessments are summarized in detail in the study design section (Section 4.1). Briefly, safety assessments include a baseline assessment, body weight, bloodwork, vitals signs, and clinical interview to assess substance use and psychiatric symptoms. Follow-up medical visits will include repeat assessments of bloodwork and scheduled time points, as well as follow-up measurements of body weight and vitals signs. Medication side effects will be assessed at each visit, and a study team member will contact participants within 2-3 days of each dosage increase to evaluate potential side effects. Other assessments (questionnaires, interviews) are noted in Section 4.1.

Safety precautions during alcohol administration sessions. Our team has considerable experience with alcohol administration procedures. Our procedures follow the NIAAA guidelines for administering alcohol in human studies (https://www.niaaa.nih.gov/research/guidelines-and-resources/administering-alcohol-human-studies). Based on these guidelines, alcohol administration studies with participants who meet criteria for AUD are typically restricted to non-treatment-seeking participants in order to avoid ethical concerns, as will be the approach in this study. Additionally, our approach in this study is to only recruit people who meet criteria for AUD with <8 of out of 11 symptoms endorsed. Regarding the alcohol dose for alcohol challenge sessions, we will use sex- and body weight adjusted formulas [70] to ensure that dosage is calibrated across participants to achieve the target BrAC (.06g%). Participants will

have undergone a medical screen prior to participation, and will be excluded if the study medical staff has reason to believe that alcohol is contraindicated. Participants will receive a BrAC reading on arrival to the alcohol sessions; the session will be rescheduled if the participant presents with a positive BrAC.

Following the completion of alcohol sessions, participants will be escorted to a private room designated for recovery purposes. During alcohol self-administration sessions, participants will only be discharged after their breath alcohol concentration descends below .03g%, which is consistent with NIAAA guidelines. During alcohol challenge sessions, participants will be asked to remain in the lab until BrAC reaches 0mg% (in order to collect BrAC data until alcohol is fully eliminated, for purposes of pharmacokinetic analyses). Participants will also be provided with reimbursement for transportation if they do not drive and are not able to locate assistance with transportation (participants will be advised not to drive to the alcohol sessions). If a participant presents with a positive BrAC, the session will be rescheduled, and the participant will be asked to remain in the lab until it is safe to leave.

To ensure participant safety, at least two members of the study staff will be present for the duration of all alcohol administration sessions. If an urgent medical situation arises at either medication or alcohol visits, a team member will contact the physicians on the team to consult, based on a predetermined sequence (e.g., contact physician A, followed by physician B, then physician C). Additionally, any participants who express a desire to seek treatment during the study will be excused from further participation and provided with alcohol or smoking cessation resources. Co-I Dr. Jordan directs outpatient addiction treatment at UNC-CH.

8.3 ADVERSE EVENTS AND SERIOUS ADVERSE EVENTS

8.3.1 DEFINITION OF ADVERSE EVENTS (AE)

Adverse event means any untoward medical occurrence associated with the use of an intervention in humans, whether or not considered intervention-related (21 CFR 312.32 (a)).

8.3.2 DEFINITION OF SERIOUS ADVERSE EVENTS (SAE)

An adverse event (AE) or suspected adverse reaction is considered “serious” if, in the view of either the investigator or sponsor, it results in any of the following outcomes: death, a life-threatening adverse event, inpatient hospitalization or prolongation of existing hospitalization, a persistent or significant incapacity or substantial disruption of the ability to conduct normal life functions, or a congenital anomaly/birth defect. Important medical events that may not result in death, be life-threatening, or require hospitalization may be considered serious when, based upon appropriate medical judgment, they may jeopardize the participant and may require medical or surgical intervention to prevent one of the outcomes listed in this definition. Examples of such medical events include an adverse reaction requiring intensive treatment in an emergency room or at home.

8.3.3 CLASSIFICATION OF AN ADVERSE EVENT

8.3.3.1 SEVERITY OF EVENT

For adverse events (Aes) not included in the protocol defined grading system, the following guidelines will be used to describe severity.

- **Mild** – Events require minimal or no treatment and do not interfere with the participant's daily activities.
- **Moderate** – Events result in a low level of inconvenience or concern with the therapeutic measures. Moderate events may cause some interference with functioning.
- **Severe** – Events interrupt a participant's usual daily activity and may require systemic drug therapy or other treatment. Severe events are usually potentially life-threatening or incapacitating. Of note, the term "severe" does not necessarily equate to "serious".

8.3.3.2 RELATIONSHIP TO STUDY INTERVENTION

All adverse events (Aes) must have their relationship to study intervention assessed by the clinician who examines and evaluates the participant based on temporal relationship and his/her clinical judgment. The degree of certainty about causality will be graded using the categories below. In a clinical trial, the study product must always be suspect.

- **Related** – The AE is known to occur with the study intervention, there is a reasonable possibility that the study intervention caused the AE, or there is a temporal relationship between the study intervention and event. Reasonable possibility means that there is evidence to suggest a causal relationship between the study intervention and the AE.
- **Not Related** – There is not a reasonable possibility that the administration of the study intervention caused the event, there is no temporal relationship between the study intervention and event onset, or an alternate etiology has been established.

8.3.3.3 EXPECTEDNESS

The study physicians will be responsible for determining whether an adverse event (AE) is expected or unexpected. An AE will be considered unexpected if the nature, severity, or frequency of the event is not consistent with the risk information previously described for the study intervention.

8.3.4 TIME PERIOD AND FREQUENCY FOR EVENT ASSESSMENT AND FOLLOW-UP

The occurrence of an adverse event (AE) or serious adverse event (SAE) may come to the attention of study personnel during study visits and interviews of a study participant presenting for medical care, or upon review by a study monitor. At weekly medication visits, all participants will review a side effects checklist (SAFTEE) with a research assistant, which will then be provided for review by a medical staff member at CTRC. The staff member will begin with an open-ended question about side effects or any other adverse events, followed by a review of specific side effects using the SAFTEE. Any previously reported side effects will be queried to determine any change in status (presence or severity). These weekly visits will be a primary method for identifying side effects. Additionally, a research staff member will contact participants by phone in the week following the initial dose, as well as the week following each dose escalation. Phone calls will be scheduled 2-3 days following these events.

All Aes including local and systemic reactions not meeting the criteria for SAEs will be captured on the appropriate case report form (CRF). Information to be collected includes event description, time of onset, clinician's assessment of severity, relationship to study product (assessed only by those with the training and authority to make a diagnosis), and time of resolution/stabilization of the event. All Aes occurring while on study will be documented appropriately regardless of relationship. All Aes will be followed to adequate resolution.

Any medical condition that is present at the time that the participant is screened will be considered as baseline and not reported as an AE. However, if the study participant's condition deteriorates at any time during the study, it will be recorded as an AE.

Changes in the severity of an AE will be documented to allow an assessment of the duration of the event at each level of severity to be performed. Aes characterized as intermittent require documentation of onset and duration of each episode.

Under the direction of the PI, members of the research team will record all reportable events with start dates occurring any time after informed consent is obtained until 7 (for non-serious Aes) or 30 days (for SAEs) after the last day of study participation. At each study visit, the team member will inquire about the occurrence of AE/SAEs since the last visit. Any such events will be followed for outcome information until resolution or stabilization.

8.3.5 ADVERSE EVENT REPORTING

All project staff will be trained to identify adverse events (Aes), which must be reported to the PI and study physicians within 24 hours. In addition, any unanticipated participant issues will be discussed in weekly staff meetings, which will provide another opportunity to identify Aes.

8.3.6 SERIOUS ADVERSE EVENT REPORTING

Research staff will be trained to report potential Aes or SAEs to the PI immediately. For any potential Aes/SAEs, the study physician will make a determination to distinguish between Aes, SAEs and unanticipated problems and provide judgment about attributions. Management and reporting of Aes, SAEs, and unanticipated problems will be carried out in accordance with Good Clinical Practice (GCP) standards, as outlined by the Food and Drug Administration. Aes will be defined as any negative medical event during the trial (whether or not the event is deemed related to the study medication). SAEs are defied as events that are potentially life threatening or result in heath; cause congenital malformation; result in persistent/significant disability or incapacity; require inpatient hospitalization or prolongation of hospitalization; or reflect an otherwise notable medical event in the judgment of the study physician. Aes/SAEs will be monitored regularly by the study research team and medical staff, in particular by inquiring about adverse events at each weekly visit. Should Aes or SAEs occur, they will be recorded, rated as mild/moderate/severe by the study physician, and reported in accordance with UNC IRB requirements and GCP standards. Any Aes will be reported to the IRB in progress reports and at the conclusion of the study. SAEs and unanticipated events will be reported to the IRB and DSMB within 48 hours, and to NIAAA if judged as treatment-related. All staff working on this study will be made aware of the monitoring and reporting requirements for Aes/SAEs, and will have completed GCP training and other trainings required to conduct human subjects research at UNC.

Any Aes, SAEs or unanticipated problems will be followed until resolution by the Pis and/or research staff. Participants will be contacted regularly during follow-up and any observations of Aes/SAEs will be discussed among the Pis and the study physician at regular meetings (or immediately, in the event of SAEs or unanticipated problems). In the event of SAEs or unanticipated problems, the physician and

study Pis will be notified immediately, and the participant will be referred to or provided with appropriate medical support.

Participants will be advised to seek prompt medical attention in the event of an unanticipated medical event. In the event of an SAE, a member of the study medical staff will maintain regular contact with the participant until the event is resolved and fully documented. A determination of relatedness to medication will be made based on symptoms, timing, and other factors.

Monitoring will be the primary responsibility of the PI. In addition, a Data and Safety Monitoring Board will be established to maximize safety (the TraCS DSMB will be involved for this project, and will review progress at scheduled semiannual meetings during the trial).

8.3.7 REPORTING EVENTS TO PARTICIPANTS

If an event is deemed an AE or SAE, participants will receive a phone call from study personnel to inform them that the event has been recorded and will be reported as such. Any incidental findings uncovered during the baseline medical exam or weekly visits (e.g., via bloodwork or other measurements) will be reported to the participant by the study physician, and an appropriate follow-up plan will be recommended to the participant.

8.3.8 EVENTS OF SPECIAL INTEREST

Not applicable

8.3.9 REPORTING OF PREGNANCY

At the baseline visit and each study visit that involves an alcohol session, female subjects will undergo a urine pregnancy test upon arrival to the clinical site. In the case of a positive pregnancy test, the participant will be notified immediately, and study intervention will be discontinued while continuing safety follow-up. If any follow-up plan is recommended by the study physician, one of the staff members will communicate this recommendation to the participant.

8.4 UNANTICIPATED PROBLEMS

8.4.1 DEFINITION OF UNANTICIPATED PROBLEMS (UP)

The Office for Human Research Protections (OHRP) considers unanticipated problems involving risks to participants or others to include, in general, any incident, experience, or outcome that meets **all** of the following criteria:

- Unexpected in terms of nature, severity, or frequency given (a) the research procedures that are described in the protocol-related documents, such as the Institutional Review Board (IRB)-approved research protocol and informed consent document; and (b) the characteristics of the participant population being studied;

- Related or possibly related to participation in the research ("possibly related" means there is a reasonable possibility that the incident, experience, or outcome may have been caused by the procedures involved in the research); and
- Suggests that the research places participants or others at a greater risk of harm (including physical, psychological, economic, or social harm) than was previously known or recognized.

Any Ups identified by the study team will be discussed amongst the study leadership team, and corrective actions will be identified. If applicable, a protocol amendment will be developed and submitted to the IRB.

8.4.2 UNANTICIPATED PROBLEM REPORTING

The investigator will report unanticipated problems (Ups) to the reviewing Institutional Review Board (IRB), and if applicable, the DSMB. The UP report will include the following information:

- Protocol identifying information: protocol title and number, PI's name, and the IRB project number;
- A detailed description of the event, incident, experience, or outcome;
- An explanation of the basis for determining that the event, incident, experience, or outcome represents an UP;
- A description of any changes to the protocol or other corrective actions that have been taken or are proposed in response to the UP.

8.4.3 REPORTING UNANTICIPATED PROBLEMS TO PARTICIPANTS

In the case of a UP that may impact participants' safety or alter their experience during the trial (e.g., by necessitating a change in protocol), participants will be notified by a study team member at the subsequent visit. In the event of any UP that may have an imminent impact on participant safety, a team member will contact the participant by phone to inform them of the problem.

9 STATISTICAL CONSIDERATIONS

9.1 STATISTICAL HYPOTHESES

- Primary Efficacy Endpoint(s):
    - Breath alcohol concentration (BrAC) and volume of alcohol consumed during laboratory alcohol self-administration procedures. The outcomes are continuous variables. The effect of interest is the interaction between medication group (semaglutide vs. placebo) and time/dosage (baseline vs. 0.5mg). The baseline session will take place between Weeks 0 and 1, and the 0.5mg session will take place between weeks 8 and 9.
- Secondary Efficacy Endpoint(s):
    - Changes in subjective responses to alcohol (stimulation, sedation, craving) and alcohol and cigarette demand during laboratory alcohol challenge procedures. The outcomes will be

analyzed as continuous variables. The effect of interest is the interaction between medication group (semaglutide vs. placebo) and time/dosage (baseline vs. 0.5mg). The baseline session will take place between Weeks 0 and 1, and the 0.5mg session will take place between weeks 8 and 9.

- Changes in drinks per day and cigarette consumption over the course of the trial, as assessed via daily surveys. The outcome variables will be treated as continuous, however, this assumption will be tested and the modeling approach will revised as needed to properly account for the nature of the outcome variable (e.g., analyze as count variable with negative binomial distribution). The effect of interest is the interaction between medication group (semaglutide vs. placebo) and time (Week 1 to Week 10).

9.2 SAMPLE SIZE DETERMINATION

Because no human data on semaglutide's effects on alcohol consumption outcomes are available, there is no a priori effect size estimate to facilitate power calculations. We will seek a final sample size of 36 participants. Assuming at least 36 enrollees have complete data, our analyses suggest that this allows sufficient power (.80) to detect a medium effect size (f = .25) for an interaction between group (medication vs. placebo) and dosage (baseline, 0.25mg, 0.5mg). This estimate assumes an alpha level of .05 and a within-person correlation ® of 0.5 across time points. Further, because our multilevel model (MLM) analyses will involve more time points for certain analyses (e.g., daily assessments), this approach will increase power further, which is advantageous in the event that medication effects are in the small range. Assuming a 25% attrition rate, we anticipate needing to randomize 48 enrollees to arrive at a final sample of 36.

9.3 POPULATIONS FOR ANALYSES

Primary analyses will include all participants who were randomized and received a medication or placebo dose following the Week 0 visit (randomization will occur after the Week 0 visit, just prior to the Week 1 visit). MLM analyses can accommodate missing data, allowing inclusion of participants without complete data (e.g., missed visits or missed daily survey responses). Reasons for missing data and drop-out will be documented in the study database and reported in any publications.

A supplementary per-protocol analyses will be conducted using all participants who received 1) at least 7 of the 9 scheduled doses, and 2) those participants who receive the final (1.0mg) dose. This planned analysis will be conducted irrespective of the number of participants in these groups. If the sample permits, an exploratory subgroup analysis will examine participants as a function of weight (overweight/obese vs. not overweight or obese).

9.4 STATISTICAL ANALYSES

9.4.1 GENERAL APPROACH

Primary analyses will rely on multilevel models (MLM), which are well suited to examining within-person changes over time, while also accommodating missing data and unequal group sizes, and allowing the evaluation of between-subjects moderators (e.g., overweight/obesity status) if necessary. MLM with

maximum likelihood estimation can accommodate missing data under the assumption that the data are missing at random. Secondary, sensitivity analyses will be conducted to test the robustness of study findings for violations of the assumption that data is missing at random. Specifically, we will examine if baseline variables are associated with data missingness (for instance, age, sex, level of substance use) using t-tests and chi-square analyses (for continuous and dichotomous variables, respectively). Baseline variables that are associated with data missingness will be included as covariates in additional adjusted models (that adjust for the baseline variables related to missingness). Assumptions for the statistical methods being used will be tested. Should there be evidence of violations of these assumptions (e.g., presence of outlying and influential cases, distributional concerns for residuals, and so on), appropriate corrective actions will be taken. Sensitivity analyses will be used to determine the extent to which the main study results are influenced by these violations. We will focus our interpretations on small p-values ($p < .05$) that indicate evidence against the null hypothesis. We will report the number of decimal places of the p-value in line with the journal's guidelines and APA recommendations (usually 2 or 3 decimal places). For specific outcomes, the units of measurement and expected ranges of values (or example ranges, if the full range is unknown) are noted in the subsequent analysis sections 9.4.2 and 9.4.3. Any extreme outliers (defined as >3.0 standard deviations from the mean) will be recoded to one unit greater than the next most extreme value, in order to reduce undue influence of extreme outliers. For all statistical tests, confidence intervals will be evaluated in combination with tests of statistical significance. Assumptions for the statistical methods being used will be tested. Should there be evidence of violations of these assumptions (e.g., presence of outlying and influential cases, distributional concerns for residuals, and so on), appropriate corrective actions will be taken. Sensitivity analyses will be used to determine the extent to which the main study results are influenced by these violations.

9.4.2 ANALYSIS OF THE PRIMARY EFFICACY ENDPOINT(S)

<u>Alcohol self-administration sessions:</u> MLM will be used to examine changes in laboratory self-administration (defined as peak BAC and quantity of alcohol consumed, co-primary outcomes) as a function of medication phase (baseline; 0.5mg). These analyses will use medication phase as the within-subjects factor and condition (semaglutide vs. placebo) as the between-person factor. Self-administration will be measured based on recorded breath alcohol concentration (BrAC), measured in g% (e.g., .00g% - .08g%).

9.4.3 ANALYSIS OF THE SECONDARY ENDPOINT(S)

<u>Alcohol challenge sessions:</u> MLM will also be used to examine changes in 1) cigarette and alcohol demand during alcohol challenge (based on corresponding purchase tasks) (e.g., based on number of hypothetical drinks/cigarettes consumed at a given price point), and 2) self-reported stimulation (0-10 range), sedation (0-10 range), and craving (1-7 range), from BAC = .00 to BAC = .06g%, as a function of medication phase (within-person) and drug condition (between person). These analyses will examine changes in outcomes from BAC = .00 to .06 using medication phase as the within-subjects factor and condition as the between-person factor. The analyses will be complemented by analyses of peak subjective effects (e.g., stimulation, sedation, craving, on the same scales as noted above), across the two alcohol administration sessions (AC1 and AC2).

<u>Daily self-report data:</u> We will use MLM to estimate within-person changes in primary outcomes (e.g., daily alcohol use [number of drinks consumed per day] and cigarette use [number of cigarettes smoked per day] over the trial period), using study week as the within-subjects factor and condition (semaglutide, placebo) as the between-person factor.

9.4.4 SAFETY ANALYSES

Total side effects reported (e.g., 0-15), and number of severe side effects reported during each medication phase will be assessed using MLM with medication phase (baseline, 0.25mg, 0.5mg, 1.0mg) as the within-subject factor and condition (semaglutide, placebo) as the between-person factor. . These count variables will be analyzed using a negative binomial MLM, however, this assumption will be tested and the modeling approach will revised as needed to properly account for the nature of the outcome variable (e.g., zero-inflated negative binomial regression).

9.4.5 BASELINE DESCRIPTIVE STATISTICS

Descriptive statistics will be used to report relevant demographics such as age, sex, and education.

9.4.6 PLANNED INTERIM ANALYSES

No interim analyses of efficacy will be planned.

9.4.7 SUB-GROUP ANALYSES

Not applicable

9.4.8 TABULATION OF INDIVIDUAL PARTICIPANT DATA

Not applicable

9.4.9 EXPLORATORY ANALYSES

Sex will be examined as an exploratory moderator in all analyses.

10 SUPPORTING DOCUMENTATION AND OPERATIONAL CONSIDERATIONS

10.1 REGULATORY, ETHICAL, AND STUDY OVERSIGHT CONSIDERATIONS

10.1.1 INFORMED CONSENT PROCESS

In obtaining and documenting informed consent, the team will comply with applicable regulatory requirements (e.g., 45 CFR Part 46, 21 CFR Part 50, 21 CFR Part 56) and adhere to ICH GCP. Consent will be obtained remotely (via web teleconference and Docusign, as described previously) by a qualified study staff member in person at the outset of the baseline session. Additional procedures are noted below.

10.1.1.1 CONSENT/ASSENT AND OTHER INFORMATIONAL DOCUMENTS PROVIDED TO PARTICIPANTS

Consent forms will describe the study intervention, study procedures, and potential risks. The participant's electronic signature indicating consent is required prior to starting intervention/administering study intervention. Participants will also be given an informational sheet about the study medication. Additional procedures are described below.

10.1.1.2 CONSENT PROCEDURES AND DOCUMENTATION

Informed consent is a process that is initiated prior to the individual's agreeing to participate in the study and continues throughout the individual's study participation. Consent forms will be Institutional Review Board (IRB)-approved and the participant will be asked to read and review the document. The initial consent process will be conducted remotely using a HIPAA-compliant web platform (e.g., WebEx, Zoom, Teams) and Part 11-compliant Docusign to maintain optimal safety. A copy of the consent form will be provided to the participant in advance of the scheduled videoconference. A qualified team member will explain the research study to the participant and answer any questions that may arise. A verbal explanation will be provided in terms suited to the participant's comprehension of the purposes, procedures, and potential risks of the study and of their rights as research participants. Participants will have the opportunity to carefully review the consent form and ask questions prior to signing. The participants should have the opportunity to discuss the study with their family or surrogates or think about it prior to agreeing to participate. The participant will electronically sign the informed consent document prior to any procedures being done specifically for the study. Participants must be informed that participation is voluntary and that they may withdraw from the study at any time, without prejudice. A copy of the signed informed consent document will be given to the participants for their records. The informed consent process will be conducted and documented in the source document (including the date), and the form signed (via Docusign), before the participant undergoes any study-specific procedures. The rights and welfare of the participants will be protected by emphasizing to them that the study is voluntary and the quality of their medical care will not be adversely affected if they decline to participate. While the initial consent form will be conducted electronically, supplementary consent forms (e.g., consent to unencrypted communication, HIPAA authorization, and biospecimen consent) may be conducted either remotely (via Docusign) in advance of the baseline visit or in person (paper form) at the baseline visit, depending on timing.

10.1.2 STUDY DISCONTINUATION AND CLOSURE

This study may be temporarily suspended or prematurely terminated if there is sufficient reasonable cause. Written notification, documenting the reason for study suspension or termination, will be provided by the suspending or terminating party to study participants, investigator, funding agency, and regulatory authorities. If the study is prematurely terminated or suspended, the Principal Investigator (PI) will promptly inform study participants, the Institutional Review Board (IRB), and sponsor and will provide the reason(s) for the termination or suspension. Study participants will be contacted, as applicable, and be informed of changes to study visit schedule. Circumstances that may warrant termination or suspension include, but are not limited to determination of unexpected, significant, or unacceptable risk to participants. Study may resume once concerns about safety, protocol compliance, and data quality are addressed, and satisfy the sponsor, IRB and/or Food and Drug Administration (FDA).

10.1.3 CONFIDENTIALITY AND PRIVACY

Participant confidentiality and privacy is strictly held in trust by the participating investigators, their staff, and the sponsor(s) and their interventions. This confidentiality is extended to cover testing of biological samples and genetic tests in addition to the clinical information relating to participants. Therefore, the study protocol, documentation, data, and all other information generated will be held in strict confidence. No information concerning the study or the data will be released to any unauthorized third party. All research activities will be conducted in as private a setting as possible.

Any computerized data will be password protected and contained on a secure, password-protected computer or a secure server. Recruitment data (de-identified) will be stored in a secure, password-protected file accessible only to the study investigators and personnel. At the end of recruitment, eligible subject files will be separated from those failing to meet eligibility criteria, and records of ineligible recruitment subjects will be anonymized. Data pertaining to screening failures will be tabulated in an anonymized fashion. Data collected in other formats (e.g., medical screening notes) will be labeled only by study identifier and stored in binders in secure, locked cabinets while the study is taking place. Files will be stored and archived in accordance with Good Clinical Practice (GCP) standards. For all questionnaire and interview assessments, participants will be informed that they may decline to answer any question if they so choose.

The study monitor, other authorized representatives of the sponsor, representatives of the Institutional Review Board (IRB), regulatory agencies or pharmaceutical company supplying study product may inspect all documents and records required to be maintained by the investigator, including but not limited to, medical records (office, clinic, or hospital) and pharmacy records for the participants in this study. The clinical study site will permit access to such records.

The study participant's contact information will be securely stored during the study for purposes of maintain contact with participants, and for communicating any safety information or unanticipated problems. At the end of the study, all records will continue to be kept in a secure location for as long a period as dictated by the reviewing IRB, Institutional policies, or sponsor requirements.

Study participant research data, which is for purposes of statistical analysis and scientific reporting, will be stored at UNC in the PI's laboratory and on secure backup servers with password-protected access. This will not include the participant's contact or identifying information. Rather, individual participants and their research data will be identified by a unique study identification number. Electronic data are stored on a password protected drive only accessible by the research team. Data obtained on paper will be transferred to an electronic data file (Excel, SPSS, REDCap), stored on a secure server, and identified by participants ID. For non-computer based forms, the data collection sheets are stored in a locked cabinet in a separate locked data storage space at the PI's laboratory.

At the end of the study, all study databases will be de-identified and archived in the PI's laboratory and backed up on a secure server hosted at UNC. De-identified data resulting from this study may also be presented at meetings, published in journals or presentations. In addition, this study will be registered at ClinicalTrials.gov, and de-identified information from this study will be submitted to ClinicalTrials.gov.

10.1.4 FUTURE USE OF STORED SPECIMENS AND DATA

Data collected for this study will be analyzed and stored at the PI's laboratory. The study biostatistician will be given access to the data necessary for conducting specified analyses, and data will be de-identified prior to giving access.

De-identified biological samples will be stored at UNC for the duration of the study, until all biological tests are completed and the data transferred to electronic format. De-identified DNA samples will be stored indefinitely, provided the participant has given consent for DNA storage, in order to examine future questions related to phenotypes of interest for this line of research.

During the conduct of the study, an individual participant can choose to withdraw consent to have biological specimens stored for future research. However, withdrawal of consent with regard to biosample storage may not be possible after the study is completed (after specimens are de-identified).

10.1.5 KEY ROLES AND STUDY GOVERNANCE

Provide the name and contact information of the Principal Investigator and the Medical Monitor.

**Principal Investigator**	**Medical Monitor**
Christian Hendershot, Ph.D., Associate Professor	*TBD (The medical monitor and DSMB members will be identified and added prior to data collection)*
UNC-Chapel Hill	
104 Manning Dr.	
Christian_Hendershot@med.unc."	
Christian_Hendershot@med.unc.edu	

The investigative team is summarized on the face page of this protocol. As noted, a DSMB will be formed prior to carrying out the study.

Safety oversight will be under the direction of a Data and Safety Monitoring Board (DSMB). Members of the DSMB will be independent from the study conduct and free of conflict of interest. The DSMB will dictate the intervals at which they meet to assess safety and efficacy data. At this time, each data element that the DSMB needs to assess will be clearly defined. The DSMB will provide its input to the IRB and National Institutes of Health as necessary.

10.1.6 CLINICAL MONITORING

Clinical site monitoring is conducted to ensure that the rights and well-being of trial participants are protected, that the reported trial data are accurate, complete, and verifiable, and that the conduct of the trial is in compliance with the currently approved protocol/amendment(s), with International Conference on Harmonisation Good Clinical Practice (ICH GCP), and with applicable regulatory requirement(s). A local study monitor will be appointed to provide on-site monitoring during the course of the research study.

10.1.7 QUALITY ASSURANCE AND QUALITY CONTROL

The PI will be responsible for addressing quality control issues and correcting errors or protocol deviations. The PI will have oversight over training study staff and ensuring that protocol elements are carried out with fidelity. Quality control will be ensured by developing SOPs for protocol elements to standardize data collection procedures. The investigational site will provide direct access to all trial related sites, source data/documents, and reports for the purpose of monitoring and auditing by the sponsor, and inspection by local and regulatory authorities.

10.1.8 DATA HANDLING AND RECORD KEEPING

10.1.8.1 DATA COLLECTION AND MANAGEMENT RESPONSIBILITIES

As a single-site, small-scale trial, the project will not involve coordinated data collection and management responsibilities across sites/teams. Data safety and storage precautions are outlined elsewhere in the study protocol.

10.1.8.2 STUDY RECORDS RETENTION

Study records will be retained for a minimum of 7 years following study completion. Paper source documents will be archived in accordance with UNC policies. Electronic materials and de-identified datasets will be stored securely as outlined above.

10.1.9 PROTOCOL DEVIATIONS

A protocol deviation is any noncompliance with the clinical trial protocol, International Conference on Harmonisation Good Clinical Practice (ICH GCP), or Manual of Procedures (MOP) requirements. The PI will report deviations within 7 working days of identification of the protocol deviation, or within 7 working days of the scheduled protocol-required activity. All deviations must be addressed in study source documents, reported to NIAAA Program Official and the IRB. Protocol deviations must be sent to the reviewing Institutional Review Board (IRB) per their policies. The site investigator is responsible for knowing and adhering to the reviewing IRB requirements. Further details about the handling of protocol deviations will be included in the MOP.

10.1.10 PUBLICATION AND DATA SHARING POLICY

This study will be conducted in accordance with the following publication and data sharing policies and regulations:

National Institutes of Health (NIH) Public Access Policy, which ensures that the public has access to the published results of NIH funded research. It requires scientists to submit final peer-reviewed journal manuscripts that arise from NIH funds to the digital archive PubMed Central upon acceptance for publication.

This study will comply with the NIH Data Sharing Policy and Policy on the Dissemination of NIH-Funded Clinical Trial Information and the Clinical Trials Registration and Results Information Submission rule. As such, this trial will be registered at ClinicalTrials.gov, and results information from this trial will be submitted to ClinicalTrials.gov. In addition, every attempt will be made to publish results in peer-reviewed journals. Data from this study may be requested from other researchers after the completion of the primary endpoint by contacting the PI.

10.1.11 CONFLICT OF INTEREST POLICY

Any actual conflict of interest of persons who have a role in the design, conduct, analysis, publication, or any aspect of this trial will be disclosed and managed. Furthermore, persons who have a perceived conflict of interest will be required to have such conflicts managed in a way that is appropriate to their participation in the design and conduct of this trial. All investigators will make a conflict of interest declaration at the outset of the study and at regular intervals thereafter in accordance with UNC policies.

10.2 ADDITIONAL CONSIDERATIONS

Not applicable

10.3 ABBREVIATIONS

AASE	Alcohol Abstinence Self-Efficacy
AE	Adverse Event
APT	Alcohol Purchase Task
AUD	Alcohol Use Disorder
AUDIT	Alcohol Use Disorder Identification Test
AUQ	Alcohol Urge Questionnaire
BAES	Biphasic Alcohol Effects Scale
BIS-11	Barratt Impulsivity Scale
BMI	Body Mass Index
BrAC	Breath Alcohol Concentration
CASE	Cigarette Abstinence Self-Efficacy
CES-D	Center for Epidemiological Studies Depression Scale
CEQ	Control of Eating Questionnaire
CFR	Code of Federal Regulations
CMP	Clinical Monitoring Plan
CO	Carbon Monoxide
CPT	Cigarette Purchase Task
CRF	Case Report Form
CTRC	Clinical & Translational Research Center
DEQ	Drug Effects Questionnaire
DNA	Deoxyribonucleic acid
DSM-5	Diagnostic and Statistical Manual for Mental Disorders v.5

DSMB	Data Safety Monitoring Board
FDA	Food and Drug Administration
FPT	Food Purchase Task
FTND	Fagerstrom test for Nicotine Dependence
GCP	Good Clinical Practice
GLP-1	Glucagon-Like Peptide 1
GLP-1R	Glucagon-Like Peptide 1 Receptor
HbA1c	Glycated hemoglobin
ICH	International Conference on Harmonisation
ICS	Impaired Control Scale
IDS	Inventory of Drinking Situations
IRB	Institutional Review Board
MCQ	Monetary Choice Questionnaire
MNWS	Minnesota Nicotine Withdrawal Scale
MOP	Manual of Procedures
NC Tracs	North Carolina Translational & Clinical Sciences Institute
NDSS	Nicotine Dependence Syndrome Scale
NIAAA	National Institute on Alcohol Abuse and Alcoholism
NIH	National Institutes of Health
NRT	Nicotine Replacement Therapy
PACS	Penn Alcohol Craving Scale
PI	Principal Investigator
POMS-SF	Profile Of Mood States Short Form
PAWS	Prediction of Alcohol Withdrawal Severity Scale
QA	Quality Assurance
QC	Quality Control
RBEDS	Reward Based Eating Drive Scale
RPI-EROS	Reward Probability Index – Environmental Reward Observation Scale
SAE	Serious Adverse Event
SAFTEE	Systematic Assessment for Treatment Emergent Effects
SAWS	Short Alcohol Withdrawal Scale
SNAQ	Simplified Nutritional Appetite Questionnaire
SOA	Schedule of Activities
SOP	Standard Operating Procedure
SRE	Self-Report of the Effects of Alcohol Questionnaire
SUD	Substance Use Disorder
T2D	Type II Diabetes
TAS-20	Toronto Alexithymia Scale
TFEQ	Three Factor Eating Questionnaire
TLFB	Timeline Follow-Back Interview
TQSU	Tiffany Questionnaire of Smoking Urges
UP	Unanticipated Problem
UPPS-P	UPPS-P Impulsive Behavior Scale
WISDM	Wisconsin Inventory of Smoking Dependence Motives
WSWS	Wisconsin Smoking Withdrawal Scale
5-HT2C	Serotonin 2C Receptor

10.4 PROTOCOL AMENDMENT HISTORY

The table below is intended to capture changes of IRB-approved versions of the protocol, including a description of the change and rationale. A Summary of Changes table for the current amendment is located in the Protocol Title Page.

Version	Date	Description of Change	Brief Rationale
1.2	July 26 2021	Elaboration on statistical analyses and clarification on other procedural issues.	Minor changes requested during initial review of the protocol.
1.3	Feb 28 2021	Clarification of medication dispensing procedures, addition of personnel, addition of Docusign procedures, other protocol modifications.	Changes addressed issues that emerged while preparing for recruitment and meeting with IDS and study investigators.
1.4	June 23 2022	Minor revisions to select assessment instruments, exclusion criteria, and clarification of medication dosing and DSMB meeting plan	Refinement of study procedures and clarification of monitoring schedule as requested by DSMB and IDS
1.5	August 22 2022	Updates to medication storage, use of Phone.com for text messaging, select questionnaire modifications, modifications to alcohol self-administration session	Medication storage changes requested by IDS; Phone.com added as per UNC requirements for text messaging; other changes intended to streamline and improve assessment procedures

11 REFERENCES

1. Koob, G.F., G. Kenneth Lloyd, and B.J. Mason, *Development of pharmacotherapies for drug addiction: a Rosetta stone approach.* Nat Rev Drug Discov, 2009. **8**(6): p. 500-15.
2. Litten, R.Z., et al., *Medications development to treat alcohol dependence: a vision for the next decade.* Addiction biology, 2012. **17**(3): p. 513-27.
3. Litten, R.Z., et al., *Discovery, Development, and Adoption of Medications to Treat Alcohol Use Disorder: Goals for the Phases of Medications Development.* Alcohol Clin Exp Res, 2016. **40**(7): p. 1368-79.
4. Litten, R.Z., et al., *Potential medications for the treatment of alcohol use disorder: An evaluation of clinical efficacy and safety.* Subst Abus, 2016. **37**(2): p. 286-98.
5. Litten, R.Z., et al., *Heterogeneity of alcohol use disorder: understanding mechanisms to advance personalized treatment.* Alcohol Clin Exp Res, 2015. **39**(4): p. 579-84.
6. Heilig, M., et al., *Pharmacogenetic approaches to the treatment of alcohol addiction.* Nature reviews. Neuroscience, 2011. **12**(11): p. 670-84.
7. Falk, D.E., H.Y. Yi, and S. Hiller-Sturmhofel, *An epidemiologic analysis of co-occurring alcohol and tobacco use and disorders: findings from the National Epidemiologic Survey on Alcohol and Related Conditions.* Alcohol Res Health, 2006. **29**(3): p. 162-71.
8. McKee, S.A. and A.H. Weinberger, *How can we use our knowlegde of alcohol-tobacco interactions to reduce alcohol use?* Annu Rev Clin Psychol, 2013. **9**: p. 649–674.
9. Organization, W.H., *Global Health Risks. Mortality and Burden of Disease Attributable to Selected Major Risks*. 2009, World Health Organization: Geneva, Switzerland.
10. Dawson, D.A., *Drinking as a risk factor for sustained smoking.* Drug Alcohol Depend, 2000. **59**(3): p. 235-49.
11. Yardley, M.M., M.M. Mirbaba, and L.A. Ray, *Pharmacological Options for Smoking Cessation in Heavy-Drinking Smokers.* CNS Drugs, 2015. **29**(10): p. 833-45.
12. Prabhu, A., K.O. Obi, and J.H. Rubenstein, *The synergistic effects of alcohol and tobacco consumption on the risk of esophageal squamous cell carcinoma: a meta-analysis.* Am J Gastroenterol, 2014. **109**(6): p. 822-7.
13. Pelucchi, C., et al., *Cancer risk associated with alcohol and tobacco use: focus on upper aero-digestive tract and liver.* Alcohol Res Health, 2006. **29**(3): p. 193-8.
14. Kahler, C.W., N.S. Spillane, and J. Metrik, *Alcohol use and initial smoking lapses among heavy drinkers in smoking cessation treatment.* Nicotine Tob Res, 2010. **12**(7): p. 781-5.
15. Roche, D.J., et al., *Current insights into the mechanisms and development of treatments for heavy drinking cigarette smokers.* Curr Addict Rep, 2016. **3**(1): p. 125-137.
16. McKee, S.A., et al., *Varenicline reduces alcohol self-administration in heavy-drinking smokers.* Biol Psychiatry, 2009. **66**(2): p. 185-90.
17. King, A., et al., *Naltrexone decreases heavy drinking rates in smoking cessation treatment: an exploratory study.* Alcohol Clin Exp Res, 2009. **33**(6): p. 1044-50.
18. O'Malley, S.S., et al., *Dose-dependent reduction of hazardous alcohol use in a placebo-controlled trial of naltrexone for smoking cessation.* Int J Neuropsychopharmacol, 2009. **12**(5): p. 589-97.
19. King, A., et al., *Effects of the opioid receptor antagonist naltrexone on smoking and related behaviors in smokers preparing to quit: a randomized controlled trial.* Addiction, 2013. **108**(10): p. 1836-44.
20. Anton, R.F., et al., *Nicotine-Use/Smoking Is Associated with the Efficacy of Naltrexone in the Treatment of Alcohol Dependence.* Alcohol Clin Exp Res, 2018. **42**(4): p. 751-760.
21. Fucito, L.M., et al., *Cigarette smoking predicts differential benefit from naltrexone for alcohol dependence.* Biol Psychiatry, 2012. **72**(10): p. 832-8.
22. Hartmann-Boyce, J., et al., *Efficacy of interventions to combat tobacco addiction: Cochrane update of 2013 reviews.* Addiction, 2014. **109**(9): p. 1414-25.
23. Kahler, C.W., et al., *A Double-Blind Randomized Placebo-Controlled Trial of Oral Naltrexone for Heavy-Drinking Smokers Seeking Smoking Cessation Treatment.* Alcohol Clin Exp Res, 2017. **41**(6): p. 1201-1211.

24. Mitchell, J.M., et al., *Varenicline decreases alcohol consumption in heavy-drinking smokers.* Psychopharmacology (Berl), 2012. **223**(3): p. 299-306.
25. Fucito, L.M., et al., *A preliminary investigation of varenicline for heavy drinking smokers.* Psychopharmacology (Berl), 2011. **215**(4): p. 655-63.
26. Litten, R.Z., et al., *A double-blind, placebo-controlled trial assessing the efficacy of varenicline tartrate for alcohol dependence.* J Addict Med, 2013. **7**(4): p. 277-86.
27. Roche, D.J., et al., *Combined varenicline and naltrexone treatment reduces smoking topography intensity in heavy-drinking smokers.* Pharmacol Biochem Behav, 2015. **134**: p. 92-8.
28. Ray, L.A., et al., *Varenicline, low dose naltrexone, and their combination for heavy-drinking smokers: human laboratory findings.* Psychopharmacology (Berl), 2014. **231**(19): p. 3843-53.
29. Andersen, A., et al., *Glucagon-like peptide 1 in health and disease.* Nature Reviews Endocrinology, 2018. **14**(7): p. 390-403.
30. Jerlhag, E., *Alcohol-mediated behaviours and the gut-brain axis; with focus on glucagon-like peptide-1.* Brain Res, 2020. **1727**: p. 146562.
31. Brunchmann, A., M. Thomsen, and A. Fink-Jensen, *The effect of glucagon-like peptide-1 (GLP-1) receptor agonists on substance use disorder (SUD)-related behavioural effects of drugs and alcohol: A systematic review.* Physiol Behav, 2019. **206**: p. 232-242.
32. Egecioglu, E., J.A. Engel, and E. Jerlhag, *The glucagon-like peptide 1 analogue Exendin-4 attenuates the nicotine-induced locomotor stimulation, accumbal dopamine release, conditioned place preference as well as the expression of locomotor sensitization in mice.* PLoS One, 2013. **8**(10): p. e77284.
33. Anderberg, R.H., et al., *Glucagon-Like Peptide 1 and Its Analogs Act in the Dorsal Raphe and Modulate Central Serotonin to Reduce Appetite and Body Weight.* Diabetes, 2017. **66**(4): p. 1062-1073.
34. Howell, E., et al., *Glucagon-Like Peptide-1 (GLP-1) and 5-Hydroxytryptamine 2c (5-HT(2c)) Receptor Agonists in the Ventral Tegmental Area (VTA) Inhibit Ghrelin-Stimulated Appetitive Reward.* Int J Mol Sci, 2019. **20**(4).
35. Asarian, L., *Loss of cholecystokinin and glucagon-like peptide-1-induced satiation in mice lacking serotonin 2C receptors.* American Journal of Physiology-Regulatory, Integrative and Comparitive Physiology, 2009. **296**(1): p. R51-R56.
36. Nonogaki, K., et al., *The contribution of serotonin 5-HT2C and melanocortin-4 receptors to the satiety signaling of glucagon-like peptide 1 and liraglutide, a glucagon-like peptide 1 receptor agonist, in mice.* Biochem Biophys Res Commun, 2011. **411**(2): p. 445-8.
37. Doggrell, S.A., *Sgemaglutide in type 2 diabetes - is it the best glucagon-like peptide 1 receptor agonist (GLP-1R agonist)?* Expert Opin Drug Metab Toxicol, 2018. **14**(3): p. 371-377.
38. Litten, R.Z., et al., *Development of medications for alcohol use disorders: recent advances and ongoing challenges.* Expert Opin Emerg Drugs, 2005. **10**(2): p. 323-43.
39. Bujarski, S. and L.A. Ray, *Experimental psychopathology paradigms for alcohol use disorders: Applications for translational research.* Behav Res Ther, 2016.
40. Hendershot, C.S., et al., *Effects of naltrexone on alcohol self-administration and craving: meta-analysis of human laboratory studies.* Addict Biol, 2016.
41. McKee, S.A., *Developing human laboratory models of smoking lapse behavior for medication screening.* Addict Biol, 2009. **14**(1): p. 99-107.
42. Plebani, J.G., et al., *Human laboratory paradigms in alcohol research.* Alcohol Clin Exp Res, 2012. **36**(6): p. 972-83.
43. Ray, L.A., K.E. Hutchison, and M. Tartter, *Application of human laboratory models to pharmacotherapy development for alcohol dependence.* Curr Pharm Des, 2010. **16**(19): p. 2149-58.
44. Lerman, C., et al., *Translational research in medication development for nicotine dependence.* Nat Rev Drug Discov, 2007. **6**(9): p. 746-62.
45. MacKillop, J. and J.G. Murphy, *A behavioral economic measure of demand for alcohol predicts brief intervention outcomes.* Drug Alcohol Depend, 2007. **89**(2-3): p. 227-33.
46. Amlung, M., et al., *Increased behavioral economic demand and craving for alcohol following a laboratory alcohol challenge.* Addiction, 2015. **110**(9): p. 1421-8.
47. Green, R. and L.A. Ray, *Effects of varenicline on subjective craving and relative reinforcing value of cigarettes.* Drug Alcohol Depend, 2018. **188**: p. 53-59.

48. Murphy, C.M., et al., *Effects of varenicline versus transdermal nicotine replacement therapy on cigarette demand on quit day in individuals with substance use disorders.* Psychopharmacology (Berl), 2017. **234**(16): p. 2443-2452.
49. Roache, J.D., *Role of the Human Laboratory in the Development of Medications for Alcohol and Drug Dependence*, in *Addiction Medicine*, B.A. Johnson, Editor. 2010, Springer: New York. p. 129-157.
50. Enoch, M.A., et al., *Ethical considerations for administering alcohol or alcohol cues to treatment-seeking alcoholics in a research setting: can the benefits to society outweigh the risks to the individual? A commentary in the context of the National Advisory Council on Alcohol Abuse and Alcoholism -- Recommended Council Guidelines on Ethyl Alcohol Administration in Human Experimentation (2005).* Alcohol Clin Exp Res, 2009. **33**(9): p. 1508-12.
51. Motschman, C.A., et al., *Selection criteria limit generalizability of smoking pharmacotherapy studies differentially across clinical trials and laboratory studies: A systematic review on varenicline.* Drug Alcohol Depend, 2016. **169**: p. 180-189.
52. Alcoholism, N.I.o.A.A.a., *Recommended council guidelines on ethyl alcohol administration in human experimentation*. 2005, Department of Health and Human Services: Rockville, MD.
53. First MB, W.J., Karg RS, Spitzer RL, *Structured Clinical Interview for DSM-5—Research Version (SCID-5 for DSM-5, Research Version; SCID-5-RV)*. 2015, Arlington, VA: American Psychiatric Association.
54. Sobell, L.C., et al., *Reliability of a timeline method: assessing normal drinkers' reports of recent drinking and a comparative evaluation across several populations.* Br J Addict, 1988. **83**(4): p. 393-402.
55. Saunders, J.B., et al., *Development of the Alcohol Use Disorders Identification Test (AUDIT): WHO Collaborative Project on Early Detection of Persons with Harmful Alcohol Consumption--II.* Addiction, 1993. **88**(6): p. 791-804.
56. Schuckit, M.A., et al., *The Self-Rating of the Effects of Alcohol Questionnaire as a Predictor of Alcohol-Related Outcomes in 12-Year-Old Subjects.* Alcohol and Alcoholism, 2008. **43**(6): p. 641-646.
57. Schuckit, M.A. and T.L. Smith, *The relationships of a family history of alcohol dependence, a low level of response to alcohol and six domains of life functioning to the development of alcohol use disorders.* Journal of Studies on Alcohol, 2000. **61**(6): p. 827-835.
58. Heatherton, T.F., et al., *The Fagerstrom Test for Nicotine Dependence: a revision of the Fagerstrom Tolerance Questionnaire.* Br J Addict, 1991. **86**(9): p. 1119-27.
59. Shiffman, S., A. Waters, and M. Hickcox, *The nicotine dependence syndrome scale: a multidimensional measure of nicotine dependence.* Nicotine Tob Res, 2004. **6**(2): p. 327-48.
60. Patton, J.H., M.S. Stanford, and E.S. Barratt, *Factor structure of the Barratt impulsiveness scale.* Journal of clinical psychology, 1995. **51**(6): p. 768-774.
61. Whiteside, S.P., et al., *Validation of the UPPS impulsive behaviour scale: a four-factor model of impulsivity.* European Journal of personality, 2005. **19**(7): p. 559-574.
62. Heather, N., et al., *Development of a scale for measuring impaired control over alcohol consumption: a preliminary report.* Journal of studies on alcohol, 1993. **54**(6): p. 700-709.
63. Radloff, L.S., *The CES-D scale: A self-report depression scale for research in the general population.* Applied psychological measurement, 1977. **1**(3): p. 385-401.
64. Leising, D., T. Grande, and R. Faber, *The Toronto Alexithymia Scale (TAS-20): A measure of general psychological distress.* Journal of research in personality, 2009. **43**(4): p. 707-710.
65. Carvalho, J.P., et al., *The Reward Probability Index: Design and validation of a scale measuring access to environmental reward.* Behavior therapy, 2011. **42**(2): p. 249-262.
66. Tiffany, S.T. and D.J. Drobes, *The development and initial validation of a questionnaire on smoking urges.* Br J Addict, 1991. **86**(11): p. 1467-76.
67. Welsch, S.K., et al., *Development and validation of the Wisconsin Smoking Withdrawal Scale.* Exp Clin Psychopharmacol, 1999. **7**(4): p. 354-61.
68. DiClemente, C.C., et al., *The Alcohol Abstinence Self-Efficacy scale.* J Stud Alcohol, 1994. **55**(2): p. 141-8.
69. Gwaltney, C.J., et al., *Does smoking abstinence self-efficacy vary across situations? Identifying context-specificity within the Relapse Situation Efficacy Questionnaire.* J Consult Clin Psychol, 2001. **69**(3): p. 516-27.

70. Mann, K., et al., *Precision Medicine in Alcohol Dependence: A Controlled Trial Testing Pharmacotherapy Response Among Reward and Relief Drinking Phenotypes.* Neuropsychopharmacology, 2018. **43**(4): p. 891-899.
71. Sties, S.W., et al., *Simplified Nutritional Appetite Questionnaire (SNAQ) for cardiopulmonary and metabolic rehabilitation program.* Revista Brasileira de Medicina do Esporte, 2012. **18**: p. 313-317.
72. Dalton, M., et al., *Preliminary validation and principal components analysis of the Control of Eating Questionnaire (CoEQ) for the experience of food craving.* European journal of clinical nutrition, 2015. **69**(12): p. 1313-1317.
73. Lesdema, A., et al., *Characterization of the Three-Factor Eating Questionnaire scores of a young French cohort.* Appetite, 2012. **59**(2): p. 385-90.
74. Epel, E.S., et al., *The reward-based eating drive scale: a self-report index of reward-based eating.* PLoS One, 2014. **9**(6): p. e101350.
75. Johnson, B.A., N. Ait-Daoud, and J.D. Roache, *The COMBINE SAFTEE: a structured instrument for collecting adverse events adapted for clinical studies in the alcoholism field.* J Stud Alcohol Suppl, 2005(15): p. 157-67; discussion 140.
76. Kaplan, B.A., et al., *Automating scoring of delay discounting for the 21-and 27-item monetary choice questionnaires.* The Behavior Analyst, 2016. **39**(2): p. 293-304.
77. MacKillop, J., et al., *Further validation of a cigarette purchase task for assessing the relative reinforcing efficacy of nicotine in college smokers.* Experimental and clinical psychopharmacology, 2008. **16**(1): p. 57.
78. Epstein, L.H., et al., *Reinforcing value and hypothetical behavioral economic demand for food and their relation to BMI.* Eating behaviors, 2018. **29**: p. 120-127.
79. MacKillop, J. and J.G. Murphy, *A behavioral economic measure of demand for alcohol predicts brief intervention outcomes.* Drug and alcohol dependence, 2007. **89**(2-3): p. 227-233.
80. Jensterle, M., et al., *Does intervention with GLP-1 receptor agonist semaglutide modulate perception of sweet taste in women with obesity: study protocol of a randomized, single-blinded, placebo-controlled clinical trial.* Trials, 2021. **22**(1): p. 1-12.
81. Kampov-Polevoy, A.B., et al., *Association between sweet preference and paternal history of alcoholism in psychiatric and substance abuse patients.* Alcoholism: Clinical and Experimental Research, 2003. **27**(12): p. 1929-1936.
82. Weafer, J., S.H. Mitchell, and H. de Wit, *Recent translational findings on impulsivity in relation to drug abuse.* Current addiction reports, 2014. **1**(4): p. 289-300.
83. Brick, J., *Standardization of alcohol calculations in research.* Alcohol Clin Exp Res, 2006. **30**(8): p. 1276-87.
84. Martin, C.S., et al., *DEVELOPMENT AND VALIDATION OF THE BIPHASIC ALCOHOL EFFECTS SCALE.* Alcoholism-Clinical and Experimental Research, 1993. **17**(1): p. 140-146.
85. Bohn, M.J., D.D. Krahn, and B.A. Staehler, *DEVELOPMENT AND INITIAL VALIDATION OF A MEASURE OF DRINKING URGES IN ABSTINENT ALCOHOLICS.* Alcoholism-Clinical and Experimental Research, 1995. **19**(3): p. 600-606.
86. Johanson, C.E. and E.H. Uhlenhuth, *Drug preference and mood in humans: diazepam.* Psychopharmacology, 1980. **71**(3): p. 269-73.
87. MacKillop, J., et al., *Further validation of a cigarette purchase task for assessing the relative reinforcing efficacy of nicotine in college smokers.* Exp Clin Psychopharmacol, 2008. **16**(1): p. 57-65.
88. Curran, S.L., M.A. Andrykowski, and J.L. Studts, *Short form of the profile of mood states (POMS-SF): psychometric information.* Psychological assessment, 1995. **7**(1): p. 80.

Human Laboratory Screening of Semaglutide for Alcohol Use Disorder

Protocol Number: 21-1689

National Clinical Trial (NCT) Identified Number: Pending

Investigators:

Dr. Christian Hendershot (Principal Investigator)
Dr. Robyn Jordan (Co-Investigator)
Dr. Amanda Tow (Co-Investigator)
Dr. Sarah Dermody (Co-Investigator, Biostatistician)
Dr. Eric Claus (Co-Investigator)
Dr. Klara Klein (Co-Investigator)
Dr. Paul Fletcher (Other Significant Contributor)

Funded by: NIAAA

Version Number: v.1.5

22 Aug 2022

Summary of Changes from Previous Version:

Affected Section(s)	Summary of Revisions Made	Rationale
6.2.1, 6.2.3, Consent form	Updated medication storage protocol to remove on-site medication storage after the first medication dose	Change requested by IDS
1.3; 4.1; Questionnaire attachments	Updates to self-report instruments (added the Obsessive Compulsive Drinking Scale and Graded Pain Scale; updated images on the Food Purchase Task, updated question phrasing on Daily Diary questionnaire)	Refinement of study assessment instruments

4.1	Added Phone.Com as mechanism for sending text messages; updated study phone number on recruitment materials	Advised by UNC
5.2	Updated exclusion criterion to include current dependence on illicit drugs	Refinement of exclusion criteria
4.1, Consent	Updates to laboratory alcohol sessions: we now note that participants will be able to consume their preferred beverage at the self-administration sessions; we have removed the mandatory priming drink (which reduces the maximum potential peak blood alcohol concentration); we added a transdermal alcohol monitor (wristband) as a supplementary measure to breath readings; aadded information that participants will be eligible for reimbursement of transportation home.	To increase participant comfort/compliance in the alcohol self-administration sessions and bring the protocol in line with recently published procedures. The transdermal monitor is to supplement breath alcohol readings and allow a continuous readout of estimated alcohol concentration and to provide exploratory data.
Attachment (phone screen form)	Minor updates to phone screening form	Improving flow, wording and coverage of phone screening questions
Attachments (appointment reminder, medication and referral handouts)	Added participant information handouts (appointment reminder form, medication side effects/storage information, and treatment referral information.	To add additional helpful information related to appointments, medication, and resources for future treatment options if needed.

1 TABLE OF CONTENTS

STATEMENT OF COMPLIANCE 1
1 PROTOCOL SUMMARY 1
1.1 Synopsis 1
1.2 Schema 3
1.3 Schedule of Activities (SoA) 4
2 INTRODUCTION 5
2.1 Study Rationale 5
2.2 Background 6
2.3 Risk/Benefit Assessment 8
2.3.1 Known Potential Risks 8
2.3.2 Known Potential Benefits 10
2.3.3 Assessment of Potential Risks and Benefits 10
3 OBJECTIVES AND ENDPOINTS 11
4 STUDY DESIGN 13
4.1 Overall Design 13
4.2 Scientific Rationale for Study Design 22
4.3 Justification for Dose 22
4.4 End of Study Definition 22
5 STUDY POPULATION 23
5.1 Inclusion Criteria 23
5.2 Exclusion Criteria 23
5.3 Lifestyle Considerations 24
5.4 Screen Failures 25
5.5 Strategies for Recruitment and Retention 25
6 STUDY INTERVENTION 26
6.1 Study Intervention(s) Administration 26
6.1.1 Study Intervention Description 26
6.1.2 Dosing and Administration 27
6.2 Preparation/Handling/Storage/Accountability 28
6.2.1 Acquisition and accountability 28
6.2.2 Formulation, Appearance, Packaging, and Labeling 28

6.2.3 Product Storage and Stability 28
6.2.4 Preparation 29
6.3 Measures to Minimize Bias: Randomization and Blinding 29
6.4 Study Intervention Compliance 29
6.5 Concomitant Therapy 29
6.5.1 Rescue Medicine 30
7 STUDY INTERVENTION DISCONTINUATION AND PARTICIPANT DISCONTINUATION/WITHDRAWAL 30
7.1 Discontinuation of Study Intervention 30
7.2 Participant Discontinuation/Withdrawal from the Study 30
7.3 Lost to Follow-Up 31
8 STUDY ASSESSMENTS AND PROCEDURES 32
8.1 Efficacy Assessments 32
8.2 Safety and Other Assessments 32
8.3 Adverse Events and Serious Adverse Events 33
8.3.1 Definition of Adverse Events (AE) 33
8.3.2 Definition of Serious Adverse Events (SAE) 33
8.3.3 Classification of an Adverse Event 33
8.3.4 Time Period and Frequency for Event Assessment and Follow-Up 34
8.3.5 Adverse Event Reporting 35
8.3.6 Serious Adverse Event Reporting 35
8.3.7 Reporting Events to Participants 36
8.3.8 Events of Special Interest 36
8.3.9 Reporting of Pregnancy 36
8.4 Unanticipated Problems 36
8.4.1 Definition of Unanticipated Problems (UP) 36
8.4.2 Unanticipated Problem Reporting 37
8.4.3 Reporting Unanticipated Problems to Participants 37
9 STATISTICAL CONSIDERATIONS 37
9.1 Statistical Hypotheses 37
9.2 Sample Size Determination 38
9.3 Populations for Analyses 38
9.4 Statistical Analyses 38
9.4.1 General Approach 38
9.4.2 Analysis of the Primary Efficacy Endpoint(s) 39
9.4.3 Analysis of the Secondary Endpoint(s) 39
9.4.4 Safety Analyses 40

9.4.5 Baseline Descriptive Statistics 40
9.4.6 Planned Interim Analyses 40
9.4.7 Sub-Group Analyses 40
9.4.8 Tabulation of Individual participant Data 40
9.4.9 Exploratory Analyses 40

10 SUPPORTING DOCUMENTATION AND OPERATIONAL CONSIDERATIONS.40

10.1 Regulatory, Ethical, and Study Oversight Considerations 40

10.1.1 Informed Consent Process 40
10.1.2 Study Discontinuation and Closure 41
10.1.3 Confidentiality and Privacy 42
10.1.4 Future Use of Stored Specimens and Data 42
10.1.5 Key Roles and Study Governance 43
10.1.6 Clinical Monitoring 43
10.1.7 Quality Assurance and Quality Control 44
10.1.8 Data Handling and Record Keeping 44
10.1.9 Protocol Deviations 44
10.1.10 Publication and Data Sharing Policy 44
10.1.11 Conflict of Interest Policy 45

10.2 Additional Considerations........45

10.3 Abbreviations........45

10.4 Protocol Amendment History 48

11 REFERENCES........50

STATEMENT OF COMPLIANCE

(1) The trial will be carried out in accordance with International Conference on Harmonization Good Clinical Practice (ICH GCP) and the following:

- United States (US) Code of Federal Regulations (CFR) applicable to clinical studies (45 CFR Part 46, 21 CFR Part 50, 21 CFR Part 56, 21 CFR Part 312, and/or 21 CFR Part 812)

National Institutes of Health (NIH)-funded investigators and clinical trial site staff who are responsible for the conduct, management, or oversight of NIH-funded clinical trials have completed Human Subjects Protection and ICH GCP Training.

The protocol, informed consent form(s), recruitment materials, and all participant materials will be submitted to the Institutional Review Board (IRB) for review and approval. Approval of both the protocol and the consent form must be obtained before any participant is enrolled. Any amendment to the protocol will require review and approval by the IRB before the changes are implemented to the study. In addition, all changes to the consent form will be IRB-approved; a determination will be made regarding whether a new consent needs to be obtained from participants who provided consent, using a previously approved consent form.

1 PROTOCOL SUMMARY

1.1 SYNOPSIS

Title: Human Laboratory Screening of Semaglutide for Alcohol Use Disorder

Study Description: This is an early-Phase II human laboratory trial using a randomized, placebo-controlled, dose-ranging design to investigate the effects of semaglutide (Ozempic®), a GLP-1 receptor agonist, on laboratory alcohol responses and consumption, naturalistic alcohol and cigarette consumption, and weight loss in smokers with alcohol use disorder (AUD). Participants will attend weekly visits while semaglutide dosage is increased to 1.0mg over a period of approximately 10 weeks. Participants will attend weekly visits for medication or placebo administration. At scheduled intervals, participants will complete 4 laboratory sessions involving alcohol self-administration and alcohol challenge to characterize medication effects on alcohol-related outcomes.

Objectives: Primary Objective: To examine effects of semaglutide vs. placebo on laboratory alcohol intake during a validated alcohol self-administration medication screening procedure.

Secondary Objectives: To examine medication vs. placebo group differences in 1) laboratory responses to alcohol, and 2) changes in naturalistic alcohol use and cigarette consumption over the course of the treatment period.

	Tertiary Objectives: To generate preliminary data on weight loss, HbA1c reductions and alcohol elimination among participants with AUD during treatment with a GLP-1 receptor agonist.
**Endpoints:**	Primary Endpoint: Change in amount of alcohol consumed during a validated laboratory alcohol self-administration protocol (primary endpoint). The specific effect of interest is the change in laboratory consumption from baseline to the maintenance dose (.5mg) as a function of medication condition (semaglutide vs. placebo) (i.e., treatment x time interaction).<br><br>Secondary Endpoints: Secondary endpoints will include laboratory measures of alcohol and cigarette demand (behavioral economic indices of drug motivation) and subjective responses to alcohol during an alcohol challenge procedure, and changes in naturalistic alcohol use and cigarette smoking over the treatment period. Within-person changes in these outcomes before vs. after treatment (baseline vs. 0.5mg) will be compared as a function of medication condition.
**Study Population:**	Participants who meet criteria for mild or moderate alcohol use disorder/AUD (N=48) and smoke cigarettes daily will be enrolled. Participants will be recruited from the Triangle area (community and hospital settings). Participants must not be seeking current treatment for alcohol or smoking cessation.
**Phase:**	Phase II
**Description of Sites/Facilities Enrolling Participants:**	This is a single-site study (UNC-Chapel Hill). The project will be based at the Bowles Center for Alcohol studies and the Department of Psychiatry, with clinical operations taking place at Eastowne Clinical Research Unit and CTRC.
**Description of Study Intervention:**	Semaglutide (Ozempic®) will be administered according to the FDA-approved schedule, which begins with a starting dose of 0.25mg once per week for four weeks. At week five, dosage is increased to 0.5mg/week for a subsequent four weeks (maintenance dose). Following four weeks at the maintenance dose, dosage will be increased to 1.0mg provided the patient has tolerated the medication. Semaglutide is administered subcutaneously via the Ozempic® pen, a cartridge-based device that calibrates medication delivery. The placebo condition will consist of weekly sham doses, which will involve a brief needle insertion (without an injection).
**Study Duration:**	Up to 13 months.
**Participant Duration:**	Up to 40-50 hours total (including in-person visits and remote questionnaires) over a period of approximately 12 weeks.

1.2 SCHEMA

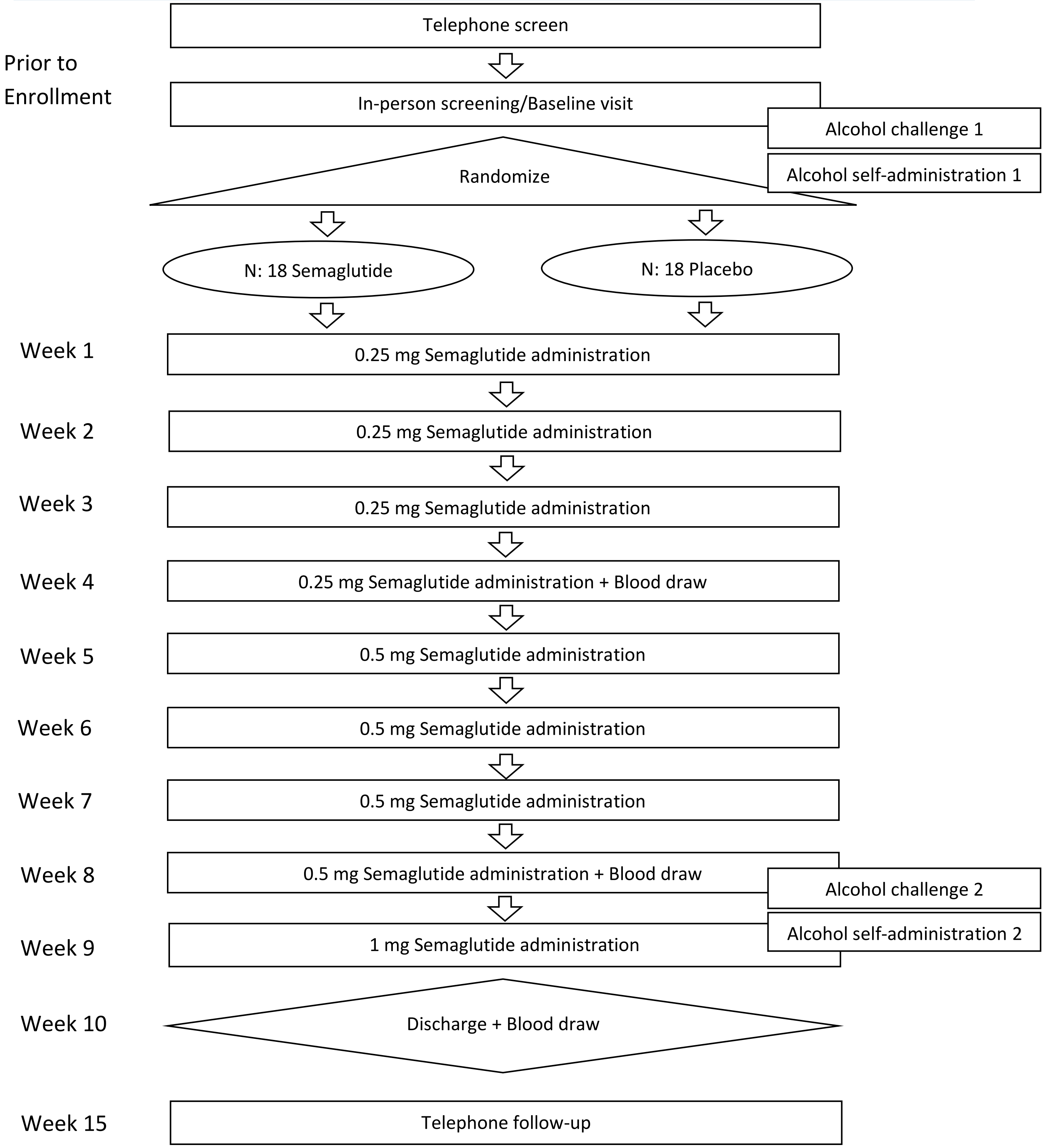
Telephone screen
Prior to Enrollment
In-person screening/Baseline visit
Alcohol challenge 1
Randomize
Alcohol self-administration 1
N: 18 Semaglutide
N: 18 Placebo
Week 1
0.25 mg Semaglutide administration
Week 2
0.25 mg Semaglutide administration
Week 3
0.25 mg Semaglutide administration
Week 4
0.25 mg Semaglutide administration + Blood draw
Week 5
0.5 mg Semaglutide administration
Week 6
0.5 mg Semaglutide administration
Week 7
0.5 mg Semaglutide administration
Week 8
0.5 mg Semaglutide administration + Blood draw
Alcohol challenge 2
Week 9
1 mg Semaglutide administration
Alcohol self-administration 2
Week 10
Discharge + Blood draw
Week 15
Telephone follow-up

1.3 SCHEDULE OF ACTIVITIES (SOA)

**Procedures and Questionnaires**	Telephone Screening	Screening/Baseline	Study Visit 1 – 3 Target Day 7, 14, 21	Study Visit 4 Target Day 28	Study Visit 5 – 7 Target Day 35, 42, 49	Study Visit 8 Target Day 56	Study Visit 9 Target Day 63	Discharge Target Day 70	Telephone follow-up Target Day 77, 105	Alcohol Challenge Target Day -10, 58	Alcohol Self-Admin. Target Day -12, 60
Telephone screening	X										
Administer 0.25 mg Semaglutide or placebo			X	X							
Administer 0.5 mg Semaglutide or placebo					X	X					
Administer 1.0 mg Semaglutide or placebo							X				
Breath Alcohol Concentration (BrAC) Test		X	X	X	X	X	X	X		X	X
Urine Drug Screen		X								X	X
Expired carbon monoxide (CO)		X	X	X	X	X	X	X		X	X
Locator		X									
Timeline Follow-Back (TLFB)		X	X	X	X	X	X	X			
BAC		X	X	X	X	X	X	X		X	X
Blood Draw		X		X		X		X			
Blood glucose – finger prick		X	X		X		X			X	X
Pregnancy (females only)		X	X	X	X	X	X	X		X	X
BMI		X	X	X	X	X	X	X		X	X
Height		X									
Vitals (HR, BP)		X	X	X	X	X	X	X		X	X
Weight		X	X	X	X	X	X	X		X	X
Temperature		X									
Demographics Form		X									
Structured Clinical Interview for DSM-5		X									
MINI Neuropsychiatric Interview		X									
Medical and Substance Use History Interviews		X									
Prediction of Alcohol Withdrawal Severity Scale (PAWS)		X									
Short Alcohol Withdrawal Scale (SAWS)		X								X	X
Saliva Collection for DNA and nicotine metabolite		X									
Case Report Forms (CRFs)	X	X	X	X	X	X	X	X	X	X	X
Alcohol Use Disorder Identification Test (AUDIT)		X									
Alcohol, Cigarette, and Food Purchase Test		X								X	X
Self-Report of the Effects of Alcohol Questionnaire (SRE)		X									
Fagerstrom Test for Nicotine Dependence (FTND)		X									
Nicotine Dependence Syndrome Scale (NDSS)		X									
Penn Alcohol Craving Scale (PACS)		X	X	X	X	X	X	X		X	X
Tiffany Questionnaire of Smoking Urges (TQSU)		X	X	X	X	X	X	X		X	X
Wisconsin Smoking Withdrawal Scale (WSWS)		X	X	X	X	X	X	X		X	X
Alcohol and Smoking Contemplation		X	X	X	X	X	X	X			
Three Factor Eating Questionnaire (TFEQ)		X	X	X	X	X	X	X		X	X
Monetary Choice Questionnaire (MCQ)		X	X	X	X	X	X	X		X	X
Cigarette Abstinence Self-Efficacy (CASE)		X	X	X	X	X	X	X		X	X
Alcohol Abstinence Self-Efficacy (AASE)		X	X	X	X	X	X	X		X	X
Inventory of Drinking Situations (IDS)		X									
Impaired Control Scale (ICS)		X									
Barratt Impulsivity Scale (BIS-11)		X									
UPPS Impulsive Behavior Scale (UPPS)		X									
Systematic Assessment for Treatment Emergent Effects (SAFTEE)		X	X	X	X	X	X	X	X	X	X

Reward Based Eating Drive Scale (R-BEDS)		X	X	X	X	X	X	X		X	X
Simplified Nutritional Appetite Questionnaire (SNAQ)		X	X	X	X	X	X	X		X	X
Control of Eating Questionnaire (CEQ)		X	X	X	X	X	X	X		X	X
Wisconsin Inventory of Smoking Dependence Motives (WISDM)		X									
Sweet Taste Preference Test		X						X			
Center for Epidemiological Studies Depression Scale (CES-D)		X									
Reward Probability Index – Environmental Reward Observation Scale (RPI-EROS)		X									
Toronto Alexithymia Scale (TAS-20)		X									
Profile of Mood States – Short Form (POMS-SF)										X	X
Biphasic Alcohol Effects Scale (BAES)										X	X
Alcohol Urge Questionnaire (AUQ)										X	X
Drug Effects Questionnaire (DEQ)										X	X
Obsessive-Compulsive Drinking Scale		X									
Graded Pain Scale		X									

2 INTRODUCTION

2.1 STUDY RATIONALE

Pharmacotherapy development is a critical objective for reducing health and societal burdens associated with alcohol use disorder (AUD) [1-3]. Despite advances in this area, medication development remains a slow- moving endeavor with a protracted path from drug discovery to market [2-4]. Given the high degree of heterogeneity in AUD clinical presentation and treatment response, it is recognized that no single medication will be effective for all patients [5]. Therefore, the National Institute on Alcohol Abuse and Alcoholism (NIAAA) medication development strategy specifies the long-term objective of establishing a repertoire of therapies to facilitate targeted interventions for specific AUD subgroups [3, 5, 6]. Strategies for expediting the study of novel treatments are central to these aims [3, 5, 6].

Epidemiological and clinical evidence shows that cigarette smokers comprise a sizable AUD subgroup with disproportionately high health burdens [7, 8] Alcohol and cigarette use are two of the three leading causes of preventable mortality worldwide, jointly accounting for an estimated seven million deaths per year [9]. Population-based research shows that cigarette consumption increases as a function of alcohol use severity [7]. In one large epidemiological study, nearly half (44.6% of men, 47.3% of women) of those with alcohol dependence also met criteria for nicotine dependence [7]. Co-use of alcohol and cigarettes predicts synergistic increases in risk for numerous disease conditions [8, 10, 11], including esophageal and laryngeal cancers [12, 13]. Given these health burdens, increasing effort has been allocated to developing targeted interventions for smokers with AUD [8, 11, 14, 15].

In the absence of FDA-approved medications for concurrent alcohol and nicotine addiction, identifying candidate drugs for this indication is a priority [11, 15, 16]. Several recent studies have examined whether FDA- approved AUD and smoking cessation drugs, either alone or in combination, can facilitate joint reductions in alcohol and cigarette use [11]. For instance, naltrexone reduces alcohol consumption in heavy-drinking smokers [17, 18], and in some cases reduces cigarette consumption [17, 19]. Recent findings further suggest that naltrexone's efficacy for reducing drinking is stronger in AUD participants

who smoke relative to non-smokers [20, 21]. However, a Cochrane review [22] and a large, prospective AUD trial [19, 23] concluded that naltrexone does not appear to reduce cigarette smoking [11, 23].

Varenicline has also been tested as a candidate therapy for smokers with heavy drinking or AUD. Informed by early findings that varenicline reduced alcohol self-administration in a human laboratory trial [16], large-scale clinical trials supported efficacy of varenicline for reducing alcohol consumption in heavy-drinking smokers [24, 25] and those with AUD [26]. Recent laboratory-based studies have also examined the combination of varenicline and naltrexone as a novel treatment [27, 28]. Because those with concurrent alcohol and nicotine addiction reflect a high-priority subgroup, further efforts are needed to screen candidate treatments for this population [8, 11, 28] . As reflected in these recent trials, and as noted explicitly under the NIAAA medication development framework [1-3], drug repurposing strategies can significantly expedite new treatment approaches by capitalizing on therapies that have already passed the extensive regulatory steps required for FDA approval.

Compounds with preclinical evidence for reducing both alcohol and nicotine intake are particularly good candidates for investigating new treatments for heavy-drinking smokers. Accumulating evidence suggests that *Glucagon-like peptide 1* (GLP-1) receptor agonists may have promise in this respect. GLP-1 is an incretin hormone produced and secreted both peripherally (in the intestines) and centrally (in the hindbrain) [29]. GLP-1 is stimulated following food consumption and stimulates satiety and decreases in blood glucose. As a peptide with both hormonal and neurotransmitter functions, GLP-1 plays a role in the "gut-brain axis," which has recently been invoked as a target for addiction therapies [30]. GLP-1 agonist medications were designed to mimic the function of the endogenous GLP-1 peptide, with the benefit of a much longer half-life than endogenous GLP-1. Based on their glucose-lowering and appetite-reducing properties, GLP-1 agonists were developed as anti-diabetic therapies.

Several GLP-1 receptor agonists are now in widespread use as treatments for type II diabetes (T2D), including exenatide, liraglutide, lixisenatide, albiglutide, dulaglutide and semaglutide. These medications have also proven efficacious for promoting weight loss, reducing glycated hemoglobin (HbA1c), and reducing risk for some cardiovascular events [29]. Although the primary indication of GLP-1 agonists is T2D, these therapies are increasingly being studied for other indications in non-diabetic patients, including obesity/weight management.

Of note, GLP-1 agonists require subcutaneous administration, typically by way of a pre-filled medication cartridge with a micro-needle attachment, which allows for easy self-administration in the home without assistance from medical staff. Semaglutide is a recently approved (2017) FDA-approved GLP-1 agonist. A new oral formulation of semaglutide was approved by the FDA in 2019, making semaglutide the first drug in its class to be available in both subcutaneous and oral formulations.

2.2 BACKGROUND

Accumulating evidence from preclinical studies suggests that GLP-1 agonists reduce alcohol and other drug intake and/or reinstatement in animal models. In a recent review of 17 preclinical studies [31], all studies reported significant effects of GLP-1 agonists on indices of drug motivation and intake, with the evidence being most robust for alcohol [30, 31]. Notably, the effects of GLP-1 agonists on drug intake appear to extend to several addictive drugs, including nicotine [32]. These findings have led to recent studies aiming to investigate the efficacy of GLP-1 agonists as candidate therapies for reducing intake or reinstatement of alcohol, nicotine, and other drugs (e.g., cocaine). Although several human trials are

underway in various regions, no RCTs have been published to date, and human data on the efficacy of GLP-1 agonists for reducing drug intake in substance-using populations is needed.

Although the precise mechanisms mediating the effects of GLP-1 agonists on alcohol-related behaviors remain to be clarified, it has been suggested that cholinergic-dopaminergic pathways are involved in altering the reward value of alcohol following exposure to GLP-1 agonists [30]. Additionally, GLP-1 agonists appear to exert their effects in part by way of interactions with the serotonin (5-HT) system, including interactions with 5-HT2C receptors. Specifically, GLP-1 agonists appear to induce serotonin release, and serotonin signaling appears critical to the efficacy of the medications [33, 34]. Some evidence also suggests that 5-HT2C receptors appear necessary for the efficacy and satiety-inducing effects of GLP-1 agonists [35, 36].

Of the approved FDA-approved GLP-1 agonists, most studies have focused on exenatide, an older GLP-1 agonist first approved in 2005. As noted, semaglutide is the newest GLP-1 agonist and the first available in both subcutaneous and oral formulations. Some evidence suggests superiority of semaglutide relative to other GLP-1 agonists with respect to efficacy for glycemic control [37]. Although clinical trials of GLP-1 receptor agonists in participants with substance use disorder are now underway, no studies (to our knowledge) are currently underway to examine semaglutide as a treatment for alcohol use disorder, or for the joint indication of alcohol use disorder and tobacco use disorder. Demonstrating tolerability and initial efficacy of semaglutide for these populations could ultimately allow for greater clinical flexibility if GLP-1 agonists prove efficacious for these populations, given that semaglutide is available in both subcutaneous and oral formulations.

Human laboratory methods for Phase II medication screening

As emphasized in the NIAAA medication development strategy [1, 4, 38], human laboratory methods play a pivotal role in drug development by providing a bridge from preclinical studies and full-scale Phase II/III trials [39-43]. Importantly, laboratory studies can expedite drug development by providing an efficient, cost-effective, and rigorous option for “early-Phase II” medication screening, thereby informing priorities for larger and more costly randomized trials. For example, we recently published meta-analytic evidence that human laboratory studies of naltrexone, when examined in aggregate, yield conclusions highly comparable to the aggregate data from large-scale randomized trials, reinforcing the notion that laboratory trials offer a valid and efficient approach for estimating the potential efficacy of candidate therapeutics [40]. Other advantages of laboratory trials include the ability to study putative treatment mechanisms under controlled conditions, and the ability to quickly generate initial data on drug tolerability in the clinical population of interest [1, 44].

Phase II pharmacotherapy trials for alcohol use disorder often rely on alcohol administration procedures to study medication effects on acute responses to alcohol in human laboratory settings [40]. Controlled alcohol exposure offers a means of testing medication effects on subjective response to alcohol (e.g., stimulation, sedation) and alcohol-induced craving. These subjective responses to alcohol are targets of existing pharmacotherapies, such as naltrexone [40]. Alcohol self-administration has also been used as a laboratory model of alcohol motivation, and has successfully been used to screen candidate AUD therapies [40]. Some have argued that alcohol self-administration should serve as the central endpoint in human laboratory studies, and validated procedures exist for modeling alcohol self-administration safely in the laboratory [16] [40]. More recently, behavioral economic measures have also been utilized as a means of studying drug motivation in laboratory contexts, and appear sensitive for detecting experimental manipulations on alcohol-related motivation. For example, *alcohol demand* indexes the

relative reinforcing value of alcohol at a particular point in time [45]. Measures of drug demand, such as the alcohol purchase task (APT) and cigarette purchase task (CPT), are considered objective and sensitive experimental assays of drug motivation [45]. The APT predicts frequency of heavy drinking days after treatment [45], and is sensitive to temporal changes in alcohol's relative reinforcing efficacy during drug administration [46]. Notably, initial evidence suggests that these measures are sensitive to the effects of pharmacological treatments, including naltrexone and varenicline [47, 48]. Measures of drug demand offer an alternative to studying self-administration in the laboratory, complementing measures of subjective drug responses.

One limitation of many laboratory-based pharmacotherapy trials is the tendency to focus on heavy drinkers without AUD, rather than participants who report symptoms of the disorder [49, 50]. A recent systematic review found evidence that recruiting non-clinical samples for laboratory trials can potentially limit the ability of laboratory findings to generalize to larger clinical trials, perhaps undermining translational research efforts [51]. While ethical considerations normally preclude laboratory alcohol administration methods with treatment-seeking AUD participants [50], studies of *non-treatment-seeking* AUD participants are ethically justified, and can provide important data on medication effects and safety in the target clinical population [52]. Additionally, focusing on participants with *non-severe* (i.e., mild or moderate) AUD can ensure maximal safety when studying this population by eliminating the likelihood of clinically significant withdrawal or alcohol-related medical complications. Our team has ample prior experience with alcohol administration procedures in both clinical and non-clinical populations; we will utilize these methods to study the potential efficacy of semaglutide in participants with mild-to-moderate AUD.

2.3 RISK/BENEFIT ASSESSMENT

2.3.1 KNOWN POTENTIAL RISKS

Semaglutide (Ozempic®) received FDA approval in 2017 for use as an adjunct to diet and exercise to achieve glycemic control in adults with type 2 diabetes mellitus (T2D). Semaglutide received FDA approval based on the results of 25 completed trials with the goal of providing results that would be applicable to a broad type II diabetes (T2D) population, including the elderly, patients with renal impairment, and patients at high cardiovascular risk. These trials involved > 9,300 participants, of whom >5,700 were treated with semaglutide. In these trials, treatment with semaglutide alone was efficacious for reduction in hemoglobin A1C (HbA1c) below 7% (e.g., 73% of participants had HbA1c <7% at week 30, compared to 28% of placebo-treated patients). Significant body weight reductions were also reported, with the weight loss achieved at end-of-treatment ranging from -1.43 to -4.28 kg (corresponding to 2.3–4.9%). Weight reductions were evident after 4 weeks of treatment, and persisted up to 104 weeks [75]. The peak plasma concentration of semaglutide is reached 1-3 days post-dose. Steady-state exposure is achieved following 4-5 weeks of once-weekly administration, and elimination half-life is approximately 1 week.

<u>Risks related to medication.</u> The most commonly reported side effects/adverse events (AEs) are gastrointestinal, with nausea being the most frequently reported symptom. The majority of reports of nausea (0.5 mg dose vs placebo; 15.3 vs. 6.1%), vomiting (5.0 vs 2.3%), and/or diarrhea (8.5 vs 1.9%) occur shortly following a dose escalation. Abdominal pain (7.3 vs 4.6%) and constipation (5.0 vs 1.5%) were also reported [76]. In studies ranging from 30-56 weeks in duration, the discontinuation rate due

to these gastrointestinal AEs was ranged from 5-13%, with this number increasing during longer treatment durations [77].

Rarer AEs (occurring at a frequency of <5%) included symptomatic hypoglycemia (≤70 mg/dL glucose threshold), which occurred in 1.6% of semaglutide-treated participants versus 0% placebo participants; dyspepsia (3.5 vs 1.9%); eructation (2.7 vs 0%); and gastroesophageal reflux disease (1.9 vs 0%). In placebo-controlled trials, injection site reactions (e.g., discomfort, erythema) were reported in only 0.2% of semaglutide-treated patients, suggesting good tolerability of subcutaneous administration. Patients exposed to semaglutide had a mean increase from baseline in amylase of 13% and lipase of 22%. Cholelithiasis was reported in 1.5% of treated patients. The 0.5 mg dose resulted in a mean increase in heart rate of 2 to 3 beats per minute, while there was a mean decrease in heart rate of 0.3 beats per minute in placebo-treated patients. Hypersensitivity reactions (e.g., anaphylaxis, angioedema) have been reported with GLP-1 receptor agonists. [76]. Other adverse reactions with a frequency of >0.4% associated with semaglutide include fatigue, dysgeusia and dizziness [76].

Rodent studies have shown an increase in the incidence of thyroid C-cell tumors after lifetime exposure of semaglutide at clinically relevant plasma levels. However, several studies in T2D patients taking GLP-1R agonists have shown no increase in neoplasm risk, even after several years of use [78]. Nevertheless, a personal or family history of medullary thyroid cancer or multiple endocrine neoplasia 2A or 2B is considered a potential risk factor for taking the medication, and will be an exclusionary criterion.

In a 2-year trial, acute pancreatitis was observed in 8 semaglutide-treated patients (0.27 cases per 100 patient years) and 10 placebo-treated patients (0.33 cases per 100 patient years). One case of chronic pancreatitis was confirmed in a semaglutide-treated patient. History of pancreatitis will be an exclusion factor, and study participants receiving semaglutide will be observed for signs and symptoms of pancreatitis (via blood monitoring of serum amylase and lipase). If pancreatitis is suspected, semaglutide will be discontinued, and appropriate management recommended.

Other rare events include postmarketing reports of acute kidney injury and worsening of chronic renal failure, which may sometimes require hemodialysis, in patients receiving long-term treatment with GLP-1 receptor agonists. Some of these events have been reported in patients without known underlying renal disease. Most of these events occurred in patients who had experienced nausea, vomiting, diarrhea, or dehydration. Therefore, we will monitor renal function by including a measure of serum creatinine at baseline and at subsequent visits involving blood samples.

Although most safety data on semaglutide comes from trials with T2D participants, there are now a number of randomized trials underway examining GLP-1 receptor agonists in participants with substance use disorder, including trials on tobacco, cocaine, alcohol, cannabis, and opioids. A full list of registered randomized trials can be found by searching for "GLP-1" at ClinicalTrials.gov. This study will be the first to our knowledge to focus specifically on heavy-drinking smokers. The use of a short-term medication screening protocol (lasting roughly 10 weeks) will provide an opportunity to demonstrate tolerability and generate data on side effect profiles in this population while minimizing any risks associated with longer-term medication exposure.

<u>Risks Related to Questionnaire and Interview Assessments.</u> There may be potential risks associated with assessment procedures. Participants may find the battery of psychiatric and psychological assessments tedious or intrusive. Participants may experience discomfort resulting from questions about personal histories or substance use patterns.

Participation in Alcohol Administration Sessions. Other potential risks include typical risks related to participation in alcohol administration studies. For example, it is possible that participants may experience dizziness/nausea from drinking during these visits, or symptoms the following day (e.g., headache). Our group has considerable experience with alcohol administration and self- administration procedures [80-84], and the safety precautions required in these studies. Across these studies, the incidence of adverse events such as vomiting has been exceptionally low. Our procedures will follow the NIAAA recommended guidelines for administering alcohol to human subjects (https://www.niaaa.nih.gov/Resources/ResearchResources/job22.htm). Briefly, the NIAAA guidelines include recommendations for administering alcohol to participants who meet criteria for AUD. In particular, we have taken measures to protect against ethical concerns by *excluding treatment-seekers* (or those engaged in an active quit attempt), as per the NIAAA recommended guidelines. Additionally, we will screen out participants with severe AUD (defined as 6 or more of 11 symptoms). This approach allows us to generate data relevant to a clinical population while minimizing risks.

Risks Related to DNA Collection. The purpose of collecting saliva for DNA in this study is to allow secondary analyses to examine whether medication effects might be different according to certain genetic factors. These analyses may also focus on genetic factors potentially associated with drug use or related behaviors, such as nicotine and alcohol metabolism, dopaminergic function, or impulsivity. Potential risks associated with DNA collection involve potential identification, and there may be risks with genetic (DNA) tests that are as yet unknown. Genes may be shown at some point in the future to be related to mental illnesses or tendency to addiction. Samples will be labeled with participant ID only, and analyzed at an outside laboratory. Samples will be de-identified and links with participant identifiers will be broken upon completion of the analyses. We will not conduct any whole genome sequencing of the DNA samples. Additionally, the consent form will state that participants have the option to opt out of DNA collection, and to request that their samples are destroyed at any time during the study. Participants will not receive any feedback from any of the genetic tests from study staff.

2.3.2 KNOWN POTENTIAL BENEFITS

The participants in this study are not expected to benefit directly from their participation. However, the knowledge gained from this study could lead to a better understanding of a medication with potential to treat alcohol and nicotine addiction, and potentially other substance use disorders.

2.3.3 ASSESSMENT OF POTENTIAL RISKS AND BENEFITS

Given the vast health implications of substance use disorder and the currently limited array of effective medications, it is imperative to pursue novel pharmacotherapeutic options. A key strategy in this regard is to “repurpose” medications that are already approved (and have proven safe) in the context of other health conditions. Given the sizable amount of research on GLP-1 receptor agonists in other clinical populations, coupled with preclinical evidence that these medications reduce motivation for alcohol, nicotine, and other drugs, evaluating this medication class in the proposed population is warranted. Given no available pharmacotherapies for the indication of concurrent alcohol and nicotine addiction, any evidence for potential efficacy in heavy-drinking smokers could have substantial implications. From this standpoint, the risk/benefit profile for testing semaglutide in a population of non-diabetic participants is favorable.

Although we anticipate the risk of adverse events to be low based on the safety profile of the medication, gradual dose escalation, and relatively short duration of treatment, several steps will be taken to track and report medication-related side effects. As described further in later sections of this application, participants will be monitored regularly by virtue of attending weekly lab visits for medication delivery, at which side effects will be assessed and clinical lab results monitored. Participants will be given a contact number for study staff in case of emergent side effects (and instructed to seek medical attention in the event of any serious side effects), and participants will be contacted by phone shortly after dose escalations to monitor symptoms. We expect some participants to experience nausea shortly following dose escalations, but that these symptoms will usually be transient.

Given the focus on a non-obese population, we will be particularly vigilant in monitoring changes in body weight and blood glucose, and will exclude participants at the outset with body weight or blood glucose levels below normal range, based on physician judgement. We will carefully monitor participants with glucose tests and weight measurements at regular study visits (see schedule of activities table), and will instruct participants to be vigilant for potential symptoms of hypoglycemia. Periodic blood tests to measure fasting glucose, fasting insulin, serum lipase and amylase, serum creatinine, and serum calcitonin will occur at baseline and regularly thereafter (i.e., at visits prior to dose escalations). All adverse events will be documented and reported in accordance with relevant policies, and the regulations of NIH. We have enlisted the TraCS DSMB to assist with safety monitoring of the study. The board will convene at the beginning of the study and at scheduled points to review study progress and safety data, and to review any SAEs and considerations related to stopping rules (described further below).

Considering the use of an FDA approved medication that has been deemed safe in the context of chronic administration for glycemic control, the risks related to medication use in this short-term screening trial are acceptable in comparison to the potential benefits of the proposed research (i.e., demonstrating potential efficacy of a new medication for substance use disorder).

Risks Related to Alcohol Administration Sessions. Alcohol administration procedures are commonly used to study risk factors for alcohol use disorder (AUD), and have proven capable of identifying successful pharmacotherapies [40]. As per the NIAAA guidelines for administration of alcohol in human studies, such studies are typically restricted to non-treatment-seeking participants in order to minimize ethical concerns, as will be the approach in this study. Further details on safety procedures are provided later in this document.

3 OBJECTIVES AND ENDPOINTS

OBJECTIVES	ENDPOINTS	JUSTIFICATION FOR ENDPOINTS
Primary		
To examine effects of semaglutide vs. placebo on alcohol self-administration under laboratory conditions.	Amount of alcohol consumed and breath alcohol concentration (co-primary endpoints) during a validated laboratory alcohol self-administration protocol. In this procedure, participants are provided the option of choosing between drinks and	Primary: This outcome is a laboratory assay of alcohol intake, and has been used in prior human laboratory studies to screen candidate medications, allowing comparisons with effect sizes for other medications. This session will be conducted during the standard

OBJECTIVES	ENDPOINTS	JUSTIFICATION FOR ENDPOINTS
	monetary reward. The comparison of interest is the difference between medication and placebo groups in the change in laboratory consumption between baseline and the maintenance dose of 0.5mg.	maintenance dose (0.5mg) to mimic the common clinical scenario.<br><br>Secondary: Subjective alcohol responses and alcohol/cigarette demand are validated measures of drug reward/motivation, and are known to be sensitive to medication effects. Given substantial individual differences in alcohol pharmacokinetics, a within-subjects analysis is preferable for these endpoints (such that each participant will serve as their own control). Laboratory sessions will take place at two time points: baseline and 0.5mg maintenance dose.
Secondary		
To examine group differences in 1) alcohol/cigarette demand and subjective responses to alcohol during alcohol administration, and 2) naturalistic drinking and smoking behaviors over the course of the 10-week treatment period.	Laboratory measures of subjective responses to alcohol, alcohol demand, and cigarette demand during controlled laboratory alcohol administration (lab sessions will take place at baseline and again after achieving the maintenance dose).<br><br>Number of standard drinks consumed and number of cigarettes consumed per day during the treatment phase (Weeks 0-10), as measured by a daily diary report.<br><br>Similar to the primary outcome, secondary outcome analyses will examine medication vs. placebo group differences in within-subjects changes in these outcomes over time (baseline to 0.5mg).	Laboratory measures of alcohol response and alcohol/cigarette demand are clinically valid indicators of drug motivation, and are demonstrated to be sensitive to medication effects. Investigating these outcomes under controlled conditions will allow us to test for a medication "signal" on measures of alcohol and nicotine motivation.<br><br>Daily measures of alcohol consumption (standard drinks per day) and cigarettes per day are selected to afford greater power for modeling change in consumption over the treatment period. Note that these outcomes are considered secondary because this Phase II trial involves non-treatment-seeking participants who are not attempting to reduce alcohol or use or cigarette smoking.
Tertiary/Exploratory		
Body weight, HbA1c, alcohol elimination.	Changes in body weight, HbA1c, and alcohol elimination will be examined as exploratory endpoints based on potential clinical relevance.	GLP-1 receptor agonists lower HbA1c and body weight in indicated populations (e.g., Type II diabetes, obesity). Given high co-occurrence of obesity and alcohol use disorder, reporting whether medication is

OBJECTIVES	ENDPOINTS	JUSTIFICATION FOR ENDPOINTS
		associated with these reductions in the present sample is clinically relevant. Data from the alcohol administration sessions will be used to estimate whether medication predicts changes in alcohol elimination during treatment, and whether changes in body weight account for any such differences. These outcomes are exploratory.

4 STUDY DESIGN

4.1 OVERALL DESIGN

Overview of Study Design.

The aim of this protocol is to translate preclinical findings on GLP-1 agonists by conducting an early-Phase II investigation of semaglutide in non-treatment-seeking participants with AUD, with a focus on human laboratory outcomes. Given preclinical evidence for the potential efficacy of GLP-1 agonists for reducing both alcohol and nicotine intake, and the need for treatments targeting both alcohol and nicotine addiction, this proposal focuses on cigarette smokers with AUD.

This is a single-site, Phase II, two-arm, double-blind (participants, outcome assessors), randomized, placebo-controlled, dose-ranging study. Following a baseline assessment eligible participants will be randomized to medication or placebo conditions by the IDS staff at a 1:1 ratio. The treatment period will be approximately 10 weeks, taking into account the dose escalation necessary for this medication. Those randomized to semaglutide will receive 0.25mg/week for 4 weeks (Weeks 1-4), and 0.5mg/week for 4 weeks (Weeks 5-8). Provided that participants have tolerated the medication , participants will receive a final dose of 1.0mg (Week 9), and will return for a discharge lab visit with repeat lab tests at Week 10, followed by a final monitoring assessment by phone scheduled approximately 6 weeks after the last medication dose (Week 15). Those in the placebo condition will receive sham placebo administration at each visit (described in further detail in later sections). Participants will attend weekly visits at Eastowne CRU or CTRC to receive medication or placebo doses.

In addition to the medication visits, participants will be asked to attend four laboratory visits: two involving alcohol self-administration (one prior to the first medication visit and one during the 0.5mg treatment phase) and two involving alcohol challenge (one prior to the first medication visit and one during the 0.5mg treatment phase). These sessions are designed to assess changes in alcohol responses over the course of the treatment period. Laboratory sessions are described in further detail below. Measurements of naturalistic alcohol and cigarette use will also be recorded throughout the treatment period. These assessments will be captured using a daily diary that will be provided to participants. Participants will receive an automated text message daily via Phone.com as a reminder to complete the diary. Additional self-report measures of substance use will be collected at study visits.

As summarized above, the primary objectives are to examine whether treatment with semaglutide alters alcohol self-administration (primary outcome) and laboratory measures of alcohol response and motivation (i.e., demand and responses to alcohol, secondary outcomes) during alcohol administration. We hypothesize that the semaglutide treatment (versus placebo) will be associated with reduced alcohol self-administration during a laboratory session conducted at the maintenance dose of 0.5mg. We further hypothesize that semaglutide will lead to reductions in alcohol demand, cigarette demand, and subjective responses to alcohol (e.g., reduced stimulation) during alcohol administration sessions.

The primary and secondary outcomes for this trial will be assessed during exposure to the standard maintenance dose (.5mg). While an extended treatment phase at the 1.0mg dose is beyond the scope of this small-scale trial, the inclusion of a brief (1-week) phase at 1.0mg is designed to provide initial data on tolerability and any changes on selected secondary outcomes (i.e., drinks per day and cigarettes per day) in this population. Capturing these data will help to support feasibility and pilot data for subsequent funding proposals with longer treatment intervals.

Secondary objectives include examining changes in naturalistic alcohol consumption and cigarette smoking during the treatment period. Although participants will not be attempting to reduce drinking or smoking in this trial, it is hypothesized that treatment with semaglutide will result in reductions in alcohol consumption (drinks per day) and cigarettes per day over the course of the treatment period. Tertiary/exploratory outcomes include examining changes in body weight, HbA1c, and alcohol elimination during the trial.

In total, participation in this study is estimated to take a maximum of up to 50 hours of combined time over a period of up to approximately 16 weeks beginning with initial screening. This includes a screening and baseline visit (up to 4 hours), up to 9 subsequent laboratory visits for medication administration and side effects monitoring (up to 1 hour each), a final discharge visit (Week 10), and final telephone check-in (Week 15). If necessary for safety reasons, (e.g., based on lab results), participants may be asked to attend (unplanned) visit(s) for follow-up labs at the discretion of the study physician. Participants will also complete four visits involving alcohol administration over the course of Weeks 0-10; these sessions may last up to 7-8 hours each. Participants also will receive calls from staff 2-3 days after each medication phase begins (placebo, 0.25mg, 0.5mg, 1.0mg) to check in on side effects (10 minutes per call), and will be asked to complete brief daily questionnaires via a daily diary with reminders sent via daily text message prompt (1-2 minutes per day).

A detailed summary of study procedures is provided below.

Detailed Study Procedures.

Following initial recruitment (described in section 5.5), participants will be asked to complete the following procedures:

a) <u>Telephone screen</u>

Initial screening will be conducted by telephone by trained research staff and will assess participant alcohol, cigarette, and drug use, medications, and basic medical inclusion/exclusion factors. Potential participants will be given a brief description of the study. The initial telephone screen is estimated to take roughly 15-30 minutes. Contact information will be collected from interested callers who pass the initial screen. Following the initial screen, participants will proceed to be booked for an informed consent and baseline interview session. The informed consent procedures

will be conducted remotely using a HIPAA-compliant virtual meeting platform (e.g., Webex, Zoom) and Docusign. Depending on participant and staff availability, the phone screening and informed consent/baseline interview can happen on the same day (after the screening call) or at a future date.

b) Screening and Baseline Data Collection Visits

At the informed consent session, a member of the research staff will review the study procedures and carry out informed consent procedures (including electronic signature via Docusign) prior to proceeding to the baseline interview. Following informed consent, the staff member will conduct a semi-structured interview to assess inclusion/exclusion criteria. A research assistant will use a structured interview to measure diagnostic and substance use screening criteria, including symptoms of alcohol use disorder; inclusion/exclusion factors related to medical history will also be assessed (see criteria listed above). The Structured Clinical Interview for DSM-V [53] will be used to assess AUD and substance use disorder symptoms. History of other psychiatric diagnoses and medication use will be assessed via self-report. The research assistant will also collect information on demographic factors, criteria related to current medical status and medications, current alcohol, tobacco and other substance use (see "Phone Screen" document), as well as substance use disorder symptoms (via select modules from the SCID interview) and symptoms other psychiatric symptoms (using the MINI screening module). Participants who appear eligible will be booked for an in-person visit, which will take place at the laboratory site (Medical Wings C), with some procedures (e.g., blood draw) booked at CTRC as necessary.

The in-person screening/baseline visit will include a brief review of informed consent to answer any additional questions, and a brief review of self-report and interview questions from the remote screening session. The Timeline Follow-Back (TLFB) [54] will be used to assess alcohol, cigarette and other drug use in the 90 days prior. The participant will also provide a breathalyzer screen. Participants who have a positive BAC (greater than 0.00) will be monitored until their BAC is reduced to a safe level (BAC<.03g%), in accordance with NIAAA guidelines (or to 0.00g% in the event that the participant drove to the session).

Participants who remain eligible after the interview will complete vitals and measurements (HR, BP, height, weight, temp), and a measure of expired alveolar carbon monoxide (CO). A baseline saliva sample for DNA analysis will also be collected. Additionally, participants will provide a urine sample and complete a blood draw (up to 50-60 ml) for a comprehensive metabolic panel (e.g., Na, K, Cl, CO2, BUN, Creatinine, Glucose, Calcium, Albumin, Total protein, Total bilirubin, AST, ALT, Alk Phos), baseline measure of HbA1c, baseline measure of nicotine metabolites (e.g., 3HC, COT), serum amylase and lipase, and complete blood count (e.g., Auto WBC, Corrected WBC, RBC, HGB, HCT, MCV, MCH, MCHC, RDW, MPV, Platelet). These procedures will be conducted by the research assistant, and the baseline blood draw will be taken by a team member with phlebotomy training. If there is no team member with phlebotomy training available that day, the blood draw may also be booked at CTRC. If needed, the blood draw and other baseline procedures may be booked as separate visits. On the day of the visit that includes the blood draw, participants will be instructed to arrive fasting for 12 hours (to allow for assessment of baseline HbA1c and lipids). If participants are not able to arrive fasting for the baseline session, the fasting lipids sample may be deferred or completed at the next possible visit. Blood samples will be sent to McClendon laboratory for testing. A portion of the blood sample will also be stored (Thurston Bowles building) for subsequent analysis (e.g., nicotine

metabolites). The saliva sample will also be stored at Thurston Bowles building. The urine sample will be used to test for the presence of several drugs (for example, heroin, cocaine, methamphetamines, opiates). (For females, the test will include a pregnancy screen.) If a participant tests positive for drugs other than cannabis (or if females screen positive for pregnancy), the participant will be excluded from the study.

Participants will complete baseline questionnaires, which will be collected on paper. A Demographic Screening Questionnaire will assess basic demographic factors (e.g., age, education, income). A Locator form will collect contact information for three individuals who can be contacted if a participant is unable to be reached by study staff for follow-up visits. Other baseline measures will include the Alcohol Use Disorders Identification Test (AUDIT) [55], Self-Report of the Effects of Alcohol Questionnaire (SRE) [56, 57], Fagerström Test of Nicotine Dependence (FTND) [58], and the Nicotine Dependence Syndrome Scale (NDSS) [59], Barratt Impulsivity Scale (BIS-11) [60], UPPS Impulsive Behavior Scale (UPPS) [61], Impaired Control Scale (ICS) [62], Center for Epidemiological Studies Depression Scale (CES-D) [63], Toronto Alexithymia Scale (TAS-20) [64], the Reward Probability Index (RPI) and the Environmental Reward Observation Scale (EROS) [65]. Baseline measures that will be repeated at subsequent visits will include the Timeline Followback (TLFB), the Penn Alcohol Craving Scale (PACS) and Tiffany Questionnaire of Smoking Urges (TQSU) [66], and the Wisconsin Smoking Withdrawal Scale (WSWS) [67]. To assess potential medication effects on changes in motivation and self-efficacy for resisting alcohol/cigarettes, we will use alcohol and smoking Contemplation Ladder items, Alcohol and Cigarette Abstinence Self-Efficacy (AASE/CASE) (these scales will be repeated at each visit) and the Inventory of Drinking Situations (IDS) scales [68-70]. Appetite and eating behavior will be assessed with the Simplified Nutritional Appetite Questionnaire (SNAQ) [71], Control of Eating Questionnaire (CEQ) [72], the Three Factor Eating Questionnaire (TFEQ) [73] and the Reward-Based Eating Drive Scale (R-BEDS) [74]. During the medication phases, side effects will be assessed with the SAFTEE questionnaire [75], a standard tool for alcohol clinical trials. Participants will also complete previously validated Purchase Tasks for alcohol, cigarettes, and food, and delay discounting will be assessed with the Monetary Choice Questionnaire (MCQ) [76-79]. These brief behavioral economic tasks assess perceived value of reinforcers by presenting hypothetical scenarios involving choices between the reinforcer (alcohol, cigarette, food) and monetary rewards; these measures will be completed at each visit. Finally a previously validated sweet taste preference test will be administered at baseline and again on the final visit, since this phenotype has been shown to be implicated in both food and alcohol reward, and may be modulated by a GLP-1 agonist. This test involves participants tasting solutions of sugar water prepared to reach .05, .1, .2, .42, and .83 molar sucrose, and then rating preference and intensity of the taste. The sweet taste test will be overseen by a research assistant using previously published protocols from prior projects at UNC [80, **Error! Hyperlink reference not valid.**].

The baseline questionnaires and interview assessments are projected to take approximately 3-4 hours combined. To accommodate potential scheduling/time constraints or delays at the baseline visit, some questionnaires that are not time-sensitive (for instance, personality questionnaires) may be deferred until a later lab visit as needed, at the discretion of the study team. All interview records and paper questionnaires will be stored in study binders in a secure location for subsequent entry into an electronic database for analysis. Patients will be given breaks as needed. Following the blood draw, participants will be offered a snack and bottled water (given the instructions to arrive fasting). If results of the baseline screening/medical

assessment indicate that the participant is tentatively eligible, they will be given a comprehensive orientation to the next phases of study, including the follow-up medication visits, the laboratory alcohol administration visits, and the daily surveys. Locator/collateral contact information will also be obtained. A Locator form will collect contact information for three individuals who can be contacted if a participant is unable to be reached by study staff for follow-up visits. Following the completed baseline assessments, the participant's screening summary will be reviewed for eligibility, including review of medical criteria and any medications that the participant is taking by the study physician. Lab results will be uploaded to the EMR and reviewed by the study physician prior to prescribing the medication. Potential interactions with semaglutide will be verified using an established online database such as Epocrates or Lexicomp. In the event of any uncertainties about medication interactions, participants will be asked to contact their primary care physician to confirm no concerns with taking semaglutide.

For eligible participants, the study physician will provide sign-off on all medical criteria (e.g., medical history, medications, lab results), and the PI will provide sign-off on other baseline criteria. Once participants have been confirmed as eligible, the physician will submit an order to start medication or placebo. Medication and placebo will be dispensed at CTRC (Week 1 visit) at a date coordinated with the participant. Participants will be advised to be vigilant for symptoms of hypoglycemia at the medical visit, and instructed to contact the study team to review any symptoms that emerge (regular glucose tests will also be obtained at follow-up visits, as noted in the schedule of assessments). If necessary, the medication review will take place via telephone or telemedicine, depending on medical team availability.

Participants' Week 1 visit will be scheduled with enough lead time to review lab results and exclude participants (if needed) prior to administering the study medication. Additionally, if clarification of medical criteria is needed at any point during or following the phone or in-person screening process, or at any subsequent stage of the study, the study physician may meet with the participant via a remote contact method (e.g., phone, Webex/Zoom, or telehealth) to clarify medical questions or discuss test results.

c) Daily Assessments.

Throughout the study, participants will be asked to complete a brief self-report survey each day that includes measures of 1) prior-day number drinks consumed; 2) number of cigarettes smoked; and 3) prior day peak craving/demand for alcohol and cigarettes. Participants will be provided with a paper booklet in which to enter responses and will be asked to bring the booklet to each visit. At each visit the research staff will collect the interim daily surveys for retention in the participant's study binder. Participants will be texted an automated text message each day during their participation in the study as a reminder to complete the daily survey. If preferred, participants can opt to receive an email link. (No daily survey data will be collected from participants via text or email.) At each study visit, a staff member will provide compensation for the prior week's surveys, and will review survey adherence and progress towards compensation for good adherence (described later), as a means of motivating adherence to self-report assessments. In the event a participant completes the daily assessments but forgets to bring the packet to a given visit, they will be asked to bring their packet to the following session. Participants will be asked to either a) complete the measures in person at the current lab visit, or b) to schedule a brief meeting with a research team member via Zoom or Webex in order to complete the questions (depending on scheduling and availability). These

approaches will be used in specific circumstances if needed (e.g., if the participants forgets to complete the daily assessments altogether, or will be unable to bring it to the next session).

d) Weekly Laboratory Visits.

Medication administration visits will occur at either the Eastowne Clinical Research Unit (CRU) or the CTRC. Following the baseline (Week 0) visit, participants will visit CRU or UNC Hospital/CTRC once weekly (Weeks 1-9), for subsequent medication or placebo doses, and for a final follow-up/discharge visit at Week 10. (In addition, the study physician may request an additional follow-up lab visit(s) at any point in the study to monitor side effects.) In addition, to accommodate events such as missed lab visits or scheduling difficulties, assessment procedures at a given weekly visit (e.g., questionnaires, lab tests) may be conducted within a 7-day window of the original target date, as necessary, to avoid unnecessary missing data. In addition to medication/placebo administration, follow-up visits will include 1) vitals, weight measurements, and a brief side-effects assessment, 2) a finger-stick glucose test (or fasting blood draw depending on the week, see study flow chart), 3) a brief calendar-based self-report assessment of interim alcohol and cigarette use (TLFB), and 4) a repeat CO measurement. These procedures are expected to take roughly 30-60 minutes in total, and will be conducted by a trained study team member and/or a member of the CRU/CTRC team (e.g., RN). All side effects reports, labs, and other medical test results (e.g., glucose tests) will be uploaded in Epic (e.g., lab tests) or sent electronically (e.g., self-reported medication changes) for review by the study physician. If side effects or lab results suggest that further assessment is indicated, a member of the medical team will advise the study team (and if necessary, contact the participant) to provide direction. At the Week 4, Week 8, and final discharge visits, participants will be asked to arrive fasting to allow for a blood draw for follow-up lab assays (identical to those described in the baseline procedures above). As with the baseline sample, a portion of each sample will be stored for follow-up analyses (e.g., nicotine metabolites).

e) Alcohol Sessions.

To evaluate effects of medication phase on laboratory measures of alcohol response and self-administration, participants will be asked to complete four laboratory visits over the course of the study. Two visits will occur between the Week 0 and 1 visits (baseline phase), and two will occur between the Week 8 and 9 visits (0.5 mg phase). Minor scheduling deviations will be accommodated as needed, provided that the participant has completed at least three weeks of the .5mg phase at the time of the second set of laboratory sessions.

During each of these sessions participants will be asked to report to the lab having received instructions to abstain from alcohol and recreational drugs for 24h, and not to consume food for 4 hours prior to arrival. Participants will also be advised not to drive to these study appointments. Participants will also be asked to schedule a smoking break just prior to arrival (to ensure that participants are not nicotine-deprived during testing) [16]. Upon arrival participants will provide breathalyzer and expired CO readings, as well as measures of body weight and vitals (HR, BP) and a finger-stick blood glucose test. Participants will also receive the Short Alcohol Withdrawal Scale (SAWS) to ensure that no significant withdrawal symptoms are present. Evidence of withdrawal symptoms will require a consultation with an addiction medicine physician (Dr. Tow or Dr. Jordan) prior to proceeding. (We except a low likelihood that this will happen because participants with severe AUD or history of withdrawal symptoms will be excluded). Female participants will also be asked to repeat the urine pregnancy screen at each alcohol session.

In the interim, participants will complete self-report questionnaires (e.g., Timeline Follow-Back to assess recent substance use). Participants will be seated in a comfortable recliner chair for the alcohol administration procedures. Participants will first complete baseline physiological readings (systolic and diastolic blood pressure, heart rate), and baseline measurements of self-report questionnaires (see below). Next, participants will complete one of two procedures (alcohol challenge, alcohol self-administration), dependent on the visit week. Specifically, the first alcohol challenge session (AC1) and the first self-administration session (SA1) will be scheduled to take place between Week 0 and 1. The second alcohol challenge session (AC2) and the first self-administration session (SA2) will be scheduled to take place between Week 8 and 9 (0.5mg treatment phase), allowing for minor scheduling adjustments as noted above. Prior to the self-administration session only (which is longer in duration), participants will consume a standardized, isocaloric meal in advance of the alcohol self-administration procedures, with a standardized wait time between completion of the meal and onset of beverage consumption. The two session types will be scheduled with at least one interim day scheduled between sessions when possible. For both sessions, participants will be instructed to expect to remain in the lab for a full day (e.g., 8 hours).

Alcohol Challenge Sessions (AC1, AC2)
The AC sessions are designed to generate data to estimate within-person changes in acute responses to alcohol as a function of medication phase (baseline vs. 0.5mg). Following baseline assessments (described above), participants will be asked to consume a pre-determined dose of alcohol calculated to achieve a target peak breath alcohol concentration (BrAC) of .06g%. This BrAC level reflects a moderate alcohol dose. Individualized doses will be calculated using established formulas [83] that account for determinants of total body water (e.g., height, weight, sex, age). Beverages will consist of 80-proof vodka combined with a non-alcoholic, sugar-free mixer at a 1:5 ratio. Beverages will be allocated to three equal portions in separate cups. Participants will be asked to consume the beverages at an even pace over 10 minutes. A separate placebo session is not incorporated for the AC sessions, because our primary interest is in examining *within-person* changes in laboratory alcohol responses as a function of medication dose (baseline vs. 0.5mg), and because our primary outcome is self-administration (described below). Although a limitation of this approach is the inability to dissociate expectancy from pharmacological effects in measuring subjective responses to alcohol, increasing the number of sessions to dissociate expectancy effects is not a major aim for this preliminary project.

After completion of the beverages, participants will wait 10 minutes (to allow an absorption period), then will complete an initial breath alcohol concentration (BrAC) reading. BrAC readings will be repeated at 10-minute intervals during the first hour of the session (peak BrAC is anticipated to occur around 30-40 minutes, on average, with variability across subjects). Participants will repeat the battery of self-report questionnaires (outlined below) and physiological assessments at three points on the ascending limb of the blood alcohol curve: upon the first BrAC reading that meets or surpasses .02g%, .04g%, .06g%. To account for the fact that some participants may not reach the .06g% target, the latter assessment will be conducted if/when it appears the participant will not reach the .06g% target (as evidenced by two consecutive descending BrAC readings after surpassing the .05g% mark).

Following completion of the last set of measures (.06g%), participants will then be escorted to a private room to begin the recovery phase, during which they will receive water and will be monitored, and take bathroom breaks as needed. During this time, BrAC measurements will

continue every 15 minutes. On the descending limb of the blood alcohol concentration curve, participants will complete follow-up measures and physiological readings at the first BrAC readings that meet or fall below .04g% and .02g%. Prior to discharge, participants will be asked to provide an additional blood glucose sample (via fingerpick) to assess any differential changes in glucose levels following alcohol administration as a function of medication phase. Participants will also complete the Food Purchase Task to assess motivation for food following alcohol administration. After this point, participants will be provided with a lunch and snacks to consume during the recovery period. Participants will be compensated and discharged once BrAC reading reaches .00g% (to allow for estimating alcohol elimination rate across sessions AC1 and AC2 as a function of medication group).

Subjective Measures. At baseline (prior to alcohol ingestion) and each subsequent assessment point noted above, participants will complete several measures. Measures of subjective alcohol effects will include the Biphasic Alcohol Effects Scale (BAES) [84], the Alcohol Urge Questionnaire (AUQ) [85] and Drug Effects Questionnaire (DEQ) [86]; the latter will be supplemented with additional items to assess potential effects related to medication (e.g., "tired", "confused", "nauseous", "dizzy", "sick", "drunk/intoxicated") [16]. The Alcohol Purchase Task [45] Cigarette Purchase Task [87] Food Purchase Task [78] and Monetary Choice Questionnaire [76]will be also administered at the corresponding intervals noted above. Changes in mood will be assessed using the Profile of Mood States – Short Form (POMS-SF) [88]. During the recovery period, participants will complete another battery (all measures noted above) once reaching .04g% and .02g% on the descending slope of the blood alcohol curve.

Alcohol Self-Administration Sessions (SA1, SA2).

Medication effects on laboratory alcohol consumption will be examined using a validated oral self-administration procedure. Pre-session instructions and arrival procedures/tests will be identical to those noted above. After completing baseline physiological and baseline questionnaires, participants will be presented with their preferred beverage (beer, wine, or mixed drink). Participants will elect when to begin drinking and will then complete a 2-hour *ad libitum* self-administration period. Participants will have access to their beverage at a volume calculated (based on the person's sex, weight, height, and age using established formulas) intended not to exceed a maximum predicted peak blood alcohol concentration .12g% over the consumption period of two hours (should the participant consume all of the beverages). Participants will be instructed to consume the amount of alcohol they wish (i.e., they are not required to drink all of the beverages). Participants will be seated in a comfortable chair/sofa and will view a neutral video (e.g., nature documentary) during the session. Beverages will be presented to the participant at the onset of the two-hour period, along with ice if requested. Participants will repeat rounds of self-report assessments (described above) at 30-minute intervals during the *ad libitum* phase (i.e., at 60, 90, and 120 min. after the end of the priming phase), with physiological assessments and BrAC readings taken at these intervals (or more frequently if indicated). The primary outcomes for this session are peak BrAC and volume of alcohol consumed.

Participants will then be escorted to a private room for the recovery period. Assessments during the recovery period will be identical to those described above. Participants will be monitored until being discharged when BrAC declines below .03% for two consecutive readings, consistent with NIAAA recommended guidelines. [52] As noted, participants will be instructed not to drive, and will be asked to arrange for a ride home from the study site. If a participant cannot arrange a ride home, they will be provided with reimbursement for a ride home via a rideshare service (e.g., Uber or Lyft) up to $25. If a participant reports having driven to a session, they will be required to remain at the site until BrAC is measured at 0mg% for two consecutive readings. At alcohol sessions, participants may also be provided with a wristband transdermal alcohol monitor (BACTrack Skyn) to wear during the session, which will provide a continuous readout of estimated alcohol concentration (measured as total alcohol concentration in sweat). Estimated transdermal alcohol concentration will not be a primary outcome -

as it is not an accurate measure of blood alcohol concentration - but will be collected for purposes of secondary data analyses. Specifically, we will record readouts from the transdermal device at regular intervals to compare to BrAC readings, which will inform our use of this device in future studies. Because we will only have one transdermal device available, it may not be used in all sessions (depending on availability).

<u>List of Study Assessment Measures (See Section 1.3 for assessment time points)</u>

1. Demographics form
2. Timeline Follow-Back (TLFB)
3. Structured Clinical Interview for DSM-5 Mental Disorders (select modules)
4. MINI Neuropsychiatric Interview (select modules)
5. Sweet Taste Preference Test
6. Alcohol Use Disorder Identification Test (AUDIT)
7. Self-Report of the Effects of Alcohol Questionnaire (SRE)
8. Center for Epidemiological Studies Depression Scale (CES-D)
9. Toronto Alexithymia Scale (TAS-20)
10. Barratt Impulsivity Scale (BIS-11)
11. UPPS Impulsive Behavior Scale (UPPS)
12. Reward Probability Index – Environmental Reward Observation Scale (RPI-EROS)
13. Impaired Control Scale (ICS)
14. Fagerstrom Test for Nicotine Dependence (FTND)
15. Nicotine Dependence Syndrome Scale (NDSS)
16. Penn Alcohol Craving Scale (PACS)
17. Tiffany Questionnaire of Smoking Urges (TQSU)
18. Wisconsin Smoking Withdrawal Scale (WSWS)
19. Wisconsin Inventory of Smoking Dependence Motives (WISDM)
20. Alcohol and Smoking Contemplation Ladder
21. Three Factor Eating Questionnaire (TFEQ)
22. Reward-Based Eating Drive Scale (R-BEDS)
23. Control of Eating Questionnaire (CEQ)
24. Simplified Nutritional Appetite Questionnaire (SNAQ)
25. Monetary Choice Questionnaire (MCQ)
26. Cigarette Abstinence Self-Efficacy (CASE)
27. Alcohol Abstinence Self-Efficacy (AASE)
28. Inventory of Drinking Situations (IDS)
29. Biphasic Alcohol Effects Scale (BAES)
30. Alcohol Urge Questionnaire (AUQ)
31. Drug Effects Questionnaire (DEQ)
32. Alcohol Purchase Task (APT)
33. Cigarette Purchase Task (CPT)
34. Food Purchase Task (FPT)
35. Profile of Mood States – Short Form (POMS-SF)
36. Systematic Assessment for Treatment Emergent Effects (SAFTEE)
37. Prediction of Alcohol Withdrawal Severity Scale (PAWS)
38. Short Alcohol Withdrawal Scale (SAWS)
39. Obsessive Compulsive Drinking Scale
40. Graded Pain Scale

Analysis schedule. Analyses of side effect frequency and AE/SAE frequency will be conducted prior to scheduled DSMB meetings. Other analyses will be conducted following the completion of the trial, or as otherwise required to fulfill DSMB requests or other reporting requirements.

4.2 SCIENTIFIC RATIONALE FOR STUDY DESIGN

This short-term trial is intended to produce initial data on the effects of semaglutide in non-treatment-seeking participants with AUD, with an emphasis on human laboratory outcomes. The dose escalation schedule is based on the standard clinical regimen, which is intended to minimize side effects common to GLP-1 agonists (i.e., nausea). Participants in the placebo group will complete an identical sequence while receiving sham subcutaneous injections (a small needle stick using a micro-needle of the same size, without an injection). This placebo condition is selected due to the absence of an available matching placebo cartridge from the manufacturer (see section 4.3). All participants will complete laboratory alcohol administration sessions at two time points, corresponding with the baseline and 0.5mg phases. This design allow for within-subjects analyses for laboratory outcomes that are best examined at the within-subject level (owing to high inter-individual variability in alcohol pharmacokinetics). Overall, this design is intended to maximize feasibility and sample size while also accommodating the extended dosage titration period required of GLP-1 agonists. Findings from this trial will be used to justify a larger, placebo-controlled Phase II clinical trial application via the R01 mechanism.

4.3 JUSTIFICATION FOR DOSE

Semaglutide (Ozempic®) will be administered according to the FDA-approved schedule, which begins with a starting dose of 0.25mg (for titration purposes) once per week for four weeks. At week five, dosage is increased to 0.5mg/week (the initial first maintenance dose) for a subsequent four weeks, at which point dosage can be increased further (1.0mg) if indicated (and presuming that the 0.5mg dose has been tolerated). The intent for this initial trial is to mimic the typical clinical scenario over the first several weeks of medication titration. Although primary analyses will focus on the 0.5mg dosage, participants will receive one week of 1.0mg treatment at the conclusion of the 0.5mg phase, in order to collect initial data on side effects and naturalistic drinking/cigarette use at this dosage in the current population. Participants will be monitored carefully and will not be escalated to the next dose if experiencing persistent side effects indicative of poor tolerability.

Given the lack of a true placebo (i.e., no manufacturer-developed placebo Ozempic® pen/cartridge), this study will use a placebo condition that consists of weekly sham doses. Sham doses will involve a brief needle prick using a very small (e.g., 32 gauge) needle. These procedures are described in more detail later in this protocol. By necessity, medical staff administering the medication cannot be blind to condition. However, other experimenters and outcomes assessors will remain blind to condition. Participants will be instructed during the informed consent and study orientation that they have a 50/50 chance of being randomized to medication and placebo groups.

4.4 END OF STUDY DEFINITION

A participant is considered to have completed the study if he or she has completed all phases of the study including the last visit shown in the Schedule of Activities (SoA), Section 1.3. The end of the study is defined as completion of the last visit shown in the SoA in the trial overall.

5 STUDY POPULATION

5.1 INCLUSION CRITERIA

In order to be eligible to participate in this study, an individual must meet the following criteria:

- Age 21-65
- Meeting DSM-5 criteria for current (past year) mild or moderate AUD (i.e., from 2-5 symptoms endorsed), and NIAAA criteria for current at-risk drinking (i.e., >7/14 drinks in one week for women/men, with at least two episodes of 4+/5+ drinks in the past 30 days)
- Daily smoker, defined as reporting smoking 1+ cigarettes per day, on average, over the past 12 months (past year avg cigarettes per day ≥ 1) and daily/near-daily smoking in the past month (smoking at least 25 days in the past 30)
- Willingness/availability to take study medication and complete study procedures, including attending weekly visits for medication administration, side effect assessments, and glucose monitoring
- Willingness to complete laboratory sessions involving alcohol administration
- Ability to communicate and read in English

5.2 EXCLUSION CRITERIA

- Regular use of electronic nicotine delivery systems (ENDs; vaping), cigars, chewing tobacco or snuff, based on at least weekly use in the past 30 days
- Past 30-day use of nicotine replacement therapies/products
- Reporting past 30-day use of illicit drugs other than cannabis at baseline, or having a positive toxicology screen for illicit drugs other than cannabis at baseline
- Meeting past-year criteria for a substance use disorder (with the exception of alcohol, tobacco or mild cannabis use disorder)
- Current engagement in alcohol or smoking cessation treatments, or currently engaged in intentional efforts to quit alcohol use
- Past 30-day use of: Sincalide, Sulfonylureas, insulin and insulin products or other medications that may interact with semaglutide;, or weight control medications
- Prior use of semaglutide or other GLP-1 agonists
- Known or suspected hypersensitivity to study medication or related products
- Lifetime diagnosis of severe mental illness (including schizophrenia and bipolar disorder)
- History of suicide attempt, or recent (past 30 day) suicidal ideation, or psychiatric hospitalization in the last 6 months

- Current significant medical or neurological illness (based on self-report or medical record) including severe hepatic impairment or cirrhosis, impaired renal function (eGFR <50ml/min), acute or chronic pancreatitis, gastroparesis, gallbladder disease or cholelithiasis, other severe gastrointestinal disease, heart failure, coronary artery disease, stroke, seizure disorder, or other medical condition that poses a risk for the medication or alcohol administration components of the study (as determined by the MD)
- A personal or family history of medullary thyroid cancer or multiple endocrine neoplasia 2A or 2B
- Calcitonin greater than or equal to 50 ng/L
- Uncontrolled thyroid disease TSH >6.0 mIU/L or <0.4 mIU/L at screening
- History of major surgical procedures involving the stomach potentially affecting absorption of trial product (e.g., subtotal and total gastrectomy, sleeve gastrectomy, gastric bypass surgery)
- History of Type 1 or Type 2 diabetes, or HbA1c >6.5% measured at screening
- History of diabetic retinopathy, proliferative retinopathy, or maculopathy
- History of diabetic ketoacidosis
- History or presence of malignant neoplasms within the last 5 years (except basal and squamous cell skin cancer and carcinoma in situ)
- Currently nursing, pregnant, anticipating pregnancy in the next 6 months, or not using a highly effective contraceptive method as judged by the MD, and defined as:
    a. combined (estrogen and progestogen containing) hormonal contraception associated with inhibition of ovulation (oral, intravaginal, transdermal)
    b. progestogen-only hormonal contraception associated with inhibition of ovulation (oral, injectable, implantable)
    c. intrauterine device
    d. intrauterine hormone-releasing system
    e. bilateral tubal occlusion
    f. vasectomized partner
    g. sexual abstinence
- Elevation of serum lipase, amylase, direct (conjugated) bilirubin, or alkaline phosphatase (ALP), ALT, or AST) more than 3X the upper limit of normal on baseline bloodwork
- Baseline body mass index (BMI) <25kg/m2 or >35kg/m2
- Uncontrolled hypertension or systolic BP >180 mmHg and/or diastolic BP >105 mmHg, averaged from three measurements
- Plans for travel outside of the local area in the upcoming 12 weeks that would interfere with lab visits during the study period (or other logistic factors that would make it difficult to commit to entire duration of study)

5.3 LIFESTYLE CONSIDERATIONS

Specific lifestyle instructions will include:

- Refrain from alcohol consumption or other drug use for 24 hours prior to alcohol administration sessions
- Arrive fasting at sessions scheduled for blood draws as needed
- Refrain from consuming food for 4 hours prior to alcohol sessions. Participants will receive a standardized snack on arrival for alcohol sessions.)
- Refrain from driving to the lab on the day of alcohol sessions. (Participants will be asked to arrange for transportation, or will be provided with transportation home via taxi or Uber)
- Refrain from operating other vehicles or machinery for the duration of day upon discharge from alcohol sessions
- Women participating in the study must report being willing to use a reliable birth control method during the trial (if sexually active), and must report no plans for pregnancy in the next 6 months.
- Participants will also understand that they may be excluded if they test positive for drugs other than cannabis during the time of scheduled drug screens (repeat drug tests will take place during weeks 4 and 8).

5.4 SCREEN FAILURES

Screen failures are defined as participants who consent to participate in the clinical trial but are not subsequently randomly assigned to the study intervention or entered in the study. A minimal set of screen failure information is required to ensure transparent reporting of screen failure participants, to meet the Consolidated Standards of Reporting Trials (CONSORT) publishing requirements and to respond to queries from regulatory authorities. Minimal information includes demography, screen failure details, eligibility criteria, and any serious adverse event (SAE).

Examples of screen failures would include participants excluded following baseline laboratory work, or those excluded based on a change in treatment-seeking status at the time of the baseline assessment. Individuals who do not meet the criteria for participation in this trial (screen failure) because of a specific modifiable factor (e.g., upcoming travel) may be rescreened at a later date. Rescreened participants will be assigned the same participant number as for the initial screening. Data pertaining to screening failures will be tabulated in an anonymized fashion.

5.5 STRATEGIES FOR RECRUITMENT AND RETENTION

Participants will be recruited from the community through advertisements, which may include ads in local online or print newspapers, print flyers placed in community or hospital settings, business cards, campus listservs, and online postings (e.g. Craigslist (ads will not be placed in employment section)), and social media ads (Facebook, Twitter, Instagram) that direct potential participants toward a study website/landing page (hosted on Research for Me). Participants may also be recruited from local medical clinics (e.g., primary care clinics) that grant approval for assisting with recruitment (e.g., by placing study advertisements in clinics). Flyers and online advertisements will include study contact information and a web link and/or QR code leading to the landing page.

In addition to these “passive” methods of recruitment, we will also include “active” recruitment methods. These methods will include members of the study team setting up recruitment booths and

passing out business cards at public events (e.g., festivals) or public venues. Once initial contact with participants is made (e.g., via email or in person), research staff will schedule the participant for a phone call to explain the study and conduct a screening interview.

Participants will receive compensation. The payments will be allocated as follows (please see 1.2 for a summary of all scheduled sessions):

- $50 for completing the consent/screening/baseline sequence (Week 0)
- $75 for alcohol challenge session 1 (To be scheduled between Weeks 0-1)
- $75 for alcohol self-administration session 1 (To be scheduled between Weeks 0-1)
- $100 for alcohol challenge session 2 (To be scheduled between Weeks 9-10)
- $100 for alcohol self-administration session 2 (To be scheduled between Weeks 9-10)
- $30 per visit for each follow-up/medication administration visit during Weeks 1-10 (10 visits x$30 per visit = $300)
- Up to $150 for completing and returning daily questionnaires over the study period ($15 per weekly set of surveys x 10 weekly visits), which includes the week following the 1.0mg dose, to be paid at weekly medication visits)
- A $50 bonus for completing at least 9 of 10 weekly assessments
- Participants will receive a final bonus of $100 for completing the study through Week 10 (to be paid at the final discharge visit).

Therefore, the total maximum compensation over 10 weeks for study visits is $1000. Compensation for each pair of the alcohol sessions will occur at the end of the second of the session pair. The final payment (including bonuses) will be paid at the Week 10 discharge visit. Overall, these payments are structured to maximize retention in the study (given the long dose escalation period and time-intensive laboratory sessions). Additionally, an incentive-based structure that includes weekly incentives is important to reinforce engagement with daily surveys, and to minimize missing data. Participants will be updated at each visit with their progress toward the bonus incentives for adherence (e.g., weekly bonus of $10 and progress toward final adherence bonuses). In the event participants are not able to pick their final payments in person, we will offer the option of issuing the final payment via electronic gift card to the participant's email address, with their permission.

We anticipate phone screening up to 400 individuals and randomizing up to 48 participants to medication or placebo conditions, in order to obtain a final sample of at least 36 eligible participants who complete the full course of the 0.5mg phase (allowing for 25% attrition). If attrition is higher than anticipated, we will submit an amendment to enroll additional participants to arrive at a final sample of at least 36 participants who complete study procedures through the end of the 0.5mg dosage phase (i.e., through the end of Week 9).

6 STUDY INTERVENTION

6.1 STUDY INTERVENTION(S) ADMINISTRATION

6.1.1 STUDY INTERVENTION DESCRIPTION

Semaglutide is a relatively new (approved in 2017) GLP-1 receptor agonist. A new oral formulation of

semaglutide was approved by the FDA in 2019, making semaglutide the first drug in its class to be available in both subcutaneous and oral formulations. GLP-1 agonist medications were designed to mimic the function of the endogenous GLP-1 peptide, with the benefit of a much longer half-life than endogenous GLP-1. Based on their glucose-lowering and appetite-reducing properties, GLP-1 agonists were developed as anti-diabetic therapies. Several GLP-1 agonists (e.g., exenatide, liraglutide, lixisenatide, albiglutide, dulaglutide and semaglutide,) are now in widespread use as treatments for type II diabetes (T2D); these medications have also proven efficacious for weight loss, reducing glycated hemoglobin (HbA1c), and reducing risk for some cardiovascular events [29]. Although the primary indication of GLP-1 agonists is T2D, these therapies are increasingly being studied for other indications in non-diabetic patients, including obesity/weight management. In June 2021, the FDA approved semaglutide at a higher dosage (2.4mg/week) for the indication of weight management, under the brand name Wegovy.

6.1.2 DOSING AND ADMINISTRATION

Semaglutide will be administered according to the FDA-approved schedule, which begins with a starting dose of 0.25mg once per week for four weeks. At week five, dosage is increased to 0.5mg for a subsequent four weeks (initial maintenance dose), after which point dosage can be increased further (to 1.0mg), presuming the medication is well tolerated.

Although semaglutide can be self-administered in the home setting, medication and placebo administration will be delivered by a research nurse or other medical team member at Eastowne CRU or CTRC in order to execute the placebo manipulation and ensure adherence to medication. This approach also allows us to collect safety outcomes on a regular basis, further ensuring that participants will be monitored carefully. Semaglutide is administered subcutaneously (in the abdomen, thigh, or upper arm). Doses are delivered via the Ozempic® pen, a cartridge-based device that calibrates medication delivery based on the desired dose. An extremely small needle (4mm, 32-gauge needle – the size of 2 human hairs) is used to deposit the medication subcutaneously. Because each pen contains 2mg of medication, each participant in the medication group will be allocated one pen to cover the first six weeks (i.e., 0.25mg/week for 4 weeks, followed by 0.5mg/week for 2 weeks), and a second pen for that will supply medication for the remaining three weeks (2 weeks at 0.5mg, and 1 week at 1.0mg). This schedule results in 9 medication administration sessions. At the week 9 visit, the 1.0mg dose will be delivered as 2 x 0.5 mg doses given in succession. This is because the medication pens being used for this study deliver dosages of 0.25 and 0.5mg, and participants are not being maintained on the 1.0mg dose after week 9. This timeframe also allows sufficient time to reach steady-state plasma concentration levels (achieved within 4-5 weeks of initiating the starting dose).

At the present time, we do not have access to a manufacturer-developed placebo (the medication relies on the proprietary Ozempic® pen for delivery). Therefore, this study will use weekly sham injections for the placebo group. Sham doses will involve a brief needle prick using a very small (e.g., 32 gauge) needle, identical to the one used in the Ozempic® pen (NovoFine), or a needle of similar gauge (e.g., an insulin needle). Given the extremely small volume of medication delivered in the treatment condition, we will not utilize saline or another inert substance for the placebo administration. During medication and placebo administration, participants' view will be shielded from the injection site to prevent unblinding. Medication will be delivered in the upper arm, abdomen, or thigh. Given the extremely small volume of the injections, we will not utilize saline or another inert substance for the placebo administration; rather, the sham doses will consist of briefly inserting the micro-needle.

Due to the nature of the placebo condition, it will not be possible to blind the staff RNs administering the medication. However, we will provide training to study staff to minimize the likelihood of contaminating the blinding procedures. We will also develop procedures to minimize contact between the RNs and outcome assessors. All other staff members (including research assistants and outcomes assessors) will remain blind to condition, unless unblinding is necessary (e.g., due to an adverse event).

6.2 PREPARATION/HANDLING/STORAGE/ACCOUNTABILITY

6.2.1 ACQUISITION AND ACCOUNTABILITY

The team will collaborate with IDS to source the medication (and NovoFine or insulin needles for the placebo condition), which will be sourced and stored at the IDS pharmacy prior to dispensing. Participants assigned to medication will be prescribed one Ozempic® pen following randomization. As per IDS requirements, in between study visits participants will be given their assigned medication pen and asked to bring their pen back to each follow-up visit. In between visits, pens will be placed in a small plastic container and then encased opaque, tamper-proof bag in order to prevent unblinding. (For participants in the placebo condition, a plastic object of roughly equal size and weight will be placed in the plastic container and medication bag.) Participants will be instructed not to open the bag in between visits. A refill pen will be prescribed prior to the Week 7 visit, once the doses from the first pen have been allocated. Any unused doses from individual pens (e.g., in the event of participant drop-out) will be discarded/destroyed by IDS, as will any pens that expire during the course of the trial. In the event a pen expires before a participant completes the study, a replacement pen will be prescribed at the discretion of the study physicians.

6.2.2 FORMULATION, APPEARANCE, PACKAGING, AND LABELING

Ozempic® is manufactured by Novo Nordisk. As described above, the medication is dispensed from a proprietary device, the Ozempic® pen. Each pen contains 2mg of medication, requiring that each participant is prescribed two pens over the course of the study. A full description and visuals can be found at the following site:
https://www.Ozempic®pro.com/how-to-prescribe/Ozempic®-pen.html

The *placebo group* will receive sham injections that involve a brief needle-prick without dispensing a substance. The needles used for the sham injections will be the NovoFine 32G needle, the same needle in the Ozempic® pen (https://www.novoneedles.com/novofine.html) or a comparable needle of a similar gauge (e.g., an insulin needle). In no case will any fluid be injected during placebo sessions.
To mimic the active medication administration, the medical staff member will insert the microneedle briefly and then remove it while the participant's view is shielded.

6.2.3 PRODUCT STORAGE AND STABILITY

Pens will be dispensed from IDS. For drug dispensing visits at CRU (Eastowne), scheduled for Weeks 1 and 7, pens will be delivered the day of the visit by courier. Ozempic pens will be kept in the participant's possession (sealed in a tamper-proof bag) between visits. According to the manufacturer, following the first use, the Ozempic® pen can be stored for up to 56 days at room temperature (59 to 86 F) or refrigerated at 36 to 46 F. As per our plan developed in collaboration with IDS, participants will be instructed to keep the medication bag at room temperature for simplicity. Each participant's pen will be

labeled with the study ID number and pens will not be shared across participants. Placebo needles will remain packaged until use, and will be stored at room temperature in a secure location.

6.2.4 PREPARATION

Semaglutide does not require advance preparation; cartridges are pre-filled and doses are calibrated using the pen device. The RN or medical staff member will select the correct dosage and administer the medication as per manufacturer guidelines.

6.3 MEASURES TO MINIMIZE BIAS: RANDOMIZATION AND BLINDING

Following the baseline assessment, participants will be randomized to medication or placebo conditions at a 1:1 ratio by a member of the IDS team using a pre-determined randomization scheme. Medication and placebo administration will be carried out by a staff RN at CRU or CTRC. Due to the nature of the placebo condition, it will not be possible to blind the RNs to condition. However, all other staff members will remain blind to condition, and the staff members administering medication will be trained in protecting the blind (e.g., redirecting the conversation if the participant comments about medication condition; minimizing contact with experimenters.) To maintain blinding of participants, participants' view will be shielded and they will be asked to face away from the injection site during all placebo and medication administrations. Aside from the staff members administering medication, no study team members interacting with participants will be aware of participants' assignments. At the stage of interim analyses (e.g., in preparing safety reports for DSMB meetings) and final analyses, the study statistician will be provided with the assignment log. None of the study personnel involved in recruitment or enrollment will have knowledge of the details of the randomization table that will be used by the IDS pharmacy.

At the completion of the trial, participants will be provided with a questionnaire asking about perceived medication assignment (to assess the success of maintaining the blind). Due to common side effects of semaglutide (in particular nausea), we do not expect blinding to be successful in all cases.

6.4 STUDY INTERVENTION COMPLIANCE

Medication will be administered by CRU or CTRC staff to assure compliance and reduce potential sources of non-adherence. A missed session will constitute a missed medication dose. As per manufacturer directions, any missed doses/appointments will be corrected by asking the participant to come in for the dose as soon as possible within 5 days of the missed dose. After the 5-day mark has passed, the dose will be skipped, and the participant will resume with the next scheduled dose. Study medication logs will serve as the primary source document for tracking medication and placebo administration. Participants will be coded as non-adherent if they miss more than 2 scheduled doses between Weeks 1-9. In the event of missed medication doses or missed appointments, the physician will have the discretion to adjust the appointment date (within the manufacturer or standard practice guidelines), or to prescribe a replacement pen if needed to accommodate the target medication schedule.

6.5 CONCOMITANT THERAPY

Participants may be taking medications for other conditions (provided those treatments are not included in the list of exclusion criteria). For this protocol, a prescription medication is defined as a medication that can be prescribed only by a properly authorized/licensed clinician. Medications to be reported in the Case Report Form (CRF) are concomitant prescription medications, over-the-counter medications and supplements. Changes to medication status or other treatments will be assessed at each in-person visit, with any changes reviewed by a study physician. Should a concomitant therapy preclude further participation for safety or scientific reasons (for instance, if a participant starts a therapy that constitutes an exclusion factor), this issue will be discussed by the study leads and the participant will be excused from further participation, if required to ensure safety. By definition, participants will not be seeking treatment for alcohol use or smoking cessation. If a participant makes the decision to seek treatment for alcohol or smoking during the study, s/he will be referred to appropriate treatment resources and excused from further participation.

6.5.1 RESCUE MEDICINE

Not applicable

7 STUDY INTERVENTION DISCONTINUATION AND PARTICIPANT DISCONTINUATION/WITHDRAWAL

7.1 DISCONTINUATION OF STUDY INTERVENTION

Study discontinuation: The study may be halted if deemed necessary by the lead investigators, DSMB, or IRB. Examples of reasons for study discontinuation include unexpectedly high frequency of serious adverse events, or significant increases in alcohol use during treatment, leading to the conclusion that the medication is not safe in this population. In addition to the participant discontinuation rules noted below, the following study stopping rules will trigger halting the study pending a DSMB evaluation: 1) Occurrence of any hospitalization or any SAE rated at Grade 4-5 (CTCAE) in a medication-treated participant (and deemed likely to be medication-attributable); 2) Documentation of SAEs in >3 medication-treated participants that are deemed likely attributable to medication; 3) Removal of >5 subjects due to adverse events deemed attributable to study-related procedures, inclusive of SAE and/or medical stopping rules (e.g., weight, alcohol consumption, disease progression), as defined in 7.2 below. In order to classify events as medication-attributable, the PI will request that IDS breaks the blind and provides a file containing condition assignment to the study physician(s). If any of the aforementioned events trigger a DSMB meeting, the full randomization table will be provided to a statistician, so that group differences can be fully evaluated at the meeting. Given the safety profile of semaglutide for other clinical conditions, as well as preclinical evidence that GLP-1 receptor agonists reduce drug intake, these scenarios are expected to be unlikely.

7.2 PARTICIPANT DISCONTINUATION/WITHDRAWAL FROM THE STUDY

Participants are free to withdraw from participation in the study at any time upon request. The investigators may discontinue or withdraw a participant from the study if clinically or otherwise indicated. Examples of reasons removing a participant include:

- Significant study intervention non-compliance
- Any clinical adverse event (AE), laboratory abnormality, or other medical condition or situation that indicates that continued participation in the study would be unsafe
- Pregnancy
- Diagnosis of significant disease or disease progression which requires discontinuation of the study intervention
- The participant meets an exclusion criterion (either newly developed or not previously recognized) that precludes further study participation
- Participant relocates from the local area, or is otherwise unable to attend visits
- Significant or persistent side effects that pose significant discomfort or preclude escalation to the 0.5mg dose (e.g., persistent significant nausea, or hypoglycemia, as indicated by repeated fasting glucose values below normal 54 mg/dL
- Changes in lab tests or clinical status from baseline that render the participant ineligible to continue in the judgement of the physician(s) (e.g., elevated liver enzymes, elevated serum amylase or lipase levels, weight loss as defined below)
- Excessive weight loss (defined as loss exceeding 5% of baseline weight or BMI < 20)
- A change in treatment-seeking status (i.e., the participant expresses a desire to enter treatment for alcohol or smoking cessation).
- A significant increase in alcohol consumption, as evidenced by a >50% increase in drinks per week (relative to baseline) lasting more than two weeks. Drinks per week in the 30 days preceding the Week 0 visit (measured by the TLFB) will serve as the baseline measure.
- Evidence of significant alcohol withdrawal symptoms
- Any other change in medical status or side effects that is significant enough to warrant removal in the judgement of the study physicians

If a participant exhibits changes in symptoms or clinical status that warrant potential removal, the study physicians will confer and render a decision as to whether to withdraw the participant. With respect to dosage increase, if a participant's side effects profile or laboratory tests during the initial 4-week phase (.25mg) indicate that s/he is unable to safely proceed to the 0.5mg dose, the participant may be removed from the study at the discretion of the physicians. If lab tests or side effects during the standard maintenance dose of 0.5mg suggest that a participant is unlikely to tolerate a higher dose (1mg), they may be excluded from the 1.0mg dose escalation at the discretion of the physicians. In this case, participants will be given the option of one additional week at the standard therapeutic dose of 0.5mg (i.e., highest tolerated dose). Reasons for participant discontinuation or withdrawal from the study will be recorded on the Case Report Form (CRF). Participants who sign the informed consent form but do not receive the study intervention (e.g., fail to attend the Week 1 visit for randomization) may be replaced. Participants who sign the informed consent form, and are randomized and receive the study intervention, and subsequently withdraw, or are withdrawn or discontinued from the study, may be replaced.

7.3 LOST TO FOLLOW-UP

A participant will be considered lost to follow-up if he or she fails to return for 2 consecutive scheduled medication visits and is unable to confirm attendance at the following visit (or is unable to be contacted by the study staff

The following actions will be taken if a participant fails to return to a required study visit:

- The team will attempt to contact the participant and reschedule the missed visit within one week, and counsel the participant on the importance of maintaining the assigned visit schedule and ascertain if the participant wishes to and/or should continue in the study.
- The team will attempt to contact the individuals that the participant had listed on the Locator Form at baseline (maximum of three attempted contacts per individual).
- Before a participant is deemed lost to follow-up, the investigator or designee will make every effort to regain contact with the participant (where possible, up to 3 telephone messages, up to 3 email messages, and if necessary, a letter to the participant's last known mailing address or local equivalent methods). These contact attempts will be documented in the participant's medical record or study file.
- Should the participant continue to be unreachable, he or she will be considered to have withdrawn from the study with a primary reason of lost to follow-up.

8 STUDY ASSESSMENTS AND PROCEDURES

8.1 EFFICACY ASSESSMENTS

The efficacy assessments/outcomes are noted in summary form in section 3. The ascertainment of these outcomes is described in further detail in section 4 (study design). Briefly, the endpoints are human laboratory measures of alcohol consumption and alcohol response; self-reported alcohol consumption and cigarette use over the course of the trial; and changes in weight, HbA1c, and alcohol elimination.

8.2 SAFETY AND OTHER ASSESSMENTS

Safety and assessments are summarized in detail in the study design section (Section 4.1). Briefly, safety assessments include a baseline assessment, body weight, bloodwork, vitals signs, and clinical interview to assess substance use and psychiatric symptoms. Follow-up medical visits will include repeat assessments of bloodwork and scheduled time points, as well as follow-up measurements of body weight and vitals signs. Medication side effects will be assessed at each visit, and a study team member will contact participants within 2-3 days of each dosage increase to evaluate potential side effects. Other assessments (questionnaires, interviews) are noted in Section 4.1.

Safety precautions during alcohol administration sessions. Our team has considerable experience with alcohol administration procedures. Our procedures follow the NIAAA guidelines for administering alcohol in human studies (https://www.niaaa.nih.gov/research/guidelines-and-resources/administering-alcohol-human-studies). Based on these guidelines, alcohol administration studies with participants who meet criteria for AUD are typically restricted to non-treatment-seeking participants in order to avoid ethical concerns, as will be the approach in this study. Additionally, our approach in this study is to only recruit people who meet criteria for mild or moderate AUD (i.e., endorsing 2, 3, 4 or 5 symptoms out of 11). Regarding the alcohol dose for alcohol challenge sessions, we will use sex- and body weight adjusted formulas [70] to ensure that dosage is calibrated across participants to achieve the target BrAC

(.06g%). Participants will have undergone a medical screen prior to participation, and will be excluded if the study medical staff has reason to believe that alcohol is contraindicated. Participants will receive a BrAC reading on arrival to the alcohol sessions; the session will be rescheduled if the participant presents with a positive BrAC.

Following the completion of alcohol sessions, participants will be escorted to a private room designated for recovery purposes. During alcohol self-administration sessions, participants will only be discharged after their breath alcohol concentration descends below .03g%, which is consistent with NIAAA guidelines. During alcohol challenge sessions, participants will be asked to remain in the lab until BrAC reaches 0mg% (in order to collect BrAC data until alcohol is fully eliminated, for purposes of pharmacokinetic analyses). Participants will also be provided with reimbursement for transportation if they do not drive and are not able to locate assistance with transportation (participants will be advised not to drive to the alcohol sessions). If a participant presents with a positive BrAC, the session will be rescheduled, and the participant will be asked to remain in the lab until it is safe to leave.

To ensure participant safety, at least two members of the study staff will be present for the duration of all alcohol administration sessions. If an urgent medical situation arises at either medication or alcohol visits, a team member will contact the physicians on the team to consult, based on a predetermined sequence (e.g., contact physician A, followed by physician B, then physician C). Additionally, any participants who express a desire to seek treatment during the study will be excused from further participation and provided with alcohol or smoking cessation resources. Co-I Dr. Jordan directs outpatient addiction treatment at UNC-CH.

8.3 ADVERSE EVENTS AND SERIOUS ADVERSE EVENTS

8.3.1 DEFINITION OF ADVERSE EVENTS (AE)

Adverse event means any untoward medical occurrence associated with the use of an intervention in humans, whether or not considered intervention-related (21 CFR 312.32 (a)).

8.3.2 DEFINITION OF SERIOUS ADVERSE EVENTS (SAE)

An adverse event (AE) or suspected adverse reaction is considered “serious” if, in the view of either the investigator or sponsor, it results in any of the following outcomes: death, a life-threatening adverse event, inpatient hospitalization or prolongation of existing hospitalization, a persistent or significant incapacity or substantial disruption of the ability to conduct normal life functions, or a congenital anomaly/birth defect. Important medical events that may not result in death, be life-threatening, or require hospitalization may be considered serious when, based upon appropriate medical judgment, they may jeopardize the participant and may require medical or surgical intervention to prevent one of the outcomes listed in this definition. Examples of such medical events include an adverse reaction requiring intensive treatment in an emergency room or at home.

8.3.3 CLASSIFICATION OF AN ADVERSE EVENT

8.3.3.1 SEVERITY OF EVENT

For adverse events (Aes) not included in the protocol defined grading system, the following guidelines will be used to describe severity.

- **Mild** – Events require minimal or no treatment and do not interfere with the participant's daily activities.
- **Moderate** – Events result in a low level of inconvenience or concern with the therapeutic measures. Moderate events may cause some interference with functioning.
- **Severe** – Events interrupt a participant's usual daily activity and may require systemic drug therapy or other treatment. Severe events are usually potentially life-threatening or incapacitating. Of note, the term "severe" does not necessarily equate to "serious".

8.3.3.2 RELATIONSHIP TO STUDY INTERVENTION

All adverse events (Aes) must have their relationship to study intervention assessed by the clinician who examines and evaluates the participant based on temporal relationship and his/her clinical judgment. The degree of certainty about causality will be graded using the categories below. In a clinical trial, the study product must always be suspect.

- **Related** – The AE is known to occur with the study intervention, there is a reasonable possibility that the study intervention caused the AE, or there is a temporal relationship between the study intervention and event. Reasonable possibility means that there is evidence to suggest a causal relationship between the study intervention and the AE.
- **Not Related** – There is not a reasonable possibility that the administration of the study intervention caused the event, there is no temporal relationship between the study intervention and event onset, or an alternate etiology has been established.

8.3.3.3 EXPECTEDNESS

The study physicians will be responsible for determining whether an adverse event (AE) is expected or unexpected. An AE will be considered unexpected if the nature, severity, or frequency of the event is not consistent with the risk information previously described for the study intervention.

8.3.4 TIME PERIOD AND FREQUENCY FOR EVENT ASSESSMENT AND FOLLOW-UP

The occurrence of an adverse event (AE) or serious adverse event (SAE) may come to the attention of study personnel during study visits and interviews of a study participant presenting for medical care, or upon review by a study monitor. At weekly medication visits, all participants will review a side effects checklist (SAFTEE) with a research assistant, which will then be provided for review by a medical staff member at CTRC. The staff member will begin with an open-ended question about side effects or any other adverse events, followed by a review of specific side effects using the SAFTEE. Any previously reported side effects will be queried to determine any change in status (presence or severity). These weekly visits will be a primary method for identifying side effects. Additionally, a research staff member will contact participants by phone in the week following the initial dose, as well as the week following each dose escalation. Phone calls will be scheduled 2-3 days following these events.

All Aes including local and systemic reactions not meeting the criteria for SAEs will be captured on the appropriate case report form (CRF). Information to be collected includes event description, time of onset, clinician's assessment of severity, relationship to study product (assessed only by those with the training and authority to make a diagnosis), and time of resolution/stabilization of the event. All Aes

occurring while on study will be documented appropriately regardless of relationship. All Aes will be followed to adequate resolution.

Any medical condition that is present at the time that the participant is screened will be considered as baseline and not reported as an AE. However, if the study participant's condition deteriorates at any time during the study, it will be recorded as an AE.

Changes in the severity of an AE will be documented to allow an assessment of the duration of the event at each level of severity to be performed. Aes characterized as intermittent require documentation of onset and duration of each episode.

Under the direction of the PI, members of the research team will record all reportable events with start dates occurring any time after informed consent is obtained until 7 (for non-serious Aes) or 30 days (for SAEs) after the last day of study participation. At each study visit, the team member will inquire about the occurrence of AE/SAEs since the last visit. Any such events will be followed for outcome information until resolution or stabilization.

8.3.5 ADVERSE EVENT REPORTING

All project staff will be trained to identify adverse events (Aes), which must be reported to the PI and study physicians within 24 hours. In addition, any unanticipated participant issues will be discussed in weekly staff meetings, which will provide another opportunity to identify Aes.

8.3.6 SERIOUS ADVERSE EVENT REPORTING

Research staff will be trained to report potential Aes or SAEs to the PI immediately. For any potential Aes/SAEs, the study physician will make a determination to distinguish between Aes, SAEs and unanticipated problems and provide judgment about attributions. Management and reporting of Aes, SAEs, and unanticipated problems will be carried out in accordance with Good Clinical Practice (GCP) standards, as outlined by the Food and Drug Administration. Aes will be defined as any negative medical event during the trial (whether or not the event is deemed related to the study medication). SAEs are defied as events that are potentially life threatening or result in heath; cause congenital malformation; result in persistent/significant disability or incapacity; require inpatient hospitalization or prolongation of hospitalization; or reflect an otherwise notable medical event in the judgment of the study physician. Aes/SAEs will be monitored regularly by the study research team and medical staff, in particular by inquiring about adverse events at each weekly visit. Should Aes or SAEs occur, they will be recorded, rated as mild/moderate/severe by the study physician, and reported in accordance with UNC IRB requirements and GCP standards. Any Aes will be reported to the IRB in progress reports and at the conclusion of the study. SAEs and unanticipated events will be reported to the IRB and DSMB within 48 hours, and to NIAAA if judged as treatment-related. All staff working on this study will be made aware of the monitoring and reporting requirements for Aes/SAEs, and will have completed GCP training and other trainings required to conduct human subjects research at UNC.

Any Aes, SAEs or unanticipated problems will be followed until resolution by the Pis and/or research staff. Participants will be contacted regularly during follow-up and any observations of Aes/SAEs will be

discussed among the Pis and the study physician at regular meetings (or immediately, in the event of SAEs or unanticipated problems). In the event of SAEs or unanticipated problems, the physician and study Pis will be notified immediately, and the participant will be referred to or provided with appropriate medical support.

Participants will be advised to seek prompt medical attention in the event of an unanticipated medical event. In the event of an SAE, a member of the study medical staff will maintain regular contact with the participant until the event is resolved and fully documented. A determination of relatedness to medication will be made based on symptoms, timing, and other factors.

Monitoring will be the primary responsibility of the PI. In addition, a Data and Safety Monitoring Board will be established to maximize safety (the TraCS DSMB will be involved for this project, and will review progress at scheduled semiannual meetings during the trial).

8.3.7 REPORTING EVENTS TO PARTICIPANTS

If an event is deemed an AE or SAE, participants will receive a phone call from study personnel to inform them that the event has been recorded and will be reported as such. Any incidental findings uncovered during the baseline medical exam or weekly visits (e.g., via bloodwork or other measurements) will be reported to the participant by the study physician, and an appropriate follow-up plan will be recommended to the participant.

8.3.8 EVENTS OF SPECIAL INTEREST

Not applicable

8.3.9 REPORTING OF PREGNANCY

At the baseline visit and each study visit that involves an alcohol session, female subjects will undergo a urine pregnancy test upon arrival to the clinical site. In the case of a positive pregnancy test, the participant will be notified immediately, and study intervention will be discontinued while continuing safety follow-up. If any follow-up plan is recommended by the study physician, one of the staff members will communicate this recommendation to the participant.

8.4 UNANTICIPATED PROBLEMS

8.4.1 DEFINITION OF UNANTICIPATED PROBLEMS (UP)

The Office for Human Research Protections (OHRP) considers unanticipated problems involving risks to participants or others to include, in general, any incident, experience, or outcome that meets **all** of the following criteria:

- Unexpected in terms of nature, severity, or frequency given (a) the research procedures that are described in the protocol-related documents, such as the Institutional Review Board (IRB)-approved research protocol and informed consent document; and (b) the characteristics of the participant population being studied;

- Related or possibly related to participation in the research ("possibly related" means there is a reasonable possibility that the incident, experience, or outcome may have been caused by the procedures involved in the research); and
- Suggests that the research places participants or others at a greater risk of harm (including physical, psychological, economic, or social harm) than was previously known or recognized.

Any Ups identified by the study team will be discussed amongst the study leadership team, and corrective actions will be identified. If applicable, a protocol amendment will be developed and submitted to the IRB.

8.4.2 UNANTICIPATED PROBLEM REPORTING

The investigator will report unanticipated problems (Ups) to the reviewing Institutional Review Board (IRB), and if applicable, the DSMB. The UP report will include the following information:

- Protocol identifying information: protocol title and number, PI's name, and the IRB project number;
- A detailed description of the event, incident, experience, or outcome;
- An explanation of the basis for determining that the event, incident, experience, or outcome represents an UP;
- A description of any changes to the protocol or other corrective actions that have been taken or are proposed in response to the UP.

8.4.3 REPORTING UNANTICIPATED PROBLEMS TO PARTICIPANTS

In the case of a UP that may impact participants' safety or alter their experience during the trial (e.g., by necessitating a change in protocol), participants will be notified by a study team member at the subsequent visit. In the event of any UP that may have an imminent impact on participant safety, a team member will contact the participant by phone to inform them of the problem.

9 STATISTICAL CONSIDERATIONS

9.1 STATISTICAL HYPOTHESES

- Primary Efficacy Endpoint(s):
    - Breath alcohol concentration (BrAC) and volume of alcohol consumed during laboratory alcohol self-administration procedures. The outcomes are continuous variables. The effect of interest is the interaction between medication group (semaglutide vs. placebo) and time/dosage (baseline vs. 0.5mg). The baseline session will take place between Weeks 0 and 1, and the 0.5mg session will take place between weeks 8 and 9.
- Secondary Efficacy Endpoint(s):
    - Changes in subjective responses to alcohol (stimulation, sedation, craving) and alcohol and cigarette demand during laboratory alcohol challenge procedures. The outcomes will be

analyzed as continuous variables. The effect of interest is the interaction between medication group (semaglutide vs. placebo) and time/dosage (baseline vs. 0.5mg). The baseline session will take place between Weeks 0 and 1, and the 0.5mg session will take place between weeks 8 and 9.

- Changes in drinks per day and cigarette consumption over the course of the trial, as assessed via daily surveys. The outcome variables will be treated as continuous, however, this assumption will be tested and the modeling approach will revised as needed to properly account for the nature of the outcome variable (e.g., analyze as count variable with negative binomial distribution). The effect of interest is the interaction between medication group (semaglutide vs. placebo) and time (Week 1 to Week 10).

9.2 SAMPLE SIZE DETERMINATION

Because no human data on semaglutide's effects on alcohol consumption outcomes are available, there is no a priori effect size estimate to facilitate power calculations. We will seek a final sample size of 36 participants. Assuming at least 36 enrollees have complete data, our analyses suggest that this allows sufficient power (.80) to detect a medium effect size (f = .25) for an interaction between group (medication vs. placebo) and dosage (baseline, 0.25mg, 0.5mg). This estimate assumes an alpha level of .05 and a within-person correlation ® of 0.5 across time points. Further, because our multilevel model (MLM) analyses will involve more time points for certain analyses (e.g., daily assessments), this approach will increase power further, which is advantageous in the event that medication effects are in the small range. Assuming a 25% attrition rate, we anticipate needing to randomize 48 enrollees to arrive at a final sample of 36.

9.3 POPULATIONS FOR ANALYSES

Primary analyses will include all participants who were randomized and received a medication or placebo dose following the Week 0 visit (randomization will occur after the Week 0 visit, just prior to the Week 1 visit). MLM analyses can accommodate missing data, allowing inclusion of participants without complete data (e.g., missed visits or missed daily survey responses). Reasons for missing data and drop-out will be documented in the study database and reported in any publications.

A supplementary per-protocol analyses will be conducted using all participants who received 1) at least 7 of the 9 scheduled doses, and 2) those participants who receive the final (1.0mg) dose. This planned analysis will be conducted irrespective of the number of participants in these groups. If the sample permits, an exploratory subgroup analysis will examine participants as a function of weight (overweight/obese vs. not overweight or obese).

9.4 STATISTICAL ANALYSES

9.4.1 GENERAL APPROACH

Primary analyses will rely on multilevel models (MLM), which are well suited to examining within-person changes over time, while also accommodating missing data and unequal group sizes, and allowing the evaluation of between-subjects moderators (e.g., overweight/obesity status) if necessary. MLM with

maximum likelihood estimation can accommodate missing data under the assumption that the data are missing at random. Secondary, sensitivity analyses will be conducted to test the robustness of study findings for violations of the assumption that data is missing at random. Specifically, we will examine if baseline variables are associated with data missingness (for instance, age, sex, level of substance use) using t-tests and chi-square analyses (for continuous and dichotomous variables, respectively). Baseline variables that are associated with data missingness will be included as covariates in additional adjusted models (that adjust for the baseline variables related to missingness). Assumptions for the statistical methods being used will be tested. Should there be evidence of violations of these assumptions (e.g., presence of outlying and influential cases, distributional concerns for residuals, and so on), appropriate corrective actions will be taken. Sensitivity analyses will be used to determine the extent to which the main study results are influenced by these violations. We will focus our interpretations on small p-values ($p < .05$) that indicate evidence against the null hypothesis. We will report the number of decimal places of the p-value in line with the journal's guidelines and APA recommendations (usually 2 or 3 decimal places). For specific outcomes, the units of measurement and expected ranges of values (or example ranges, if the full range is unknown) are noted in the subsequent analysis sections 9.4.2 and 9.4.3. Any extreme outliers (defined as >3.0 standard deviations from the mean) will be recoded to one unit greater than the next most extreme value, in order to reduce undue influence of extreme outliers. For all statistical tests, confidence intervals will be evaluated in combination with tests of statistical significance. Assumptions for the statistical methods being used will be tested. Should there be evidence of violations of these assumptions (e.g., presence of outlying and influential cases, distributional concerns for residuals, and so on), appropriate corrective actions will be taken. Sensitivity analyses will be used to determine the extent to which the main study results are influenced by these violations.

9.4.2 ANALYSIS OF THE PRIMARY EFFICACY ENDPOINT(S)

Alcohol self-administration sessions: MLM will be used to examine changes in laboratory self-administration (defined as peak BAC and quantity of alcohol consumed, co-primary outcomes) as a function of medication phase (baseline; 0.5mg). These analyses will use medication phase as the within-subjects factor and condition (semaglutide vs. placebo) as the between-person factor. Self-administration will be measured based on recorded breath alcohol concentration (BrAC), measured in g% (e.g., .00g% - .08g%).

9.4.3 ANALYSIS OF THE SECONDARY ENDPOINT(S)

Alcohol challenge sessions: MLM will also be used to examine changes in 1) cigarette and alcohol demand during alcohol challenge (based on corresponding purchase tasks) (e.g., based on number of hypothetical drinks/cigarettes consumed at a given price point), and 2) self-reported stimulation (0-10 range), sedation (0-10 range), and craving (1-7 range), from BAC = .00 to BAC = .06g%, as a function of medication phase (within-person) and drug condition (between person). These analyses will examine changes in outcomes from BAC = .00 to .06 using medication phase as the within-subjects factor and condition as the between-person factor. The analyses will be complemented by analyses of peak subjective effects (e.g., stimulation, sedation, craving, on the same scales as noted above), across the two alcohol administration sessions (AC1 and AC2).

Daily self-report data: We will use MLM to estimate within-person changes in primary outcomes (e.g., daily alcohol use [number of drinks consumed per day] and cigarette use [number of cigarettes smoked per day] over the trial period), using study week as the within-subjects factor and condition (semaglutide, placebo) as the between-person factor.

9.4.4 SAFETY ANALYSES

Total side effects reported (e.g., 0-15), and number of severe side effects reported during each medication phase will be assessed using MLM with medication phase (baseline, 0.25mg, 0.5mg, 1.0mg) as the within-subject factor and condition (semaglutide, placebo) as the between-person factor. . These count variables will be analyzed using a negative binomial MLM, however, this assumption will be tested and the modeling approach will revised as needed to properly account for the nature of the outcome variable (e.g., zero-inflated negative binomial regression).

9.4.5 BASELINE DESCRIPTIVE STATISTICS

Descriptive statistics will be used to report relevant demographics such as age, sex, and education.

9.4.6 PLANNED INTERIM ANALYSES

No interim analyses of efficacy will be planned.

9.4.7 SUB-GROUP ANALYSES

Not applicable

9.4.8 TABULATION OF INDIVIDUAL PARTICIPANT DATA

Not applicable

9.4.9 EXPLORATORY ANALYSES

Sex will be examined as an exploratory moderator in all analyses.

10 SUPPORTING DOCUMENTATION AND OPERATIONAL CONSIDERATIONS

10.1 REGULATORY, ETHICAL, AND STUDY OVERSIGHT CONSIDERATIONS

10.1.1 INFORMED CONSENT PROCESS

In obtaining and documenting informed consent, the team will comply with applicable regulatory requirements (e.g., 45 CFR Part 46, 21 CFR Part 50, 21 CFR Part 56) and adhere to ICH GCP. Consent will be obtained remotely (via web teleconference and Docusign, as described previously) by a qualified study staff member in person at the outset of the baseline session. Additional procedures are noted below.

10.1.1.1 CONSENT/ASSENT AND OTHER INFORMATIONAL DOCUMENTS PROVIDED TO PARTICIPANTS

Consent forms will describe the study intervention, study procedures, and potential risks. The participant's electronic signature indicating consent is required prior to starting intervention/administering study intervention. Participants will also be given an informational sheet about the study medication. Additional procedures are described below.

10.1.1.2 CONSENT PROCEDURES AND DOCUMENTATION

Informed consent is a process that is initiated prior to the individual's agreeing to participate in the study and continues throughout the individual's study participation. Consent forms will be Institutional Review Board (IRB)-approved and the participant will be asked to read and review the document. The initial consent process will be conducted remotely using a HIPAA-compliant web platform (e.g., WebEx, Zoom, Teams) and Part 11-compliant Docusign to maintain optimal safety. A copy of the consent form will be provided to the participant in advance of the scheduled videoconference. A qualified team member will explain the research study to the participant and answer any questions that may arise. A verbal explanation will be provided in terms suited to the participant's comprehension of the purposes, procedures, and potential risks of the study and of their rights as research participants. Participants will have the opportunity to carefully review the consent form and ask questions prior to signing. The participants should have the opportunity to discuss the study with their family or surrogates or think about it prior to agreeing to participate. The participant will electronically sign the informed consent document prior to any procedures being done specifically for the study. Participants must be informed that participation is voluntary and that they may withdraw from the study at any time, without prejudice. A copy of the signed informed consent document will be given to the participants for their records. The informed consent process will be conducted and documented in the source document (including the date), and the form signed (via Docusign), before the participant undergoes any study-specific procedures. The rights and welfare of the participants will be protected by emphasizing to them that the study is voluntary and the quality of their medical care will not be adversely affected if they decline to participate. While the initial consent form will be conducted electronically, supplementary consent forms (e.g., consent to unencrypted communication, HIPAA authorization, and biospecimen consent) may be conducted either remotely (via Docusign) in advance of the baseline visit or in person (paper form) at the baseline visit, depending on timing.

10.1.2 STUDY DISCONTINUATION AND CLOSURE

This study may be temporarily suspended or prematurely terminated if there is sufficient reasonable cause. Written notification, documenting the reason for study suspension or termination, will be provided by the suspending or terminating party to study participants, investigator, funding agency, and regulatory authorities. If the study is prematurely terminated or suspended, the Principal Investigator (PI) will promptly inform study participants, the Institutional Review Board (IRB), and sponsor and will provide the reason(s) for the termination or suspension. Study participants will be contacted, as applicable, and be informed of changes to study visit schedule. Circumstances that may warrant termination or suspension include, but are not limited to determination of unexpected, significant, or unacceptable risk to participants. Study may resume once concerns about safety, protocol compliance, and data quality are addressed, and satisfy the sponsor, IRB and/or Food and Drug Administration (FDA).

10.1.3 CONFIDENTIALITY AND PRIVACY

Participant confidentiality and privacy is strictly held in trust by the participating investigators, their staff, and the sponsor(s) and their interventions. This confidentiality is extended to cover testing of biological samples and genetic tests in addition to the clinical information relating to participants. Therefore, the study protocol, documentation, data, and all other information generated will be held in strict confidence. No information concerning the study or the data will be released to any unauthorized third party. All research activities will be conducted in as private a setting as possible.

Any computerized data will be password protected and contained on a secure, password-protected computer or a secure server. Recruitment data (de-identified) will be stored in a secure, password-protected file accessible only to the study investigators and personnel. At the end of recruitment, eligible subject files will be separated from those failing to meet eligibility criteria, and records of ineligible recruitment subjects will be anonymized. Data pertaining to screening failures will be tabulated in an anonymized fashion. Data collected in other formats (e.g., medical screening notes) will be labeled only by study identifier and stored in binders in secure, locked cabinets while the study is taking place. Files will be stored and archived in accordance with Good Clinical Practice (GCP) standards. For all questionnaire and interview assessments, participants will be informed that they may decline to answer any question if they so choose.

The study monitor, other authorized representatives of the sponsor, representatives of the Institutional Review Board (IRB), regulatory agencies or pharmaceutical company supplying study product may inspect all documents and records required to be maintained by the investigator, including but not limited to, medical records (office, clinic, or hospital) and pharmacy records for the participants in this study. The clinical study site will permit access to such records.

The study participant's contact information will be securely stored during the study for purposes of maintain contact with participants, and for communicating any safety information or unanticipated problems. At the end of the study, all records will continue to be kept in a secure location for as long a period as dictated by the reviewing IRB, Institutional policies, or sponsor requirements.

Study participant research data, which is for purposes of statistical analysis and scientific reporting, will be stored at UNC in the PI's laboratory and on secure backup servers with password-protected access. This will not include the participant's contact or identifying information. Rather, individual participants and their research data will be identified by a unique study identification number. Electronic data are stored on a password protected drive only accessible by the research team. Data obtained on paper will be transferred to an electronic data file (Excel, SPSS, REDCap), stored on a secure server, and identified by participants ID. For non-computer based forms, the data collection sheets are stored in a locked cabinet in a separate locked data storage space at the PI's laboratory.

At the end of the study, all study databases will be de-identified and archived in the PI's laboratory and backed up on a secure server hosted at UNC. De-identified data resulting from this study may also be presented at meetings, published in journals or presentations. In addition, this study will be registered at ClinicalTrials.gov, and de-identified information from this study will be submitted to ClinicalTrials.gov.

10.1.4 FUTURE USE OF STORED SPECIMENS AND DATA

Data collected for this study will be analyzed and stored at the PI's laboratory. The study biostatistician will be given access to the data necessary for conducting specified analyses, and data will be de-identified prior to giving access.

De-identified biological samples will be stored at UNC for the duration of the study, until all biological tests are completed and the data transferred to electronic format. De-identified DNA samples will be stored indefinitely, provided the participant has given consent for DNA storage, in order to examine future questions related to phenotypes of interest for this line of research.

During the conduct of the study, an individual participant can choose to withdraw consent to have biological specimens stored for future research. However, withdrawal of consent with regard to biosample storage may not be possible after the study is completed (after specimens are de-identified).

10.1.5 KEY ROLES AND STUDY GOVERNANCE

Provide the name and contact information of the Principal Investigator and the Medical Monitor.

**Principal Investigator**	**Medical Monitor**
Christian Hendershot, Ph.D., Associate Professor	*TBD (The medical monitor and DSMB members will be identified and added prior to data collection)*
UNC-Chapel Hill	
104 Manning Dr.	
Christian_Hendershot@med.unc."	
Christian_Hendershot@med.unc.edu	

The investigative team is summarized on the face page of this protocol. As noted, a DSMB will be formed prior to carrying out the study.

Safety oversight will be under the direction of a Data and Safety Monitoring Board (DSMB). Members of the DSMB will be independent from the study conduct and free of conflict of interest. The DSMB will dictate the intervals at which they meet to assess safety and efficacy data. At this time, each data element that the DSMB needs to assess will be clearly defined. The DSMB will provide its input to the IRB and National Institutes of Health as necessary.

10.1.6 CLINICAL MONITORING

Clinical site monitoring is conducted to ensure that the rights and well-being of trial participants are protected, that the reported trial data are accurate, complete, and verifiable, and that the conduct of the trial is in compliance with the currently approved protocol/amendment(s), with International Conference on Harmonisation Good Clinical Practice (ICH GCP), and with applicable regulatory requirement(s). A local study monitor will be appointed to provide on-site monitoring during the course of the research study.

10.1.7 QUALITY ASSURANCE AND QUALITY CONTROL

The PI will be responsible for addressing quality control issues and correcting errors or protocol deviations. The PI will have oversight over training study staff and ensuring that protocol elements are carried out with fidelity. Quality control will be ensured by developing SOPs for protocol elements to standardize data collection procedures. The investigational site will provide direct access to all trial related sites, source data/documents, and reports for the purpose of monitoring and auditing by the sponsor, and inspection by local and regulatory authorities.

10.1.8 DATA HANDLING AND RECORD KEEPING

10.1.8.1 DATA COLLECTION AND MANAGEMENT RESPONSIBILITIES

As a single-site, small-scale trial, the project will not involve coordinated data collection and management responsibilities across sites/teams. Data safety and storage precautions are outlined elsewhere in the study protocol.

10.1.8.2 STUDY RECORDS RETENTION

Study records will be retained for a minimum of 7 years following study completion. Paper source documents will be archived in accordance with UNC policies. Electronic materials and de-identified datasets will be stored securely as outlined above.

10.1.9 PROTOCOL DEVIATIONS

A protocol deviation is any noncompliance with the clinical trial protocol, International Conference on Harmonisation Good Clinical Practice (ICH GCP), or Manual of Procedures (MOP) requirements. The PI will report deviations within 7 working days of identification of the protocol deviation, or within 7 working days of the scheduled protocol-required activity. All deviations must be addressed in study source documents, reported to NIAAA Program Official and the IRB. Protocol deviations must be sent to the reviewing Institutional Review Board (IRB) per their policies. The site investigator is responsible for knowing and adhering to the reviewing IRB requirements. Further details about the handling of protocol deviations will be included in the MOP.

10.1.10 PUBLICATION AND DATA SHARING POLICY

This study will be conducted in accordance with the following publication and data sharing policies and regulations:

National Institutes of Health (NIH) Public Access Policy, which ensures that the public has access to the published results of NIH funded research. It requires scientists to submit final peer-reviewed journal manuscripts that arise from NIH funds to the digital archive PubMed Central upon acceptance for publication.

This study will comply with the NIH Data Sharing Policy and Policy on the Dissemination of NIH-Funded Clinical Trial Information and the Clinical Trials Registration and Results Information Submission rule. As such, this trial will be registered at ClinicalTrials.gov, and results information from this trial will be submitted to ClinicalTrials.gov. In addition, every attempt will be made to publish results in peer-reviewed journals. Data from this study may be requested from other researchers after the completion of the primary endpoint by contacting the PI.

10.1.11 CONFLICT OF INTEREST POLICY

Any actual conflict of interest of persons who have a role in the design, conduct, analysis, publication, or any aspect of this trial will be disclosed and managed. Furthermore, persons who have a perceived conflict of interest will be required to have such conflicts managed in a way that is appropriate to their participation in the design and conduct of this trial. All investigators will make a conflict of interest declaration at the outset of the study and at regular intervals thereafter in accordance with UNC policies.

10.2 ADDITIONAL CONSIDERATIONS

Not applicable

10.3 ABBREVIATIONS

AASE	Alcohol Abstinence Self-Efficacy
AE	Adverse Event
APT	Alcohol Purchase Task
AUD	Alcohol Use Disorder
AUDIT	Alcohol Use Disorder Identification Test
AUQ	Alcohol Urge Questionnaire
BAES	Biphasic Alcohol Effects Scale
BIS-11	Barratt Impulsivity Scale
BMI	Body Mass Index
BrAC	Breath Alcohol Concentration
CASE	Cigarette Abstinence Self-Efficacy
CES-D	Center for Epidemiological Studies Depression Scale
CEQ	Control of Eating Questionnaire
CFR	Code of Federal Regulations
CMP	Clinical Monitoring Plan
CO	Carbon Monoxide
CPT	Cigarette Purchase Task
CRF	Case Report Form
CTRC	Clinical & Translational Research Center
DEQ	Drug Effects Questionnaire
DNA	Deoxyribonucleic acid
DSM-5	Diagnostic and Statistical Manual for Mental Disorders v.5

DSMB	Data Safety Monitoring Board
FDA	Food and Drug Administration
FPT	Food Purchase Task
FTND	Fagerstrom test for Nicotine Dependence
GCP	Good Clinical Practice
GLP-1	Glucagon-Like Peptide 1
GLP-1R	Glucagon-Like Peptide 1 Receptor
HbA1c	Glycated hemoglobin
ICH	International Conference on Harmonisation
ICS	Impaired Control Scale
IDS	Inventory of Drinking Situations
IRB	Institutional Review Board
MCQ	Monetary Choice Questionnaire
MNWS	Minnesota Nicotine Withdrawal Scale
MOP	Manual of Procedures
NC Tracs	North Carolina Translational & Clinical Sciences Institute
NDSS	Nicotine Dependence Syndrome Scale
NIAAA	National Institute on Alcohol Abuse and Alcoholism
NIH	National Institutes of Health
NRT	Nicotine Replacement Therapy
PACS	Penn Alcohol Craving Scale
PI	Principal Investigator
POMS-SF	Profile Of Mood States Short Form
PAWS	Prediction of Alcohol Withdrawal Severity Scale
QA	Quality Assurance
QC	Quality Control
RBEDS	Reward Based Eating Drive Scale
RPI-EROS	Reward Probability Index – Environmental Reward Observation Scale
SAE	Serious Adverse Event
SAFTEE	Systematic Assessment for Treatment Emergent Effects
SAWS	Short Alcohol Withdrawal Scale
SNAQ	Simplified Nutritional Appetite Questionnaire
SOA	Schedule of Activities
SOP	Standard Operating Procedure
SRE	Self-Report of the Effects of Alcohol Questionnaire
SUD	Substance Use Disorder
T2D	Type II Diabetes
TAS-20	Toronto Alexithymia Scale
TFEQ	Three Factor Eating Questionnaire
TLFB	Timeline Follow-Back Interview
TQSU	Tiffany Questionnaire of Smoking Urges
UP	Unanticipated Problem
UPPS-P	UPPS-P Impulsive Behavior Scale
WISDM	Wisconsin Inventory of Smoking Dependence Motives
WSWS	Wisconsin Smoking Withdrawal Scale
5-HT2C	Serotonin 2C Receptor

10.4 PROTOCOL AMENDMENT HISTORY

The table below is intended to capture changes of IRB-approved versions of the protocol, including a description of the change and rationale. A Summary of Changes table for the current amendment is located in the Protocol Title Page.

Version	Date	Description of Change	Brief Rationale
1.2	July 26 2021	Elaboration on statistical analyses and clarification on other procedural issues.	Minor changes requested during initial review of the protocol.
1.3	Feb 28 2021	Clarification of medication dispensing procedures, addition of personnel, addition of Docusign procedures, other protocol modifications.	Changes addressed issues that emerged while preparing for recruitment and meeting with IDS and study investigators.
1.4	June 23 2022	Minor revisions to select assessment instruments, exclusion criteria, and clarification of medication dosing and DSMB meeting plan	Refinement of study procedures and clarification of monitoring schedule as requested by DSMB and IDS

11 REFERENCES

1. Koob, G.F., G. Kenneth Lloyd, and B.J. Mason, *Development of pharmacotherapies for drug addiction: a Rosetta stone approach.* Nat Rev Drug Discov, 2009. **8**(6): p. 500-15.
2. Litten, R.Z., et al., *Medications development to treat alcohol dependence: a vision for the next decade.* Addiction biology, 2012. **17**(3): p. 513-27.
3. Litten, R.Z., et al., *Discovery, Development, and Adoption of Medications to Treat Alcohol Use Disorder: Goals for the Phases of Medications Development.* Alcohol Clin Exp Res, 2016. **40**(7): p. 1368-79.
4. Litten, R.Z., et al., *Potential medications for the treatment of alcohol use disorder: An evaluation of clinical efficacy and safety.* Subst Abus, 2016. **37**(2): p. 286-98.
5. Litten, R.Z., et al., *Heterogeneity of alcohol use disorder: understanding mechanisms to advance personalized treatment.* Alcohol Clin Exp Res, 2015. **39**(4): p. 579-84.
6. Heilig, M., et al., *Pharmacogenetic approaches to the treatment of alcohol addiction.* Nature reviews. Neuroscience, 2011. **12**(11): p. 670-84.
7. Falk, D.E., H.Y. Yi, and S. Hiller-Sturmhofel, *An epidemiologic analysis of co-occurring alcohol and tobacco use and disorders: findings from the National Epidemiologic Survey on Alcohol and Related Conditions.* Alcohol Res Health, 2006. **29**(3): p. 162-71.
8. McKee, S.A. and A.H. Weinberger, *How can we use our knowlegde of alcohol-tobacco interactions to reduce alcohol use?* Annu Rev Clin Psychol, 2013. **9**: p. 649–674.
9. Organization, W.H., *Global Health Risks. Mortality and Burden of Disease Attributable to Selected Major Risks*. 2009, World Health Organization: Geneva, Switzerland.
10. Dawson, D.A., *Drinking as a risk factor for sustained smoking.* Drug Alcohol Depend, 2000. **59**(3): p. 235-49.
11. Yardley, M.M., M.M. Mirbaba, and L.A. Ray, *Pharmacological Options for Smoking Cessation in Heavy-Drinking Smokers.* CNS Drugs, 2015. **29**(10): p. 833-45.
12. Prabhu, A., K.O. Obi, and J.H. Rubenstein, *The synergistic effects of alcohol and tobacco consumption on the risk of esophageal squamous cell carcinoma: a meta-analysis.* Am J Gastroenterol, 2014. **109**(6): p. 822-7.
13. Pelucchi, C., et al., *Cancer risk associated with alcohol and tobacco use: focus on upper aero-digestive tract and liver.* Alcohol Res Health, 2006. **29**(3): p. 193-8.
14. Kahler, C.W., N.S. Spillane, and J. Metrik, *Alcohol use and initial smoking lapses among heavy drinkers in smoking cessation treatment.* Nicotine Tob Res, 2010. **12**(7): p. 781-5.
15. Roche, D.J., et al., *Current insights into the mechanisms and development of treatments for heavy drinking cigarette smokers.* Curr Addict Rep, 2016. **3**(1): p. 125-137.
16. McKee, S.A., et al., *Varenicline reduces alcohol self-administration in heavy-drinking smokers.* Biol Psychiatry, 2009. **66**(2): p. 185-90.
17. King, A., et al., *Naltrexone decreases heavy drinking rates in smoking cessation treatment: an exploratory study.* Alcohol Clin Exp Res, 2009. **33**(6): p. 1044-50.
18. O'Malley, S.S., et al., *Dose-dependent reduction of hazardous alcohol use in a placebo-controlled trial of naltrexone for smoking cessation.* Int J Neuropsychopharmacol, 2009. **12**(5): p. 589-97.
19. King, A., et al., *Effects of the opioid receptor antagonist naltrexone on smoking and related behaviors in smokers preparing to quit: a randomized controlled trial.* Addiction, 2013. **108**(10): p. 1836-44.
20. Anton, R.F., et al., *Nicotine-Use/Smoking Is Associated with the Efficacy of Naltrexone in the Treatment of Alcohol Dependence.* Alcohol Clin Exp Res, 2018. **42**(4): p. 751-760.
21. Fucito, L.M., et al., *Cigarette smoking predicts differential benefit from naltrexone for alcohol dependence.* Biol Psychiatry, 2012. **72**(10): p. 832-8.
22. Hartmann-Boyce, J., et al., *Efficacy of interventions to combat tobacco addiction: Cochrane update of 2013 reviews.* Addiction, 2014. **109**(9): p. 1414-25.
23. Kahler, C.W., et al., *A Double-Blind Randomized Placebo-Controlled Trial of Oral Naltrexone for Heavy-Drinking Smokers Seeking Smoking Cessation Treatment.* Alcohol Clin Exp Res, 2017. **41**(6): p. 1201-1211.

24. Mitchell, J.M., et al., *Varenicline decreases alcohol consumption in heavy-drinking smokers.* Psychopharmacology (Berl), 2012. **223**(3): p. 299-306.
25. Fucito, L.M., et al., *A preliminary investigation of varenicline for heavy drinking smokers.* Psychopharmacology (Berl), 2011. **215**(4): p. 655-63.
26. Litten, R.Z., et al., *A double-blind, placebo-controlled trial assessing the efficacy of varenicline tartrate for alcohol dependence.* J Addict Med, 2013. **7**(4): p. 277-86.
27. Roche, D.J., et al., *Combined varenicline and naltrexone treatment reduces smoking topography intensity in heavy-drinking smokers.* Pharmacol Biochem Behav, 2015. **134**: p. 92-8.
28. Ray, L.A., et al., *Varenicline, low dose naltrexone, and their combination for heavy-drinking smokers: human laboratory findings.* Psychopharmacology (Berl), 2014. **231**(19): p. 3843-53.
29. Andersen, A., et al., *Glucagon-like peptide 1 in health and disease.* Nature Reviews Endocrinology, 2018. **14**(7): p. 390-403.
30. Jerlhag, E., *Alcohol-mediated behaviours and the gut-brain axis; with focus on glucagon-like peptide-1.* Brain Res, 2020. **1727**: p. 146562.
31. Brunchmann, A., M. Thomsen, and A. Fink-Jensen, *The effect of glucagon-like peptide-1 (GLP-1) receptor agonists on substance use disorder (SUD)-related behavioural effects of drugs and alcohol: A systematic review.* Physiol Behav, 2019. **206**: p. 232-242.
32. Egecioglu, E., J.A. Engel, and E. Jerlhag, *The glucagon-like peptide 1 analogue Exendin-4 attenuates the nicotine-induced locomotor stimulation, accumbal dopamine release, conditioned place preference as well as the expression of locomotor sensitization in mice.* PLoS One, 2013. **8**(10): p. e77284.
33. Anderberg, R.H., et al., *Glucagon-Like Peptide 1 and Its Analogs Act in the Dorsal Raphe and Modulate Central Serotonin to Reduce Appetite and Body Weight.* Diabetes, 2017. **66**(4): p. 1062-1073.
34. Howell, E., et al., *Glucagon-Like Peptide-1 (GLP-1) and 5-Hydroxytryptamine 2c (5-HT(2c)) Receptor Agonists in the Ventral Tegmental Area (VTA) Inhibit Ghrelin-Stimulated Appetitive Reward.* Int J Mol Sci, 2019. **20**(4).
35. Asarian, L., *Loss of cholecystokinin and glucagon-like peptide-1-induced satiation in mice lacking serotonin 2C receptors.* American Journal of Physiology-Regulatory, Integrative and Comparitive Physiology, 2009. **296**(1): p. R51-R56.
36. Nonogaki, K., et al., *The contribution of serotonin 5-HT2C and melanocortin-4 receptors to the satiety signaling of glucagon-like peptide 1 and liraglutide, a glucagon-like peptide 1 receptor agonist, in mice.* Biochem Biophys Res Commun, 2011. **411**(2): p. 445-8.
37. Doggrell, S.A., *Sgemaglutide in type 2 diabetes - is it the best glucagon-like peptide 1 receptor agonist (GLP-1R agonist)?* Expert Opin Drug Metab Toxicol, 2018. **14**(3): p. 371-377.
38. Litten, R.Z., et al., *Development of medications for alcohol use disorders: recent advances and ongoing challenges.* Expert Opin Emerg Drugs, 2005. **10**(2): p. 323-43.
39. Bujarski, S. and L.A. Ray, *Experimental psychopathology paradigms for alcohol use disorders: Applications for translational research.* Behav Res Ther, 2016.
40. Hendershot, C.S., et al., *Effects of naltrexone on alcohol self-administration and craving: meta-analysis of human laboratory studies.* Addict Biol, 2016.
41. McKee, S.A., *Developing human laboratory models of smoking lapse behavior for medication screening.* Addict Biol, 2009. **14**(1): p. 99-107.
42. Plebani, J.G., et al., *Human laboratory paradigms in alcohol research.* Alcohol Clin Exp Res, 2012. **36**(6): p. 972-83.
43. Ray, L.A., K.E. Hutchison, and M. Tartter, *Application of human laboratory models to pharmacotherapy development for alcohol dependence.* Curr Pharm Des, 2010. **16**(19): p. 2149-58.
44. Lerman, C., et al., *Translational research in medication development for nicotine dependence.* Nat Rev Drug Discov, 2007. **6**(9): p. 746-62.
45. MacKillop, J. and J.G. Murphy, *A behavioral economic measure of demand for alcohol predicts brief intervention outcomes.* Drug Alcohol Depend, 2007. **89**(2-3): p. 227-33.
46. Amlung, M., et al., *Increased behavioral economic demand and craving for alcohol following a laboratory alcohol challenge.* Addiction, 2015. **110**(9): p. 1421-8.
47. Green, R. and L.A. Ray, *Effects of varenicline on subjective craving and relative reinforcing value of cigarettes.* Drug Alcohol Depend, 2018. **188**: p. 53-59.

48. Murphy, C.M., et al., *Effects of varenicline versus transdermal nicotine replacement therapy on cigarette demand on quit day in individuals with substance use disorders.* Psychopharmacology (Berl), 2017. **234**(16): p. 2443-2452.
49. Roache, J.D., *Role of the Human Laboratory in the Development of Medications for Alcohol and Drug Dependence*, in *Addiction Medicine*, B.A. Johnson, Editor. 2010, Springer: New York. p. 129-157.
50. Enoch, M.A., et al., *Ethical considerations for administering alcohol or alcohol cues to treatment-seeking alcoholics in a research setting: can the benefits to society outweigh the risks to the individual? A commentary in the context of the National Advisory Council on Alcohol Abuse and Alcoholism -- Recommended Council Guidelines on Ethyl Alcohol Administration in Human Experimentation (2005).* Alcohol Clin Exp Res, 2009. **33**(9): p. 1508-12.
51. Motschman, C.A., et al., *Selection criteria limit generalizability of smoking pharmacotherapy studies differentially across clinical trials and laboratory studies: A systematic review on varenicline.* Drug Alcohol Depend, 2016. **169**: p. 180-189.
52. Alcoholism, N.I.o.A.A.a., *Recommended council guidelines on ethyl alcohol administration in human experimentation*. 2005, Department of Health and Human Services: Rockville, MD.
53. First MB, W.J., Karg RS, Spitzer RL, *Structured Clinical Interview for DSM-5—Research Version (SCID-5 for DSM-5, Research Version; SCID-5-RV)*. 2015, Arlington, VA: American Psychiatric Association.
54. Sobell, L.C., et al., *Reliability of a timeline method: assessing normal drinkers' reports of recent drinking and a comparative evaluation across several populations.* Br J Addict, 1988. **83**(4): p. 393-402.
55. Saunders, J.B., et al., *Development of the Alcohol Use Disorders Identification Test (AUDIT): WHO Collaborative Project on Early Detection of Persons with Harmful Alcohol Consumption--II.* Addiction, 1993. **88**(6): p. 791-804.
56. Schuckit, M.A., et al., *The Self-Rating of the Effects of Alcohol Questionnaire as a Predictor of Alcohol-Related Outcomes in 12-Year-Old Subjects.* Alcohol and Alcoholism, 2008. **43**(6): p. 641-646.
57. Schuckit, M.A. and T.L. Smith, *The relationships of a family history of alcohol dependence, a low level of response to alcohol and six domains of life functioning to the development of alcohol use disorders.* Journal of Studies on Alcohol, 2000. **61**(6): p. 827-835.
58. Heatherton, T.F., et al., *The Fagerstrom Test for Nicotine Dependence: a revision of the Fagerstrom Tolerance Questionnaire.* Br J Addict, 1991. **86**(9): p. 1119-27.
59. Shiffman, S., A. Waters, and M. Hickcox, *The nicotine dependence syndrome scale: a multidimensional measure of nicotine dependence.* Nicotine Tob Res, 2004. **6**(2): p. 327-48.
60. Patton, J.H., M.S. Stanford, and E.S. Barratt, *Factor structure of the Barratt impulsiveness scale.* Journal of clinical psychology, 1995. **51**(6): p. 768-774.
61. Whiteside, S.P., et al., *Validation of the UPPS impulsive behaviour scale: a four-factor model of impulsivity.* European Journal of personality, 2005. **19**(7): p. 559-574.
62. Heather, N., et al., *Development of a scale for measuring impaired control over alcohol consumption: a preliminary report.* Journal of studies on alcohol, 1993. **54**(6): p. 700-709.
63. Radloff, L.S., *The CES-D scale: A self-report depression scale for research in the general population.* Applied psychological measurement, 1977. **1**(3): p. 385-401.
64. Leising, D., T. Grande, and R. Faber, *The Toronto Alexithymia Scale (TAS-20): A measure of general psychological distress.* Journal of research in personality, 2009. **43**(4): p. 707-710.
65. Carvalho, J.P., et al., *The Reward Probability Index: Design and validation of a scale measuring access to environmental reward.* Behavior therapy, 2011. **42**(2): p. 249-262.
66. Tiffany, S.T. and D.J. Drobes, *The development and initial validation of a questionnaire on smoking urges.* Br J Addict, 1991. **86**(11): p. 1467-76.
67. Welsch, S.K., et al., *Development and validation of the Wisconsin Smoking Withdrawal Scale.* Exp Clin Psychopharmacol, 1999. **7**(4): p. 354-61.
68. DiClemente, C.C., et al., *The Alcohol Abstinence Self-Efficacy scale.* J Stud Alcohol, 1994. **55**(2): p. 141-8.
69. Gwaltney, C.J., et al., *Does smoking abstinence self-efficacy vary across situations? Identifying context-specificity within the Relapse Situation Efficacy Questionnaire.* J Consult Clin Psychol, 2001. **69**(3): p. 516-27.

70. Mann, K., et al., *Precision Medicine in Alcohol Dependence: A Controlled Trial Testing Pharmacotherapy Response Among Reward and Relief Drinking Phenotypes.* Neuropsychopharmacology, 2018. **43**(4): p. 891-899.
71. Sties, S.W., et al., *Simplified Nutritional Appetite Questionnaire (SNAQ) for cardiopulmonary and metabolic rehabilitation program.* Revista Brasileira de Medicina do Esporte, 2012. **18**: p. 313-317.
72. Dalton, M., et al., *Preliminary validation and principal components analysis of the Control of Eating Questionnaire (CoEQ) for the experience of food craving.* European journal of clinical nutrition, 2015. **69**(12): p. 1313-1317.
73. Lesdema, A., et al., *Characterization of the Three-Factor Eating Questionnaire scores of a young French cohort.* Appetite, 2012. **59**(2): p. 385-90.
74. Epel, E.S., et al., *The reward-based eating drive scale: a self-report index of reward-based eating.* PLoS One, 2014. **9**(6): p. e101350.
75. Johnson, B.A., N. Ait-Daoud, and J.D. Roache, *The COMBINE SAFTEE: a structured instrument for collecting adverse events adapted for clinical studies in the alcoholism field.* J Stud Alcohol Suppl, 2005(15): p. 157-67; discussion 140.
76. Kaplan, B.A., et al., *Automating scoring of delay discounting for the 21-and 27-item monetary choice questionnaires.* The Behavior Analyst, 2016. **39**(2): p. 293-304.
77. MacKillop, J., et al., *Further validation of a cigarette purchase task for assessing the relative reinforcing efficacy of nicotine in college smokers.* Experimental and clinical psychopharmacology, 2008. **16**(1): p. 57.
78. Epstein, L.H., et al., *Reinforcing value and hypothetical behavioral economic demand for food and their relation to BMI.* Eating behaviors, 2018. **29**: p. 120-127.
79. MacKillop, J. and J.G. Murphy, *A behavioral economic measure of demand for alcohol predicts brief intervention outcomes.* Drug and alcohol dependence, 2007. **89**(2-3): p. 227-233.
80. Jensterle, M., et al., *Does intervention with GLP-1 receptor agonist semaglutide modulate perception of sweet taste in women with obesity: study protocol of a randomized, single-blinded, placebo-controlled clinical trial.* Trials, 2021. **22**(1): p. 1-12.
81. Kampov-Polevoy, A.B., et al., *Association between sweet preference and paternal history of alcoholism in psychiatric and substance abuse patients.* Alcoholism: Clinical and Experimental Research, 2003. **27**(12): p. 1929-1936.
82. Weafer, J., S.H. Mitchell, and H. de Wit, *Recent translational findings on impulsivity in relation to drug abuse.* Current addiction reports, 2014. **1**(4): p. 289-300.
83. Brick, J., *Standardization of alcohol calculations in research.* Alcohol Clin Exp Res, 2006. **30**(8): p. 1276-87.
84. Martin, C.S., et al., *DEVELOPMENT AND VALIDATION OF THE BIPHASIC ALCOHOL EFFECTS SCALE.* Alcoholism-Clinical and Experimental Research, 1993. **17**(1): p. 140-146.
85. Bohn, M.J., D.D. Krahn, and B.A. Staehler, *DEVELOPMENT AND INITIAL VALIDATION OF A MEASURE OF DRINKING URGES IN ABSTINENT ALCOHOLICS.* Alcoholism-Clinical and Experimental Research, 1995. **19**(3): p. 600-606.
86. Johanson, C.E. and E.H. Uhlenhuth, *Drug preference and mood in humans: diazepam.* Psychopharmacology, 1980. **71**(3): p. 269-73.
87. MacKillop, J., et al., *Further validation of a cigarette purchase task for assessing the relative reinforcing efficacy of nicotine in college smokers.* Exp Clin Psychopharmacol, 2008. **16**(1): p. 57-65.
88. Curran, S.L., M.A. Andrykowski, and J.L. Studts, *Short form of the profile of mood states (POMS-SF): psychometric information.* Psychological assessment, 1995. **7**(1): p. 80.

Human Laboratory Screening of Semaglutide for Alcohol Use Disorder

Protocol Number: 21-1689

National Clinical Trial (NCT) Identified Number: Pending

Investigators:

Dr. Christian Hendershot (Principal Investigator)
Dr. Robyn Jordan (Co-Investigator)
Dr. Amanda Tow (Co-Investigator)
Dr. Sarah Dermody (Co-Investigator, Biostatistician)
Dr. Eric Claus (Co-Investigator)
Dr. Klara Klein (Co-Investigator)
Dr. Paul Fletcher (Other Significant Contributor)

Funded by: NIAAA

Version Number: v.1.3

4 March 2022

Summary of Changes from Previous Version:

**Affected Section(s)**	**Summary of Revisions Made**	**Rationale**
2.3.3<br>4.1<br>4.2<br>4.3<br>5.2<br>5.5<br>6.1.2<br>6.2.1<br>6.2.2<br>6.2.3<br>6.3<br>6.4<br>7.1<br>7.2<br>7.3<br>8.2	Added clarification of medication dispensing procedures and clarification of thresholds for selected inclusion/exclusion criteria and stopping rules. Added Dr. Klara Klein (Endocrinology) as a Co-I. Added the the Clinical Research Unit at Eastowne as a visit site. Added additional procedural clarifications and safety checks. Added remote consent procedures (Docusign), and removed the use of REDCap (replacing with paper questionnaires). Minor	Clarifications to medication/placebo dispensing and storage are based on ongoing conversations with IDS and the study medical team. We have clarified specific cutoffs for certain inclusion/exclusion criteria (e.g., BMI, blood glucose) and stopping rules, and clarified other procedure details, in consultation with the study medical team. We have added the Eastowne CRU as the primary visit site based on Dr. Klein's location, as well as additional convenience factors (e.g., parking). REDCap was removed due to

8.3.4<br>8.3.6<br>9.3<br>9.4.6<br>10.1.1<br>10.1.1.1<br>10.1.1.2<br>10.1.3<br><br>10.1.5	additions to self-report questionnaires and other minor procedural clarifications.	compliance restrictions (Part 11) preventing use of this software in clinical trials at UNC.

1 TABLE OF CONTENTS

STATEMENT OF COMPLIANCE 1

1 PROTOCOL SUMMARY 1

1.1 Synopsis 1

1.2 Schema 3

1.3 Schedule of Activities (SoA) 4

2 INTRODUCTION 5

2.1 Study Rationale 5

2.2 Background 6

2.3 Risk/Benefit Assessment 8

2.3.1 Known Potential Risks 8

2.3.2 Known Potential Benefits 10

2.3.3 Assessment of Potential Risks and Benefits 10

3 OBJECTIVES AND ENDPOINTS 11

4 STUDY DESIGN 13

4.1 Overall Design 13

4.2 Scientific Rationale for Study Design 21

4.3 Justification for Dose 22

4.4 End of Study Definition 22

5 STUDY POPULATION 22

5.1 Inclusion Criteria 22

5.2 Exclusion Criteria 23

5.3 Lifestyle Considerations 24

5.4 Screen Failures 25

5.5 Strategies for Recruitment and Retention 25

6 STUDY INTERVENTION 26

6.1 Study Intervention(s) Administration 26

6.1.1 Study Intervention Description 26

6.1.2 Dosing and Administration 26

6.2 Preparation/Handling/Storage/Accountability 27

6.2.1 Acquisition and accountability 27

6.2.2 Formulation, Appearance, Packaging, and Labeling 28

6.2.3 Product Storage and Stability 28
6.2.4 Preparation 28
6.3 Measures to Minimize Bias: Randomization and Blinding 28
6.4 Study Intervention Compliance 29
6.5 Concomitant Therapy 29
6.5.1 Rescue Medicine 29
7 STUDY INTERVENTION DISCONTINUATION AND PARTICIPANT DISCONTINUATION/WITHDRAWAL 29
7.1 Discontinuation of Study Intervention 29
7.2 Participant Discontinuation/Withdrawal from the Study 30
7.3 Lost to Follow-Up 31
8 STUDY ASSESSMENTS AND PROCEDURES 31
8.1 Efficacy Assessments 31
8.2 Safety and Other Assessments 32
8.3 Adverse Events and Serious Adverse Events 33
8.3.1 Definition of Adverse Events (AE) 33
8.3.2 Definition of Serious Adverse Events (SAE) 33
8.3.3 Classification of an Adverse Event 33
8.3.4 Time Period and Frequency for Event Assessment and Follow-Up 34
8.3.5 Adverse Event Reporting 34
8.3.6 Serious Adverse Event Reporting 35
8.3.7 Reporting Events to Participants 35
8.3.8 Events of Special Interest 36
8.3.9 Reporting of Pregnancy 36
8.4 Unanticipated Problems 36
8.4.1 Definition of Unanticipated Problems (UP) 36
8.4.2 Unanticipated Problem Reporting 36
8.4.3 Reporting Unanticipated Problems to Participants 37
9 STATISTICAL CONSIDERATIONS 37
9.1 Statistical Hypotheses 37
9.2 Sample Size Determination 37
9.3 Populations for Analyses 38
9.4 Statistical Analyses 38
9.4.1 General Approach 38
9.4.2 Analysis of the Primary Efficacy Endpoint(s) 39
9.4.3 Analysis of the Secondary Endpoint(s) 39
9.4.4 Safety Analyses 39

9.4.5 Baseline Descriptive Statistics 39
9.4.6 Planned Interim Analyses 39
9.4.7 Sub-Group Analyses 39
9.4.8 Tabulation of Individual participant Data 40
9.4.9 Exploratory Analyses 40

10 SUPPORTING DOCUMENTATION AND OPERATIONAL CONSIDERATIONS. 40

10.1 Regulatory, Ethical, and Study Oversight Considerations 40

10.1.1 Informed Consent Process 40
10.1.2 Study Discontinuation and Closure 41
10.1.3 Confidentiality and Privacy 41
10.1.4 Future Use of Stored Specimens and Data 42
10.1.5 Key Roles and Study Governance 42
10.1.6 Clinical Monitoring 43
10.1.7 Quality Assurance and Quality Control 43
10.1.8 Data Handling and Record Keeping 43
10.1.9 Protocol Deviations 44
10.1.10 Publication and Data Sharing Policy 44
10.1.11 Conflict of Interest Policy 44

10.2 Additional Considerations 44

10.3 Abbreviations 44

10.4 Protocol Amendment History 47

11 REFERENCES 49

STATEMENT OF COMPLIANCE

(1) The trial will be carried out in accordance with International Conference on Harmonization Good Clinical Practice (ICH GCP) and the following:

- United States (US) Code of Federal Regulations (CFR) applicable to clinical studies (45 CFR Part 46, 21 CFR Part 50, 21 CFR Part 56, 21 CFR Part 312, and/or 21 CFR Part 812)

National Institutes of Health (NIH)-funded investigators and clinical trial site staff who are responsible for the conduct, management, or oversight of NIH-funded clinical trials have completed Human Subjects Protection and ICH GCP Training.

The protocol, informed consent form(s), recruitment materials, and all participant materials will be submitted to the Institutional Review Board (IRB) for review and approval. Approval of both the protocol and the consent form must be obtained before any participant is enrolled. Any amendment to the protocol will require review and approval by the IRB before the changes are implemented to the study. In addition, all changes to the consent form will be IRB-approved; a determination will be made regarding whether a new consent needs to be obtained from participants who provided consent, using a previously approved consent form.

1 PROTOCOL SUMMARY

1.1 SYNOPSIS

Title: Human Laboratory Screening of Semaglutide for Alcohol Use Disorder

Study Description: This is an early-Phase II human laboratory trial using a randomized, placebo-controlled, dose-ranging design to investigate the effects of semaglutide (Ozempic®), a GLP-1 receptor agonist, on laboratory alcohol responses and consumption, naturalistic alcohol and cigarette consumption, and weight loss in smokers with alcohol use disorder (AUD). Participants will attend weekly visits while semaglutide dosage is increased to 1.0mg over a period of approximately 10 weeks. Participants will attend weekly visits for medication or placebo administration. At scheduled intervals, participants will complete 4 laboratory sessions involving alcohol self-administration and alcohol challenge to characterize medication effects on alcohol-related outcomes.

Objectives: Primary Objective: To examine effects of semaglutide vs. placebo on laboratory alcohol intake during a validated alcohol self-administration medication screening procedure.

Secondary Objectives: To examine medication vs. placebo group differences in 1) laboratory responses to alcohol, and 2) changes in naturalistic alcohol use and cigarette consumption over the course of the treatment period.

	Tertiary Objectives: To generate preliminary data on weight loss, HbA1c reductions and alcohol elimination among participants with AUD during treatment with a GLP-1 receptor agonist.
**Endpoints:**	Primary Endpoint: Change in amount of alcohol consumed during a validated laboratory alcohol self-administration protocol (primary endpoint). The specific effect of interest is the change in laboratory consumption from baseline to the maintenance dose (.5mg) as a function of medication condition (semaglutide vs. placebo) (i.e., treatment x time interaction).<br><br>Secondary Endpoints: Secondary endpoints will include laboratory measures of alcohol and cigarette demand (behavioral economic indices of drug motivation) and subjective responses to alcohol during an alcohol challenge procedure, and changes in naturalistic alcohol use and cigarette smoking over the treatment period. Within-person changes in these outcomes before vs. after treatment (baseline vs. 0.5mg) will be compared as a function of medication condition.
**Study Population:**	Participants who meet criteria for mild or moderate alcohol use disorder/AUD (N=48) and smoke cigarettes daily will be enrolled. Participants will be recruited from the Triangle area (community and hospital settings). Participants must not be seeking current treatment for alcohol or smoking cessation.
**Phase:**	Phase II
**Description of Sites/Facilities Enrolling Participants:**	This is a single-site study (UNC-Chapel Hill). The project will be based at the Bowles Center for Alcohol studies and the Department of Psychiatry, with clinical operations taking place at Eastowne Clinical Research Unit and CTRC.
**Description of Study Intervention:**	Semaglutide (Ozempic®) will be administered according to the FDA-approved schedule, which begins with a starting dose of 0.25mg once per week for four weeks. At week five, dosage is increased to 0.5mg/week for a subsequent four weeks (maintenance dose). Following four weeks at the maintenance dose, dosage will be increased to 1.0mg provided the patient has tolerated the medication. Semaglutide is administered subcutaneously via the Ozempic® pen, a cartridge-based device that calibrates medication delivery. The placebo condition will consist of weekly sham doses, which will involve a brief needle insertion (without an injection).
**Study Duration:**	Up to 13 months.
**Participant Duration:**	Up to 40-50 hours total (including in-person visits and remote questionnaires) over a period of approximately 12 weeks.

1.2 SCHEMA

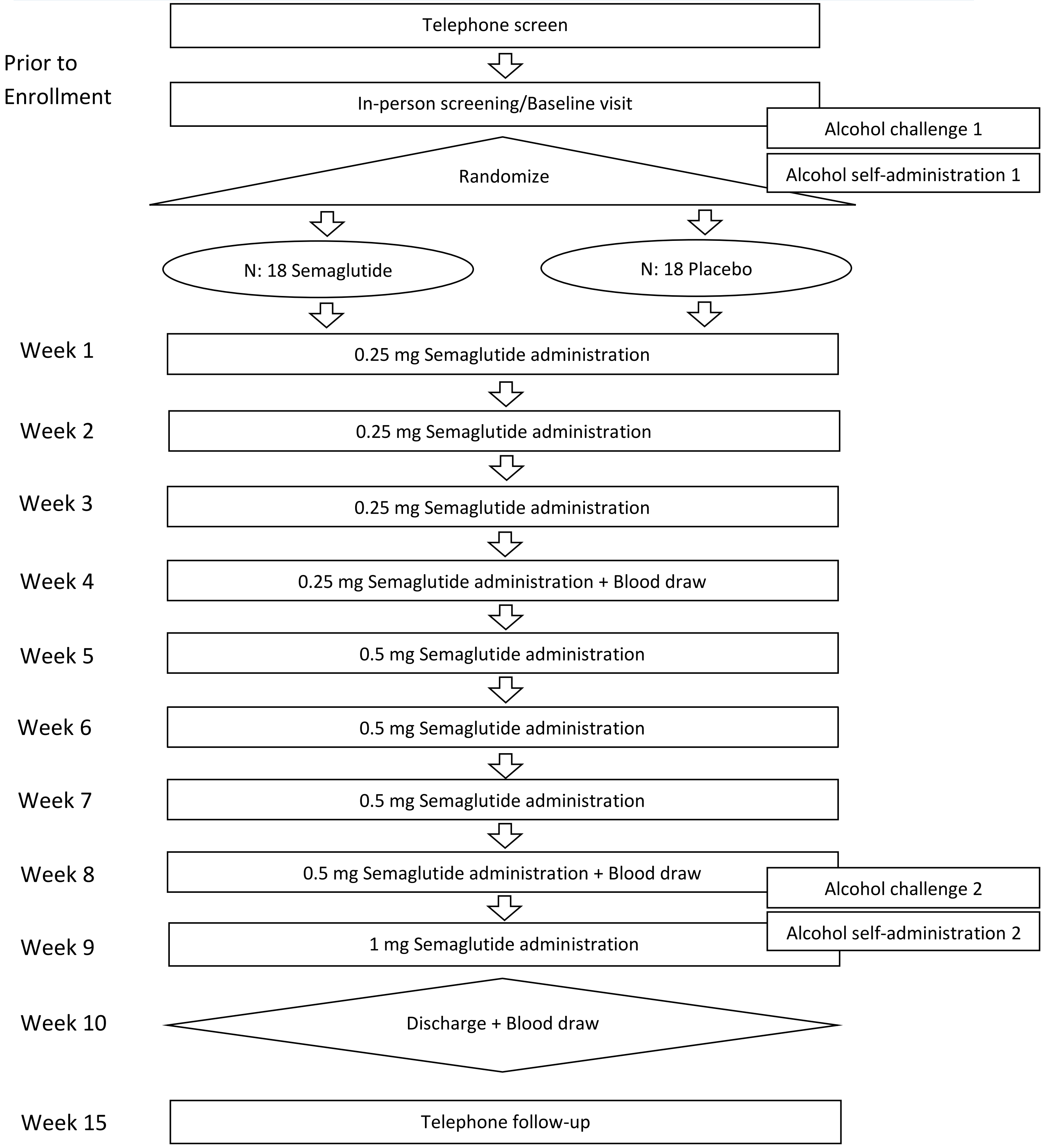
Telephone screen
Prior to Enrollment
In-person screening/Baseline visit
Alcohol challenge 1
Alcohol self-administration 1
Randomize
N: 18 Semaglutide
N: 18 Placebo
Week 1
0.25 mg Semaglutide administration
Week 2
0.25 mg Semaglutide administration
Week 3
0.25 mg Semaglutide administration
Week 4
0.25 mg Semaglutide administration + Blood draw
Week 5
0.5 mg Semaglutide administration
Week 6
0.5 mg Semaglutide administration
Week 7
0.5 mg Semaglutide administration
Week 8
0.5 mg Semaglutide administration + Blood draw
Alcohol challenge 2
Alcohol self-administration 2
Week 9
1 mg Semaglutide administration
Week 10
Discharge + Blood draw
Week 15
Telephone follow-up

1.3 SCHEDULE OF ACTIVITIES (SOA)

**Procedures and Questionnaires**	Telephone Screening	Screening/Baseline	Study Visit 1 - 3<br>Target Day 7, 14, 21	Study Visit 4<br>Target Day 28	Study Visit 5 - 7<br>Target Day 35, 42, 49	Study Visit 8<br>Target Day 56	Study Visit 9<br>Target Day 63	Discharge<br>Target Day 70	Telephone follow-up<br>Target Day 77, 105	Alcohol Challenge<br>Target Day -10, 58	Alcohol Self-Admin.<br>Target Day -12, 60
Telephone screening	X										
Administer 0.25 mg Semaglutide or placebo			X	X							
Administer 0.5 mg Semaglutide or placebo					X	X					
Administer 1.0 mg Semaglutide or placebo							X				
Breath Alcohol Concentration (BrAC) Test		X	X	X	X	X	X	X		X	X
Urine Drug Screen		X								X	X
Expired carbon monoxide (CO)		X	X	X	X	X	X	X		X	X
Locator		X									
Timeline Follow-Back (TLFB)		X	X	X	X	X	X	X			
BAC		X	X	X	X	X	X	X		X	X
Blood Draw		X		X		X		X			
Blood glucose – finger prick		X	X		X		X			X	X
Pregnancy (females only)		X	X	X	X	X	X	X		X	X
BMI		X	X	X	X	X	X	X		X	X
Height		X									
Vitals (HR, BP)		X	X	X	X	X	X	X		X	X
Weight		X	X	X	X	X	X	X		X	X
Temperature		X									
Demographics Form		X									
Structured Clinical Interview for DSM-5		X									
MINI Neuropsychiatric Interview		X									
Medical and Substance Use History Interviews		X									
Saliva Collection for DNA and nicotine metabolite		X									
Case Report Forms (CRFs)	X	X	X	X	X	X	X	X	X	X	X
Alcohol Use Disorder Identification Test (AUDIT)		X									
Alcohol, Cigarette, and Food Purchase Test		X								X	X
Self-Report of the Effects of Alcohol Questionnaire (SRE)		X									
Fagerstrom Test for Nicotine Dependence (FTND)		X									
Nicotine Dependence Syndrome Scale (NDSS)		X									
Penn Alcohol Craving Scale (PACS)		X	X	X	X	X	X	X		X	X
Tiffany Questionnaire of Smoking Urges (TQSU)		X	X	X	X	X	X	X		X	X
Wisconsin Smoking Withdrawal Scale (WSWS)		X	X	X	X	X	X	X		X	X
Alcohol and Smoking Contemplation		X	X	X	X	X	X	X			
Three Factor Eating Questionnaire (TFEQ)		X	X	X	X	X	X	X		X	X
Monetary Choice Questionnaire (MCQ)		X	X	X	X	X	X	X		X	X
Cigarette Abstinence Self-Efficacy (CASE)		X	X	X	X	X	X	X		X	X
Alcohol Abstinence Self-Efficacy (AASE)		X	X	X	X	X	X	X		X	X
Inventory of Drinking Situations (IDS)		X									
Impaired Control Scale (ICS)		X									
Barratt Impulsivity Scale (BIS-11)		X									
UPPS Impulsive Behavior Scale (UPPS)		X									
Systematic Assessment for Treatment Emergent Effects (SAFTEE)		X	X	X	X	X	X	X	X	X	X
Reward Based Eating Drive Scale (R-BEDS)		X	X	X	X	X	X	X		X	X
Simplified Nutritional Appetite Questionnaire (SNAQ)		X	X	X	X	X	X	X		X	X

Control of Eating Questionnaire (CEQ)		X	X	X	X	X	X	X		X	X
Wisconsin Inventory of Smoking Dependence Motives (WISDM)		X									
Sweet Taste Preference Test		X						X			
Center for Epidemiological Studies Depression Scale (CES-D)		X									
Reward Probability Index – Environmental Reward Observation Scale (RPI-EROS)		X									
Toronto Alexithymia Scale (TAS-20)		X									
Profile of Mood States – Short Form (POMS-SF)										X	X
Biphasic Alcohol Effects Scale (BAES)										X	X
Alcohol Urge Questionnaire (AUQ)										X	X
Drug Effects Questionnaire (DEQ)										X	X

2 INTRODUCTION

2.1 STUDY RATIONALE

Pharmacotherapy development is a critical objective for reducing health and societal burdens associated with alcohol use disorder (AUD) [1-3]. Despite advances in this area, medication development remains a slow- moving endeavor with a protracted path from drug discovery to market [2-4]. Given the high degree of heterogeneity in AUD clinical presentation and treatment response, it is recognized that no single medication will be effective for all patients [5]. Therefore, the National Institute on Alcohol Abuse and Alcoholism (NIAAA) medication development strategy specifies the long-term objective of establishing a repertoire of therapies to facilitate targeted interventions for specific AUD subgroups [3, 5, 6]. Strategies for expediting the study of novel treatments are central to these aims [3, 5, 6].

Epidemiological and clinical evidence shows that cigarette smokers comprise a sizable AUD subgroup with disproportionately high health burdens [7, 8] Alcohol and cigarette use are two of the three leading causes of preventable mortality worldwide, jointly accounting for an estimated seven million deaths per year [9]. Population-based research shows that cigarette consumption increases as a function of alcohol use severity [7]. In one large epidemiological study, nearly half (44.6% of men, 47.3% of women) of those with alcohol dependence also met criteria for nicotine dependence [7]. Co-use of alcohol and cigarettes predicts synergistic increases in risk for numerous disease conditions [8, 10, 11], including esophageal and laryngeal cancers [12, 13]. Given these health burdens, increasing effort has been allocated to developing targeted interventions for smokers with AUD [8, 11, 14, 15].

In the absence of FDA-approved medications for concurrent alcohol and nicotine addiction, identifying candidate drugs for this indication is a priority [11, 15, 16]. Several recent studies have examined whether FDA- approved AUD and smoking cessation drugs, either alone or in combination, can facilitate joint reductions in alcohol and cigarette use [11]. For instance, naltrexone reduces alcohol consumption in heavy-drinking smokers [17, 18], and in some cases reduces cigarette consumption [17, 19]. Recent findings further suggest that naltrexone's efficacy for reducing drinking is stronger in AUD participants who smoke relative to non-smokers [20, 21]. However, a Cochrane review [22] and a large, prospective AUD trial [19, 23] concluded that naltrexone does not appear to reduce cigarette smoking [11, 23].

Varenicline has also been tested as a candidate therapy for smokers with heavy drinking or AUD. Informed by early findings that varenicline reduced alcohol self-administration in a human laboratory

trial [16], large-scale clinical trials supported efficacy of varenicline for reducing alcohol consumption in heavy-drinking smokers [24, 25] and those with AUD [26]. Recent laboratory-based studies have also examined the combination of varenicline and naltrexone as a novel treatment [27, 28]. Because those with concurrent alcohol and nicotine addiction reflect a high-priority subgroup, further efforts are needed to screen candidate treatments for this population [8, 11, 28] . As reflected in these recent trials, and as noted explicitly under the NIAAA medication development framework [1-3], drug repurposing strategies can significantly expedite new treatment approaches by capitalizing on therapies that have already passed the extensive regulatory steps required for FDA approval.

Compounds with preclinical evidence for reducing both alcohol and nicotine intake are particularly good candidates for investigating new treatments for heavy-drinking smokers. Accumulating evidence suggests that *Glucagon-like peptide 1* (GLP-1) receptor agonists may have promise in this respect. GLP-1 is an incretin hormone produced and secreted both peripherally (in the intestines) and centrally (in the hindbrain) [29]. GLP-1 is stimulated following food consumption and stimulates satiety and decreases in blood glucose. As a peptide with both hormonal and neurotransmitter functions, GLP-1 plays a role in the "gut-brain axis," which has recently been invoked as a target for addiction therapies [30]. GLP-1 agonist medications were designed to mimic the function of the endogenous GLP-1 peptide, with the benefit of a much longer half-life than endogenous GLP-1. Based on their glucose-lowering and appetite-reducing properties, GLP-1 agonists were developed as anti-diabetic therapies.

Several GLP-1 receptor agonists are now in widespread use as treatments for type II diabetes (T2D), including exenatide, liraglutide, lixisenatide, albiglutide, dulaglutide and semaglutide. These medications have also proven efficacious for promoting weight loss, reducing glycated hemoglobin (HbA1c), and reducing risk for some cardiovascular events [29]. Although the primary indication of GLP-1 agonists is T2D, these therapies are increasingly being studied for other indications in non-diabetic patients, including obesity/weight management.

Of note, GLP-1 agonists require subcutaneous administration, typically by way of a pre-filled medication cartridge with a micro-needle attachment, which allows for easy self-administration in the home without assistance from medical staff. Semaglutide is a recently approved (2017) FDA-approved GLP-1 agonist. A new oral formulation of semaglutide was approved by the FDA in 2019, making semaglutide the first drug in its class to be available in both subcutaneous and oral formulations.

2.2 BACKGROUND

Accumulating evidence from preclinical studies suggests that GLP-1 agonists reduce alcohol and other drug intake and/or reinstatement in animal models. In a recent review of 17 preclinical studies [31], all studies reported significant effects of GLP-1 agonists on indices of drug motivation and intake, with the evidence being most robust for alcohol [30, 31]. Notably, the effects of GLP-1 agonists on drug intake appear to extend to several addictive drugs, including nicotine [32]. These findings have led to recent studies aiming to investigate the efficacy of GLP-1 agonists as candidate therapies for reducing intake or reinstatement of alcohol, nicotine, and other drugs (e.g., cocaine). Although several human trials are underway in various regions, no RCTs have been published to date, and human data on the efficacy of GLP-1 agonists for reducing drug intake in substance-using populations is needed.

Although the precise mechanisms mediating the effects of GLP-1 agonists on alcohol-related behaviors remain to be clarified, it has been suggested that cholinergic-dopaminergic pathways are involved in altering the reward value of alcohol following exposure to GLP-1 agonists [30]. Additionally, GLP-1

agonists appear to exert their effects in part by way of interactions with the serotonin (5-HT) system, including interactions with 5-HT2C receptors. Specifically, GLP-1 agonists appear to induce serotonin release, and serotonin signaling appears critical to the efficacy of the medications [33, 34]. Some evidence also suggests that 5-HT2C receptors appear necessary for the efficacy and satiety-inducing effects of GLP-1 agonists [35, 36].

Of the approved FDA-approved GLP-1 agonists, most studies have focused on exenatide, an older GLP-1 agonist first approved in 2005. As noted, semaglutide is the newest GLP-1 agonist and the first available in both subcutaneous and oral formulations. Some evidence suggests superiority of semaglutide relative to other GLP-1 agonists with respect to efficacy for glycemic control [37]. Although clinical trials of GLP-1 receptor agonists in participants with substance use disorder are now underway, no studies (to our knowledge) are currently underway to examine semaglutide as a treatment for alcohol use disorder, or for the joint indication of alcohol use disorder and tobacco use disorder. Demonstrating tolerability and initial efficacy of semaglutide for these populations could ultimately allow for greater clinical flexibility if GLP-1 agonists prove efficacious for these populations, given that semaglutide is available in both subcutaneous and oral formulations.

Human laboratory methods for Phase II medication screening

As emphasized in the NIAAA medication development strategy [1, 4, 38], human laboratory methods play a pivotal role in drug development by providing a bridge from preclinical studies and full-scale Phase II/III trials [39-43]. Importantly, laboratory studies can expedite drug development by providing an efficient, cost-effective, and rigorous option for “early-Phase II” medication screening, thereby informing priorities for larger and more costly randomized trials. For example, we recently published meta-analytic evidence that human laboratory studies of naltrexone, when examined in aggregate, yield conclusions highly comparable to the aggregate data from large-scale randomized trials, reinforcing the notion that laboratory trials offer a valid and efficient approach for estimating the potential efficacy of candidate therapeutics [40]. Other advantages of laboratory trials include the ability to study putative treatment mechanisms under controlled conditions, and the ability to quickly generate initial data on drug tolerability in the clinical population of interest [1, 44].

Phase II pharmacotherapy trials for alcohol use disorder often rely on alcohol administration procedures to study medication effects on acute responses to alcohol in human laboratory settings [40]. Controlled alcohol exposure offers a means of testing medication effects on subjective response to alcohol (e.g., stimulation, sedation) and alcohol-induced craving. These subjective responses to alcohol are targets of existing pharmacotherapies, such as naltrexone [40]. Alcohol self-administration has also been used as a laboratory model of alcohol motivation, and has successfully been used to screen candidate AUD therapies [40]. Some have argued that alcohol self-administration should serve as the central endpoint in human laboratory studies, and validated procedures exist for modeling alcohol self-administration safely in the laboratory [16] [40]. More recently, behavioral economic measures have also been utilized as a means of studying drug motivation in laboratory contexts, and appear sensitive for detecting experimental manipulations on alcohol-related motivation. For example, *alcohol demand* indexes the relative reinforcing value of alcohol at a particular point in time [45]. Measures of drug demand, such as the alcohol purchase task (APT) and cigarette purchase task (CPT), are considered objective and sensitive experimental assays of drug motivation [45]. The APT predicts frequency of heavy drinking days after treatment [45], and is sensitive to temporal changes in alcohol’s relative reinforcing efficacy during drug administration [46]. Notably, initial evidence suggests that these measures are sensitive to the effects of pharmacological treatments, including naltrexone and varenicline [47, 48]. Measures of

drug demand offer an alternative to studying self-administration in the laboratory, complementing measures of subjective drug responses.

One limitation of many laboratory-based pharmacotherapy trials is the tendency to focus on heavy drinkers without AUD, rather than participants who report symptoms of the disorder [49, 50]. A recent systematic review found evidence that recruiting non-clinical samples for laboratory trials can potentially limit the ability of laboratory findings to generalize to larger clinical trials, perhaps undermining translational research efforts [51]. While ethical considerations normally preclude laboratory alcohol administration methods with treatment-seeking AUD participants [50], studies of *non-treatment-seeking* AUD participants are ethically justified, and can provide important data on medication effects and safety in the target clinical population [52]. Additionally, focusing on participants with *non-severe* (i.e., mild or moderate) AUD can ensure maximal safety when studying this population by eliminating the likelihood of clinically significant withdrawal or alcohol-related medical complications. Our team has ample prior experience with alcohol administration procedures in both clinical and non-clinical populations; we will utilize these methods to study the potential efficacy of semaglutide in participants with mild-to-moderate AUD.

2.3 RISK/BENEFIT ASSESSMENT

2.3.1 KNOWN POTENTIAL RISKS

Semaglutide (Ozempic®) received FDA approval in 2017 for use as an adjunct to diet and exercise to achieve glycemic control in adults with type 2 diabetes mellitus (T2D). Semaglutide received FDA approval based on the results of 25 completed trials with the goal of providing results that would be applicable to a broad type II diabetes (T2D) population, including the elderly, patients with renal impairment, and patients at high cardiovascular risk. These trials involved > 9,300 participants, of whom >5,700 were treated with semaglutide. In these trials, treatment with semaglutide alone was efficacious for reduction in hemoglobin A1C (HbA1c) below 7% (e.g., 73% of participants had HbA1c <7% at week 30, compared to 28% of placebo-treated patients). Significant body weight reductions were also reported, with the weight loss achieved at end-of-treatment ranging from -1.43 to -4.28 kg (corresponding to 2.3–4.9%). Weight reductions were evident after 4 weeks of treatment, and persisted up to 104 weeks [75]. The peak plasma concentration of semaglutide is reached 1-3 days post-dose. Steady-state exposure is achieved following 4-5 weeks of once-weekly administration, and elimination half-life is approximately 1 week.

<u>Risks related to medication.</u> The most commonly reported side effects/adverse events (AEs) are gastrointestinal, with nausea being the most frequently reported symptom. The majority of reports of nausea (0.5 mg dose vs placebo; 15.3 vs. 6.1%), vomiting (5.0 vs 2.3%), and/or diarrhea (8.5 vs 1.9%) occur shortly following a dose escalation. Abdominal pain (7.3 vs 4.6%) and constipation (5.0 vs 1.5%) were also reported [76]. In studies ranging from 30-56 weeks in duration, the discontinuation rate due to these gastrointestinal AEs was ranged from 5-13%, with this number increasing during longer treatment durations [77].

Rarer AEs (occurring at a frequency of <5%) included symptomatic hypoglycemia (≤70 mg/dL glucose threshold), which occurred in 1.6% of semaglutide-treated participants versus 0% placebo participants; dyspepsia (3.5 vs 1.9%); eructation (2.7 vs 0%); and gastroesophageal reflux disease (1.9 vs 0%). In

placebo-controlled trials, injection site reactions (e.g., discomfort, erythema) were reported in only 0.2% of semaglutide-treated patients, suggesting good tolerability of subcutaneous administration. Patients exposed to semaglutide had a mean increase from baseline in amylase of 13% and lipase of 22%. Cholelithiasis was reported in 1.5% of treated patients. The 0.5 mg dose resulted in a mean increase in heart rate of 2 to 3 beats per minute, while there was a mean decrease in heart rate of 0.3 beats per minute in placebo-treated patients. Hypersensitivity reactions (e.g., anaphylaxis, angioedema) have been reported with GLP-1 receptor agonists. [76]. Other adverse reactions with a frequency of >0.4% associated with semaglutide include fatigue, dysgeusia and dizziness [76].

Rodent studies have shown an increase in the incidence of thyroid C-cell tumors after lifetime exposure of semaglutide at clinically relevant plasma levels. However, several studies in T2D patients taking GLP-1R agonists have shown no increase in neoplasm risk, even after several years of use [78]. Nevertheless, a personal or family history of medullary thyroid cancer or multiple endocrine neoplasia 2A or 2B is considered a potential risk factor for taking the medication, and will be an exclusionary criterion.

In a 2-year trial, acute pancreatitis was observed in 8 semaglutide-treated patients (0.27 cases per 100 patient years) and 10 placebo-treated patients (0.33 cases per 100 patient years). One case of chronic pancreatitis was confirmed in a semaglutide-treated patient. History of pancreatitis will be an exclusion factor, and study participants receiving semaglutide will be observed for signs and symptoms of pancreatitis (via blood monitoring of serum amylase and lipase). If pancreatitis is suspected, semaglutide will be discontinued, and appropriate management recommended.

Other rare events include postmarketing reports of acute kidney injury and worsening of chronic renal failure, which may sometimes require hemodialysis, in patients receiving long-term treatment with GLP-1 receptor agonists. Some of these events have been reported in patients without known underlying renal disease. Most of these events occurred in patients who had experienced nausea, vomiting, diarrhea, or dehydration. Therefore, we will monitor renal function by including a measure of serum creatinine at baseline and at subsequent visits involving blood samples.

Although most safety data on semaglutide comes from trials with T2D participants, there are now a number of randomized trials underway examining GLP-1 receptor agonists in participants with substance use disorder, including trials on tobacco, cocaine, alcohol, cannabis, and opioids. A full list of registered randomized trials can be found by searching for “GLP-1” at ClinicalTrials.gov. This study will be the first to our knowledge to focus specifically on heavy-drinking smokers. The use of a short-term medication screening protocol (lasting roughly 10 weeks) will provide an opportunity to demonstrate tolerability and generate data on side effect profiles in this population while minimizing any risks associated with longer-term medication exposure.

<u>Risks Related to Questionnaire and Interview Assessments.</u> There may be potential risks associated with assessment procedures. Participants may find the battery of psychiatric and psychological assessments tedious or intrusive. Participants may experience discomfort resulting from questions about personal histories or substance use patterns.

<u>Participation in Alcohol Administration Sessions.</u> Other potential risks include typical risks related to participation in alcohol administration studies. For example, it is possible that participants may experience dizziness/nausea from drinking during these visits, or symptoms the following day (e.g., headache). Our group has considerable experience with alcohol administration and self- administration procedures [80-84], and the safety precautions required in these studies. Across these studies, the

incidence of adverse events such as vomiting has been exceptionally low. Our procedures will follow the NIAAA recommended guidelines for administering alcohol to human subjects (https://www.niaaa.nih.gov/Resources/ResearchResources/job22.htm). Briefly, the NIAAA guidelines include recommendations for administering alcohol to participants who meet criteria for AUD. In particular, we have taken measures to protect against ethical concerns by *excluding treatment-seekers* (or those engaged in an active quit attempt), as per the NIAAA recommended guidelines. Additionally, we will screen out participants with severe AUD (defined as 6 or more of 11 symptoms). This approach allows us to generate data relevant to a clinical population while minimizing risks.

Risks Related to DNA Collection. The purpose of collecting saliva for DNA in this study is to allow secondary analyses to examine whether medication effects might be different according to certain genetic factors. These analyses may also focus on genetic factors potentially associated with drug use or related behaviors, such as nicotine and alcohol metabolism, dopaminergic function, or impulsivity. Potential risks associated with DNA collection involve potential identification, and there may be risks with genetic (DNA) tests that are as yet unknown. Genes may be shown at some point in the future to be related to mental illnesses or tendency to addiction. Samples will be labeled with participant ID only, and analyzed at an outside laboratory. Samples will be de-identified and links with participant identifiers will be broken upon completion of the analyses. We will not conduct any whole genome sequencing of the DNA samples. Additionally, the consent form will state that participants have the option to opt out of DNA collection, and to request that their samples are destroyed at any time during the study. Participants will not receive any feedback from any of the genetic tests from study staff.

2.3.2 KNOWN POTENTIAL BENEFITS

The participants in this study are not expected to benefit directly from their participation. However, the knowledge gained from this study could lead to a better understanding of a medication with potential to treat alcohol and nicotine addiction, and potentially other substance use disorders.

2.3.3 ASSESSMENT OF POTENTIAL RISKS AND BENEFITS

Given the vast health implications of substance use disorder and the currently limited array of effective medications, it is imperative to pursue novel pharmacotherapeutic options. A key strategy in this regard is to “repurpose” medications that are already approved (and have proven safe) in the context of other health conditions. Given the sizable amount of research on GLP-1 receptor agonists in other clinical populations, coupled with preclinical evidence that these medications reduce motivation for alcohol, nicotine, and other drugs, evaluating this medication class in the proposed population is warranted. Given no available pharmacotherapies for the indication of concurrent alcohol and nicotine addiction, any evidence for potential efficacy in heavy-drinking smokers could have substantial implications. From this standpoint, the risk/benefit profile for testing semaglutide in a population of non-diabetic participants is favorable.

Although we anticipate the risk of adverse events to be low based on the safety profile of the medication, gradual dose escalation, and relatively short duration of treatment, several steps will be taken to track and report medication-related side effects. As described further in later sections of this application, participants will be monitored regularly by virtue of attending weekly lab visits for medication delivery, at which side effects will be assessed and clinical lab results monitored. Participants

will be given a contact number for study staff in case of emergent side effects (and instructed to seek medical attention in the event of any serious side effects), and participants will be contacted by phone shortly after dose escalations to monitor symptoms. We expect some participants to experience nausea shortly following dose escalations, but that these symptoms will usually be transient.

Given the focus on a non-obese population, we will be particularly vigilant in monitoring changes in body weight and blood glucose, and will exclude participants at the outset with body weight or blood glucose levels below normal range, based on physician judgement. We will carefully monitor participants with glucose tests and weight measurements at regular study visits (see schedule of activities table), and will instruct participants to be vigilant for potential symptoms of hypoglycemia. Periodic blood tests to measure fasting glucose, fasting insulin, serum lipase and amylase, serum creatinine, and serum calcitonin will occur at baseline and regularly thereafter (i.e., at visits prior to dose escalations). All adverse events will be documented and reported in accordance with relevant policies, and the regulations of NIH. We have enlisted the TraCS DSMB to assist with safety monitoring of the study. The board will convene at the beginning of the study and at scheduled points to review study progress and safety data, and to review any SAEs and considerations related to stopping rules (described further below).

Considering the use of an FDA approved medication that has been deemed safe in the context of chronic administration for glycemic control, the risks related to medication use in this short-term screening trial are acceptable in comparison to the potential benefits of the proposed research (i.e., demonstrating potential efficacy of a new medication for substance use disorder).

Risks Related to Alcohol Administration Sessions. Alcohol administration procedures are commonly used to study risk factors for alcohol use disorder (AUD), and have proven capable of identifying successful pharmacotherapies [40]. As per the NIAAA guidelines for administration of alcohol in human studies, such studies are typically restricted to non-treatment-seeking participants in order to minimize ethical concerns, as will be the approach in this study. Further details on safety procedures are provided later in this document.

3 OBJECTIVES AND ENDPOINTS

OBJECTIVES	ENDPOINTS	JUSTIFICATION FOR ENDPOINTS
Primary		
To examine effects of semaglutide vs. placebo on alcohol self-administration under laboratory conditions.	Amount of alcohol consumed and breath alcohol concentration (co-primary endpoints) during a validated laboratory alcohol self-administration protocol. In this procedure, participants are provided the option of choosing between drinks and monetary reward. The comparison of interest is the difference between medication and placebo groups in the change in laboratory consumption	Primary: This outcome is a laboratory assay of alcohol intake, and has been used in prior human laboratory studies to screen candidate medications, allowing comparisons with effect sizes for other medications. This session will be conducted during the standard maintenance dose (0.5mg) to mimic the common clinical scenario.<br><br>Secondary: Subjective alcohol responses and alcohol/cigarette demand are validated measures of drug reward/motivation, and are

OBJECTIVES	ENDPOINTS	JUSTIFICATION FOR ENDPOINTS
	between baseline and the maintenance dose of 0.5mg.	known to be sensitive to medication effects. Given substantial individual differences in alcohol pharmacokinetics, a within-subjects analysis is preferable for these endpoints (such that each participant will serve as their own control). Laboratory sessions will take place at two time points: baseline and 0.5mg maintenance dose.
Secondary		
To examine group differences in 1) alcohol/cigarette demand and subjective responses to alcohol during alcohol administration, and 2) naturalistic drinking and smoking behaviors over the course of the 10-week treatment period.	Laboratory measures of subjective responses to alcohol, alcohol demand, and cigarette demand during controlled laboratory alcohol administration (lab sessions will take place at baseline and again after achieving the maintenance dose).<br><br>Number of standard drinks consumed and number of cigarettes consumed per day during the treatment phase (Weeks 0-10), as measured by a daily diary report.<br><br>Similar to the primary outcome, secondary outcome analyses will examine medication vs. placebo group differences in within-subjects changes in these outcomes over time (baseline to 0.5mg).	Laboratory measures of alcohol response and alcohol/cigarette demand are clinically valid indicators of drug motivation, and are demonstrated to be sensitive to medication effects. Investigating these outcomes under controlled conditions will allow us to test for a medication "signal" on measures of alcohol and nicotine motivation.<br><br>Daily measures of alcohol consumption (standard drinks per day) and cigarettes per day are selected to afford greater power for modeling change in consumption over the treatment period. Note that these outcomes are considered secondary because this Phase II trial involves non-treatment-seeking participants who are not attempting to reduce alcohol or use or cigarette smoking.
Tertiary/Exploratory		
Body weight, HbA1c, alcohol elimination.	Changes in body weight, HbA1c, and alcohol elimination will be examined as exploratory endpoints based on potential clinical relevance.	GLP-1 receptor agonists lower HbA1c and body weight in indicated populations (e.g., Type II diabetes, obesity). Given high co-occurrence of obesity and alcohol use disorder, reporting whether medication is associated with these reductions in the present sample is clinically relevant. Data from the alcohol administration sessions will be used to estimate whether medication predicts changes in alcohol elimination during treatment, and

OBJECTIVES	ENDPOINTS	JUSTIFICATION FOR ENDPOINTS
		whether changes in body weight account for any such differences. These outcomes are exploratory.

4 STUDY DESIGN

4.1 OVERALL DESIGN

Overview of Study Design.

The aim of this protocol is to translate preclinical findings on GLP-1 agonists by conducting an early-Phase II investigation of semaglutide in non-treatment-seeking participants with AUD, with a focus on human laboratory outcomes. Given preclinical evidence for the potential efficacy of GLP-1 agonists for reducing both alcohol and nicotine intake, and the need for treatments targeting both alcohol and nicotine addiction, this proposal focuses on cigarette smokers with AUD.

This is a single-site, Phase II, two-arm, double-blind (participants, outcome assessors), randomized, placebo-controlled, dose-ranging study. Following a baseline assessment eligible participants will be randomized to medication or placebo conditions by the IDS staff at a 1:1 ratio. The treatment period will be approximately 10 weeks, taking into account the dose escalation necessary for this medication. Those randomized to semaglutide will receive 0.25mg/week for 4 weeks (Weeks 1-4), and 0.5mg/week for 4 weeks (Weeks 5-8). Provided that participants have tolerated the medication , participants will receive a final dose of 1.0mg (Week 9), and will return for a discharge lab visit with repeat lab tests at Week 10, followed by a final monitoring assessment by phone scheduled approximately 6 weeks after the last medication dose (Week 15). Those in the placebo condition will receive sham placebo administration at each visit (described in further detail in later sections). Participants will attend weekly visits at Eastowne CRU or CTRC to receive medication or placebo doses.

In addition to the medication visits, participants will be asked to attend four laboratory visits: two involving alcohol self-administration (one prior to the first medication visit and one during the 0.5mg treatment phase) and two involving alcohol challenge (one prior to the first medication visit and one during the 0.5mg treatment phase). These sessions are designed to assess changes in alcohol responses over the course of the treatment period. Laboratory sessions are described in further detail below. Measurements of naturalistic alcohol and cigarette use will also be recorded throughout the treatment period. These assessments will be captured using a daily diary that will be provided to participants. Participants will receive an automated text message daily via cell phone as a reminder to complete the diary. Additional self-report measures of substance use will be collected at study visits.

As summarized above, the primary objectives are to examine whether treatment with semaglutide alters alcohol self-administration (primary outcome) and laboratory measures of alcohol response and motivation (i.e., demand and responses to alcohol, secondary outcomes) during alcohol administration. We hypothesize that the semaglutide treatment (versus placebo) will be associated with reduced alcohol self-administration during a laboratory session conducted at the maintenance dose of 0.5mg.

We further hypothesize that semaglutide will lead to reductions in alcohol demand, cigarette demand, and subjective responses to alcohol (e.g., reduced stimulation) during alcohol administration sessions.

The primary and secondary outcomes for this trial will be assessed during exposure to the standard maintenance dose (.5mg). While an extended treatment phase at the 1.0mg dose is beyond the scope of this small-scale trial, the inclusion of a brief (1-week) phase at 1.0mg is designed to provide initial data on tolerability and any changes on selected secondary outcomes (i.e., drinks per day and cigarettes per day) in this population. Capturing these data will help to support feasibility and pilot data for subsequent funding proposals with longer treatment intervals.

Secondary objectives include examining changes in naturalistic alcohol consumption and cigarette smoking during the treatment period. Although participants will not be attempting to reduce drinking or smoking in this trial, it is hypothesized that treatment with semaglutide will result in reductions in alcohol consumption (drinks per day) and cigarettes per day over the course of the treatment period. Tertiary/exploratory outcomes include examining changes in body weight, HbA1c, and alcohol elimination during the trial.

In total, participation in this study is estimated to take a maximum of up to 50 hours of combined time over a period of up to approximately 16 weeks beginning with initial screening. This includes a screening and baseline visit (up to 4 hours), up to 9 subsequent laboratory visits for medication administration and side effects monitoring (up to 1 hour each), a final discharge visit (Week 10), and final telephone check-in (Week 15). If necessary for safety reasons, (e.g., based on lab results), participants may be asked to attend (unplanned) visit(s) for follow-up labs at the discretion of the study physician. Participants will also complete four visits involving alcohol administration over the course of Weeks 0-10; these sessions may last up to 7-8 hours each. Participants also will receive calls from staff 2-3 days after each medication phase begins (placebo, 0.25mg, 0.5mg, 1.0mg) to check in on side effects (10 minutes per call), and will be asked to complete brief daily questionnaires via a daily diary with reminders sent via daily text message prompt (1-2 minutes per day).

A detailed summary of study procedures is provided below.

Detailed Study Procedures.

Following initial recruitment (described in section 5.5), participants will be asked to complete the following procedures:

a) <u>Telephone screen</u>

Initial screening will be conducted by telephone by trained research staff and will assess participant alcohol, cigarette, and drug use, medications, and basic medical inclusion/exclusion factors. Potential participants will be given a brief description of the study. The initial telephone screen is estimated to take roughly 15-30 minutes. Contact information will be collected from interested callers who pass the initial screen. Following the initial screen, participants will proceed to be booked for an informed consent and baseline interview session. The informed consent procedures will be conducted remotely using a HIPAA-compliant virtual meeting platform (e.g., Webex, Zoom) and Docusign. Depending on participant and staff availability, the phone screening and informed consent/baseline interview can happen on the same day (after the screening call) or at a future date.

b) <u>Screening and Baseline Data Collection Visits</u>

At the informed consent session, a member of the research staff will review the study procedures and carry out informed consent procedures (including electronic signature via Docusign) prior to proceeding to the baseline interview. Following informed consent, the staff member will conduct a semi-structured interview to assess inclusion/exclusion criteria. A research assistant will use a structured interview to measure diagnostic and substance use screening criteria, including symptoms of alcohol use disorder; inclusion/exclusion factors related to medical history will also be assessed (see criteria listed above). The Structured Clinical Interview for DSM-V [53] will be used to assess AUD and substance use disorder symptoms. History of other psychiatric diagnoses and medication use will be assessed via self-report. The research assistant will also collect information on demographic factors, criteria related to current medical status and medications, current alcohol, tobacco and other substance use (see "Phone Screen" document), as well as substance use disorder symptoms (via select modules from the SCID interview) and symptoms other psychiatric symptoms (using the MINI screening module). Participants who appear eligible will be booked for an in-person visit, which will take place at the laboratory site (Medical Wings C), with some procedures (e.g., blood draw) booked at CTRC as necessary.

The in-person screening/baseline visit will include a brief review of informed consent to answer any additional questions, and a brief review of self-report and interview questions from the remote screening session. The Timeline Follow-Back (TLFB) [54] will be used to assess alcohol, cigarette and other drug use in the 90 days prior. The participant will also provide a breathalyzer screen. Participants who have a positive BAC (greater than 0.00) will be monitored until their BAC is reduced to a safe level (BAC<.03g%), in accordance with NIAAA guidelines (or to 0.00g% in the event that the participant drove to the session).

Participants who remain eligible after the interview will complete vitals and measurements (HR, BP, height, weight, temp), and a measure of expired alveolar carbon monoxide (CO). A baseline saliva sample for DNA analysis will also be collected. Additionally, participants will provide a urine sample and complete a blood draw (up to 50-60 ml) for a comprehensive metabolic panel (e.g., Na, K, Cl, CO2, BUN, Creatinine, Glucose, Calcium, Albumin, Total protein, Total bilirubin, AST, ALT, Alk Phos), baseline measure of HbA1c, baseline measure of nicotine metabolites (e.g., 3HC, COT), serum amylase and lipase, and complete blood count (e.g., Auto WBC, Corrected WBC, RBC, HGB, HCT, MCV, MCH, MCHC, RDW, MPV, Platelet). These procedures will be conducted by the research assistant, and the baseline blood draw will be taken by a team member with phlebotomy training. If there is no team member with phlebotomy training available that day, the blood draw may also be booked at CTRC. If needed, the blood draw and other baseline procedures may be booked as separate visits. On the day of the visit that includes the blood draw, participants will be instructed to arrive fasting for 12 hours (to allow for assessment of baseline HbA1c and lipids). If participants are not able to arrive fasting for the baseline session, the fasting lipids sample may be deferred or completed at the next possible visit. Blood samples will be sent to McClendon laboratory for testing. A portion of the blood sample will also be stored (Thurston Bowles building) for subsequent analysis (e.g., nicotine metabolites). The saliva sample will also be stored at Thurston Bowles building. The urine sample will be used to test for the presence of several drugs (for example, heroin, cocaine, methamphetamines, opiates). (For females, the test will include a pregnancy screen.) If a participant tests positive for drugs other than cannabis (or if females screen positive for pregnancy), the participant will be excluded from the study.

Participants will complete baseline questionnaires, which will be collected on paper. A Demographic Screening Questionnaire will assess basic demographic factors (e.g., age, education, income). A Locator form will collect contact information for three individuals who can be contacted if a participant is unable to be reached by study staff for follow-up visits. Other baseline measures will include the Alcohol Use Disorders Identification Test (AUDIT) [55], Self-Report of the Effects of Alcohol Questionnaire (SRE) [56, 57], Fagerström Test of Nicotine Dependence (FTND) [58], and the Nicotine Dependence Syndrome Scale (NDSS) [59], Barratt Impulsivity Scale (BIS-11) [60], UPPS Impulsive Behavior Scale (UPPS) [61], Impaired Control Scale (ICS) [62], Center for Epidemiological Studies Depression Scale (CES-D) [63], Toronto Alexithymia Scale (TAS-20) [64], the Reward Probability Index (RPI) and the Environmental Reward Observation Scale (EROS) [65]. Baseline measures that will be repeated at subsequent visits will include the Timeline Followback (TLFB), the Penn Alcohol Craving Scale (PACS) and Tiffany Questionnaire of Smoking Urges (TQSU) [66], and the Wisconsin Smoking Withdrawal Scale (WSWS) [67]. To assess potential medication effects on changes in motivation and self-efficacy for resisting alcohol/cigarettes, we will use alcohol and smoking Contemplation Ladder items, Alcohol and Cigarette Abstinence Self-Efficacy (AASE/CASE) (these scales will be repeated at each visit) and the Inventory of Drinking Situations (IDS) scales [68-70]. Appetite and eating behavior will be assessed with the Simplified Nutritional Appetite Questionnaire (SNAQ) [71], Control of Eating Questionnaire (CEQ) [72], the Three Factor Eating Questionnaire (TFEQ) [73] and the Reward-Based Eating Drive Scale (R-BEDS) [74]. During the medication phases, side effects will be assessed with the SAFTEE questionnaire [75], a standard tool for alcohol clinical trials. Participants will also complete previously validated Purchase Tasks for alcohol, cigarettes, and food, and delay discounting will be assessed with the Monetary Choice Questionnaire (MCQ) [76-79]. These brief behavioral economic tasks assess perceived value of reinforcers by presenting hypothetical scenarios involving choices between the reinforcer (alcohol, cigarette, food) and monetary rewards; these measures will be completed at each visit. Finally a previously validated sweet taste preference test will be administered at baseline and again on the final visit, since this phenotype has been shown to be implicated in both food and alcohol reward, and may be modulated by a GLP-1 agonist. This test involves participants tasting solutions of sugar water prepared to reach .05, .1, .2, .42, and .83 molar sucrose, and then rating preference and intensity of the taste. The sweet taste test will be overseen by a research assistant using previously published protocols from prior projects at UNC [80, 81].

The baseline questionnaires and interview assessments are projected to take approximately 3-4 hours combined. To accommodate potential scheduling/time constraints or delays at the baseline visit, some questionnaires that are not time-sensitive (for instance, personality questionnaires) may be deferred until a later lab visit as needed, at the discretion of the study team. All interview records and paper questionnaires will be stored in study binders in a secure location for subsequent entry into an electronic database for analysis. Patients will be given breaks as needed. Following the blood draw, participants will be offered a snack and bottled water (given the instructions to arrive fasting). If results of the baseline screening/medical assessment indicate that the participant is tentatively eligible, they will be given a comprehensive orientation to the next phases of study, including the follow-up medication visits, the laboratory alcohol administration visits, and the daily surveys. Locator/collateral contact information will also be obtained. A Locator form will collect contact information for three individuals who can be contacted if a participant is unable to be reached by study staff for follow-up visits. Following the completed baseline assessments, the participant’s screening summary will be reviewed for eligibility, including review of medical criteria and any

medications that the participant is taking by the study physician. Lab results will be uploaded to the EMR and reviewed by the study physician prior to prescribing the medication. Potential interactions with semaglutide will be verified using an established online database such as Epocrates or Lexicomp. In the event of any uncertainties about medication interactions, participants will be asked to contact their primary care physician to confirm no concerns with taking semaglutide.

For eligible participants, the study physician will provide sign-off on all medical criteria (e.g., medical history, medications, lab results), and the PI will provide sign-off on other baseline criteria. Once participants have been confirmed as eligible, the physician will submit an order to start medication or placebo. Medication and placebo will be dispensed at CTRC (Week 1 visit) at a date coordinated with the participant. Participants will be advised to be vigilant for symptoms of hypoglycemia at the medical visit, and instructed to contact the study team to review any symptoms that emerge (regular glucose tests will also be obtained at follow-up visits, as noted in the schedule of assessments). If necessary, the medication review will take place via telephone or telemedicine, depending on medical team availability.

Participants' Week 1 visit will be scheduled with enough lead time to review lab results and exclude participants (if needed) prior to administering the study medication. Additionally, if clarification of medical criteria is needed at any point during or following the phone or in-person screening process, or at any subsequent stage of the study, the study physician may meet with the participant via a remote contact method (e.g., phone, Webex/Zoom, or telehealth) to clarify medical questions or discuss test results.

c) Daily Assessments.

Throughout the study, participants will be asked to complete a brief self-report survey each day that includes measures of 1) prior-day number drinks consumed; 2) number of cigarettes smoked; and 3) prior day peak craving/demand for alcohol and cigarettes. Participants will be provided with a paper booklet in which to enter responses and will be asked to bring the booklet to each visit. At each visit the research staff will collect the interim daily surveys for retention in the participant's study binder. Participants will be texted an automated text message each day during their participation in the study as a reminder to complete the daily survey. If preferred, participants can opt to receive an email link. (No daily survey data will be collected from participants via text or email.) At each study visit, a staff member will provide compensation for the prior week's surveys, and will review survey adherence and progress towards compensation for good adherence (described later), as a means of motivating adherence to self-report assessments. In the event a participant completes the daily assessments but forgets to bring the packet to a given visit, they will be asked to bring their packet to the following session. Participants will be asked to either a) complete the measures in person at the current lab visit, or b) to schedule a brief meeting with a research team member via Zoom or Webex in order to complete the questions (depending on scheduling and availability). These approaches will be used in specific circumstances if needed (e.g., if the participants forgets to complete the daily assessments altogether, or will be unable to bring it to the next session).

d) Weekly Laboratory Visits.

Medication administration visits will occur at either the Eastowne Clinical Research Unit (CRU) or the CTRC. Following the baseline (Week 0) visit, participants will visit CRU or UNC Hospital/CTRC once weekly (Weeks 1-9), for subsequent medication or placebo doses, and for a final follow-

up/discharge visit at Week 10. (In addition, the study physician may request an additional follow-up lab visit(s) at any point in the study to monitor side effects.) In addition, to accommodate events such as missed lab visits or scheduling difficulties, assessment procedures at a given weekly visit (e.g., questionnaires, lab tests) may be conducted within a 7-day window of the original target date, as necessary, to avoid unnecessary missing data. In addition to medication/placebo administration, follow-up visits will include 1) vitals, weight measurements, and a brief side-effects assessment, 2) a finger-stick glucose test (or fasting blood draw depending on the week, see study flow chart), 3) a brief calendar-based self-report assessment of interim alcohol and cigarette use (TLFB), and 4) a repeat CO measurement. These procedures are expected to take roughly 30-60 minutes in total, and will be conducted by a trained study team member and/or a member of the CRU/CTRC team (e.g., RN). All side effects reports, labs, and other medical test results (e.g., glucose tests) will be uploaded in Epic (e.g., lab tests) or sent electronically (e.g., self-reported medication changes) for review by the study physician. If side effects or lab results suggest that further assessment is indicated, a member of the medical team will advise the study team (and if necessary, contact the participant) to provide direction. At the Week 4, Week 8, and final discharge visits, participants will be asked to arrive fasting to allow for a blood draw for follow-up lab assays (identical to those described in the baseline procedures above). As with the baseline sample, a portion of each sample will be stored for follow-up analyses (e.g., nicotine metabolites).

e) <u>Alcohol Sessions.</u>

To evaluate effects of medication phase on laboratory measures of alcohol response and self-administration, participants will be asked to complete four laboratory visits over the course of the study. Two visits will occur between the Week 0 and 1 visits (baseline phase), and two will occur between the Week 8 and 9 visits (0.5 mg phase). Minor scheduling deviations will be accommodated as needed, provided that the participant has completed at least three weeks of the .5mg phase at the time of the second set of laboratory sessions.

During each of these sessions participants will be asked to report to the lab having received instructions to abstain from alcohol and recreational drugs for 24h, and not to consume food for 4 hours prior to arrival. Participants will also be advised not to drive to these study appointments. Participants will also be asked to schedule a smoking break just prior to arrival (to ensure that participants are not nicotine-deprived during testing) [16]. Upon arrival participants will provide breathalyzer and expired CO readings, as well as measures of body weight and vitals (HR, BP) and a finger-stick blood glucose test. Participants will also receive the Clinical Institute Withdrawal Assessment for Alcohol (CIWA-AR) to ensure that no significant withdrawal symptoms are present. A CIWA score of 10 or greater will require a consultation with an addiction medicine physician (Dr. Tow or Dr. Jordan) prior to proceeding. (We except a low likelihood that this will happen because participants with severe AUD or history of withdrawal symptoms will be excluded). Female participants will also be asked to repeat the urine pregnancy screen at each alcohol session.

In the interim, participants will complete self-report questionnaires (e.g., Timeline Follow-Back to assess recent substance use). Participants will be seated in a comfortable recliner chair for the alcohol administration procedures. Participants will first complete baseline physiological readings (systolic and diastolic blood pressure, heart rate), and baseline measurements of self-report questionnaires (see below). Next, participants will complete one of two procedures (alcohol challenge, alcohol self-administration), dependent on the visit week. Specifically, the first alcohol challenge session (AC1) and the first self-administration session (SA1) will be scheduled to take place

between Week 0 and 1. The second alcohol challenge session (AC2) and the first self-administration session (SA2) will be scheduled to take place between Week 8 and 9 (0.5mg treatment phase), allowing for minor scheduling adjustments as noted above. Prior to the self-administration session only (which is longer in duration), participants will consume a standardized, isocaloric meal in advance of the alcohol self-administration procedures, with a standardized wait time (60 min) between completion of the meal and onset of beverage consumption. The two session types will be scheduled with at least one interim day scheduled between sessions when possible. For both sessions, participants will be instructed to expect to remain in the lab for a full day (e.g., 8 hours).

Alcohol Challenge Sessions (AC1, AC2)
The AC sessions are designed to generate data to estimate within-person changes in acute responses to alcohol as a function of medication phase (baseline vs. 0.5mg). Following baseline assessments (described above), participants will be asked to consume a pre-determined dose of alcohol calculated to achieve a target peak breath alcohol concentration (BrAC) of .06g%. This BrAC level reflects a moderate alcohol dose. Individualized doses will be calculated using established formulas [83] that account for determinants of total body water (e.g., height, weight, sex, age). Beverages will consist of 80-proof vodka combined with a non-alcoholic, sugar-free mixer at a 1:5 ratio. Beverages will be allocated to three equal portions in separate cups. Participants will be asked to consume the beverages at an even pace over 10 minutes. A separate placebo session is not incorporated for the AC sessions, because our primary interest is in examining *within-person* changes in laboratory alcohol responses as a function of medication dose (baseline vs. 0.5mg), and because our primary outcome is self-administration (described below). Although a limitation of this approach is the inability to dissociate expectancy from pharmacological effects in measuring subjective responses to alcohol, increasing the number of sessions to dissociate expectancy effects is not a major aim for this preliminary project.

After completion of the beverages, participants will wait 10 minutes (to allow an absorption period), then will complete an initial breath alcohol concentration (BrAC) reading. BrAC readings will be repeated at 10-minute intervals during the first hour of the session (peak BrAC is anticipated to occur around 30-40 minutes, on average, with variability across subjects). Participants will repeat the battery of self-report questionnaires (outlined below) and physiological assessments at three points on the ascending limb of the blood alcohol curve: upon the first BrAC reading that meets or surpasses .02g%, .04g%, .06g%. To account for the fact that some participants may not reach the .06g% target, the latter assessment will be conducted if/when it appears the participant will not reach the .06g% target (as evidenced by two consecutive descending BrAC readings after surpassing the .05g% mark).

Following completion of the last set of measures (.06g%), participants will then be escorted to a private room to begin the recovery phase, during which they will receive water and will be monitored, and take bathroom breaks as needed. During this time, BrAC measurements will continue every 15 minutes. On the descending limb of the blood alcohol concentration curve, participants will complete follow-up measures and physiological readings at the first BrAC readings that meet or fall below .04g% and .02g%. Prior to discharge, participants will be asked to provide an additional blood glucose sample (via fingerpick) to assess any differential changes in glucose levels following alcohol administration as a function of medication phase. Participants will also complete the Food Purchase Task to assess motivation for food following alcohol administration. After this point, participants will be provided with a lunch and snacks to consume during the recovery period.

Participants will be compensated and discharged once BrAC reading reaches .00g% (to allow for estimating alcohol elimination rate across sessions AC1 and AC2 as a function of medication group).

Subjective Measures. At baseline (prior to alcohol ingestion) and each subsequent assessment point noted above, participants will complete several measures. Measures of subjective alcohol effects will include the Biphasic Alcohol Effects Scale (BAES) [84], the Alcohol Urge Questionnaire (AUQ) [85] and Drug Effects Questionnaire (DEQ) [86]; the latter will be supplemented with additional items to assess potential effects related to medication (e.g., “tired”, “confused”, “nauseous”, “dizzy”, “sick”, “drunk/intoxicated”) [16]. The Alcohol Purchase Task [45] Cigarette Purchase Task [87] Food Purchase Task [78] and Monetary Choice Questionnaire [76]will be also administered at the corresponding intervals noted above. Changes in mood will be assessed using the Profile of Mood States – Short Form (POMS-SF) [88]. During the recovery period, participants will complete another battery (all measures noted above) once reaching .04g% and .02g% on the descending slope of the blood alcohol curve.

Alcohol Self-Administration Sessions (SA1, SA2).

Medication effects on laboratory alcohol consumption will be examined using a validated oral self-administration procedure, which has been established as sensitive to medication effects. Pre-session instructions and arrival procedures/tests will be identical to those noted above. After completing baseline physiological and baseline questionnaires, the procedure will commence with a *priming drink*, calculated to achieve a target breath alcohol concentration (BrAC) of .03g%. In this procedure, a priming drink is administered as a means of prompting subsequent self-administration. The priming drink will include 1 part 80-proof liquor (of the participant's choice) to 5 parts mixer, and will be consumed over 5 minutes, followed by a 20-minute absorption period. At the end of the absorption period participants will complete the second round of self-report questionnaires (see Measures), as well as physiological and BrAC readings.

After the post-priming assessments, participants will complete a 2-hour *ad libitum* self-administration period, separated into two 1-hour blocks. During each block, participants are allowed to consume the same alcohol beverage as they selected for the priming drink, with a total volume equivalent to up to 4 “mini-drinks”, each calculated (based on the person’s sex, weight, height, and age) to achieve an average additional BrAC increment of .015g% per drink using established formulas. Beverages will be presented to the participant at onset of each block, and the remaining amount will be removed and weighed at the conclusion of each block. Consistent with prior studies using this protocol, participants will have the choice of receiving monetary reinforcement (at the end of the session) for drinks not consumed ($3 per mini-drink beverage equivalent), which provides a competing incentive against consumption. Participants will repeat additional rounds of self-report assessments (described above) at 30-minute intervals during the *ad libitum* phase (i.e., at 60, 90, and 120 min. after the end of the priming phase), with physiological assessments and BrAC readings taken at these intervals. The primary outcomes for this session are peak BrAC and volume of alcohol consumed.

Participants will then be escorted to a private room for the recovery period. Assessments during the recovery period will be identical to those described above. Participants will be monitored until being discharged when BrAC declines below .03% for two consecutive readings, consistent with NIAAA recommended guidelines. [52] As noted, participants will be instructed not to drive, and will be asked to arrange for a ride home from the study site. If a participant cannot arrange a ride home, they will be provided with transportation home via taxi or Uber. If a participant reports having driven to a session, they will be required to remain at the site until BrAC is measured at 0mg% for two consecutive readings.

List of Study Assessment Measures (See Section 1.3 for assessment time points)

1. Demographics form
2. Timeline Follow-Back (TLFB)
3. Structured Clinical Interview for DSM-5 Mental Disorders (select modules)
4. MINI Neuropsychiatric Interview (select modules)
5. Sweet Taste Preference Test
6. Alcohol Use Disorder Identification Test (AUDIT)
7. Self-Report of the Effects of Alcohol Questionnaire (SRE)
8. Center for Epidemiological Studies Depression Scale (CES-D)
9. Toronto Alexithymia Scale (TAS-20)
10. Barratt Impulsivity Scale (BIS-11)
11. UPPS Impulsive Behavior Scale (UPPS)
12. Reward Probability Index – Environmental Reward Observation Scale (RPI-EROS)
13. Impaired Control Scale (ICS)
14. Fagerstrom Test for Nicotine Dependence (FTND)
15. Nicotine Dependence Syndrome Scale (NDSS)
16. Penn Alcohol Craving Scale (PACS)
17. Tiffany Questionnaire of Smoking Urges (TQSU)
18. Wisconsin Smoking Withdrawal Scale (WSWS)
19. Wisconsin Inventory of Smoking Dependence Motives (WISDM)
20. Alcohol and Smoking Contemplation Ladder
21. Three Factor Eating Questionnaire (TFEQ)
22. Reward-Based Eating Drive Scale (R-BEDS)
23. Control of Eating Questionnaire (CEQ)
24. Simplified Nutritional Appetite Questionnaire (SNAQ)
25. Monetary Choice Questionnaire (MCQ)
26. Cigarette Abstinence Self-Efficacy (CASE)
27. Alcohol Abstinence Self-Efficacy (AASE)
28. Inventory of Drinking Situations (IDS)
29. Biphasic Alcohol Effects Scale (BAES)
30. Alcohol Urge Questionnaire (AUQ)
31. Drug Effects Questionnaire (DEQ)
32. Alcohol Purchase Task (APT)
33. Cigarette Purchase Task (CPT)
34. Food Purchase Task (FPT)
35. Profile of Mood States – Short Form (POMS-SF)
36. Systematic Assessment for Treatment Emergent Effects (SAFTEE)

Analysis schedule. Analyses of side effect frequency and AE/SAE frequency will be conducted prior to scheduled DSMB meetings. Other analyses will be conducted following the completion of the trial, or as otherwise required to fulfill DSMB requests or other reporting requirements.

4.2 SCIENTIFIC RATIONALE FOR STUDY DESIGN

This short-term trial is intended to produce initial data on the effects of semaglutide in non-treatment-seeking participants with AUD, with an emphasis on human laboratory outcomes. The dose escalation schedule is based on the standard clinical regimen, which is intended to minimize side effects common

to GLP-1 agonists (i.e., nausea). Participants in the placebo group will complete an identical sequence while receiving sham subcutaneous injections (a small needle stick using a micro-needle of the same size, without an injection). This placebo condition is selected due to the absence of an available matching placebo cartridge from the manufacturer (see section 4.3). All participants will complete laboratory alcohol administration sessions at two time points, corresponding with the baseline and 0.5mg phases. This design allow for within-subjects analyses for laboratory outcomes that are best examined at the within-subject level (owing to high inter-individual variability in alcohol pharmacokinetics). Overall, this design is intended to maximize feasibility and sample size while also accommodating the extended dosage titration period required of GLP-1 agonists. Findings from this trial will be used to justify a larger, placebo-controlled Phase II clinical trial application via the R01 mechanism.

4.3 JUSTIFICATION FOR DOSE

Semaglutide (Ozempic®) will be administered according to the FDA-approved schedule, which begins with a starting dose of 0.25mg (for titration purposes) once per week for four weeks. At week five, dosage is increased to 0.5mg/week (the initial first maintenance dose) for a subsequent four weeks, at which point dosage can be increased further (1.0mg) if indicated (and presuming that the 0.5mg dose has been tolerated). The intent for this initial trial is to mimic the typical clinical scenario over the first several weeks of medication titration. Although primary analyses will focus on the 0.5mg dosage, participants will receive one week of 1.0mg treatment at the conclusion of the 0.5mg phase, in order to collect initial data on side effects and naturalistic drinking/cigarette use at this dosage in the current population. Participants will be monitored carefully and will not be escalated to the next dose if experiencing persistent side effects indicative of poor tolerability.

Given the lack of a true placebo (i.e., no manufacturer-developed placebo Ozempic® pen/cartridge), this study will use a placebo condition that consists of weekly sham doses. Sham doses will involve a brief needle prick using a very small (e.g., 32 gauge) needle. These procedures are described in more detail later in this protocol. By necessity, medical staff administering the medication cannot be blind to condition. However, other experimenters and outcomes assessors will remain blind to condition. Participants will be instructed during the informed consent and study orientation that they have a 50/50 chance of being randomized to medication and placebo groups.

4.4 END OF STUDY DEFINITION

A participant is considered to have completed the study if he or she has completed all phases of the study including the last visit shown in the Schedule of Activities (SoA), Section 1.3. The end of the study is defined as completion of the last visit shown in the SoA in the trial overall.

5 STUDY POPULATION

5.1 INCLUSION CRITERIA

In order to be eligible to participate in this study, an individual must meet the following criteria:

- Age 21-65

- Meeting DSM-5 criteria for current (past year) mild or moderate AUD (i.e., from 2-5 symptoms endorsed), and NIAAA criteria for current at-risk drinking (i.e., >7/14 drinks in one week for women/men, with at least two episodes of 4+/5+ drinks in the past 30 days)
- Daily smoker, defined as reporting smoking 1+ cigarettes per day, on average, over the past 12 months (past year avg cigarettes per day ≥ 1) and daily/near-daily smoking in the past month (smoking at least 25 days in the past 30)
- Willingness/availability to take study medication and complete study procedures, including attending weekly visits for medication administration, side effect assessments, and glucose monitoring
- Willingness to complete laboratory sessions involving alcohol administration
- Ability to communicate and read in English

5.2 EXCLUSION CRITERIA

- Regular use of electronic nicotine delivery systems (ENDs; vaping), cigars, chewing tobacco or snuff, based on at least weekly use in the past 30 days
- Past 30-day use of nicotine replacement therapies/products
- Current engagement in alcohol or smoking cessation treatments, or currently engaged in intentional efforts to quit alcohol use
- Past 30-day use of: Sincalide, Sulfonylureas, insulin and insulin products or other medications that may interact with semaglutide;, or weight control medications
- Prior use of semaglutide or other GLP-1 agonists
- Known or suspected hypersensitivity to study medication or related products
- Lifetime diagnosis of severe mental illness (including schizophrenia and bipolar disorder)
- History of suicide attempt, or recent (past 30 day) suicidal ideation, or psychiatric hospitalization in the last 6 months
- Current significant medical or neurological illness (based on self-report or medical record) including severe hepatic impairment or cirrhosis, impaired renal function (eGFR <50ml/min), acute or chronic pancreatitis, gastroparesis, gallbladder disease or cholelithiasis, other severe gastrointestinal disease, heart failure, coronary artery disease, stroke, seizure disorder, or other medical condition that poses a risk for the medication or alcohol administration components of the study (as determined by the MD)
- A personal or family history of medullary thyroid cancer or multiple endocrine neoplasia 2A or 2B
- Calcitonin greater than or equal to 50 ng/L
- Uncontrolled thyroid disease TSH >6.0 mIU/L or <0.4 mIU/L at screening
- History of major surgical procedures involving the stomach potentially affecting absorption of trial product (e.g., subtotal and total gastrectomy, sleeve gastrectomy, gastric bypass surgery)
- History of Type 1 or Type 2 diabetes, or HbA1c >6.5% measured at screening
- History of diabetic retinopathy, proliferative retinopathy, or maculopathy
- History of diabetic ketoacidosis

- History or presence of malignant neoplasms within the last 5 years (except basal and squamous cell skin cancer and carcinoma in situ)
- Currently nursing, pregnant, anticipating pregnancy in the next 6 months, or not using a highly effective contraceptive method as judged by the MD, and defined as:
    a. combined (estrogen and progestogen containing) hormonal contraception associated with inhibition of ovulation (oral, intravaginal, transdermal)
    b. progestogen-only hormonal contraception associated with inhibition of ovulation (oral, injectable, implantable)
    c. intrauterine device
    d. intrauterine hormone-releasing system
    e. bilateral tubal occlusion
    f. vasectomized partner
    g. sexual abstinence
- Elevation of serum lipase, amylase, direct (conjugated) bilirubin, or alkaline phosphatase (ALP), ALT, or AST) more than 3X the upper limit of normal on baseline bloodwork
- Baseline body mass index (BMI) <25kg/m2 or >35kg/m2
- Uncontrolled hypertension or systolic BP >180 mmHg and/or diastolic BP >105 mmHg, averaged from three measurements
- Plans for travel outside of the local area in the upcoming 12 weeks that would interfere with lab visits during the study period (or other logistic factors that would make it difficult to commit to entire duration of study)

5.3 LIFESTYLE CONSIDERATIONS

Specific lifestyle instructions will include:

- Refrain from alcohol consumption or other drug use for 24 hours prior to alcohol administration sessions
- Arrive fasting at sessions scheduled for blood draws as needed
- Refrain from consuming food for 4 hours prior to alcohol sessions. Participants will receive a standardized snack on arrival for alcohol sessions.)
- Refrain from driving to the lab on the day of alcohol sessions. (Participants will be asked to arrange for transportation, or will be provided with transportation home via taxi or Uber)
- Refrain from operating other vehicles or machinery for the duration of day upon discharge from alcohol sessions
- Women participating in the study must report being willing to use a reliable birth control method during the trial (if sexually active), and must report no plans for pregnancy in the next 6 months.
- Participants will also understand that they may be excluded if they test positive for drugs other than cannabis during the time of scheduled drug screens (repeat drug tests will take place during weeks 4 and 8).

5.4 SCREEN FAILURES

Screen failures are defined as participants who consent to participate in the clinical trial but are not subsequently randomly assigned to the study intervention or entered in the study. A minimal set of screen failure information is required to ensure transparent reporting of screen failure participants, to meet the Consolidated Standards of Reporting Trials (CONSORT) publishing requirements and to respond to queries from regulatory authorities. Minimal information includes demography, screen failure details, eligibility criteria, and any serious adverse event (SAE).

Examples of screen failures would include participants excluded following baseline laboratory work, or those excluded based on a change in treatment-seeking status at the time of the baseline assessment. Individuals who do not meet the criteria for participation in this trial (screen failure) because of a specific modifiable factor (e.g., upcoming travel) may be rescreened at a later date. Rescreened participants will be assigned the same participant number as for the initial screening. Data pertaining to screening failures will be tabulated in an anonymized fashion.

5.5 STRATEGIES FOR RECRUITMENT AND RETENTION

Participants will be recruited from the community through advertisements, which may include ads in local online or print newspapers, print flyers placed in community or hospital settings, business cards, campus listservs, and online postings (e.g. Craigslist (ads will not be placed in employment section)), and social media ads (Facebook, Twitter, Instagram) that direct potential participants toward a study website/landing page (hosted on Research for Me). Participants may also be recruited from local medical clinics (e.g., primary care clinics) that grant approval for assisting with recruitment (e.g., by placing study advertisements in clinics). Flyers and online advertisements will include study contact information and a web link and/or QR code leading to the landing page.

In addition to these “passive” methods of recruitment, we will also include “active” recruitment methods. These methods will include members of the study team setting up recruitment booths and passing out business cards at public events (e.g., festivals) or public venues. Once initial contact with participants is made (e.g., via email or in person), research staff will schedule the participant for a phone call to explain the study and conduct a screening interview.

Participants will receive compensation. The payments will be allocated as follows (please see 1.2 for a summary of all scheduled sessions):

- $50 for completing the consent/screening/baseline sequence (Week 0)
- $75 for alcohol challenge session 1 (To be scheduled between Weeks 0-1)
- $75 for alcohol self-administration session 1 (To be scheduled between Weeks 0-1)
- $100 for alcohol challenge session 2 (To be scheduled between Weeks 9-10)
- $100 for alcohol self-administration session 2 (To be scheduled between Weeks 9-10)
- $30 per visit for each follow-up/medication administration visit during Weeks 1-10 (10 visits x$30 per visit = $300)

- Up to $150 for completing and returning daily questionnaires over the study period ($15 per weekly set of surveys x 10 weekly visits), which includes the week following the 1.0mg dose, to be paid at weekly medication visits)
- A $50 bonus for completing at least 9 of 10 weekly assessments
- Participants will receive a final bonus of $100 for completing the study through Week 10 (to be paid at the final discharge visit).

Therefore, the total maximum compensation over 10 weeks for study visits is $1000. Compensation for each pair of the alcohol sessions will occur at the end of the second of the session pair. The final payment (including bonuses) will be paid at the Week 10 discharge visit. Overall, these payments are structured to maximize retention in the study (given the long dose escalation period and time-intensive laboratory sessions). Additionally, an incentive-based structure that includes weekly incentives is important to reinforce engagement with daily surveys, and to minimize missing data. Participants will be updated at each visit with their progress toward the bonus incentives for adherence (e.g., weekly bonus of $10 and progress toward final adherence bonuses). In the event participants are not able to pick their final payments in person, we will offer the option of issuing the final payment via electronic gift card to the participant's email address, with their permission.

We anticipate phone screening up to 400 individuals and randomizing up to 48 participants to medication or placebo conditions, in order to obtain a final sample of at least 36 eligible participants who complete the full course of the 0.5mg phase (allowing for 25% attrition). If attrition is higher than anticipated, we will submit an amendment to enroll additional participants to arrive at a final sample of at least 36 participants who complete study procedures through the end of the 0.5mg dosage phase (i.e., through the end of Week 9).

6 STUDY INTERVENTION

6.1 STUDY INTERVENTION(S) ADMINISTRATION

6.1.1 STUDY INTERVENTION DESCRIPTION

Semaglutide is a relatively new (approved in 2017) GLP-1 receptor agonist. A new oral formulation of semaglutide was approved by the FDA in 2019, making semaglutide the first drug in its class to be available in both subcutaneous and oral formulations. GLP-1 agonist medications were designed to mimic the function of the endogenous GLP-1 peptide, with the benefit of a much longer half-life than endogenous GLP-1. Based on their glucose-lowering and appetite-reducing properties, GLP-1 agonists were developed as anti-diabetic therapies. Several GLP-1 agonists (e.g., exenatide, liraglutide, lixisenatide, albiglutide, dulaglutide and semaglutide,) are now in widespread use as treatments for type II diabetes (T2D); these medications have also proven efficacious for weight loss, reducing glycated hemoglobin (HbA1c), and reducing risk for some cardiovascular events [29]. Although the primary indication of GLP-1 agonists is T2D, these therapies are increasingly being studied for other indications in non-diabetic patients, including obesity/weight management. In June 2021, the FDA approved semaglutide at a higher dosage (2.4mg/week) for the indication of weight management, under the brand name Wegovy.

6.1.2 DOSING AND ADMINISTRATION

Semaglutide will be administered according to the FDA-approved schedule, which begins with a starting dose of 0.25mg once per week for four weeks. At week five, dosage is increased to 0.5mg for a subsequent four weeks (initial maintenance dose), after which point dosage can be increased further (to 1.0mg), presuming the medication is well tolerated.

Although semaglutide can be self-administered in the home setting, medication and placebo administration will be delivered by a research nurse or other medical team member at Eastowne CRU or CTRC in order to execute the placebo manipulation and ensure adherence to medication. This approach also allows us to collect safety outcomes on a regular basis, further ensuring that participants will be monitored carefully. Semaglutide is administered subcutaneously (in the abdomen, thigh, or upper arm). Doses are delivered via the Ozempic® pen, a cartridge-based device that calibrates medication delivery based on the desired dose. An extremely small needle (4mm, 32-gauge needle – the size of 2 human hairs) is used to deposit the medication subcutaneously. Because each pen contains 2mg of medication, each participant in the medication group will be allocated one pen to cover the first six weeks (i.e., 0.25mg/week for 4 weeks, followed by 0.5mg/week for 2 weeks), and a second pen for that will supply medication for the remaining three weeks (2 weeks at 0.5mg, and 1 week at 1.0mg). This schedule results in 9 medication administration sessions. This timeframe also allows sufficient time to reach steady-state plasma concentration levels (achieved within 4-5 weeks of initiating the starting dose).

At the present time, we do not have access to a manufacturer-developed placebo (the medication relies on the proprietary Ozempic® pen for delivery). Therefore, this study will use weekly sham injections for the placebo group. Sham doses will involve a brief needle prick using a very small (e.g., 32 gauge) needle, identical to the one used in the Ozempic® pen (NovoFine), or a needle of similar gauge (e.g., an insulin needle). Given the extremely small volume of medication delivered in the treatment condition, we will not utilize saline or another inert substance for the placebo administration. During medication and placebo administration, participants' view will be shielded from the injection site to prevent unblinding. Medication will be delivered in the upper arm, abdomen, or thigh. Given the extremely small volume of the injections, we will not utilize saline or another inert substance for the placebo administration; rather, the sham doses will consist of briefly inserting the micro-needle.

Due to the nature of the placebo condition, it will not be possible to blind the staff RNs administering the medication. However, we will provide training to study staff to minimize the likelihood of contaminating the blinding procedures. We will also develop procedures to minimize contact between the RNs and outcome assessors. All other staff members (including research assistants and outcomes assessors) will remain blind to condition, unless unblinding is necessary (e.g., due to an adverse event).

6.2 PREPARATION/HANDLING/STORAGE/ACCOUNTABILITY

6.2.1 ACQUISITION AND ACCOUNTABILITY

The team will collaborate with IDS to source the medication (and NovoFine or insulin needles for the placebo condition), which will be stored at the IDS pharmacy. Participants assigned to medication will be prescribed one Ozempic® pen following randomization. Pens will be stored by IDS and not shared across participants. A refill pen will be prescribed prior to the Week 7 visit, once the doses from the first pen have been allocated. Any unused doses from individual pens (e.g., in the event of participant drop-out) will be discarded/destroyed by IDS, as will any pens that expire during the course of the trial. In the event a pen expires before a participant completes the study, a replacement pen will be prescribed at the discretion of the study physicians.

6.2.2 FORMULATION, APPEARANCE, PACKAGING, AND LABELING

Ozempic® is manufactured by Novo Nordisk. As described above, the medication is dispensed from a proprietary device, the Ozempic® pen. Each pen contains 2mg of medication, requiring that each participant is prescribed two pens over the course of the study. A full description and visuals can be found at the following site:
https://www.Ozempic®pro.com/how-to-prescribe/Ozempic®-pen.html

The *placebo group* will receive sham injections that involve a brief needle-prick without dispensing a substance. The needles used for the sham injections will be the NovoFine 32G needle, the same needle in the Ozempic® pen (https://www.novoneedles.com/novofine.html) or a comparable needle of a similar gauge (e.g., an insulin needle). In no case will any fluid be injected during placebo sessions. To mimic the active medication administration, the medical staff member will insert the microneedle briefly and then remove it while the participant's view is shielded.

6.2.3 PRODUCT STORAGE AND STABILITY

Pens will be dispensed from IDS. For visits at CRU (Eastowne), pens will be delivered the day of the visit by courier. Ozempic pens will be stored at CRU in between visits. According to the manufacturer, following the first use, the Ozempic® pen can be stored for up to 56 days at room temperature (59 to 86 F) or refrigerated at 36 to 46 F. The IDS team will oversee the appropriate and secure storage of the drug. Each participant's pen will be labeled with the study ID number and pens will not be shared across participants. Placebo needles will remain packaged until use, and will be stored at room temperature in a secure location.

6.2.4 PREPARATION

Semaglutide does not require advance preparation; cartridges are pre-filled and doses are calibrated using the pen device. The RN or medical staff member will select the correct dosage and administer the medication as per manufacturer guidelines.

6.3 MEASURES TO MINIMIZE BIAS: RANDOMIZATION AND BLINDING

Following the baseline assessment, participants will be randomized to medication or placebo conditions at a 1:1 ratio by a member of the IDS team using a pre-determined randomization scheme. Medication and placebo administration will be carried out by a staff RN at CRU or CTRC. Due to the nature of the placebo condition, it will not be possible to blind the RNs to condition. However, all other staff members will remain blind to condition, and the staff members administering medication will be trained in protecting the blind (e.g., redirecting the conversation if the participant comments about medication condition; minimizing contact with experimenters.) To maintain blinding of participants, participants' view will be shielded and they will be asked to face away from the injection site during all placebo and medication administrations. Aside from the staff members administering medication, no study team members interacting with participants will be aware of participants' assignments. At the stage of interim analyses (e.g., in preparing safety reports for DSMB meetings) and final analyses, the study statistician will be provided with the assignment log. None of the study personnel involved in recruitment or

enrollment will have knowledge of the details of the randomization table that will be used by the IDS pharmacy.

At the completion of the trial, participants will be provided with a questionnaire asking about perceived medication assignment (to assess the success of maintaining the blind). Due to common side effects of semaglutide (in particular nausea), we do not expect blinding to be successful in all cases.

6.4 STUDY INTERVENTION COMPLIANCE

Medication will be administered by CRU or CTRC staff to assure compliance and reduce potential sources of non-adherence. A missed session will constitute a missed medication dose. As per manufacturer directions, any missed doses/appointments will be corrected by asking the participant to come in for the dose as soon as possible within 5 days of the missed dose. After the 5-day mark has passed, the dose will be skipped, and the participant will resume with the next scheduled dose. Study medication logs will serve as the primary source document for tracking medication and placebo administration. Participants will be coded as non-adherent if they miss more than 2 scheduled doses between Weeks 1-9. In the event of missed medication doses or missed appointments, the physician will have the discretion to adjust the appointment date (within the manufacturer or standard practice guidelines), or to prescribe a replacement pen if needed to accommodate the target medication schedule.

6.5 CONCOMITANT THERAPY

Participants may be taking medications for other conditions (provided those treatments are not included in the list of exclusion criteria). For this protocol, a prescription medication is defined as a medication that can be prescribed only by a properly authorized/licensed clinician. Medications to be reported in the Case Report Form (CRF) are concomitant prescription medications, over-the-counter medications and supplements. Changes to medication status or other treatments will be assessed at each in-person visit, with any changes reviewed by a study physician. Should a concomitant therapy preclude further participation for safety or scientific reasons (for instance, if a participant starts a therapy that constitutes an exclusion factor), this issue will be discussed by the study leads and the participant will be excused from further participation, if required to ensure safety. By definition, participants will not be seeking treatment for alcohol use or smoking cessation. If a participant makes the decision to seek treatment for alcohol or smoking during the study, s/he will be referred to appropriate treatment resources and excused from further participation.

6.5.1 RESCUE MEDICINE

Not applicable

7 STUDY INTERVENTION DISCONTINUATION AND PARTICIPANT DISCONTINUATION/WITHDRAWAL

7.1 DISCONTINUATION OF STUDY INTERVENTION

Study discontinuation: The study may be halted if deemed necessary by the lead investigators, DSMB, or IRB. Examples of reasons for study discontinuation include unexpectedly high frequency of serious adverse events, or significant increases in alcohol use during treatment, leading to the conclusion that the medication is not safe in this population. In addition to the participant discontinuation rules noted below, the following study stopping rules will trigger halting the study pending a DSMB evaluation: 1) Occurrence of any hospitalization or any SAE rated at Grade 4-5 (CTCAE) in a medication-treated participant (and deemed likely to be medication-attributable); 2) Documentation of SAEs in >3 medication-treated participants that are deemed likely attributable to medication; 3) Removal of >5 subjects due to adverse events deemed attributable to study-related procedures, inclusive of SAE and/or medical stopping rules (e.g., weight, alcohol consumption, disease progression), as defined in 7.2 below. In order to classify events as medication-attributable, the PI will request that IDS breaks the blind and provides a file containing condition assignment to the study physician(s). If any of the aforementioned events trigger a DSMB meeting, the full randomization table will be provided to a statistician, so that group differences can be fully evaluated at the meeting. Given the safety profile of semaglutide for other clinical conditions, as well as preclinical evidence that GLP-1 receptor agonists reduce drug intake, these scenarios are expected to be unlikely.

7.2 PARTICIPANT DISCONTINUATION/WITHDRAWAL FROM THE STUDY

Participants are free to withdraw from participation in the study at any time upon request. The investigators may discontinue or withdraw a participant from the study if clinically or otherwise indicated. Examples of reasons removing a participant include:

- Significant study intervention non-compliance
- Any clinical adverse event (AE), laboratory abnormality, or other medical condition or situation that indicates that continued participation in the study would be unsafe
- Pregnancy
- Diagnosis of significant disease or disease progression which requires discontinuation of the study intervention
- The participant meets an exclusion criterion (either newly developed or not previously recognized) that precludes further study participation
- Participant relocates from the local area, or is otherwise unable to attend visits
- Significant or persistent side effects that pose significant discomfort or preclude escalation to the 0.5mg dose (e.g., persistent significant nausea, or hypoglycemia, as indicated by repeated fasting glucose values below normal 54 mg/dL
- Changes in lab tests or clinical status from baseline that render the participant ineligible to continue in the judgement of the physician(s) (e.g., elevated liver enzymes, elevated serum amylase or lipase levels, weight loss as defined below)
- Excessive weight loss (defined as loss exceeding 5% of baseline weight or BMI < 20)
- A change in treatment-seeking status (i.e., the participant expresses a desire to enter treatment for alcohol or smoking cessation).
- A significant increase in alcohol consumption, as evidenced by a >50% increase in drinks per week (relative to baseline) lasting more than two weeks. Drinks per week in the 30 days preceding the Week 0 visit (measured by the TLFB) will serve as the baseline measure.
- Evidence of significant alcohol withdrawal symptoms (based on CIWA)

- Any other change in medical status or side effects that is significant enough to warrant removal in the judgement of the study physicians

If a participant exhibits changes in symptoms or clinical status that warrant potential removal, the study physicians will confer and render a decision as to whether to withdraw the participant. With respect to dosage increase, if a participant's side effects profile or laboratory tests during the initial 4-week phase (.25mg) indicate that s/he is unable to safely proceed to the 0.5mg dose, the participant may be removed from the study at the discretion of the physicians. If lab tests or side effects during the standard maintenance dose of 0.5mg suggest that a participant is unlikely to tolerate a higher dose (1mg), they may be excluded from the 1.0mg dose escalation at the discretion of the physicians. In this case, participants will be given the option of one additional week at the standard therapeutic dose of 0.5mg (i.e., highest tolerated dose). Reasons for participant discontinuation or withdrawal from the study will be recorded on the Case Report Form (CRF). Participants who sign the informed consent form but do not receive the study intervention (e.g., fail to attend the Week 1 visit for randomization) may be replaced. Participants who sign the informed consent form, and are randomized and receive the study intervention, and subsequently withdraw, or are withdrawn or discontinued from the study, may be replaced.

7.3 LOST TO FOLLOW-UP

A participant will be considered lost to follow-up if he or she fails to return for 2 consecutive scheduled medication visits and is unable to confirm attendance at the following visit (or is unable to be contacted by the study staff

The following actions will be taken if a participant fails to return to a required study visit:

- The team will attempt to contact the participant and reschedule the missed visit within one week, and counsel the participant on the importance of maintaining the assigned visit schedule and ascertain if the participant wishes to and/or should continue in the study.
- The team will attempt to contact the individuals that the participant had listed on the Locator Form at baseline (maximum of three attempted contacts per individual).
- Before a participant is deemed lost to follow-up, the investigator or designee will make every effort to regain contact with the participant (where possible, up to 3 telephone messages, up to 3 email messages, and if necessary, a letter to the participant's last known mailing address or local equivalent methods). These contact attempts will be documented in the participant's medical record or study file.
- Should the participant continue to be unreachable, he or she will be considered to have withdrawn from the study with a primary reason of lost to follow-up.

8 STUDY ASSESSMENTS AND PROCEDURES

8.1 EFFICACY ASSESSMENTS

The efficacy assessments/outcomes are noted in summary form in section 3. The ascertainment of these outcomes is described in further detail in section 4 (study design). Briefly, the endpoints are human laboratory measures of alcohol consumption and alcohol response; self-reported alcohol consumption and cigarette use over the course of the trial; and changes in weight, HbA1c, and alcohol elimination.

8.2 SAFETY AND OTHER ASSESSMENTS

Safety and assessments are summarized in detail in the study design section (Section 4.1). Briefly, safety assessments include a baseline assessment, body weight, bloodwork, vitals signs, and clinical interview to assess substance use and psychiatric symptoms. Follow-up medical visits will include repeat assessments of bloodwork and scheduled time points, as well as follow-up measurements of body weight and vitals signs. Medication side effects will be assessed at each visit, and a study team member will contact participants within 2-3 days of each dosage increase to evaluate potential side effects. Other assessments (questionnaires, interviews) are noted in Section 4.1.

Safety precautions during alcohol administration sessions. Our team has considerable experience with alcohol administration procedures. Our procedures follow the NIAAA guidelines for administering alcohol in human studies (https://www.niaaa.nih.gov/research/guidelines-and-resources/administering-alcohol-human-studies). Based on these guidelines, alcohol administration studies with participants who meet criteria for AUD are typically restricted to non-treatment-seeking participants in order to avoid ethical concerns, as will be the approach in this study. Additionally, our approach in this study is to only recruit people who meet criteria for mild or moderate AUD (i.e., endorsing 2, 3, 4 or 5 symptoms out of 11). Regarding the alcohol dose for alcohol challenge sessions, we will use sex- and body weight adjusted formulas [70] to ensure that dosage is calibrated across participants to achieve the target BrAC (.06g%). Participants will have undergone a medical screen prior to participation, and will be excluded if the study medical staff has reason to believe that alcohol is contraindicated. Participants will receive a BrAC reading on arrival to the alcohol sessions; the session will be rescheduled if the participant presents with a positive BrAC.

Following the completion of alcohol sessions, participants will be escorted to a private room designated for recovery purposes. During alcohol self-administration sessions, participants will only be discharged after their breath alcohol concentration descends below .03g%, which is consistent with NIAAA guidelines. During alcohol challenge sessions, participants will be asked to remain in the lab until BrAC reaches 0mg% (in order to collect BrAC data until alcohol is fully eliminated, for purposes of pharmacokinetic analyses). Participants will also be provided with reimbursement for transportation if they do not drive and are not able to locate assistance with transportation (participants will be advised not to drive to the alcohol sessions). If a participant presents with a positive BrAC, the session will be rescheduled, and the participant will be asked to remain in the lab until it is safe to leave.

To ensure participant safety, at least two members of the study staff will be present for the duration of all alcohol administration sessions. If an urgent medical situation arises at either medication or alcohol visits, a team member will contact the physicians on the team to consult, based on a predetermined sequence (e.g., contact physician A, followed by physician B, then physician C). Additionally, any participants who express a desire to seek treatment during the study will be excused from further participation and provided with alcohol or smoking cessation resources. Co-I Dr. Jordan directs outpatient addiction treatment at UNC-CH.

8.3 ADVERSE EVENTS AND SERIOUS ADVERSE EVENTS

8.3.1 DEFINITION OF ADVERSE EVENTS (AE)

Adverse event means any untoward medical occurrence associated with the use of an intervention in humans, whether or not considered intervention-related (21 CFR 312.32 (a)).

8.3.2 DEFINITION OF SERIOUS ADVERSE EVENTS (SAE)

An adverse event (AE) or suspected adverse reaction is considered "serious" if, in the view of either the investigator or sponsor, it results in any of the following outcomes: death, a life-threatening adverse event, inpatient hospitalization or prolongation of existing hospitalization, a persistent or significant incapacity or substantial disruption of the ability to conduct normal life functions, or a congenital anomaly/birth defect. Important medical events that may not result in death, be life-threatening, or require hospitalization may be considered serious when, based upon appropriate medical judgment, they may jeopardize the participant and may require medical or surgical intervention to prevent one of the outcomes listed in this definition. Examples of such medical events include an adverse reaction requiring intensive treatment in an emergency room or at home.

8.3.3 CLASSIFICATION OF AN ADVERSE EVENT

8.3.3.1 SEVERITY OF EVENT

For adverse events (AEs) not included in the protocol defined grading system, the following guidelines will be used to describe severity.

- **Mild** – Events require minimal or no treatment and do not interfere with the participant’s daily activities.
- **Moderate** – Events result in a low level of inconvenience or concern with the therapeutic measures. Moderate events may cause some interference with functioning.
- **Severe** – Events interrupt a participant’s usual daily activity and may require systemic drug therapy or other treatment. Severe events are usually potentially life-threatening or incapacitating. Of note, the term “severe” does not necessarily equate to “serious”.

8.3.3.2 RELATIONSHIP TO STUDY INTERVENTION

All adverse events (AEs) must have their relationship to study intervention assessed by the clinician who examines and evaluates the participant based on temporal relationship and his/her clinical judgment. The degree of certainty about causality will be graded using the categories below. In a clinical trial, the study product must always be suspect.

- **Related** – The AE is known to occur with the study intervention, there is a reasonable possibility that the study intervention caused the AE, or there is a temporal relationship between the study intervention and event. Reasonable possibility means that there is evidence to suggest a causal relationship between the study intervention and the AE.
- **Not Related** – There is not a reasonable possibility that the administration of the study intervention caused the event, there is no temporal relationship between the study intervention and event onset, or an alternate etiology has been established.

8.3.3.3 EXPECTEDNESS

The study physicians will be responsible for determining whether an adverse event (AE) is expected or unexpected. An AE will be considered unexpected if the nature, severity, or frequency of the event is not consistent with the risk information previously described for the study intervention.

8.3.4 TIME PERIOD AND FREQUENCY FOR EVENT ASSESSMENT AND FOLLOW-UP

The occurrence of an adverse event (AE) or serious adverse event (SAE) may come to the attention of study personnel during study visits and interviews of a study participant presenting for medical care, or upon review by a study monitor. At weekly medication visits, all participants will review a side effects checklist (SAFTEE) with a research assistant, which will then be provided for review by a medical staff member at CTRC. The staff member will begin with an open-ended question about side effects or any other adverse events, followed by a review of specific side effects using the SAFTEE. Any previously reported side effects will be queried to determine any change in status (presence or severity). These weekly visits will be a primary method for identifying side effects. Additionally, a research staff member will contact participants by phone in the week following the initial dose, as well as the week following each dose escalation. Phone calls will be scheduled 2-3 days following these events.

All AEs including local and systemic reactions not meeting the criteria for SAEs will be captured on the appropriate case report form (CRF). Information to be collected includes event description, time of onset, clinician's assessment of severity, relationship to study product (assessed only by those with the training and authority to make a diagnosis), and time of resolution/stabilization of the event. All AEs occurring while on study will be documented appropriately regardless of relationship. All AEs will be followed to adequate resolution.

Any medical condition that is present at the time that the participant is screened will be considered as baseline and not reported as an AE. However, if the study participant's condition deteriorates at any time during the study, it will be recorded as an AE.

Changes in the severity of an AE will be documented to allow an assessment of the duration of the event at each level of severity to be performed. AEs characterized as intermittent require documentation of onset and duration of each episode.

Under the direction of the PI, members of the research team will record all reportable events with start dates occurring any time after informed consent is obtained until 7 (for non-serious AEs) or 30 days (for SAEs) after the last day of study participation. At each study visit, the team member will inquire about the occurrence of AE/SAEs since the last visit. Any such events will be followed for outcome information until resolution or stabilization.

8.3.5 ADVERSE EVENT REPORTING

All project staff will be trained to identify adverse events (AEs), which must be reported to the PI and study physicians within 24 hours. In addition, any unanticipated participant issues will be discussed in weekly staff meetings, which will provide another opportunity to identify AEs.

8.3.6 SERIOUS ADVERSE EVENT REPORTING

Research staff will be trained to report potential AEs or SAEs to the PI immediately. For any potential AEs/SAEs, the study physician will make a determination to distinguish between AEs, SAEs and unanticipated problems and provide judgment about attributions. Management and reporting of AEs, SAEs, and unanticipated problems will be carried out in accordance with Good Clinical Practice (GCP) standards, as outlined by the Food and Drug Administration. AEs will be defined as any negative medical event during the trial (whether or not the event is deemed related to the study medication). SAEs are defied as events that are potentially life threatening or result in heath; cause congenital malformation; result in persistent/significant disability or incapacity; require inpatient hospitalization or prolongation of hospitalization; or reflect an otherwise notable medical event in the judgment of the study physician. AEs/SAEs will be monitored regularly by the study research team and medical staff, in particular by inquiring about adverse events at each weekly visit. Should AEs or SAEs occur, they will be recorded, rated as mild/moderate/severe by the study physician, and reported in accordance with UNC IRB requirements and GCP standards. Any AEs will be reported to the IRB in progress reports and at the conclusion of the study. SAEs and unanticipated events will be reported to the IRB and DSMB within 48 hours, and to NIAAA if judged as treatment-related. All staff working on this study will be made aware of the monitoring and reporting requirements for AEs/SAEs, and will have completed GCP training and other trainings required to conduct human subjects research at UNC.

Any AEs, SAEs or unanticipated problems will be followed until resolution by the PIs and/or research staff. Participants will be contacted regularly during follow-up and any observations of AEs/SAEs will be discussed among the PIs and the study physician at regular meetings (or immediately, in the event of SAEs or unanticipated problems). In the event of SAEs or unanticipated problems, the physician and study PIs will be notified immediately, and the participant will be referred to or provided with appropriate medical support.

Participants will be advised to seek prompt medical attention in the event of an unanticipated medical event. In the event that the randomization code needs to be broken to appropriately resolve an SAE, the study pharmacist or delegate will break the blind and reveal the status to the study physician. In the event of an SAE, a member of the study medical staff will maintain regular contact with the participant until the event is resolved and fully documented.

Monitoring will be the primary responsibility of the PI. In addition, a Data and Safety Monitoring Board will be established to maximize safety (the TraCS DSMB will be involved for this project, and will review progress at their scheduled meetings at planned intervals during the trial).

8.3.7 REPORTING EVENTS TO PARTICIPANTS

If an event is deemed an AE or SAE, participants will receive a phone call from study personnel to inform them that the event has been recorded and will be reported as such. Any incidental findings uncovered during the baseline medical exam or weekly visits (e.g., via bloodwork or other measurements) will be reported to the participant by the study physician, and an appropriate follow-up plan will be recommended to the participant.

8.3.8 EVENTS OF SPECIAL INTEREST

Not applicable

8.3.9 REPORTING OF PREGNANCY

At the baseline visit and each study visit that involves an alcohol session, female subjects will undergo a urine pregnancy test upon arrival to the clinical site. In the case of a positive pregnancy test, the participant will be notified immediately, and study intervention will be discontinued while continuing safety follow-up. If any follow-up plan is recommended by the study physician, one of the staff members will communicate this recommendation to the participant.

8.4 UNANTICIPATED PROBLEMS

8.4.1 DEFINITION OF UNANTICIPATED PROBLEMS (UP)

The Office for Human Research Protections (OHRP) considers unanticipated problems involving risks to participants or others to include, in general, any incident, experience, or outcome that meets **all** of the following criteria:

- Unexpected in terms of nature, severity, or frequency given (a) the research procedures that are described in the protocol-related documents, such as the Institutional Review Board (IRB)-approved research protocol and informed consent document; and (b) the characteristics of the participant population being studied;
- Related or possibly related to participation in the research ("possibly related" means there is a reasonable possibility that the incident, experience, or outcome may have been caused by the procedures involved in the research); and
- Suggests that the research places participants or others at a greater risk of harm (including physical, psychological, economic, or social harm) than was previously known or recognized.

Any UPs identified by the study team will be discussed amongst the study leadership team, and corrective actions will be identified. If applicable, a protocol amendment will be developed and submitted to the IRB.

8.4.2 UNANTICIPATED PROBLEM REPORTING

The investigator will report unanticipated problems (UPs) to the reviewing Institutional Review Board (IRB), and if applicable, the DSMB. The UP report will include the following information:

- Protocol identifying information: protocol title and number, PI's name, and the IRB project number;
- A detailed description of the event, incident, experience, or outcome;
- An explanation of the basis for determining that the event, incident, experience, or outcome represents an UP;
- A description of any changes to the protocol or other corrective actions that have been taken or are proposed in response to the UP.

8.4.3 REPORTING UNANTICIPATED PROBLEMS TO PARTICIPANTS

In the case of a UP that may impact participants' safety or alter their experience during the trial (e.g., by necessitating a change in protocol), participants will be notified by a study team member at the subsequent visit. In the event of any UP that may have an imminent impact on participant safety, a team member will contact the participant by phone to inform them of the problem.

9 STATISTICAL CONSIDERATIONS

9.1 STATISTICAL HYPOTHESES

- Primary Efficacy Endpoint(s):
    - Breath alcohol concentration (BrAC) and volume of alcohol consumed during laboratory alcohol self-administration procedures. The outcomes are continuous variables. The effect of interest is the interaction between medication group (semaglutide vs. placebo) and time/dosage (baseline vs. 0.5mg). The baseline session will take place between Weeks 0 and 1, and the 0.5mg session will take place between weeks 8 and 9.
- Secondary Efficacy Endpoint(s):
    - Changes in subjective responses to alcohol (stimulation, sedation, craving) and alcohol and cigarette demand during laboratory alcohol challenge procedures. The outcomes will be analyzed as continuous variables. The effect of interest is the interaction between medication group (semaglutide vs. placebo) and time/dosage (baseline vs. 0.5mg). The baseline session will take place between Weeks 0 and 1, and the 0.5mg session will take place between weeks 8 and 9.
    - Changes in drinks per day and cigarette consumption over the course of the trial, as assessed via daily surveys. The outcome variables will be treated as continuous, however, this assumption will be tested and the modeling approach will revised as needed to properly account for the nature of the outcome variable (e.g., analyze as count variable with negative binomial distribution). The effect of interest is the interaction between medication group (semaglutide vs. placebo) and time (Week 1 to Week 10).

9.2 SAMPLE SIZE DETERMINATION

Because no human data on semaglutide's effects on alcohol consumption outcomes are available, there is no a priori effect size estimate to facilitate power calculations. We will seek a final sample size of 36 participants. Assuming at least 36 enrollees have complete data, our analyses suggest that this allows sufficient power (.80) to detect a medium effect size (f = .25) for an interaction between group (medication vs. placebo) and dosage (baseline, 0.25mg, 0.5mg). This estimate assumes an alpha level of .05 and a within-person correlation (r) of 0.5 across time points. Further, because our multilevel model (MLM) analyses will involve more time points for certain analyses (e.g., daily assessments), this approach will increase power further, which is advantageous in the event that medication effects are in

the small range. Assuming a 25% attrition rate, we anticipate needing to randomize 48 enrollees to arrive at a final sample of 36.

9.3 POPULATIONS FOR ANALYSES

Primary analyses will include all participants who were randomized and received a medication or placebo dose following the Week 0 visit (randomization will occur after the Week 0 visit, just prior to the Week 1 visit). MLM analyses can accommodate missing data, allowing inclusion of participants without complete data (e.g., missed visits or missed daily survey responses). Reasons for missing data and drop-out will be documented in the study database and reported in any publications.

A supplementary per-protocol analyses will be conducted using all participants who received 1) at least 7 of the 9 scheduled doses, and 2) those participants who receive the final (1.0mg) dose. This planned analysis will be conducted irrespective of the number of participants in these groups. If the sample permits, an exploratory subgroup analysis will examine participants as a function of weight (overweight/obese vs. not overweight or obese).

9.4 STATISTICAL ANALYSES

9.4.1 GENERAL APPROACH

Primary analyses will rely on multilevel models (MLM), which are well suited to examining within-person changes over time, while also accommodating missing data and unequal group sizes, and allowing the evaluation of between-subjects moderators (e.g., overweight/obesity status) if necessary. MLM with maximum likelihood estimation can accommodate missing data under the assumption that the data are missing at random. Secondary, sensitivity analyses will be conducted to test the robustness of study findings for violations of the assumption that data is missing at random. Specifically, we will examine if baseline variables are associated with data missingness (for instance, age, sex, level of substance use) using t-tests and chi-square analyses (for continuous and dichotomous variables, respectively). Baseline variables that are associated with data missingness will be included as covariates in additional adjusted models (that adjust for the baseline variables related to missingness). Assumptions for the statistical methods being used will be tested. Should there be evidence of violations of these assumptions (e.g., presence of outlying and influential cases, distributional concerns for residuals, and so on), appropriate corrective actions will be taken. Sensitivity analyses will be used to determine the extent to which the main study results are influenced by these violations. We will focus our interpretations on small p-values ($p < .05$) that indicate evidence against the null hypothesis. We will report the number of decimal places of the p-value in line with the journal's guidelines and APA recommendations (usually 2 or 3 decimal places). For specific outcomes, the units of measurement and expected ranges of values (or example ranges, if the full range is unknown) are noted in the subsequent analysis sections 9.4.2 and 9.4.3. Any extreme outliers (defined as >3.0 standard deviations from the mean) will be recoded to one unit greater than the next most extreme value, in order to reduce undue influence of extreme outliers. For all statistical tests, confidence intervals will be evaluated in combination with tests of statistical significance. Assumptions for the statistical methods being used will be tested. Should there be evidence of violations of these assumptions (e.g., presence of outlying and influential cases, distributional concerns for residuals, and so on), appropriate corrective actions will be taken. Sensitivity analyses will be used to determine the extent to which the main study results are influenced by these violations.

9.4.2 ANALYSIS OF THE PRIMARY EFFICACY ENDPOINT(S)

Alcohol self-administration sessions: MLM will be used to examine changes in laboratory self-administration (defined as peak BAC and quantity of alcohol consumed, co-primary outcomes) as a function of medication phase (baseline; 0.5mg). These analyses will use medication phase as the within-subjects factor and condition (semaglutide vs. placebo) as the between-person factor. Self-administration will be measured based on recorded breath alcohol concentration (BrAC), measured in g% (e.g., .00g% - .08g%).

9.4.3 ANALYSIS OF THE SECONDARY ENDPOINT(S)

Alcohol challenge sessions: MLM will also be used to examine changes in 1) cigarette and alcohol demand during alcohol challenge (based on corresponding purchase tasks) (e.g., based on number of hypothetical drinks/cigarettes consumed at a given price point), and 2) self-reported stimulation (0-10 range), sedation (0-10 range), and craving (1-7 range), from BAC = .00 to BAC = .06g%, as a function of medication phase (within-person) and drug condition (between person). These analyses will examine changes in outcomes from BAC = .00 to .06 using medication phase as the within-subjects factor and condition as the between-person factor. The analyses will be complemented by analyses of peak subjective effects (e.g., stimulation, sedation, craving, on the same scales as noted above), across the two alcohol administration sessions (AC1 and AC2).

Daily self-report data: We will use MLM to estimate within-person changes in primary outcomes (e.g., daily alcohol use [number of drinks consumed per day] and cigarette use [number of cigarettes smoked per day] over the trial period), using study week as the within-subjects factor and condition (semaglutide, placebo) as the between-person factor.

9.4.4 SAFETY ANALYSES

Total side effects reported (e.g., 0-15), and number of severe side effects reported during each medication phase will be assessed using MLM with medication phase (baseline, 0.25mg, 0.5mg, 1.0mg) as the within-subject factor and condition (semaglutide, placebo) as the between-person factor. . These count variables will be analyzed using a negative binomial MLM, however, this assumption will be tested and the modeling approach will revised as needed to properly account for the nature of the outcome variable (e.g., zero-inflated negative binomial regression).

9.4.5 BASELINE DESCRIPTIVE STATISTICS

Descriptive statistics will be used to report relevant demographics such as age, sex, and education.

9.4.6 PLANNED INTERIM ANALYSES

No interim analyses of efficacy will be planned.

9.4.7 SUB-GROUP ANALYSES

Not applicable

9.4.8 TABULATION OF INDIVIDUAL PARTICIPANT DATA

Not applicable

9.4.9 EXPLORATORY ANALYSES

Sex will be examined as an exploratory moderator in all analyses.

10 SUPPORTING DOCUMENTATION AND OPERATIONAL CONSIDERATIONS

10.1 REGULATORY, ETHICAL, AND STUDY OVERSIGHT CONSIDERATIONS

10.1.1 INFORMED CONSENT PROCESS

In obtaining and documenting informed consent, the team will comply with applicable regulatory requirements (e.g., 45 CFR Part 46, 21 CFR Part 50, 21 CFR Part 56) and adhere to ICH GCP. Consent will be obtained remotely (via web teleconference and Docusign, as described previously) by a qualified study staff member in person at the outset of the baseline session. Additional procedures are noted below.

10.1.1.1 CONSENT/ASSENT AND OTHER INFORMATIONAL DOCUMENTS PROVIDED TO PARTICIPANTS

Consent forms will describe the study intervention, study procedures, and potential risks. The participant's electronic signature indicating consent is required prior to starting intervention/administering study intervention. Participants will also be given an informational sheet about the study medication. Additional procedures are described below.

10.1.1.2 CONSENT PROCEDURES AND DOCUMENTATION

Informed consent is a process that is initiated prior to the individual's agreeing to participate in the study and continues throughout the individual's study participation. Consent forms will be Institutional Review Board (IRB)-approved and the participant will be asked to read and review the document. The initial consent process will be conducted remotely using a HIPAA-compliant web platform (e.g., WebEx, Zoom, Teams) and Part 11-compliant Docusign to maintain optimal safety. A copy of the consent form will be provided to the participant in advance of the scheduled videoconference. A qualified team member will explain the research study to the participant and answer any questions that may arise. A verbal explanation will be provided in terms suited to the participant's comprehension of the purposes, procedures, and potential risks of the study and of their rights as research participants. Participants will have the opportunity to carefully review the consent form and ask questions prior to signing. The participants should have the opportunity to discuss the study with their family or surrogates or think about it prior to agreeing to participate. The participant will electronically sign the informed consent

document prior to any procedures being done specifically for the study. Participants must be informed that participation is voluntary and that they may withdraw from the study at any time, without prejudice. A copy of the signed informed consent document will be given to the participants for their records. The informed consent process will be conducted and documented in the source document (including the date), and the form signed (via Docusign), before the participant undergoes any study-specific procedures. The rights and welfare of the participants will be protected by emphasizing to them that the study is voluntary and the quality of their medical care will not be adversely affected if they decline to participate. While the initial consent form will be conducted electronically, supplementary consent forms (e.g., consent to unencrypted communication, HIPAA authorization, and biospecimen consent) may be conducted either remotely (via Docusign) in advance of the baseline visit or in person (paper form) at the baseline visit, depending on timing.

10.1.2 STUDY DISCONTINUATION AND CLOSURE

This study may be temporarily suspended or prematurely terminated if there is sufficient reasonable cause. Written notification, documenting the reason for study suspension or termination, will be provided by the suspending or terminating party to study participants, investigator, funding agency, and regulatory authorities. If the study is prematurely terminated or suspended, the Principal Investigator (PI) will promptly inform study participants, the Institutional Review Board (IRB), and sponsor and will provide the reason(s) for the termination or suspension. Study participants will be contacted, as applicable, and be informed of changes to study visit schedule. Circumstances that may warrant termination or suspension include, but are not limited to determination of unexpected, significant, or unacceptable risk to participants. Study may resume once concerns about safety, protocol compliance, and data quality are addressed, and satisfy the sponsor, IRB and/or Food and Drug Administration (FDA).

10.1.3 CONFIDENTIALITY AND PRIVACY

Participant confidentiality and privacy is strictly held in trust by the participating investigators, their staff, and the sponsor(s) and their interventions. This confidentiality is extended to cover testing of biological samples and genetic tests in addition to the clinical information relating to participants. Therefore, the study protocol, documentation, data, and all other information generated will be held in strict confidence. No information concerning the study or the data will be released to any unauthorized third party. All research activities will be conducted in as private a setting as possible.

Any computerized data will be password protected and contained on a secure, password-protected computer or a secure server. Recruitment data (de-identified) will be stored in a secure, password-protected file accessible only to the study investigators and personnel. At the end of recruitment, eligible subject files will be separated from those failing to meet eligibility criteria, and records of ineligible recruitment subjects will be anonymized. Data pertaining to screening failures will be tabulated in an anonymized fashion. Data collected in other formats (e.g., medical screening notes) will be labeled only by study identifier and stored in binders in secure, locked cabinets while the study is taking place. Files will be stored and archived in accordance with Good Clinical Practice (GCP) standards. For all questionnaire and interview assessments, participants will be informed that they may decline to answer any question if they so choose.

The study monitor, other authorized representatives of the sponsor, representatives of the Institutional Review Board (IRB), regulatory agencies or pharmaceutical company supplying study product may

inspect all documents and records required to be maintained by the investigator, including but not limited to, medical records (office, clinic, or hospital) and pharmacy records for the participants in this study. The clinical study site will permit access to such records.

The study participant's contact information will be securely stored during the study for purposes of maintain contact with participants, and for communicating any safety information or unanticipated problems. At the end of the study, all records will continue to be kept in a secure location for as long a period as dictated by the reviewing IRB, Institutional policies, or sponsor requirements.

Study participant research data, which is for purposes of statistical analysis and scientific reporting, will be stored at UNC in the PI's laboratory and on secure backup servers with password-protected access. This will not include the participant's contact or identifying information. Rather, individual participants and their research data will be identified by a unique study identification number. Electronic data are stored on a password protected drive only accessible by the research team. Data obtained on paper will be transferred to an electronic data file (Excel, SPSS, REDCap), stored on a secure server, and identified by participants ID. For non-computer based forms, the data collection sheets are stored in a locked cabinet in a separate locked data storage space at the PI's laboratory.

At the end of the study, all study databases will be de-identified and archived in the PI's laboratory and backed up on a secure server hosted at UNC. De-identified data resulting from this study may also be presented at meetings, published in journals or presentations. In addition, this study will be registered at ClinicalTrials.gov, and de-identified information from this study will be submitted to ClinicalTrials.gov.

10.1.4 FUTURE USE OF STORED SPECIMENS AND DATA

Data collected for this study will be analyzed and stored at the PI's laboratory. The study biostatistician will be given access to the data necessary for conducting specified analyses, and data will be de-identified prior to giving access.

De-identified biological samples will be stored at UNC for the duration of the study, until all biological tests are completed and the data transferred to electronic format. De-identified DNA samples will be stored indefinitely, provided the participant has given consent for DNA storage, in order to examine future questions related to phenotypes of interest for this line of research.

During the conduct of the study, an individual participant can choose to withdraw consent to have biological specimens stored for future research. However, withdrawal of consent with regard to biosample storage may not be possible after the study is completed (after specimens are de-identified).

10.1.5 KEY ROLES AND STUDY GOVERNANCE

Provide the name and contact information of the Principal Investigator and the Medical Monitor.

**Principal Investigator**	**Medical Monitor**
Christian Hendershot, Ph.D., Associate Professor	*TBD (The medical monitor and DSMB members will be identified and added prior to data collection)*

UNC-Chapel Hill	
104 Manning Dr.	
Christian_Hendershot@med.unc.edu	

The investigative team is summarized on the face page of this protocol. As noted, a DSMB will be formed prior to carrying out the study.

Safety oversight will be under the direction of a Data and Safety Monitoring Board (DSMB). Members of the DSMB will be independent from the study conduct and free of conflict of interest. The DSMB will dictate the intervals at which they meet to assess safety and efficacy data. At this time, each data element that the DSMB needs to assess will be clearly defined. The DSMB will provide its input to the IRB and National Institutes of Health as necessary.

10.1.6 CLINICAL MONITORING

Clinical site monitoring is conducted to ensure that the rights and well-being of trial participants are protected, that the reported trial data are accurate, complete, and verifiable, and that the conduct of the trial is in compliance with the currently approved protocol/amendment(s), with International Conference on Harmonisation Good Clinical Practice (ICH GCP), and with applicable regulatory requirement(s). A local study monitor will be appointed to provide on-site monitoring during the course of the research study.

10.1.7 QUALITY ASSURANCE AND QUALITY CONTROL

The PI will be responsible for addressing quality control issues and correcting errors or protocol deviations. The PI will have oversight over training study staff and ensuring that protocol elements are carried out with fidelity. Quality control will be ensured by developing SOPs for protocol elements to standardize data collection procedures. The investigational site will provide direct access to all trial related sites, source data/documents, and reports for the purpose of monitoring and auditing by the sponsor, and inspection by local and regulatory authorities.

10.1.8 DATA HANDLING AND RECORD KEEPING

10.1.8.1 DATA COLLECTION AND MANAGEMENT RESPONSIBILITIES

As a single-site, small-scale trial, the project will not involve coordinated data collection and management responsibilities across sites/teams. Data safety and storage precautions are outlined elsewhere in the study protocol.

10.1.8.2 STUDY RECORDS RETENTION

Study records will be retained for a minimum of 7 years following study completion. Paper source documents will be archived in accordance with UNC policies. Electronic materials and de-identified datasets will be stored securely as outlined above.

10.1.9 PROTOCOL DEVIATIONS

A protocol deviation is any noncompliance with the clinical trial protocol, International Conference on Harmonisation Good Clinical Practice (ICH GCP), or Manual of Procedures (MOP) requirements. The PI will report deviations within 7 working days of identification of the protocol deviation, or within 7 working days of the scheduled protocol-required activity. All deviations must be addressed in study source documents, reported to NIAAA Program Official and the IRB. Protocol deviations must be sent to the reviewing Institutional Review Board (IRB) per their policies. The site investigator is responsible for knowing and adhering to the reviewing IRB requirements. Further details about the handling of protocol deviations will be included in the MOP.

10.1.10 PUBLICATION AND DATA SHARING POLICY

This study will be conducted in accordance with the following publication and data sharing policies and regulations:

National Institutes of Health (NIH) Public Access Policy, which ensures that the public has access to the published results of NIH funded research. It requires scientists to submit final peer-reviewed journal manuscripts that arise from NIH funds to the digital archive PubMed Central upon acceptance for publication.

This study will comply with the NIH Data Sharing Policy and Policy on the Dissemination of NIH-Funded Clinical Trial Information and the Clinical Trials Registration and Results Information Submission rule. As such, this trial will be registered at ClinicalTrials.gov, and results information from this trial will be submitted to ClinicalTrials.gov. In addition, every attempt will be made to publish results in peer-reviewed journals. Data from this study may be requested from other researchers after the completion of the primary endpoint by contacting the PI.

10.1.11 CONFLICT OF INTEREST POLICY

Any actual conflict of interest of persons who have a role in the design, conduct, analysis, publication, or any aspect of this trial will be disclosed and managed. Furthermore, persons who have a perceived conflict of interest will be required to have such conflicts managed in a way that is appropriate to their participation in the design and conduct of this trial. All investigators will make a conflict of interest declaration at the outset of the study and at regular intervals thereafter in accordance with UNC policies.

10.2 ADDITIONAL CONSIDERATIONS

Not applicable

10.3 ABBREVIATIONS

AASE	Alcohol Abstinence Self-Efficacy
AE	Adverse Event
APT	Alcohol Purchase Task
AUD	Alcohol Use Disorder
AUDIT	Alcohol Use Disorder Identification Test
AUQ	Alcohol Urge Questionnaire
BAES	Biphasic Alcohol Effects Scale
BIS-11	Barratt Impulsivity Scale
BMI	Body Mass Index
BrAC	Breath Alcohol Concentration
CASE	Cigarette Abstinence Self-Efficacy
CES-D	Center for Epidemiological Studies Depression Scale
CEQ	Control of Eating Questionnaire
CFR	Code of Federal Regulations
CIWA	Clinical Institutes of Withdrawal Assessment
CMP	Clinical Monitoring Plan
CO	Carbon Monoxide
CPT	Cigarette Purchase Task
CRF	Case Report Form
CTRC	Clinical & Translational Research Center
DEQ	Drug Effects Questionnaire
DNA	Deoxyribonucleic acid
DSM-5	Diagnostic and Statistical Manual for Mental Disorders v.5
DSMB	Data Safety Monitoring Board
FDA	Food and Drug Administration
FPT	Food Purchase Task
FTND	Fagerstrom test for Nicotine Dependence
GCP	Good Clinical Practice
GLP-1	Glucagon-Like Peptide 1
GLP-1R	Glucagon-Like Peptide 1 Receptor
HbA1c	Glycated hemoglobin
ICH	International Conference on Harmonisation
ICS	Impaired Control Scale
IDS	Inventory of Drinking Situations
IRB	Institutional Review Board
MCQ	Monetary Choice Questionnaire
MNWS	Minnesota Nicotine Withdrawal Scale
MOP	Manual of Procedures
NC Tracs	North Carolina Translational & Clinical Sciences Institute
NDSS	Nicotine Dependence Syndrome Scale
NIAAA	National Institute on Alcohol Abuse and Alcoholism
NIH	National Institutes of Health
NRT	Nicotine Replacement Therapy
PACS	Penn Alcohol Craving Scale
PI	Principal Investigator
POMS-SF	Profile Of Mood States Short Form
QA	Quality Assurance

QC	Quality Control
RBEDS	Reward Based Eating Drive Scale
RPI-EROS	Reward Probability Index – Environmental Reward Observation Scale
SAE	Serious Adverse Event
SAFTEE	Systematic Assessment for Treatment Emergent Effects
SNAQ	Simplified Nutritional Appetite Questionnaire
SOA	Schedule of Activities
SOP	Standard Operating Procedure
SRE	Self-Report of the Effects of Alcohol Questionnaire
SUD	Substance Use Disorder
T2D	Type II Diabetes
TAS-20	Toronto Alexithymia Scale
TFEQ	Three Factor Eating Questionnaire
TLFB	Timeline Follow-Back Interview
TQSU	Tiffany Questionnaire of Smoking Urges
UP	Unanticipated Problem
UPPS-P	UPPS-P Impulsive Behavior Scale
WISDM	Wisconsin Inventory of Smoking Dependence Motives
WSWS	Wisconsin Smoking Withdrawal Scale
5-HT2C	Serotonin 2C Receptor

10.4 PROTOCOL AMENDMENT HISTORY

The table below is intended to capture changes of IRB-approved versions of the protocol, including a description of the change and rationale. A Summary of Changes table for the current amendment is located in the Protocol Title Page.

Version	Date	Description of Change	Brief Rationale
1.2	July 26 2021	Elaboration on statistical analyses and clarification on other procedural issues.	Minor changes requested during initial review of the protocol.

11 REFERENCES

1. Koob, G.F., G. Kenneth Lloyd, and B.J. Mason, *Development of pharmacotherapies for drug addiction: a Rosetta stone approach.* Nat Rev Drug Discov, 2009. **8**(6): p. 500-15.
2. Litten, R.Z., et al., *Medications development to treat alcohol dependence: a vision for the next decade.* Addiction biology, 2012. **17**(3): p. 513-27.
3. Litten, R.Z., et al., *Discovery, Development, and Adoption of Medications to Treat Alcohol Use Disorder: Goals for the Phases of Medications Development.* Alcohol Clin Exp Res, 2016. **40**(7): p. 1368-79.
4. Litten, R.Z., et al., *Potential medications for the treatment of alcohol use disorder: An evaluation of clinical efficacy and safety.* Subst Abus, 2016. **37**(2): p. 286-98.
5. Litten, R.Z., et al., *Heterogeneity of alcohol use disorder: understanding mechanisms to advance personalized treatment.* Alcohol Clin Exp Res, 2015. **39**(4): p. 579-84.
6. Heilig, M., et al., *Pharmacogenetic approaches to the treatment of alcohol addiction.* Nature reviews. Neuroscience, 2011. **12**(11): p. 670-84.
7. Falk, D.E., H.Y. Yi, and S. Hiller-Sturmhofel, *An epidemiologic analysis of co-occurring alcohol and tobacco use and disorders: findings from the National Epidemiologic Survey on Alcohol and Related Conditions.* Alcohol Res Health, 2006. **29**(3): p. 162-71.
8. McKee, S.A. and A.H. Weinberger, *How can we use our knowlegde of alcohol-tobacco interactions to reduce alcohol use?* Annu Rev Clin Psychol, 2013. **9**: p. 649–674.
9. Organization, W.H., *Global Health Risks. Mortality and Burden of Disease Attributable to Selected Major Risks*. 2009, World Health Organization: Geneva, Switzerland.
10. Dawson, D.A., *Drinking as a risk factor for sustained smoking.* Drug Alcohol Depend, 2000. **59**(3): p. 235-49.
11. Yardley, M.M., M.M. Mirbaba, and L.A. Ray, *Pharmacological Options for Smoking Cessation in Heavy-Drinking Smokers.* CNS Drugs, 2015. **29**(10): p. 833-45.
12. Prabhu, A., K.O. Obi, and J.H. Rubenstein, *The synergistic effects of alcohol and tobacco consumption on the risk of esophageal squamous cell carcinoma: a meta-analysis.* Am J Gastroenterol, 2014. **109**(6): p. 822-7.
13. Pelucchi, C., et al., *Cancer risk associated with alcohol and tobacco use: focus on upper aero-digestive tract and liver.* Alcohol Res Health, 2006. **29**(3): p. 193-8.
14. Kahler, C.W., N.S. Spillane, and J. Metrik, *Alcohol use and initial smoking lapses among heavy drinkers in smoking cessation treatment.* Nicotine Tob Res, 2010. **12**(7): p. 781-5.
15. Roche, D.J., et al., *Current insights into the mechanisms and development of treatments for heavy drinking cigarette smokers.* Curr Addict Rep, 2016. **3**(1): p. 125-137.
16. McKee, S.A., et al., *Varenicline reduces alcohol self-administration in heavy-drinking smokers.* Biol Psychiatry, 2009. **66**(2): p. 185-90.
17. King, A., et al., *Naltrexone decreases heavy drinking rates in smoking cessation treatment: an exploratory study.* Alcohol Clin Exp Res, 2009. **33**(6): p. 1044-50.
18. O'Malley, S.S., et al., *Dose-dependent reduction of hazardous alcohol use in a placebo-controlled trial of naltrexone for smoking cessation.* Int J Neuropsychopharmacol, 2009. **12**(5): p. 589-97.
19. King, A., et al., *Effects of the opioid receptor antagonist naltrexone on smoking and related behaviors in smokers preparing to quit: a randomized controlled trial.* Addiction, 2013. **108**(10): p. 1836-44.
20. Anton, R.F., et al., *Nicotine-Use/Smoking Is Associated with the Efficacy of Naltrexone in the Treatment of Alcohol Dependence.* Alcohol Clin Exp Res, 2018. **42**(4): p. 751-760.
21. Fucito, L.M., et al., *Cigarette smoking predicts differential benefit from naltrexone for alcohol dependence.* Biol Psychiatry, 2012. **72**(10): p. 832-8.
22. Hartmann-Boyce, J., et al., *Efficacy of interventions to combat tobacco addiction: Cochrane update of 2013 reviews.* Addiction, 2014. **109**(9): p. 1414-25.
23. Kahler, C.W., et al., *A Double-Blind Randomized Placebo-Controlled Trial of Oral Naltrexone for Heavy-Drinking Smokers Seeking Smoking Cessation Treatment.* Alcohol Clin Exp Res, 2017. **41**(6): p. 1201-1211.

24. Mitchell, J.M., et al., *Varenicline decreases alcohol consumption in heavy-drinking smokers.* Psychopharmacology (Berl), 2012. **223**(3): p. 299-306.
25. Fucito, L.M., et al., *A preliminary investigation of varenicline for heavy drinking smokers.* Psychopharmacology (Berl), 2011. **215**(4): p. 655-63.
26. Litten, R.Z., et al., *A double-blind, placebo-controlled trial assessing the efficacy of varenicline tartrate for alcohol dependence.* J Addict Med, 2013. **7**(4): p. 277-86.
27. Roche, D.J., et al., *Combined varenicline and naltrexone treatment reduces smoking topography intensity in heavy-drinking smokers.* Pharmacol Biochem Behav, 2015. **134**: p. 92-8.
28. Ray, L.A., et al., *Varenicline, low dose naltrexone, and their combination for heavy-drinking smokers: human laboratory findings.* Psychopharmacology (Berl), 2014. **231**(19): p. 3843-53.
29. Andersen, A., et al., *Glucagon-like peptide 1 in health and disease.* Nature Reviews Endocrinology, 2018. **14**(7): p. 390-403.
30. Jerlhag, E., *Alcohol-mediated behaviours and the gut-brain axis; with focus on glucagon-like peptide-1.* Brain Res, 2020. **1727**: p. 146562.
31. Brunchmann, A., M. Thomsen, and A. Fink-Jensen, *The effect of glucagon-like peptide-1 (GLP-1) receptor agonists on substance use disorder (SUD)-related behavioural effects of drugs and alcohol: A systematic review.* Physiol Behav, 2019. **206**: p. 232-242.
32. Egecioglu, E., J.A. Engel, and E. Jerlhag, *The glucagon-like peptide 1 analogue Exendin-4 attenuates the nicotine-induced locomotor stimulation, accumbal dopamine release, conditioned place preference as well as the expression of locomotor sensitization in mice.* PLoS One, 2013. **8**(10): p. e77284.
33. Anderberg, R.H., et al., *Glucagon-Like Peptide 1 and Its Analogs Act in the Dorsal Raphe and Modulate Central Serotonin to Reduce Appetite and Body Weight.* Diabetes, 2017. **66**(4): p. 1062-1073.
34. Howell, E., et al., *Glucagon-Like Peptide-1 (GLP-1) and 5-Hydroxytryptamine 2c (5-HT(2c)) Receptor Agonists in the Ventral Tegmental Area (VTA) Inhibit Ghrelin-Stimulated Appetitive Reward.* Int J Mol Sci, 2019. **20**(4).
35. Asarian, L., *Loss of cholecystokinin and glucagon-like peptide-1-induced satiation in mice lacking serotonin 2C receptors.* American Journal of Physiology-Regulatory, Integrative and Comparitive Physiology, 2009. **296**(1): p. R51-R56.
36. Nonogaki, K., et al., *The contribution of serotonin 5-HT2C and melanocortin-4 receptors to the satiety signaling of glucagon-like peptide 1 and liraglutide, a glucagon-like peptide 1 receptor agonist, in mice.* Biochem Biophys Res Commun, 2011. **411**(2): p. 445-8.
37. Doggrell, S.A., *Sgemaglutide in type 2 diabetes - is it the best glucagon-like peptide 1 receptor agonist (GLP-1R agonist)?* Expert Opin Drug Metab Toxicol, 2018. **14**(3): p. 371-377.
38. Litten, R.Z., et al., *Development of medications for alcohol use disorders: recent advances and ongoing challenges.* Expert Opin Emerg Drugs, 2005. **10**(2): p. 323-43.
39. Bujarski, S. and L.A. Ray, *Experimental psychopathology paradigms for alcohol use disorders: Applications for translational research.* Behav Res Ther, 2016.
40. Hendershot, C.S., et al., *Effects of naltrexone on alcohol self-administration and craving: meta-analysis of human laboratory studies.* Addict Biol, 2016.
41. McKee, S.A., *Developing human laboratory models of smoking lapse behavior for medication screening.* Addict Biol, 2009. **14**(1): p. 99-107.
42. Plebani, J.G., et al., *Human laboratory paradigms in alcohol research.* Alcohol Clin Exp Res, 2012. **36**(6): p. 972-83.
43. Ray, L.A., K.E. Hutchison, and M. Tartter, *Application of human laboratory models to pharmacotherapy development for alcohol dependence.* Curr Pharm Des, 2010. **16**(19): p. 2149-58.
44. Lerman, C., et al., *Translational research in medication development for nicotine dependence.* Nat Rev Drug Discov, 2007. **6**(9): p. 746-62.
45. MacKillop, J. and J.G. Murphy, *A behavioral economic measure of demand for alcohol predicts brief intervention outcomes.* Drug Alcohol Depend, 2007. **89**(2-3): p. 227-33.
46. Amlung, M., et al., *Increased behavioral economic demand and craving for alcohol following a laboratory alcohol challenge.* Addiction, 2015. **110**(9): p. 1421-8.
47. Green, R. and L.A. Ray, *Effects of varenicline on subjective craving and relative reinforcing value of cigarettes.* Drug Alcohol Depend, 2018. **188**: p. 53-59.

48. Murphy, C.M., et al., *Effects of varenicline versus transdermal nicotine replacement therapy on cigarette demand on quit day in individuals with substance use disorders.* Psychopharmacology (Berl), 2017. **234**(16): p. 2443-2452.
49. Roache, J.D., *Role of the Human Laboratory in the Development of Medications for Alcohol and Drug Dependence*, in *Addiction Medicine*, B.A. Johnson, Editor. 2010, Springer: New York. p. 129-157.
50. Enoch, M.A., et al., *Ethical considerations for administering alcohol or alcohol cues to treatment-seeking alcoholics in a research setting: can the benefits to society outweigh the risks to the individual? A commentary in the context of the National Advisory Council on Alcohol Abuse and Alcoholism -- Recommended Council Guidelines on Ethyl Alcohol Administration in Human Experimentation (2005).* Alcohol Clin Exp Res, 2009. **33**(9): p. 1508-12.
51. Motschman, C.A., et al., *Selection criteria limit generalizability of smoking pharmacotherapy studies differentially across clinical trials and laboratory studies: A systematic review on varenicline.* Drug Alcohol Depend, 2016. **169**: p. 180-189.
52. Alcoholism, N.I.o.A.A.a., *Recommended council guidelines on ethyl alcohol administration in human experimentation*. 2005, Department of Health and Human Services: Rockville, MD.
53. First MB, W.J., Karg RS, Spitzer RL, *Structured Clinical Interview for DSM-5—Research Version (SCID-5 for DSM-5, Research Version; SCID-5-RV)*. 2015, Arlington, VA: American Psychiatric Association.
54. Sobell, L.C., et al., *Reliability of a timeline method: assessing normal drinkers' reports of recent drinking and a comparative evaluation across several populations.* Br J Addict, 1988. **83**(4): p. 393-402.
55. Saunders, J.B., et al., *Development of the Alcohol Use Disorders Identification Test (AUDIT): WHO Collaborative Project on Early Detection of Persons with Harmful Alcohol Consumption--II.* Addiction, 1993. **88**(6): p. 791-804.
56. Schuckit, M.A., et al., *The Self-Rating of the Effects of Alcohol Questionnaire as a Predictor of Alcohol-Related Outcomes in 12-Year-Old Subjects.* Alcohol and Alcoholism, 2008. **43**(6): p. 641-646.
57. Schuckit, M.A. and T.L. Smith, *The relationships of a family history of alcohol dependence, a low level of response to alcohol and six domains of life functioning to the development of alcohol use disorders.* Journal of Studies on Alcohol, 2000. **61**(6): p. 827-835.
58. Heatherton, T.F., et al., *The Fagerstrom Test for Nicotine Dependence: a revision of the Fagerstrom Tolerance Questionnaire.* Br J Addict, 1991. **86**(9): p. 1119-27.
59. Shiffman, S., A. Waters, and M. Hickcox, *The nicotine dependence syndrome scale: a multidimensional measure of nicotine dependence.* Nicotine Tob Res, 2004. **6**(2): p. 327-48.
60. Patton, J.H., M.S. Stanford, and E.S. Barratt, *Factor structure of the Barratt impulsiveness scale.* Journal of clinical psychology, 1995. **51**(6): p. 768-774.
61. Whiteside, S.P., et al., *Validation of the UPPS impulsive behaviour scale: a four-factor model of impulsivity.* European Journal of personality, 2005. **19**(7): p. 559-574.
62. Heather, N., et al., *Development of a scale for measuring impaired control over alcohol consumption: a preliminary report.* Journal of studies on alcohol, 1993. **54**(6): p. 700-709.
63. Radloff, L.S., *The CES-D scale: A self-report depression scale for research in the general population.* Applied psychological measurement, 1977. **1**(3): p. 385-401.
64. Leising, D., T. Grande, and R. Faber, *The Toronto Alexithymia Scale (TAS-20): A measure of general psychological distress.* Journal of research in personality, 2009. **43**(4): p. 707-710.
65. Carvalho, J.P., et al., *The Reward Probability Index: Design and validation of a scale measuring access to environmental reward.* Behavior therapy, 2011. **42**(2): p. 249-262.
66. Tiffany, S.T. and D.J. Drobes, *The development and initial validation of a questionnaire on smoking urges.* Br J Addict, 1991. **86**(11): p. 1467-76.
67. Welsch, S.K., et al., *Development and validation of the Wisconsin Smoking Withdrawal Scale.* Exp Clin Psychopharmacol, 1999. **7**(4): p. 354-61.
68. DiClemente, C.C., et al., *The Alcohol Abstinence Self-Efficacy scale.* J Stud Alcohol, 1994. **55**(2): p. 141-8.
69. Gwaltney, C.J., et al., *Does smoking abstinence self-efficacy vary across situations? Identifying context-specificity within the Relapse Situation Efficacy Questionnaire.* J Consult Clin Psychol, 2001. **69**(3): p. 516-27.

70. Mann, K., et al., *Precision Medicine in Alcohol Dependence: A Controlled Trial Testing Pharmacotherapy Response Among Reward and Relief Drinking Phenotypes.* Neuropsychopharmacology, 2018. **43**(4): p. 891-899.
71. Sties, S.W., et al., *Simplified Nutritional Appetite Questionnaire (SNAQ) for cardiopulmonary and metabolic rehabilitation program.* Revista Brasileira de Medicina do Esporte, 2012. **18**: p. 313-317.
72. Dalton, M., et al., *Preliminary validation and principal components analysis of the Control of Eating Questionnaire (CoEQ) for the experience of food craving.* European journal of clinical nutrition, 2015. **69**(12): p. 1313-1317.
73. Lesdema, A., et al., *Characterization of the Three-Factor Eating Questionnaire scores of a young French cohort.* Appetite, 2012. **59**(2): p. 385-90.
74. Epel, E.S., et al., *The reward-based eating drive scale: a self-report index of reward-based eating.* PLoS One, 2014. **9**(6): p. e101350.
75. Johnson, B.A., N. Ait-Daoud, and J.D. Roache, *The COMBINE SAFTEE: a structured instrument for collecting adverse events adapted for clinical studies in the alcoholism field.* J Stud Alcohol Suppl, 2005(15): p. 157-67; discussion 140.
76. Kaplan, B.A., et al., *Automating scoring of delay discounting for the 21-and 27-item monetary choice questionnaires.* The Behavior Analyst, 2016. **39**(2): p. 293-304.
77. MacKillop, J., et al., *Further validation of a cigarette purchase task for assessing the relative reinforcing efficacy of nicotine in college smokers.* Experimental and clinical psychopharmacology, 2008. **16**(1): p. 57.
78. Epstein, L.H., et al., *Reinforcing value and hypothetical behavioral economic demand for food and their relation to BMI.* Eating behaviors, 2018. **29**: p. 120-127.
79. MacKillop, J. and J.G. Murphy, *A behavioral economic measure of demand for alcohol predicts brief intervention outcomes.* Drug and alcohol dependence, 2007. **89**(2-3): p. 227-233.
80. Jensterle, M., et al., *Does intervention with GLP-1 receptor agonist semaglutide modulate perception of sweet taste in women with obesity: study protocol of a randomized, single-blinded, placebo-controlled clinical trial.* Trials, 2021. **22**(1): p. 1-12.
81. Kampov-Polevoy, A.B., et al., *Association between sweet preference and paternal history of alcoholism in psychiatric and substance abuse patients.* Alcoholism: Clinical and Experimental Research, 2003. **27**(12): p. 1929-1936.
82. Weafer, J., S.H. Mitchell, and H. de Wit, *Recent translational findings on impulsivity in relation to drug abuse.* Current addiction reports, 2014. **1**(4): p. 289-300.
83. Brick, J., *Standardization of alcohol calculations in research.* Alcohol Clin Exp Res, 2006. **30**(8): p. 1276-87.
84. Martin, C.S., et al., *DEVELOPMENT AND VALIDATION OF THE BIPHASIC ALCOHOL EFFECTS SCALE.* Alcoholism-Clinical and Experimental Research, 1993. **17**(1): p. 140-146.
85. Bohn, M.J., D.D. Krahn, and B.A. Staehler, *DEVELOPMENT AND INITIAL VALIDATION OF A MEASURE OF DRINKING URGES IN ABSTINENT ALCOHOLICS.* Alcoholism-Clinical and Experimental Research, 1995. **19**(3): p. 600-606.
86. Johanson, C.E. and E.H. Uhlenhuth, *Drug preference and mood in humans: diazepam.* Psychopharmacology, 1980. **71**(3): p. 269-73.
87. MacKillop, J., et al., *Further validation of a cigarette purchase task for assessing the relative reinforcing efficacy of nicotine in college smokers.* Exp Clin Psychopharmacol, 2008. **16**(1): p. 57-65.
88. Curran, S.L., M.A. Andrykowski, and J.L. Studts, *Short form of the profile of mood states (POMS-SF): psychometric information.* Psychological assessment, 1995. **7**(1): p. 80.

Human Laboratory Screening of Semaglutide for Alcohol Use Disorder

Protocol Number: 21-4939

National Clinical Trial (NCT) Identified Number: N/A

Investigators:

Dr. Christian Hendershot (Principal Investigator)
Dr. Robyn Jordan (Co-Investigator)
Dr. Amanda Tow (Co-Investigator)
Dr. Sarah Dermody (Co-Investigator, Biostatistician)
Dr. Eric Claus (Co-Investigator)
Dr. Paul Fletcher (Other Significant Contributor)

Funded by: NIAAA

Version Number: v.1.2

26 July 2021

Summary of Changes from Previous Version:

Affected Section(s)	Summary of Revisions Made	Rationale

Table of Contents

STATEMENT OF COMPLIANCE 1
1 PROTOCOL SUMMARY 1
1.1 Synopsis 1
1.2 Schema 4
1.3 Schedule of Activities (SoA) 5
2 INTRODUCTION 6
2.1 Study Rationale 6
2.2 Background 7
2.3 Risk/Benefit Assessment 9
2.3.1 Known Potential Risks 9
2.3.2 Known Potential Benefits 11
2.3.3 Assessment of Potential Risks and Benefits 11
3 OBJECTIVES AND ENDPOINTS 12
4 STUDY DESIGN 14
4.1 Overall Design 14
4.2 Scientific Rationale for Study Design 21
4.3 Justification for Dose 22
4.4 End of Study Definition 22
5 STUDY POPULATION 22
5.1 Inclusion Criteria 22
5.2 Exclusion Criteria 23
5.3 Lifestyle Considerations 24
5.4 Screen Failures 24
5.5 Strategies for Recruitment and Retention 25
6 STUDY INTERVENTION 26
6.1 Study Intervention(s) Administration 26
6.1.1 Study Intervention Description 26
6.1.2 Dosing and Administration 26
6.2 Preparation/Handling/Storage/Accountability 27
6.2.1 Acquisition and accountability 27
6.2.2 Formulation, Appearance, Packaging, and Labeling 27
6.2.3 Product Storage and Stability 28
6.2.4 Preparation 28
6.3 Measures to Minimize Bias: Randomization and Blinding 28
6.4 Study Intervention Compliance 29
6.5 Concomitant Therapy 29
6.5.1 Rescue Medicine 29
7 STUDY INTERVENTION DISCONTINUATION AND PARTICIPANT DISCONTINUATION/WITHDRAWAL 29
7.1 Discontinuation of Study Intervention 29
7.2 Participant Discontinuation/Withdrawal from the Study 30
7.3 Lost to Follow-Up 30
8 STUDY ASSESSMENTS AND PROCEDURES 31
8.1 Efficacy Assessments 31
8.2 Safety and Other Assessments 31
8.3 Adverse Events and Serious Adverse Events 32
8.3.1 Definition of Adverse Events (AE) 32
8.3.2 Definition of Serious Adverse Events (SAE) 32
8.3.3 Classification of an Adverse Event 32

8.3.4 Time Period and Frequency for Event Assessment and Follow-Up 33
8.3.5 Adverse Event Reporting 34
8.3.6 Serious Adverse Event Reporting 34
8.3.7 Reporting Events to Participants 35
8.3.8 Events of Special Interest 35
8.3.9 Reporting of Pregnancy 35
8.4 Unanticipated Problems 35
8.4.1 Definition of Unanticipated Problems (UP) 36
8.4.2 Unanticipated Problem Reporting 36
8.4.3 Reporting Unanticipated Problems to Participants 36
9 STATISTICAL CONSIDERATIONS 36
9.1 Statistical Hypotheses 36
9.2 Sample Size Determination 37
9.3 Populations for Analyses 37
9.4 Statistical Analyses 37
9.4.1 General Approach 37
9.4.2 Analysis of the Primary Efficacy Endpoint(s) 37
9.4.3 Analysis of the Secondary Endpoint(s) 38
9.4.4 Safety Analyses 38
9.4.5 Baseline Descriptive Statistics 38
9.4.6 Planned Interim Analyses 38
9.4.7 Sub-Group Analyses 38
9.4.8 Tabulation of Individual participant Data 38
9.4.9 Exploratory Analyses 38
10 SUPPORTING DOCUMENTATION AND OPERATIONAL CONSIDERATIONS 38
10.1 Regulatory, Ethical, and Study Oversight Considerations 38
10.1.1 Informed Consent Process 39
10.1.2 Study Discontinuation and Closure 39
10.1.3 Confidentiality and Privacy 40
10.1.4 Future Use of Stored Specimens and Data 41
10.1.5 Key Roles and Study Governance 42
10.1.6 Safety Oversight 41
10.1.7 Clinical Monitoring 42
10.1.8 Quality Assurance and Quality Control 42
10.1.9 Data Handling and Record Keeping 42
10.1.10 Protocol Deviations 42
10.1.11 Publication and Data Sharing Policy 42
10.1.12 Conflict of Interest Policy 43
10.2 Additional Considerations 43
10.3 List of Abbreviations....................43
10.4 Protocol Amendment History 45
11 REFERENCES 46

STATEMENT OF COMPLIANCE

(1) The trial will be carried out in accordance with International Conference on Harmonization Good Clinical Practice (ICH GCP) and the following:

- United States (US) Code of Federal Regulations (CFR) applicable to clinical studies (45 CFR Part 46, 21 CFR Part 50, 21 CFR Part 56, 21 CFR Part 312, and/or 21 CFR Part 812)

National Institutes of Health (NIH)-funded investigators and clinical trial site staff who are responsible for the conduct, management, or oversight of NIH-funded clinical trials have completed Human Subjects Protection and ICH GCP Training.

The protocol, informed consent form(s), recruitment materials, and all participant materials will be submitted to the Institutional Review Board (IRB) for review and approval. Approval of both the protocol and the consent form must be obtained before any participant is enrolled. Any amendment to the protocol will require review and approval by the IRB before the changes are implemented to the study. In addition, all changes to the consent form will be IRB-approved; a determination will be made regarding whether a new consent needs to be obtained from participants who provided consent, using a previously approved consent form.

1 PROTOCOL SUMMARY

1.1 SYNOPSIS

Title: Human Laboratory Screening of Semaglutide for Alcohol Use Disorder

Study Description: This is an early-Phase II human laboratory trial using a randomized, placebo-controlled, dose-ranging design to investigate the effects of semaglutide (Ozempic®), a GLP-1 receptor agonist, on laboratory alcohol responses and consumption, naturalistic alcohol and cigarette consumption, and weight loss in smokers with alcohol use disorder (AUD). Participants will attend weekly visits while semaglutide dosage is increased from 0mg (placebo lead-in) to 1.0mg over a period of approximately 10 weeks. Participants will attend weekly visits for medication or placebo administration. At scheduled intervals, participants will complete 4 laboratory sessions involving alcohol self-administration and alcohol challenge to characterize medication effects on alcohol-related outcomes.

Objectives: Primary Objective: To examine effects of semaglutide vs. placebo on laboratory alcohol intake during a validated alcohol self-administration medication screening procedure.

Secondary Objectives: To examine medication vs. placebo group differences in 1) laboratory responses to alcohol, and 2) changes in naturalistic alcohol use and cigarette consumption over the course of the treatment period.

Tertiary Objectives: To generate preliminary data on weight loss, HbA1c reductions and alcohol elimination among participants with AUD during treatment with a GLP-1 receptor agonist.

Endpoints:

Primary Endpoint: Change in amount of alcohol consumed during a validated laboratory alcohol self-administration protocol (primary endpoint). The specific effect of interest is the change in laboratory consumption from placebo lead-in (0mg) to the maintenance dose (.5mg) as a function of medication condition (semaglutide vs. placebo) (i.e., treatment x time interaction).

Secondary Endpoints: Secondary endpoints will include laboratory measures of alcohol and cigarette demand (behavioral economic indices of drug motivation) and subjective responses to alcohol during an alcohol challenge procedure, and changes in naturalistic alcohol use and cigarette smoking over the treatment period. Within-person changes in these outcomes before vs. after treatment (0mg placebo lead-in vs. 0.5mg) will be compared as a function of medication condition.

Study Population: Participants who meet criteria for mild or moderate alcohol use disorder/AUD (N=48) and smoke cigarettes daily will be enrolled. Participants will be recruited from the Triangle area (community and hospital settings). Participants must not be seeking current treatment for alcohol or smoking cessation.

Phase: Phase II

Description of Sites/Facilities Enrolling Participants: This is a single-site study (UNC-Chapel Hill). The project will be based at the Bowles Center for Alcohol studies and the Department of Psychiatry, with clinical operations taking place at CTRC.

Description of Study Intervention: Following a 1-week placebo lead-in, semaglutide (Ozempic®) will be administered according to the FDA-approved schedule, which begins with a starting dose of 0.25mg once per week for four weeks. At week five, dosage is increased to 0.5mg/week for a subsequent four weeks (maintenance dose). Following four weeks at the maintenance dose, dosage will be increased to 1.0mg provided the patient has tolerated the medication. Semaglutide is administered subcutaneously via the Ozempic® pen, a cartridge-based device that calibrates medication delivery. The placebo condition will consist of weekly sham doses, which will involve a brief needle insertion (without an injection) using the same micro-needle used in the Ozemic cartridge (NovoFine Plus, 32 gauge).

Study Duration: Up to 13 months.

Participant Duration: Up to 40-50 hours total (including in-person visits and remote questionnaires) over a period of approximately 12 weeks.

1.2 SCHEMA

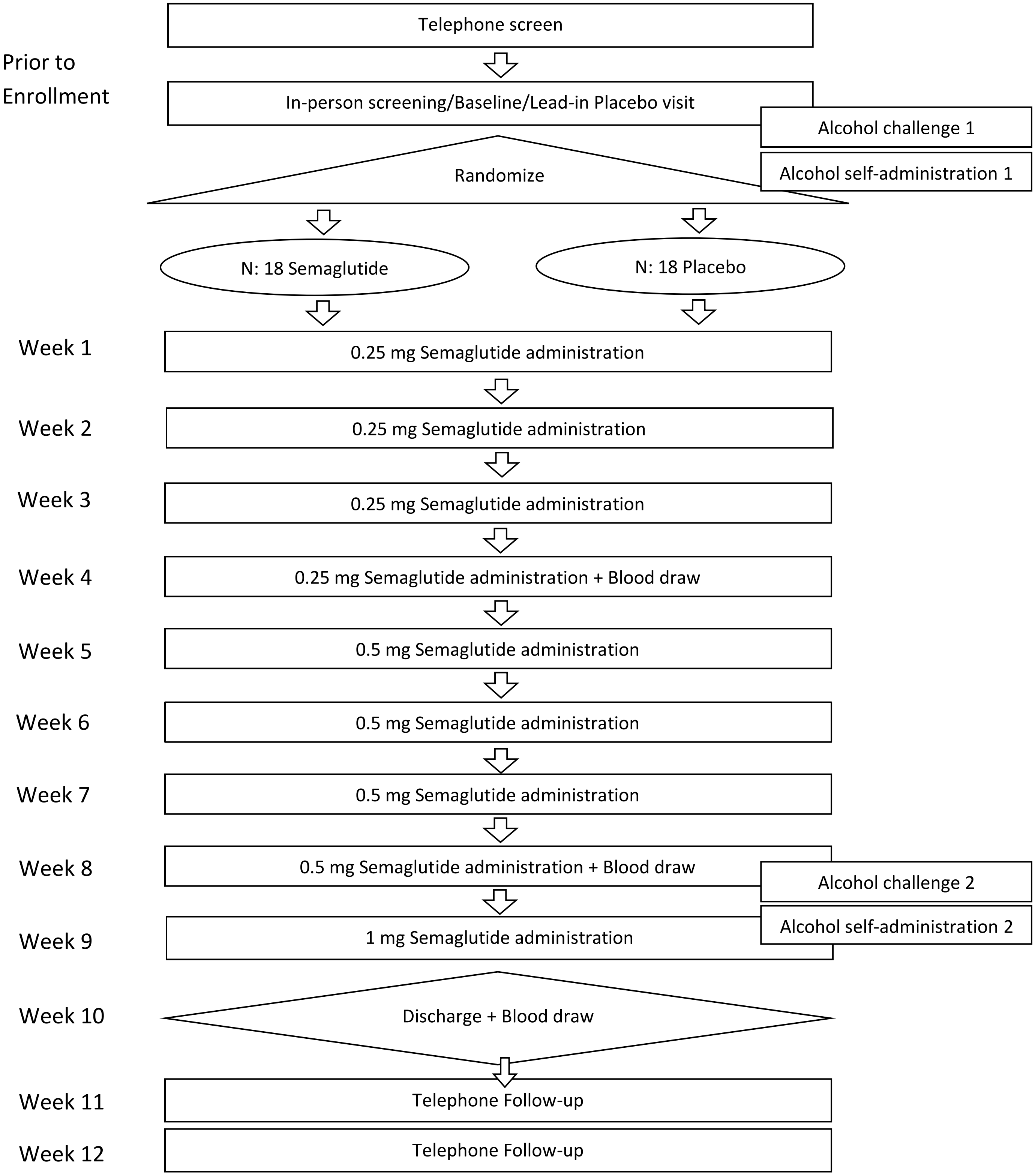
Telephone screen
Prior to Enrollment
In-person screening/Baseline/Lead-in Placebo visit
Alcohol challenge 1
Alcohol self-administration 1
Randomize
N: 18 Semaglutide
N: 18 Placebo
Week 1
0.25 mg Semaglutide administration
Week 2
0.25 mg Semaglutide administration
Week 3
0.25 mg Semaglutide administration
Week 4
0.25 mg Semaglutide administration + Blood draw
Week 5
0.5 mg Semaglutide administration
Week 6
0.5 mg Semaglutide administration
Week 7
0.5 mg Semaglutide administration
Week 8
0.5 mg Semaglutide administration + Blood draw
Alcohol challenge 2
Alcohol self-administration 2
Week 9
1 mg Semaglutide administration
Week 10
Discharge + Blood draw
Week 11
Telephone Follow-up
Week 12
Telephone Follow-up

1.3 SCHEDULE OF ACTIVITIES (SOA)

**Procedures and Questionnaires**	Telephone Screening Day -14 to -7	Screening/Baseline Day -7 to -1	Study Visit 1 - 3 Day 7, 14, 21	Study Visit 4 Day 28	Study Visit 5 - 7 Day 35, 42, 49	Study Visit 8 Day 56	Study Visit 9 Day 63	Discharge Day 70	Telephone follow-up Day 77, 44	Alcohol Challenge Day -10, 58 +/-2	Alcohol Self-Admin. Day -12, 60 +/-2
Informed consent		X									
Demographics	X	X									
Structured Clinical Interview for DSM-5		X									
Randomization		X									
Administer lead-in placebo		X									
Administer 0.25 mg Semaglutide or placebo			X	X							
Administer 0.5 mg Semaglutide or placebo					X	X					
Administer 1.0 mg Semaglutide or placebo							X				
Physical exam (including height and weight)		X									
Breath Alcohol Concentration (BrAC) Test		X	X	X	X	X	X	X		X	X
Urine Drug Screen		X	X	X	X	X	X	X		X	X
Expired carbon monoxide (CO)		X	X	X	X	X	X	X		X	X
Locator		X									
Timeline Follow-Back (TLFB)		X	X	X	X	X	X	X			
Blood Draw		X		X		X		X			
Physical Exam		X									
Medical History Interview		X									
Saliva Collection for DNA and nicotine metabolite		X									
Case Report Forms (CRFs)		X	X	X	X	X	X	X	X	X	X
Alcohol and Cigarette Purchase Test		X	X	X	X	X	X	X			X
Alcohol Use Disorder Identification Test (AUDIT)		X									
Self-Report of the Effects of Alcohol Questionnaire (SRE)		X									
Fagerstrom test for Nicotine Dependence (FTND)		X									
Nicotine Dependence Syndrome Scale		X									
Penn Alcohol Craving Scale (PACS)		X	X	X	X	X	X	X			X
Tiffany Questionnaire of Smoking Urges (TQSU)		X	X	X	X	X	X	X			X
Yale Craving Scale		X	X	X	X	X	X	X			X
Minnesota Nicotine Withdrawal Scale (MNWS)		X									X
Alcohol and Smoking Contemplation Ladder		X									
Three Factor Eating Questionnaire		X	X	X	X	X	X	X			X
Monetary Choice Questionnaire		X									
Cigarette Abstinence Self-Efficacy		X									
Alcohol Abstinence Self-Efficacy		X									
Inventory of Drinking Situations		X									
Impaired Control Scale (ICS)		X									
Barratt Impulsivity Scale (BIS-11)		X									
UPPS Impulsive Behavior Scale (UPPS)		X									
Stop Signal Task											X
Food Delay Discounting		X	X	X	X	X	X	X		X	X
Systematic Assessment for Treatment Emergent Effects (SAFTEE)		X	X	X	X	X	X	X	X	X	X
Vitals (Heart Rate/Blood Pressure)		X	X	X	X	X	X	X		X	X
Finger Prick Glucose Test		X	X		X		X	X		X	X
Weight		X	X	X	X	X	X	X		X	X
Reward Based Eating Drive Scale		X	X	X	X	X	X	X		X	X
Simplified Nutritional Appetite Questionnaire		X	X	X	X	X	X	X		X	X

Procedures and Questionnaires	Telephone Screening Day -14 to -7	Screening/Baseline Day -7 to -1	Study Visit 1 - 3 Day 7, 14, 21	Study Visit 4 Day 28	Study Visit 5 - 7 Day 35, 42, 49	Study Visit 8 Day 56	Study Visit 9 Day 63	Discharge Day 70	Telephone follow-up Day 77, 44	Alcohol Challenge Day -10, 58 +/-2	Alcohol Self-Admin. Day -12, 60 +/-2
Biphasic Alcohol Effects Scale										X	X
Alcohol Urge Questionnaire										X	X
Drug Effects Questionnaire										X	X

2 INTRODUCTION

2.1 STUDY RATIONALE

Pharmacotherapy development is a critical objective for reducing health and societal burdens associated with alcohol use disorder (AUD) [1-3]. Despite advances in this area, medication development remains a slow- moving endeavor with a protracted path from drug discovery to market [2-4]. Given the high degree of heterogeneity in AUD clinical presentation and treatment response, it is recognized that no single medication will be effective for all patients [5]. Therefore, the National Institute on Alcohol Abuse and Alcoholism (NIAAA) medication development strategy specifies the long-term objective of establishing a repertoire of therapies to facilitate targeted interventions for specific AUD subgroups [3, 5, 6]. Strategies for expediting the study of novel treatments are central to these aims [3, 5, 6].

Epidemiological and clinical evidence shows that cigarette smokers comprise a sizable AUD subgroup with disproportionately high health burdens [7, 8] Alcohol and cigarette use are two of the three leading causes of preventable mortality worldwide, jointly accounting for an estimated seven million deaths per year [9]. Population-based research shows that cigarette consumption increases as a function of alcohol use severity [7]. In one large epidemiological study, nearly half (44.6% of men, 47.3% of women) of those with alcohol dependence also met criteria for nicotine dependence [7]. Co-use of alcohol and cigarettes predicts synergistic increases in risk for numerous disease conditions [8, 10, 11], including esophageal and laryngeal cancers [12, 13]. Given these health burdens, increasing effort has been allocated to developing targeted interventions for smokers with AUD [8, 11, 14, 15].

In the absence of FDA-approved medications for concurrent alcohol and nicotine addiction, identifying candidate drugs for this indication is a priority [11, 15, 16]. Several recent studies have examined whether FDA- approved AUD and smoking cessation drugs, either alone or in combination, can facilitate joint reductions in alcohol and cigarette use [11]. For instance, naltrexone reduces alcohol consumption in heavy-drinking smokers [17, 18], and in some cases reduces cigarette consumption [17, 19]. Recent findings further suggest that naltrexone's efficacy for reducing drinking is stronger in AUD participants who smoke relative to non-smokers [20, 21]. However, a Cochrane review [22] and a large, prospective AUD trial [19, 23] concluded that naltrexone does not appear to reduce cigarette smoking [11, 23].

Varenicline has also been tested as a candidate therapy for smokers with heavy drinking or AUD. Informed by early findings that varenicline reduced alcohol self-administration in a human laboratory trial [16], large-scale clinical trials supported efficacy of varenicline for reducing alcohol consumption in heavy-drinking smokers [24, 25] and those with AUD [26]. Recent laboratory-based studies have also

examined the combination of varenicline and naltrexone as a novel treatment [27, 28]. Because those with concurrent alcohol and nicotine addiction reflect a high-priority subgroup, further efforts are needed to screen candidate treatments for this population [8, 11, 28] . As reflected in these recent trials, and as noted explicitly under the NIAAA medication development framework [1-3], drug repurposing strategies can significantly expedite new treatment approaches by capitalizing on therapies that have already passed the extensive regulatory steps required for FDA approval.

Compounds with preclinical evidence for reducing both alcohol and nicotine intake are particularly good candidates for investigating new treatments for heavy-drinking smokers. Accumulating evidence suggests that *Glucagon-like peptide 1* (GLP-1) receptor agonists may have promise in this respect. GLP-1 is an incretin hormone produced and secreted both peripherally (in the intestines) and centrally (in the hindbrain) [29]. GLP-1 is stimulated following food consumption and stimulates satiety and decreases in blood glucose. As a peptide with both hormonal and neurotransmitter functions, GLP-1 plays a role in the "gut-brain axis," which has recently been invoked as a target for addiction therapies [30]. GLP-1 agonist medications were designed to mimic the function of the endogenous GLP-1 peptide, with the benefit of a much longer half-life than endogenous GLP-1. Based on their glucose-lowering and appetite-reducing properties, GLP-1 agonists were developed as anti-diabetic therapies.

Several GLP-1 receptor agonists are now in widespread use as treatments for type II diabetes (T2D), including exenatide, liraglutide, lixisenatide, albiglutide, dulaglutide and semaglutide. These medications have also proven efficacious for promoting weight loss, reducing glycated hemoglobin (HbA1c), and reducing risk for some cardiovascular events [29]. Although the primary indication of GLP-1 agonists is T2D, these therapies are increasingly being studied for other indications in non-diabetic patients, including obesity/weight management.

Of note, GLP-1 agonists require subcutaneous administration, typically by way of a pre-filled medication cartridge with a micro-needle attachment, which allows for easy self-administration in the home without assistance from medical staff. Semaglutide is a recently approved (2017) FDA-approved GLP-1 agonist. A new oral formulation of semaglutide was approved by the FDA in 2019, making semaglutide the first drug in its class to be available in both subcutaneous and oral formulations.

2.2 BACKGROUND

Accumulating evidence from preclinical studies suggests that GLP-1 agonists reduce alcohol and other drug intake and/or reinstatement in animal models. In a recent review of 17 preclinical studies [31], all studies reported significant effects of GLP-1 agonists on indices of drug motivation and intake, with the evidence being most robust for alcohol [30, 31]. Notably, the effects of GLP-1 agonists on drug intake appear to extend to several addictive drugs, including nicotine [32]. These findings have led to recent studies aiming to investigate the efficacy of GLP-1 agonists as candidate therapies for reducing intake or reinstatement of alcohol, nicotine, and other drugs (e.g., cocaine). Although several human trials are underway in various regions, no RCTs have been published to date, and human data on the efficacy of GLP-1 agonists for reducing drug intake in substance-using populations is needed.

Although the precise mechanisms mediating the effects of GLP-1 agonists on alcohol-related behaviors remain to be clarified, it has been suggested that cholinergic-dopaminergic pathways are involved in altering the reward value of alcohol following exposure to GLP-1 agonists [30]. Additionally, GLP-1 agonists appear to exert their effects in part by way of interactions with the serotonin (5-HT) system, including interactions with 5-HT2C receptors. Specifically, GLP-1 agonists appear to induce serotonin

release, and serotonin signaling appears critical to the efficacy of the medications [33, 34]. Some evidence also suggests that 5-HT2C receptors appear necessary for the efficacy and satiety-inducing effects of GLP-1 agonists [35, 36].

Of the approved FDA-approved GLP-1 agonists, most studies have focused on exenatide, an older GLP-1 agonist first approved in 2005. As noted, semaglutide is the newest GLP-1 agonist and the first available in both subcutaneous and oral formulations. Some evidence suggests superiority of semaglutide relative to other GLP-1 agonists with respect to efficacy for glycemic control [37]. Although clinical trials of GLP-1 receptor agonists in participants with substance use disorder are now underway, no studies (to our knowledge) are currently underway to examine semaglutide as a treatment for alcohol use disorder, or for the joint indication of alcohol use disorder and tobacco use disorder. Demonstrating tolerability and initial efficacy of semaglutide for these populations could ultimately allow for greater clinical flexibility if GLP-1 agonists prove efficacious for these populations, given that semaglutide is available in both subcutaneous and oral formulations.

Human laboratory methods for Phase II medication screening

As emphasized in the NIAAA medication development strategy [1, 4, 38], human laboratory methods play a pivotal role in drug development by providing a bridge from preclinical studies and full-scale Phase II/III trials [39-43]. Importantly, laboratory studies can expedite drug development by providing an efficient, cost-effective, and rigorous option for “early-Phase II” medication screening, thereby informing priorities for larger and more costly randomized trials. For example, we recently published meta-analytic evidence that human laboratory studies of naltrexone, when examined in aggregate, yield conclusions highly comparable to the aggregate data from large-scale randomized trials, reinforcing the notion that laboratory trials offer a valid and efficient approach for estimating the potential efficacy of candidate therapeutics [40]. Other advantages of laboratory trials include the ability to study putative treatment mechanisms under controlled conditions, and the ability to quickly generate initial data on drug tolerability in the clinical population of interest [1, 44].

Phase II pharmacotherapy trials for alcohol use disorder often rely on alcohol administration procedures to study medication effects on acute responses to alcohol in human laboratory settings [40]. Controlled alcohol exposure offers a means of testing medication effects on subjective response to alcohol (e.g., stimulation, sedation) and alcohol-induced craving. These subjective responses to alcohol are targets of existing pharmacotherapies, such as naltrexone [40]. Alcohol self-administration has also been used as a laboratory model of alcohol motivation, and has successfully been used to screen candidate AUD therapies [40]. Some have argued that alcohol self-administration should serve as the central endpoint in human laboratory studies, and validated procedures exist for modeling alcohol self-administration safely in the laboratory [16] [40]. More recently, behavioral economic measures have also been utilized as a means of studying drug motivation in laboratory contexts, and appear sensitive for detecting experimental manipulations on alcohol-related motivation. For example, *alcohol demand* indexes the relative reinforcing value of alcohol at a particular point in time [45]. Measures of drug demand, such as the alcohol purchase task (APT) and cigarette purchase task (CPT), are considered objective and sensitive experimental assays of drug motivation [45]. The APT predicts frequency of heavy drinking days after treatment [45], and is sensitive to temporal changes in alcohol’s relative reinforcing efficacy during drug administration [46]. Notably, initial evidence suggests that these measures are sensitive to the effects of pharmacological treatments, including naltrexone and varenicline [47, 48]. Measures of drug demand offer an alternative to studying self-administration in the laboratory, complementing measures of subjective drug responses.

One limitation of many laboratory-based pharmacotherapy trials is the tendency to focus on heavy drinkers without AUD, rather than participants who report symptoms of the disorder [49, 50]. A recent systematic review found evidence that recruiting non-clinical samples for laboratory trials can potentially limit the ability of laboratory findings to generalize to larger clinical trials, perhaps undermining translational research efforts [51]. While ethical considerations normally preclude laboratory alcohol administration methods with treatment-seeking AUD participants [50], studies of *non-treatment-seeking* AUD participants are ethically justified, and can provide important data on medication effects and safety in the target clinical population [52]. Additionally, focusing on participants with *non-severe* (i.e., mild or moderate) AUD can ensure maximal safety when studying this population by eliminating the likelihood of clinically significant withdrawal or alcohol-related medical complications. Our team has ample prior experience with alcohol administration procedures in both clinical and non-clinical populations; we will utilize these methods to study the potential efficacy of semaglutide in participants with mild-to-moderate AUD.

2.3 RISK/BENEFIT ASSESSMENT

2.3.1 KNOWN POTENTIAL RISKS

Semaglutide (Ozempic®®) received FDA approval in 2017 for use as an adjunct to diet and exercise to achieve glycemic control in adults with type 2 diabetes mellitus (T2D). Semaglutide received FDA approval based on the results of 25 completed trials with the goal of providing results that would be applicable to a broad type II diabetes (T2D) population, including the elderly, patients with renal impairment, and patients at high cardiovascular risk. These trials involved > 9,300 participants, of whom >5,700 were treated with semaglutide. In these trials, treatment with semaglutide alone was efficacious for reduction in hemoglobin A1C (HbA1c) below 7% (e.g., 73% of participants had HbA1c <7% at week 30, compared to 28% of placebo-treated patients). Significant body weight reductions were also reported, with the weight loss achieved at end-of-treatment ranging from -1.43 to -4.28 kg (corresponding to 2.3–4.9%). Weight reductions were evident after 4 weeks of treatment, and persisted up to 104 weeks [75]. The peak plasma concentration of semaglutide is reached 1-3 days post-dose. Steady-state exposure is achieved following 4-5 weeks of once-weekly administration, and elimination half-life is approximately 1 week.

<u>Risks related to medication.</u> The most commonly reported side effects/adverse events (AEs) are gastrointestinal, with nausea being the most frequently reported symptom. The majority of reports of nausea (0.5 mg dose vs placebo; 15.3 vs. 6.1%), vomiting (5.0 vs 2.3%), and/or diarrhea (8.5 vs 1.9%) occur shortly following a dose escalation. Abdominal pain (7.3 vs 4.6%) and constipation (5.0 vs 1.5%) were also reported [76]. In studies ranging from 30-56 weeks in duration, the discontinuation rate due to these gastrointestinal AEs was ranged from 5-13%, with this number increasing during longer treatment durations [77].

Rarer AEs (occurring at a frequency of <5%) included symptomatic hypoglycemia (≤70 mg/dL glucose threshold), which occurred in 1.6% of semaglutide-treated participants versus 0% placebo participants; dyspepsia (3.5 vs 1.9%); eructation (2.7 vs 0%); and gastroesophageal reflux disease (1.9 vs 0%). In placebo-controlled trials, injection site reactions (e.g., discomfort, erythema) were reported in only 0.2% of semaglutide-treated patients, suggesting good tolerability of subcutaneous administration. Patients exposed to semaglutide had a mean increase from baseline in amylase of 13% and lipase of 22%.

Cholelithiasis was reported in 1.5% of treated patients. The 0.5 mg dose resulted in a mean increase in heart rate of 2 to 3 beats per minute, while there was a mean decrease in heart rate of 0.3 beats per minute in placebo-treated patients. Hypersensitivity reactions (e.g., anaphylaxis, angioedema) have been reported with GLP-1 receptor agonists. [76]. Other adverse reactions with a frequency of >0.4% associated with semaglutide include fatigue, dysgeusia and dizziness [76].

Rodent studies have shown an increase in the incidence of thyroid C-cell tumors after lifetime exposure of semaglutide at clinically relevant plasma levels. However, several studies in T2D patients taking GLP-1R agonists have shown no increase in neoplasm risk, even after several years of use [78]. Nevertheless, a personal or family history of medullary thyroid cancer or multiple endocrine neoplasia 2A or 2B is considered a potential risk factor for taking the medication, and will be an exclusionary criterion.

In a 2-year trial, acute pancreatitis was observed in 8 semaglutide-treated patients (0.27 cases per 100 patient years) and 10 placebo-treated patients (0.33 cases per 100 patient years). One case of chronic pancreatitis was confirmed in a semaglutide-treated patient. History of pancreatitis will be an exclusion factor, and study participants receiving semaglutide will be observed for signs and symptoms of pancreatitis (via blood monitoring of serum amylase and lipase). If pancreatitis is suspected, semaglutide will be discontinued, and appropriate management recommended.

Other rare events include postmarketing reports of acute kidney injury and worsening of chronic renal failure, which may sometimes require hemodialysis, in patients receiving long-term treatment with GLP-1 receptor agonists. Some of these events have been reported in patients without known underlying renal disease. Most of these events occurred in patients who had experienced nausea, vomiting, diarrhea, or dehydration. Therefore, we will monitor renal function by including a measure of serum creatinine at baseline and at subsequent visits involving blood samples.

Although most safety data on semaglutide comes from trials with T2D participants, there are now a number of randomized trials underway examining GLP-1 receptor agonists in participants with substance use disorder, including trials on tobacco, cocaine, alcohol, cannabis, and opioids. A full list of registered randomized trials can be found by searching for “GLP-1” at ClinicalTrials.gov. This study will be the first to our knowledge to focus specifically on heavy-drinking smokers. The use of a short-term medication screening protocol (lasting roughly 10 weeks) will provide an opportunity to demonstrate tolerability and generate data on side effect profiles in this population while minimizing any risks associated with longer-term medication exposure.

<u>Risks Related to Questionnaire and Interview Assessments.</u> There may be potential risks associated with assessment procedures. Participants may find the battery of psychiatric and psychological assessments tedious or intrusive. Participants may experience discomfort resulting from questions about personal histories or substance use patterns.

<u>Participation in Alcohol Administration Sessions.</u> Other potential risks include typical risks related to participation in alcohol administration studies. For example, it is possible that participants may experience dizziness/nausea from drinking during these visits, or symptoms the following day (e.g., headache). Our group has considerable experience with alcohol administration and self- administration procedures [80-84], and the safety precautions required in these studies. Across these studies, the incidence of adverse events such as vomiting has been exceptionally low. Our procedures will follow the NIAAA recommended guidelines for administering alcohol to human subjects (https://www.niaaa.nih.gov/Resources/ResearchResources/job22.htm). Briefly, the NIAAA guidelines

include recommendations for administering alcohol to participants who meet criteria for AUD. In particular, we have taken measures to protect against ethical concerns by *excluding treatment-seekers* (or those engaged in an active quit attempt), as per the NIAAA recommended guidelines. Additionally, we will screen out participants with severe AUD (defined as 6 or more of 11 symptoms). This approach allows us to generate data relevant to a clinical population while minimizing risks.

Risks Related to DNA Collection. The purpose of collecting saliva for DNA in this study is to allow secondary analyses to examine whether medication effects might be different according to certain genetic factors. These analyses may also focus on genetic factors potentially associated with drug use or related behaviors, such as nicotine and alcohol metabolism, dopaminergic function, or impulsivity. Potential risks associated with DNA collection involve potential identification, and there may be risks with genetic (DNA) tests that are as yet unknown. Genes may be shown at some point in the future to be related to mental illnesses or tendency to addiction. Samples will be labeled with participant ID only, and analyzed at an outside laboratory. Samples will be de-identified and links with participant identifiers will be broken upon completion of the analyses. We will not conduct any whole genome sequencing of the DNA samples. Additionally, the consent form will state that participants have the option to opt out of DNA collection, and to request that their samples are destroyed at any time during the study. Participants will not receive any feedback from any of the genetic tests from study staff.

2.3.2 KNOWN POTENTIAL BENEFITS

The participants in this study are not expected to benefit directly from their participation. However, the knowledge gained from this study could lead to a better understanding of a medication with potential to treat alcohol and nicotine addiction, and potentially other substance use disorders.

2.3.3 ASSESSMENT OF POTENTIAL RISKS AND BENEFITS

Given the vast health implications of substance use disorder and the currently limited array of effective medications, it is imperative to pursue novel pharmacotherapeutic options. A key strategy in this regard is to “repurpose” medications that are already approved (and have proven safe) in the context of other health conditions. Given the sizable amount of research on GLP-1 receptor agonists in other clinical populations, coupled with preclinical evidence that these medications reduce motivation for alcohol, nicotine, and other drugs, evaluating this medication class in the proposed population is warranted. Given no available pharmacotherapies for the indication of concurrent alcohol and nicotine addiction, any evidence for potential efficacy in heavy-drinking smokers could have substantial implications. From this standpoint, the risk/benefit profile for testing semaglutide in a population of non-diabetic participants is favorable.

Although we anticipate the risk of adverse events to be low based on the safety profile of the medication, gradual dose escalation, and relatively short duration of treatment, several steps will be taken to track and report medication- related side effects. As described further in later sections of this application, participants will be monitored regularly by virtue of attending weekly lab visits for medication delivery, at which side effects will be assessed and clinical lab results monitored. Participants will be given a contact number for study medical staff in case of serious emergent side effects, and participants will be contacted by phone shortly after dose escalations to monitor symptoms. We expect

some participants to experience nausea shortly following dose escalations, but that these symptoms will usually be transient.

Given the focus on a non-obese population, we will be particularly vigilant in monitoring changes in body weight and blood glucose, and will exclude participants at the outset with body weight or blood glucose levels below normal range. We will carefully monitor participants with glucose tests and weight measurements each week, and will instruct participants to be vigilant for potential symptoms of hypoglycemia. Periodic blood tests to measure fasting glucose, fasting insulin, serum lipase and amylase, serum creatinine, and serum calcitonin will occur at baseline and regularly thereafter (i.e., at visits prior to dose escalations). All adverse events will be documented and reported in accordance with relevant policies, and the regulations of NIH. A DSMB will be formed at the outset of the study to review safety data at scheduled intervals. The DSMB will include a physician with expertise in GLP-1 medications, and 2 additional members who are not in conflict with the members of the investigative team. This board will meet periodically to review data quality, recruitment and retention, and to review all serious or clinically significant adverse events. In addition, the board will review safety data following any serious adverse event that appears to be study related. Patterns of adverse events as well as individual events may indicate the need for operational changes, protocol modifications, a decrease in dose, or, conceivably, discontinuation of the trial.

Considering the use of an FDA approved medication that has been deemed safe in the context of chronic administration for glycemic control, the risks related to medication use in this short-term screening trial are acceptable in comparison to the potential benefits of the proposed research (i.e., demonstrating potential efficacy of a new medication for substance use disorder).

Risks Related to Alcohol Administration Sessions. Alcohol administration procedures are commonly used to study risk factors for alcohol use disorder (AUD), and have proven capable of identifying successful pharmacotherapies [40]. As per the NIAAA guidelines for administration of alcohol in human studies, such studies are typically restricted to non-treatment-seeking participants in order to minimize ethical concerns, as will be the approach in this study. Further details on safety procedures are provided later in this document.

3 OBJECTIVES AND ENDPOINTS

OBJECTIVES	ENDPOINTS	JUSTIFICATION FOR ENDPOINTS
Primary		
To examine effects of semaglutide vs. placebo on alcohol self-administration under laboratory conditions.	Amount of alcohol consumed and breath alcohol concentration (co-primary endpoints) during a validated laboratory alcohol self-administration protocol. In this procedure, participants are provided the option of choosing between drinks and monetary reward. The comparison of interest is the difference between medication and placebo groups in the change in	Primary: This outcome is a laboratory assay of alcohol intake, and has been used in prior human laboratory studies to screen candidate medications, allowing comparisons with effect sizes for other medications. This session will be conducted during the standard maintenance dose (0.5mg) to mimic the common clinical scenario.<br><br>Secondary: Subjective alcohol responses and alcohol/cigarette demand are validated measures of

OBJECTIVES	ENDPOINTS	JUSTIFICATION FOR ENDPOINTS
	laboratory consumption between baseline (placebo lead-in) and the maintenance dose of 0.5mg.	drug reward/motivation, and are known to be sensitive to medication effects. Given substantial individual differences in alcohol pharmacokinetics, a within-subjects analysis is preferable for these endpoints (such that each participant will serve as their own control). Laboratory sessions will take place at two time points: placebo lead-in and 0.5mg maintenance dose.
Secondary		
To examine group differences in 1) alcohol/cigarette demand and subjective responses to alcohol during alcohol administration, and 2) naturalistic drinking and smoking behaviors over the course of the 10-week treatment period.	Laboratory measures of subjective responses to alcohol, alcohol demand, and cigarette demand during controlled laboratory alcohol administration (lab sessions will take place during the placebo lead-in and again after achieving the maintenance dose).<br><br>Number of standard drinks consumed and number of cigarettes consumed per day during the treatment phase (Weeks 0-10), as measured by a daily cellphone survey.<br><br>Similar to the primary outcome, secondary outcome analyses will examine medication vs. placebo group differences in within-subjects changes in these outcomes over time (0mg to 0.5mg).	Laboratory measures of alcohol response and alcohol/cigarette demand are clinically valid indicators of drug motivation, and are demonstrated to be sensitive to medication effects. Investigating these outcomes under controlled conditions will allow us to test for a medication "signal" on measures of alcohol and nicotine motivation.<br><br>Daily measures of alcohol consumption (standard drinks per day) and cigarettes per day are selected to afford greater power for modeling change in consumption over the treatment period. Note that these outcomes are considered secondary because this Phase II trial involves non-treatment-seeking participants who are not attempting to reduce alcohol or use or cigarette smoking.
Tertiary/Exploratory		
Body weight, HbA1c, alcohol elimination.	Changes in body weight, HbA1c, and alcohol elimination will be examined as exploratory endpoints based on potential clinical relevance.	GLP-1 receptor agonists lower HbA1c and body weight in indicated populations (e.g., Type II diabetes, obesity). Given high co-occurrence of obesity and alcohol use disorder, reporting whether medication is associated with these reductions in the present sample is clinically relevant. Data from the alcohol administration sessions will be used to estimate whether medication

OBJECTIVES	ENDPOINTS	JUSTIFICATION FOR ENDPOINTS
		predicts changes in alcohol elimination during treatment, and whether changes in body weight account for any such differences. These outcomes are exploratory.
All secondary and teriary outcomes will also be investigated		

4 STUDY DESIGN

4.1 OVERALL DESIGN

Overview of Study Design.

The aim of this protocol is to translate preclinical findings on GLP-1 agonists by conducting an early-Phase II investigation of semaglutide (Ozempic®) in non-treatment-seeking participants with AUD, with a focus on human laboratory outcomes. Given preclinical evidence for the potential efficacy of GLP-1 agonists for reducing both alcohol and nicotine intake, and the need for treatments targeting both alcohol and nicotine addiction, this proposal focuses on cigarette smokers with AUD.

This is a single-site, Phase II, two-arm, double-blind (participants, outcome assessors), randomized, placebo-controlled, dose-ranging study. Following a baseline assessment and one-week placebo lead-in period, eligible participants will be randomized to medication or placebo conditions by the IDS staff at a 1:1 ratio. The treatment period will be approximately 10 weeks, taking into account the dose escalation necessary for this medication. Following the placebo lead-in (administered at baseline/Week 0), those randomized to semaglutide will receive 0.25mg/week for 4 weeks (Weeks 1-4), and 0.5mg/week for 4 weeks (Weeks 5-9). Provided that participants have tolerated the medication adequately, participants will receive a final dose of 1.0mg (Week 10), and will be monitored for the subsequent three weeks. Those in the placebo condition will receive sham placebo administration at each visit (described in further detail in later sections). Participants will attend weekly visits at CTRC to receive medication or placebo doses.

In addition to the medication visits, participants will be asked to attend four laboratory visits: two involving alcohol self-administration (one during the placebo lead-in week and one during the 0.5mg treatment phase) and two involving alcohol challenge (one during the placebo lead-in week and one during the 0.5mg treatment phase). These sessions are designed to assess changes in alcohol responses over the course of the treatment period. Laboratory sessions are described in further detail below. Measurements of naturalistic alcohol and cigarette use will also be recorded throughout the treatment period. These assessments will be captured using brief questionnaires administered daily via cell phone. Additional self-report measures of substance use will be collected at study visits.

As summarized above, the primary objectives are to examine whether treatment with semaglutide alters alcohol self-administration (primary outcome) and laboratory measures of alcohol response and motivation (i.e., demand and responses to alcohol, secondary outcomes) during alcohol administration.

We hypothesize that the semaglutide treatment (versus placebo) will be associated with reduced alcohol self-administration during a laboratory session conducted at the maintenance dose of 0.5mg. We further hypothesize that semaglutide will lead to reductions in alcohol demand, cigarette demand, and subjective responses to alcohol (e.g., reduced stimulation) during alcohol administration sessions.

The primary and secondary outcomes for this trial will be assessed during exposure to the standard maintenance dose (.5mg). While an extended treatment phase at the 1.0mg dose is beyond the scope of this small-scale trial, the inclusion of a brief (1-week) phase at 1.0mg is designed to provide initial data on tolerability and any changes on selected secondary outcomes (i.e., drinks per day and cigarettes per day) in this population. Capturing these data will help to support feasibility and pilot data for subsequent funding proposals with longer treatment intervals.

Secondary objectives include examining changes in naturalistic alcohol consumption and cigarette smoking during the treatment period. Although participants will not be attempting to reduce drinking or smoking in this trial, it is hypothesized that treatment with semaglutide will result in reductions in alcohol consumption (drinks per day) and cigarettes per day over the course of the treatment period. Tertiary/exploratory outcomes include examining changes in body weight, HbA1c, and alcohol elimination during the trial.

In total, participation in this study is estimated to take a maximum of up to 50 hours of combined time over a period of approximately 11-12 weeks beginning with initial screening. This includes a screening and baseline visit (up to 4 hours), up to 9 subsequent laboratory visits for medication administration and blood glucose monitoring (up to 1 hour each), and a final discharge visit (Week 11). Participants will also complete four visits involving alcohol administration over the course of Weeks 0-10; these sessions may last up to 7-8 hours each. Participants also will receive calls from staff 2-3 days after each medication phase begins (placebo, 0.25mg, 0.5mg, 1.0mg) to check in on side effects (10 minutes per call), and will be asked to complete brief daily questionnaires via text message prompt (1-2 minutes per day).

A detailed summary of study procedures is provided below.

Detailed Study Procedures.

Following initial recruitment (described in section 5.5), participants will be asked to complete the following procedures:

a) Telephone screen

Initial screening will be conducted by telephone by trained research staff and will assess participant alcohol, cigarette, and drug use, medications, and basic medical inclusion/exclusion factors. Potential participants will be given a brief description of the study. The initial telephone screen is estimated to take roughly 15-30 minutes. Contact information will be collected from interested callers who pass the initial screen. Following the screen, research staff will verify any medical questions (e.g., medication contraindications) with the study physicians if necessary. Participants will then be scheduled for the in-person screening, consent and baseline visit (Week 0).

b) Screening and Baseline Data Collection Visits

The in-person screening/baseline visit will include informed consent, a clinical screening session, self-report and interview questions, a baseline blood sample and weight measurement, a baseline saliva sample for DNA analysis, and (if preliminary eligibility criteria are satisfied)

administration of the lead-in placebo dose. These procedures will be conducted by the research assistant and/or NC TraCS staff member, and may be separated into two visits depending on staffing availability. Participants will be instructed to arrive in the morning. Participants will be instructed to refrain from alcohol use starting the night before their scheduled in- person medical screening visit, and to arrive fasting (to allow for assessment of baseline HbA1c and lipids).

Upon arrival the study team member will direct the participant through informed consent procedures. Next, participants will complete a breathalyzer and urine drug screen (for females, the test will include a pregnancy screen), and then briefly review and verify information collected over the phone. Participants who have a positive BAC (greater than 0.00) will be monitored until their BAC is reduced to a safe level (BAC<.03g%), in accordance with NIAAA guidelines (or to 0.00g% in the event that the participant drove to the session). Participants will complete an interview with a research assistant to assess diagnostic and substance use screening criteria, including symptoms of alcohol use disorder and aspects of medical history (see criteria listed above), via interview and self-report questionnaires. The Structured Clinical Interview for DSM-V [53] will be used to assess AUD symptoms. History of other psychiatric diagnoses and medication use will be assessed via self-report. The Timeline Follow-Back (TLFB) [54] will be used to assess alcohol, cigarette and other drug use in the 90 days prior.

Participants who remain eligible after the interview will complete vitals measurements (HR, BP), and a measure of expired alveolar carbon monoxide (CO). Additionally, participants will complete a blood draw (up to 20 ml) for a basic metabolic and lipids panel (fasting), baseline measure of HbA1c, baseline measure of nicotine metabolites (e.g., 3HC, COT), serum amylase and lipase, and hepatic function panels. (If participants are not able to arrive fasting for the baseline session, the fasting lipids sample may be deferred or completed at the next possible visit.) Blood draws will be performed by trained CTRC staff and samples will be sent to a local laboratory for testing. The urine sample will be used to test for the presence of several drugs (for example, heroin, cocaine, methamphetamines, opiates) and will be stored for future analysis of nicotine metabolites (e.g., 3HC, COT). If a participant tests positive for drugs other than cannabis (or if females screen positive for pregnancy) the participant will be excluded from the study.

During the medical history assessment, any medications that the participant is taking will be recorded for subsequent review by the study physician. Potential interactions with semaglutide will be verified using an established online database such as Epocrates or Lexicomp. In the event of any uncertainties about medication interactions, participants will be asked to contact their primary care physician to confirm no concerns with taking semaglutide. A medical staff member (RN or MD) will review the medication with the participant. Participants will be advised to be vigilant for symptoms of hypoglycemia at the medical visit, and instructed to contact the study team to review any symptoms that emerge (finger-prick glucose tests will also be obtained at all follow-up visits). If necessary, the medication review will take place via telephone or telemedicine, depending on medical team availability.

Participants will complete baseline questionnaires, which will be programmed in RedCap software. A Demographic Screening Questionnaire will assess basic demographic factors (e.g., age, education, income). A Locator form will collect contact information for three individuals who can be contacted if a participant is unable to be reached by study staff for follow-up visits. Other baseline measures (to be administered by computer using RedCap software) will include

the Alcohol Use Disorders Identification Test [55], Self-Report of the Effects of Alcohol Questionnaire (SRE) [56, 57], Fagerström Test of Nicotine Dependence [58], and the Nicotine Dependence Syndrome Scale [59], Barratt Impulsivity Scale (BIS-11) [60], UPPS Impulsive Behavior Scale [61], and the Impaired Control Scale (ICS) [62]. Baseline measures that will be repeated at subsequent visits will include the Timeline Followback, the Penn Alcohol Craving Scale (PACS) and Tiffany Questionnaire of Smoking Urges (TQSU) [63], the Yale Craving Scale, and the Minnesota Nicotine Withdrawal Scale [64]. To assess potential medication effects on changes in motivation and self-efficacy for resisting alcohol/cigarettes, we will use alcohol and smoking Contemplation Ladder items, alcohol and cigarette Abstinence Self-Efficacy (AASE and SEQ) (these scales will be repeated at each visit) and the Inventory of Drinking Situations scales [65-67]. Appetite and eating behavior will be assessed with the Control of Eating Questionnaire, the Three Factor Eating Questionnaire [68] and the Reward-Based Eating Drive Scale [69]. During the medication phases, side effects will be assessed with the SAFTEE questionnaire [70], a standard tool for alcohol clinical trials, and an Adverse Events Checklist [71]. Participants will also complete previously validated Purchase Tasks for alcohol, cigarettes, and food. These brief behavioral economic tasks assess perceived value of reinforcers by presenting hypothetical scenarios involving choices between the reinforcer (alcohol, cigarette, food) and monetary rewards; these measures will be completed at each visit.

The baseline questionnaires and interview assessments are projected to take approximately 3-4 hours combined. Patients will be given breaks as needed. Following the blood draw, participants will be offered a snack and bottled water (given the instructions to arrive fasting). If results of the baseline screening/medical assessment indicate that the participant is tentatively eligible, they will be given a comprehensive orientation to the next phases of study, including the follow-up medication visits, the laboratory alcohol administration visits, and the daily surveys. Locator/collateral contact information will also be obtained.

Before leaving, participants will be given the placebo lead-in “dose” of the medication (justification for this approach is described in the medication/intervention section of this application). The 1-week placebo lead-in also provides time to review lab results and exclude participants (if needed) prior to administering the study medication. Lab results will be uploaded to the EMR and reviewed by the study physician prior to prescribing the medication. Additionally, the study physician may meet with the participant via telemedicine between the baseline visit and the subsequent visit (Week 1), if indicated to clarify medical questions or discuss test results.

c) <u>Daily Assessments.</u>

Throughout the study, participants will be asked to complete a brief remote assessment each morning using REDCap (an automated link will be texted to the participant each morning). The brief questions will include measures of 1) prior-day number drinks consumed; 2) number of cigarettes smoked; and 3) prior day peak craving for alcohol and cigarettes. Participants will be texted a link to the survey, which can be completed via smartphone. If preferred, participants can opt to receive an email link. At each study visit, a staff member will provide compensation for the prior week’s surveys, and will review survey adherence and progress towards compensation for good adherence (described later), as a means of motivating adherence to self-report assessments.

d) <u>Weekly Laboratory Visits.</u>

Following the baseline (Week 0) visit and initial placebo lead-in dose, participants will be asked to report back to the CTRC once weekly (Weeks 1-9), for subsequent medication or placebo doses, and for a final follow-up/discharge visit at Week 10. In addition to medication/placebo administration, all CTRC visits will include 1) vitals, weight measurements and a brief side-effects assessment, 2) a finger-stick glucose test (or fasting blood draw, depending on the week, see study flow chart), 3) a brief calendar-based self-report assessment of interim alcohol and cigarette use, and 4) a repeat CO measurement. These procedures are expected to take roughly 30 minutes in total, and will be conducted by a trained study team member and/or a member of the CTRC team (e.g., RN). All side effects reports, labs, and other medical test results (e.g., glucose tests) will be uploaded for review by the study physician. If side effects or lab results suggest that further assessment is indicated, a member of the medical team advise the study team (and if necessary, contact the participant to provide direction). At the Week 4, Week 8, and final discharge visits, participants will be asked to arrive fasting to allow for a blood draw for follow-up lab assays (identical to those described in the baseline procedures above).

e) Alcohol Sessions.

To evaluate effects of medication phase on laboratory measures of alcohol response and self-administration, participants will be asked to complete four laboratory visits over the course of the study. Two visits will occur between the Week 0 and 1 visits (placebo lead-in phase), and two will occur between the Week 8 and 9 visits (0.5 mg phase). Minor scheduling deviations will be accommodated as needed, provided that the participant has completed at least three weeks of the maintenance dose (.5mg) phase at the time of the second set of laboratory sessions.

During each of these sessions participants will be asked to report to the lab in the morning, having received instructions to abstain from alcohol and recreational drugs for 24h, and not to consume food for 4 hours prior to arrival. Participants will also be advised not to drive to these study appointments. Participants will also be asked to schedule a smoking break just prior to arrival (to ensure that participants are not nicotine-deprived during testing) [16]. Upon arrival participants will provide breathalyzer and expired CO readings, as well as measures of body weight and vitals (HR, BP) and a finger-stick blood glucose test. Female participants will also be asked to repeat the urine pregnancy screen at each alcohol session.

Participants will consume a standardized, isocaloric meal in advance of the alcohol administration procedures, with a standardized wait time (60 min) between completion of the meal and onset of beverage consumption. In the interim, participants will complete a baseline measure of inhibitory control (stop signal task) [72], and self-report questionnaires (e.g., Timeline Follow-Back to assess recent substance use). Participants will be seated in a comfortable recliner chair for the alcohol administration procedures. Participants will first complete baseline physiological readings (systolic and diastolic blood pressure, heart rate), and baseline measurements of self-report questionnaires (see below). Next, participants will complete one of two procedures (alcohol challenge, alcohol self-administration), dependent on the visit week. Specifically, the first alcohol challenge session (AC1) and the first self-administration session (SA1) will be scheduled to take place between Week 0 and 1 (placebo lead-in phase). The second alcohol challenge session (AC2) and the first self-administration session (SA2) will be scheduled to take place between Week 8 and 9 (0.5mg treatment phase), allowing for minor scheduling adjustments as noted above. The two session types will be scheduled in a fixed order (AC followed by SA), with at least 24 hours between sessions.

Alcohol Challenge Sessions (AC1, AC2)
The AC sessions are designed to generate data to estimate within-person changes in acute responses to alcohol as a function of medication phase (placebo lead-in vs. 0.5mg). Following baseline assessments (described above), participants will be asked to consume a pre-determined dose of alcohol calculated to achieve a target peak breath alcohol concentration (BrAC) of .06g%. This BrAC level reflects a moderate alcohol dose. Individualized doses will be calculated using established formulas [73] that account for determinants of total body water (e.g., height, weight, sex, age). Beverages will consist of 80-proof vodka combined with a non-alcoholic, sugar-free mixer at a 1:5 ratio. Beverages will be allocated to three equal portions in separate cups. Participants will be asked to consume the beverages at an even pace over 10 minutes. A separate placebo session is not incorporated for the AC sessions, because our primary interest is in examining *within-person* changes in laboratory alcohol responses as a function of medication dose (0mg/placebo lead-in vs. 0.5mg), and because our primary outcome is self-administration (described below). Although a limitation of this approach is the inability to dissociate expectancy from pharmacological effects in measuring subjective responses to alcohol, increasing the number of sessions to dissociate expectancy effects is not a major aim for this preliminary project.

After completion of the beverages, participants will wait 10 minutes (to allow an absorption period), then will complete an initial breath alcohol concentration (BrAC) reading. BrAC readings will be repeated at 10 minute intervals during the first hour of the session (peak BrAC is anticipated to occur around 30-40 minutes, on average, with variability across subjects). Participants will repeat the battery of self-report questionnaires (outlined below) and physiological assessments at three points on the ascending limb of the blood alcohol curve: upon the first BrAC reading that meets or surpasses .02g%, .04g%, .06g%. To account for the fact that some participants may not reach the .06g% target, the latter assessment will be conducted if/when it appears the participant will not reach the .06g% target (as evidenced by two consecutive descending BrAC readings after surpassing the .05g% mark).

Following completion of the last set of measures (.06g%), participants will be allowed to take a bathroom break. Participants will then be escorted to a private room to begin the recovery phase, during which they will receive water and will be monitored. During this time BrAC measurements will continue every 15 minutes. On the descending limb of the blood alcohol concentration curve, participants will complete follow-up measures and physiological readings at the first BrAC readings that meet or fall below .04g% and .02g%. After the first descending assessment (.04g%), participants will be asked to provide an additional blood glucose sample (via fingerpick) to assess any differential changes in glucose levels following alcohol administration as a function of medication phase. Participants will also complete the Food Purchase Task to assess motivation for food following alcohol administration. After this point, participants will be provided with a lunch and snacks to consume during the recovery period. Participants will be compensated and discharged once BrAC reading reaches .00g% (to allow for estimating alcohol elimination rate across sessions AC1 and AC2 as a function of medication group).

<u>Subjective Measures.</u> At baseline (prior to alcohol ingestion) and each subsequent assessment point noted above, participants will complete several measures. Measures of subjective alcohol effects will include the Biphasic Alcohol Effects Scale (BAES) [74], the Alcohol Urge Questionnaire (AUQ) [75] and Drug Effects Questionnaire [76]; the latter will be supplemented with additional items to assess potential effects related to medication (e.g., “tired”, “confused”, “nauseous”, “dizzy”, “sick”, “drunk/intoxicated”) [16]. The Alcohol Purchase Task [45] and Cigarette Purchase Task [77] will be

also administered at the corresponding intervals noted above. During the recovery period, participants will complete another battery (all measures noted above) once reaching .04g% and .02g% on the descending slope of the blood alcohol curve.

Alcohol Self-Administration Sessions (SA1, SA2).

Medication effects on laboratory alcohol consumption will be examined using a validated oral self-administration procedure, which has been established as sensitive to medication effects. Pre-session instructions and arrival procedures/tests will be identical to those noted above. After completing baseline physiological and baseline questionnaires, the procedure will commence with a *priming drink*, calculated to achieve a target breath alcohol concentration (BrAC) of .03g%. In this procedure, a priming drink is administered as a means of prompting subsequent self-administration. The priming drink will include 1 part 80-proof liquor (of the participant's choice) to 4 parts mixer, and will be consumed over 5 minutes, followed by a 30-minute absorption period. At the end of the absorption period participants will complete the second round of self-report questionnaires (see Measures), as well as physiological and BrAC readings.

After the post-priming assessments, participants will complete a 2-hour *ad libitum* self-administration period, separated into two 1-hour blocks. During each block, participants are allowed to consume up to 4 “mini-drinks”, each calculated (based on the person’s sex, weight, height, and age) to achieve an average additional BrAC increment of .015g% per drink using established formulas. Beverages will be presented to the participant at onset of each block, and the remaining amount will be removed and weighed at the conclusion of each block. Consistent with prior studies using this protocol, participants will have the choice of receiving monetary reinforcement (at the end of the session) for drinks not consumed ($3 per beverage), which provides a competing incentive against consumption. Participants will repeat additional rounds of self-report assessments (described above) at 30-minute intervals during the *ad libitum* phase (i.e., at 60, 90, and 120 min. after the end of the priming phase), with physiological assessments and BrAC readings taken at these intervals. The primary outcomes for this session are peak BrAC and volume of alcohol consumed.

Participants will then be escorted to a private room for the recovery period. Assessments during the recovery period will be identical to those described above. Participants will be monitored until being discharged when BrAC declines below .03% for two consecutive readings, consistent with NIAAA recommended guidelines. [52] As noted, participants will be instructed not to drive, and will be asked to arrange for a ride home from the study site. If a participant cannot arrange a ride home, they will be provided with transportation home via taxi or Uber. If a participant reports having driven to a session, they will be required to remain at the site until BrAC is measured at 0mg% for two consecutive readings.

<u>List of Study Assessment Measures (See Section 1.3 for assessment time points)</u>

1) Demographics form
2) Timeline Follow-Back (TLFB)
3) Structured Clinical Interview for DSM-5 Mental Disorders (select modules)
4) MINI Neuropsychiatric Interview (select modules)
5) Alcohol Use Disorder Identification Test (AUDIT)
6) Self-Report of the Effects of Alcohol Questionnaire (SRE)
7) Barratt Impulsivity Scale (BIS-11)
8) UPPS Impulsive Behavior Scale (UPPS)
9) Impaired Control Scale (ICS)
10) Stop signal task
11) Fagerstrom Test for Nicotine Dependence (FTND)
12) Nicotine Dependence Syndrome Scale

13) Penn Alcohol Craving Scale (PACS)
14) Tiffany Questionnaire of Smoking Urges (TQSU)
15) Yale Craving Scale
16) Minnesota Nicotine Withdrawal Scale (MNWS)
17) Alcohol and Smoking Contemplation Ladder
18) Three Factor Eating Questionnaire
19) Reward Based Eating Drive Scale
20) Control of Eating Questionnaire (COEQ)
21) Simplified Nutritional Appetite Questionnaire
22) Monetary Choice Questionnaire
23) Cigarette Abstinence Self-Efficacy
24) Alcohol Abstinence Self-Efficacy
25) Inventory of Drinking Situations
26) Biphasic Alcohol Effects Scale (BAES)
27) Alcohol Urge Questionnaire (AUQ)
28) Drug Effects Questionnaire (DEQ)
29) Alcohol Purchase Task
30) Cigarette Purchase Task
31) Food Purchase Task

Analysis schedule. A planned interim analysis of primary and secondary outcomes will be conducted once half of the target sample is reached. Analyses of side effect frequency and AE/SAE frequency will be conducted prior to scheduled DSMB meetings. Other analyses will be conducted following the completion of the trial.

4.2 SCIENTIFIC RATIONALE FOR STUDY DESIGN

This short-term trial is intended to produce initial data on the effects of semaglutide in non-treatment-seeking participants with AUD, with an emphasis on human laboratory outcomes. The dose escalation schedule is based on the standard clinical regimen, which is intended to minimize side effects common to GLP-1 agonists (i.e., nausea). Participants in the placebo group will complete an identical sequence while receiving sham subcutaneous injections (a small needle stick using a micro-needle of the same size, without an injection). This placebo condition is selected due to the absence of an available matching placebo cartridge from the manufacturer (see section 4.3). All participants will complete laboratory alcohol administration sessions at two time points, corresponding with the baseline (placebo lead-in) and 0.5mg, phases. This design allow for within-subjects analyses for laboratory outcomes that are best examined at the within-subject level (owing to high inter-individual variability in alcohol pharmacokinetics).

Additionally, all participants will receive one sham injection as the first medication “dose,” which allows for a 1-week placebo lead-in period. The one-week placebo lead-in phase serves three purposes. First, it is known that participation in pharmacotherapy trials leads to reductions in alcohol consumption independent of pharmacotherapy effects. A placebo lead-in phase allows a period for medication-nonspecific reductions in drinking (e.g., due to assessment reactivity or “placebo response”) to manifest prior to initiating active medication. Second, a lead-in period provides a baseline with which to examine within-person changes in alcohol-induced subjective responses, craving, and alcohol demand in the laboratory – as well as within-person trajectories of alcohol and cigarette use outside the laboratory - as

a function of increasing dosage. Third, this lead-in phase allows an opportunity for early non-adherent participants or "drop-outs" to be identified prior to administering the study medication, which is advantageous for this modest-sized trial involving a relatively costly drug. Overall, this design is intended to maximize feasibility and sample size while also accommodating the extended dosage titration period required of GLP-1 agonists. Findings from this trial will be used to justify a larger, placebo-controlled Phase II clinical trial application via the R01 mechanism.

4.3 JUSTIFICATION FOR DOSE

Semaglutide (Ozempic®) will be administered according to the FDA-approved schedule, which begins with a starting dose of 0.25mg (for titration purposes) once per week for four weeks. At week five, dosage is increased to 0.5mg/week (the initial first maintenance dose) for a subsequent four weeks, at which point dosage can be increased further (1.0mg) if indicated (and presuming that the 0.5mg dose has been tolerated). The intent for this initial trial is to mimic the typical clinical scenario over the first several weeks of medication titration. Although primary analyses will focus on the 0.5mg dosage, participants will receive one week of 1.0mg treatment at the conclusion of the 0.5mg phase, in order to collect initial data on side effects and naturalistic drinking/cigarette use at this dosage in the current population. Of note, recent large-scale efficacy trials of semaglutide for the indication of obesity have utilized long-term dosing protocols with substantially higher doses (up to 2.4 mg/week), and the FDA has now (June, 2021) approved this dosage for weight management (under the brand name Wegoby). Participants will be monitored carefully and will not be escalated to the next dose if experiencing persistent side effects indicative of poor tolerability.

Given the lack of a true placebo (i.e., no manufacturer-developed placebo Ozempic® pen/cartridge), this study will use a placebo condition that consists of weekly sham doses. Sham doses will involve a brief needle prick using a very small (e.g., 32 gauge) needle, using the NovoFine proprietary needle (as is used in the Ozempic® pen). Additionally, all participants will receive one placebo injection at the first study visit (lead-in placebo phase). These procedures are described in more detail later in this protocol. By necessity, medical staff administering the medication at CTRC cannot be blind to condition. However, other experimenters and outcomes assessors will remain blind to condition. Participants will be instructed during the informed consent and study orientation that they have a 50/50 chance of being randomized to medication and placebo groups.

4.4 END OF STUDY DEFINITION

A participant is considered to have completed the study if he or she has completed all phases of the study including the last visit or the last scheduled procedure shown in the Schedule of Activities (SoA), Section 1.3. The end of the study is defined as completion of the last visit or procedure shown in the SoA in the trial overall.

5 STUDY POPULATION

5.1 INCLUSION CRITERIA

In order to be eligible to participate in this study, an individual must meet the following criteria:

- Age 21-65

- Meeting DSM-5 criteria for current (past year) mild or moderate AUD (i.e., from 2-5 symptoms endorsed), and NIAAA criteria for current at-risk drinking (i.e., >7/14 drinks per week for women/men, on average, with at least two episodes of 4+/5+ drinks in the past 30 days)
- Daily smoker, defined as reporting smoking 1+ cigarettes per day, on average, over the past 12 months (past year avg cigarettes per day ≥ 1) and daily/near-daily smoking in the past month (smoking at least 25 days in the past 30)
- Willingness/availability to take study medication and complete study procedures, including attending weekly visits for medication administration, side effect assessments, and glucose monitoring
- Willingness to complete laboratory sessions involving alcohol administration
- Ability to communicate and read in English

5.2 EXCLUSION CRITERIA

An individual who meets any of the following criteria will be excluded from participation in this study:

- Recent (30 day) illicit drug use (with the exception of cannabis) based on self-report, or positive reading on a baseline toxicology screen
- Meeting DSM-5 criteria for severe alcohol use disorder, or another past-year substance use disorder (with the exception of tobacco use disorder or mild cannabis use disorder)
- Reported history of severe withdrawal symptoms (e.g., seizures, DTs)
- Past 30-day use of nicotine replacement therapies/products
- Past 30-day use of: Sincalide, Sulfonylureas, or other medications that may interact with semaglutide; psychiatric medications, or weight control medications,
- Lifetime diagnosis of severe mental illness (e.g., bipolar I or psychotic disorder)
- Current significant medical or neurological illness based on medical staff (i.e., physician or nurse practitioner) evaluation, including severe hepatic impairment or cirrhosis, impaired renal function (eGFR <50ml/min), pancreatitis, gastroparesis, gall bladder disease and cholelithiasis, other severe gastrointestinal disease, heart failure, coronary artery disease, stroke, or other medical condition that poses a risk for the medication or alcohol administration components of the study (as determined by the QI)
- Liver function values AST or ALT greater than four times the normal limit, or elevated alkaline phosphatase or bilirubin.
- History of Type 1/ insulin-dependent diabetes (Type II diabetes without insulin use will be permitted if other criteria are satisfied)
- Past history of pancreatitis
- Elevated serum amylase or lipase levels
- A personal or family history of medullary thyroid cancer or multiple endocrine neoplasia 2A or 2B
- Prior use of semaglutide or other GLP-1 agonists
- Current engagement in alcohol or smoking cessation treatments, or currently engaged in intentional efforts to quit alcohol use
- Baseline body mass index (BMI) <19.5kg/m2 or >30kg/m2
- Baseline fasting blood glucose levels under normal range

- Currently nursing, pregnant, anticipating pregnancy in the next 6 months, or unable/unwilling to use a reliable form of birth control (if sexually active) during the study period
- history of suicide attempt, or recent (past 30 day) suicidal ideation (i.e., intent or plan)
- Plans for travel outside of the local area in the upcoming 12 weeks that would interfere with lab visits during the study period (or other logistic factors that would make it difficult to commit to entire duration of study)
- History of diabetic retinopathy, proliferative retinopathy or maculopathy
- History or presence of malignant neoplasms within the last 5 years (except basal and squamous cell skin cancer and carcinoma in situ)
- History of major surgical procedures involving the stomach potentially affecting absorption of trial product (e.g., subtotal and total gastrectomy, sleeve gastrectomy, gastric bypass surgery)
- History of diabetic ketoacidosis
- Regular use of electronic nicotine delivery systems (vaping), cigars, chewing tobacco or snuff, based on at least weekly use in the past 30 days

5.3 LIFESTYLE CONSIDERATIONS

Specific lifestyle instructions will include:

- Refrain from alcohol consumption or other drug use for 24 hours prior to alcohol administration sessions
- Arrive fasting at sessions scheduled for blood draws (Weeks 0, 4, 8, and final discharge visit)
- Refrain from consuming food for 4 hours prior to alcohol sessions. Participants will receive a standardized snack on arrival for alcohol sessions.)
- Refrain from driving to the lab on the day of alcohol sessions. (Participants will be asked to arrange for transportation, or will be provided with transportation home via taxi or Uber)
- Refrain from operating other vehicles or machinery for the duration of day upon discharge from alcohol sessions
- Women participating in the study must report being willing to use a reliable birth control method during the trial (if sexually active), and must report no plans for pregnancy in the next 6 months.
- Participants will also understand that they may be excluded if they test positive for drugs other than cannabis during the time of scheduled drug screens (repeat drug tests will take place during weeks 4 and 8).

5.4 SCREEN FAILURES

Screen failures are defined as participants who consent to participate in the clinical trial but are not subsequently randomly assigned to the study intervention or entered in the study. A minimal set of screen failure information is required to ensure transparent reporting of screen failure participants, to meet the Consolidated Standards of Reporting Trials (CONSORT) publishing requirements and to

respond to queries from regulatory authorities. Minimal information includes demography, screen failure details, eligibility criteria, and any serious adverse event (SAE).

Examples of screen failures would include participants excluded following baseline laboratory work, or those excluded based on a change in treatment-seeking status at the time of the baseline assessment. Individuals who do not meet the criteria for participation in this trial (screen failure) because of a specify modifiable factor (e.g., upcoming travel) may be rescreened at a later date. Rescreened participants will be assigned the same participant number as for the initial screening. Data pertaining to screening failures will be tabulated in an anonymized fashion.

5.5 STRATEGIES FOR RECRUITMENT AND RETENTION

Participants will be recruited from the community through advertisements, which may include ads in local online or print newspapers, print flyers placed in community or hospital settings, business cards, and online postings (e.g. Craigslist (ads will not be placed in employment section), and social media ads (Facebook, Twitter, Instagram) that direct potential participants toward a study website/landing page. Participants may also be recruited from local medical clinics (e.g., primary care clinics) that grant approval for assisting with recruitment (e.g., by placing study advertisements in clinics). Flyers and online advertisements will include a web link and/or QR code leading to a brief preliminary eligibility screener. The eligibility screener will be programmed in REDCap and will contain questions related to the following eligibility criteria: Current alcohol use (quantity/frequency of drinking and AUDIT questionnaire); cigarette use (cigarettes per day and Fagerstrom Questionnaire), body weight and height (for estimating BMI), and birth sex. This questionnaire is expected to take roughly 5 minutes to complete. Participants will be asked to submit information for their preferred contact method (email address, phone number) in order to complete a full eligibility screening screening.

In addition to these “passive” methods of recruitment, we will also include “active” recruitment methods. These methods will include members of the study team setting up recruitment booths and passing out business cards at public events (e.g., festivals) or public venues. Once initial contact with participants is made (e.g., via email or in person), research staff will schedule the participant for a phone call to explain the study and conduct a screening interview.

Participants will receive cash compensation. In the event a cash payment is not possible due to inability to pick up the payment in person, participants will have the option of receiving an electronic gift card via email. The payments will be allocated as follows (please see 1.2 for a summary of all scheduled sessions):

- $50 for completing the consent/screening/baseline and placebo lead-in visit (Week 0)
- $75 for alcohol challenge session 1 (To be scheduled between Weeks 0-1)
- $75 for alcohol self-administration session 1 (To be scheduled between Weeks 0-1)
- $100 for alcohol challenge session 2 (To be scheduled between Weeks 9-10)
- $100 for alcohol self-administration session 2 (To be scheduled between Weeks 9-10)
- $30 per visit for each follow-up/medication administration visit during Weeks 1-10 (10 visits x$30 per visit = $300)
- Up to $154 for completing daily questionnaires via cellphone over the study period ($2 per daily survey x 77 days, which includes the week following the 1.0mg dose, to be paid at weekly medication visits)

- Up to $110 maximum for completing at least 6 of 7 daily assessments per week ($10 bonus per week x 11 weeks of daily surveys, to be paid at weekly medication visits)
- Participants will receive a final bonus of $100 for completing the study through at least the end of Week 9 (to be paid at the final discharge visit).

Therefore, the total maximum compensation over 10 weeks is $1064. Overall, these payments are structured to maximize retention in the study (given the long dose escalation period and time-intensive laboratory sessions). Additionally, an incentive-based structure that includes daily token incentives ($2 per brief daily survey) is important to reinforce engagement with daily surveys, and to minimize missing data. Participants will be updated at each visit with their progress toward the bonus incentives for adherence (e.g., weekly bonus of $10 for completing at least 6 of 7 surveys). In the event participants are not able to pick their final cash payment (or other payments) in person, we will offer the option of issuing the final payment via electronic gift card to the participant's email address, with their permission.

We anticipate phone screening up to 400 individuals and randomizing up to 48 participants to medication or placebo conditions, in order to obtain a final sample of at least 36 eligible participants who complete the full course of the maintenance dose (i.e., through at least the week 9 visit), allowing for 25% attrition). If attrition is higher than anticipated, we will submit an amendment to enroll additional participants to arrive at a final sample of at least 36 participants who complete study procedures through the end of the 0.5mg dosage phase (i.e., through the end of Week 9).

6 STUDY INTERVENTION

6.1 STUDY INTERVENTION(S) ADMINISTRATION

6.1.1 STUDY INTERVENTION DESCRIPTION

Semaglutide is a relatively new (approved in 2017) GLP-1 receptor agonist. A new oral formulation of semaglutide was approved by the FDA in 2019, making semaglutide the first drug in its class to be available in both subcutaneous and oral formulations. GLP-1 agonist medications were designed to mimic the function of the endogenous GLP-1 peptide, with the benefit of a much longer half-life than endogenous GLP-1. Based on their glucose-lowering and appetite-reducing properties, GLP-1 agonists were developed as anti-diabetic therapies. Several GLP-1 agonists (e.g., exenatide, liraglutide, lixisenatide, albiglutide, dulaglutide and semaglutide,) are now in widespread use as treatments for type II diabetes (T2D); these medications have also proven efficacious for weight loss, reducing glycated hemoglobin (HbA1c), and reducing risk for some cardiovascular events [29]. Although the primary indication of GLP-1 agonists is T2D, these therapies are increasingly being studied for other indications in non-diabetic patients, including obesity/weight management. In June 2021, the FDA approved semaglutide at a higher dosage (2.4mg/week) for the indication of weight management, under the brand name Wegoby.

6.1.2 DOSING AND ADMINISTRATION

Semaglutide (Ozempic®) will be administered according to the FDA-approved schedule, which begins with a starting dose of 0.25mg once per week for four weeks. At week five, dosage is increased to 0.5mg

for a subsequent four weeks (initial maintenance dose), after which point dosage can be increased further (to 1.0mg), presuming the medication is well tolerated.

Although semaglutide can be self-administered in the home setting, medication and placebo administration will be delivered by a research nurse at CTRC in order to execute the placebo manipulation and ensure adherence to medication. This approach also allows us to collect safety outcomes on a regular basis, further ensuring that participants will be monitored carefully. Semaglutide is administered subcutaneously (in the abdomen, thigh, or upper arm). Doses are delivered via the Ozempic® pen, a cartridge-based device that calibrates medication delivery based on the desired dose. An extremely small needle (4mm, 32 gauge needle – the size of 2 human hairs) is used to deposit the medication subcutaneously. Because each pen contains 2mg of medication, each participant in the medication group will be allocated one pen to cover the first six weeks following the placebo lead-in (i.e., 0.25mg/week for 4 weeks, followed by 0.5mg/week for 2 weeks), and a second pen for that will supply medication for the remaining three weeks (2 weeks at 0.5mg, and 1 week at 1.0mg). This schedule results in 10 medication administration sessions (inclusive of the placebo lead-in). This timeframe also allows sufficient time to reach steady-state plasma concentration levels (achieved within 4-5 weeks of initiating the starting dose). During the medication phase, participants' pens will be labeled by subject ID number and stored in a secure clinic location under the supervision of UNC's Investigational Drug Services (IDS) staff.

At the present time, we do not have access to a manufacturer-developed placebo (the medication relies on the proprietary Ozempic® pen for delivery). Therefore, this study will use weekly sham injections for the placebo group. Sham doses will involve a brief needle prick using a very small (e.g., 32 gauge) needle, identical to the one used in the Ozempic® pen (NovoFine). Given the extremely small volume of medication delivered in the treatment condition, we will not utilize saline or another inert substance for the placebo administration. During medication and placebo administration, participants will be asked to wear a blindfold and avert their gaze. Medication will be delivered in the upper non-dominant arm, unless the participant requests another site (abdomen, thigh). The same approach will be used for all participants for the initial lead-in placebo "dose". Given the extremely small volume of the Ozempic® injections, we will not utilize saline or another inert substance for the placebo administration; rather, the sham doses will consist of briefly inserting the micro-needle.

Due to the nature of the placebo condition, it will not be possible to blind the staff RNs administering the medication. However, we will provide training to study staff to minimize the likelihood of contaminating the blinding procedures. We will also develop procedures to minimize contact between the RNs and outcome assessors. All other staff members (including research assistants and outcomes assessors) will remain blind to condition.

6.2 PREPARATION/HANDLING/STORAGE/ACCOUNTABILITY

6.2.1 ACQUISITION AND ACCOUNTABILITY

The team will collaborate with IDS to source the medication (and NovoFine needles for the placebo condition), which will be stored at the IDS pharmacy. Participants assigned to medication will be prescribed one Ozempic® pen following randomization. Pens will be stored by IDS and not shared across participants. A refill pen will be prescribed prior to the Week 7 visit, once the doses from the first pen have been allocated. Any unused doses from individual pens (e.g., in the event of participant drop-out) will be discarded/destroyed by IDS, as will any pens that expire during the course of the trial. In the

event a pen expires before a participant completes the study, a replacement pen will be prescribed at the discretion of the study physicians.

6.2.2 FORMULATION, APPEARANCE, PACKAGING, AND LABELING

Ozempic® is manufactured by Novo Nordisk. As described above, the medication is dispensed from a proprietary device, the Ozempic® pen. Each pen contains 2mg of medication, requiring that each participant is prescribed two pens over the course of the study. A full description and visuals can be found at the following site:
https://www.Ozempic®pro.com/how-to-prescribe/Ozempic®-pen.html

The *placebo group* will receive sham injections that involve a brief needle-prick without dispensing a substance. The needles used for the sham injections will be the NovoFine 32G needle, the same needle in the Ozempic® pen:
https://www.novoneedles.com/novofine.html
To mimic the active medication administration, the medical staff member will insert the microneedle and hold it in place for a period of several seconds. The same approach will be used for the initial placebo “lead-in” dose (for all participants). Needles will remain in sterile packaging until use, and will be attached to an empty Ozempic® pen to mimic the active medication procedure.

6.2.3 PRODUCT STORAGE AND STABILITY

According to the manufacturer, following the first use, the Ozempic® pen can be stored for up to 56 days at room temperature (59 to 86 F) or refrigerated at 36 to 46 F. The IDS team will oversee the appropriate and secure storage of the drug. Each participant’s pen will be labeled with the study ID number and pens will not be shared across participants. Placebo needles will remain packaged until use, and will be stored at room temperature in a secure location.

6.2.4 PREPARATION

Semaglutide does not require advance preparation; cartridges are pre-filled and doses are calibrated using the pen device. The RN or medical staff member will select the correct dosage and administer the medication as per manufacturer guidelines. For each placebo session, a new sterile needle will be removed from its package and attached to an empty Ozempic® pen to carry out the sham dose.

6.3 MEASURES TO MINIMIZE BIAS: RANDOMIZATION AND BLINDING

Following the baseline assessment and one-week placebo lead-in period, participants will be randomized to medication or placebo conditions at a 1:1 ratio by a member of the IDS team using a pre-determined randomization scheme. The staff member administering medication will receive an active or empty pen from the IDS team in a sealed bag. Medication and placebo administration will be carried out by a staff RN at CTRC. Due to the nature of the placebo condition, it will not be possible to blind the RN to condition. However, all other staff members will remain blind to condition, and the staff members administering medication will be trained in protecting the blind (e.g., redirecting the conversation if the participant comments about medication condition; minimizing contact with experimenters.) To maintain blinding of participants, participants will be asked to wear a blindfold and face away from the injection

site during all placebo and medication administrations. Aside from the staff members administering medication, no study team members interacting with participants will be aware of participants' assignments. At the stage of interim analyses (e.g., in preparing safety reports for DSMB meetings) and final analyses, the study statistician will be provided with the assignment log. None of the study personnel involved in recruitment or enrollment will have knowledge of the details of the randomization table that will be used by the IDS pharmacy.

At the completion of the trial, participants will be provided with a questionnaire asking about perceived medication assignment (to assess the success of maintaining the blind). Due to common side effects of semaglutide (in particular nausea), we do not expect blinding to be successful in all cases. Upon completion of study procedures, participants will be informed of the medication doses that were administered over the course of the trial, including the initial lead-in placebo.

6.4 STUDY INTERVENTION COMPLIANCE

Medication will be administered by CTRC staff to assure compliance and reduce potential sources of non-adherence. A missed session will constitute a missed medication dose. As per manufacturer directions, any missed doses/appointments will be corrected by asking the participant to come in for the dose as soon as possible within 5 days of the missed dose. After the 5-day mark has passed, the dose will be skipped and the participant will resume with the next scheduled dose. Study medication logs will serve as the primary source document for tracking medication and placebo administration. Participants will be coded as non-adherent if they miss more than 2 scheduled doses between Weeks 0-9.

6.5 CONCOMITANT THERAPY

Participants may be taking medications for other conditions (provided those treatments are not included in the list of exclusion criteria). For this protocol, a prescription medication is defined as a medication that can be prescribed only by a properly authorized/licensed clinician. Medications to be reported in the Case Report Form (CRF) are concomitant prescription medications, over-the-counter medications and supplements. Changes to medication status or other treatments will be assessed at each in-person visit, with any changes reviewed by a study physician. Should a concomitant therapy preclude further participation for safety or scientific reasons (for instance, if a participant starts a therapy that constitutes an exclusion factor), this issue will be discussed by the study leads and the participant will be excused from further participation, if required to ensure safety. By definition, participants will not be seeking treatment for alcohol use or smoking cessation. If a participant makes the decision to seek treatment for alcohol or smoking during the study, s/he will be referred to appropriate treatment resources and excused from further participation.

6.5.1 RESCUE MEDICINE

Not applicable

7 STUDY INTERVENTION DISCONTINUATION AND PARTICIPANT DISCONTINUATION/WITHDRAWAL

7.1 DISCONTINUATION OF STUDY INTERVENTION

The study may be halted if deemed necessary by the lead investigators, DSMB, or IRB. Examples of reasons for discontinuation include unexpectedly high frequency of serious adverse events, or significant increases in alcohol use during treatment, leading to the conclusion that the medication is not safe in this population. Given the safety profile of semaglutide for other clinical conditions, as well as preclinical evidence that GLP-1 receptor agonists reduce drug intake, these scenarios are expected to be unlikely.

7.2 PARTICIPANT DISCONTINUATION/WITHDRAWAL FROM THE STUDY

Participants are free to withdraw from participation in the study at any time upon request. The investigators may discontinue or withdraw a participant from the study if clinically or otherwise indicated. Examples of reasons removing a participant include:

- Significant study intervention non-compliance
- Any clinical adverse event (AE), laboratory abnormality, or other medical condition or situation that indicates that continued participation in the study would be unsafe
- Pregnancy
- Diagnosis of significant disease or disease progression which requires discontinuation of the study intervention
- The participant meets an exclusion criterion (either newly developed or not previously recognized) that precludes further study participation
- Participant relocates from the local area, or is otherwise unable to attend visits
- Significant or persistent side effects that pose significant discomfort or preclude escalation to the 0.5mg dose (e.g., nausea, hypoglycemia)
- Changes in lab tests or clinical status that render the participant ineligible, based on the baseline inclusion/exclusion criteria (e.g., elevated liver enzymes, elevated serum amylase or lipase levels, weight loss resulting in BMI below the normal range)
- A change in treatment-seeking status (i.e., the participant expresses a desire to enter treatment for alcohol or smoking cessation).

If a participant exhibits changes in symptoms or clinical status that warrant potential removal, the study physicians will confer and render a decision as to whether to withdraw the participant. With respect to dosage increase, if a participant’s side effects profile or laboratory tests during the initial 4-week phase (.25mg) indicate that s/he is unable to safely proceed to the 0.5mg dose, the participant may be removed from the study at the discretion of the physicians. If lab tests during the standard maintenance dose of 0.5mg suggest that a participant is unlikely to tolerate a higher dose (1mg), they may be excluded from the 1.0mg dose escalation at the discretion of the physicians. In this case, participants will be given the option of one additional week at the standard therapeutic dose of 0.5mg (i.e., highest tolerated dose). Reasons for participant discontinuation or withdrawal from the study will be recorded on the Case Report Form (CRF). Participants who sign the informed consent form but do not receive the study intervention (e.g., fail to attend the Week 1 visit for randomization) may be replaced. Participants who sign the informed consent form, and are randomized and receive the study

intervention, and subsequently withdraw, or are withdrawn or discontinued from the study, may be replaced.

7.3 LOST TO FOLLOW-UP

A participant will be considered lost to follow-up if he or she fails to return for 2 consecutive scheduled medication visits and is unable to confirm attendance at the following visit (or is unable to be contacted by the study staff).

The following actions will be taken if a participant fails to return to a required study visit:

- The team will attempt to contact the participant and reschedule the missed visit within one week, and counsel the participant on the importance of maintaining the assigned visit schedule and ascertain if the participant wishes to and/or should continue in the study.
- Before a participant is deemed lost to follow-up, the investigator or designee will make every effort to regain contact with the participant (where possible, up to 3 telephone messages, up to 3 email messages, and if necessary, a letter to the participant's last known mailing address or local equivalent methods). These contact attempts will be documented in the participant's medical record or study file.
- Should the participant continue to be unreachable, he or she will be considered to have withdrawn from the study with a primary reason of lost to follow-up.

8 STUDY ASSESSMENTS AND PROCEDURES

8.1 EFFICACY ASSESSMENTS

The efficacy assessments/outcomes are noted in summary form in section 3. The ascertainment of these outcomes is described in further detail in section 4 (study design). Briefly, the endpoints are human laboratory measures of alcohol consumption and alcohol response; self-reported alcohol consumption and cigarette use over the course of the trial; and changes in weight, HbA1c, and alcohol elimination.

8.2 SAFETY AND OTHER ASSESSMENTS

Safety and assessments are summarized in detail in the study design section (Section 4.1). Briefly, safety assessments include a baseline medical examination, body weight, bloodwork, vitals signs, and clinical interview to assess substance use and psychiatric symptoms. Follow-up medical visits will include repeat assessments of bloodwork and scheduled time points, as well as follow-up measurements of body weight and vitals signs. Medication side effects will be assessed at each visit, and a study team member will contact participants within 2-3 days of each dosage increase to evaluate potential side effects. Other assessments (questionnaires, interviews) are noted in Section 4.1.

Safety precautions during alcohol administration sessions. Our team has considerable experience with alcohol administration procedures. Our procedures follow the NIAAA guidelines for administering alcohol in human studies (https://www.niaaa.nih.gov/research/guidelines-and-resources/administering-alcohol-human-studies). Based on theses guidelines, alcohol administration studies with participants who meet criteria for AUD are typically restricted to non-treatment-seeking participants in order to avoid ethical concerns, as will be the approach in this study. Additionally, our approach in this study is to

only recruit people who meet criteria for mild or moderate AUD (i.e., endorsing 2, 3, 4 or 5 symptoms out of 11). Regarding the alcohol dose for alcohol challenge sessions, we will use sex- and body-weight adjusted formulas [70] to ensure that dosage is calibrated across participants to achieve the target BrAC (.06g%). Participants will have undergone a medical screen prior to participation, and will be excluded if the study medical staff has reason to believe that alcohol is contraindicated. Participants will receive a BrAC reading on arrival to the alcohol sessions; the session will be rescheduled if the participant presents with a positive BrAC.

Following the completion of alcohol sessions, participants will be escorted to a private room designated for recovery purposes. During alcohol self-administration sessions, participants will only be discharged after their breath alcohol concentration descends below .03g%, which is consistent with NIAAA guidelines. During alcohol challenge sessions, participants will be asked to remain in the lab until BrAC reaches 0mg% (in order to collect BrAC data until alcohol is fully eliminated, for purposes of pharmacokinetic analyses). Participants will also be provided with reimbursement for transportation if they do not drive and are not able to locate assistance with transportation (participants will be advised not to drive to the alcohol sessions). If a participant presents with a positive BrAC, the session will be rescheduled, and the participant will be asked to remain in the lab until it is safe to leave.
To ensure participant safety, at least two members of the study staff will be present for the duration of all alcohol administration sessions, and a member of the study medical staff will be on call during these sessions. Additionally, any participants who express a desire to seek treatment during the study will be excused from further participation and provided with alcohol or smoking cessation resources. Co-I Dr. Jordan directs outpatient addiction treatment at UNC-CH.

8.3 ADVERSE EVENTS AND SERIOUS ADVERSE EVENTS

8.3.1 DEFINITION OF ADVERSE EVENTS (AE)

Adverse event means any untoward medical occurrence associated with the use of an intervention in humans, whether or not considered intervention-related (21 CFR 312.32 (a)).

8.3.2 DEFINITION OF SERIOUS ADVERSE EVENTS (SAE)

An adverse event (AE) or suspected adverse reaction is considered "serious" if, in the view of either the investigator or sponsor, it results in any of the following outcomes: death, a life-threatening adverse event, inpatient hospitalization or prolongation of existing hospitalization, a persistent or significant incapacity or substantial disruption of the ability to conduct normal life functions, or a congenital anomaly/birth defect. Important medical events that may not result in death, be life-threatening, or require hospitalization may be considered serious when, based upon appropriate medical judgment, they may jeopardize the participant and may require medical or surgical intervention to prevent one of the outcomes listed in this definition. Examples of such medical events include an adverse reaction requiring intensive treatment in an emergency room or at home.

8.3.3 CLASSIFICATION OF AN ADVERSE EVENT

8.3.3.1 SEVERITY OF EVENT

For adverse events (AEs) not included in the protocol defined grading system, the following guidelines will be used to describe severity.

- **Mild** – Events require minimal or no treatment and do not interfere with the participant's daily activities.
- **Moderate** – Events result in a low level of inconvenience or concern with the therapeutic measures. Moderate events may cause some interference with functioning.
- **Severe** – Events interrupt a participant's usual daily activity and may require systemic drug therapy or other treatment. Severe events are usually potentially life-threatening or incapacitating. Of note, the term "severe" does not necessarily equate to "serious".

8.3.3.2 RELATIONSHIP TO STUDY INTERVENTION

All adverse events (AEs) must have their relationship to study intervention assessed by the clinician who examines and evaluates the participant based on temporal relationship and his/her clinical judgment. The degree of certainty about causality will be graded using the categories below. In a clinical trial, the study product must always be suspect.

- **Related** – The AE is known to occur with the study intervention, there is a reasonable possibility that the study intervention caused the AE, or there is a temporal relationship between the study intervention and event. Reasonable possibility means that there is evidence to suggest a causal relationship between the study intervention and the AE.
- **Not Related** – There is not a reasonable possibility that the administration of the study intervention caused the event, there is no temporal relationship between the study intervention and event onset, or an alternate etiology has been established.

8.3.3.3 EXPECTEDNESS

The study physicians will be responsible for determining whether an adverse event (AE) is expected or unexpected. An AE will be considered unexpected if the nature, severity, or frequency of the event is not consistent with the risk information previously described for the study intervention.

8.3.4 TIME PERIOD AND FREQUENCY FOR EVENT ASSESSMENT AND FOLLOW-UP

The occurrence of an adverse event (AE) or serious adverse event (SAE) may come to the attention of study personnel during study visits and interviews of a study participant presenting for medical care, or upon review by a study monitor. At weekly medication visits, all participants will review a side effects checklist (SAFTEE) with a medical staff member at CTRC. The staff member will begin with an open-ended question about side effects or any other adverse events, followed by a review of specific side effects using the SAFTEE. Any previously reported side effects will be queried to determine any change in status (presence or severity). These weekly visits will be a primary method for identifying side effects. Additionally, a research staff member will contact participants by phone in the week following the placebo lead-in dose, as well as the week following each dose escalation. Phone calls will be scheduled 2-3 days following these events.

All AEs including local and systemic reactions not meeting the criteria for SAEs will be captured on the appropriate case report form (CRF). Information to be collected includes event description, time of onset, clinician's assessment of severity, relationship to study product (assessed only by those with the training and authority to make a diagnosis), and time of resolution/stabilization of the event. All AEs

occurring while on study will be documented appropriately regardless of relationship. All AEs will be followed to adequate resolution.

Any medical condition that is present at the time that the participant is screened will be considered as baseline and not reported as an AE. However, if the study participant's condition deteriorates at any time during the study, it will be recorded as an AE.

Changes in the severity of an AE will be documented to allow an assessment of the duration of the event at each level of severity to be performed. AEs characterized as intermittent require documentation of onset and duration of each episode.

Under the direction of the PI, members of the research team will record all reportable events with start dates occurring any time after informed consent is obtained until 7 (for non-serious AEs) or 30 days (for SAEs) after the last day of study participation. At each study visit, the team member will inquire about the occurrence of AE/SAEs since the last visit. Any such events will be followed for outcome information until resolution or stabilization.

8.3.5 ADVERSE EVENT REPORTING

All project staff will be trained to identify adverse events (AEs), which must be reported to the PI and study physicians within 24 hours. In addition, any unanticipated participant issues will be discussed in weekly staff meetings, which will provide another opportunity to identify AEs.

8.3.6 SERIOUS ADVERSE EVENT REPORTING

Research staff will be trained to report potential AEs or SAEs to the PI immediately. For any potential AEs/SAEs, the study physician will make a determination to distinguish between AEs, SAEs and unanticipated problems and provide judgment about attributions. Management and reporting of AEs, SAEs, and unanticipated problems will be carried out in accordance with Good Clinical Practice (GCP) standards, as outlined by the Food and Drug Administration. AEs will be defined as any negative medical event during the trial (whether or not the event is deemed related to the study medication). SAEs are defied as events that are potentially life threatening or result in heath; cause congenital malformation; result in persistent/significant disability or incapacity; require inpatient hospitalization or prolongation of hospitalization; or reflect an otherwise notable medical event in the judgment of the study physician. AEs/SAEs will be monitored regularly by the study research team and medical staff, in particular by inquiring about adverse events at each weekly visit. Should AEs or SAEs occur, they will be recorded, rated as mild/moderate/severe by the study physician, and reported in accordance with UNC IRB requirements and GCP standards. Any AEs will be reported to the IRB in progress reports and at the conclusion of the study. SAEs and unanticipated events will be reported to the IRB within 48 hours, and to NIAAA if judged as treatment-related. All staff working on this study will be made aware of the monitoring and reporting requirements for AEs/SAEs, and will have completed GCP training and other trainings required to conduct human subjects research at UNC.

Any AEs, SAEs or unanticipated problems will be followed until resolution by the PIs and/or research staff. Participants will be contacted regularly during follow-up and any observations of AEs/SAEs will be

discussed among the PIs and the study physician at regular meetings (or immediately, in the event of SAEs or unanticipated problems). In the event of SAEs or unanticipated problems, the physician and study PIs will be notified immediately, and the participant will be referred to or provided with appropriate medical support.

Participants will be advised to seek prompt medical attention in the event of an unanticipated medical event. In the event that the randomization code needs to be broken to appropriately resolve an SAE, the study pharmacist or delegate will break the blind and reveal the status to the study physician. In the event of an SAE, a member of the study medical staff will maintain regular contact with the participant until the event is resolved and fully documented.

Monitoring will be the primary responsibility of the PI. In addition, a Data and Safety Monitoring Board will be established to maximize safety. At minimum the DSMB will include one physician reviewer, and two additional scientists with expertise in medication trials. This board will meet semi-annually to review data quality, recruitment and retention, and to review all serious or clinically significant adverse events. In addition, the board will review safety data as-needed following any serious adverse event that appears to be study related. Patterns of adverse events as well as individual events may indicate the need for operational changes, protocol modifications, a decrease in dose, or, conceivably, discontinuation of the trial.

8.3.7 REPORTING EVENTS TO PARTICIPANTS

If an event is deemed an AE or SAE, participants will receive a phone call from study personnel to inform them that the event has been recorded and will be reported as such. Any incidental findings uncovered during the baseline medical exam or weekly visits (e.g., via bloodwork or other measurements) will be reported to the participant by the study physician, and an appropriate follow-up plan will be recommended to the participant.

8.3.8 EVENTS OF SPECIAL INTEREST

Not applicable

8.3.9 REPORTING OF PREGNANCY

At the baseline visit and each study visit that involves an alcohol session, female subjects will undergo a urine pregnancy test upon arrival to the clinical site. In the case of a positive pregnancy test, the participant will be notified immediately, and study intervention will be discontinued while continuing safety follow-up. If any follow-up plan is recommended by the study physician, one of the staff members will communicate this recommendation to the participant.

8.4 UNANTICIPATED PROBLEMS

8.4.1 DEFINITION OF UNANTICIPATED PROBLEMS (UP)

The Office for Human Research Protections (OHRP) considers unanticipated problems involving risks to participants or others to include, in general, any incident, experience, or outcome that meets **all** of the following criteria:

- Unexpected in terms of nature, severity, or frequency given (a) the research procedures that are described in the protocol-related documents, such as the Institutional Review Board (IRB)-approved research protocol and informed consent document; and (b) the characteristics of the participant population being studied;
- Related or possibly related to participation in the research ("possibly related" means there is a reasonable possibility that the incident, experience, or outcome may have been caused by the procedures involved in the research); and
- Suggests that the research places participants or others at a greater risk of harm (including physical, psychological, economic, or social harm) than was previously known or recognized.

Any UPs identified by the study team will be discussed amongst the study leadership team, and corrective actions will be identified. If applicable, a protocol amendment will be developed and submitted to the IRB.

8.4.2 UNANTICIPATED PROBLEM REPORTING

The investigator will report unanticipated problems (UPs) to the reviewing Institutional Review Board (IRB), and if applicable, the DSMB. The UP report will include the following information:

- Protocol identifying information: protocol title and number, PI's name, and the IRB project number;
- A detailed description of the event, incident, experience, or outcome;
- An explanation of the basis for determining that the event, incident, experience, or outcome represents an UP;
- A description of any changes to the protocol or other corrective actions that have been taken or are proposed in response to the UP.

8.4.3 REPORTING UNANTICIPATED PROBLEMS TO PARTICIPANTS

In the case of a UP that may impact participants' safety or alter their experience during the trial (e.g., by necessitating a change in protocol), participants will be notified by a study team member at the subsequent visit. In the event of any UP that may have an imminent impact on participant safety, a team member will contact the participant by phone to inform them of the problem.

9 STATISTICAL CONSIDERATIONS

9.1 STATISTICAL HYPOTHESES

- Primary Efficacy Endpoint(s):
  - Breath alcohol concentration (BrAC) and volume of alcohol consumed during laboratory alcohol self-administration procedures. The outcomes are continuous variables. The effect

of interest is the interaction between medication group (semaglutide vs. placebo) and time/dosage (0mg placebo lead-in vs. 0.5mg). The placebo lead-in session will take place between Weeks 0 and 1, and the 0.5mg session will take place between weeks 8 and 9.

- Secondary Efficacy Endpoint(s):
  - Changes in subjective responses to alcohol (stimulation, sedation, craving) and alcohol and cigarette demand during laboratory alcohol challenge procedures. The outcomes will be analyzed as continuous variables. The effect of interest is the interaction between medication group (semaglutide vs. placebo) and time/dosage (0mg placebo lead-in vs. 0.5mg). The placebo lead-in session will take place between Weeks 0 and 1, and the 0.5mg session will take place between weeks 8 and 9.
  - Changes in drinks per day and cigarette consumption over the course of the trial, as assessed via daily cell phone surveys. The outcome variables will be treated as continuous, however, this assumption will be tested and the modeling approach will revised as needed to properly account for the nature of the outcome variable (e.g., analyze as count variable with negative binomial distribution). The effect of interest is the interaction between medication group (semaglutide vs. placebo) and time (Week 1 to Week 11).

9.2 SAMPLE SIZE DETERMINATION

Because no human data on semaglutide's effects on alcohol consumption outcomes are available, there is no a priori effect size estimate to facilitate power calculations. We will seek a final sample size of 36 participants. Assuming at least 36 enrollees have complete data, our analyses suggest that this allows sufficient power (.80) to detect a medium effect size (f = .25) for an interaction between group (medication vs. placebo) and dosage (0mg lead-in, 0.5mg). This estimate assumes an alpha level of .05 and a within-person correlation (r) of 0.5 across time points. Further, because our multilevel model (MLM) analyses will involve more time points for certain analyses (e.g., daily assessments), this approach will increase power further, which is advantageous in the event that medication effects are in the small range. Assuming a 25% attrition rate, we anticipate needing to randomize 48 enrollees to arrive at a final sample of 36.

9.3 POPULATIONS FOR ANALYSES

Primary analyses will include all participants who were randomized and received a medication or placebo dose following the 1-week placebo lead-in (randomization will occur after the lead-in placebo dose). MLM analyses can accommodate missing data, allowing inclusion of participants without complete data (e.g., missed visits or missed daily survey responses). Reasons for missing data and drop-out will be documented in the study database and reported in any publications.

A supplementary per-protocol analysis will be conducted using all participants who received at least 80% of scheduled doses (following the placebo lead-in week). This planned analysis will be conducted irrespective of the number of participants who receive at least 80% of doses. If the sample permits, an exploratory subgroup analysis will examine participants as a function of weight (overweight/obese vs. not overweight or obese).

9.4 STATISTICAL ANALYSES

9.4.1 GENERAL APPROACH

Primary analyses will rely on multilevel models (MLM), which are well suited to examining within-person changes over time, while also accommodating missing data and unequal group sizes, and allowing the evaluation of between-subjects moderators (e.g., overweight/obesity status) if necessary. MLM with maximum likelihood estimation can accommodate missing data under the assumption that the data are missing at random. Secondary, sensitivity analyses will be conducted to test the robustness of study findings for violations of the assumption that data is missing at random. Specifically, we will examine if baseline variables are associated with data missingness (for instance, age, sex, level of substance use) using t-tests and chi-square analyses (for continuous and dichotomous variables, respectively). Baseline variables that are associated with data missingness will be included as covariates in additional adjusted models (that adjust for the baseline variables related to missingness). Assumptions for the statistical methods being used will be tested. Should there be evidence of violations of these assumptions (e.g., presence of outlying and influential cases, distributional concerns for residuals, and so on), appropriate corrective actions will be taken. Sensitivity analyses will be used to determine the extent to which the main study results are influenced by these violations. We will focus our interpretations on small p-values ($p < .05$) that indicate evidence against the null hypothesis. We will report the number of decimal places of the p-value in line with the journal's guidelines and APA recommendations (usually 2 or 3 decimal places). For specific outcomes, the units of measurement and expected ranges of values (or example ranges, if the full range is unknown) are noted in the subsequent analysis sections. Any extreme outliers (defined as >3.0 standard deviations from the mean) will be recoded to one unit greater than the next most extreme value, in order to reduce undue influence of extreme outliers. For all statistical tests, confidence intervals will be evaluated in combination with tests of statistical significance. Assumptions for the statistical methods being used will be tested. Should there be evidence of violations of these assumptions (e.g., presence of outlying and influential cases, distributional concerns for residuals, and so on), appropriate corrective actions will be taken. Sensitivity analyses will be used to determine the extent to which the main study results are influenced by these violations.

9.4.2 ANALYSIS OF THE PRIMARY EFFICACY ENDPOINT(S)

Alcohol self-administration sessions: MLM will be used to examine changes in laboratory self-administration (primary outcome) as a function of medication phase (0mg placebo lead-in; 0.5mg). These analyses will use medication phase as the within-subjects factor and condition (semaglutide vs. placebo) as the between-person factor. Self-administration will be measured based on recorded breath alcohol concentration (BrAC), measured in g% (e.g., .00g% - .08g%).

9.4.3 ANALYSIS OF THE SECONDARY ENDPOINT(S)

Alcohol challenge sessions: MLM will also be used to examine changes in 1) cigarette and alcohol demand during alcohol challenge (based on corresponding purchase tasks) (e.g., based on number of hypothetical drinks/cigarettes consumed at a given price point), and 2) self-reported stimulation (0-10 range), sedation (0-10 range), and craving (1-7 range), from BAC = .00 to BAC = .06g%, as a function of medication phase (within-person) and drug condition (between person). These analyses will examine changes in outcomes from BAC = .00 to .06 using medication phase as the within-subjects factor and condition as the between-person factor. The analyses will be complemented by analyses of peak

subjective effects (e.g., stimulation, sedation, craving, on the same scales as noted above), across the two alcohol administration sessions (AC1 and AC2).

Daily self-report data: We will use MLM to estimate within-person changes in primary outcomes (e.g., daily alcohol use [number of drinks consumed per day] and cigarette use [number of cigarettes smoked per day] over the trial period), using study week as the within-subjects factor and condition (semaglutide, placebo) as the between-person factor.

9.4.4 SAFETY ANALYSES

Total side effects reported (e.g., 0-15), and number of severe side effects reported during each medication phase will be assessed using MLM with medication phase (placebo lead-in, 0.25mg, 0.5mg, 1.0mg) as the within-subject factor and condition (semaglutide, placebo) as the between-person factor. . These count variables will be analyzed using a negative binomial MLM, however, this assumption will be tested and the modeling approach will revised as needed to properly account for the nature of the outcome variable (e.g., zero-inflated negative binomial regression).

9.4.5 BASELINE DESCRIPTIVE STATISTICS

Descriptive statistics will be used to report relevant demographics such as age, sex, and education.

9.4.6 PLANNED INTERIM ANALYSES

No interim analyses of efficacy will be performed.

9.4.7 SUB-GROUP ANALYSES

Not applicable

9.4.8 TABULATION OF INDIVIDUAL PARTICIPANT DATA

Not applicable

9.4.9 EXPLORATORY ANALYSES

Sex will be examined as an exploratory moderator in all analyses.

10 SUPPORTING DOCUMENTATION AND OPERATIONAL CONSIDERATIONS

10.1 REGULATORY, ETHICAL, AND STUDY OVERSIGHT CONSIDERATIONS

10.1.1 INFORMED CONSENT PROCESS

In obtaining and documenting informed consent, the team will comply with applicable regulatory requirements (e.g., 45 CFR Part 46, 21 CFR Part 50, 21 CFR Part 56) and adhere to ICH GCP. Consent will be obtained by a qualified study staff member in person at the outset of the baseline session. Additional procedures are noted below.

10.1.1.1 CONSENT/ASSENT AND OTHER INFORMATIONAL DOCUMENTS PROVIDED TO PARTICIPANTS

Consent forms will describe the study intervention, study procedures, and potential risks. The participant's written consent is required prior to starting intervention/administering study intervention. Participants will also be given an informational sheet about the study medication. Additional procedures are described below.

10.1.1.2 CONSENT PROCEDURES AND DOCUMENTATION

Informed consent is a process that is initiated prior to the individual's agreeing to participate in the study and continues throughout the individual's study participation. Consent forms will be Institutional Review Board (IRB)-approved and the participant will be asked to read and review the document. The investigator will explain the research study to the participant and answer any questions that may arise. A verbal explanation will be provided in terms suited to the participant's comprehension of the purposes, procedures, and potential risks of the study and of their rights as research participants. Participants will have the opportunity to carefully review the written consent form and ask questions prior to signing. The participants should have the opportunity to discuss the study with their family or surrogates or think about it prior to agreeing to participate. The participant will sign the informed consent document prior to any procedures being done specifically for the study. Participants must be informed that participation is voluntary and that they may withdraw from the study at any time, without prejudice. A copy of the informed consent document will be given to the participants for their records. The informed consent process will be conducted and documented in the source document (including the date), and the form signed, before the participant undergoes any study-specific procedures. The rights and welfare of the participants will be protected by emphasizing to them that the study is voluntary and the quality of their medical care will not be adversely affected if they decline to participate.

10.1.2 STUDY DISCONTINUATION AND CLOSURE

This study may be temporarily suspended or prematurely terminated if there is sufficient reasonable cause. Written notification, documenting the reason for study suspension or termination, will be provided by the suspending or terminating party to study participants, investigator, funding agency, and regulatory authorities. If the study is prematurely terminated or suspended, the Principal Investigator (PI) will promptly inform study participants, the Institutional Review Board (IRB), and sponsor and will provide the reason(s) for the termination or suspension. Study participants will be contacted, as applicable, and be informed of changes to study visit schedule. Circumstances that may warrant termination or suspension include, but are not limited to determination of unexpected, significant, or unacceptable risk to participants. Study may resume once concerns about safety, protocol compliance, and data quality are addressed, and satisfy the sponsor, IRB and/or Food and Drug Administration (FDA).

10.1.3 CONFIDENTIALITY AND PRIVACY

Participant confidentiality and privacy is strictly held in trust by the participating investigators, their staff, and the sponsor(s) and their interventions. This confidentiality is extended to cover testing of biological samples and genetic tests in addition to the clinical information relating to participants. Therefore, the study protocol, documentation, data, and all other information generated will be held in strict confidence. No information concerning the study or the data will be released to any unauthorized third party. All research activities will be conducted in as private a setting as possible.

Any computerized data will be password protected and contained on a secure, password-protected computer or a secure server. Recruitment data (de-identified) will be stored in a secure, password-protected file accessible only to the study investigators and personnel. At the end of recruitment, eligible subject files will be separated from those failing to meet eligibility criteria, and records of ineligible recruitment subjects will be anonymized. Data pertaining to screening failures will be tabulated in an anonymized fashion. Data collected in other formats (e.g., medical screening notes) will be labeled only by study identifier and stored in binders in secure, locked cabinets while the study is taking place. Files will be stored and archived in accordance with Good Clinical Practice (GCP) standards. For all questionnaire and interview assessments, participants will be informed that they may decline to answer any question if they so choose.

The study monitor, other authorized representatives of the sponsor, representatives of the Institutional Review Board (IRB), regulatory agencies or pharmaceutical company supplying study product may inspect all documents and records required to be maintained by the investigator, including but not limited to, medical records (office, clinic, or hospital) and pharmacy records for the participants in this study. The clinical study site will permit access to such records.

The study participant's contact information will be securely stored during the study for purposes of maintain contact with participants, and for communicating any safety information or unanticipated problems. At the end of the study, all records will continue to be kept in a secure location for as long a period as dictated by the reviewing IRB, Institutional policies, or sponsor requirements.

Study participant research data, which is for purposes of statistical analysis and scientific reporting, will be stored at UNC in the PI's laboratory and on secure backup servers with password-protected access. This will not include the participant's contact or identifying information. Rather, individual participants and their research data will be identified by a unique study identification number. Electronic data are stored on a password protected drive only accessible by the research team. Electronic data captured by REDCap are stored on a secure server, and identified by participants ID. For non-computer based forms, the data collection sheets are stored in a locked cabinet in a separate locked data storage space at the PI's laboratory.

At the end of the study, all study databases will be de-identified and archived in the PI's laboratory and backed up on a secure server hosted at UNC. De-identified data resulting from this study may also be presented at meetings, published in journals or presentations. In addition, this study will be registered at ClinicalTrials.gov, and de-identified information from this study will be submitted to ClinicalTrials.gov.

10.1.4 FUTURE USE OF STORED SPECIMENS AND DATA

Data collected for this study will be analyzed and stored at the PI's laboratory. The study biostatistician will be given access to the data necessary for conducting specified analyses, and data will be de-identified prior to giving access.

De-identified biological samples will be stored at UNC for the duration of the study, until all biological tests are completed and the data transferred to electronic format. De-identified DNA samples will be stored indefinitely, provided the participant has given consent for DNA storage, in order to examine future questions related to phenotypes of interest for this line of research.

During the conduct of the study, an individual participant can choose to withdraw consent to have biological specimens stored for future research. However, withdrawal of consent with regard to biosample storage may not be possible after the study is completed (after specimens are de-identified).

10.1.5 KEY ROLES AND STUDY GOVERNANCE

Provide the name and contact information of the Principal Investigator and the Medical Monitor.

**Principal Investigator**	**Medical Monitor**
Christian Hendershot, Ph.D., Associate Professor	*TBD (The medical monitor and DSMB members will be identified and added prior to data collection)*
UNC-Chapel Hill	
104 Manning Dr.	
Christian_Hendershot@med.unc.edu	

The investigative team is summarized on the face page of this protocol. As noted, a DSMB will be formed prior to carrying out the study.

Safety oversight will be under the direction of a Data and Safety Monitoring Board (DSMB) composed of individuals with the appropriate expertise, including addiction medicine and endocrinology. Members of the DSMB will be independent from the study conduct and free of conflict of interest. The DSMB will meet at least semiannually to assess safety and efficacy data on each arm of the study. The DMSB will operate under the rules of an approved charter that will be written and reviewed at the organizational meeting of the DSMB. At this time, each data element that the DSMB needs to assess will be clearly defined. The DSMB will provide its input to the IRB and National Institutes of Health as necessary.

10.1.6 CLINICAL MONITORING

Clinical site monitoring is conducted to ensure that the rights and well-being of trial participants are protected, that the reported trial data are accurate, complete, and verifiable, and that the conduct of the trial is in compliance with the currently approved protocol/amendment(s), with International Conference on Harmonisation Good Clinical Practice (ICH GCP), and with applicable regulatory requirement(s). A local study monitor will be appointed to provide on-site monitoring during the course of the research study.

10.1.7 QUALITY ASSURANCE AND QUALITY CONTROL

The PI will be responsible for addressing quality control issues and correcting errors or protocol deviations. The PI will have oversight over training study staff and ensuring that protocol elements are carried out with fidelity. Quality control will be ensured by developing SOPs for protocol elements to standardize data collection procedures. The investigational site will provide direct access to all trial related sites, source data/documents, and reports for the purpose of monitoring and auditing by the sponsor, and inspection by local and regulatory authorities.

10.1.8 DATA HANDLING AND RECORD KEEPING

10.1.8.1 DATA COLLECTION AND MANAGEMENT RESPONSIBILITIES

As a single-site, small-scale trial, the project will not involve coordinated data collection and management responsibilities across sites/teams. Data safety and storage precautions are outlined elsewhere in the study protocol.

10.1.8.2 STUDY RECORDS RETENTION

Study records will be retained for a minimum of 7 years following study completion. Paper source documents will be archived in accordance with UNC policies. Electronic materials and de-identified datasets will be stored securely as outlined above.

10.1.9 PROTOCOL DEVIATIONS

A protocol deviation is any noncompliance with the clinical trial protocol, International Conference on Harmonisation Good Clinical Practice (ICH GCP), or Manual of Procedures (MOP) requirements. The PI will report deviations within 7 working days of identification of the protocol deviation, or within 7 working days of the scheduled protocol-required activity. All deviations must be addressed in study source documents, reported to NIAAA Program Official and the IRB. Protocol deviations must be sent to the reviewing Institutional Review Board (IRB) per their policies. The site investigator is responsible for knowing and adhering to the reviewing IRB requirements. Further details about the handling of protocol deviations will be included in the MOP.

10.1.10 PUBLICATION AND DATA SHARING POLICY

This study will be conducted in accordance with the following publication and data sharing policies and regulations:

National Institutes of Health (NIH) Public Access Policy, which ensures that the public has access to the published results of NIH funded research. It requires scientists to submit final peer-reviewed journal manuscripts that arise from NIH funds to the digital archive PubMed Central upon acceptance for publication.

This study will comply with the NIH Data Sharing Policy and Policy on the Dissemination of NIH-Funded Clinical Trial Information and the Clinical Trials Registration and Results Information Submission rule. As

such, this trial will be registered at ClinicalTrials.gov, and results information from this trial will be submitted to ClinicalTrials.gov. In addition, every attempt will be made to publish results in peer-reviewed journals. Data from this study may be requested from other researchers after the completion of the primary endpoint by contacting the PI.

10.1.11 CONFLICT OF INTEREST POLICY

Any actual conflict of interest of persons who have a role in the design, conduct, analysis, publication, or any aspect of this trial will be disclosed and managed. Furthermore, persons who have a perceived conflict of interest will be required to have such conflicts managed in a way that is appropriate to their participation in the design and conduct of this trial. All investigators will make a conflict of interest declaration at the outset of the study and at regular intervals thereafter in accordance with UNC policies.

10.2 ADDITIONAL CONSIDERATIONS

Not applicable

10.3 ABBREVIATIONS

AE	Adverse Event
AUD	Alcohol Use Disorder
AUDIT	Alcohol Use Disorder Identification Test
BIS-11	Barratt Impulsivity Scale
BMI	Body Mass Index
CFR	Code of Federal Regulations
CMP	Clinical Monitoring Plan
CO	Carbon Monoxide
CRF	Case Report Form
CTRC	Clinical & Translational Research Center
DEQ	Drug Effects Questionnaire
DNA	Deoxyribonucleic acid
DSM-5	Diagnostic and Statistical Manual for Mental Disorders v.5
DSMB	Data Safety Monitoring Board
FDA	Food and Drug Administration
FTND	Fagerstrom test for Nicotine Dependence
GCP	Good Clinical Practice
GLP-1	Glucagon-Like Peptide 1
GLP-1R	Glucagon-Like Peptide 1 Receptor
HbA1c	Glycated hemoglobin
ICH	International Conference on Harmonisation
ICS	Impaired Control Scale
IRB	Institutional Review Board
MNWS	Minnesota Nicotine Withdrawal Scale

MOP	Manual of Procedures
NC Tracs	North Carolina Translational & Clinical Sciences Institute
NIAAA	National Institute on Alcohol Abuse and Alcoholism
NIH	National Institutes of Health
NRT	Nicotine Replacement Therapy
PACS	Penn Alcohol Craving Scale
PANAS	Positive and Negative Affect Scale
PPC	Peak Provoked Craving
PI	Principal Investigator
QA	Quality Assurance
QC	Quality Control
SAE	Serious Adverse Event
SAFTEE	Systematic Assessment for Treatment Emergent Effects
SOA	Schedule of Activities
SOP	Standard Operating Procedure
SRE	Self-Report of the Effects of Alcohol Questionnaire
SUD	Substance Use Disorder
T2D	Type II Diabetes
TLFB	Timeline Follow-Back Interview
TQSU	Tiffany Questionnaire of Smoking Urges
UP	Unanticipated Problem
UPPS-P	UPPS-P Impulsive Behavior Scale
5-HT2C	Serotonin 2C Receptpr

10.4 PROTOCOL AMENDMENT HISTORY

The table below is intended to capture changes of IRB-approved versions of the protocol, including a description of the change and rationale. A Summary of Changes table for the current amendment is located in the Protocol Title Page.

Version	Date	Description of Change	Brief Rationale

11 REFERENCES

1. Koob, G.F., G. Kenneth Lloyd, and B.J. Mason, *Development of pharmacotherapies for drug addiction: a Rosetta stone approach.* Nat Rev Drug Discov, 2009. **8**(6): p. 500-15.
2. Litten, R.Z., et al., *Medications development to treat alcohol dependence: a vision for the next decade.* Addiction biology, 2012. **17**(3): p. 513-27.
3. Litten, R.Z., et al., *Discovery, Development, and Adoption of Medications to Treat Alcohol Use Disorder: Goals for the Phases of Medications Development.* Alcohol Clin Exp Res, 2016. **40**(7): p. 1368-79.
4. Litten, R.Z., et al., *Potential medications for the treatment of alcohol use disorder: An evaluation of clinical efficacy and safety.* Subst Abus, 2016. **37**(2): p. 286-98.
5. Litten, R.Z., et al., *Heterogeneity of alcohol use disorder: understanding mechanisms to advance personalized treatment.* Alcohol Clin Exp Res, 2015. **39**(4): p. 579-84.
6. Heilig, M., et al., *Pharmacogenetic approaches to the treatment of alcohol addiction.* Nature reviews. Neuroscience, 2011. **12**(11): p. 670-84.
7. Falk, D.E., H.Y. Yi, and S. Hiller-Sturmhofel, *An epidemiologic analysis of co-occurring alcohol and tobacco use and disorders: findings from the National Epidemiologic Survey on Alcohol and Related Conditions.* Alcohol Res Health, 2006. **29**(3): p. 162-71.
8. McKee, S.A. and A.H. Weinberger, *How can we use our knowlegde of alcohol-tobacco interactions to reduce alcohol use?* Annu Rev Clin Psychol, 2013. **9**: p. 649–674.
9. Organization, W.H., *Global Health Risks. Mortality and Burden of Disease Attributable to Selected Major Risks*. 2009, World Health Organization: Geneva, Switzerland.
10. Dawson, D.A., *Drinking as a risk factor for sustained smoking.* Drug Alcohol Depend, 2000. **59**(3): p. 235-49.
11. Yardley, M.M., M.M. Mirbaba, and L.A. Ray, *Pharmacological Options for Smoking Cessation in Heavy-Drinking Smokers.* CNS Drugs, 2015. **29**(10): p. 833-45.
12. Prabhu, A., K.O. Obi, and J.H. Rubenstein, *The synergistic effects of alcohol and tobacco consumption on the risk of esophageal squamous cell carcinoma: a meta-analysis.* Am J Gastroenterol, 2014. **109**(6): p. 822-7.
13. Pelucchi, C., et al., *Cancer risk associated with alcohol and tobacco use: focus on upper aero-digestive tract and liver.* Alcohol Res Health, 2006. **29**(3): p. 193-8.
14. Kahler, C.W., N.S. Spillane, and J. Metrik, *Alcohol use and initial smoking lapses among heavy drinkers in smoking cessation treatment.* Nicotine Tob Res, 2010. **12**(7): p. 781-5.
15. Roche, D.J., et al., *Current insights into the mechanisms and development of treatments for heavy drinking cigarette smokers.* Curr Addict Rep, 2016. **3**(1): p. 125-137.
16. McKee, S.A., et al., *Varenicline reduces alcohol self-administration in heavy-drinking smokers.* Biol Psychiatry, 2009. **66**(2): p. 185-90.
17. King, A., et al., *Naltrexone decreases heavy drinking rates in smoking cessation treatment: an exploratory study.* Alcohol Clin Exp Res, 2009. **33**(6): p. 1044-50.
18. O'Malley, S.S., et al., *Dose-dependent reduction of hazardous alcohol use in a placebo-controlled trial of naltrexone for smoking cessation.* Int J Neuropsychopharmacol, 2009. **12**(5): p. 589-97.
19. King, A., et al., *Effects of the opioid receptor antagonist naltrexone on smoking and related behaviors in smokers preparing to quit: a randomized controlled trial.* Addiction, 2013. **108**(10): p. 1836-44.
20. Anton, R.F., et al., *Nicotine-Use/Smoking Is Associated with the Efficacy of Naltrexone in the Treatment of Alcohol Dependence.* Alcohol Clin Exp Res, 2018. **42**(4): p. 751-760.

21. Fucito, L.M., et al., *Cigarette smoking predicts differential benefit from naltrexone for alcohol dependence.* Biol Psychiatry, 2012. **72**(10): p. 832-8.
22. Hartmann-Boyce, J., et al., *Efficacy of interventions to combat tobacco addiction: Cochrane update of 2013 reviews.* Addiction, 2014. **109**(9): p. 1414-25.
23. Kahler, C.W., et al., *A Double-Blind Randomized Placebo-Controlled Trial of Oral Naltrexone for Heavy-Drinking Smokers Seeking Smoking Cessation Treatment.* Alcohol Clin Exp Res, 2017. **41**(6): p. 1201-1211.
24. Mitchell, J.M., et al., *Varenicline decreases alcohol consumption in heavy-drinking smokers.* Psychopharmacology (Berl), 2012. **223**(3): p. 299-306.
25. Fucito, L.M., et al., *A preliminary investigation of varenicline for heavy drinking smokers.* Psychopharmacology (Berl), 2011. **215**(4): p. 655-63.
26. Litten, R.Z., et al., *A double-blind, placebo-controlled trial assessing the efficacy of varenicline tartrate for alcohol dependence.* J Addict Med, 2013. **7**(4): p. 277-86.
27. Roche, D.J., et al., *Combined varenicline and naltrexone treatment reduces smoking topography intensity in heavy-drinking smokers.* Pharmacol Biochem Behav, 2015. **134**: p. 92-8.
28. Ray, L.A., et al., *Varenicline, low dose naltrexone, and their combination for heavy-drinking smokers: human laboratory findings.* Psychopharmacology (Berl), 2014. **231**(19): p. 3843-53.
29. Andersen, A., et al., *Glucagon-like peptide 1 in health and disease.* Nature Reviews Endocrinology, 2018. **14**(7): p. 390-403.
30. Jerlhag, E., *Alcohol-mediated behaviours and the gut-brain axis; with focus on glucagon-like peptide-1.* Brain Res, 2020. **1727**: p. 146562.
31. Brunchmann, A., M. Thomsen, and A. Fink-Jensen, *The effect of glucagon-like peptide-1 (GLP-1) receptor agonists on substance use disorder (SUD)-related behavioural effects of drugs and alcohol: A systematic review.* Physiol Behav, 2019. **206**: p. 232-242.
32. Egecioglu, E., J.A. Engel, and E. Jerlhag, *The glucagon-like peptide 1 analogue Exendin-4 attenuates the nicotine-induced locomotor stimulation, accumbal dopamine release, conditioned place preference as well as the expression of locomotor sensitization in mice.* PLoS One, 2013. **8**(10): p. e77284.
33. Anderberg, R.H., et al., *Glucagon-Like Peptide 1 and Its Analogs Act in the Dorsal Raphe and Modulate Central Serotonin to Reduce Appetite and Body Weight.* Diabetes, 2017. **66**(4): p. 1062-1073.
34. Howell, E., et al., *Glucagon-Like Peptide-1 (GLP-1) and 5-Hydroxytryptamine 2c (5-HT(2c)) Receptor Agonists in the Ventral Tegmental Area (VTA) Inhibit Ghrelin-Stimulated Appetitive Reward.* Int J Mol Sci, 2019. **20**(4).
35. Asarian, L., *Loss of cholecystokinin and glucagon-like peptide-1-induced satiation in mice lacking serotonin 2C receptors.* American Journal of Physiology-Regulatory, Integrative and Comparitive Physiology, 2009. **296**(1): p. R51-R56.
36. Nonogaki, K., et al., *The contribution of serotonin 5-HT2C and melanocortin-4 receptors to the satiety signaling of glucagon-like peptide 1 and liraglutide, a glucagon-like peptide 1 receptor agonist, in mice.* Biochem Biophys Res Commun, 2011. **411**(2): p. 445-8.
37. Doggrell, S.A., *Sgemaglutide in type 2 diabetes - is it the best glucagon-like peptide 1 receptor agonist (GLP-1R agonist)?* Expert Opin Drug Metab Toxicol, 2018. **14**(3): p. 371-377.
38. Litten, R.Z., et al., *Development of medications for alcohol use disorders: recent advances and ongoing challenges.* Expert Opin Emerg Drugs, 2005. **10**(2): p. 323-43.
39. Bujarski, S. and L.A. Ray, *Experimental psychopathology paradigms for alcohol use disorders: Applications for translational research.* Behav Res Ther, 2016.
40. Hendershot, C.S., et al., *Effects of naltrexone on alcohol self-administration and craving: meta-analysis of human laboratory studies.* Addict Biol, 2016.

41. McKee, S.A., *Developing human laboratory models of smoking lapse behavior for medication screening.* Addict Biol, 2009. **14**(1): p. 99-107.
42. Plebani, J.G., et al., *Human laboratory paradigms in alcohol research.* Alcohol Clin Exp Res, 2012. **36**(6): p. 972-83.
43. Ray, L.A., K.E. Hutchison, and M. Tartter, *Application of human laboratory models to pharmacotherapy development for alcohol dependence.* Curr Pharm Des, 2010. **16**(19): p. 2149-58.
44. Lerman, C., et al., *Translational research in medication development for nicotine dependence.* Nat Rev Drug Discov, 2007. **6**(9): p. 746-62.
45. MacKillop, J. and J.G. Murphy, *A behavioral economic measure of demand for alcohol predicts brief intervention outcomes.* Drug Alcohol Depend, 2007. **89**(2-3): p. 227-33.
46. Amlung, M., et al., *Increased behavioral economic demand and craving for alcohol following a laboratory alcohol challenge.* Addiction, 2015. **110**(9): p. 1421-8.
47. Green, R. and L.A. Ray, *Effects of varenicline on subjective craving and relative reinforcing value of cigarettes.* Drug Alcohol Depend, 2018. **188**: p. 53-59.
48. Murphy, C.M., et al., *Effects of varenicline versus transdermal nicotine replacement therapy on cigarette demand on quit day in individuals with substance use disorders.* Psychopharmacology (Berl), 2017. **234**(16): p. 2443-2452.
49. Roache, J.D., *Role of the Human Laboratory in the Development of Medications for Alcohol and Drug Dependence*, in *Addiction Medicine*, B.A. Johnson, Editor. 2010, Springer: New York. p. 129-157.
50. Enoch, M.A., et al., *Ethical considerations for administering alcohol or alcohol cues to treatment-seeking alcoholics in a research setting: can the benefits to society outweigh the risks to the individual? A commentary in the context of the National Advisory Council on Alcohol Abuse and Alcoholism -- Recommended Council Guidelines on Ethyl Alcohol Administration in Human Experimentation (2005).* Alcohol Clin Exp Res, 2009. **33**(9): p. 1508-12.
51. Motschman, C.A., et al., *Selection criteria limit generalizability of smoking pharmacotherapy studies differentially across clinical trials and laboratory studies: A systematic review on varenicline.* Drug Alcohol Depend, 2016. **169**: p. 180-189.
52. Alcoholism, N.I.o.A.A.a., *Recommended council guidelines on ethyl alcohol administration in human experimentation*. 2005, Department of Health and Human Services: Rockville, MD.
53. First MB, W.J., Karg RS, Spitzer RL, *Structured Clinical Interview for DSM-5—Research Version (SCID-5 for DSM-5, Research Version; SCID-5-RV)*. 2015, Arlington, VA: American Psychiatric Association.
54. Sobell, L.C., et al., *Reliability of a timeline method: assessing normal drinkers' reports of recent drinking and a comparative evaluation across several populations.* Br J Addict, 1988. **83**(4): p. 393-402.
55. Saunders, J.B., et al., *Development of the Alcohol Use Disorders Identification Test (AUDIT): WHO Collaborative Project on Early Detection of Persons with Harmful Alcohol Consumption--II.* Addiction, 1993. **88**(6): p. 791-804.
56. Schuckit, M.A., et al., *The Self-Rating of the Effects of Alcohol Questionnaire as a Predictor of Alcohol-Related Outcomes in 12-Year-Old Subjects.* Alcohol and Alcoholism, 2008. **43**(6): p. 641-646.
57. Schuckit, M.A. and T.L. Smith, *The relationships of a family history of alcohol dependence, a low level of response to alcohol and six domains of life functioning to the development of alcohol use disorders.* Journal of Studies on Alcohol, 2000. **61**(6): p. 827-835.
58. Heatherton, T.F., et al., *The Fagerstrom Test for Nicotine Dependence: a revision of the Fagerstrom Tolerance Questionnaire.* Br J Addict, 1991. **86**(9): p. 1119-27.

59. Shiffman, S., A. Waters, and M. Hickcox, *The nicotine dependence syndrome scale: a multidimensional measure of nicotine dependence.* Nicotine Tob Res, 2004. **6**(2): p. 327-48.
60. Patton, J.H., M.S. Stanford, and E.S. Barratt, *Factor structure of the Barratt impulsiveness scale.* Journal of clinical psychology, 1995. **51**(6): p. 768-774.
61. Whiteside, S.P., et al., *Validation of the UPPS impulsive behaviour scale: a four-factor model of impulsivity.* European Journal of personality, 2005. **19**(7): p. 559-574.
62. Heather, N., et al., *Development of a scale for measuring impaired control over alcohol consumption: a preliminary report.* Journal of studies on alcohol, 1993. **54**(6): p. 700-709.
63. Tiffany, S.T. and D.J. Drobes, *The development and initial validation of a questionnaire on smoking urges.* Br J Addict, 1991. **86**(11): p. 1467-76.
64. Welsch, S.K., et al., *Development and validation of the Wisconsin Smoking Withdrawal Scale.* Exp Clin Psychopharmacol, 1999. **7**(4): p. 354-61.
65. DiClemente, C.C., et al., *The Alcohol Abstinence Self-Efficacy scale.* J Stud Alcohol, 1994. **55**(2): p. 141-8.
66. Gwaltney, C.J., et al., *Does smoking abstinence self-efficacy vary across situations? Identifying context-specificity within the Relapse Situation Efficacy Questionnaire.* J Consult Clin Psychol, 2001. **69**(3): p. 516-27.
67. Mann, K., et al., *Precision Medicine in Alcohol Dependence: A Controlled Trial Testing Pharmacotherapy Response Among Reward and Relief Drinking Phenotypes.* Neuropsychopharmacology, 2018. **43**(4): p. 891-899.
68. Lesdema, A., et al., *Characterization of the Three-Factor Eating Questionnaire scores of a young French cohort.* Appetite, 2012. **59**(2): p. 385-90.
69. Epel, E.S., et al., *The reward-based eating drive scale: a self-report index of reward-based eating.* PLoS One, 2014. **9**(6): p. e101350.
70. Johnson, B.A., N. Ait-Daoud, and J.D. Roache, *The COMBINE SAFTEE: a structured instrument for collecting adverse events adapted for clinical studies in the alcoholism field.* J Stud Alcohol Suppl, 2005(15): p. 157-67; discussion 140.
71. George, T.P., et al., *A preliminary placebo-controlled trial of selegiline hydrochloride for smoking cessation.* Biol Psychiatry, 2003. **53**(2): p. 136-43.
72. Weafer, J., S.H. Mitchell, and H. de Wit, *Recent translational findings on impulsivity in relation to drug abuse.* Current addiction reports, 2014. **1**(4): p. 289-300.
73. Brick, J., *Standardization of alcohol calculations in research.* Alcohol Clin Exp Res, 2006. **30**(8): p. 1276-87.
74. Martin, C.S., et al., *DEVELOPMENT AND VALIDATION OF THE BIPHASIC ALCOHOL EFFECTS SCALE.* Alcoholism-Clinical and Experimental Research, 1993. **17**(1): p. 140-146.
75. Bohn, M.J., D.D. Krahn, and B.A. Staehler, *DEVELOPMENT AND INITIAL VALIDATION OF A MEASURE OF DRINKING URGES IN ABSTINENT ALCOHOLICS.* Alcoholism-Clinical and Experimental Research, 1995. **19**(3): p. 600-606.
76. Johanson, C.E. and E.H. Uhlenhuth, *Drug preference and mood in humans: diazepam.* Psychopharmacology, 1980. **71**(3): p. 269-73.
77. MacKillop, J., et al., *Further validation of a cigarette purchase task for assessing the relative reinforcing efficacy of nicotine in college smokers.* Exp Clin Psychopharmacol, 2008. **16**(1): p. 57-65.
