# Supplementary material for: Once-Weekly Semaglutide in Adults With Alcohol Use Disorder: A Randomized Clinical Trial
Source: JAMA Psychiatry. 2025 Feb 12;82(4):395–405. doi: 10.1001/jamapsychiatry.2024.4789 (PMC11822619; doi:10.1001/jamapsychiatry.2024.4789)
Supplement: Supplement 2. — eMethods. Supplementary protocol and analysis plan information eResults. Supplementary results information eTable 1. Adverse events by medication group and treatment dose/month eTable 2. Full results for all models examining laboratory self-administration eTable 3. Full results for linear mixed models examining changes in weekly outcomes eFigure 1. Duration of delay in laboratory consumption by medication group eFigure 2. Mean cigarettes per day by week in semaglutide and placebo groups (n = 13) eFigure 3. Descriptive comparison of drinking outcomes by baseline body mass index (BMI) [file jamapsychiatry-e244789-s002.pdf]

Supplemental Online Content

Hendershot CS, Bremmer MP, Paladino MB, et al. Once-weekly semaglutide in adults with alcohol use disorder: a phase 2 randomized clinical trial. *JAMA Psychiatry*. Published online February 12, 2025. doi:10.1001/jamapsychiatry.2024.4789

eMethods. Supplementary protocol, analysis and results information.

eTable 1. Adverse events by medication group and treatment dose/month

eTable 2. Full results for all models examining laboratory self-administration

eTable 3. Full results for linear mixed models examining changes in weekly outcomes

eFigure 1. Duration of delay in laboratory consumption by medication group

eFigure 2. Mean cigarettes per day by week in semaglutide and placebo groups (n = 13)

eFigure 3. Descriptive comparison of drinking outcomes by baseline body mass index (BMI)

This supplemental material has been provided by the authors to give readers additional information about their work.

© 2025 Hendershot CS et al. *JAMA Psychiatry*.

eMethods

Participant Sample

Participants were recruited from local community settings using a combination of advertising methods, including social media, public transit, and hospital-based advertisements. Because the protocol included laboratory alcohol administration procedures, non-treatment-seeking status was required. Study procedures emphasized that participants would not be asked to reduce or stop drinking, and participants actively seeking to cut down or quit alcohol use were excluded. Initial eligibility criteria requiring regular cigarette smoking were removed after trial commencement to facilitate recruitment. The full inclusion and exclusion criteria are as follows:

Inclusion criteria:

- Age 21-65
- Meeting DSM-5 criteria for current (past year) AUD, with between 2-7 symptoms endorsed, and NIAAA criteria for current at-risk drinking (i.e., >7/14 drinks in one week for women/men, with at least two episodes of 4+/5+ drinks in the past 30 days)
- Willingness/availability to take study medication and complete study procedures, including attending weekly visits for medication administration, side effect assessments, and glucose monitoring
- Willingness to complete laboratory sessions involving alcohol administration
- Ability to communicate and read in English

Exclusion criteria:

- Reporting past 30-day use of illicit drugs other than cannabis at baseline, or having a positive toxicology screen for illicit drugs other than cannabis at baseline
- Meeting past-year criteria for a substance use disorder (with the exception of alcohol, tobacco or mild cannabis use disorder)
- Current engagement in alcohol treatments, or currently engaged in intentional efforts to quit alcohol use
- Past 30-day use of: Sincalide, Sulfonylureas, insulin and insulin products or other medications that may interact with semaglutide, or weight control medications
- Prior use of semaglutide or other GLP-1 agonists
- Known or suspected hypersensitivity to study medication or related products
- Lifetime diagnosis of severe mental illness (including schizophrenia and bipolar disorder)
- History of suicide attempt, or recent (past 30 day) suicidal ideation, or psychiatric hospitalization in the last 6 months
- Current significant medical or neurological illness (based on self-report or medical record) including severe hepatic impairment or cirrhosis, impaired renal function (eGFR <50ml/min), acute or chronic pancreatitis, gastroparesis, gallbladder disease or cholelithiasis, other severe gastrointestinal disease, heart failure, coronary artery disease, stroke, seizure disorder, or other medical condition that poses a risk for the medication or alcohol administration components of the study (as determined by the MD)
- A personal or family history of medullary thyroid cancer or multiple endocrine neoplasia 2A or 2B
- Calcitonin greater than or equal to 50 ng/L
- Uncontrolled thyroid disease at screening
- History of major surgical procedures involving the stomach potentially affecting absorption of trial product (e.g., subtotal and total gastrectomy, sleeve gastrectomy, gastric bypass surgery)
- History of Type 1 or Type 2 diabetes, or HbA1c >6.5% measured at screening
- History of diabetic retinopathy, proliferative retinopathy, or maculopathy
- History of diabetic ketoacidosis
- History or presence of malignant neoplasms within the last 5 years (except basal and squamous cell skin cancer and carcinoma in situ)
- Currently nursing, pregnant, anticipating pregnancy in the next 6 months, or not using a highly effective contraceptive method as judged by the MD, and defined as:
    a. combined (estrogen and progestogen containing) hormonal contraception associated with inhibition of ovulation (oral, intravaginal, transdermal)

© 2025 Hendershot CS et al. *JAMA Psychiatry*.

   b. progestogen-only hormonal contraception associated with inhibition of ovulation (oral, injectable, implantable)
   c. intrauterine device
   d. intrauterine hormone-releasing system
   e. bilateral tubal occlusion
   f. vasectomized partner
   g. sexual abstinence
- Elevation of serum lipase, amylase, direct (conjugated) bilirubin, or alkaline phosphatase (ALP), ALT, or AST) more than 3X the upper limit of normal on baseline bloodwork
- Baseline body mass index (BMI) <23kg/m2
- Uncontrolled hypertension or systolic BP >180 mmHg and/or diastolic BP >105 mmHg, averaged from three measurements
- Plans for travel outside of the local area in the upcoming 12 weeks that would interfere with lab visits during the study period (or other logistic factors that would make it difficult to commit to entire duration of study)

Study Procedures

Eligibility was determined in three stages (phone screen, structured clinical interview, and baseline medical assessment); informed consent occurred at the structured interview. A pre-randomization visit (Week 0) included verification of eligibility criteria, weight and height measurements, assessment of alcohol, cigarette and other substance use, blood collection, and a urine toxicology screen. Blood collection was also repeated at Weeks 4, 8, and 10. Female participants were required to complete a urine pregnancy screen and confirm use of contraceptive measures or absence of pregnancy risk. Final eligibility was determined by study physicians and principal investigator. Confirmation of diagnostic eligibility criteria (including presence of criteria for AUD within the past 12 months and absence of other substance use disorders except for tobacco use disorder or mild cannabis use disorder) was obtained during the structured interview session. The Mini-International Neuropsychiatric Interview (MINI) [1]to screen for potential presence of exclusionary psychiatric disorders. The Structured Clinical Interview for DSM5 (SCID-5)[2] was used to confirm the presence of exclusionary substance use disorder.

A pharmacy staff member assigned participants to semaglutide or placebo (1:1) using a randomization sequence (pre-defined by the statistician using a random allocation code) immediately prior to the Week 1 visit. Semaglutide (purchased commercially in 2mg/3ml injection pens) was dispensed by the research pharmacy and administered subcutaneously by research nurses. Study staff and investigators were blind to assignment. Given requirements for medication/placebo, nurses (external to the research team) were not blind. The placebo group received sham subcutaneous injections using a needle of equivalent gauge as the medication delivery device. Similar approaches are being used in other trials of GLP-1 receptor agonists for substance use disorder. All participants were blindfolded for all medication procedures, with nurse interactions limited to 1-2 minute encounters. Post-treatment assessments of the effectiveness of blinding procedures were not planned as part of the trial.

Alcohol Self-Administration Procedures

Laboratory alcohol self-administration was selected as the *a priori* primary outcome on the basis that self-administration is objectively measured and sensitive to the effects of pharmacotherapy. [3,4] Medication effects on laboratory consumption were tested using a previously reported oral self-administration procedure that models extent of self-administration and the ability to delay/resist drinking.[4] Self-administration sessions occurred in a hospital setting at pre-treatment (prior to the Week 1 mediation visit) and post-treatment (following Week 8). Participants were asked to abstain from alcohol and recreational drugs for 24 hours, from food for at least 4 hours, and to not drive to sessions. Transportation was offered via rideshare for participants who did not have a ride to and from sessions. Upon arrival to the sessions, the mean reported duration since last meal for the medication and placebo groups (respectively) was 12.17 (SD = 3.47) hours and 11.97 (SD = 2.23) hours for self-administration session 1 (week 0), and 12.03 (SD = 3.41) and 11.92 (SD = 2.32) for self-administration session 2.

After arrival and completion of questionnaires, participants were situated in a dormitory-type setting and were presented with their preferred brand and type of alcohol beverage (with preferred mixers and glassware if applicable).[4] The available alcohol volume was determined based on beverage alcohol concentration and anthropometric formula-based estimates[5] of the maximum ethanol volume (g) that could be consumed without

exceeding a theoretical safety threshold (.12g/dl), with formulas taking into account body weight measured immediately before the session. Upon beverage presentation, participants were instructed that they could elect to delay drinking in exchange for nominal monetary reinforcement, available on a decreasing schedule, for up to 50 minutes.[4] In this task, the amount of delay time in minutes (not a registered outcome) indexes the ability to delay/resist drinking.[4] Participants were instructed that once drinking commenced (i.e., upon electing to drink or at the 50 minute mark) they should consume at their preferred pace over the next 120-min period in order to maintain pleasurable effects, but without experiencing aversive effects. Participants provided serial measures of breath alcohol concentration (BrAC) every 30 minutes following the onset of self-administration. At 120 minutes the remaining alcohol volume was measured and discarded, and participants were monitored until discharge. Self-administration was indexed by measurements of g-ETOH consumed and maximum BrAC measured at any point after the initiation of drinking. BrAC readings across time points during the self-administration session are depicted in **Figure 2d**. A separate alcohol administration session, involving a fixed dose of alcohol and assessment of subjective responses to alcohol and alcohol elimination rate, preceded the alcohol self-administration by at least 24 hours (data to be reported separately). Laboratory days were censored from weekly (calendar-based) drinking estimates.

Clinical and Safety Outcomes Assessment

Weight, vital signs, side effects/adverse events (AEs), and alcohol and cigarette use outcomes were obtained at weekly visits. Side effects were assessed using the Systematic Assessment for Treatment Emergent Events (SAFTEE), a structured instrument for assessing AEs[6]. Identical AEs reported across more than one timepoint were coded as a single event if experienced continuously (i.e., neither symptom offset nor increase in severity was recorded between assessments), and distinct events if increased severity or offset were recorded between assessments. Change in appetite was recorded as a side effect/AE. Final determinations of AE severity and relation to treatment were made at the discretion of the study physician.

Weekly Alcohol Use Outcomes

Weekly drinking outcomes were defined as follows: a) average drinks per day (total standard drinks divided by calendar days since the prior visit), b) average drinks per drinking day (total drinks divided by number of drinking days since the prior visit), c) number of drinking/non-abstinent days since the prior visit, and number of heavy drinking days (HDD), defined as ≥4 drinks for women and ≥5 drinks for men) since the prior visit. Drinks per calendar day was registered as a secondary outcome; others were included as exploratory and hypothesis-generating outcomes that align with typical outcomes in AUD clinical trials. From these data, the proportion of participants with zero HDD was computed, based on relevance as an acceptable FDA endpoint in Phase III clinical trials. The proportion of participants with zero HDD was estimated separately by arm (semaglutide, placebo) and by dosage phase (0.25mg/week, Weeks 1-4; 0.5mg/week, Weeks 5-8) to estimate potential dosage effects on change in the absence of HDD (note that all participants had HDD at baseline, based on eligibility criteria). For descriptive purposes we calculated baseline alcohol consumption risk levels, defined by World Health Organization (WHO) drinking risk levels. WHO level is also under consideration as a new FDA endpoint for pivotal trials.[7] WHO risk level criteria are determined by sex-specific cutoffs for average daily quantity of alcohol consumption: Level 1 (Low Risk) = <40g for males, <20g for females; Level 2 (Moderate Risk) = 40-60g for males, 20-40g for females, Level 3 (High Risk) = 60-100g for males, 40-60g for females, and Level 4 (Very High Risk) = >100g for males, >60g for females. Risk levels were determined based on average daily consumption over the index period (e.g., baseline, Weeks 1-4, Weeks 4-8). Risk levels were not examined as exploratory endpoints due to the sample size, which led to small numbers of participants within each risk level. Biological indicators of drinking/abstinence were not included due to the non-treatment nature of the trial (participants were provided no instructions to reduce drinking, and participants were not attempting to achieve drinking reductions or abstinence).

Sample Size Determination and Analysis Plan

Sample size determination. A priori power analyses were conducted in GPower software[8]. Power analyses were based on a repeated-measures ANOVA to conservatively approximate power for linear mixed models. Analyses determined that a minimum final sample size of 36 would yield sufficient power (>.80) to detect a medium effect size (f=.25) for an interaction between group (medication vs. placebo) and time, presuming a minimum of two time points (i.e., pre- and post-treatment for laboratory alcohol outcomes), an alpha level of .05, and within-person correlations of r=0.5 (observed within-person correlations for self-administrating outcomes in the present sample were r = .60 for grams of alcohol consumed and r = .51 for peak breath alcohol concentration). The sample size was

© 2025 Hendershot CS et al. *JAMA Psychiatry*.

determined based on the primary outcomes (laboratory self-administration); repeated weekly measurements were expected to yield relatively greater power. A goal of 48 randomized participants was set to allow a final sample of at least 36 (allowing for up to 25% attrition).

Baseline sample characteristics, side effects/AEs, and adherence (outpatient attendance) rates were examined descriptively. For all weekly outcomes (e.g., body weight, weekly alcohol use and craving), time was denoted by treatment week to align with the outpatient schedule (with Week 1 commencing on the day of the first injection). Because prior-week drinking and craving outcomes are assessed at the following clinic visit, drinking/craving estimates for a given week reflect values recorded at the subsequent visit (i.e., weekly outcomes for the Week 1 injection were recorded at the Week 2 visit). Baseline values were estimated based on weekly consumption over the 28 days preceding baseline.

For laboratory outcomes (grams consumed, peak BrAC), the original analytic plan specified testing the treatment-by-time interaction. Subsequently, based on recommendations from two anonymous reviewers, this approach was modified to estimate the medication effect by modeling post-treatment consumption (at the second laboratory session) while controlling for consumption at the first laboratory session. This approach serves to isolate the test of post-treatment consumption by modeling residualized change in consumption at post-treatment, allowing for a more direct test of post-treatment gains free from bias related to pre-treatment differences. Based on these recommendations, tests of medication effects were revised to focus on residualized change. However, full results of both residualized change models and linear mixed effects models are presented for transparency (**eTable2)**. The linear mixed effects models examined changes in the outcome with a time-by-condition interaction. Additionally, at the recommendation of an anonymous reviewer, a supplemental examination of the time course of BrAC measurements following self-administration was conducted (see **Figure 2d** for descriptive data and **Figure 2c** for mean BrAC values).

Although all participants (N=48) contributed data to the alcohol delay outcome, analyses of laboratory self-administration outcomes were impacted by a number of sessions in which participants elected to not initiate alcohol consumption (contrary to instructions to consume at their desired pace to achieve desired effects). Specifically, four participants elected not to consume during the pre-treatment laboratory session prior to randomization (2 placebo, 2 semaglutide), and 16 participants (7 placebo, 9 semaglutide) elected not to consume at the post-treatment laboratory session, leaving 25 participants with post-treatment self-administration data. Based on experimenter reports, decisions not to consume often related to low motivation to consume in the laboratory setting in the morning. Although participants who decided not to consume contributed data to the delay outcome, decisions not to consume resulted in the presence of zeros for self-administration outcomes, undermining the accuracy of estimates of medication effects on the amount of grams of alcohol consumed and pBrAC during alcohol consumption. Importantly, the decision not to engage in the laboratory consumption task was not accounted for by medication assignment, as confirmed by a binary logistic regression; Odds Ratio = .84, CI [.23, 2.98], p = .79). Participants who declined to engage in drinking therefore had missing data for the self-administration outcomes (g-EOTH and peak BrAC). Comparison of those with and without missing data for self-administration outcomes showed that those without missing data had significantly higher baseline craving, higher AUDIT scores, and higher mean number of AUD symptoms (*p*s<.05). However, the two groups did not differ on any other baseline estimates of quantity/frequency (e.g., drinks per day, drinks per drinking day, number of heavy drinking days; *p*s>.35). Although missing data reduces sample size, these models nonetheless estimate the effect of semaglutide on quantity of consumption once drinking begins (the effect of interest for laboratory outcomes). Missing data impacted both the residualized change models and the linear mixed effects models. Results of all models are presented in **eTable2**.

Except where noted, weekly outcomes were evaluated with linear mixed effects models and full information maximum likelihood estimation to accommodate missing data (lme4 package (v35.5)[9], RStudio (v2024.04.2+764)[10]. All models used intent-to-treat (ITT) principles (all 48 randomized participants included). Mixed models included a random effect for participant, a fixed effect of time (within-subjects factor), medication/treatment group (semaglutide, placebo), the treatment-by-time interaction, and covariates (e.g., biological sex, baseline value of the corresponding outcome). The number of drinking days and the number heavy drinking days are count variables and the number of heavy drinking days was overdispersed. Thus, the number of drinking days was modeled with a Poisson distribution and the number heavy drinking days was modeled with a negative binomial distribution. For weekly outcomes, the effect of interest was the medication condition effect (semaglutide vs. placebo). Condition-by-time interactions were also tested to examine the possibility of changes in the magnitude of group differences over

© 2025 Hendershot CS et al. *JAMA Psychiatry*.

time (for example, as a result of dose increase and/or greater duration of medication). Although an initial aim was to recruit a substantial proportion of cigarette smokers to examine medication effects on cigarettes per day (CPD, a registered secondary outcome), low rates of smoking in the sample population led to a relatively small number of cigarette smokers randomized reporting at least weekly smoking at baseline (N=13). Because CPD was a registered outcome, a linear mixed model evaluating CPD included this subset of participants (n=13; 7 placebo, 6 semaglutide), with restricted maximum likelihood estimation given the small sample size.

Prior to finalizing all models, sensitivity analyses were conducted to verify that findings were robust to the presence of outliers or study dropouts. To examine potential influence of outliers, analyses were repeated with and without any outliers removed, and with and without calculation of robust standard errors (using the package robustlmm version (v3.3-1))[11]. Results were consistent across these models; therefore, primary results are reported including the maximum number of available participants. Given variable medication dosage at Week 9 and some discontinuations occurring between Weeks 8-10, sensitivity analyses were also conducted for weekly outcomes while censoring data after completion of the 8th week of treatment. Alpha adjustments for multiple outcomes were not undertaken given the modest sample size, the hypothesis-generating (rather than confirmatory) aims of the study, and the risk of introducing Type II error (i.e., erroneously concluding that semaglutide does not impact alcohol consumption), the latter being an important consideration given the aim to determine whether a possible medication signal exists[12-14]. Given the modest sample size and to reduce reliance on p-values alone, observed effect sizes were also computed. Computing effect sizes is also important to estimate initial effect sizes of semaglutide on human alcohol consumption (given no prior data from randomized trials), and to guide sample size estimation for future studies. Finally, based on prior evidence[15] that the effects of exenatide on alcohol consumption might be evident only in AUD participants with obesity, drinking outcomes were also stratified by obesity status (BMI ≥30 vs. <30). These subgroups were not subjected to formal analysis and are presented for descriptive purposes only (**eFigure3**).

Treatment Adherence and Retention Results

Forty-two participants completed the medication/placebo sequence visits through Week 9; 19/24 (79.1%) from the placebo arm, and 23/24 (95.8%) from the semaglutide arm. The sole drop-out in the semaglutide arm was non-treatment-related. The mean days between medication visits (Weeks 1-9) was 7.87 (SD=1.83). Treatment adherence (proportion of 9 scheduled medication visits attended) was 92.4% (96.3% in the semaglutide arm and 88.4% in the placebo arm); this difference was not statistically significant ($p > .10$).

Of 42 participants who attended Week 9, most (n=33) were scheduled to receive the 1.0mg dose (or placebo). Six participants (4 semaglutide, 2 placebo) were maintained at 0.5mg (or placebo). Of these, 1 was maintained due to side effects, but most (5/6) were held at 0.5mg to accommodate alcohol session (re)scheduling: in the event of a delayed or rescheduled alcohol session, dosage was held at 0.5mg to ensure that the primary outcome was evaluated at the 0.5mg/week dose. Three participants (all in the semaglutide group) had the Week 9 dose held at 0.5mg (n=1) or deferred entirely (n=2) to avoid risk of worsening side effects. Two participants (both in the placebo group) who completed both dosage sequences did not complete post-treatment alcohol sessions. One participant elected to discontinue participation prior to the alcohol session, and one participant could not complete the post-treatment alcohol session (but completed the discharge visit).

Clinical/Safety Outcome Results

Nearly all (22/24) semaglutide-treated participants, and most (18/24) placebo-treated participants, reported one or more AE (**eTable1**). Of semaglutide-treated participants, 8 reported at least one side effect of moderate or greater severity; remaining participants reported only mild effects. The most frequently reported side effects in the medication group were decreased appetite (75% reported in the medication condition vs. 41.7% in placebo condition), nausea (70.8% vs. 16.7%), constipation (50% vs. 8.3%) and headache (50% vs. 20.8%). No participants in the medication arm reported serious adverse events, and no medication-related discontinuations were observed. There was one adverse event leading to study discontinuation: one participant in the placebo arm discontinued the study during COVID-19 infection.

No significant adverse events were associated with alcohol administration. Across all alcohol administration sessions (four scheduled per participant, including two alcohol challenge sessions not reported here), two events of emesis were recorded. These both occurred during the standardized alcohol administration sessions, which preceded alcohol administration sessions and did not contribute to the present analysis. One emesis event occurred in the

© 2025 Hendershot CS et al. *JAMA Psychiatry*.

semaglutide group and one in the placebo group. No emesis was recorded during self-administration sessions, indicating that semaglutide did not result in emesis when participants were able to titrate their level of alcohol consumption. Linear mixed models indicated no significant changes in HbA1C, systolic or diastolic blood pressure, or depression scores (CES-D) from baseline through Week 10 as a function of time, medication, or their interaction (all *p*s non-significant).

Laboratory Self-Administration Results

A total of 41 participants completed post-treatment alcohol self-administration sessions. All participants who completed post-treatment alcohol self-administration sessions completed the required sequence of four 0.5mg/week doses prior to collection of primary outcomes. The delay time variable (measured in minutes) exhibited bimodal distribution; therefore, this outcome was dichotomized (no delay time vs. any delay time) and analyzed with a binary logistic model. Medication did not delay drinking onset as a function of timepoint, $z = 0.14$, $p = .89$, odds ratio = 1.24; 95% CI [0.06, 24.17]), $d = .18$; **eFigure1**.

Primary effects of interest for self-administration are summarized in the main text and **Figure 2a-b**, with full model results depicted in **eTable2**. Results confirmed that the residualized change models resulted in more consistent effect sizes and confidence intervals compared to linear mixed models; thus, the residualized models are interpreted as best estimating the effect of semaglutide on in-the-moment alcohol self-administration. In residualized models, semaglutide showed medium-to-large effect sizes for both grams consumed and peak BrAC. Although peak BrAC served as a primary outcome of interest, BrAC readings were available at 30-minute intervals (for participants who consumed alcohol) and are presented for descriptive purposes in **Figure 2d.** An exploratory analysis of group differences in the mean BrAC across timepoints showed that the semaglutide group had significantly lower mean BrAC across the laboratory session $\beta = -.48$, $p = .019$, 95% CI [-0.87, -0.09]; group means are presented in **Figure2c.** Descriptively, the continuous BrAC readings suggest the possibility of earlier de-escalation or discontinuation of consumption in the semaglutide-treated group at post-treatment relative to pre-treatment, and relative to placebo-treated participants, potentially illustrating the "satiety" effect described in some patient reports[16].

Sensitivity Analyses for Weekly Alcohol Consumption, Craving, and Cigarettes Per Day Models

Results of primary ITT models for weekly outcomes are presented in the manuscript body. Sensitivity analyses in which weekly data were censored after Week 8 did not yield different conclusions, indicating that primary results were not influenced by variability in the Week 9 dose or drop-outs in the placebo group that occurred after Week 8. Similarly, sensitivity analyses that examined the potential influence of outliers on drinking variables did not alter the findings, allowing use of full randomized sample in ITT analyses. For the analysis of cigarettes per day (CPD) among smokers, one participant in the placebo group reported higher cigarette intake (approximately 40 cigarettes per day) than the remainder of the sample, contributing to increased variability in CPD in the placebo group (**eFigure2**). As an additional sensitivity analysis, the CPD model was re-run excluding this participant. Exclusion of the participant did not alter the conclusions (the medication-by-time interaction term remained significant ($p = .006$); therefore, the participant was retained in the final analysis.

BMI Subgroups. For descriptive purposes, weekly alcohol use outcomes are depicted by BMI subgroup in **eFigure 3**. Whereas a prior report found exploratory evidence that exenatide decreased alcohol consumption only in those with BMI of 30+ kg/m^2 or above,[15] descriptive results indicated relatively larger medication group differences in those with a baseline BMI < 30 kg/m^2.

eTable 1. Adverse Events By Medication Group and Treatment Dose/Month.

	Semaglutide[a]			Placebo		
	**Total**	**0.25 mg**	**0.50 mg**	**Total**	**0.25 mg**	**0.50 mg**
No. (%)	(n = 24)	(n = 24)	(n = 23)	(n = 24)	(n = 24)	(n = 21)
Any Adverse Event[b]	22 (91.7)	19 (79.2)	19 (82.6)	18 (75.0)	16 (66.7)	12 (57.1)
Serious Adverse Events	0 (0.0)	0 (0.0)	0 (0.0)	0 (0.0)	0 (0.0)	0 (0.0)
Adverse events leading to discontinuation of trial regimen	0 (0.0)	0 (0.0)	0 (0.0)	1 (4.2)[c]	1 (4.2)[c]	0 (0.0)
Severity[d]						
Mild	22 (91.7)	19 (79.2)	19 (82.6)	18 (75.0)	16 (66.7)	11 (52.4)
Moderate	8 (33.3)	4 (16.7)	5 (21.7)	4 (16.7)	2 (8.3)	2 (9.5)
Severe	2 (8.3)	0 (0.0)	2 (8.7)	1 (4.2)	0 (0.0)	1 (4.8)
Adverse events						
Decreased Appetite	18 (75.0)	15 (62.5)	16 (69.6)	10 (41.7)	9 (37.5)	5 (23.8)
Nausea	17 (70.8)	11 (45.8)	11 (47.8)	4 (16.7)	3 (12.5)	2 (9.5)
Constipation	12 (50.0)	8 (33.3)	9 (39.1)	2 (8.3)	1 (4.2)	1 (4.8)
Headache	12 (50.0)	9 (37.5)	5 (21.7)	5 (20.8)	4 (16.7)	1 (4.8)
Diarrhea	10 (41.7)	4 (16.7)	7 (30.4)	9 (37.5)	7 (29.2)	5 (23.8)
Fatigue	9 (37.5)	3 (12.5)	7 (30.4)	6 (25.0)	5 (20.8)	3 (14.3)
Abdominal Pain	6 (25.0)	2 (8.3)	5 (21.7)	4 (16.7)	3 (12.5)	2 (9.5)
Other	5 (20.8)	1 (4.2)	4 (17.4)	3 (12.5)	2 (8.3)	1 (4.8)
Dizziness	3 (12.5)	0 (0.0)	2 (8.7)	2 (8.3)	2 (8.3)	0 (0.0)
Insomnia	3 (12.5)	2 (8.3)	3 (13.0)	5 (20.8)	4 (16.7)	4 (19.0)
Increased Appetite	2 (8.3)	1 (4.2)	0 (0.0)	4 (16.7)	2 (8.3)	3 (14.3)
Nervousness/Anxiety	2 (8.3)	1 (4.2)	2 (8.7)	7 (29.2)	7 (29.2)	2 (9.5)
Vomiting	2 (8.3)	0 (0.0)	2 (8.7)	3 (12.5)	1 (4.2)	2 (9.5)
Depression	1 (4.2)	0 (0.0)	1 (4.3)	2 (8.3)	1 (4.2)	1 (4.8)
Increased Libido	1 (4.2)	0 (0.0)	1 (4.3)	2 (8.3)	2 (8.3)	1 (4.8)

© 2025 Hendershot CS et al. *JAMA Psychiatry*.

[a] Standard treatment regimen followed a 4-week interval dose escalation from 0.25mg (Weeks 1-4) to 0.50mg (Weeks 5-8). Primary outcomes were collected following the fourth 0.5mg dose (after Week 8). A final dose of 1.0mg was administered at Week 9 (n = 16) to allow additional safety data. Some participants were maintained at 0.5mg at Week 9 (n = 4) or had the Week 9 dose deferred (n = 2) due to tolerability or scheduling factors. [b]Adverse event means any untoward medical occurrence associated with the use of an intervention in humans, whether or not considered intervention-related (21 CFR 312.32 (a)). [c]Participant withdrew after contracting COVID-19. [d]Severity of adverse events are defined as follows: mild (events require minimal or no treatment and do not interfere with the participant's daily activities); moderate (events result in a low level of inconvenience or concern with the therapeutic measures, events may cause some interference with functioning); severe (events interrupt a participant's usual daily activity and may require systemic drug therapy or other treatment, events are usually potentially life-threatening or incapacitating, of note, the term "severe" does not necessarily equate to "serious").

© 2025 Hendershot CS et al. *JAMA Psychiatry*.

eTable2a-b. Full results for all models examining laboratory self-administration.

2a. Residualized Change Models

	g-EtOH Post-treatment					Peak BrAC				
Predictors	*B*	*β*	*CI*	CI_{beta}	*p*	*B*	*β*	*CI*	CI_{beta}	*p*
(Intercept)	47.07	0.00	-15.73 – 109.87	-0.36 – 0.36	0.134	0.10	0.00	0.00 – 0.21	-0.36 – 0.36	**0.042**
Baseline	0.35	0.31	-0.12 – 0.83	-0.10 – 0.72	0.135	0.18	0.17	-0.27 – 0.63	-0.26 – 0.61	0.421
Sex	-7.18	-0.13	-30.69 – 16.33	-0.54 – 0.29	0.532	-0.02	-0.21	-0.06 – 0.02	-0.63 – 0.21	0.314
Condition	-25.32	-0.48	-44.87 – -5.76	-0.85 – -0.11	**0.014**	-0.04	-0.46	-0.07 – -0.01	-0.87 – -0.06	**0.026**
Observations	25					25				
R^2 / R^2 adjusted	0.346 / 0.253					0.334 / 0.239				

Note: Peak BrAC = peak breath alcohol concentration; pBrAC Baseline = self-administration outcome at pre-treatment session; g-EtOH = grams of alcohol consumed during the laboratory task; Analyses excluded those who did not consume alcohol during the lab task; B = unstandardized regression coefficient; β = standardized regression coefficient; CI = 95% confidence interval for the unstandardized regression coefficient; CI_{beta} = 95% confidence interval for the standardized regression coefficient. Observations = number of participants with both Pre- and Post-treatment self-administration data in each analysis.

© 2025 Hendershot CS et al. *JAMA Psychiatry*.

2b. Linear Mixed Models

	g-ETOH					Peak BrAC				
Predictors	*B*	*β*	*CI*	CI_{beta}	*p*	*B*	*β*	*CI*	CI_{beta}	*p*
(Intercept)	103.48	0.21	73.33 – 133.64	-0.11 – 0.53	**<0.001**	0.17	0.32	0.12 – 0.22	0.01 – 0.64	**<0.001**
Condition	19.86	-0.47	-8.31 – 48.04	-0.92 – -0.02	0.164	<0.01	-0.66	-0.05 – 0.05	-1.10 – -0.22	0.984
Session	-2.74	-0.05	-16.50 – 11.01	-0.30 – 0.20	0.691	-0.01	-0.10	-0.03 – 0.02	-0.37 – 0.17	0.469
Sex	-24.57	-0.43	-37.71 – -11.42	-0.65 – -0.20	**<0.001**	-0.04	-0.37	-0.06 – -0.01	-0.59 – -0.15	**0.002**
Condition × Session	-23.87	-0.43	-43.03 – -4.70	-0.78 – -0.09	**0.016**	-0.02	-0.24	-0.06 – 0.01	-0.61 – 0.14	0.210
**Random Effects**										
σ^2	333.91					11.34				
τ_{00}	173.92					3.11				
ICC	0.34					0.22				
N	44					44				
Observations	69					69				
Marginal R^2 / Conditional R^2	0.317 / 0.551					0.281 / 0.436				

© 2025 Hendershot CS et al. *JAMA Psychiatry*.

Note: Peak BrAC = peak breath alcohol concentration; g-EtOH = grams of alcohol consumed during the laboratory task; B = unstandardized regression coefficient; β = standardized regression coefficient; CI = 95% confidence interval for the unstandardized regression coefficient; CI_{beta} = 95% confidence interval for the standardized regression coefficient. N = number of participants included in the analysis, i.e., number with Pre-treatment data; Observations = number of participants * number of data/time points per participant; ICC = intraclass correlation.

© 2025 Hendershot CS et al. *JAMA Psychiatry*.

eTable 3. Full results for linear mixed models examining changes in weekly outcomes.

	Drinks per calendar day					Drinks per drinking day				
Predictors	*B*	*β*	*CI*	CI_{beta}	*p*	*B*	*β*	*CI*	CI_{beta}	*p*
(Intercept)	0.39	0.15	-1.25 – 2.03	-0.10 – 0.41	0.639	0.58	0.21	-1.24 – 2.41	-0.02 – 0.44	0.529
Sex	0.26	0.06	-0.53 – 1.04	-0.12 – 0.24	0.518	0.16	0.03	-0.67 – 0.99	-0.12 – 0.18	0.705
Baseline	0.70	0.58	0.49 – 0.91	0.41 – 0.76	**<0.001**	0.80	0.67	0.63 – 0.98	0.53 – 0.82	**<0.001**
Condition	-0.52	-0.27	-1.27 – 0.22	-0.63 – 0.09	0.168	-0.86	-0.41	-1.69 – -0.04	-0.73 – -0.09	**0.041**
Week	-0.13	-0.17	-0.18 – -0.08	-0.24 – -0.10	**<0.001**	-0.04	-0.05	-0.12 – 0.03	-0.13 – 0.04	0.278
Condition x Week	-0.00	-0.00	-0.07 – 0.07	-0.09 – 0.09	0.969	-0.02	-0.03	-0.13 – 0.08	-0.14 – 0.08	0.636
**Random Effects**										
σ^2	0.78					1.47				
τ_{00}	1.37					1.43				
ICC	0.64					0.49				
N	48					48				
Observations	394					358				

© 2025 Hendershot CS et al. *JAMA Psychiatry*.

Marginal R^2 / Conditional R^2	0.408 / 0.785	0.496 / 0.745

	Drinking days					Heavy drinking days				
Predictors	*Incidence Rate Ratios*	*β*	*CI*	*CI_{beta}*	*p*	*Incidence Rate Ratios*	*β*	*CI*	*CI_{beta}*	*p*
(Intercept)	2.09	3.90	1.19 – 3.66	3.35 – 4.55	**0.010**	0.36	0.97	0.10 – 1.32	0.62 – 1.53	0.122
Sex	0.99	0.99	0.78 – 1.25	0.90 – 1.10	0.917	1.09	1.04	0.53 – 2.24	0.76 – 1.41	0.822
Baseline	1.18	1.30	1.10 – 1.27	1.16 – 1.44	**<0.001**	1.55	2.08	1.28 – 1.87	1.51 – 2.87	**<0.001**
Condition	0.98	0.90	0.77 – 1.26	0.73 – 1.12	0.894	0.84	0.64	0.43 – 1.64	0.34 – 1.22	0.606
Week	0.95	0.89	0.93 – 0.98	0.83 – 0.95	**0.001**	0.97	0.93	0.94 – 1.01	0.84 – 1.03	0.189
Condition x Week	0.98	0.95	0.94 – 1.02	0.86 – 1.05	0.275	0.93	0.84	0.88 – 1.00	0.71 – 0.99	**0.040**
**Random Effects**										
σ^2	0.23					0.83				
τ_{00}	0.10					0.99				
ICC	0.31					0.55				
N	48					48				

© 2025 Hendershot CS et al. *JAMA Psychiatry*.

Observations	394	394
Marginal R^2 / Conditional R^2	0.213 / 0.455	0.268 / 0.667

	Craving					Cigarettes per day				
Predictors	*B*	*β*	*CI*	CI_{beta}	*p*	*B*	*β*	*CI*	CI_{beta}	*p*
(Intercept)	6.07	0.21	1.66 – 10.47	-0.03 – 0.45	**0.007**	-4.33	0.01	-7.98 – -0.68	-0.11 – 0.12	**0.021**
Sex	0.11	0.01	-2.18 – 2.40	-0.16 – 0.17	0.925	2.01	0.07	-0.20 – 4.22	-0.01 – 0.14	0.074
Baseline	0.55	0.52	0.37 – 0.74	0.35 – 0.69	**<0.001**	0.96	0.97	0.88 – 1.05	0.88 – 1.05	**<0.001**
Condition	-2.93	-0.39	-5.16 – -0.70	-0.73 – -0.06	**0.010**	1.71	0.01	-0.55 – 3.96	-0.17 – 0.19	0.136
Week	-0.68	-0.29	-0.86 – -0.49	-0.37 – -0.21	**<0.001**	0.01	0.00	-0.20 – 0.22	-0.04 – 0.05	0.894
Condition x Week	0.15	0.06	-0.11 – 0.40	-0.05 – 0.17	0.261	-0.43	-0.10	-0.72 – -0.13	-0.16 – -0.03	**0.005**
**Random Effects**										
σ^2	10.75					3.35				
τ_{00}	11.09					1.93				
ICC	0.51					0.37				

© 2025 Hendershot CS et al. *JAMA Psychiatry*.

N	48	13
Observations	395	98
Marginal R^2 / Conditional R^2	0.393 / 0.701	0.959 / 0.974

Note: B = unstandardized regression coefficient; β = standardized regression coefficient; CI = 95% confidence interval for the unstandardized regression coefficient; CI_{beta} = 95% confidence interval for the standardized regression coefficient. N = number of participants included in the analysis; Observations = number of participants * number of data/time points per participant; ICC = intraclass correlation.

© 2025 Hendershot CS et al. *JAMA Psychiatry*.

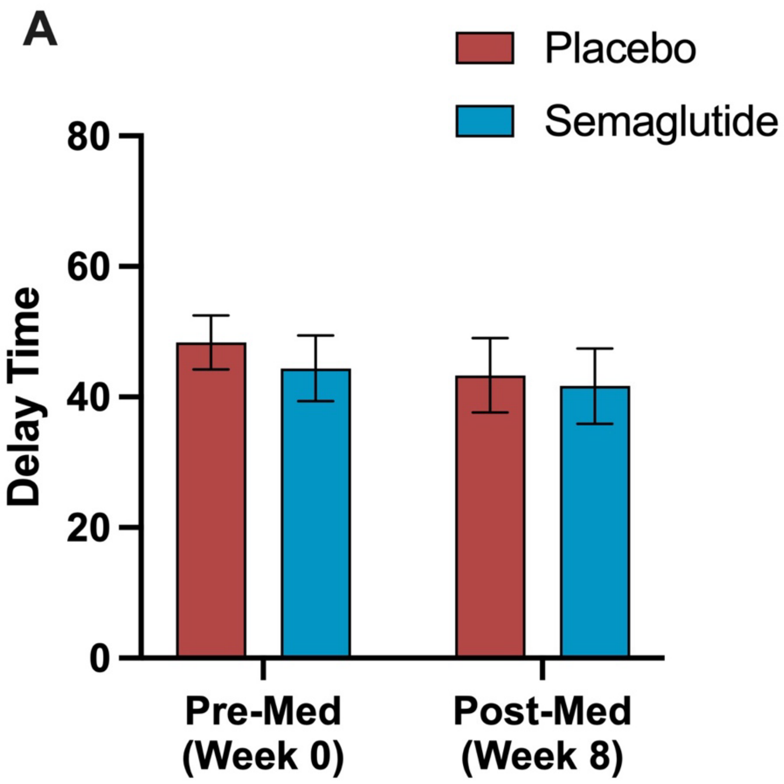

eFigure 1. A. Duration of elective delay (minutes) in laboratory consumption by medication group and time point; Pre-Med = prior to medication; Post-Med = conducted at medication dose of 0.5mg/week. Pre-Med: N=48 (Placebo: N=24; Semaglutide: N=24); Post-Med: N=41 (Placebo N=19; Semaglutide N=22).

© 2025 Hendershot CS et al. *JAMA Psychiatry*.

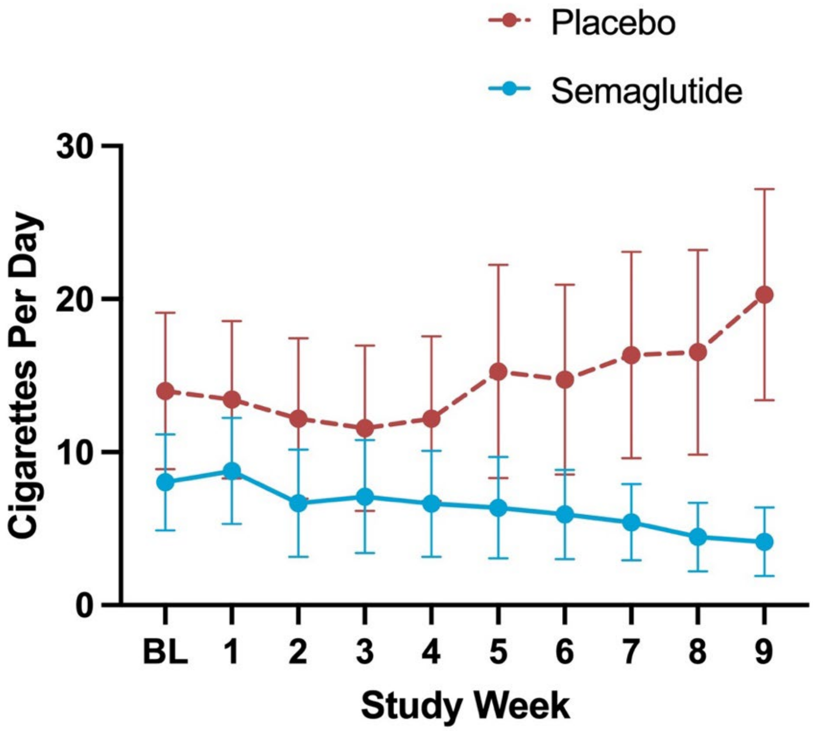

eFigure 2. Mean cigarettes per day by study week in semaglutide and placebo groups (n=13 randomized). The linear mixed model identified a significant treatment-by time interaction (*p*=.005; CI[-0.72, -0.13]). One participant in the placebo group reported a high level of cigarettes per day, contributing to increased variability in the placebo group. Sensitivity analyses confirmed that the interaction effect remained significant when removing this participant from the model (see eSupplement).

© 2025 Hendershot CS et al. *JAMA Psychiatry*.

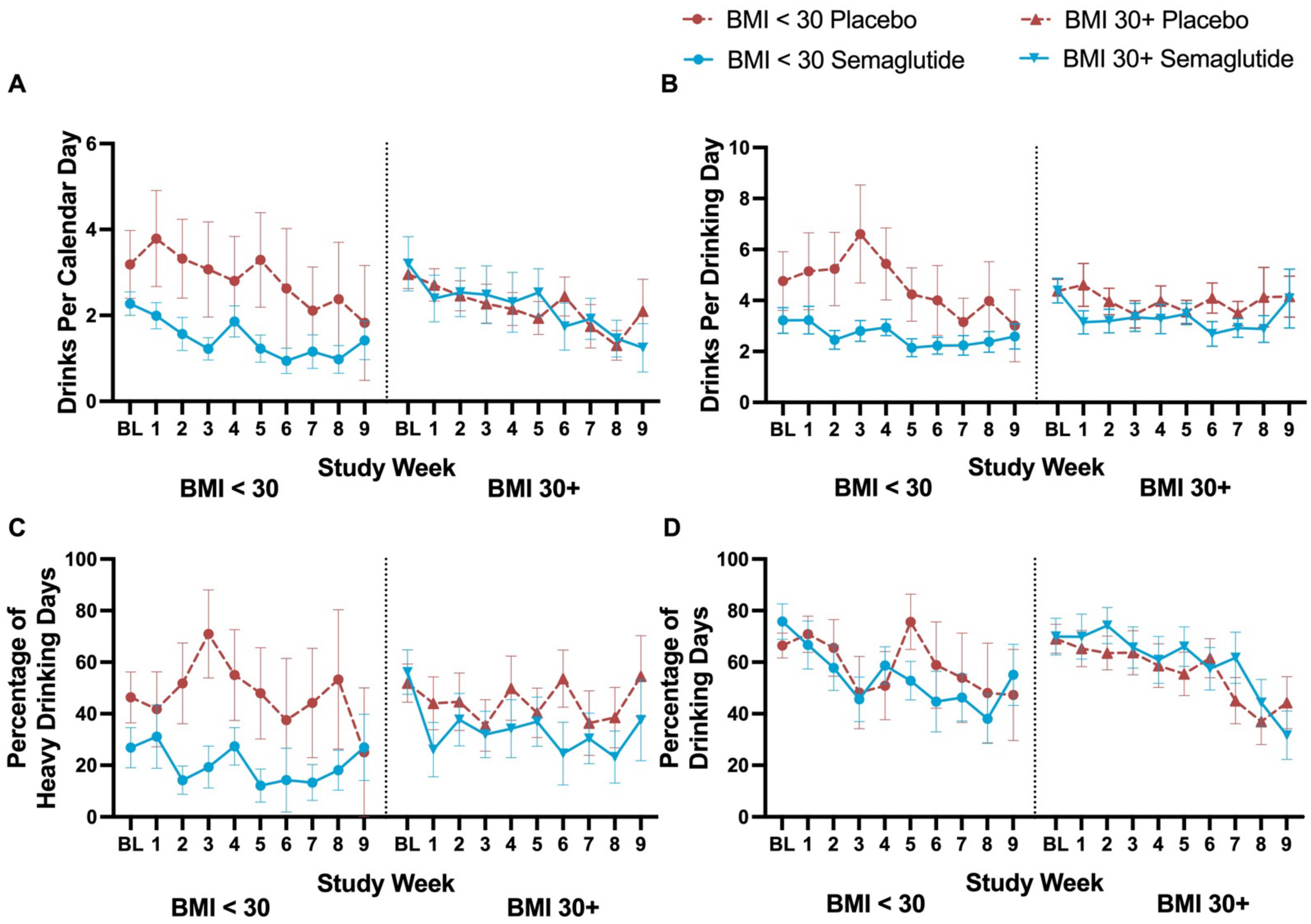

eFigure 3. Descriptive comparison of drinking outcomes by baseline body mass index (BMI). Descriptive subgroup comparison of weekly drinks per calendar day (A), drinks per drinking day (B), percentage of heavy drinking days (C), and percentage of drinking days (D) in participants with body mass index (BMI) of <30 vs. 30+. See manuscript Table 1 for distribution of BMI ranges.

© 2025 Hendershot CS et al. *JAMA Psychiatry*.

References

1. Sheehan D, Janavs J, Baker R, Harnett-Sheehan K, Knapp E, Sheehan M. Mini international neuropsychiatric interview. *Tampa: University of South Florida*. 1994;

2. First M, Williams J, Karg R, Spitzer R. Structured clinical interview for DSM-5—Research version (SCID-5 for DSM-5, research version; SCID-5-RV). *Arlington, VA: American Psychiatric Association*. 2015:1-94.

3. Hendershot CS, Wardell JD, Samokhvalov AV, Rehm J. Effects of naltrexone on alcohol self-administration and craving: meta-analysis of human laboratory studies. *Addict Biol*. Nov 2017;22(6):1515-1527. doi:10.1111/adb.12425

4. McKee SA, Verplaetse TL. A novel human laboratory alcohol self-administration paradigm for medication screening: Modeling the ability to resist drinking and heavy drinking. *Drug Alcohol Depend Rep*. Sep 2022;4doi:10.1016/j.dadr.2022.100085

5. Brick J. Standardization of alcohol calculations in research. *Alcoholism: Clinical and experimental research*. 2006;30(8):1276-1287.

6. Johnson BA, Ait-Daoud N, Roache JD. The COMBINE SAFTEE: a structured instrument for collecting adverse events adapted for clinical studies in the alcoholism field. *Journal of Studies on Alcohol, Supplement*. 2005;(15):157-167.

7. Witkiewitz K, Hallgren KA, Kranzler HR, et al. Clinical Validation of Reduced Alcohol Consumption After Treatment for Alcohol Dependence Using the World Health Organization Risk Drinking Levels. *Alcohol Clin Exp Res*. Jan 2017;41(1):179-186. doi:10.1111/acer.13272

8. Faul F, Erdfelder E, Buchner A, Lang A-G. Statistical power analyses using G* Power 3.1: Tests for correlation and regression analyses. *Behavior research methods*. 2009;41(4):1149-1160.

9. Kuznetsova A, Brockhoff PB, Christensen RHB. lmerTest package: tests in linear mixed effects models. *Journal of statistical software*. 2017;82(13)

10. Team RS. RStudio: integrated development environment for R. *(No Title)*. 2021;

11. Koller M. robustlmm: an R package for robust estimation of linear mixed-effects models. *Journal of statistical software*. 2016;75:1-24.

© 2025 Hendershot CS et al. *JAMA Psychiatry*.

12. Leek J, McShane BB, Gelman A, Colquhoun D, Nuijten MB, Goodman SN. Five ways to fix statistics. *Nature*. 2017;551(7682):557-559.

13. Perneger TV. What's wrong with Bonferroni adjustments. *Bmj*. 1998;316(7139):1236-1238.

14. Rothman KJ. No adjustments are needed for multiple comparisons. *Epidemiology*. 1990;1(1):43-46.

15. Klausen MK, Jensen ME, Møller M, et al. Exenatide once weekly for alcohol use disorder investigated in a randomized, placebo-controlled clinical trial. *JCI insight*. 2022;7(19)

16. Bremmer MP, Hendershot CS. Social Media as Pharmacovigilance: The Potential for Patient Reports to Inform Clinical Research on Glucagon-Like Peptide 1 (GLP-1) Receptor Agonists for Substance Use Disorders. *J Stud Alcohol Drugs*. Jan 2024;85(1):5-11. doi:10.15288/jsad.23-00318

© 2025 Hendershot CS et al. *JAMA Psychiatry*.
