# Supplementary material for: Once-Weekly Semaglutide in Adults With Alcohol Use Disorder: A Randomized Clinical Trial
Source: JAMA Psychiatry. 2025 Feb 12;82(4):395–405. doi: 10.1001/jamapsychiatry.2024.4789 (PMC11822619; doi:10.1001/jamapsychiatry.2024.4789)
Supplement: Supplement 3. — Data sharing statement [file jamapsychiatry-e244789-s003.pdf]

Data Sharing Statement

Data

Additional Information: ClinicalTrials.gov NCT05520775
Data available: Yes
Data types: Deidentified participant data
How to access data: Data will be made available upon reasonable request with appropriate agreements (e.g., data sharing agreement) by contacting the corresponding author.
When available: beginning date: 04-12-2025

Supporting Documents

Document types: Informed consent form
How to access documents: https://clinicaltrials.gov/study/NCT05520775
When available: With publication

Additional Information

Who can access the data: Data will be made available to researchers whose proposed use of the data has been approved, and provided a data sharing agreement is in place.
Types of analyses: Data will be made available for defined secondary data analysis or meta-analysis projects.
Mechanisms of data availability: Data will be made available with investigator support after completion of a data sharing agreement.
